# Electronically tunable luminescent bis(biaryl-substituted triarylamine) ((bTAA)_2_) electrophores prepared *via* oxidative dimerization of modularly accessible arylated monomers

**DOI:** 10.1039/d6ra04438g

**Published:** 2026-07-06

**Authors:** Regina Kohlbecher, Monika Flörke, David Kempe, Thomas J. J. Müller

**Affiliations:** a Heinrich Heine University Duesseldorf, Faculty of Mathematics and Natural Sciences, Institute of Organic Chemistry and Macromolecular Chemistry Universitätsstrasse 1 Düsseldorf D-40225 Germany ThomasJJ.Mueller@hhu.de

## Abstract

Bis(biaryl-substituted triarylamines) ((bTAA)_2_) are readily synthesized by oxidative homocoupling from modularly accessible biaryl-substituted triarylamine (bTAA) monomers. The reactivity of the intermediary formed radical cations and the regioselectivity of the dimerization can be controlled by varying the electronic nature of the employed functional groups and the biaryl substitution. The preferred coupling positions are predominantly determined by the positioning of the biaryl substituent, yielding a single product for *para*- and *ortho*-substituted dimers. However, for the *meta*-substituted dimers three possible regioisomeric products can be formed and in one case identified by NMR spectroscopy, but isolated as inseparable mixtures. Electron-donating groups facilitate a broader delocalization of electron density, while electron-withdrawing substituents enhance the localization of spin density on the *para*-position of unsubstituted phenyl rings in the radical cations, supported by DFT-calculated spin density distributions as well as cyclic voltammograms of bTAA monomers. The cyclic voltammograms of the dimers exhibit two chemical reversible oxidation processes, *i.e.* typical for multistage Wurster-type redox systems with oxidation potentials dependent on the substitution pattern. Expansion onto strong acceptor moieties additionally induces reversible reduction processes. The photonic properties of the diverse library of (bTAA)_2_ dimers are assessed by experimental (absorption and emission spectroscopy) and theoretical (TD-DFT calculations) investigations. Upon photonic excitation, the dimers exhibit positive emission solvatochromicity ranging from blue to orange emission colors with quantum yields of up to 59% in solution and 24% in the solid state. Highly sterically twisted donor–acceptor-conjugates were additionally investigated in a rigid polymethylmethacrylate (PMMA) matrix resulting in quantum yields of up to 38%.

## Abstract 2

Oxidative dimerization of biaryl triarylamines (bTAA) is controlled by substituents in reactivity and selectivity, as seen in calculations and electroanalytics. (bTAA)_2_ are reversible redox systems with blue to orange emission and high quantum yield.

## Graphical abstract

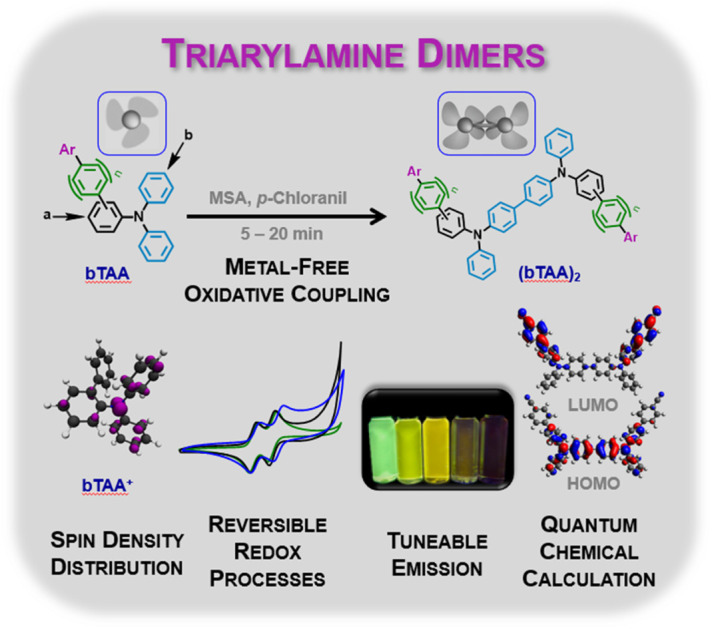

## Introduction

**Fig. 1:**
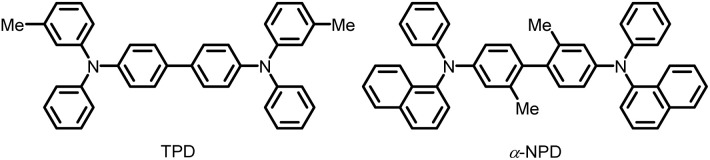
TAA-based hole transport materials TPD and α-NPD.^11,12^

The non-planar propeller-like structure with effective π–π stacking and low reversible oxidation potentials of triarylamine (TAA)-based compounds render them in general promising building blocks for various applications in the fields of photovoltaics,^1^ organic light-emitting diodes (OLED),^2^ sensor technology,^3^ bioimaging,^3,4^ and anti-counterfeiting technologies.^3^ The incorporation of biaryl substituents enhances solubility, and thereby improves the processability and broadens the applicability of such biaryl-substituted triarylamine (bTAA) systems.^5^ In particular, *N*,*N*′-tetraaryl benzidines constitute an important class of organic compounds that can be employed not only as emitter materials in OLEDs^6^ but also, due to their low redox potentials, serve as excellent hole-transport materials (HTM)^7^ in OLEDs, organic solar cells,^5^ perovskite solar cells,^8^ and various sensor systems.^9,10^ Triphenyldiamine (TPD) and naphthylphenyldiamine (NPD) derivatives represent some of the most prominent HTMs (Fig. 1), as they not only show tunable redox potentials, but also enable devices with low turn-on voltages, high hole mobilities, and stable charge-carrier recombination.^11,12^ The incorporation of bulky, asymmetric substituents into the benzidine derivatives suppresses undesired crystallization, affords materials with higher glass-transition temperatures, and ultimately results in substantially longer device lifetimes.^12^ Using tetraarylbenzidines extends the π-conjugated system and introduces tunable steric effects, further enhancing thermal stability and hole mobility.^13^ Especially the molecular linkage, whether symmetric or asymmetric, plays a crucial role in diverse properties of HTMs.^14–16^ Asymmetric structures can increase the molecular dipole moment, improve thermal stability and promote favorable film formation,^14,15^ whereas symmetric structures typically exhibit a higher degree of molecular order and, consequently, enhanced charge carrier mobility.^16^ Biaryl substituents are known to modulate the electronic properties like redox potentials of triarylamines by varying the substitution pattern (*ortho*-, *meta*- and *para*-) and electronic nature, with electron-donating groups typically increasing the separation of successive oxidation waves.^17,18^ Accordingly, biaryl-substituted tetraarylbenzidines are expected to exhibit enhanced delocalization across the extended π-system, reducing the electronic communication between the two triarylamine redox systems, similar like the observed behavior in bridged triarylamine redox systems.^19^ In contrast to classical tetraarylbenzidines such as TPD or NPD (Fig. 1), biaryl-substituted analogues provide the possibility for divers structural modulation, enabling the fine-tuning of electrochemical and photophysical properties.

To access *N*,*N*′-tetraarylbenzidines, metal-free dimerization reactions were established.^20–22^ For instance, the group of Venkatakrishnan successfully developed a metal-free oxidative dimerization of triarylamines (TAA) employing chloranil/H^+^ as a recyclable organic oxidant, enabling an efficient route to *N*,*N*′-tetraarylbenzidines in excellent yields up to 100%.^22^ In contrast to previous reports of organic oxidants at typically low temperature,^23^ chloranil/H^+^ is utilized as a novel system for these transformations as an inexpensive and commercially available oxidant at room temperature.^22^ Modular multicomponent reactions enable highly efficient syntheses of structurally diverse biaryl-substituted triarylamine (bTAA) monomers.^17,18,24,25^ Encouraged by the versatile, highly efficient synthesis of tetraarylbenzidines by Venkatakrishnan^22^ and our extensive work on one-pot syntheses as well as optoelectronic properties of biaryl-substituted triarylamine (bTAA) monomers and their occasionally observed electrochemically induced dimerization,^17,18,24,25^ we report herein on the chemical oxidation of bTAA by Venkatakrishnan's method^22^ to access the corresponding biaryl-substituted benzidine type dimers (bTAA)_2_ and their optoelectronic properties (electrochemistry, UV/vis, emission) as well as electronic structure (TD-DFT).

## Results and discussion

### Synthesis

**Scheme 1:**
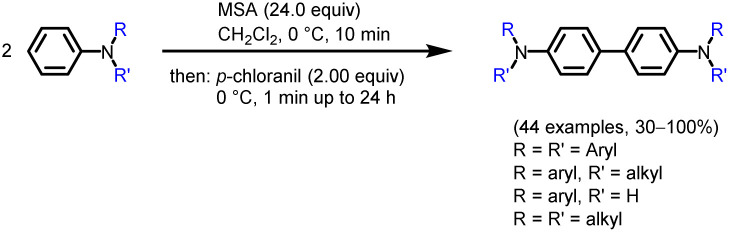
Overview reaction of Venkatakrishnan's metal-free oxidative dimerization of TAA derivatives.

Triphenylamine (TPA) undergoes facile oxidative dimerization to *N*,*N*,*N*′,*N*′-tetraphenylbenzidine (TPB), which can be observed and monitored by cyclic voltammetry.^26^ To synthetically access expanded π-conjugation in *N*,*N*,*N*′,*N*′-tetraphenylbenzidines, we decided to employ the excellent oxidative dimerization protocol of TPA derivatives in the presence of *p*-chloranil and methanesulfonic acid (MSA), reported by Venkatakrishnan (Scheme 1).^22^

**Scheme 2:**
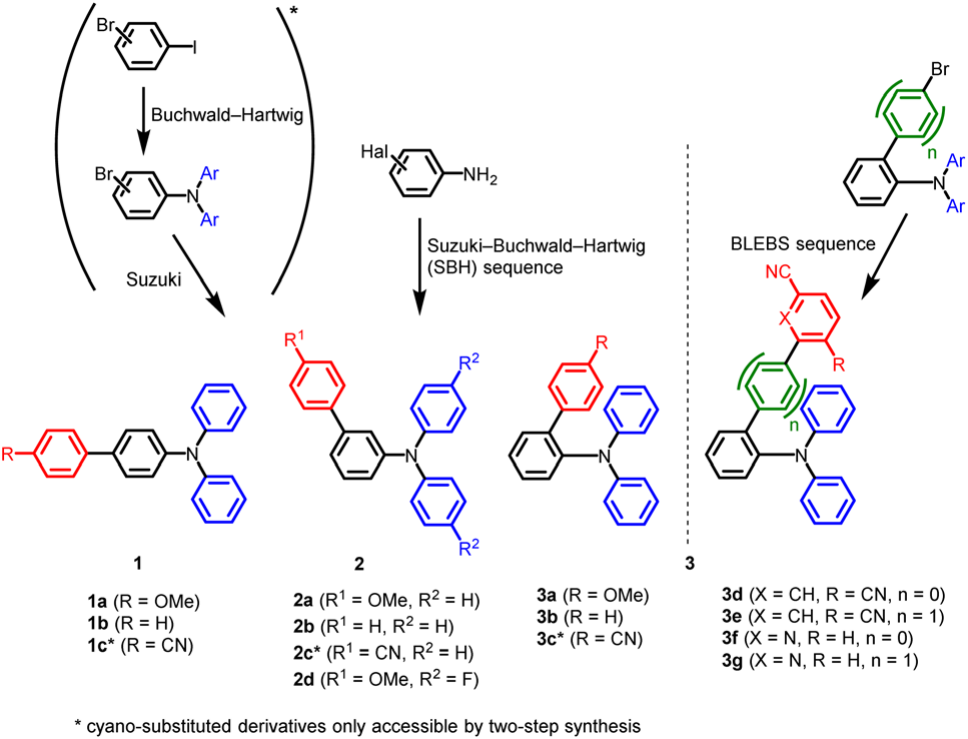
Sequentially palladium-catalyzed consecutive multicomponent reactions (MCR) to the TAA substrates 1, 2, and 3.

Our previous methodological studies have shown that diversely substituted triarylamines (TAAs) 1, 2 and 3 (Scheme 2) are readily accessible in a modular one-pot fashion starting from simple building blocks by applying concise sequentially palladium-catalyzed consecutive multicomponent reactions (MCR) (see SI for synthetic details).^17,18^ A major advantage of this synthetic approach is the catalyst economic use of a single loading of palladium complex, which operates in both steps, the Suzuki arylation and the subsequent two-fold Buchwald–Hartwig amination. In addition, more sophisticated *ortho*-substituted bTAAs 3d–g are accessible from *ortho*-bromo TAAs by bromo-lithium-exchange-borylation-Suzuki sequence (BLEBS),^27^ where after bromo-lithium exchange electrophilic borylation furnishes the corresponding boronate complex that is directly transformed by Suzuki coupling in a one-pot fashion.^24,25^

**Fig. 2:**
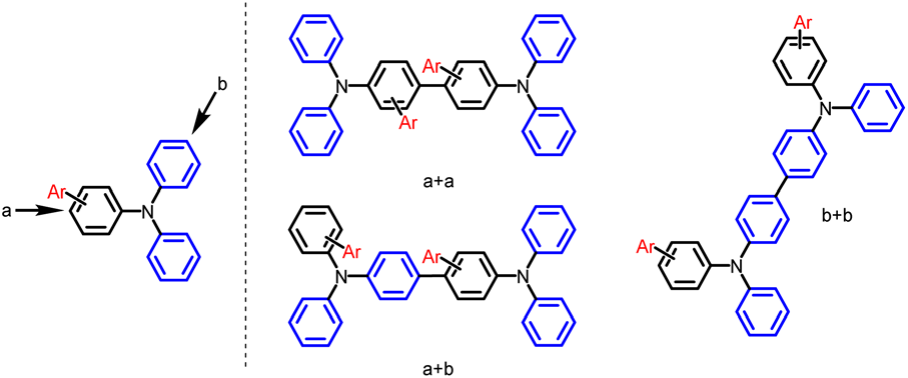
Possible dimerization positions of bTAA.

For the dimerization of our bTAAs 1, 2, and 3 two possible *para*-positions a and b have to be considered, potentially giving rise to three dimerization products through the combinations a + a, a + b and b + b positions (Fig. 2). This can be further supported by quantum chemical calculations of the ground states of the corresponding radical cations, where the localization of the spin density can be assigned to these positions (*vide infra*). While a + a and b + b combinations represent symmetric homodimers, the a + b combination will yield an unsymmetrical homodimer.

**Scheme 3:**
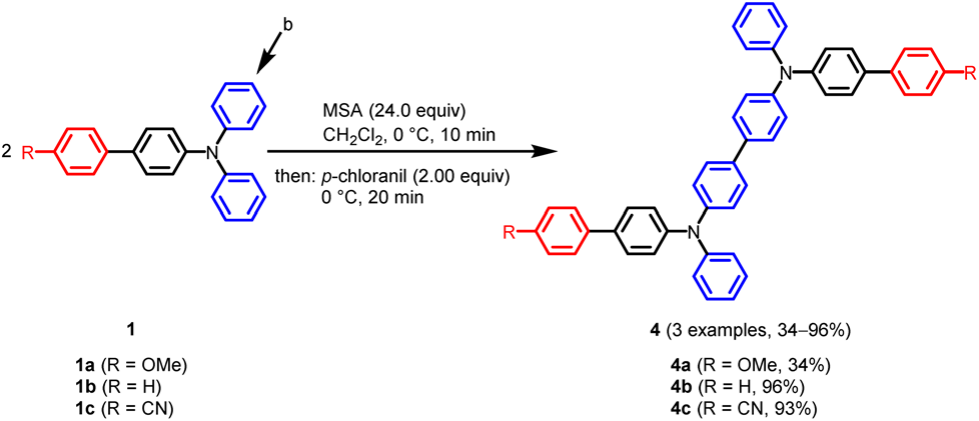
Oxidative homocoupling of *p*-bTAA 1 at position b to *p*-(bTAA)_2_4.

**Scheme 4:**
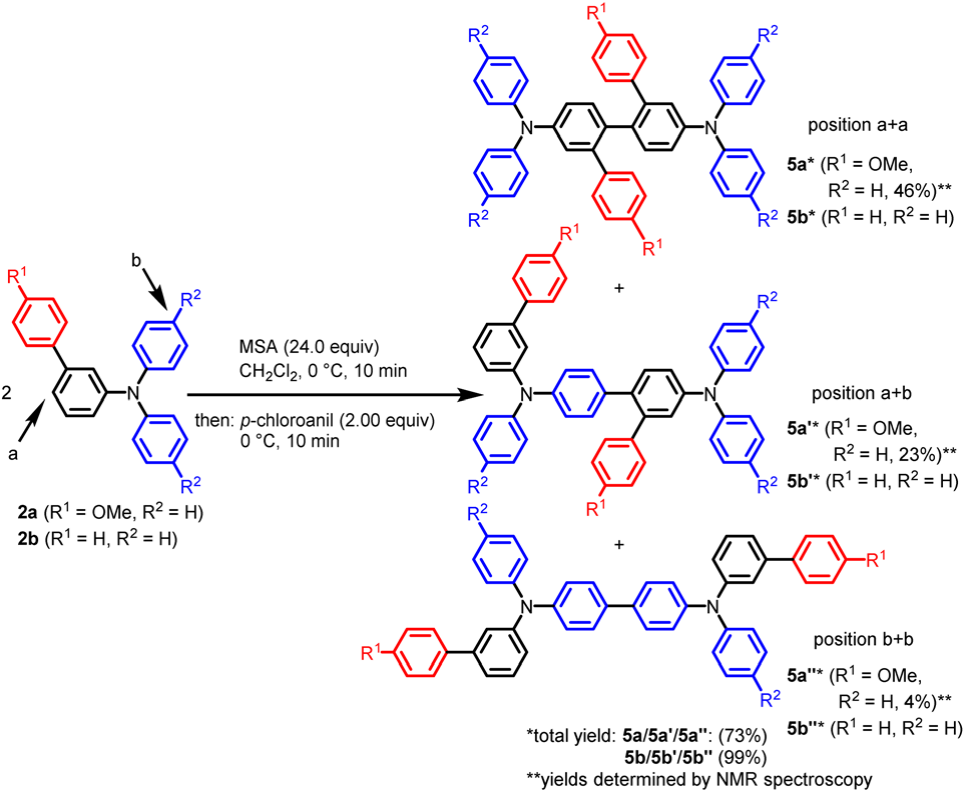
Oxidative coupling of *m*-bTAA 2a and 2b at positions a or b leading to putative product mixtures of *m*-(bTAA)_2_5a/5a′/5a″ and 5b/5b′/5b″.

**Scheme 5:**
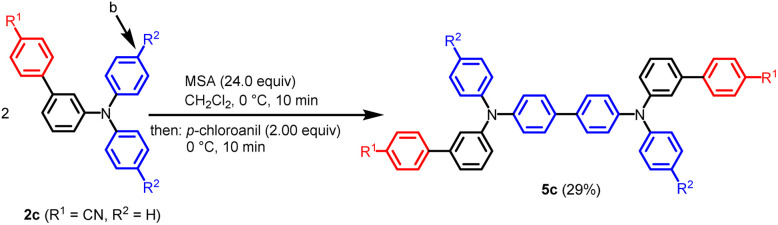
Oxidative coupling of *m*-bTAA 2c at position b to *m*-(bTAA)_2_5c.

**Scheme 6:**
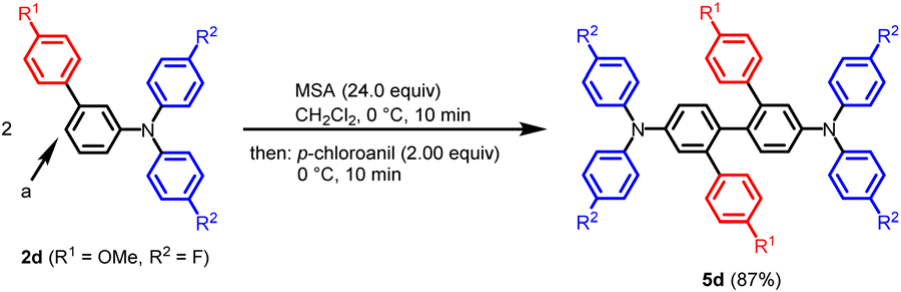
Oxidative coupling of *m*-bTAA 2d at position a to *m*-(bTAA)_2_5d.

**Scheme 7:**
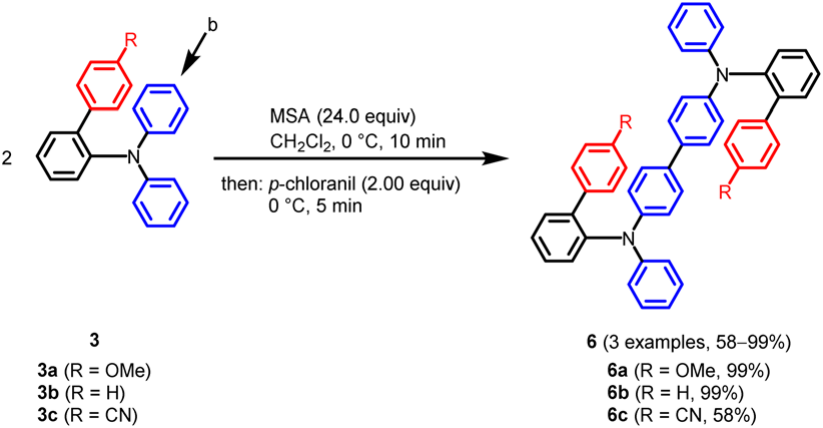
Oxidative coupling of *o*-bTAA 3 at position b to *o*-(bTAA)_2_6.

**Scheme 8:**
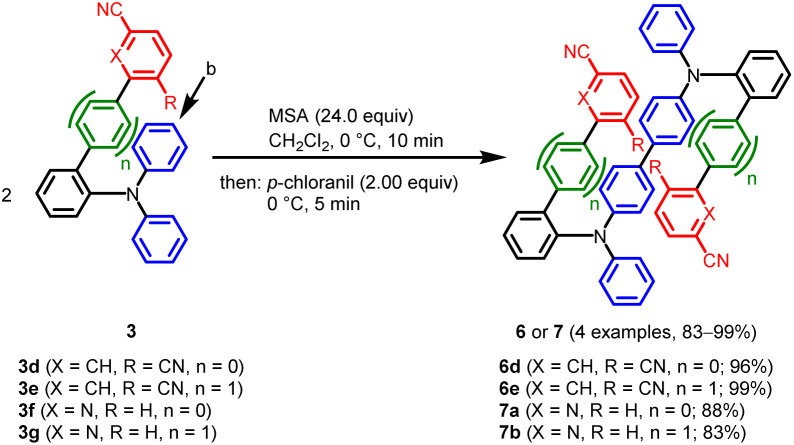
Oxidative coupling of *o*-bTAA 3 at position b to *o*-(bTAA)_2_6d, 6e, 7a and 7b.

With bTAA substrates 1, 2, and 3 in hand, Venkatakrishnan's conditions^22^ give rise to the formation of the corresponding coupling products (bTAA)_2_4–7 in moderate to excellent yields (Schemes 3–8). For *para*-bTAA 1 (*p*-bTAA), where the biaryl substitution blocks position a, dimerization can only occur at position b. Therefore, the coupling process with a reaction time of 20 min affords three dimers 4a–c in yields ranging from 34 to 96% (Scheme 3).

A comparison of the yields shows that the homocoupling of the neutral and electron-deficient *p*-bTAA 1b and 1c is highly efficient. The dimerization of the more electron-rich *p*-bTAA 1a, by contrast, proceeds less efficiently, which can be attributed to the increased stabilization of the generated radical cation in the presence of electron-donating methoxy groups.

In contrast to *p*-bTAA 1, several *meta*-bTAA (*m*-bTAA) 2 have revealed dimerization processes during cyclic voltammetry measurements.^17,18^ Under preparative conditions oxidative homocoupling occurs significantly faster for *meta*-monomers 2, *i.e.* in 10 min. However, the dimerization of *m*-bTAA 2a and 2b yields inseparable mixtures of regioisomers (no separation on TLC can be detected) of the expected products 5a/5a′/5a″ (overall yield of the mixture 73%, ratio 63 : 32 : 5, isomer yields 46% : 23% : 4%) and 5b/5b′/5b″ (overall yield of the mixture 99%). The main product 5a therefore exhibits a decreased yield of 46% due to the reduced reaction selectivity. For the product mixture 5b/5b′/5b″, no diagnostic NMR signal is discernible for determining the mixture composition so the regioselectivity for this reaction remains unkown (Scheme 4, see SI, chpt. 4).

Coupling of monomer 2c, which is controlled by the presence of an electron-withdrawing substituent, occurs at position b due to a diminished electron density at position a, and leads to isolation of product (*m*-bTAA)_2_5c as the only structurally characterized product (Scheme 5). Although the observed regioselectivity is consistent with the electronic influence of the cyano substituent, the isolated yield of 29% is comparatively low. Following oxidative dimerization of 2c, a minor by-product is detected (by TLC). However, it is obtained only in trace amounts and could not be characterized unambiguously. Consequently, the origin of the reduced yield cannot be conclusively established, but may arise from competing side reactions and/or decomposition processes under the oxidative reaction conditions.

With a *m*-bTAA bearing substituents on both R^1^ and R^2^ targeted oxidative coupling should be induced at position a (Scheme 6). However, in agreement with the cyclic voltammetry (CV) measurements of this monomer,^18^ no dimerization process can be observed for the *m*-bTAA bearing a methoxy group at both substituents R^1^ and R^2^. In contrast, dimerization has been observed during CV measurements for monomer 2d (R^1^ = OMe, R^2^ = F).^18^ Dimer 5d can be isolated with a yield of 87% (Scheme 6). Consequently, position a is accessible by replacing the methoxy groups with weakly electron-withdrawing fluorine atoms at R^2^.

Relative to the *m*-bTAA 2, the oxidative coupling of *ortho*-bTAA (*o*-bTAA) 3 not only proceeds more rapidly (5 min) and with yields between 58–99%, but also without formation of product mixtures (Scheme 7). Dimerization occurs exclusively at position b. In contrast to the coupling of *p*-bTAA 1 and *m*-bTAA 2, the reaction of the *o*-bTAA 3a bearing the electron-donating methoxy group proceeds with high efficiency and a yield of 99%.

Likewise, expansion of the *o*-bTAA systems 3a–c to donor–acceptor-conjugates 3d–g with stronger acceptor moieties, such as dicarbonitriles and cyanopyridines also gives rise to oxidative dimerization at position b with high yields in the range of 83–99% (Scheme 8). To investigate the influence of an π-extension between the donor and acceptor moieties a phenylene bridge is employed, resulting in the dimerization of 3e and 3g to the corresponding homocoupling products in high yields. Due to the highly electron-withdrawing effect of the acceptor moieties no significant side product formation can be observed.

**Scheme 9:**
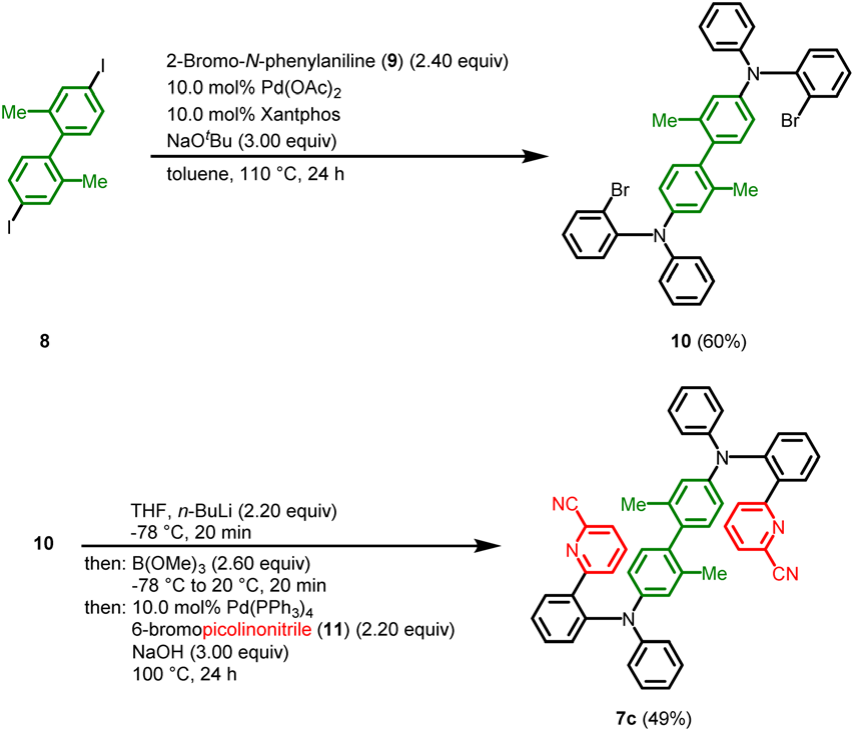
Two-step synthesis of dimer *o*-(bTAA)_2_7c by Buchwald–Hartwig amination of 2-bromo-*N*-phenylaniline (9) to give *o*-bTAA 10, followed by BLEBS reaction of *o*-bTAA 10 with 6-bromopicolinonitrile (11).

To accessing sterically twisted donor–acceptor-based dimers with structural similarity to the efficient hole transport material α-NPD (Fig. 1),^12^ Venkatakrishnan's oxidative homocoupling, which is governed by steric effects,^22^ is not applicable. Therefore, we have chosen an alternative two step strategy, employing Buchwald–Hartwig coupling of a 4,4'diiodobiphenyl followed by BLEBS reaction^27^ with the acceptor moiety containing halide. Starting with 4,4′-diiodo-2,2′-dimethyl-1,1′-biphenyl (8) and 2-bromo-*N*-phenylaniline (9), the two-fold Buchwald–Hartwig amination furnishes the dibromo substituted tetraarylbenzidine 10 in 60% yield, which is then employed as the donor moiety in a separate BLEBS sequence to give donor–acceptor-conjugate 7c in 49% yield along with the monocoupled product, which can be separated but has not been characterized in detail (Scheme 9).

The structures of all dimers 4–7 can be assigned by ^1^H and ^13^C NMR, IR spectroscopy as well as mass spectrometry (see SI). In addition, as proof of the correct structural assignment, compound 7a has been synthesized independently by employing the BLEBS sequence (see SI, chpt. 3.4) similarly to the synthesis of compound 7c, starting from the preformed biphenyl donor framework (Scheme 9). The ^1^H and ^13^C NMR spectra of compound 7a, obtained *via* the two independent approaches (see SI, Fig. S27 and S29), are completely superimposable and confirm the oxidative homocoupling at position b as proposed (*vide supra*). In addition, the molecular composition of new compounds has been determined by elemental analysis or HPLC/HRMS (high-performance liquid chromatography/high-resolution mass spectrometry) (see SI).

### Electrochemical properties

**Fig. 3:**
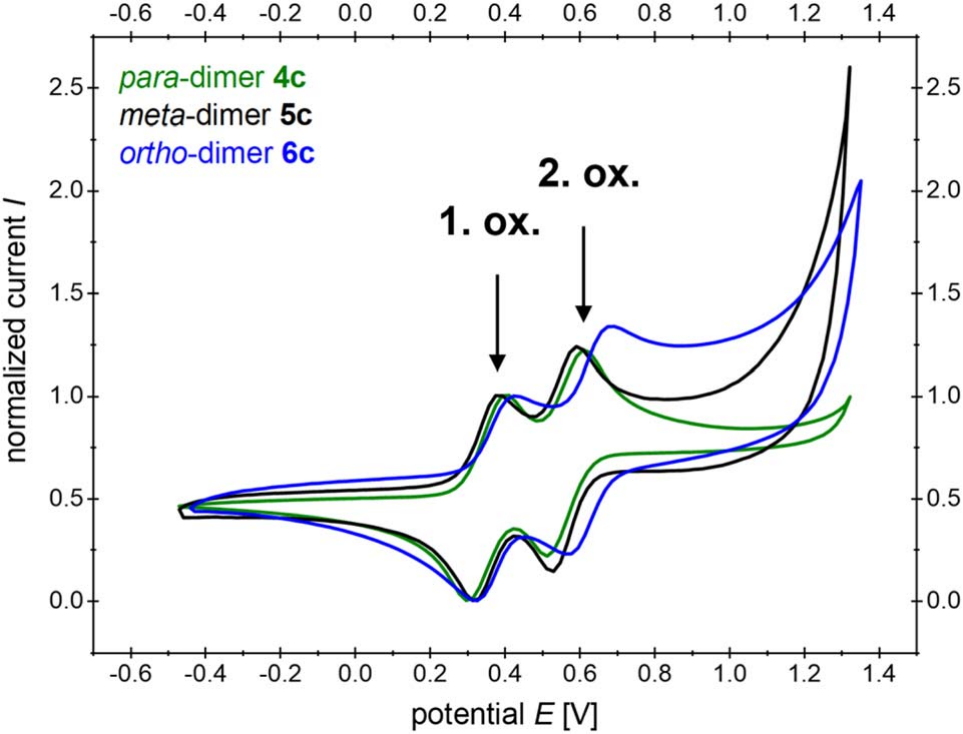
Comparison of the cyclic voltammograms of the monocyano-substituted dimers *p*-(bTAA)_2_4c (green), *m*-(bTAA)_2_5c (black) and *o*-(bTAA)_2_6c (blue) recorded in CH_2_Cl_2_ (*T* = 293 K, *ν* = 100 mV s^−1^, electrolyte: [^*n*^Bu_4_N][PF_6_], Pt working electrode, Pt counter electrode, Ag/AgCl reference electrode, redox standard: decamethylferrocene *E*_0_^0/+1^ = −0.54 V (*vs.* ferrocene *E*_0_^0/+1^ = 0.00 V (ref. 31))).

The electronic ground states of dimers (bTAA)_2_4–7 can be characterized by cyclic voltammetry, which was recorded in dichloromethane solutions at room temperature. The cyclic voltammograms of the dimers exhibit chemical reversible redox processes within the measurement window of dichloromethane (for details see SI, chpt. 5.4) and can be classified as multistage Wurster-type redox systems, including two connected triarylamine redox centers^28–30^ (Fig. 3). The electrochemical data reported for mixtures 5a/5a′/5a″ and 5b/5b′/5b″ represent averaged values rather than molecular constants of individual compounds. Therefore, their data are not discussed in comparison to the pure compounds and exclusively provided in the SI (chpt. 5 and 6).

The first oxidation potentials *E*_0_^0/+1^ of the dimers appear in a range from 0.24 to 0.62 V and the second oxidation potentials *E*_0_^+1/+2^ are found in a significantly narrower range from 0.51 to 0.67 V. These stepwise oxidation processes indicate that one-electron transfers are coupled. Compared to *N*,*N*,*N*′,*N*′-tetraphenylbenzidine (TPB)^26^ the dimers (bTAA)_2_4–7 display the typical behavior of the substance class (Table 1).

**Table 1:** Selected electrochemical properties (*E*_0_^−1/0^, *E*_0_^0/+1^ and *E*_0_^+1/+2^, semiquinone formation constant *K*_SEM_) of dimers 4–7

Compound	*E*_0_^−1/0^ a [V]	*E*_0_^0/+1^ a [V]	*E*_0_^+1/+2^ a [V]	*K*_SEM_ b
TPB^d^	c	0.25	0.50	1.73 × 10^4^
4a	c	0.29	0.51	6.84 × 10^3^
4b	c	0.28	0.51	8.47 × 10^3^
4c	c	0.36	0.56	2.54 × 10^3^
5a/5a′/5a″	See SI^f^	See SI^f^	See SI^f^	See SI^f^
5b/5b′/5b″	See SI^f^	See SI^f^	See SI^f^	See SI^f^
5c	c	0.36	0.56	2.98 × 10^3^
5d	c	0.62	c	e
6a	c	0.24	0.55	1.91 × 10^5^
6b	c	0.29	0.54	1.50 × 10^4^
6c	c	0.34	0.60	2.21 × 10^4^
6d	−2.15	0.43	0.67	1.47 × 10^4^
6e	−2.08	0.26	0.53	3.00 × 10^4^
7a	c	0.33	0.55	5.20 × 10^3^
7b	c	0.29	0.55	2.81 × 10^4^
7c	c	0.44	0.59	3.07 × 10^2^

a Recorded in CH_2_Cl_2_, *T* = 293 K, extrapolation of data with *ν* = 100, 250, 500, and 1000 mV s^−1^ to 0.00 mV s^−1^ (details see SI, chpt. 5.4), electrolyte: [^*n*^Bu_4_N][PF_6_], Pt working electrode, Pt counter electrode, Ag/AgCl reference electrode, 
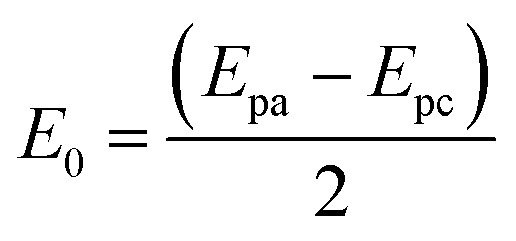
 with redox standard: decamethylferrocene *E*_0_^0/+1^ = −0.54 V (*vs.* ferrocene *E*_0_^0/+1^ = 0.00 mV).^31^

b Semiquinone formation constant 
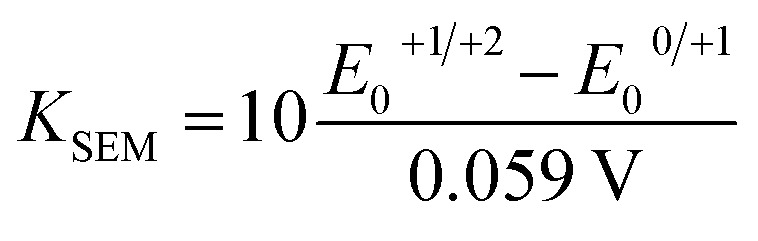
.

c Outside the measuring range.

d Values used from ref. 26 recorded in CH_2_Cl_2_, *T* = 298 K, *ν* = 20 mV s^−1^, electrolyte: [^*n*^Bu_4_N][PF_6_], Pt working electrode, Pt counter electrode, Ag/AgCl reference electrode, 
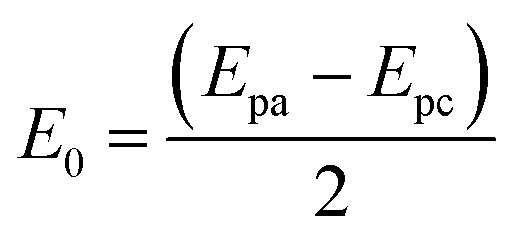
 with redox standard: ferrocene *E*_0_^0/+1^ = 0.00 V.^31^

e Cannot be estimated.

f Values obtained for product mixtures, for details see SI chpt. 5.3.

The *para*-dimers 4 expectedly exhibit a lower *E*_0_^0/+1^ than their monomers 1 (ref. 17) as a consequence of extended delocalization of the radical cation generated in the first oxidation. As a consequence of Coulomb repulsion and reduced electron density, the second oxidation process *E*_0_^+1/+2^ occurs at higher potentials.^32^ The small potential differences are reflected in lower *K*_SEM_ values, which also indicate that the formed radical cations are less stable than those of their monomers 1.^17^ For compound 4a, there is also a third redox potential *E*_0_^+2/+4^ at 1.03 V, which results from the increased electron density by the conjugated *p*-methoxy group and contributes to the overall resonance stabilization of the tetracationic species. This potential appears slightly higher than the second oxidation potential of monomer 1a.^17^ The first oxidation potentials of 4a and 4b are comparable (Table 1). In contrast, the oxidation potentials of dimer 4c are anodically shifted as a consequence of the substitution with strongly electron-withdrawing cyano groups. The influence of biaryl moiety becomes particularly evident when comparing 4b with TPB,^26^ showing only a minor effect on the first oxidation potential (Table 1).

The first oxidation potentials of the *meta*-dimers 5c and 5d are likewise lower than those of their corresponding monomers 2.^18^ From the cyclic voltammogram of 5d, only a broad half-wave potential becomes apparent (width of the peak at half height approximately 390 mV, see SI, Fig. S73). Since the potential difference between the first and the second redox potential and thus the *K*_SEM_ value is generally small for *m*-(bTAA)_2_5c, it is very likely that the two processes of 5d coincide to a single broad signal (width of the peak at half height >90.4 mV (ref. 33)). The data strongly support the presence of two unresolved oxidation processes but do not provide definitive proof (see SI, chpt. 5.5). The cyclic voltammograms of the *meta*-dimers 5c and 5d only partially match with the dimers formed during cyclic voltammetric experiments from monomers 2c, and 2d (see SI, chpt. 5.2.1, Fig. S48). This discrepancy may originate from differences in the product distributions formed under chemical and electrochemical oxidation conditions, the comparison is therefore qualitative. Electrogenerated triarylamine radical cations are formed at the electrode surface and are known to undergo radical cation–radical cation coupling reactions.^34^ In contrast, under bulk chemical oxidation conditions, reactions involving radical cations and neutral monomers cannot be excluded.^35^ Moreover, in addition to the main redox potentials of the monomers, weak and diffuse signals occur, which could be likely due to dimerization at less preferred positions however, without isolation and structural characterization of these species, a definitive assignment is not possible (see SI, chpt. 5.2.1, Fig. S48).

**Fig. 4:**
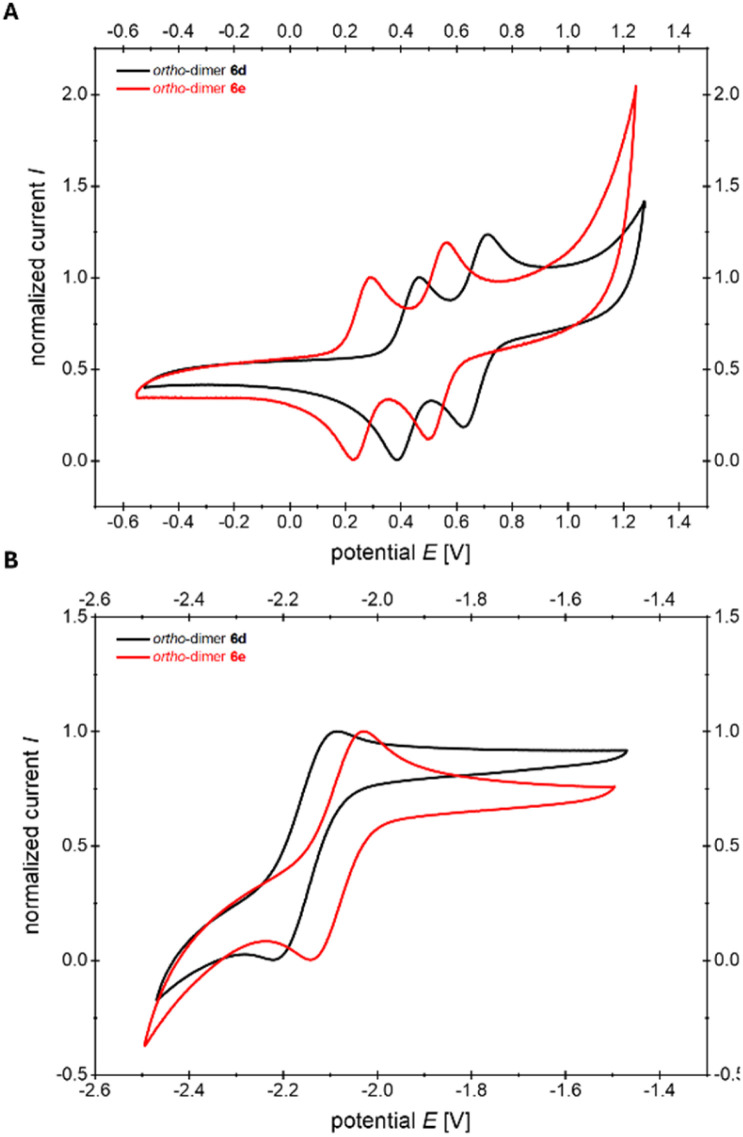
Comparison of the cyclic voltammograms ((A) reversible oxidation and (B) reversible reduction) of the cyano-substituted dimers *o*-(bTAA)_2_6d (black) and π-extended dimer *o*-(bTAA)_2_6e (red) recorded in CH_2_Cl_2_ (*T* = 293 K, *ν* = 100 mV s^−1^, electrolyte: [^*n*^Bu_4_N][PF_6_], Pt working electrode, Pt counter electrode, Ag/AgCl reference electrode, redox standard: decamethylferrocene *E*_0_^0/+1^ = −0.54 V (*vs.* ferrocene *E*_0_^0/+1^ = 0.00 V (ref. 31))).

In contrast, the *ortho*-dimers 6 and 7 are in good agreement with the dimerization signals of the corresponding monomers 3 (see SI, chpt. 5.2.2, Fig. S49 and S50), which clearly indicates that dimerization at position b can be assumed to occur exclusively. In analogy to the *para*-dimers 4, the oxidation potentials of the *ortho*-dimers 6 are shifted anodically caused by diminished donor strength of the substituent at position R. The incorporation of a stronger acceptor moiety in compound 6d causes anodically shifted oxidation potentials, whereas the cyanopyridine acceptor moiety in compound 7a exhibits values comparable to those of 6c due to their similar acceptor strengths (Table 1). Expansion of the π-system by introducing an additional phenylene bridge between the donor and acceptor moiety results for dimers 6 and 7 in cathodically shifted oxidation potentials (Table 1), *i.e.* facilitating the oxidation process (Fig. 4A). Owing to the presence of a strong acceptor moiety, compounds 6d and 6e display a reversible reduction process within the applied measuring window (Fig. 4B).

### Photophysical properties

**Fig. 5:**
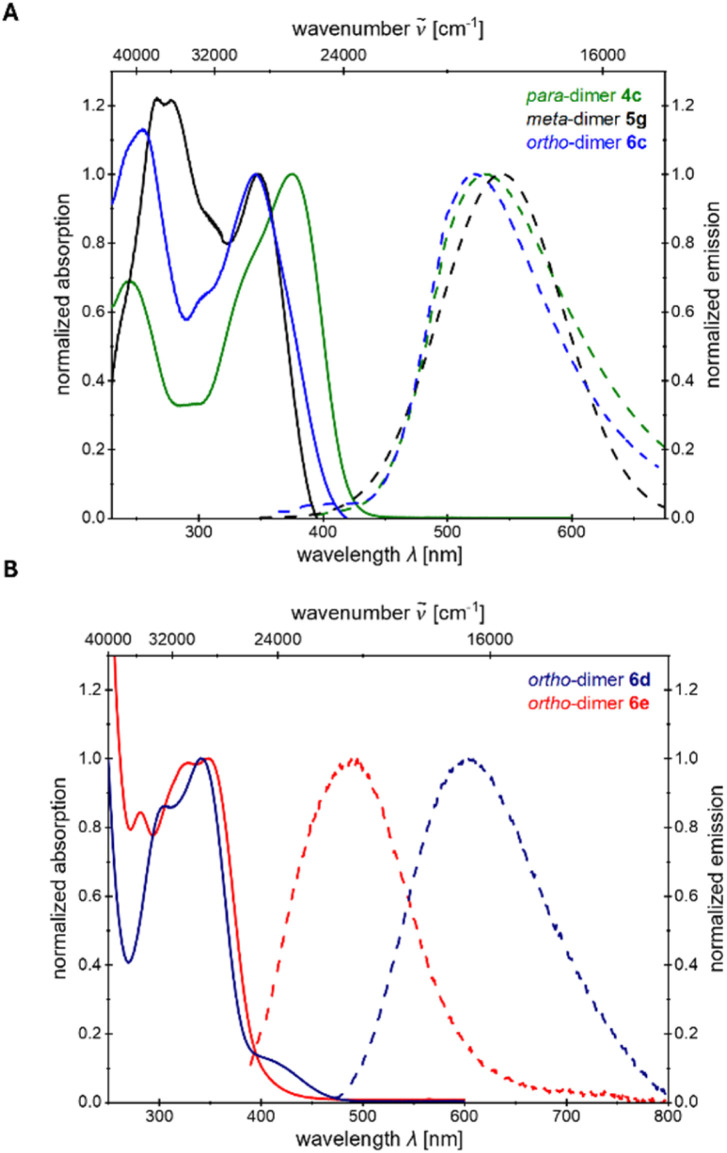
Comparison of the UV/vis absorption and emission spectra of the cyano-substituted dimers *p*-(bTAA)_2_4c (green), *m*-(bTAA)_2_5c (black) and *o*-(bTAA)_2_6c (blue) (A) and dimer *o*-(bTAA)_2_6d (dark blue) as well as the π-extended dimer *o*-(bTAA)_2_6e (red) (B) recorded in CH_2_Cl_2_ (absorption spectra: bold lines, *T* = 293 K, *c* = 10^−5^ M and emission spectra: dashed lines, *T* = 293 K, *c* = 10^−7^ M).

In addition to the ground state, the excited states of the dimers (bTAA)_2_4–7 were studied by steady-state UV/vis absorption and fluorescence spectroscopy. The photophysical data reported for mixtures 5a/5a′/5a″ and 5b/5b′/5b″ represent averaged values rather than molecular constants of individual compounds. Therefore, their data are not discussed in comparison to the pure compounds and are exclusively provided in the SI (Table S8). The absorption spectra in dichloromethane typically show three absorption bands in the UV region (238–304 nm, 300–343 nm, and 332–406 nm) (Table 2), while the solutions appear colorless up to yellow. In contrast to the monomers, the *meta*- and *ortho*-dimers 5, 6 and 7 lack a longest wavelength shoulder but instead display a longest wavelength absorption maximum that is red-shifted compared to the monomers and characterized by higher molar absorption coefficients *ε* (16 700–81 000 M^−1^ cm^−1^) (Fig. 5A).^17,18,24,25^ An exception to this behavior is observed for compounds 6d and 7c. In the case of 6d, the increased acceptor strength gives rise to a longest wavelength shoulder at 406 nm with a low molar absorption coefficient *ε* of 4300 M^−1^ cm^−1^ (Fig. 5B and Table 2). Similarly, compound 7c exhibits a longest wavelength shoulder, which can be attributed to its highly twisted molecular structure (Table 2). Upon photoexcitation, dimers 4–7 reveal fluorescence maxima between 403 and 604 nm, covering the emission range from blue to orange color (Table 2).

**Table 2:** Selected photophysical properties (absorption maxima with absorption coefficients *ε* and emission maxima with fluorescence quantum yields *Φ*_F_ in solution, Stokes shifts Δ*ṽ*_s_, emission maxima in the solid state and in a PMMA matrix with fluorescence quantum yields *Φ*_F_) of dimers 4–7

Compound	*λ*_max*,*abs_ a [nm] (*ε* [M^−1^ cm^−1^])	*λ*_max,em(l)_ b [nm] (*Φ*_F_)^c^	Δ*ṽ*_s_^d^ [cm^−1^]	*λ*_max,em(s)_ e [nm] (*Φ*_F_)^f^	*λ*_max,em(PMMA)_ g [nm] (*Φ*_F_)^h^
4a	247 (42 600), 333 (75 000), 367 (sh, 68 800)	417 (0.59)	3200	436 (0.05)	nd^i^
4b	239 (56 100), 333 (81 000), 363 (sh, 80 600)	414 (0.57)	3400	496 (0.04)	nd^i^
4c	245 (37 700), 331 (sh, 38 300), 376 (60 800)	531 (0.21)	7700	524 (0.04)	nd^i^
5a/5a′/5a″	See SI^j^	See SI^j^	See SI^j^	See SI^j^	nd^i^
5b/5b′/5b″	See SI^j^	See SI^j^	See SI^j^	See SI^j^	nd^i^
5c	266 (63 000), 280 (sh, 63 000), 302 (sh, 16 700), 348 (sh, 51 500)	532 (0.02)	11 700	488 (0.23)	nd^i^
5d	275 (42 400), 300 (sh, 36 800), 332 (29 900)	423 (0.36)	6500	397 (0.03)	nd^i^
6a	253 (42 000), 318 (38 600), 355 (55 400)	403 (0.39)	3400	406 (0.04)	nd^i^
6b	238 (37 900), 319 (29 700), 355 (41 800)	405 (0.28)	3500	392 (0.22)	nd^i^
6c	255 (66 300), 303 (37 400), 346 (58 300)	523 (0.11)	9800	452 (0.11)	nd^i^
6d	304 (sh, 26 500), 343 (35 000), 406 (sh, 4300)	604 (0.01)	8100	541 (0.23)	520 (0.31)
6e	282 (35 800), 326 (42 000), 349 (42 600)	492 (<0.01)	8300	547 (0.06)	517 (0.38)
7a	273 (sh, 38 000), 294 (43 500), 344 (57 800)	543 (0.02)	10 700	475 (0.24)	474 (0.27)
7b	277 (sh, 48 700), 294 (55 800), 348 (35 200)	558 (0.03)	10 800	476 (0.21)	464 (0.30)
7c	272 (sh, 43 700), 301 (58 800), 363 (sh, 13 400)	516 (0.08)	8200	495 (0.20)	464 (0.34)

a Recorded in CH_2_Cl_2_, *T* = 293 K, *c* = 10^−5^ M.

b Recorded in CH_2_Cl_2_, *T* = 293 K, *c* = 10^−7^ M.

c Absolute quantum yields recorded in CH_2_Cl_2_ using an integration sphere (details, see SI), *T* = 293 K, *c* = 10^−6^ M.

d Δ*ṽ*_s_ = 1/*λ*_max_,_abs_ − 1/*λ*_max_,_em_.

e Recorded in solid state at *T* = 293 K.

f Absolute quantum yields recorded in the solid state using an integration sphere (powder sample, details see SI), *T* = 293 K.

g Recorded in PMMA matrix, *T* = 293 K, *c* = 1 wt%.

h Absolute quantum yields recorded in PMMA matrix using an integration sphere (details see SI), *T* = 293 K, *c* = 1 wt%.

i Not determined.

j Values obtained for product mixtures, for details see SI Table S8.

In solution, their emission maxima are red-shifted relative to those of monomers 1–3 with analogous substitution patterns and likewise exhibit substituent dependence.^17,18,24,25^ The emission maxima of the cyano-substituted compounds 4c, 5c, and 6c are significantly bathochromically shifted relative to the other dimers 4a–b and 6a–b, with the *meta*-substituted compound 5c exhibiting the largest red shift and largest Stokes shift (Fig. 5A and Table 2). Increasing the acceptor strength in compounds 6d and 7a causes a further red-shift of the emission maxima, reaching up to 604 nm (Table 2). The introduction of a π-bridge in compound 6e, however, induces a pronounced hypsochromic shift of the emission maximum relative to 6d, which may be attributed to excitation from a different electronic state (Fig. 5B). In alignment, compound 6e also exhibits a lower sensitivity toward solvent effects compared to 6d (see SI, Fig. S113). The absolute fluorescence quantum yields *Φ*_F_ in solution with up to 59% is highest for the *para*-dimers 4a and 4b and significantly lower for the cyano- and cyanopyridine-substituted dimers 5c, 6c–e and 7 (Table 2).

All dimers 4–7 emit in the solid state with emission maxima ranging from 392 to 547 nm. Compound 6d (541 nm) and 6e (547 nm) display similar solid state emission maxima, suggesting relaxation from comparable excited state species, in contrast to their distinctly different photophysical behavior in dichloromethane solution (Table 2 and SI Fig. S112). The absolute fluorescence quantum yield *Φ*_F_ attains values of up to 24% in the solid state, exceeding those of the *meta*- and *ortho*-monomers 2 and 3a–c but falling below those of the *para*-monomers 1 and *ortho*-monomers 3d–g.^17,18,24,25^ The donor–acceptor-based dimers 6d, 6e and 7 were additionally investigated when embedded in a rigid PMMA matrix, where they emit in the wavelength range from 464 to 520 nm. Compared to the solid state, the emission maxima exhibit hypsochromic shifts, accompanied by a significant increase in the absolute fluorescence quantum yield *Φ*_F_, reaching values of up to 38% (Table 2).

**Fig. 6:**
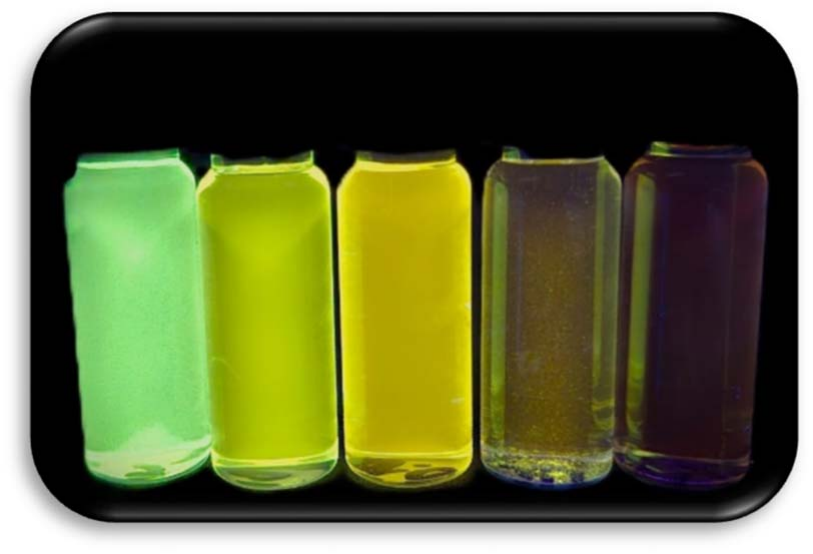
Emission solvatochromism of compound 6d recorded in different solvents (from left to right: cyclohexane, toluene, 1,4-dioxane, ethyl acetate and dichloromethane, *λ*_exc_ = 365 nm, *T* = 293 K, *c* = 10^−7^ M).

**Fig. 7:**
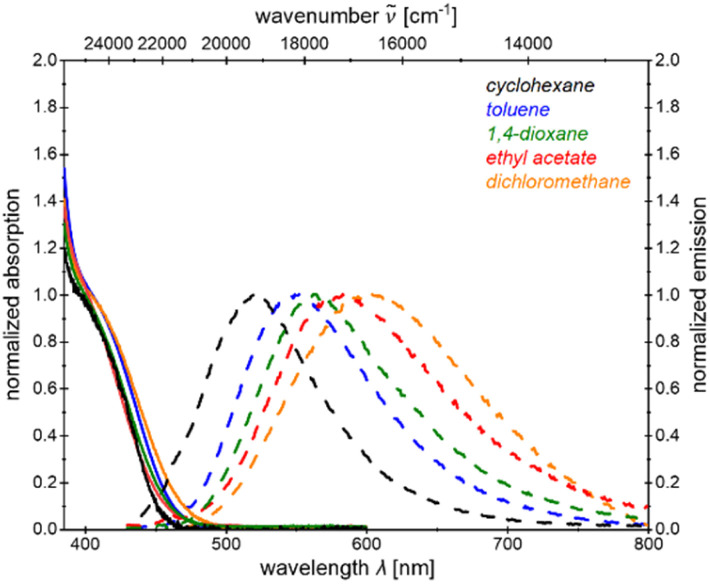
UV/vis absorption and emission spectra of the cyano-substituted dimer *o*-(bTAA)_2_6d in different solvents: cyclohexane (black), toluene (blue), 1,4-dioxane (green), ethyl acetate (red) and dichloromethane (orange). Recorded in different solvents (absorption spectra: bold lines, *T* = 293 K, *c* = 10^−5^ M and emission spectra: dashed lines, *T* = 293 K, *c* = 10^−7^ M).

For several *ortho*-linked dimers 6 and 7, pronounced positive emission solvatochromism is readily discernible with the naked eye. Consequently, absorption and emission spectra have been recorded in solvents of different polarities for compound 6d, with emission color spanning from green in cyclohexane to red in dichloromethane (Fig. 6 and 7).

While the longest wavelength absorption shoulder between 401–413 nm is not significantly affected by the solvent polarity, the emission spectra experience a strong bathochromic shift of their maxima from 520 to 604 nm with increasing solvent polarity. The emission shift is also accompanied by an increase in the Stokes shifts (5300–8100 cm^−1^) and a broadening of the emission bands, with FWHM (full width at half maximum) values ranging from 3400 to 4550 cm^−1^ (Table 3).

**Table 3:** Selected photophysical properties (absorption maxima in solution with absorption coefficients *ε* and emission maxima in solution with fluorescence quantum yields *Φ*_F_, Stokes shifts Δ*ṽ*_s_ and FWHM of emission band) of dimer 6d

Solvent	*λ*_max,abs_ a [nm] (*ε* [M^−1^cm^−1^])	*λ*_max,em(l)_ b [nm] (*Φ*_F_)^c^	Δ*ṽ*_s_^d^ [cm^−1^]	FWHM^e^ [cm^−1^] ([nm])
Cyclohexane	230 (sh, 35 100), 300 (sh, 14 300), 338 (16 900), 408 (sh, 2200)	520 (0.13)	5300	3400 (92)
Toluene	302 (sh, 31 400), 345 (35 000), 413 (sh, 3800)	552 (0.04)	6100	3640 (111)
1,4-Dioxane	306 (sh, 60 700), 341 (72 000), 401 (sh, 10 100)	562 (0.05)	7100	3670 (116)
Ethyl acetate	298 (sh, 42 300), 341 (52 000), 405 (sh, 6700)	583 (0.01)	7500	4000 (136)
Dichloromethane	304 (sh, 26 500), 343 (35 000), 406 (sh, 4300)	604 (0.01)	8100	4550 (166)

a Recorded in different solvents, *T* = 293 K, *c* = 10^−5^ M.

b Recorded in different solvents, *T* = 293 K, *c* = 10^−7^ M.

c Absolute quantum yields recorded using an integration sphere (details see SI), *T* = 293 K, *c* = 10^−6^ M.

d Δ*ṽ*_s_ = 1/*λ*_max,abs_ − 1/*λ*_max,em_.

e Recorded at *T* = 293 K.

Due to the increasing Stokes shifts, the emission solvatochromism can be explained by a significant dipole moment change of the fluorophore upon excitation (*µ*_e_ > *µ*_g_) and energetic stabilization of the S_1_ state by solvation.^36^ Compound 6d is designed as a donor–acceptor conjugate, inherently featuring an electronic separation of electron density between the distinct molecular moieties.^37^ Lippert–Mataga analysis provides a linear dependency (*r*^2^ = 0.84), assuming spherical dipole geometry (see SI, chpt. 8, Fig. S122). Deviations from linearity are presumably due to non-spherical cavity of the chromophore, only dipole–dipole interactions are considered, as well as the solute polarizability is neglected. The Onsager radius *a*, approximates the molecular volume in solution and was calculated for each solvent from the optimized ground state structure in the gas phase (see SI, chpt. 8.1).^38–43^ The pronounced effect on the observed changes of dipole moment Δ*µ* can be calculated to 18.1 debye. The pronounced bathochromic emission shifts, broadening of the emission bands, increasing Stokes shifts, and strong changes of the of dipole moment Δ*µ* collectively indicate a dipolar excited state with charge-transfer character.^44^

### Quantum chemical calculations

#### Calculated spin densities

Understanding of the spin density distribution in the bTAA monomers is crucial to determine the favored position, where the dimerization reaction takes place. Therefore, the quantum chemically calculated spin density in the fundamental bTAA monomers 1, 2, 3a–c was assessed using Gaussian 16 (ref. 45) (uPBE1PBE functional,^46,47^ Pople basis set 6-31+G**^48^).^17,18^

**Fig. 8:**
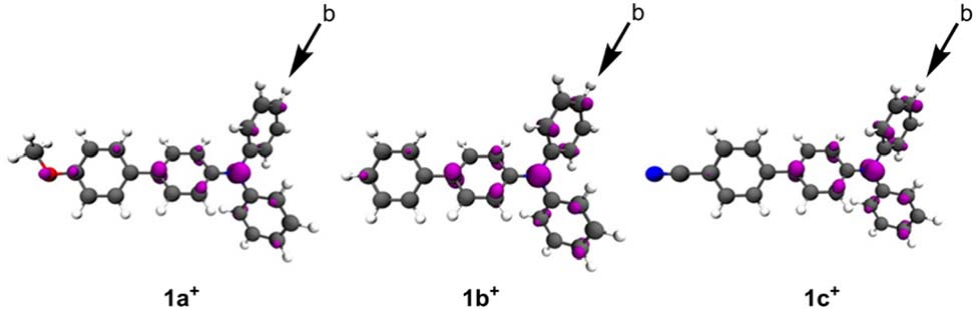
Spin density distribution of the radical cations 1a^+^ (R = OMe), 1b^+^ (R = H) and 1c^+^ (R = CN) (uPBE1PBE/6-31+G**, isosurface value at 0.008 a.u.) and probable dimerization position labeled with position b.

The spin density distribution visualizes the delocalization of the unpaired electron spin and is first discussed for the *p*-bTAA monomers 1 (Fig. 8). In the case of cation 1a^+^, the electron-donating methoxy group evidently induces a delocalization of the spin density across the entire bTAA skeleton, which stabilizes the resulting radical cation and impedes dimerization. In cation 1b^+^, there is only minimal spin density on the phenyl ring of the biaryl unit, and in cation 1c^+^, there is no spin density due to the electron-withdrawing cyano group. Consequently, the spin density is higher on the two phenyl rings directly attached to the nitrogen atom in cations 1b^+^ and 1c^+^ than in cation 1a^+^, which rationalizes different dimerization efficiencies. In the *para*-position of the phenyl rings to the nitrogen, the spin density is also slightly higher compared to the *ortho*-position, whereas no spin density is discernible in *meta*-position. The *para*-position labeled with position b is therefore considered the most reactive, analogous to the dimerization of triphenylamine.^26^ However, a dimerization process at the *para*-position R^1^ did not occur if R^2^ was blocked by a methoxy group.^18^

**Fig. 9:**
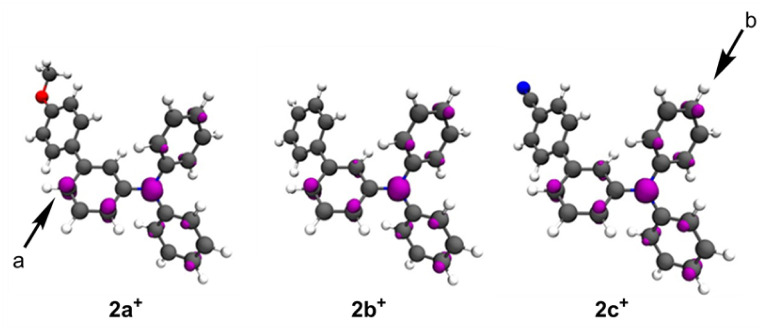
Spin density distribution of the radical cations 2a^+^ (R = OMe), 2b^+^ (R = H) and 2c^+^ (R = CN) (uPBE1PBE/6-31+G**, isosurface value at 0.011 a.u.) and probable dimerization position labeled with position a and b.

The spin density distribution is also investigated for the *m*-bTAA monomers 2 (Fig. 9). The quantum chemically calculated spin density distribution (uPBE1PBE/6-31+G**) of the methoxy substituted cation 2a^+^ reveals that the spin density, and hence the localization probability of the unpaired electron, is maximized at the sterically hindered position a (Fig. 9, left). This finding may account for the preferential formation of dimer 5a within the product mixture (Scheme 4). For the unsubstituted monomer radical cation 2b^+^ the spin density is also computational predicted highest at position a, but remains experimentally unverified (Fig. 9, center). However, for cation 2c^+^ the spin density at position a only marginally exceeds that at position b (Fig. 9, right). With nearly equal localization distribution of the unpaired electron spin at positions a and b, it is therefore reasonable to assume that dimerization preferentially occurs at position b, due to least steric hindrance.

**Fig. 10:**
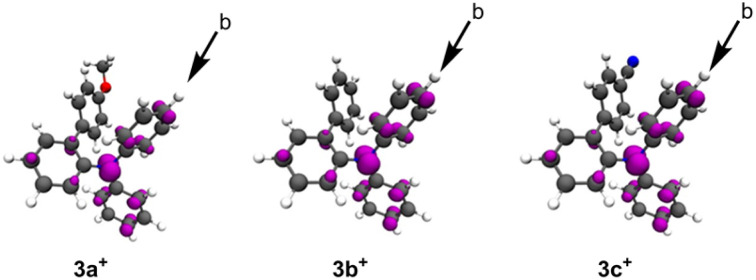
Spin density distribution of the radical cations 3a^+^ (R = OMe), 3b^+^ (R = H) and 3c^+^ (R = CN) (uPBE1PBE/6-31+G**, isosurface value at 0.008 a.u.) and probable dimerization position labeled with position b.

Quantum chemical spin density distributions of the *o*-bTAA monomer cations 3a^+^, 3b^+^, and 3c^+^ (uPBE1PBE/6-31+G**) reveal a similarly delocalized spin density, indicating that dimerization is independent of the substituent at R (Fig. 10). Coupling at position b is presumed to yield the major product, as this position exhibits reduced steric hindrance and highest spin density in all *o*-bTAA cations 3a^+^–c^+^. Dimerization at position a cannot be observed in any *o*-(bTAA)_2_6 and 7 (Schemes 7 and 8).

#### Calculated electronic structure

**Fig. 11:**
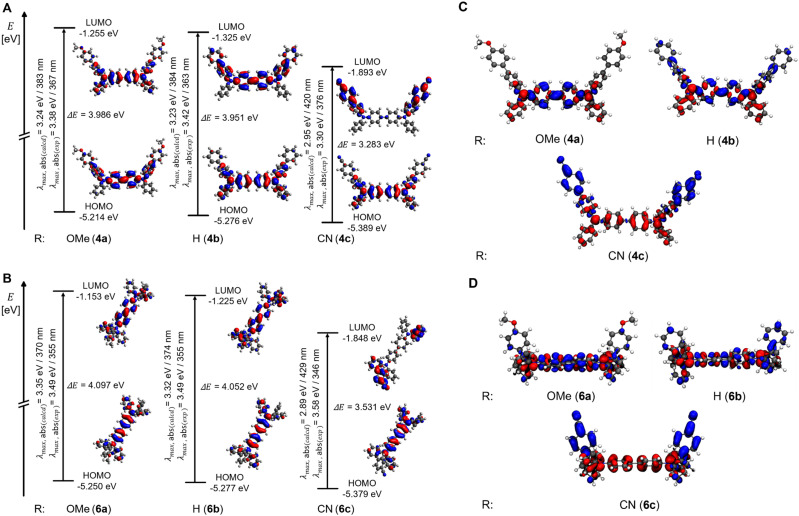
Kohn–Sham FMO of (A) *p*-(bTAA)_2_4a–c and (B) *o*-(bTAA)_2_6a–c (Gaussian 16, PBE1PBE/6-31+G**, PCM CH_2_Cl_2_, isosurface value at 0.025 a.u.) and charge density difference plots upon photoexcitation (longest wavelength absorption) of (C) *p*-(bTAA)_2_4a–c and (D) *o*-(bTAA)_2_6a–c (Gaussian 16, PBE1PBE/6-31+G**, PCM CH_2_Cl_2_, isosurface value at 0.001 a.u., blue indicates electron accumulation and red electron depletion).

For deeper insights into the electronic structure of dimers and rationalizing with the determined experimental data, TDDFT calculations were performed using Gaussian 16.^45^ Analogous to the monomers, the PBE1PBE functional^46,47^ and the Pople basis set 6-31+G**^48^ were employed.^17,18^ All minimum structures were confirmed by analytical frequency analysis (NImag = 0). Consistent with the spectroscopic properties, the calculations were carried out using the polarizable continuum model (PCM) with dichloromethane as the dielectric medium.^49^ The calculated excitation energies exhibit a slight bathochromic shift across dimers with electron donating or neutral substituents (deviations of 0.14–0.19 eV for 4a–b and 6a–b) and are in satisfactory agreement with the experimental data (Fig. 11A and B). Larger deviations are observed for the CN- and cyanopyridine substituted dimers 4–6c (0.35–0.69 eV), 6d (0.64 eV) and 7a–c (0.54–0.74 eV) which display an increased red-shift for the calculated values, with deviations of up to 0.74 eV (Fig. 11B, see SI, Tables S9–S13). The PBE1PBE functional^46,47^ is therefore unsuitable to correctly describe acceptor-substituted dimers, indicating limitations of the employed functional. Therefore, the use of long-range-corrected functionals (cam-B3LYP^50^ and ωB97X-D^51,52^) are investigated (exemplified for compound 6d and 7a) significantly reduces the deviation between calculated and experimental excitation energies (6d (0.33–0.37 eV), 7a (0.13 eV)) indicating that possible charge-transfer contributions are not fully captured at the PBE1PBE level. While these functionals underestimate the experimental transition energies, they reproduce the observed spectral trends more accurately. The calculated emission energies with the PBE1PBE functional of the dimers (4: 447–521 nm, 5: 430–557 nm, 6a–c: 411–527 nm) display similar behavior and are bathochromically shifted compared to the experimental data (4: 414–531 nm, 5: 425–532 nm, 6a–c: 403–523 nm) (see SI, chpt. 7.1, Tables S9–S11). For compound 7a, featuring a strong acceptor unit, excited-state geometry optimization at the PBE1PBE level results in an emission energy that deviates by 1.07 eV from the experimental value. This significant deviation further suggests that the PBE1PBE functional is not suitable for an accurate description of the excited-state electronic structure of this compound. Since the excitation and emission energies of mixture 5b/5b′/5b″ are very similar, comparison with the experimental results does not allow any conclusions to be reached about the possible composition of the mixture (see SI, Table S10).

The calculated absorption maxima and spectra exemplified for dimers 4b, 5c, and 6b (see SI, chpt. 7.1, Fig. S119–S121) illustrate that the longest wavelength absorption maximum is dominated by the HOMO → LUMO transition. For *meta*-dimer 5c the HOMO → LUMO transition exhibits only a small oscillator strength *f* = 0.0057 and is less allowed than in the monomer with the same substitution pattern (*f* = 0.0215)^18^ which accounts for the absence of a long-wavelength shoulder.^18^ In contrast, for the *meta*-dimers 5b–5b″ and the *ortho*-dimers 6a–c, the HOMO → LUMO transition is dominant with large *f* values (5b–5b″: *f* = 0.5579–0.12086, 6a–c: *f* = 0.2841–1.1404) and appears as a pronounced absorption maximum. For the *para*-dimers 4, as in monomers 1 (*f* = 0.7705–0.9207),^17^ the HOMO → LUMO transition is the most prominent, but with even higher *f* = 0.9938–1.3952 values (see SI, chpt. 7.1, Tables S11–S13). With decreasing donor strength at R, the HOMO energy decreases, and this effect is even more pronounced for the LUMO. Accordingly, Δ*E*(*E*_HOMO_ − *E*_LUMO_) is smallest for the CN-substituted dimers 4c, 5c, and 6c (Fig. 11A and B). The HOMO is predominantly localized on the bis(triarylamine) unit that connects the two monomeric subunits. The Δ*r* index (average hole–electron distance upon excitation)^53^ and the *S*_*r*_ index (overlap degree of hole and electron)^54^ providing measure of electron–hole separation to discuss the first excited state character. Compounds exhibiting large Δ*r* values and low *S*_*r*_ index indicate pronounced charge transfer (CT) character, although no strict universal thresholds exist.^53,54^ Photoexcitation induces a weak CT to the biaryl fragment for the dimers with electron-releasing or neutral substituents (*i.e.*4a–b and 6a–b (Δ*r* = 0.21–1.16 Å, *S*_*r*_ = 0.64–0.68)) resulting in an excited state with predominantly localized excitation (LE) character (see SI, Table S14). In the CN-substituted dimers (4c, 5c and 6c) this CT character is markedly stronger (Δ*r* = 0.91–3.72 Å, *S*_*r*_ = 0.31–0.53), such that the bis(triarylamine) unit retains little residual electron density (Fig. 11C and D). The resulting pronounced charge separation (*e.g.* for 4c and 6c) stabilizes the LUMO and thereby lowers it in energy relative to the electron-releasing or neutral substituted-dimers (Fig. 11A and B). This accounts for the strongly bathochromically shifted absorption and emission bands of the CN-substituted dimers 4c, 5c and 6c (Table 2) and the observed positive emission solvatochromism (Fig. 7). In the dimers, the HOMO level is elevated relative to the monomers,^17,18^ while the LUMO level is lowered, thereby diminishing Δ*E*(*E*_HOMO_ − *E*_LUMO_) = 3.283–4.097 eV (Fig. 11A and B). This rationalizes the experimentally observed bathochromic shift of the longest-wavelength absorption and emission bands of the dimers compared to the monomers.^17,18^ For the calculated emission spectra HOMO → LUMO transitions are dominant for all calculated dimers (4a–c, 5b–5b″, 5c and 6a–c). Indicative for an LE based emission, dimers with electron-releasing or neutral substituents (*i.e.*4a–b and 6a–b) exhibit low Δ*r* values and high *S*_*r*_ values (Δ*r* = 0.03–0.31 Å, *S*_*r*_ = 0.70–0.73) (see SI, Table S14). The calculated transitions are allowed due to high *f* values (0.8420–1.5707). The CN-substituted dimers 4c, 5c and 6c exhibiting comparatively low *f* values of 0.0103–0.5967 (see SI, Tables S9–S11). The calculated emission for these dimers displays large CT length of up to 8.17 Å indicative of a pronounced CT character (see SI, chpt. 7.1, Table S14).

## Conclusions

Various *m*-, *o*-, and *p*-bTAA, readily accessible by modular one-pot syntheses, were successfully dimerized to the corresponding tetraarylbenzidines by employing an oxidative homocoupling with methanesulfonic acid and chloranil. While the *meta*-derivatives give mixtures of products, the *ortho*- and *para*-derivatives furnish selective homocoupling at the least substituted *para*-position to the central nitrogen atom. The dimers display two reversible redox processes. For the *meta*-dimers, the cyclic voltammograms exhibit markedly less agreement with the dimerization products of the corresponding monomers, indicating a deviating dimer composition. By contrast, the redox signatures of the *ortho* dimers closely match with those of their monomeric precursors, consistent with possible identical structures. Relative to their monomers, the dimers show red-shifted absorption and emission maxima.^17,18^ The longest wavelength absorption band appears as a distinct maximum for *meta*- and *ortho*-dimers, since according to quantum chemical calculations, the HOMO → LUMO transition is no longer only weakly allowed. These findings underline that biaryl-substituted dimers ((bTAA)_2_) not only can be considered as typical hole-transport materials in organic electronic devices but also as fine-tunable emitters by suitable decoration with electron-withdrawing or releasing substituents. Further studies, addressing modular syntheses of the underlying monomers as chromophores with fine-tunable excited state properties are currently underway.

## Author contributions

Regina Kohlbecher: conceptualization, investigation (synthesis, electrochemical and photophysical characterization, (TD-)DFT calculations), data curation, formal analysis, and writing – original draft. Monika Flörke: conceptualization, investigation (synthesis, electrochemical and photophysical characterization, (TD-)DFT calculations), data curation, formal analysis, and writing – original draft. David Kempe: investigation (synthesis, electrochemical and photophysical characterization), and writing – review. Thomas J. J. Müller: conceptualization, resources, funding acquisition, project administration, supervision, and writing – review and editing. All authors have read and agreed to the published version of the manuscript.

## Conflicts of interest

There are no conflicts to declare.

## Supplementary Material

RA-OLF-D6RA04438G-s001

## Data Availability

The data supporting this article have been included as part of the supplementary information (SI). Ref. 17, 18, 24, 25, 45, 22, 31, 36, 33 and 38–43 from the manuscript are cited in the SI as ref. 1–4, 6–8, 12 and 14, 15. Ref. 55–59 are exclusively cited in SI as ref. 5, 9–11 and 13. Supplementary information: details on the synthesis and characterization (analytics) of compounds 4–7 and 10, NMR spectra of compounds 4–10, cyclic voltammograms and photophysical data of compounds 4–7 and data of the quantum chemical calculations on the structures of selected radical cations 1–3, and on the structures and photophysical data of compounds 4–6. See DOI: https://doi.org/10.1039/d6ra04438g.
