# Supplementary material for: Electronically tunable luminescent bis(biaryl-substituted triarylamine) ((bTAA)2) electrophores prepared via oxidative dimerization of modularly accessible arylated monomers
Source: RSC Adv. 2026 Jul 6;16(36):37734–45. doi: 10.1039/d6ra04438g (PMC13334536; doi:10.1039/d6ra04438g)
Supplement: Supplementary file 1 [file RA-016-D6RA04438G-s001.pdf]

Supplementary Information (SI) for RSC Advances.
This journal is © The Royal Society of Chemistry 2026

Supporting Information

Electronically tunable luminescent bis(biaryl-substituted triarylamine) ((bTAA)$_2$) electrophores prepared via oxidative dimerization of modularly accessible arylated monomers

Regina Kohlbecher,[a] Monika Flörke,[a] David Kempe,[a] and Thomas J. J. Müller*[a]

[a] Heinrich Heine University Düsseldorf, Faculty of Mathematics and Natural Sciences, Institute of Organic Chemistry and Macromolecular Chemistry, Universitätstrasse 1, Düsseldorf D-40225, Germany, E-mail: ThomasJJ.Mueller@hhu.de.

Supporting Information

Table of contents

1 General considerations 5

1.1 Experimental conditions of the synthesis 5

1.2 Analytics for structural elucidation 5

1.3 (Opto)electronic analytics 6

1.4 Quantum chemical calculations 7

2 Synthesis of symmetrical *N*,*N*-*ortho*-biaryl-substituted triaryl-amine-dimers (*sym*-*o*-bTAA-dimers) **10** 8

2.1 General procedure (**GP1**) for the synthesis of *N*,*N*-*ortho*-biaryl-substituted triarylamine-dimers (*sym*-*o*-bTAA-dimers) **10** 8

2.2 Spectroscopic data of *N*,*N*-*ortho*-biaryl-substituted triarylamine-dimers (*sym*-*o*-bTAA-dimers) **10** 9

3 Syntheses of *para*-/*meta*-/*ortho*-triarylamine dimers (*sym*-*p*/*m*/*o*-bTAA)$_2$ **4**, **5**, **6** and **7** 11

3.1 General procedure (**GP2**) for the synthesis of the *para*-/*meta*-/*ortho*-triarylamine dimers (*sym*-*p*-/*m*-/*o*-bTAA)$_2$ **4**, **5**, **6** and **7** 11

3.2 Spectroscopic data of *para*-/*meta*-/*ortho*-triarylamine dimers (*sym*-*p*-/*m*-/*o*-bTAA)$_2$ **4**, **5**, **6** and **7** 15

3.3 Synthesis of the *ortho*-triarylamine dimer (*sym*-*o*-bTAA)$_2$ **7c** 27

3.4 Synthesis of the *ortho*-triarylamine dimer (*sym*-*o*-bTAA)$_2$ **7a** via BLEBS Sequence for structural proof 28

4 ^{1}H and ^{13}C spectra of *meta*/*ortho*/*para*-triarylamine dimers (*sym*-*m*/*o*/*p*-bTAA)$_2$ **4**, **5**, **6**, and **7** 29

4.1 ^{1}H and ^{13}C spectra of *meta*/*ortho*/*para*-triarylamine dimers (*sym*-*m*/*o*/*p*-bTAA)$_2$ **4**, **5**, **6**, and **7** 29

4.2 High-Performance Liquid Chromatography (HPLC) spectra of *meta*/*ortho*/*para*-triarylamine dimers (*sym*-*m*/*o*/*p*-bTAA)$_2$ **4**, **5c**, **6**, and **7** 48

5 Cyclic voltammetry 53

5.1 Dimerization process exemplified by a *sym*-*o*-bTAA[2] 53

5.2 Cyclic voltammograms of dimers and monomers with dimerization processes in comparison 54

5.2.1 Comparison of *sym*-*m*-bTAA 2 and *sym*-*m*-(bTAA)$_2$ **5** 54

5.2.2 Comparison of *sym*-*o*-bTAA 3 and *sym*-*o*-(bTAA)$_2$ **6** 56

5.3 Cyclic voltammogram of *para*-/*meta*-/*ortho*-triarylamine dimers (*sym*-*p*-/*m*-/*o*-bTAA)$_2$ **4**, **5**, **6** and **7** 59

5.4 Electrochemical reversibility 67

5.5 Electrochemical process of compound **5d** 75

6 Photophysical data 80

6.1 Absorption and emission spectra of *para*-/*meta*-/*ortho*-triarylamine dimers (*sym*-*p*-/*m*-/*o*-bTAA)$_2$ **4**, **5**, **6** and **7** 80

6.2 Solid state emission spectra of *para-/meta-/ortho*-triarylamine dimers (*sym-p-/m-/o*-bTAA)$_2$ **4**, **5**, **6** and **7** 90
6.2.1 Solid state emission spectra of **6d** and **6e** in comparison to their spectra in solution 98
6.3 PMMA matrix emission spectra of *ortho*-triarylamine dimers (*sym-o*-bTAA)$_2$ **6d**, **6e** and **7** 100
7 Data of quantum chemical calculations 102
7.1 Overview of quantum chemical calculations of dimers **4**, **5** and **6** 102
7.2 Computed xyz-coordinates of cation **1a$^+$** (uPBE1PBE/6-31+G**) 111
7.3 Computed xyz-coordinates of cation **1b$^+$** (uPBE1PBE/6-31+G**) 114
7.4 Computed xyz-coordinates of cation **1c$^+$** (uPBE1PBE/6-31+G**) 116
7.5 Computed xyz-coordinates of cation **2a$^+$** (uPBE1PBE/6-31+G**) 119
7.6 Computed xyz-coordinates of cation **2b$^+$** (uPBE1PBE/6-31+G**) 122
7.7 Computed xyz-coordinates of cation **2c$^+$** (uPBE1PBE/6-31+G**) 125
7.8 Computed xyz-coordinates of cation **3a$^+$** (uPBE1PBE/6-31+G**) 128
7.9 Computed xyz-coordinates of cation **3b$^+$** (uPBE1PBE/6-31+G**) 131
7.10 Computed xyz-coordinates of cation **3c$^+$** (uPBE1PBE/6-31+G**) 134
7.11 Quantum chemical calculation data of *N*^{4},*N*$^{4'}$-Bis(4'-methoxy-[1,1'-biphenyl]-4-yl)-*N*^{4},*N*$^{4'}$-diphenyl-[1,1'-biphenyl]-4,4'-diamine (**4a**) 137
7.11.1 Computed xyz-coordinates of compound **4a** (PBE1PBE/6-31+G** PCM CH_2Cl_2) 137
7.11.2 Computed excitations of compound **4a** (PBE1PBE/6-31+G** PCM CH_2Cl_2) 140
7.11.3 Computed emission of compound **4a** (PBE1PBE/6-31+G** PCM CH_2Cl_2) 144
7.12 Quantum chemical calculation data of *N*^{4},*N*$^{4'}$-Di([1,1'-biphenyl]-4-yl)-*N*^{4},*N*$^{4'}$-diphenyl-[1,1'-biphenyl]-4,4'-diamine (**4b**) 147
7.12.1 Computed xyz-coordinates of compound **4b** (PBE1PBE/6-31+G** PCM CH_2Cl_2) 147
7.12.2 Computed excitations of compound **4b** (PBE1PBE/6-31+G** PCM CH_2Cl_2) 150
7.12.3 Computed emission of compound **4b** (PBE1PBE/6-31+G** PCM CH_2Cl_2) 153
7.13 Quantum chemical calculation data of 4',4'''-([1,1'-Biphenyl]-4,4'-diylbis(phenylazanediyl))bis(([1,1'-biphenyl]-4-carbonitrile)) (**4c**) 156
7.13.1 Computed xyz-coordinates of compound **4c** (PBE1PBE/6-31+G** PCM CH_2Cl_2) 156
7.13.2 Computed excitations of compound **4c** (PBE1PBE/6-31+G** PCM CH_2Cl_2) 159
7.13.3 Computed emission of compound **4c** (PBE1PBE/6-31+G** PCM CH_2Cl_2) 162
7.14 Quantum chemical calculation data of *N*$^{4''}$,*N*$^{4''}$,*N*$^{5'}$,*N*$^{5'}$-tetraphenyl-[1,1':2',1'':2'',1'''-quaterphenyl]-4'',5'-diamine (**5b**) 165
7.14.1 Computed xyz-coordinates of compound **5b** (PBE1PBE/6-31+G** PCM CH_2Cl_2) 165
7.14.2 Computed excitations of compound **5b** (PBE1PBE/6-31+G** PCM CH_2Cl_2) 168
7.14.3 Computed emission of compound **5b** (PBE1PBE/6-31+G** PCM CH_2Cl_2) 171

7.15 Quantum chemical calculation data of *N*[4]-([1,1'-biphenyl]-3-yl)-*N*[4],*N*[4']*,N*[4']-triphenyl-[1,1':2',1''-terphenyl]-4,4'-diamine (**5b'**) 174

7.15.1 Computed xyz-coordinates of compound **5b'** (PBE1PBE/6-31+G** PCM CH_2Cl_2) 174

7.15.2 Computed excitations of compound **5b'** (PBE1PBE/6-31+G** PCM CH_2Cl_2) 177

7.15.3 Computed emission of compound **5b'** (PBE1PBE/6-31+G** PCM CH_2Cl_2) 180

7.16 Quantum chemical calculation data of *N*[4],*N*[4']-Di([1,1'-biphenyl]-3-yl)-*N*[4],*N*[4']-diphenyl-[1,1'-biphenyl]-4,4'-diamine (**5b''**) 182

7.16.1 Computed xyz-coordinates of compound **5b''** (PBE1PBE/6-31+G** PCM CH_2Cl_2) 182

7.16.2 Computed excitations of compound **5b''** (PBE1PBE/6-31+G** PCM CH_2Cl_2) ...185

7.16.3 Computed emission of compound **5b''** (PBE1PBE/6-31+G** PCM CH_2Cl_2) 188

7.17 Quantum chemical calculation data of 3',3'''-([1,1'-Biphenyl]-4,4'-diylbis(phenylazanediyl))bis(([1,1'-biphenyl]-4-carbonitrile)) (**5c**) 190

7.17.1 Computed xyz-coordinates of compound **5c** (PBE1PBE/6-31+G** PCM CH_2Cl_2) 190

7.17.2 Computed excitations of compound **5c** (PBE1PBE/6-31+G** PCM CH_2Cl_2) 193

7.17.3 Computed emission of compound **5c** (PBE1PBE/6-31+G** PCM CH_2Cl_2) 196

7.18 Quantum chemical calculation data of *N*[4],*N*[4']-Bis(4'-methoxy-[1,1'-biphenyl]-2-yl)-*N*[4],*N*[4']-diphenyl-[1,1'-biphenyl]-4,4'-diamine (**6a**) 199

7.18.1 Computed xyz-coordinates of compound **6a** (PBE1PBE/6-31+G** PCM CH_2Cl_2) 199

7.18.2 Computed excitations of compound **6a** (PBE1PBE/6-31+G** PCM CH_2Cl_2) 202

7.18.3 Computed emission of compound **6a** (PBE1PBE/6-31+G** PCM CH_2Cl_2) 206

7.19 Quantum chemical calculation data of *N*[4],*N*[4']-Di([1,1'-biphenyl]-2-yl)-*N*[4],*N*[4']-diphenyl-[1,1'-biphenyl]-4,4'-diamine (**6b**) 209

7.19.1 Computed xyz-coordinates of compound **6b** (PBE1PBE/6-31+G** PCM CH_2Cl_2) 209

7.19.2 Computed excitations of compound **6b** (PBE1PBE/6-31+G** PCM CH_2Cl_2) 212

7.19.3 Computed emission of compound **6b** (PBE1PBE/6-31+G** PCM CH_2Cl_2) 215

7.20 Quantum chemical calculation data of 2',2'''-([1,1'-Biphenyl]-4,4'-diylbis(phenylazanediyl))bis(([1,1'-biphenyl]-4-carbonitrile)) (**6c**) 218

7.20.1 Computed xyz-coordinates of compound **6c** (PBE1PBE/6-31+G** PCM CH_2Cl_2) 218

7.20.2 Computed excitations of compound **6c** (PBE1PBE/6-31+G** PCM CH_2Cl_2) 221

7.20.3 Computed emission of compound **6c** (PBE1PBE/6-31+G** PCM CH_2Cl_2) 224

7.21 Quantum chemical calculation data of 2',2'''-([1,1'-Biphenyl]-4,4'-diylbis(phenylazanediyl))bis(([1,1'-biphenyl]-2,5-dicarbonitrile)) (**6d**)....................227

7.21.1 Computed xyz-coordinates of compound **6d** (PBE1PBE/6-31+G** PCM CH_2Cl_2) 227

7.21.1.1 Computed excitations of compound **6d** (PBE1PBE/6-31+G** PCM CH_2Cl_2) ..230

7.21.2 Computed xyz-coordinates of compound **6d** (ωB97XD/6-31+G** PCM CH_2Cl_2)233

7.21.2.1 Computed excitations of compound **6d** (ωB97XD /6-31+G** PCM CH_2Cl_2)....236
7.21.3 Computed xyz-coordinates of compound **6d** (CAM-B3LYP/6-31+G** PCM CH_2Cl_2)....241
7.21.3.1 Computed excitation of compound **6d** (CAM-B3LYP/6-31+G** PCM CH_2Cl_2) 244
7.22 Quantum chemical calculation data of 6,6'-(([1,1'-biphenyl]-4,4'-diylbis(phenylazanediyl))bis(2,1-phenylene))dipicolinonitrile (**7a**) 249
7.22.1 Computed xyz-coordinates of compound **7a** (PBE1PBE/6-31+G** PCM CH_2Cl_2) 249
7.22.1.1 Computed excitations of compound **7a** (PBE1PBE/6-31+G** PCM CH_2Cl_2) ..252
7.22.1.2 Computed emission of compound **7a** (PBE1PBE/6-31+G** PCM CH_2Cl_2) 255
7.22.2 Computed xyz-coordinates of compound **7a** (CAM-B3LYP/6-31+G** PCM CH_2Cl_2)....257
7.22.2.1 Computed excitation of compound **7a** (CAM-B3LYP/6-31+G** PCM CH_2Cl_2) 260
7.23 Quantum chemical calculation data of 6,6'-(([1,1'-biphenyl]-4,4'-diylbis(phenylazanediyl))bis([1,1'-biphenyl]-2',4-diyl))dipicolino-nitrile (**7b**) 264
7.23.1 Computed xyz-coordinates of compound **7b** (PBE1PBE/6-31+G** PCM CH_2Cl_2) 264
7.23.2 Computed excitation of compound **7b** (PBE1PBE/6-31+G** PCM CH_2Cl_2).......268
7.24 Quantum chemical calculation data of 6,6'-(((2,2'-dimethyl-[1,1'-biphenyl]-4,4'-diyl)bis(phenylazanediyl))bis(2,1-phenylene))dipicolinonitrile (**7c**) 272
7.24.1 Computed xyz-coordinates of compound **7c** (PBE1PBE/6-31+G** PCM CH_2Cl_2) 272
7.24.2 Computed excitation of compound **7c** (PBE1PBE/6-31+G** PCM CH_2Cl_2).......275
8 Solvatochromism....278
8.1 Quantum chemical calculation data of 2',2'''-([1,1'-Biphenyl]-4,4'-diylbis(phenylazanediyl))bis(([1,1'-biphenyl]-2,5-dicarbonitrile)) (**6d**)....279
8.1.1 Computed xyz-coordinates of compound **6d** (PBE1PBE/6-31+G**) in the gas phase 279
8.1.2 Computed molecule volume of compound **6d** (PBE1PBE/6-31+G**, volume) in the gas phase....282
8.1.3 Computed molecule volume of compound **6d** (PBE1PBE/6-31+G**, volume) in cyclohexane 283
8.1.4 Computed molecule volume of compound **6d** (PBE1PBE/6-31+G**, volume) in toluene 283
8.1.5 Computed molecule volume of compound **6d** (PBE1PBE/6-31+G**, volume) in 1,4-dioxane....284
8.1.6 Computed molecule volume of compound **6d** (PBE1PBE/6-31+G**, volume) ethyl acetate 284
8.1.7 Computed molecule volume of compound **6d** (PBE1PBE/6-31+G**, volume) dichloromethane....285
9 References....286

1 General considerations

1.1 Experimental conditions of the synthesis

All reactions were carried out in oven dried Schlenk glassware using septa and syringes under nitrogen atmosphere. The biaryl-substituted triarylamine monomers (bTAA) are known compounds (*para*-bTAA **1a–c**[1,2], *meta*-bTAA **2a–d**[1,2], *ortho*-bTAA **3a–c**[1,2]) and were synthesized according to the literature.[1,2] The *ortho*-bTAA monomers **3d–e**[3] and **3f–g**[4] were synthesized by BLEBS sequence starting from *ortho*-bromo-substituted triarylamines and final Suzuki coupling with halogenated terephthalodinitriles or cyanopyridines. Details of their synthesis will be reported in separate publications.[3,4] The other reagents and catalyst were purchased reagent-grade and used without purification. Dry solvents were dried by a solvent purification system.

The reaction progress was monitored qualitatively using TLC Silica gel 60 F234 aluminum sheets obtained from MACHEREY-NAGEL GmbH & Co. KG. The spots were detected with UV light at 254 and 366 nm. The purification of the products was performed on silica gel 60 M (0.04-0.063 mm) from MACHEREYNAGEL GmbH & Co. KG using flash technique under pressure of 2 bar. The crude mixtures were absorbed on Celite® 545 form Carl Roth GmbH & Co. KG before chromatographic purification. Ethyl acetate and mixtures of *n*-hexane/ethyl acetate were used as eluent.

1.2 Analytics for structural elucidation

^{1}H, ^{19}F, ^{13}C and 135-DEPT ^{13}C NMR spectra were recorded on Bruker AVIII-300 and AVIII-600. Acetone-d_6, DMSO-d_6, dichloromethane-d_2, benzene-d_6 and chloroform-d_1 were used as deuterated solvents. The resonances of the solvents were locked as internal standard (acetone-d_6: ^{1}H δ 2.05 (water in acetone-d_6 ^{1}H δ 2.84)*, ^{13}C δ 29.84, 206.26; DMSO-d_6: ^{1}H δ 2.50 (water in DMSO-d_6 ^{1}H δ 3.33)*, ^{13}C δ 39.52; dichloromethane-d_2: ^{1}H δ 5.32, ^{13}C δ 53.80; benzene-d_6: ^{1}H δ 7.16 (water in benzene-d_6 ^{1}H δ 0.40)*, ^{13}C δ 128.06; chloroform-d_1: ^{1}H δ 7.26, (water in chloroform-d_1 ^{1}H δ 1.56)*, ^{13}C δ 77.00). The multiplicities of the signals were abbreviated as follows: s: singlet; d: doublet; t: triplet; q: quatet; dd: doublet of doublet; dt: doublet of triplet; tt: triplet of triplets; ddd: doublet of doublet of doublets; m: multiplet. The type of carbon nucleus was determined based on 135-DEPT ^{13}C NMR spectra. For the description of the ^{13}C NMR spectra primary carbon nuclei are abbreviated with CH_3, secondary carbon nuclei with CH_2, tertiary carbon nuclei with CH and quaternary carbon nuclei with C_{quat}. The program Mestrenova 11.0 was used to evaluate the NMR spectra.

* Due to the water content in acetone-d_6, DMSO-d_6, benzene-d_6 and chloroform-d_1 the water peaks in the ^{1}H NMR spectra are also labeled with "acetone-d_6", "DMSO-d_6" "C_6D_6"and "$CDCl_3$".

EI mass spectra were measured on Finnigan MAT TSQ 7000. High-resolution ESI mass spectra and HPLC chromatograms were recorded on the UHR QTOF maXis 4G device manufactured by Bruker.
IR spectra were obtained on Shimadzu IR Affinity-1 which works with the attenuated total reflection (ATR) method. The intensity of signals is abbreviated as follows: s (strong), m (medium), w (weak).
The melting points (uncorrected) were measured on Büchi Melting Point B-540.
Combustion analyses were carried out on Perkin Elmer Series II Analyser 2400 in the micro analytical laboratory of the Institute for Pharmaceutical and Medicinal Chemistry at Heinrich Heine University Düsseldorf.

1.3 (Opto)electronic analytics

Absorption spectra were recorded in cyclohexane, toluene, 1,4-dioxane, ethyl acetate and dichloromethane high performance liquid chromatography (HPLC) grade at 293 K on a Perkin–Elmer UV/Vis/NIR Lambda 19 spectrometer. For the determination of the molar extinction coefficients ε absorption measurements at five different concentrations were carried out. Emission and excitation spectra were recorded in cyclohexane, toluene, 1,4-dioxane, ethyl acetate and dichloromethane HPLC grade as well as in the solid state and embedded in a polymethyl methacrylate (PMMA) matrix at 293 K on a Hitachi F-7000 or FS5 spectrometer. A xenon vapor lamp was employed for steady-state emission measurements. Absolute photoluminescence quantum yields (in solution, in the solid-state and embedded in a PMMA matrix) were measured using the integrating sphere module (SC30) of the FS5 spectrofluorometer as well as software with instrument-specific reference correction applied throughout the measurement procedure by Edinburgh Instruments. The absorption of each solution was adjusted to approximately 0.1 at the longest wavelength absorption maximum. Samples were excited at this absorption maximum, and the quantum yields were determined from direct integrating sphere measurements. For PMMA films and solid-state powder samples, the excitation wavelength was selected from the longest wavelength maximum of the corresponding excitation spectrum. Quantum yields of the PMMA films were determined using direct integrating sphere measurements. For powder samples, both direct and indirect integrating sphere measurements were performed.
For film preparation, 1 wt% of the compound (0.2 mg) and polymethyl methacrylate (PMMA) (20 mg) were dissolved in dichloromethane (2 ml), cast into molds, and then cured at 298 K overnight.
Cyclic voltammetry experiments were performed with 263A E&G Princeton Applied Research as potentiostatic instrumentation under argon in dry and degassed dichloromethane at 293 K and at scan rates of 100, 250, 500 and 1000 mVs^{-1}. The working electrode was a 1 mm

platinum disk, the counter electrode was a platinum wire and the reference electrode was a silver/silver chloride electrode filled with saturated sodium chloride solution (c = 3.0 M). The electrolyte was tetrabutylammonium hexafluorophosphate at a concentration of c = 0.1 M. The potential was calibrated using $[FeCp^*_2]/[FeCp^*_2]^+$ as an internal potential standard. The absolute potential of this standard was determined against $[FeCp_2]/[FeCp_2]^+$ ($E_0^{0/+1}$ = 0.00 mV).[5] This procedure provided a value of $E_0^{0/+1}$ = −540 mV for $[FeCp^*_2]/[FeCp^*_2]^+$. Further details of the measurement setup and determination of the provided redox potentials are discussed in chapter 5.4.

1.4 Quantum chemical calculations

The quantum chemical calculations were performed using the Gaussian 16[6] software package on the HILBERT high-performance computing cluster at the Center for Information and Media Technology (ZIM) at Heinrich Heine University Düsseldorf. Density functional theory methods were used, and after each geometry optimization to determine minimum structures (NImag = 0) or transition states, a frequency analysis was performed before further calculations were carried out. All methods, functionals, and basis sets used are listed under the respective results.

2 Synthesis of symmetrical *N*,*N*-*ortho*-biaryl-substituted triarylamine-dimers (*sym*-*o*-bTAA-dimers) 10

2.1 General procedure (GP1) for the synthesis of *N*,*N*-*ortho*-biaryl-substituted triarylamine-dimers (*sym*-*o*-bTAA-dimers) 10

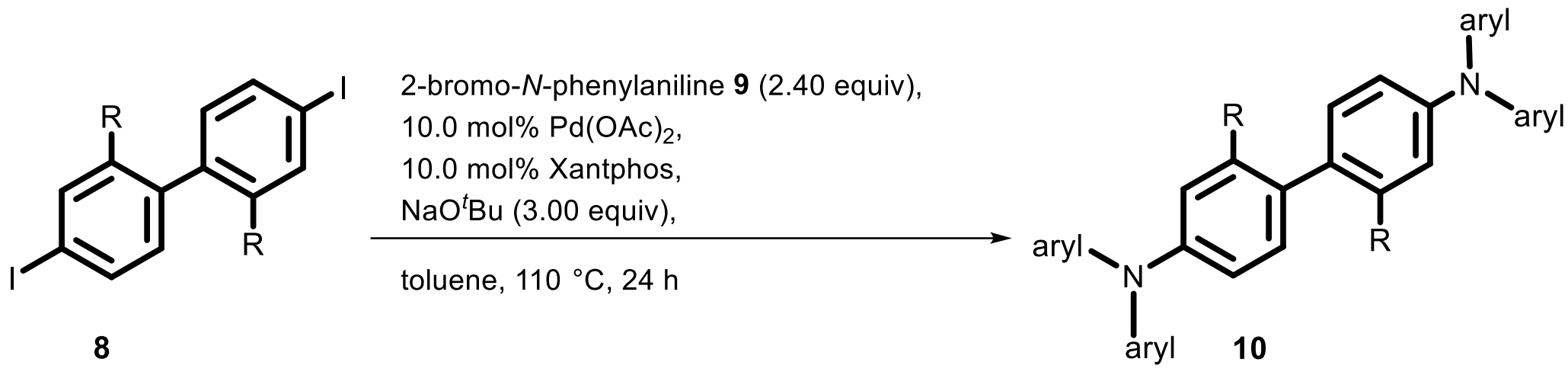

Under nitrogen in a Schlenk tube with magnetic stir bar aryldihalide **8** (1.00 equiv), 2-bromo-*N*-phenylaniline **9** (2.40 equiv), palladium(II)acetate (10.0 mol%), 4,5-bis(diphenylphosphano)-9,9-dimethylxanthene (Xantphos, 10.0 mol%) and sodium *tert*-butoxide (3.00 equiv) were dissolved in dry toluene (0.20 mmol/mL). Then, the reaction mixture was stirred at 110 °C (oil bath temperature) for 24 h. After cooling to room temperature, the reaction mixture was diluted in ethyl acetate (50.0 ml) and was purified by flash chromatography on silica gel and by recrystallization to give compound as a solid.

Table S1. Experimental details for the synthesis *sym*-*o*-bTAA-dimers **10**.

entry	Biphenyldihalide **8**	2-bromo-*N*-phenylaniline **9**	Yield of product **10**
1[a,b]	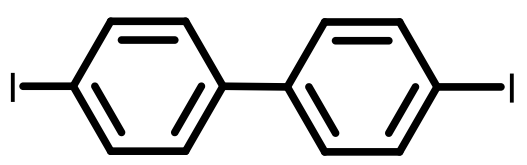 1.23 g (3.00 mmol) of **8a**	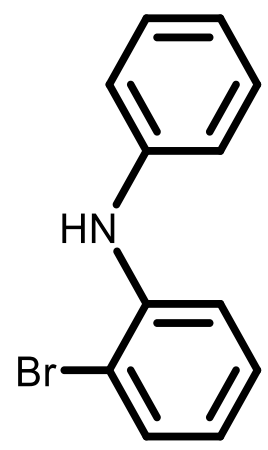 1.77 g (7.20 mmol)	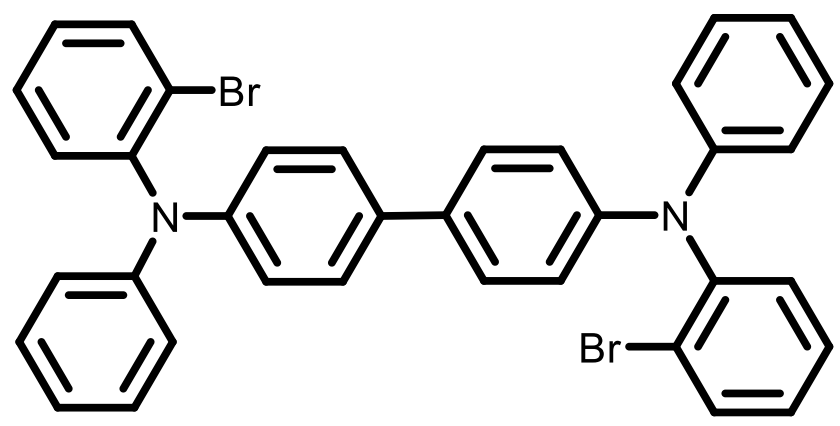 1.38 g (71%) of **10a**
2[a,c]	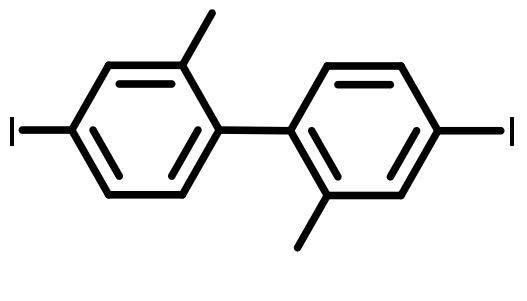 0.22 g (0.50 mmol) of **8**	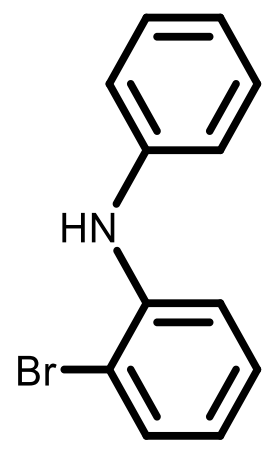 0.27 g (1.20 mmol)	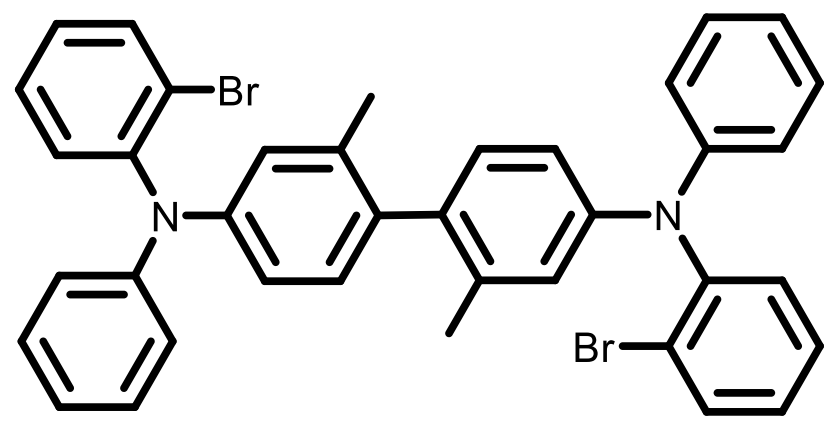 204 mg (60%) of **10**

[a] Yields after flash chromatography on silica gel.
[b] For the reaction: 67.4 mg (0.30 mmol) of $Pd(OAc)_2$, 174 mg (0.30 mmol) of Xantphos, 865 mg (9.00 mmol) of NaOtBu, 15.0 ml of toluene
[c] For the reaction: 11.3 mg (0.05 mmol) of $Pd(OAc)_2$, 28.9 mg (0.05 mmol) of Xantphos, 144 mg (1.50 mmol) of NaO^{t}Bu, 2.50 ml of toluene

2.2 Spectroscopic data of *N*,*N*-*ortho*-biaryl-substituted triarylamine-dimers (*sym*-*o*-bTAA-dimers) 10

2.2.1 *N*4,*N*4'-Bis(2-bromophenyl)-*N*4,*N*4'-diphenyl-[1,1'-biphenyl]-4,4'-diamine (10a)

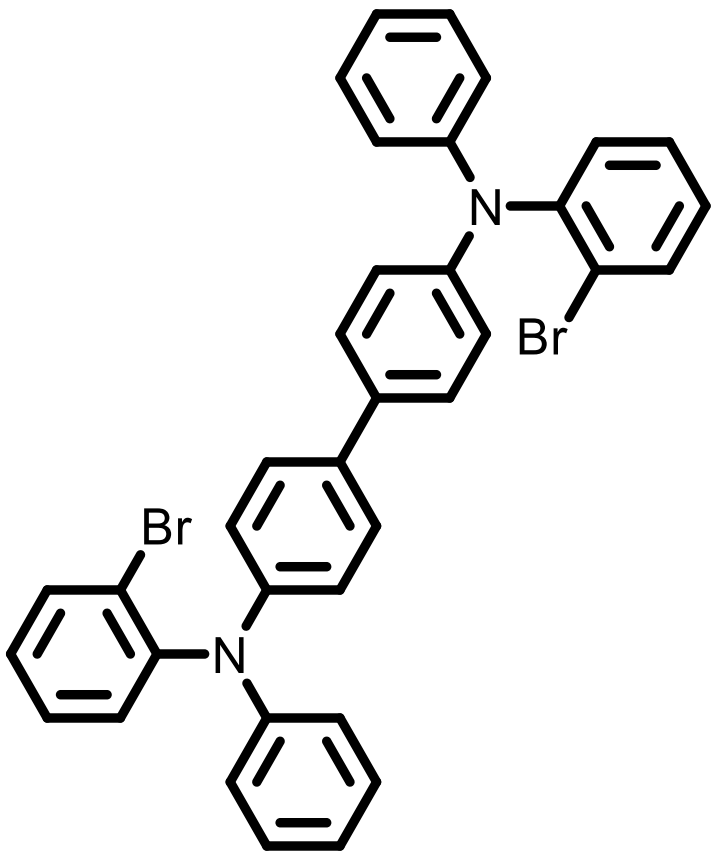

The synthesis was performed by GP1. After chromatography on silica gel (*n*-hexane/ethyl acetate 50:1) and recrystallization from *n*-hexane compound **10a** (1.38 g, 2.10 mmol, 71%) was isolated as colorless crystals, Mp 183 °C. (Lit.: 162-164 °C)[7]. R_f = 0.42 (*n*-hexane/ethyl acetate 50:1).

^{1}H NMR (300 MHz, chloroform-d$_1$): δ 6.94–7.08 (m, 10H), 7.14 (ddd, ^3J = 8.0 Hz, ^3J = 7.0 Hz, ^4J = 2.0 Hz, 2H), 7.22–7.39 (m, 8H), 7.42–7.51 (m, 4H), 7.68 (dd, ^3J = 8.0 Hz, ^4J = 1.4 Hz, 2H). ^{13}C NMR (75 MHz, chloroform-d$_1$) δ 122.2 (CH), 122.2 (CH), 123.9 (C$_{quat}$), 127.3 (CH), 127.5 (CH), 129.0 (CH), 129.2 (CH), 131.8 (CH), 134.4 (C$_{quat}$), 134.7 (CH), 145.5 (C$_{quat}$), 146.0 (C$_{quat}$), 147.0 (C$_{quat}$). EI MS (70 eV, m/z (%)) 649 (18), 648 (45), 647 (35), 646 ([^{81}Br-M]$^+$, 100), 645 (33), 644 ([^{79}Br-M]$^+$, 85), 567 (25), 566 (59), 565 (25), 564 (53), 486 (18), 485 (25), 484 (37), 408 (19), 407 (14), 406 (10), 324 (23), 323 ([$C_{18}H_{13}{}^{81}BrN$]$^+$, 36), 322 ([$C_{18}H_{13}{}^{79}BrN$]$^+$, 17), 320 (26), 319 (72), 318 (20), 317 (20), 316 (10), 283 (11), 244 (17), 243 (84), 242 (56), 241 (44), 240 (17), 204 (25), 203 (13), 167 ([$C_{12}H_9N$]$^{2+}$, 38), 166 (26), 152 (10), 77 ([C_6H_5]$^+$, 16).

2.2.2 *N*4,*N*4'-Bis(2-bromophenyl)-2,2'-dimethyl-*N*4,*N*4'-diphenyl-[1,1'-biphenyl]-4,4'-diamine (10)

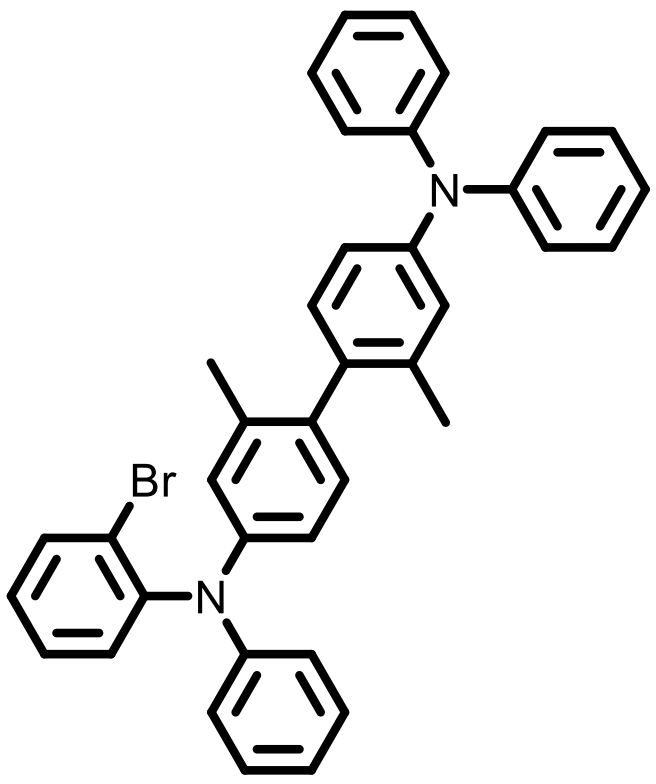

The synthesis was performed by GP1. After chromatography on silica gel (*n*-hexane/ethyl acetate 50:1) and recrystallization from *n*-hexane compound **10** (204 mg, 0.30 mmol, 60%) was isolated as colorless crystals. The product was obtained as an inseparable mixture with the side product *N*4-(2-bromophenyl)-2,2'-dimethyl-*N*4,*N*4',*N*4'-triphenyl-[1,1'-biphenyl]-4,4'-diamine and was used as such in the next step. Mp 93 °C. R_f 0.31 (*n*-hexane/ethyl acetate 50:1).

^{1}H NMR (600 MHz, chloroform-d$_1$) δ 2.02 (s, 6H), 6.86 (dd, ^3J = 8.2 Hz, ^4J = 2.4 Hz, 2H), 6.92 (d, ^4J = 2.4 Hz, 2H), 6.99 – 7.04 (m, 6H), 7.15 (dd, ^3J = 7.6 Hz, ^4J = 1.8 Hz, 2H), 7.30 – 7.25 (m, 6H), 7.33 (dd, ^3J = 7.9 Hz, ^4J = 1.9 Hz, 2H), 7.41 – 7.34 (m, 2H), 7.71 – 7.66 (m, 2H).^{13}C NMR (151 MHz, chloroform-d$_1$) δ 20.3 (CH_3), 119.5 (CH), 121.8 (CH), 121.9 (CH), 123.4 (CH), 123.9 (C_{quat}), 127.3 (CH), 129.0 (CH), 129.1 (CH), 130.0 (CH), 130.4 (CH), 131.8 (CH), 134.7 (CH), 135.6 (C_{quat}), 137.2 (C_{quat}), 145.8 (C_{quat}), 145.9 (C_{quat}), 147.2 (C_{quat}). EI MS (70 eV, 678 (20), 677 (54), 676 (Br^{81}-$[M]^+$-Br^{81},48), 675 ($[M]^+$,100), 674 (Br^{81}-$[M]^+$-Br^{79}, 35), 673 (51), 649 (13), 648 (11), 647 (24), 645 (12), 596* ($[C_{38}H_{31}{}^{81}BrN_2]^+$, 21), 595 ($[C_{38}H_{30}{}^{81}BrN_2]^+$, 49), 594* ($[C_{38}H_{31}{}^{79}BrN_2]^+$, 25), 593 ($[C_{38}H_{30}{}^{79}BrN_2]^+$, 44), 567 (14), 565 (11), *515 ($[C_{38}H_{31}N_2]^+$, 13), 514 ($[C_{38}H_{30}N_2]^{2+}$, 15), 513 (16), 499 (18), 485 (15), 348* ($[C_{26}H_{22}N]^+$, 10), 338 ($[C_{19}H_{15}{}^{81}BrN]^+$, 13), 337 (13), 332 (10), 331 (15), 330 (12), 297 (11), 258* ($[C_{19}H_{16}N]^+$, 18), 257 (53), 256 (18), 254 (14), 250 (37), 249 (28), 248 (13), 243* ($[C_{18}H_{13}N]^{2+}$, 30), 242 (24), 241 (20), 211 (16), 210 (29), 209 (18), 204 (15), 203 (10), 167 (55), 166 (42), 165 ($[C_{13}H_9]^{3+}$, 17), 152 (12), 140 (12), 139 (12), 115 (10), 77 ($[C_6H_5]^+$, 28). IR ($\tilde{\nu}$ [cm^{-1}]) 3057 (w), 2961 (w), 2922 (w), 2870 (w), 2853 (w), 1728 (w), 1593 (w), 1580 (w), 1485 (m), 1470 (m), 1454 (w), 1439 (w), 1412 (w), 1379 (w), 1306 (m), 1287 (w), 1260 (m), 1248 (w), 1207 (w), 1177 (w), 1157 (w), 1119 (w), 1076 (w), 1049 (w), 1028 (m), 1005 (w), 885 (w), 864 (w), 816 (w), 748 (m), 721 (m), 694 (m), 681 (w), 660 (w). HRMS (ESI) calcd. for $[C_{38}H_{30}Br_2N_2]^+$: 672.0770; Found 672.0771.

*Mass signals due to the side product *N*4-(2-bromophenyl)-2,2'-dimethyl-*N*4,*N*4',*N*4'-triphenyl-[1,1'-biphenyl]-4,4'-diamine

3 Syntheses of *para-/meta-/ortho*-triarylamine dimers (*sym-p/m/o*-bTAA)$_2$ 4, 5,6 and 7

3.1 General procedure (GP2) for the synthesis of the *para-/meta-/ortho*-triarylamine dimers (*sym-p-/m-/o*-bTAA)$_2$ 4, 5, 6 and 7

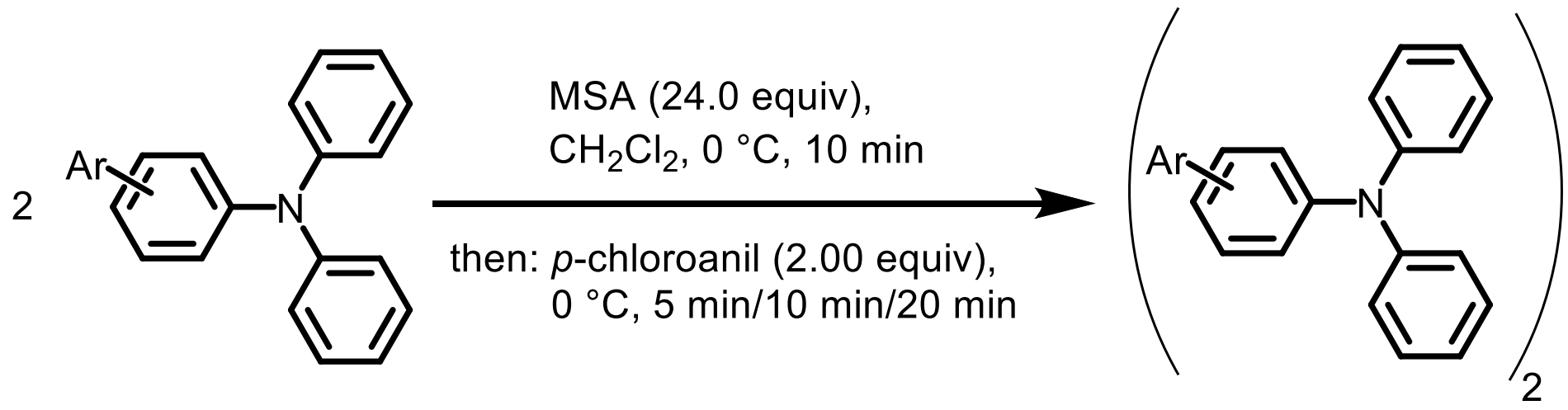

1,**2** or **3** → **4**, **5**, **6** or **7**

In a secured Schlenk tube with magnetic stirbar *sym-p*-bTAA **1**, *sym-m*-bTAA **2** or *sym-o*-bTAA **3** (0.50 mmol, 1.00 equiv) was added and dissolved in dry dichloromethane (6.30 mL). The reaction mixture was then cooled to 0 °C and methanesulfonic acid (1.15 g, 12.0 mmol, 24.0 equiv) was added. The reaction mixture was then stirred for 10 min at 0 °C. Next, *p*-chloroanil (246 mg, 1.00 mmol, 2.00 equiv) was added and stirred at 0 °C for 5 min (*sym-o*-bTAA **3**), 10 min (*sym-m*-bTAA **2**) or 20 min (*sym-p*-bTAA **1**). After warming to room temp, ethyl acetate and aqueous sodium hydrogen carbonate solution were added to the reaction mixture and the organic phase was separated off. The aqueous phase was then extracted three times with ethyl acetate. The organic phases were combined and, after drying with anhydrous magnesium sulfate, the solvent was removed under reduced pressure. The crude product **4**, **5**, **6** or **7** was purified by column chromatography on silica gel and then recrystallized to give the pure compounds.

Table S2. Experimental details for the synthesis of *sym-p/m/o*-bTAA)$_2$ **4**, **5**, **6** and **7**.

entry	*sym-p/m/o*-bTAA **1**, **2** or **3**	(*sym-p/m/o*-bTAA)$_2$ **4**, **5**, **6** or **7**
1[a,b]	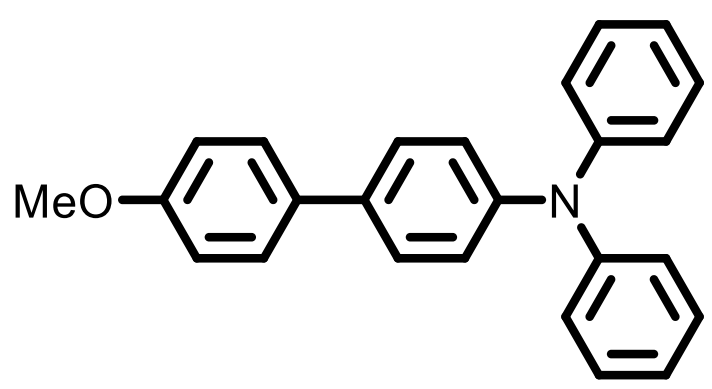 141 mg (0.40 mmol) of **1a**	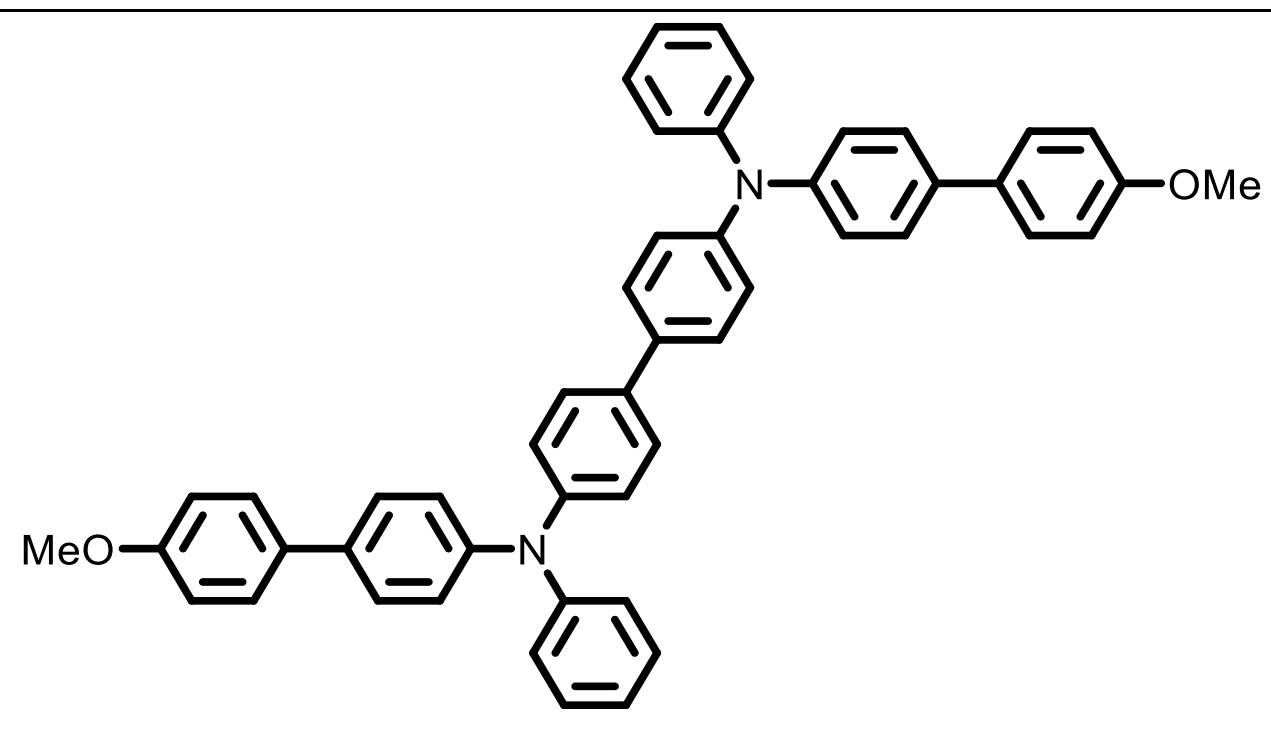 47.0 mg (34%) of **4a**

[a] Yields after flash chromatography on silica gel.

[b] Reduction in batch size due to only a small amount of starting material remaining.

Table S2. Experimental details for the synthesis of *sym-p*/*m*/*o*-bTAA)$_2$ **4**, **6** and **7**.

entry	*sym-p*/*m*/*o*-bTAA **1**, **2** or **3**	(*sym-p*/*m*/*o*-bTAA)$_2$ **4**, **5**, **6** or **7**
2[a]	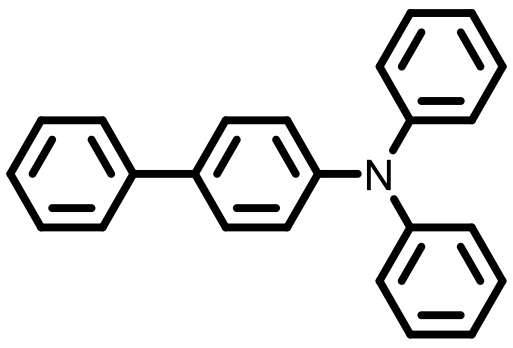 161 mg (0.50 mmol) of **1b**	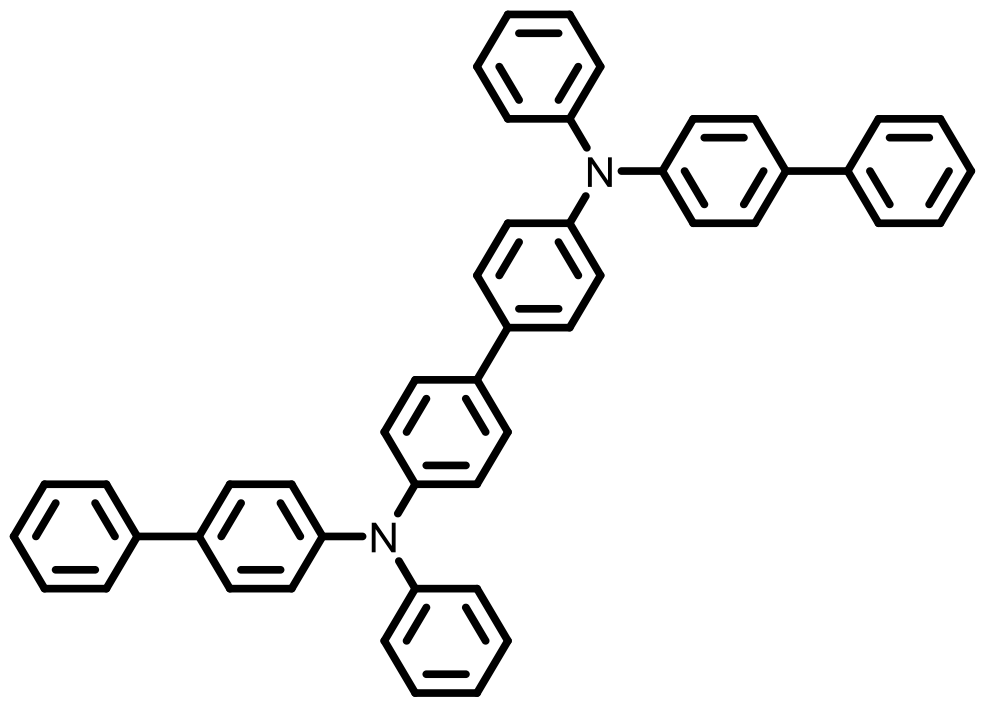 153 mg (96%) of **4b**
3[a,b]	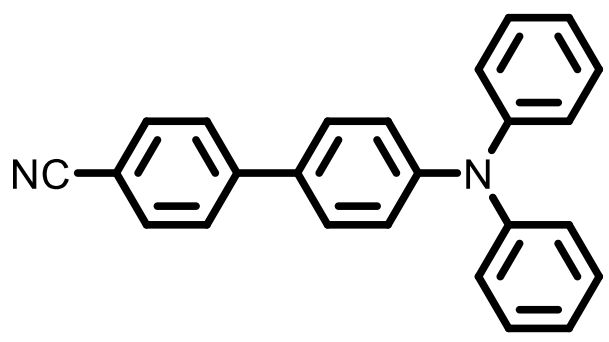 69.0 mg (0.20 mmol) of **1c**	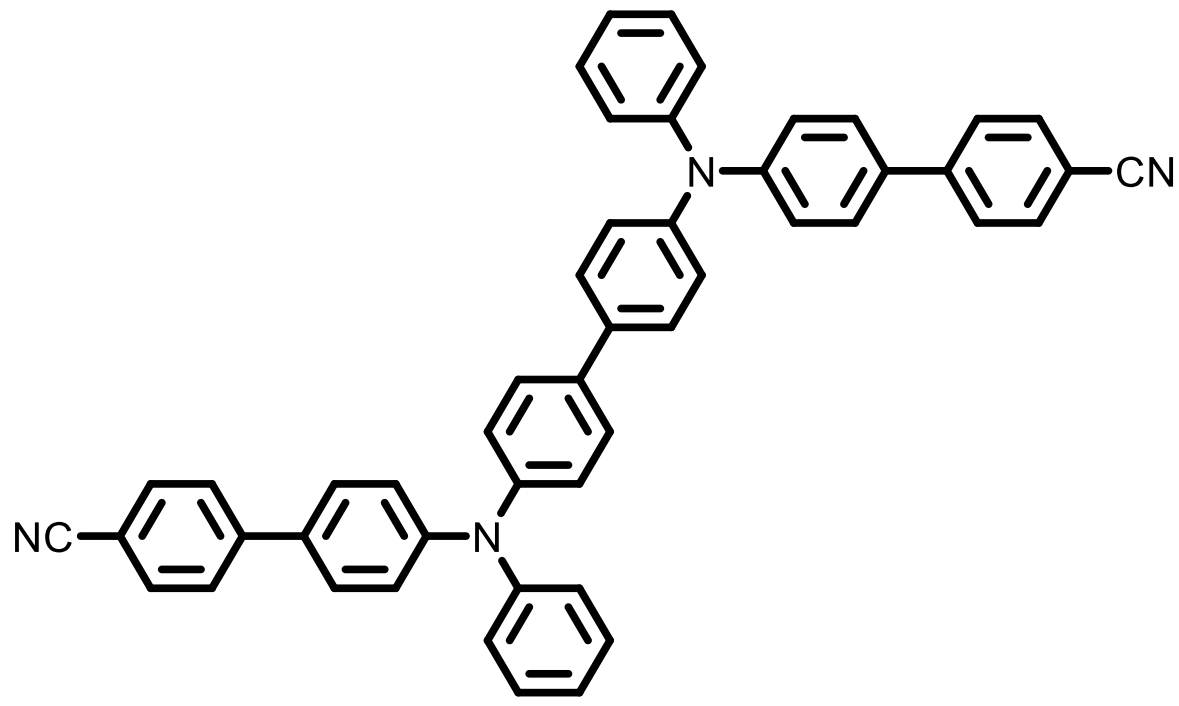 64.0 mg (93%) of **4c**
4[a]	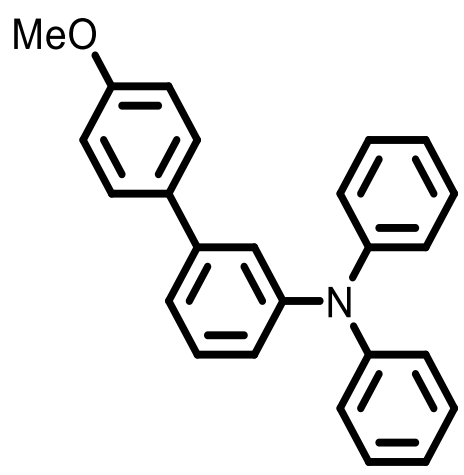 176 mg (0.50 mmol) of **2a**	128 mg (73%) of **5a/5a'/5a''**
5[a]	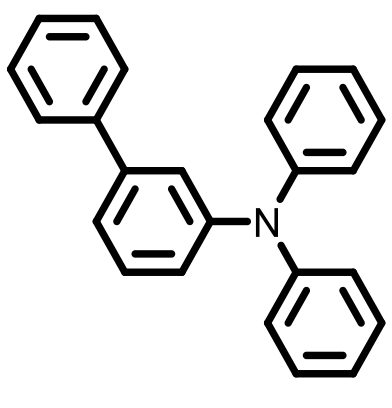 161 mg (0.50 mmol) of **2b**	210 mg (99%) of **5b/5b'/5b''**

[a] Yields after flash chromatography on silica gel.

[b] Reduction in batch size due to only a small amount of starting material remaining.

Table S2. Experimental details for the synthesis of *sym-p*/*m*/*o*-bTAA)$_2$ **4**, **5**, **6** and **7**.

entry	*sym-p*/*m*/*o*-bTAA **1**, **2** or **3**	(*sym-p*/*m*/*o*-bTAA)$_2$ **4**, **5**, **6** or **7**
6[a,b]	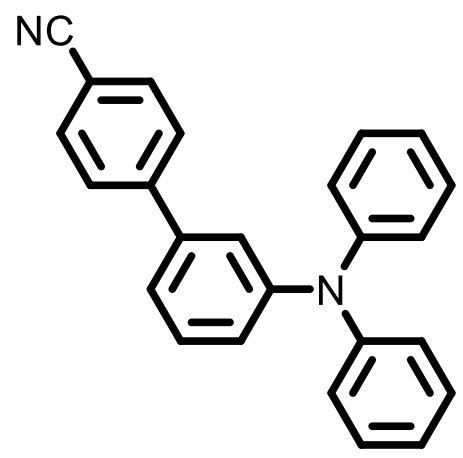 69.0 mg (0.20 mmol) of **2c**	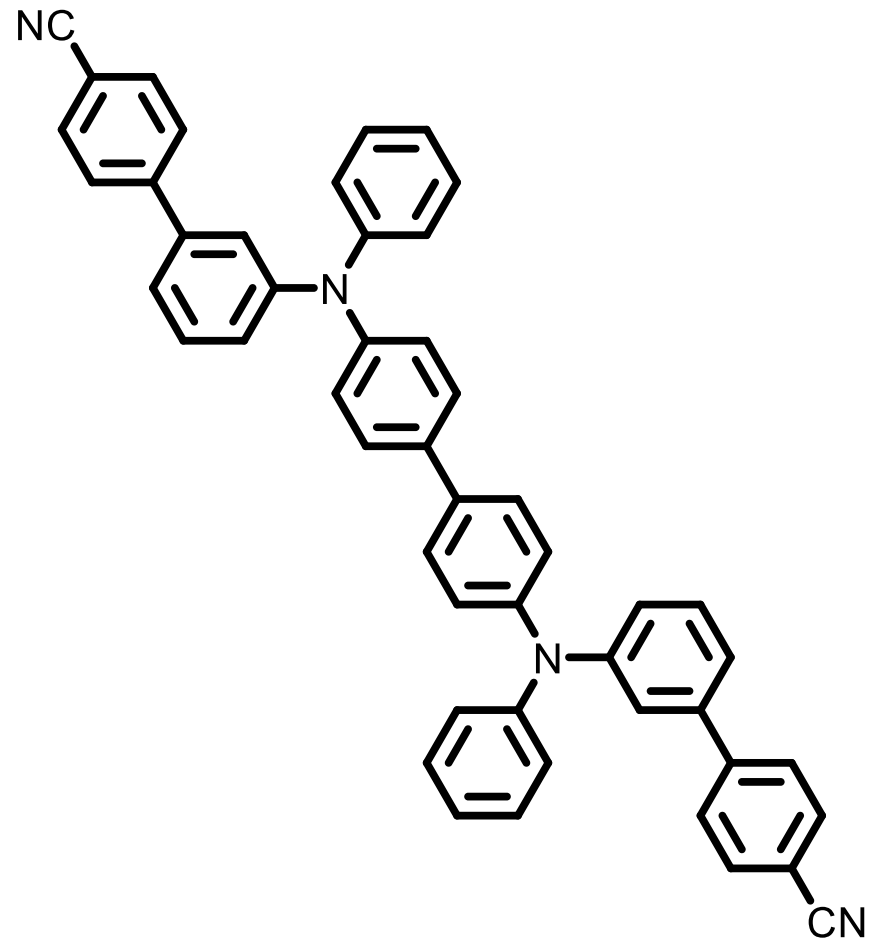 20.0 mg (29%) of **5c**
7[a,b]	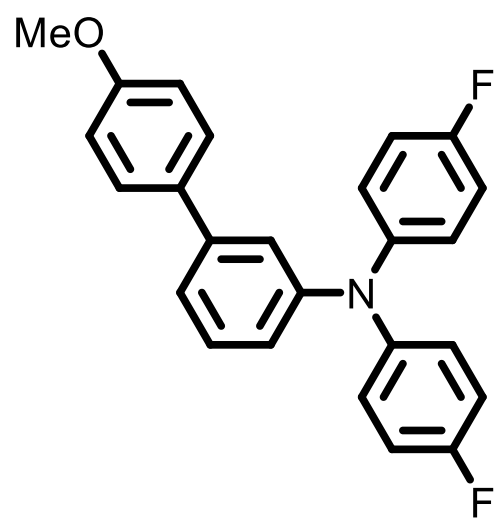 77.0 mg (0.20 mmol) of **2d**	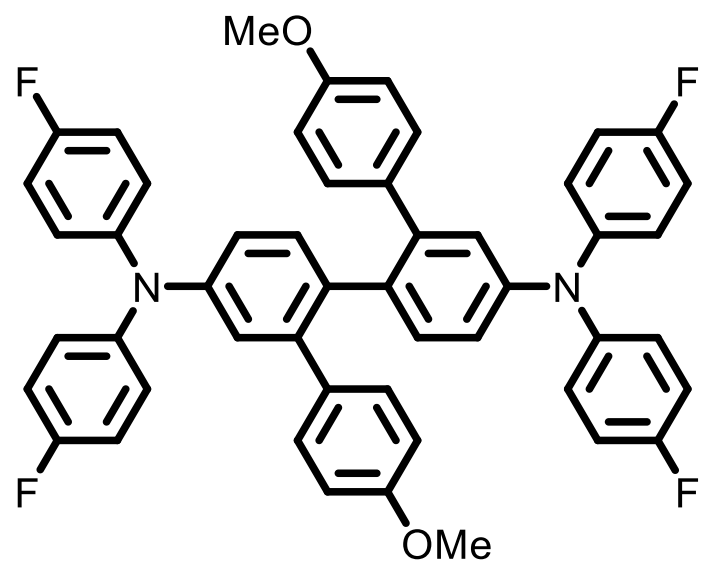 67.0 mg (87%) of **5d**
8[a,c]	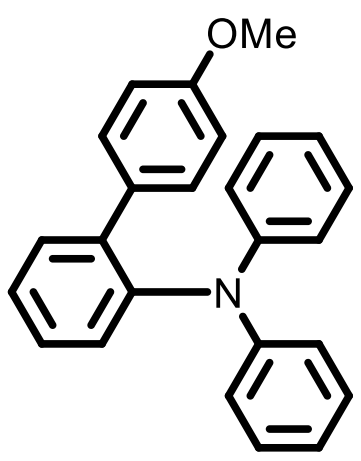 281 mg (0.80 mmol) of **3a**	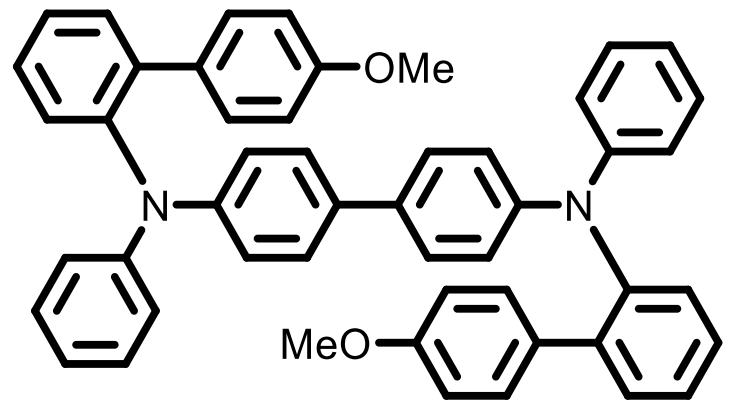 301 mg (99%) of **6a**
9[a,b]	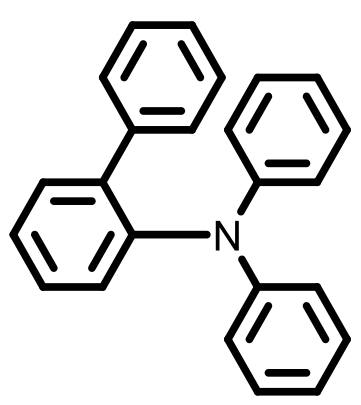 64.0 mg (0.20 mmol) of **3b**	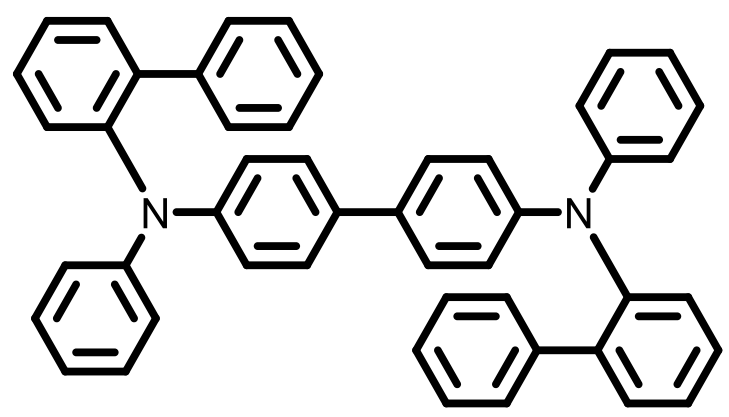 68.0 mg (99%) of **6b**
10[a,b]	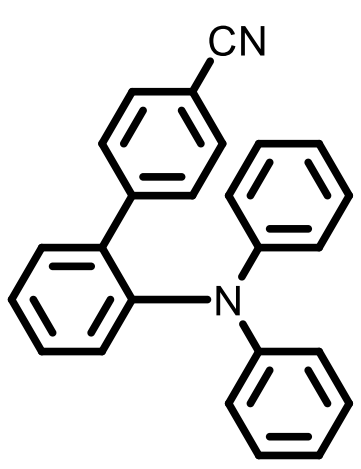 69.0 mg (0.20 mmol) of **3c**	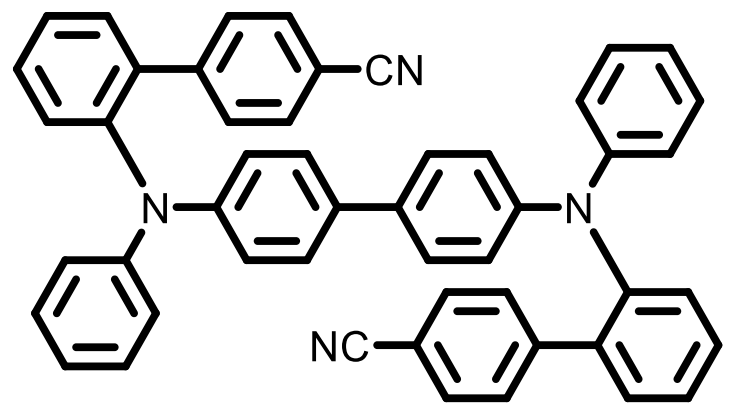 40.0 mg (58%) of **6c**

Table S2. Experimental details for the synthesis of *sym-p*/*m*/*o*-bTAA)$_2$ **4**, **5**, **6** and **7**.

entry	*sym-p*/*m*/*o*-bTAA **1**, **2** or **3**	(*sym-p*/*m*/*o*-bTAA)$_2$ **4**, **5**, **6** or **7**
11[a]	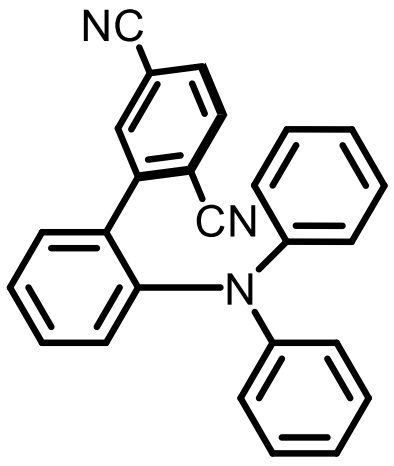<br>186 mg (0.50 mmol) of **3d**	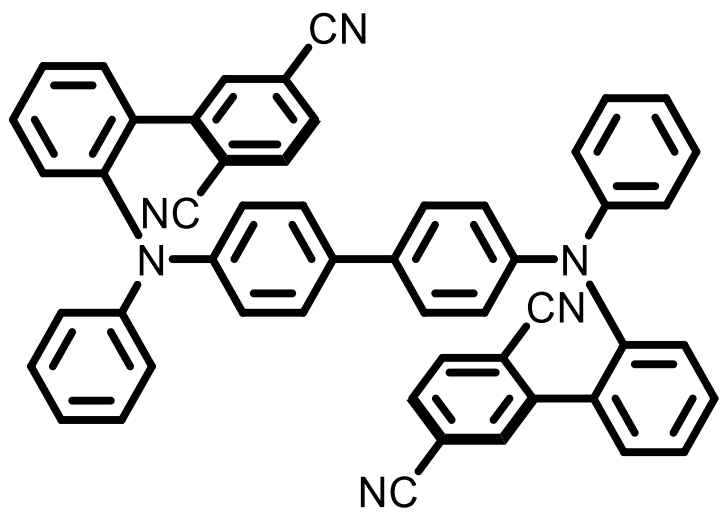<br>152 mg (96%) of **6d**
12[a]	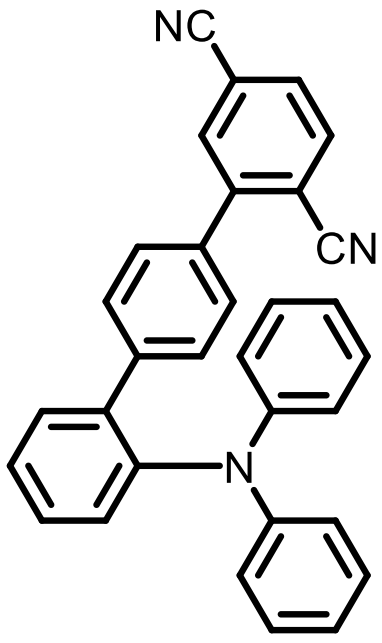<br>224 mg (0.50 mmol) of **3e**	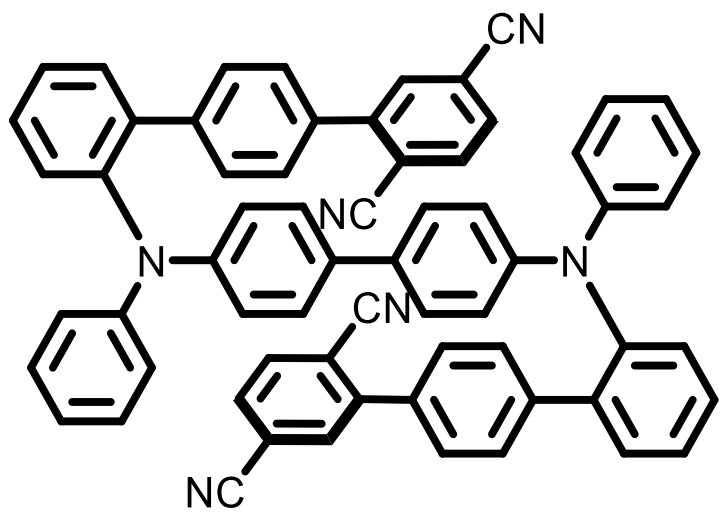<br>222 mg (99%) of **6e**
13[a]	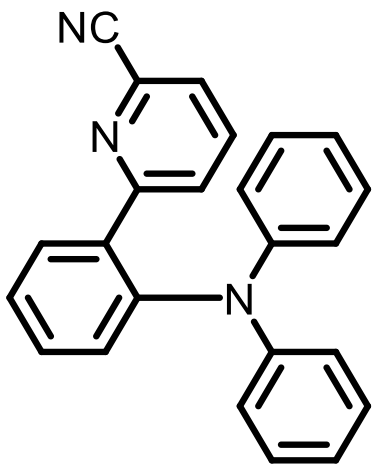<br>174 mg (0.50 mmol) of **3f,**	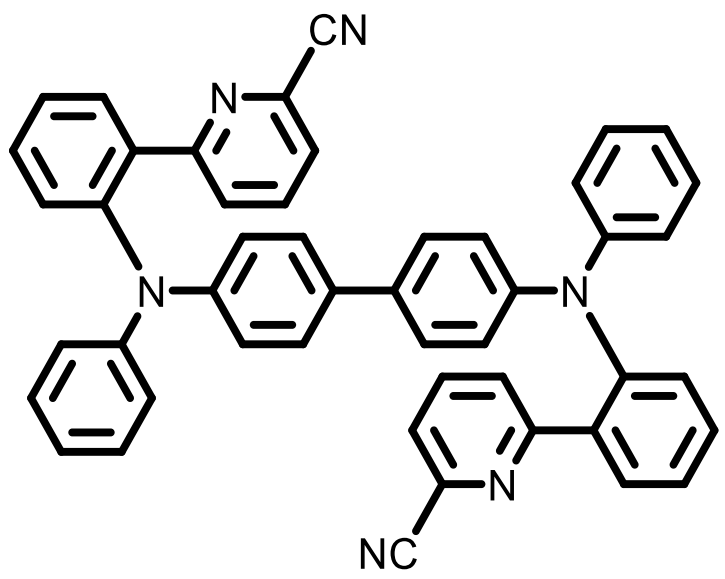<br>153 mg (88%) of **7a**
14[a]	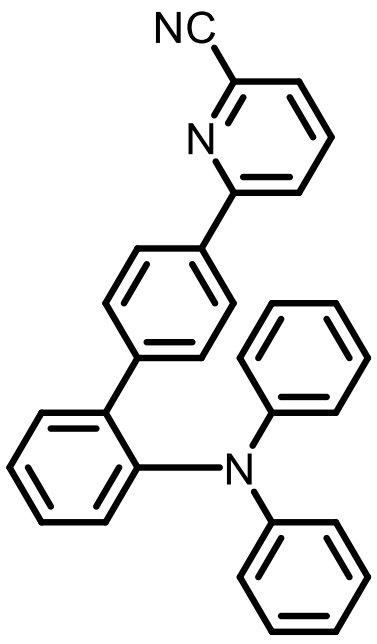<br>212 mg (0.50 mmol) of **3g**	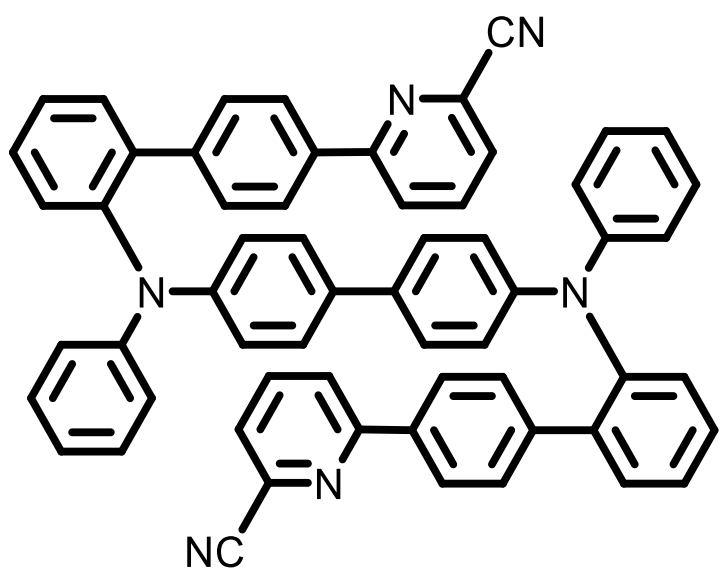<br>175 mg (83%) of **7b**

[a] Yields after flash chromatography on silica gel.
[b] Reduction in batch size due to only a small amount of starting material remaining.
[c] Larger batch size, as test substance.

3.2 Spectroscopic data of *para-/meta-/ortho*-triarylamine dimers (*sym-p-/m-/o*-bTAA)$_2$ 4, 5, 6 and 7

N^4,$N^{4'}$-Bis(4'-methoxy-[1,1'-biphenyl]-4-yl)-N^4,$N^{4'}$-diphenyl-[1,1'-biphenyl]-4,4'-diamine (4a)

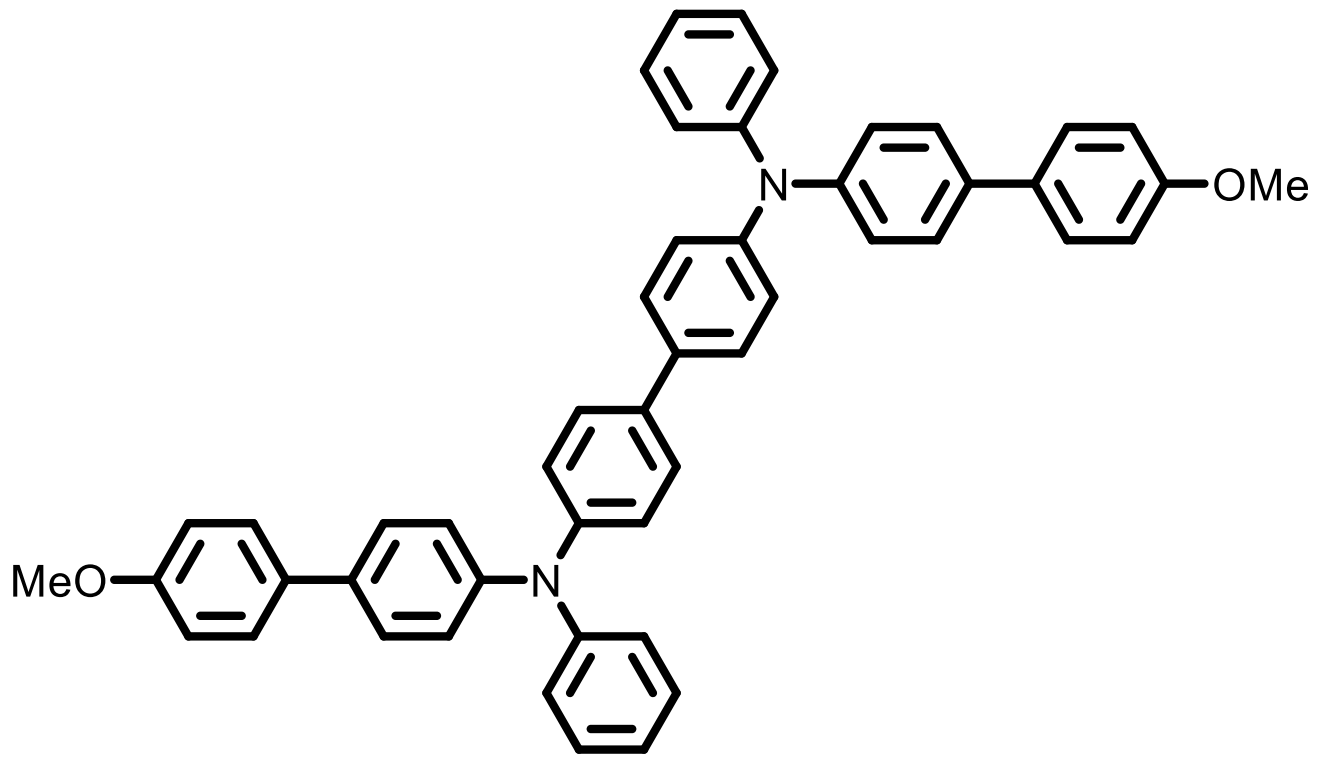

The synthesis was performed by GP2. After chromatography on silica gel (*n*-hexane/ethyl acetate 10:1) and recrystallization from *n*-hexane compound **4a** (47.0 mg, 0.07 mmol, 34%) was isolated as colorless crystals, Mp 235 °C. R_f 0.24 (*n*-hexane/ethyl acetate 10:1).

^{1}H NMR (300 MHz, benzene-d$_6$) δ 3.34 (s, 6 H), 6.82-6.87 (m, 4 H), 6.89 (tt, ^3J = 7.3 Hz, ^4J = 1.2 Hz, 2 H), 7.09-7.13 (m, 4 H), 7.19-7.24 (m, 12 H), 7.39-7.46 (m, 12 H). ^{13}C NMR (150 MHz, benzene-d$_6$) δ 54.9 (CH_3), 114.7 (CH), 123.2 (CH), 124.7 (CH), 124.8 (CH), 125.2 (CH), 128.3 (CH), 129.7 (CH), 133.7 (C_{quat}), 135.5 (C_{quat}), 136.1 (C_{quat}), 147.0 (C_{quat}), 147.3 (C_{quat}), 148.3 (C_{quat}), 159.6 (C_{quat}). EI MS (70 eV, m/z (%)) 702 (53), 701 ([M]$^+$, 100), 700 (21), 350 ([$C_{25}H_{20}NO$]$^+$, 58), 343 (51), 335 ([$C_{24}H_{17}NO$]$^+$, 51). IR ($\tilde{\nu}$ [cm^{-1}]) 1591 (m), 1558 (w), 1489 (s), 1383 (w), 1325 (m), 1279 (m), 1250 (m), 1179 (m), 1115 (w), 1074i (w), 1038 (m), 999 (w), 818 (s), 806 (m), 754 (m), 735 (w), 696 (m). HRMS (ESI) calcd. for [$C_{50}H_{40}N_2O_2$]$^+$: 700.3084; Found 700.3084.

N^4,$N^{4'}$-Di([1,1'-biphenyl]-4-yl)-N^4,$N^{4'}$-diphenyl-[1,1'-biphenyl]-4,4'-diamine (4b)

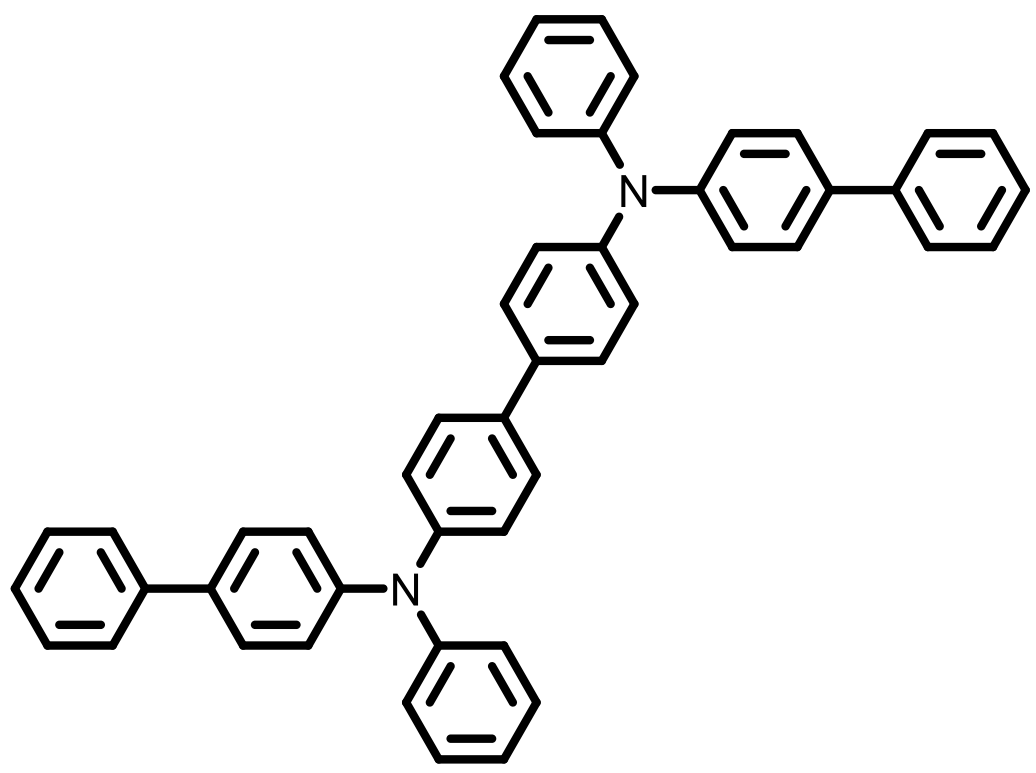

The synthesis was performed by GP2. After chromatography on silica gel (*n*-hexane/ethyl acetate 10:1) and recrystallization from *n*-hexane compound **4b** (153 mg, 0.23 mmol, 96%) was isolated as colorless crystals.

Mp 210 °C. R_f 0.55 (*n*-hexane/ethyl acetate 10:1). ^{1}H NMR (600 MHz, benzene-d$_6$) δ 6.92 (m, 2 H), 7.07-7.12 (m, 4 H), 7.12-7.15 (m, 2 H), 7.16-7.20 (m, 12 H), 7.23 (t, ^3J = 7.7 Hz, 4 H), 7.37-7.44 (m, 8 H), 7.48-7.51 (m, 4 H). ^{13}C NMR (150 MHz, benzene-d$_6$) δ 123.4 (CH), 124.8 (CH), 124.9 (CH), 124.9 (CH), 127.1 (CH), 127.2 (CH), 128.0 (CH), 128.4 (CH), 129.1 (CH), 129.7 (CH), 135.6 (C_{quat}), 136.1 (C_{quat}), 141.2 (C_{quat}), 147.2 (C_{quat}), 147.6 (C_{quat}), 148.2 (C_{quat}). EI MS (70 eV, m/z (%)) 642 (15), 641 (56), 640 ([M]$^+$, 100). IR ($\tilde{\nu}$ [cm^{-1}]) 1591 (m), 1516 (w), 1481 (s), 1452 (w), 1406 (w), 1383 (w), 1325 (m), 1277 (m), 1263 (m), 1233 (w), 1177 (w), 1155 (w), 1074 (m), 1057 (m), 1028 (m), 1005 (w), 972 (w), 916 (w), 893 (w), 870 (w), 831 (m), 820 (m), 760 (m), 750 (m), 725 (m), 696 (s), 664 (w), 640 (w), 625 (w). HRMS (ESI) calcd. for $[C_{48}H_{36}N_2+H]^+$: 641.2951; Found: 641.2946.

4',4'''-([1,1'-Biphenyl]-4,4'-diylbis(phenylazanediyl))bis(([1,1'-biphenyl]-4-carbonitrile)) (4c)

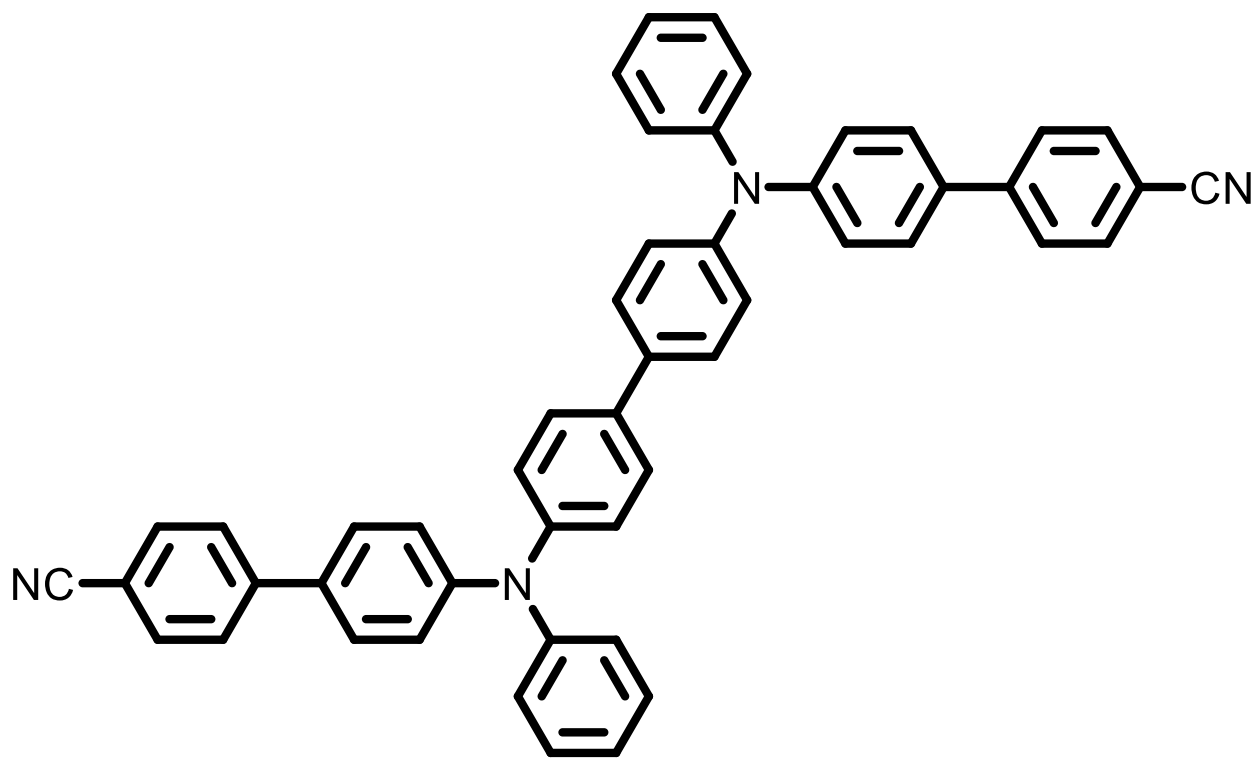

The synthesis was performed by GP2. After chromatography on silica gel (*n*-hexane/ethyl acetate 10:1) and recrystallization from *n*-hexane compound **4c** (64.0 mg, 0.09 mmol, 46%) was isolated as yellow crystals.

Mp 252 °C. R_f 0.13 (*n*-hexane/ethyl acetate 10:1). ^{1}H NMR (600 MHz, acetone-d$_6$) δ 7.15 (tt, ^3J = 7.3 Hz, ^4J = 1.1 Hz, 2 H), 7.17-7.21 (m, 12 H), 7.36-7.41 (m, 4 H), 7.63-7.69 (m, 4 H), 7.69-7.73 (m, 4 H), 7.81-7.87 (m, 4 H), 7.87-7.90 (m, 4 H). ^{13}C NMR (150 MHz, acetone-d$_6$) δ 111.0 (C_{quat}), 119.5 (C_{quat}), 124.0 (CH), 124.8 (CH), 125.7 (CH), 126.1 (CH), 127.9 (CH), 128.4 (CH), 128.9 (CH), 130.5 (CH), 133.3 (C_{quat}), 133.6 (CH), 136.2 (C_{quat}), 145.6 (C_{quat}), 147.4 (C_{quat}), 148.1 (C_{quat}), 148.6 (C_{quat}). EI MS (70 eV, m/z (%)) 692 (16), 691 (58), 690 ([M]$^+$, 100), 345 ($[C_{25}H_{17}N_2]^+$, 14). IR ($\tilde{\nu}$ [cm^{-1}]) 2220 (m), 1587 (m), 1518 (w), 1489 (s), 1452 (w), 1395 (w), 1381 (w), 1323 (m), 1275 (s), 1177 (m), 1153 (w), 1107 (w), 1076 (m), 1057 (m), 1028 (w), 1016 (w), 1003 (w), 962 (w), 951 (w), 920 (w), 893 (w), 858 (w), 820 (s), 752 (s), 741 (w), 721 (w), 696 (s), 662 (w), 621 (w). HRMS (ESI) calcd. for $[C_{50}H_{34}N_4+H]^+$: 691.2856; Found 691.2845.

N^4,$N^{4'}$-Bis(4'-methoxy-[1,1'-biphenyl]-3-yl)-N^4,$N^{4'}$-diphenyl-[1,1'-biphenyl]-4,4'-diamine (5a/5a'/5a'')

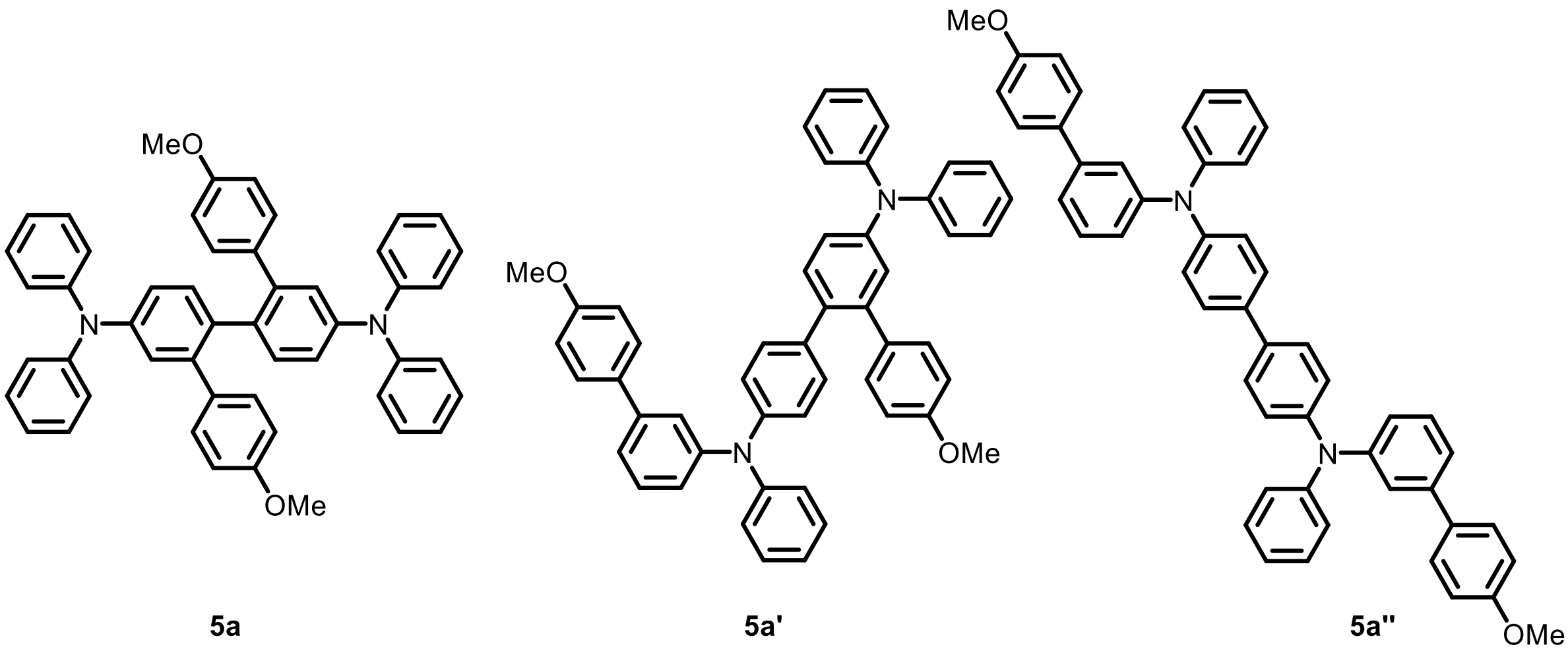

5a **5a'** **5a''**

The synthesis was performed by GP2. After chromatography on silica gel (*n*-hexane/ethyl acetate 10:1) and recrystallization from *n*-hexane compounds **5a/5a'/5a''** (128 mg, 0.18 mmol, 73%) were isolated as colorless crystals, Mp 223 °C. R_f 0.54 (*n*-hexane/ethyl acetate 5:1).

^{1}H NMR (600 MHz, benzene-d$_6$) δ 3.23 (s, 1.5 H), 3.29 (s, 0.6 H), 3.31 (s, 6 H), 3.33 (s, 1.50 H), 6.41-6.45 (m, 0.2 H), 6.42-6.53 (m, 4 H), 6.51-6.58 (m, 0.4 H), 6.58-6.62 (m, 1 H), 6.66-6.73 (m, 4 H), 6.79-6.87 (m, 7 H), 6.94-6.98 (m, 1 H), 7.03 (dd, ^3J = 8.3 Hz, ^4J = 2.3 Hz, 3.5 H), 7.04-7.08 (m, 11 H), 7.07-7.15 (m, 4 H), 7.16-7.20 (m, 11.5 H), 7.24 (d, ^4J = 2.4 Hz, 2 H), 7.30 (d, ^3J = 8.3 Hz, 2 H), 7.32 (d, ^3J = 8.3 Hz, 0.5 H), 7.33-7.40 (m, 1.5 H), 7.45 (d, ^4J = 2.4 Hz, 0.5 H), 7.53 (t, ^4J = 2.0 Hz, 0.5 H), 7.62-7.64 (m, 0.1 H). ^{13}C NMR (150 MHz, benzene-d$_6$) δ 54.9 (CH$_3$), 54.9 (CH$_3$), 55.0 (CH$_3$), 55.0 (CH$_3$), 113.5 (CH), 114.0 (CH), 114.8 (CH), 121.8 (CH), 123.2 (CH), 123.3 (CH), 123.3 (CH), 123.6 (CH), 124.0 (CH), 124.7 (CH), 124.9 (CH), 125.0 (CH), 126.3 (CH), 126.8 (CH), 128.6 (CH), 129.7 (CH), 129.8 (CH), 129.8 (CH), 130.2 (CH), 130.9 (CH), 131.4 (CH), 131.4 (CH), 132.1 (CH), 133.0 (CH), 134.0 (C$_{quat}$), 134.0 (C$_{quat}$), 134.4 (C$_{quat}$), 135.3 (C$_{quat}$), 136.4 (C$_{quat}$), 141.9 (C$_{quat}$), 142.7 (C$_{quat}$), 142.8 (C$_{quat}$), 146.8 (C$_{quat}$), 147.8 (C$_{quat}$), 148.5 (C$_{quat}$), 149.0 (C$_{quat}$), 159.0 (C$_{quat}$), 159.3 (C$_{quat}$), 160.0 (C$_{quat}$).EI MS (70 eV, m/z (%)) 702 (24), 701 (73), 700 ([M]$^+$, 100), 351 ([C$_{25}$H$_{20}$NO]$^+$, 13). IR ($\tilde{\nu}$ [cm^{-1}]) 3034 (w), 1587 (m), 1514 (m), 1476 (s), 1332 (m), 1314 (m), 1283 (m), 1244 (s), 1177 (m), 1109 (m), 1030 (m), 829 (s), 806 (m), 750 (s), 694 (s), 654 (m), 627 (m). Anal. calcd. for $C_{50}H_{40}N_2O_2$ [700.9]: C 85.68, H 5.75, N 4.00; Found C 85.63, H 5.95, N 3.79.

N^4,$N^{4'}$-Di([1,1'-biphenyl]-3-yl)-N^4,$N^{4'}$-diphenyl-[1,1'-biphenyl]-4,4'-diamine (5b/5b'/5b'')

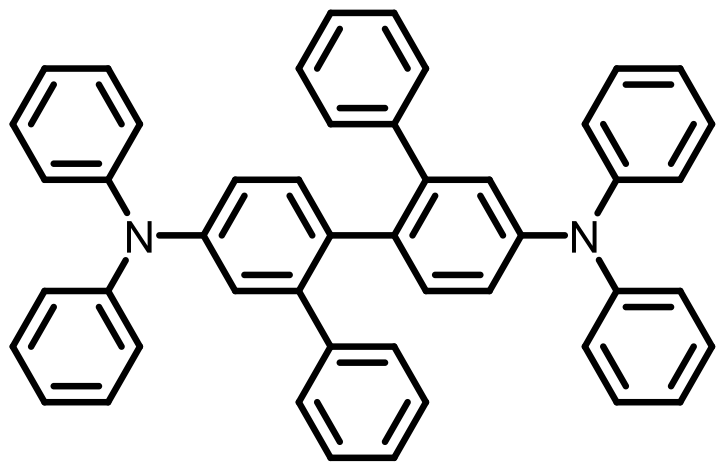

5b

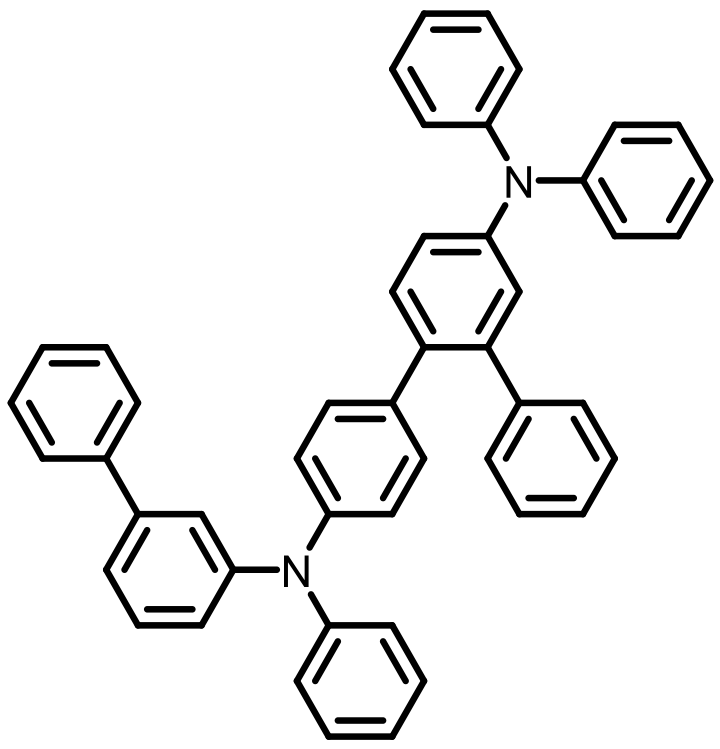

5b'

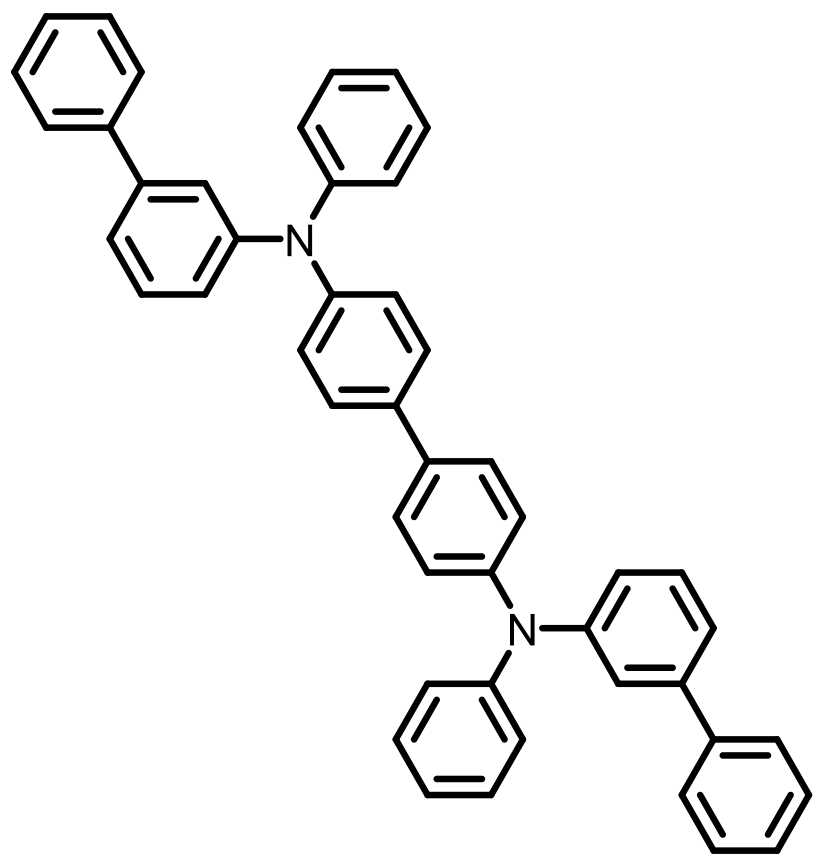

5b''

The synthesis was performed by GP2. After chromatography on silica gel (*n*-hexane/ethyl acetate 15:1) and recrystallization from *n*-hexane compounds **5b/5b'/5b''** (159 mg, 0.25 mmol, 99%) were isolated as colorless crystals, Mp 121 °C. R_f 0.50 (*n*-hexane/ethyl acetate 10:1)

^{1}H NMR (600 MHz, acetone-d$_6$) δ 7.05-7.11 (m, 4 H), 7.14-7.18 (m, 8 H), 7.30-7.37 (m, 8 H), 7.38 (t, ^3J = 2.0 Hz, 2 H), 7.38-7.44 (m, 6 H), 7.54-7.57 (m, 4 H), 7.61-7.64 (m, 4 H). ^{13}C NMR (150 MHz, acetone-d$_6$) δ 122.4 (CH), 123.3 (CH), 123.8 (CH), 124.2 (CH), 125.0 (CH), 125.4 (CH), 127.7 (CH), 128.2 (CH), 128.4 (CH), 129.7 (CH), 130.4 (CH), 130.8 (CH), 135.6 (C$_{quat}$), 141.5 (C$_{quat}$), 143.2 (C$_{quat}$), 147.7 (C$_{quat}$), 148.6 (C$_{quat}$), 149.2 (C$_{quat}$). EI MS (70 eV, m/z (%)) 642 (18), 641 (64), 640 ([M]$^+$, 100). IR ($\tilde{\nu}$ [cm^{-1}]) 1591 (m), 1568 (w), 1487 (m), 1474 (m), 1456 (w), 1422 (w), 1329 (w), 1308 (w), 1269 (m), 1254 (m), 1175 (w), 1113 (w), 1026 (w), 995 (w), 941 (w), 912 (w), 887 (w), 827 (m), 789 (w), 750 (s), 692 (s), 667 (w), 638 (w), 615 (m). HRMS (ESI) calcd. for [$C_{48}H_{36}N_2$+H]$^+$: 641.2912; Found 641.2935.

3',3'''-([1,1'-Biphenyl]-4,4'-diylbis(phenylazanediyl))bis(([1,1'-biphenyl]-4-carbonitrile)) (5c)

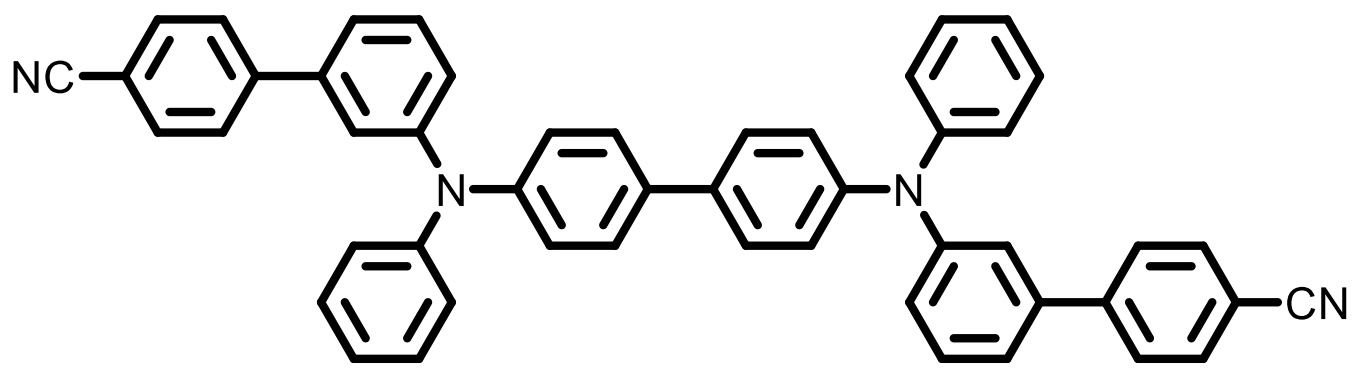

The synthesis was performed by GP2. After chromatography on silica gel (*n*-hexane/ethyl acetate 10:1) and recrystallization from *n*-hexane compound **5c** (67.0 mg, 0.09 mmol, 87%) was isolated as colorless crystals, Mp 262 °C. R_f 0.16 (*n*-hexane/ethyl acetate 10:1).

^{1}H NMR (600 MHz, benzene-d$_6$) δ 6.86-6.91 (m, 4 H), 6.92-6.97 (m, 8 H), 7.04-7.09 (m, 10 H), 7.11-7.15 (m, 8 H), 7.26-7.44 (m, 6 H). ^{13}C NMR (150 MHz, benzene-d$_6$) δ 111.6 (C$_{quat}$), 118.8 (C$_{quat}$), 121.9 (CH), 123.1 (CH), 123.7 (CH), 124.5 (CH), 124.8 (CH), 124.9 (CH), 127.5 (CH), 128.3 (CH), 129.9 (CH), 130.3 (CH), 132.5 (CH), 135.7 (C$_{quat}$), 141.0 (C$_{quat}$), 144.7 (C$_{quat}$), 147.2 (C$_{quat}$), 148.0 (C$_{quat}$), 149.1 (C$_{quat}$). Maldi MS (*m/z* (%)) 690.29 ([M]$^+$, 100). IR ($\tilde{\nu}$ [cm^{-1}]) 2226 (m), 1647 (w), 1593 (s), 1580 (m), 1553 (w), 1491 (s), 1476 (s), 1439 (w), 1425 (w), 1402 (w), 1395 (w), 1335 (m), 1306 (m), 1277 (m), 1269 (m), 1250 (m), 1169 (w), 1157 (w), 1138 (w), 1112 (w), 1076 (w), 1028 (w), 1016 (w), 995 (w), 970 (w), 941 (w), 891 (w), 839 (s), 831 (s), 793 (s), 760 (s), 737 (w), 719 (w), 696 (s), 667 (w), 654 (w). HRMS (ESI) calcd. for [$C_{50}H_{34}N_4$+H]$^+$: 691.2856; Found 691.2855.

$N^{4''}$,$N^{4''}$,$N^{5'}$,$N^{5'}$-Tetrakis(4-fluorphenyl)-4,4'''-dimethoxy-[1,1':2',1'':2'',1'''-quaterphenyl]-4'',5'-diamin (5d)

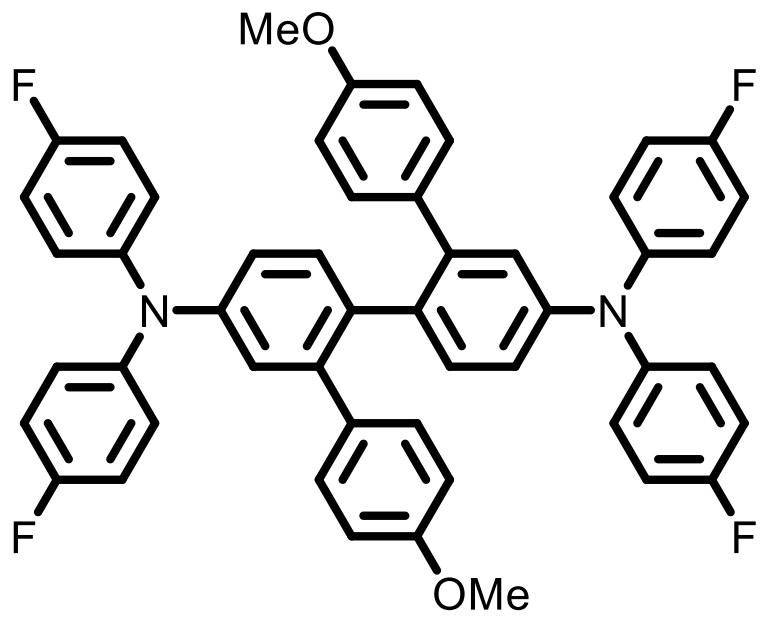

The synthesis was performed by GP2. After chromatography on silica gel (*n*-hexane/ethyl acetate 10:1) and recrystallization from *n*-hexane compound **5d** (67.0 mg, 0.09 mmol, 87%) was isolated as colorless crystals.

Mp 245 °C. R_f 0.38 (*n*-hexane/ethyl acetate 10:1). ^{1}H NMR (600 MHz, benzene-d$_6$) δ 3.28 (s, 6 H), 6.52-6.55 (m, 4 H), 6.66-6.72 (m, 8 H), 6.75-6.79 (m, 4 H), 6.82-6.86 (m, 8 H), 6.87 (dd, ^3J = 8.3 Hz, ^4J = 2.4 Hz, 2 H), 7.10 (d, ^4J = 2.4 Hz, 2 H), 7.32 (d, ^3J = 8.3 Hz, 2 H). ^{13}C NMR (150 MHz, benzene-d$_6$) δ 54.8 (CH_3), 113.4 (CH), 116.4 (d, CH, ^2J = 22.6 Hz), 121.7 (CH), 124.8 (CH), 126.2 (d, CH, ^3J = 7.8 Hz), 130.7 (CH), 132.9 (CH), 133.8 (C_{quat}), 134.6 (C_{quat}), 142.6 (C_{quat}), 144.2 (d, C_{quat}, ^4J = 2.9 Hz), 147.5 (C_{quat}), 159.0 (C_{quat}), 159.3 (d, C_{quat}, ^1J = 243.2 Hz). EI MS (70 eV, m/z (%)) 775 (15), 774 (53), 773 ($[M]^+$, 100), 772 (12), 386 ($[C_{25}H_{18}F_2NO]^+$, 45), 204 ($[C_{12}H_8F_2N]^+$, 43), 203 ($[C_{12}H_7F_2N]^+$, 43). IR ($\tilde{\nu}$ [cm^{-1}]) 1595 (w), 1551 (w), 1503 (s), 1472 (m), 1439 (w), 1427 (w), 1393 (w), 1319 (m), 1287 (m), 1215 (s), 1179 (m), 1153 (m), 1128 (w), 1096 (w), 1055 (w), 1030 (m), 1011 (w), 1001 (w), 951 (w), 928 (w), 893 (w), 878 (w), 829 (s), 800 (m), 758 (w), 741 (w), 706 (m), 689 (w), 650 (w), 627 (m). Anal. calcd. for $C_{50}H_{36}F_4N_2O_2$ [772.8] C 77.71, H 4.70, N 3.62; Found C 77.57, H 4.58, N 3.50.

N^4,$N^{4'}$-Bis(4'-methoxy-[1,1'-biphenyl]-2-yl)-N^4,$N^{4'}$-diphenyl-[1,1'-biphenyl]-4,4'-diamine (6a)

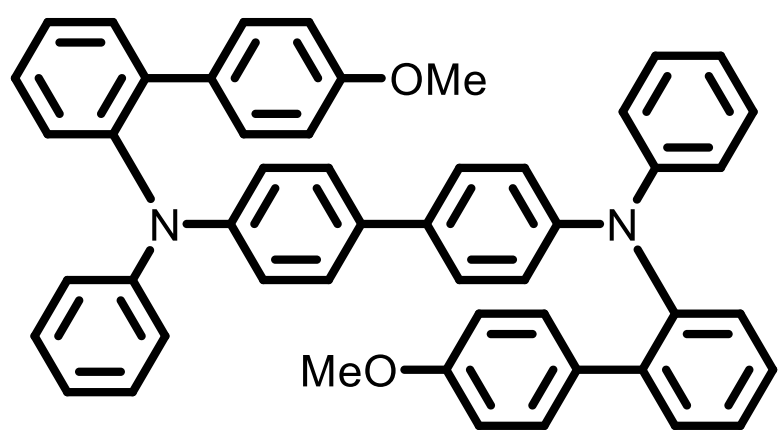

The synthesis was performed by GP2. After chromatography on silica gel (*n*-hexane/ethyl acetate 24:1) and recrystallization from *n*-hexane compound **6a** (278 mg, 0.40 mmol, 99%) was isolated as colorless crystals.

Mp 244 °C. R_f 0.26 (*n*-hexane/ethyl acetate 10:1). ^{1}H NMR (300 MHz, benzene-d$_6$) δ 3.21 (s, 6 H), 6.53-6.60 (m, 4 H), 6.71 (tt, ^4J = 4.5 Hz, 3.5 Hz, 2 H), 6.91-7.02 (m, 12 H), 7.05-7.12 (m, 4 H), 7.20-7.27 (m, 10 H), 7.29-7.38 (m, 2 H). ^{13}C NMR (75 MHz, benzene-d$_6$) δ 54.7 (CH_3), 113.8 (CH), 121.7 (CH), 122.5 (CH), 122.7 (CH), 126.3 (CH), 127.2 (CH), 128.8 (CH), 129.1 (CH), 130.1 (CH), 130.2 (CH), 132.3 (CH), 132.6 (C_{quat}), 134.3 (C_{quat}), 141.0 (C_{quat}), 145.3 (C_{quat}), 147.0 (C_{quat}), 148.0 (C_{quat}), 159.3 (C_{quat}). EI MS (70 eV, m/z (%)) 702 (20), 701 (62), 700 ($[M]^+$, 100), 351 (11), 350 ($[C_{25}H_{20}NO]^+$, 31). IR ($\tilde{\nu}$ [cm^{-1}]) 1608 (w), 1591 (m), 1564 (w), 1514 (w), 1483 (s), 1464 (w), 1441 (w), 1400 (w), 1329 (m), 1304 (m), 1288 (w), 1269 (m), 1240 (s), 1179 (m), 1150 (w), 1080 (w), 1047 (w), 1036 (w), 1018 (w), 1003 (w), 868 (w), 829 (s), 800 (w), 754 (s), 721 (m), 698 (m), 638 (w), 615 (m). HRMS (ESI) calcd. for $[C_{50}H_{40}N_2O_2+H]^+$: 701.3152; Found 701.3163.

N^4,$N^{4'}$-Di([1,1'-biphenyl]-2-yl)-N^4,$N^{4'}$-diphenyl-[1,1'-biphenyl]-4,4'-diamine (6b)

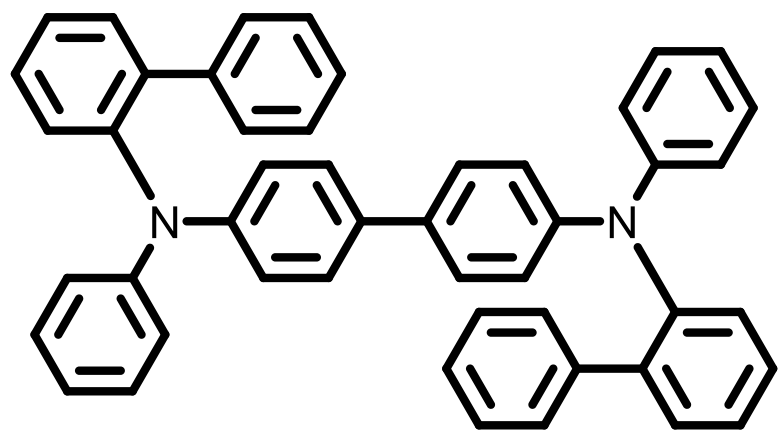

The synthesis was performed by GP2. After chromatography on silica gel (*n*-hexane/ethyl acetate 10:1) and recrystallization from *n*-hexane compound **6b** (159 mg, 0.24 mmol, 99%) was isolated as light green crystals.

Mp 233 °C. R_f 0.57 (*n*-hexane/ethyl acetate 10:1). ^{1}H NMR (600 MHz, chloroform- d$_1$) δ 6.81 (tt, ^3J = 7.3 Hz, ^4J = 1.1 Hz, 2 H), 6.83-6.86 (m, 4 H), 6.86-6.90 (m, 4 H), 7.03-7.07 (m, 4 H), 7.08-7.13 (m, 6 H), 7.15-7.19 (m, 4 H), 7.21-7.24 (m, 4 H), 7.29 (ddd, ^3J = 7.7 Hz, 6.4 Hz, ^4J = 2.1 Hz, 2 H), 7.33-7.40 (m, 6 H). ^{13}C NMR (150 MHz, chloroform- d$_1$) δ 121.5 (CH), 122.2 (CH), 122.3 (CH), 126.0 (CH), 126.8 (CH), 126.8 (CH), 127.9 (CH), 128.7 (CH), 128.8 (CH), 128.9 (CH), 129.7 (CH), 132.0 (CH), 133.7 (C_{quat}), 139.9 (C_{quat}), 140.7 (C_{quat}), 144.9 (C_{quat}), 146.5 (C_{quat}), 147.5 (C_{quat}). Maldi MS (*m/z* (%)) 640.29, 641.29 ($[M]^+$, 100), 642.29, 642.29. IR ($\tilde{\nu}$ [cm^{-1}])

1742 (w), 1591 (m), 1566 (w), 1489 (s), 1477 (s), 1450 (w), 1433 (w), 1412 (w), 1319 (m), 1288 (m), 1271 (m), 1252 (w), 1240 (w), 1179 (w), 1153 (w), 1109 (w), 1076 (w), 1045 (w), 1028 (w), 1009 (w), 908 (w), 885 (w), 874 (w), 818 (w), 775 (w), 741 (s), 694 (s), 671 (w), 619 (m). HRMS (ESI) calcd. for $[C_{48}H_{36}N_2]^+$: 640.2862 ; Found 640.2873.

2',2'''-([1,1'-Biphenyl]-4,4'-diylbis(phenylazanediyl))bis(([1,1'-biphenyl]-4-carbonitrile)) (6c)

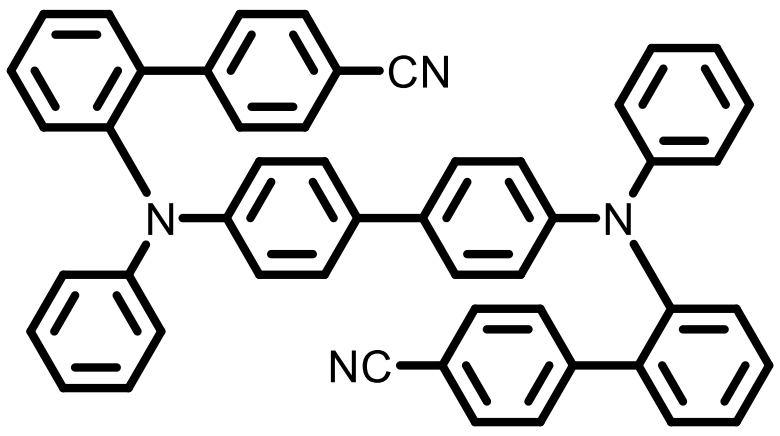

The synthesis was performed by GP2. After chromatography on silica gel (*n*-hexane/ethyl acetate 10:1) and recrystallization from *n*-hexane compound **6c** (40.0 mg, 0.06 mmol, 58%) was isolated as colorless crystals.

Mp 269 °C. R_f 0.20 (*n*-hexane/ethyl acetate 10:1). ^{1}H NMR (300 MHz, benzene-d$_6$) δ 6.69 (tt, ^3J = 7.2 Hz, ^4J = 1.2 Hz, 2 H), 6.71-6.75 (m, 4 H), 6.76-6.80 (m, 4 H), 6.81-6.85 (m, 4 H), 6.87-6.89 (m, 4 H), 6.89-6.93 (m, 4 H), 6.95-7.00 (m, 4 H), 7.05 (ddd, ^3J = 8.0 Hz, 5.3 Hz, ^4J = 3.7 Hz, 2 H), 7.14 (dd, ^3J = 7.9 Hz, ^4J = 1.0 Hz, 2 H), 7.17-7.20 (m, 4 H). ^{13}C NMR (75 MHz, benzene-d$_6$) δ 111.2 (C_{quat}), 118.9 (C_{quat}), 122.3 (CH), 122.7 (CH), 122.9 (CH), 126.0 (CH), 127.3 (CH), 128.0 (CH), 128.3 (CH), 129.2 (CH), 129.5 (CH), 129.7 (CH), 130.0 (CH), 131.7 (CH), 131.8 (CH), 134.5 (C_{quat}), 138.7 (C_{quat}), 144.4 (C_{quat}), 145.4 (C_{quat}), 146.7 (C_{quat}), 147.5 (C_{quat}). Maldi MS (*m/z* (%)) 690 ([M]$^+$, 100), 451. IR ($\tilde{\nu}$ [cm^{-1}]) 2226 (m), 1919 (w), 1593 (m), 1574 (w), 1489 (s), 1479 (s), 1443 (w), 1396 (w), 1323 (m), 1310 (m), 1288 (m), 1267 (m), 1238 (w), 1179 (w), 1153 (w), 1109 (w), 1082 (w), 1030 (w), 1005 (w), 959 (w), 918 (w), 839 (m), 827 (m), 750 (s), 721 (m), 696 (m), 671 (w), 615 (w). HRMS (ESI) calcd. for $[C_{50}H_{34}N_4+H]^+$: 690.2778; Found 690.2759.

2',2'''-([1,1'-Biphenyl]-4,4'-diylbis(phenylazanediyl))bis(([1,1'-biphenyl]-2,5-dicarbonitrile)) (6d)

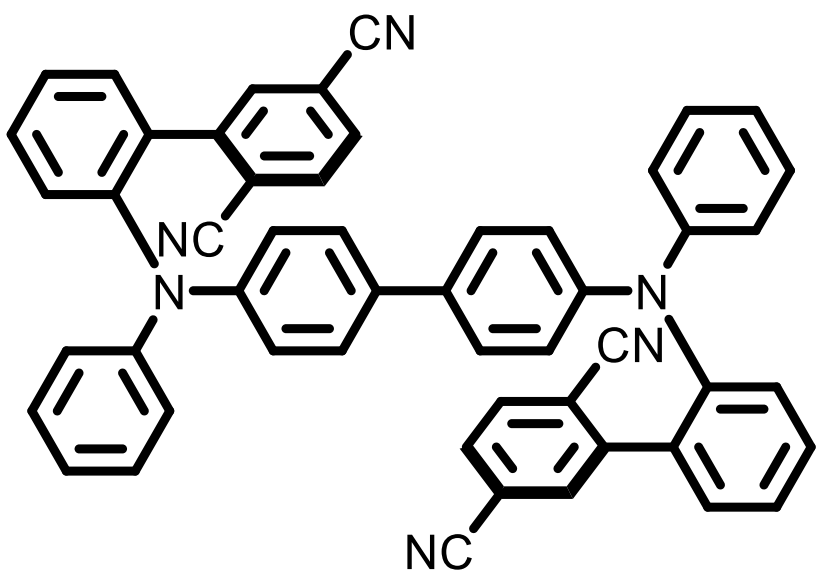

The synthesis was performed by GP2. After chromatography on silica gel (*n*-hexane/ethyl acetate 7:1) and recrystallization from ethanol/acetone-mixture compound **6d** (152 mg, 0.21 mmol, 96%) was isolated as a yellow solid.

Mp 282 °C. R_f 0.44 (*n*-hexane/ethyl acetate 7:1). ^{1}H NMR (600 MHz, chloroform-d$_1$) δ 6.87 – 6.94 (m, 10H), 7.11 – 7.15 (m, 4H), 7.24 – 7.33 (m, 8H), 7.41 (dd, ^3J = 8.1 Hz, ^4J = 1.2 Hz, 2H), 7.43 – 7.49 (m, 6H), 7.51 (d, ^3J = 8.0 Hz, 2H). ^{13}C NMR (151 MHz, chloroform- d$_1$) δ 115.7 (C$_{quat}$), 116.9 (C$_{quat}$), 117.1 (C$_{quat}$), 117.2 (C$_{quat}$), 123.0 (CH), 123.4 (CH), 123.4 (CH), 125.4 (CH), 127.5 (CH), 128.0 (CH), 129.4 (CH), 130.4 (CH), 131.2 (CH), 131.6 (CH), 132.9 (C$_{quat}$), 133.3 (CH), 134.4 (CH), 134.9 (C$_{quat}$), 145.2 (C$_{quat}$), 146.2 (C$_{quat}$), 146.4 (C$_{quat}$), 147.2 (C$_{quat}$). EI MS (70 eV, *m*/*z* (%)): 742 (24), 741 ([M]$^+$, 71), 740 ([M]$^+$, 100), 370 ([$C_{26}H_{16}N_3$]$^+$, 20). IR ($\tilde{\nu}$ [cm^{-1}]) 3071 (w), 3026 (w), 2999 (w), 2980 (w), 2884 (w), 2232 (w), 1940 (w), 1713 (w), 1589 (w), 1572 (w), 1487 (m), 1474 (m), 1447 (w), 1396 (w), 1362 (w), 1319 (m), 1296 (m), 1271 (m), 1250 (w), 1223 (w), 1179 (w), 899 (w), 843 (w), 818 (m), 760 (m), 739 (w), 696 (m), 662 (w), 646 (w), 625 (m). HRMS (ESI) calcd. for [$C_{52}H_{32}N_6$]$^+$: 740.2683; Found 740.2666.

2'',2'''''-([1,1'-Biphenyl]-4,4'-diylbis(phenylazanediyl))bis(([1,1':4',1''-terphenyl]-2,5-dicarbonitrile)) (6e)

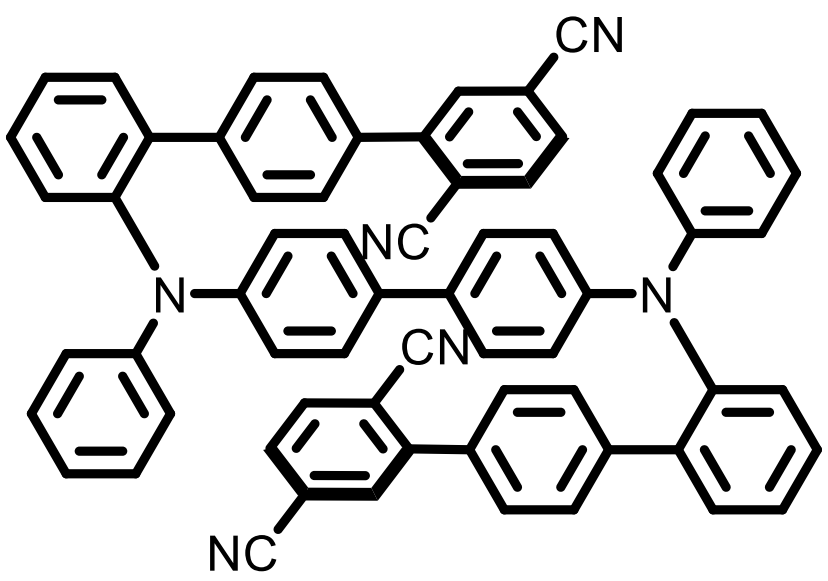

The synthesis was performed by GP2. After chromatography on silica gel (*n*-hexane/ethyl acetate 4:1) and recrystallization from acetone compound **6e** (222 mg, 0.25 mmol, 99%) was isolated as a light-yellow solid, Mp 296 °C. R_f 0.26 (*n*-hexane/ethyl acetate 4:1).

^{1}H NMR (600 MHz, chloroform-d$_1$) δ 6.80 – 6.88 (m, 6H), 6.93 (d, ^3J = 8.2 Hz, 4H), 7.07 (t, ^3J = 7.7 Hz, 4H), 7.26 – 7.36 (m, 14H), 7.37 – 7.43 (m, 6H), 7.66 – 7.71 (m, 4H), 7.84 (dd, ^3J =

8.0 Hz, ^{4}J = 1.5 Hz Hz, 2H). ^{13}C NMR (151 MHz, chloroform-d_1) δ 115.5 (C_{quat}), 116.7 (C_{quat}),117.0 (C_{quat}), 117.2 (C_{quat}), 121.9 (CH), 122.2 (CH), 122.7 (CH), 126.0 (CH), 127.0 (CH), 128.3 (CH), 128.9 (CH), 129.5 (CH), 129.5 (CH), 130.5 (CH), 131.7 (CH), 133.5 (CH), 133.9 (C_{quat}), 134.4 (C_{quat}), 134.5 (CH), 139.2 (C_{quat}), 141.7 (C_{quat}), 145.2 (C_{quat}), 146.5 (C_{quat}), 147.3 (C_{quat}). EI MS (70 eV, *m/z* (%)): 895 (25), 894 (70), 893 ($[M]^+$, 100), 447 (10), 446 ($[C_{32}H_{20}N_3]^+$, 12). IR ($\tilde{\nu}$ [cm^{-1}]) 3082 (w), 3063 (w), 3032 (w), 2953 (w), 2922 (w), 2853 (w), 2230 (w), 2162 (w), 2031 (w), 1946 (w), 1917 (w), 1902 (w), 1593 (w), 1572 (w), 1549 (w), 1489 (m), 1458 (w), 1443 (w), 1412 (w), 1389 (w), 1364 (w), 1317 (w), 1287 (m), 1273 (w), 1179 (w), 1155 (w), 1109 (w), 1078 (w), 1040 (w), 1030 (w), 1020 (w), 1007 (w), 997 (w), 961 (w), 908 (w), 870 (w), 835 (m), 820 (w), 748 (m), 718 (w), 694 (m), 660 (w), 619 (m). HRMS (ESI) calcd. for $[C_{64}H_{40}N_6+H]^+$: 893.3387; Found: 893.3387.

6,6'-(([1,1'-biphenyl]-4,4'-diylbis(phenylazanediyl))bis(2,1-phenylene))dipicolinonitrile (7a)

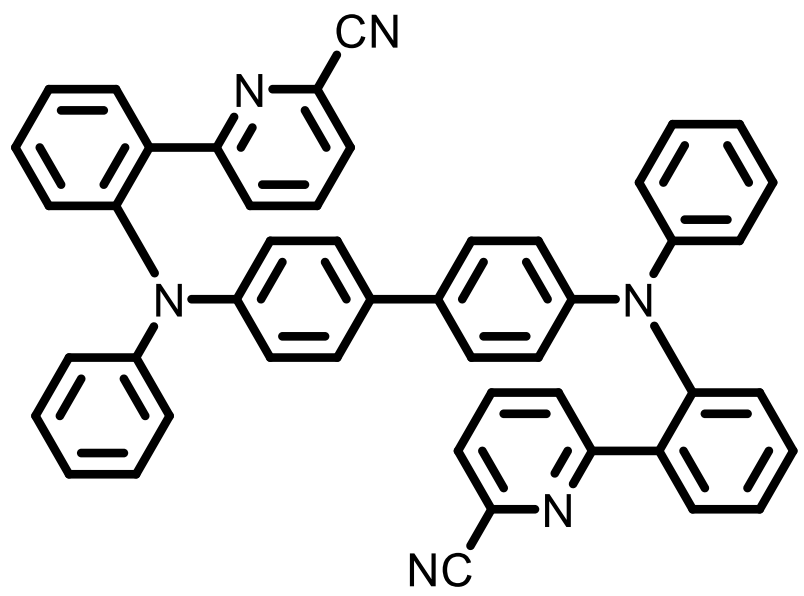

The synthesis was performed by GP2. After chromatography on silica gel (*n*-hexane/ethyl acetate 10:1) and recrystallization from ethanol compound **7a** (153 mg, 0.22 mmol, 88%) was isolated as yellow crystals, Mp 243 °C. R_f 0.33 (*n*-hexane/ethyl acetate 10:1).

^{1}H NMR (600 MHz, chloroform-d$_1$) δ 6.87 (tt, ^3J = 7.3 Hz, ^4J = 1.2 Hz, 2H), 6.96 – 6.91 (m, 8H), 7.14 – 7.09 (m, 4H), 7.27 – 7.23 (m, 4H), 7.30 (dd, ^3J = 8.0 Hz, ^4J = 1.2 Hz, 2H), 7.34 (td, ^3J = 7.5 Hz, ^4J = 1.2 Hz, 2H), 7.38 (dd, ^3J = 7.6 Hz, ^4J = 1.0 Hz, 2H), 7.45 (td, ^3J = 7.7 Hz, ^4J = 1.7 Hz, 2H), 7.57 (t, ^3J = 8.0 Hz, 2H), 7.65 (dd, ^3J = 7.7 Hz, ^4J = 1.6 Hz, 2H), 7.74 (dd, ^3J = 8.0 Hz, ^4J = 1.0 Hz, 2H). ^{13}C NMR (151 MHz, chloroform-d$_1$) δ 117.4 (C$_{quat}$), 122.4 (CH), 122.6 (CH), 122.7 (CH), 126.0 (CH), 126.4 (CH), 127.1 (CH), 127.3 (CH), 129.2 (CH), 129.2 (CH), 131.1 (CH), 131.9 (CH), 133.3 (C$_{quat}$), 134.2 (C$_{quat}$), 135.9 (C$_{quat}$), 136.7 (CH), 145.2 (C$_{quat}$), 146.2 (C$_{quat}$), 147.1 (C$_{quat}$), 159.3 (C$_{quat}$). EI MS (70 eV, *m*/*z* (%)) 695 (13), 694 (49), 693 ([M]$^+$, 100), 692 (42), 347 (25), 346 ([$C_{24}H_{16}N_3$]$^+$, 78), 345 (35), 294 (11), 243 ([$C_{18}H_{13}N$]$^{2+}$, 12), 242 (15), 241 (14), 167 ([$C_{12}H_9N$]$^+$, 11), 77 ([C_6H_5]$^+$, 11). IR ($\tilde{\nu}$ [cm^{-1}]) 3034 (w), 2961 (w), 2870 (w), 2237 (w), 2070 (w), 1890 (w), 1852 (w), 1726 (w), 1715 (w), 1611 (w), 1595 (m), 1574 (m), 1557 (w), 1483 (m), 1449 (m), 1435 (m), 1396 (w), 1364 (w), 1312 (m), 1288 (m), 1258 (m), 1240 (w), 1206 (w), 1180 (w), 1171 (w), 1142 (w), 1113 (w), 1074 (w), 1040 (w), 1028 (w), 988 (w), 951 (w), 924 (w), 910 (w), 893 (w), 851 (w), 814 (m), 768 (m), 752 (m), 737 (m), 714 (w), 694 (m), 685 (m), 652 (w), 623 (m). Anal. calcd. for $C_{48}H_{32}N_6$ [692.8] C 83.21, H 4.66, N 12.13; Found C 82.98, H 4.67, N 11.74.

6,6'-(([1,1'-biphenyl]-4,4'-diylbis(phenylazanediyl))bis([1,1'-biphenyl]-2',4-diyl))dipicolinonitrile (7b)

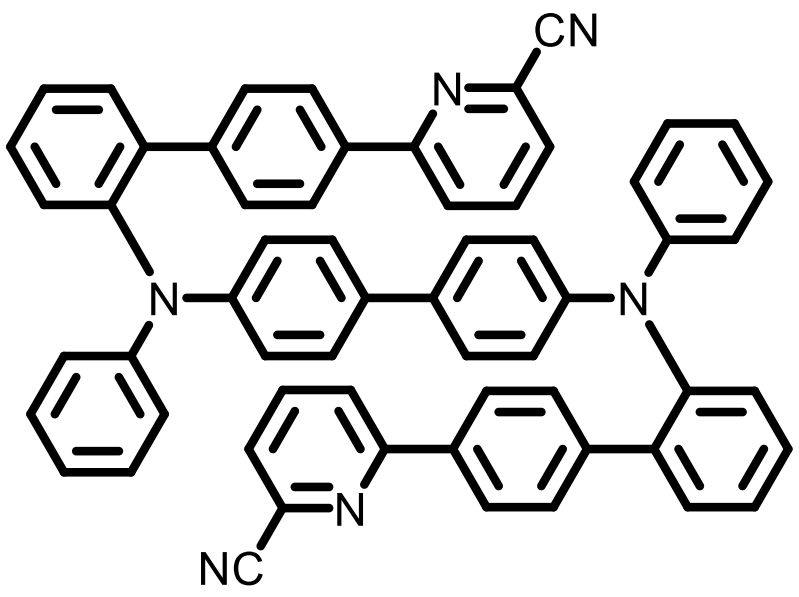

The synthesis was performed by GP2. After chromatography on silica gel (*n*-hexane/ethyl acetate 4:1) and recrystallization from ethanol compound **7b** (175 mg, 0.21 mmol, 83%) was isolated as a colorless solid, Mp 287 °C. R_f 0.33 (*n*-hexane/ethyl acetate 4:1).

^{1}H NMR (600 MHz, benzene-d$_6$) δ 6.57 (dd, ^3J = 7.6 Hz, ^4J = 2.4 Hz, 2H), 6.62 (td, ^3J = 7.9 Hz, ^4J = 2.6 Hz, 2H), 6.67 – 6.71 (m, 2H), 6.89 – 6.96 (m, 12H), 6.99 (dd, ^3J = 8.1 Hz, ^4J = 2.2 Hz, 2H), 7.07 (t, ^3J = 7.5 Hz, 2H), 7.12 (dd, ^3J = 7.5 Hz, ^4J = 2.0 Hz, 2H), 7.19 – 7.25 (m, 6H), 7.30 (dd, ^3J = 7.5 Hz, ^4J = 2.0 Hz, 2H), 7.33 – 7.36 (m, 4H), 7.66 – 7.68 (m, 4H). ^{13}C NMR (151 MHz, benzene-d$_6$) δ 117.9 (C$_{quat}$), 122.0 (CH), 122.3 (CH), 122.6 (CH), 122.8 (CH), 126.0 (CH), 126.3 (CH), 126.9 (CH), 127.3 (CH), 129.2 (CH), 129.5 (CH), 129.6 (CH), 130.1 (CH), 132.1 (CH), 134.2 (C$_{quat}$), 134.4 (C$_{quat}$), 135.8 (C$_{quat}$), 137.0 (CH), 140.1 (C$_{quat}$), 142.2 (C$_{quat}$), 145.4 (C$_{quat}$), 146.9 (C$_{quat}$), 147.8 (C$_{quat}$), 158.1 (C$_{quat}$). EI MS (70 eV, *m*/*z* (%)) 847 (16), 846 (54), 845 ([M]$^+$, 100), 844 (20). IR ($\tilde{\nu}$ [cm^{-1}]) 3061 (w), 1607 (w), 1586 (w), 1551 (w), 1491 (m), 1443 (m), 1418 (w), 1325 (w), 1308 (w), 1267 (w), 1238 (w), 1206 (w), 1184 (w), 1161 (w), 1119 (w), 1109 (w), 1005 (w), 988 (w), 849 (w), 826 (w), 808 (m), 756 (m), 735 (w), 721 (w), 694 (w), 673 (w), 652 (w), 638 (w), 615 (w). HRMS (ESI) calcd. for [$C_{60}H_{40}N_6$+H]$^+$: 845.3387; Found 845.3379.

3.3 Synthesis of the *ortho*-triarylamine dimer (*sym*-*o*-bTAA)$_2$ 7c

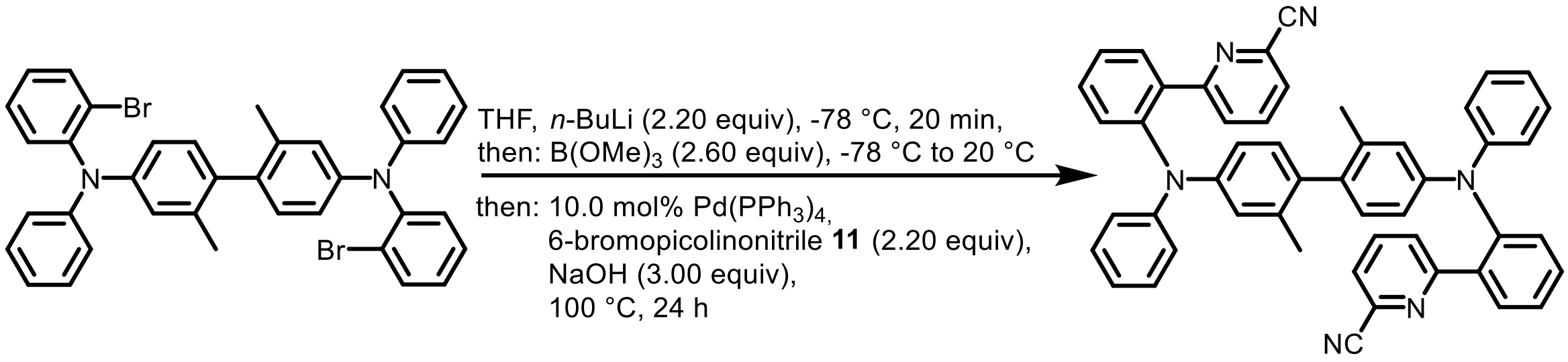

10 **7c**

Under nitrogen in a dried Schlenk tube *sym*-*o*-bTAA-dimer **10** (337 mg, 0.50 mmol, 1.00 equiv) was dissolved in dry THF (3.60 ml) and cooled to −78 °C (dry ice/acetone bath). Then, *n*-butyllithium solution in hexanes (0.78 ml, 1.10 mmol, 2.20 equiv, 1.39 M) was added dropwise and the solution was stirred for 20 min at –78 °C. Afterwards, trimethylborate (0.16 ml, 1.30 mmol, 2.60 equiv) was added and the solution was warmed to 20 °C. Then, $Pd(PPh_3)_4$ (57.8 mg, 0.05 mmol, 10.0 mol%), the acceptor **11** (201 mg, 1.10 mmol, 2.20 equiv) and sodium hydroxide (60.0 mg, 1.50 mmol, 3.00 equiv) was added to the reaction mixture. The solution was stirred at 100 °C (oil bath) for 24 h. After cooling to room temperature, the reaction mixture was diluted with ethyl acetate and purified by flash chromatography on silica gel (*n*-hexane/ethyl acetate 7:1). After recrystallisation from ethanol and then drying under high vacuum compound **7c** (176 mg, 0.24 mmol, 49%), was optained as a light yellow solid, Mp 113 °C. R_f 0.30 (*n*-hexane/ethyl acetate 7:1).

^{1}H NMR (600 MHz, chloroform-d$_1$) δ 1.78 (s, 6H), 6.68 (dd, ^3J = 8.0 Hz, ^4J = 2.6 Hz, 2H), 6.72 – 6.73 (m, 4H), 6.90 (tt, ^3J = 7.3 Hz, ^4J = 1.1 Hz, 2H), 7.02 – 6.99 (m, 4H), 7.18 – 7.14 (m, 4H), 7.29 (dd, ^3J = 8.0 Hz, ^4J = 1.2 Hz, 2H), 7.32 (td, ^3J = 7.5 Hz, ^4J = 1.2 Hz, 2H), 7.36 (dd, ^3J = 7.6 Hz, ^4J = 1.0 Hz, 2H), 7.47 – 7.41 (m, 2H), 7.57 (t, ^3J = 7.8 Hz, 2H), 7.63 (dd, ^3J = 7.7 Hz, ^4J = 1.7 Hz, 2H), 7.77 (dd, ^3J = 7.8 Hz, ^4J = 1.7 Hz, 2H). ^{13}C NMR (151 MHz, chloroform-d$_1$) δ 20.0 (CH_3), 117.4 (C_{quat}), 119.9 (CH), 122.2 (CH), 122.6 (CH), 123.7 (CH), 125.7 (CH), 126.2 (CH), 127.5 (CH), 128.9 (CH), 129.2 (CH), 130.1 (CH), 131.0 (CH), 131.9 (CH), 133.3 (C_{quat}), 135.2 (C_{quat}), 135.6 (C_{quat}), 136.5 (CH), 136.9 (C_{quat}), 145.5 (C_{quat}), 145.9 (C_{quat}), 147.5 (C_{quat}), 159.5 (C_{quat}). EI MS (70 eV, *m/z* (%)) 723 (15), 722 (56), 721 ([M]$^+$, 100), 720 (13), 361 (23), 360 ([$C_{25}H_{18}N_3$]$^+$, 66), 359 (22). IR ($\tilde{\nu}$ [cm^{-1}]) 3061 (w), 2955 (w), 2926 (w), 2864 (w), 2359 (w), 1591 (w), 1586 (w), 1572 (w), 1559 (w), 1483 (m), 1451 (w), 1437 (w), 1418 (w), 1397 (w), 1308 (w), 1260 (w), 1204 (w), 1159 (w), 1115 (w), 1082 (w), 1072 (w), 1032 (w), 1005 (w), 988 (w), 870 (w), 814 (w), 754 (m), 739 (w), 694 (m), 642 (w), 629 (w). HRMS (ESI) calcd. for [$C_{50}H_{36}N_6$+H]$^+$: 721.3074; Found 721.3055.

3.4 Synthesis of the *ortho*-triarylamine dimer (*sym*-*o*-bTAA)$_2$ 7a via BLEBS Sequence for structural proof

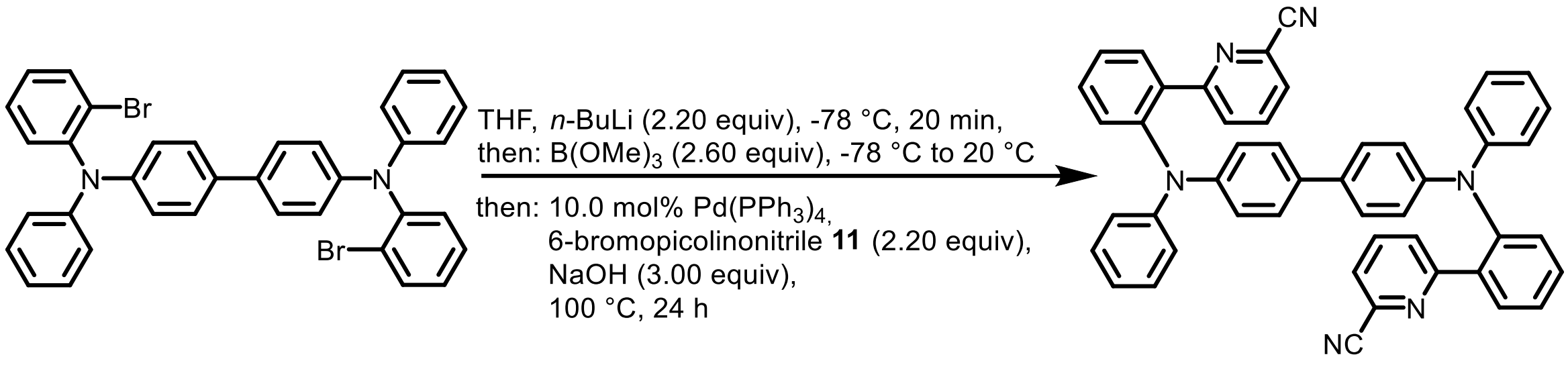

10a **7a**

Under nitrogen in a dried Schlenk tube *sym*-*o*-bTAA-dimer **10a** (323 mg, 0.50 mmol, 1.00 equiv) was dissolved in dry THF (3.6 ml) and cooled to −78 °C (dry ice/acetone bath). Then, *n*-butyllithium solution in hexanes (0.78 ml, 1.10 mmol, 2.20 equiv, 1.39 M) was added dropwise and the solution was stirred for 20 min at –78 °C. Afterwards, trimethylborate (0.16 ml, 1.30 mmol, 2.60 equiv) was added and the solution was warmed to 20 °C. Then, $Pd(PPh_3)_4$ (57.8 mg, 0.05 mmol, 10.0 mol%), the acceptor **11** (201 mg, 1.10 mmol, 2.20 equiv) and sodium hydroxide (60.0 mg, 1.50 mmol, 3.00 equiv) was added to the reaction mixture. The solution was stirred at 100 °C (oil bath) for 24 h. After cooling to room temperature, the reaction mixture was diluted with ethyl acetate and purified by flash chromatography on silica gel (*n*-hexane/ethyl acetate 7:1). After recrystallisation from ethanol and then drying under high vacuum compound **7a** (96.0 mg, 0.14 mmol, 28%) was obtained as yellow crystals.

4 ^{1}H and ^{13}C spectra of *meta*/*ortho*/*para*-triarylamine dimers (*sym-m*/*o*/*p*-bTAA)$_2$ 4, 5, 6, and 7

4.1 ^{1}H and ^{13}C spectra of *meta*/*ortho*/*para*-triarylamine dimers (*sym-m*/*o*/*p*-bTAA)$_2$ 4, 5, 6, and 7

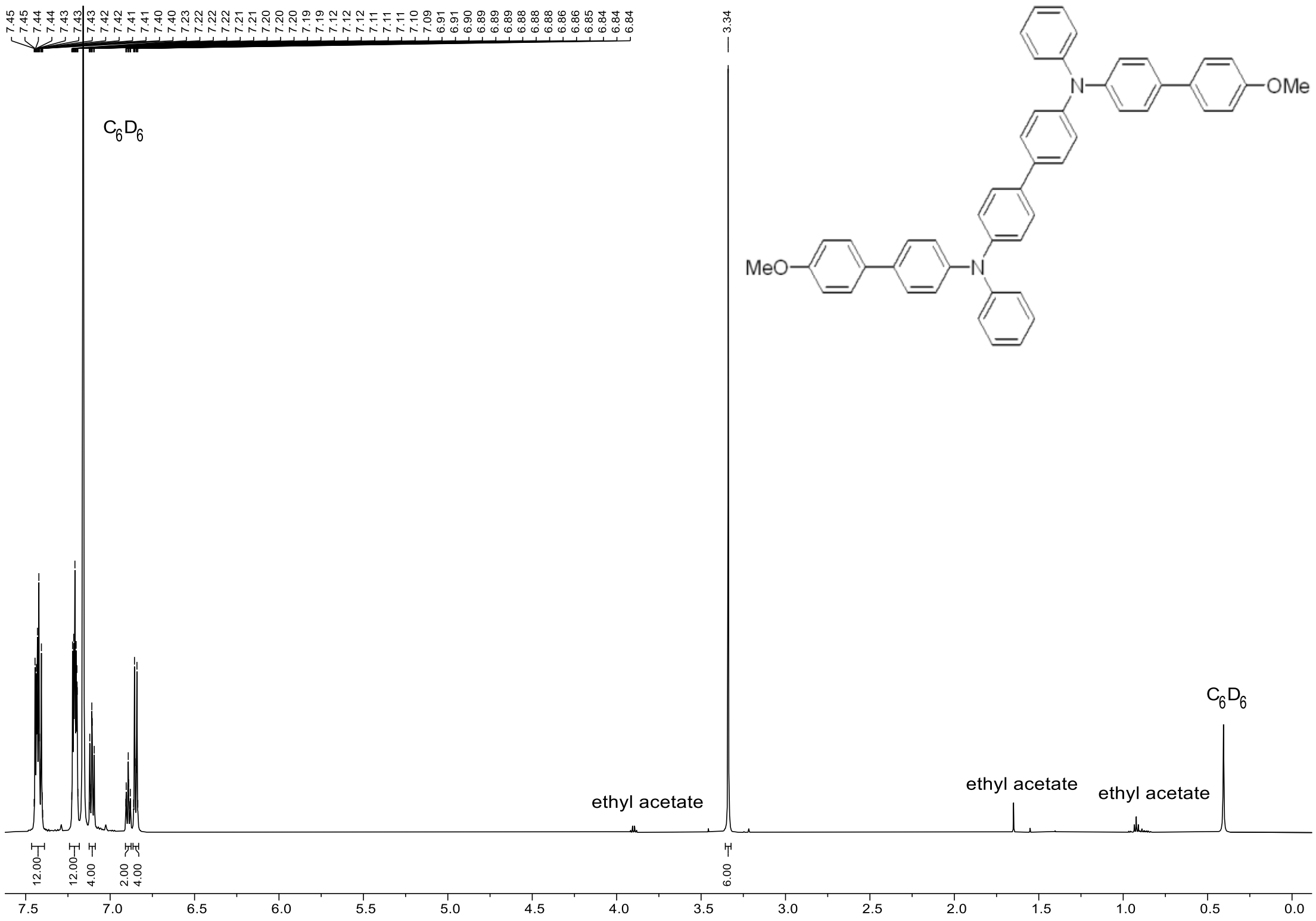

Figure S1. ^{1}H NMR spectrum (benzene-d_6, 600 MHz, 298 K) of compound **4a**.

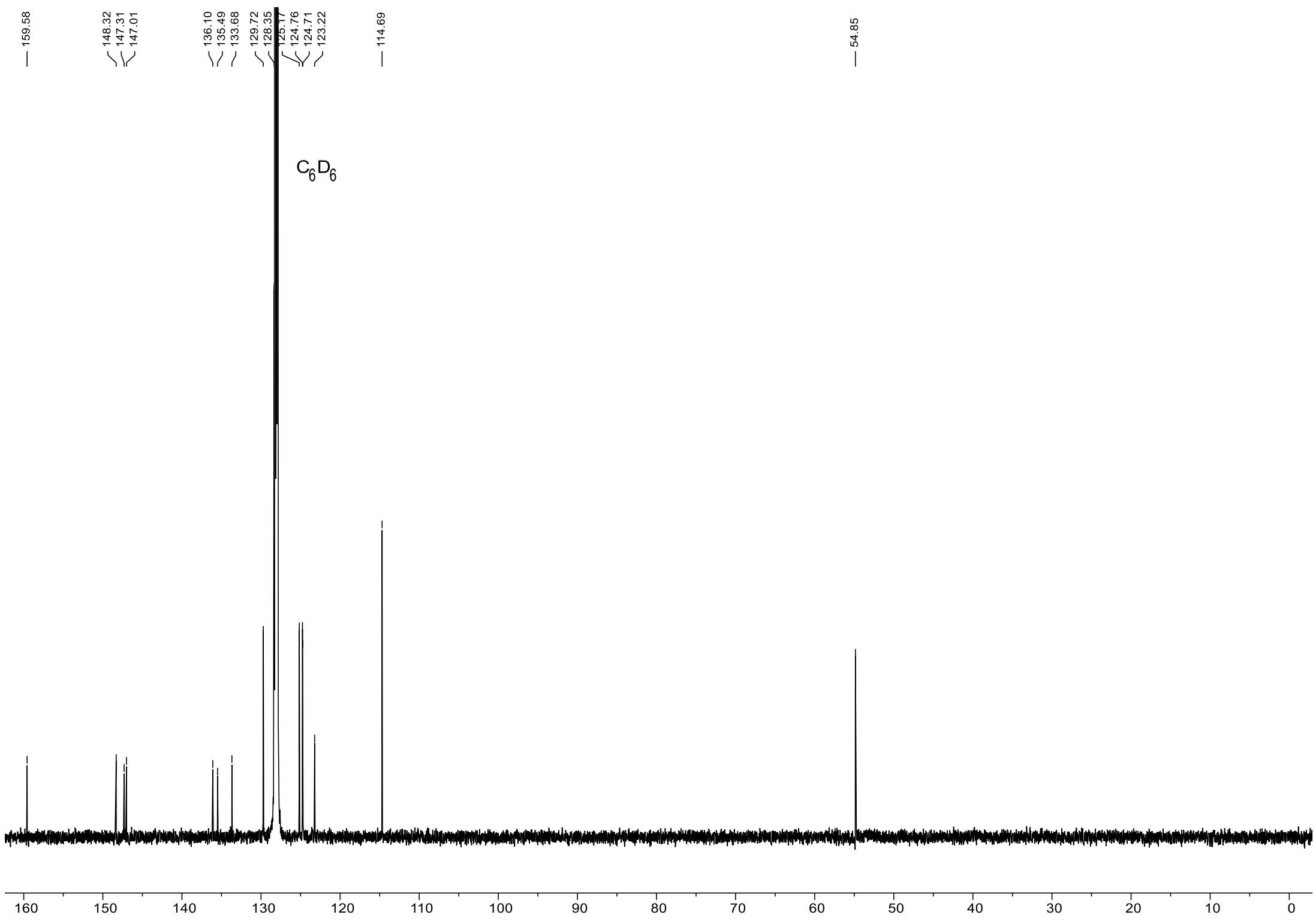

Figure S2. ^{13}C NMR spectrum (benzene-d_6, 150 MHz, 298 K) of compound **4a**.

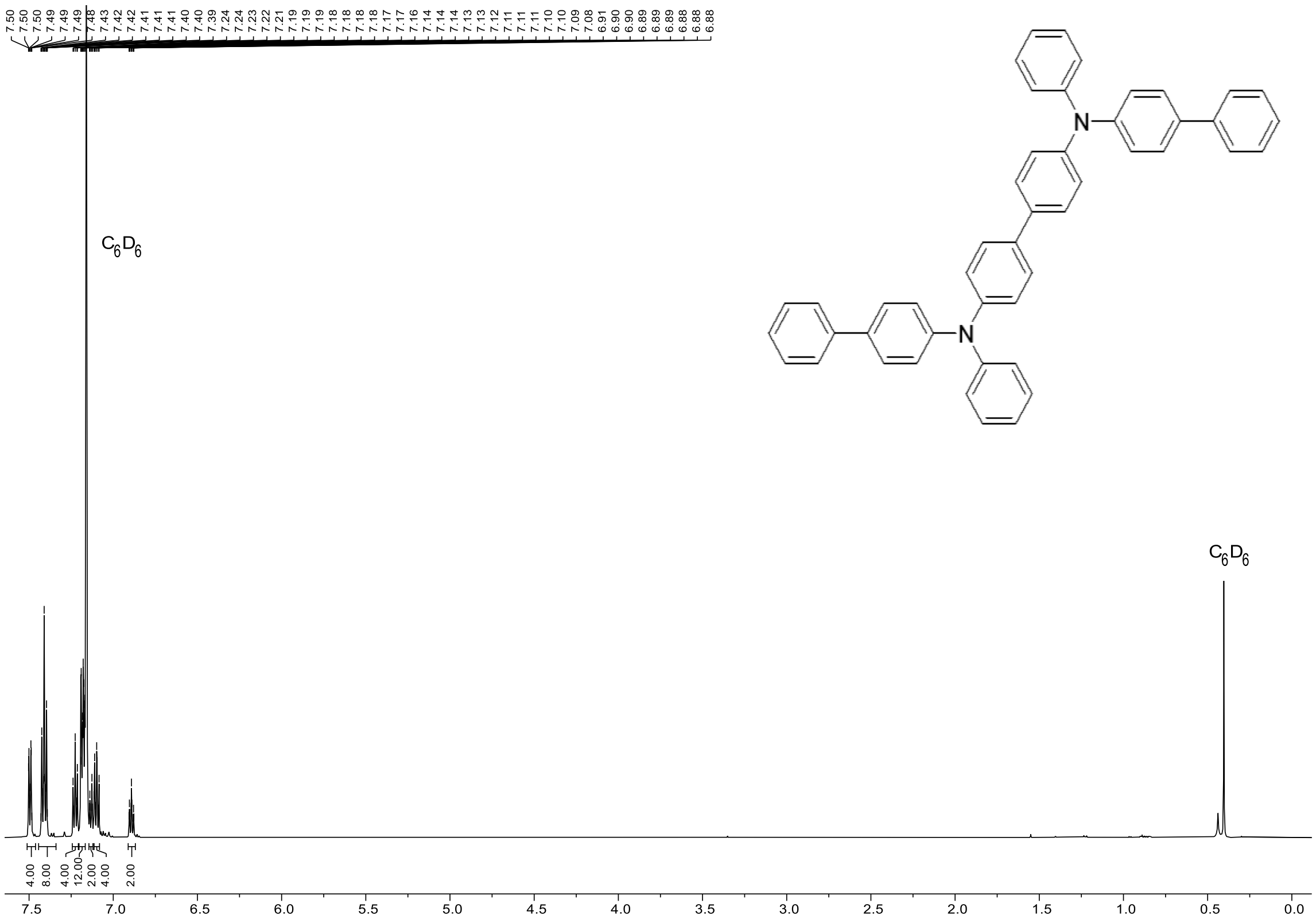

Figure S3. ^{1}H NMR spectrum (benzene-d_6, 600 MHz, 298 K) of compound **4b**.

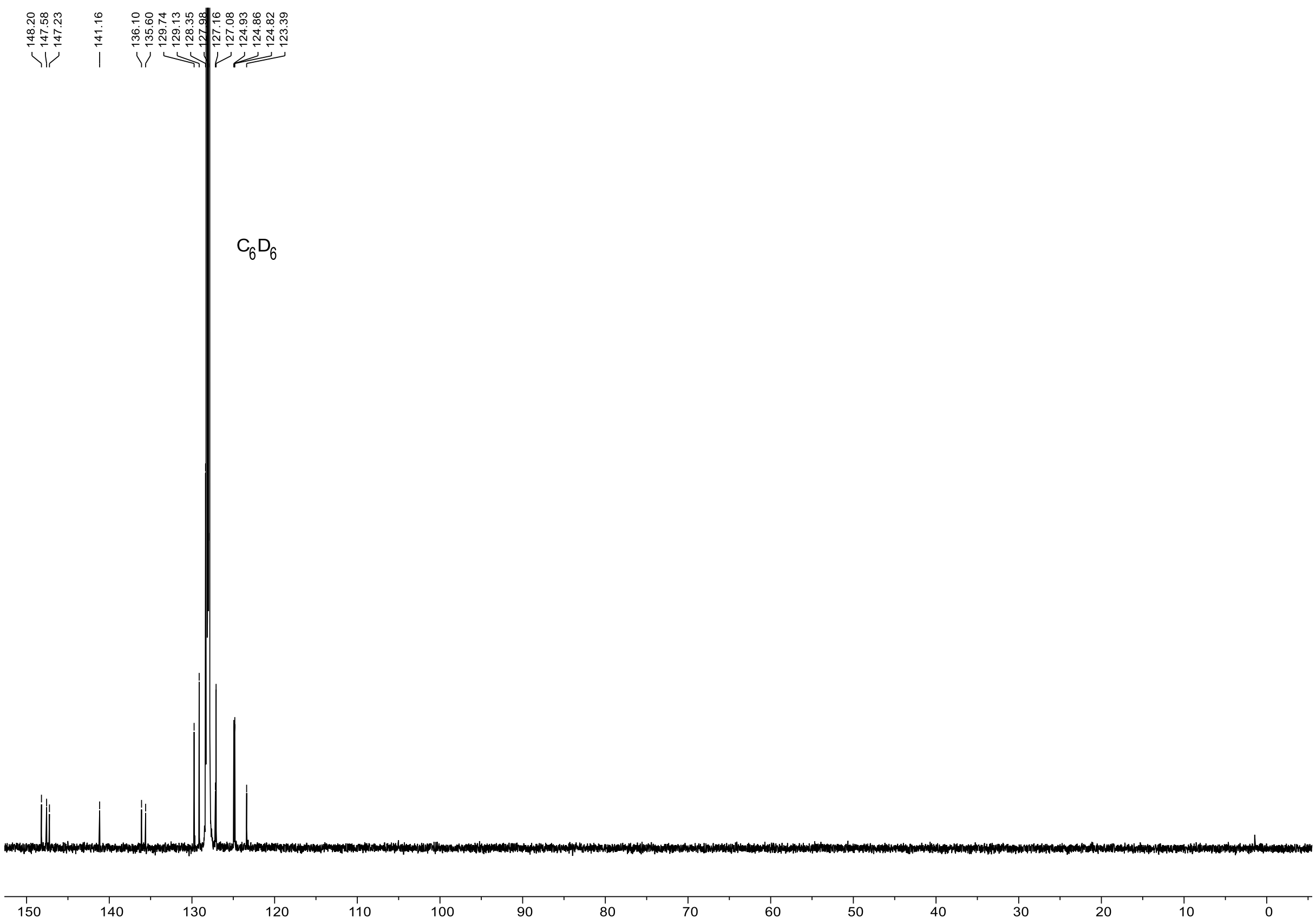

Figure S4. ^{13}C NMR spectrum (benzene-d_6, 150 MHz, 298 K) of compound **4b**.

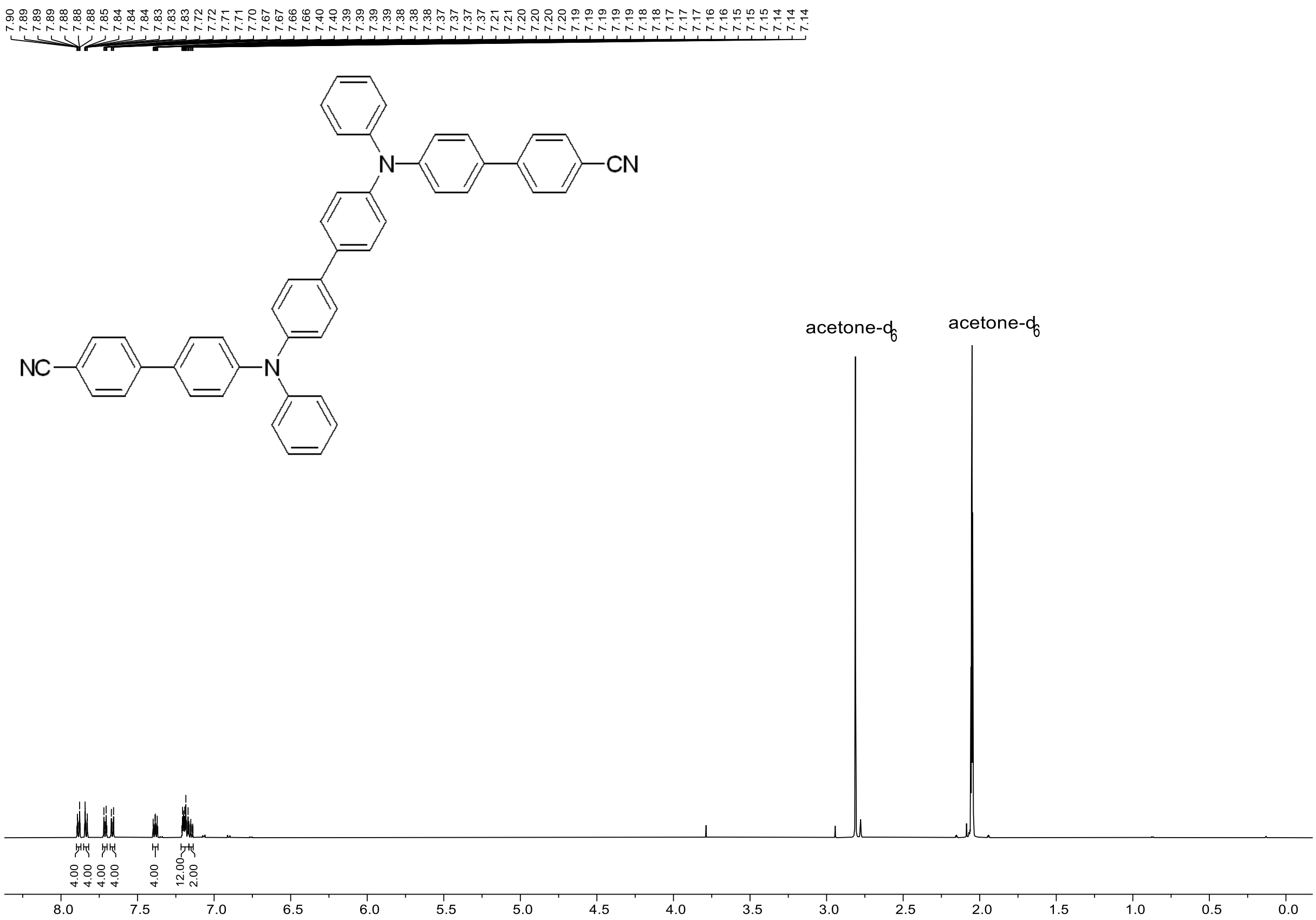

Figure S5. ^{1}H NMR spectrum (acetone-d_6, 600 MHz, 298 K) of compound **4c**.

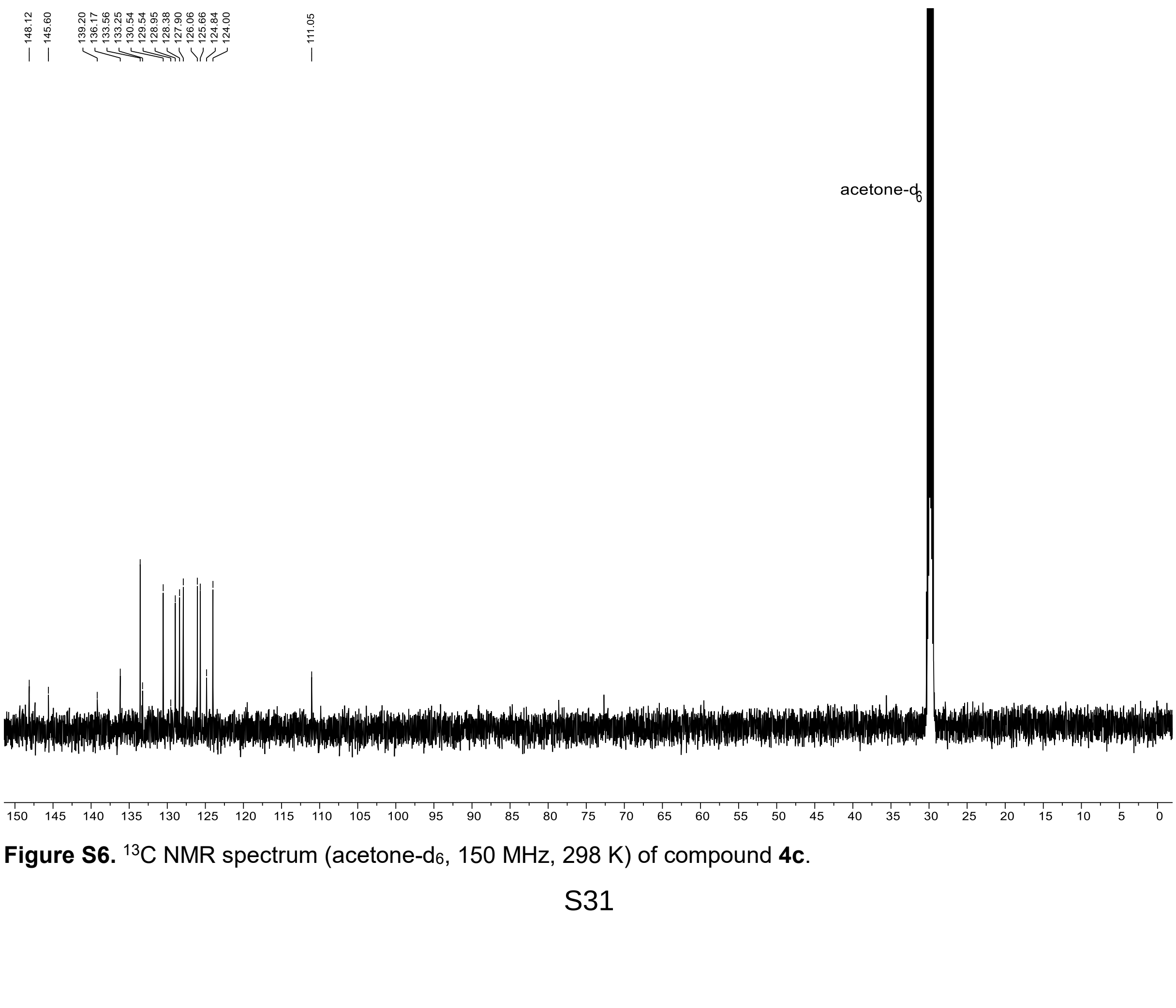

Figure S6. ^{13}C NMR spectrum (acetone-d_6, 150 MHz, 298 K) of compound **4c**.

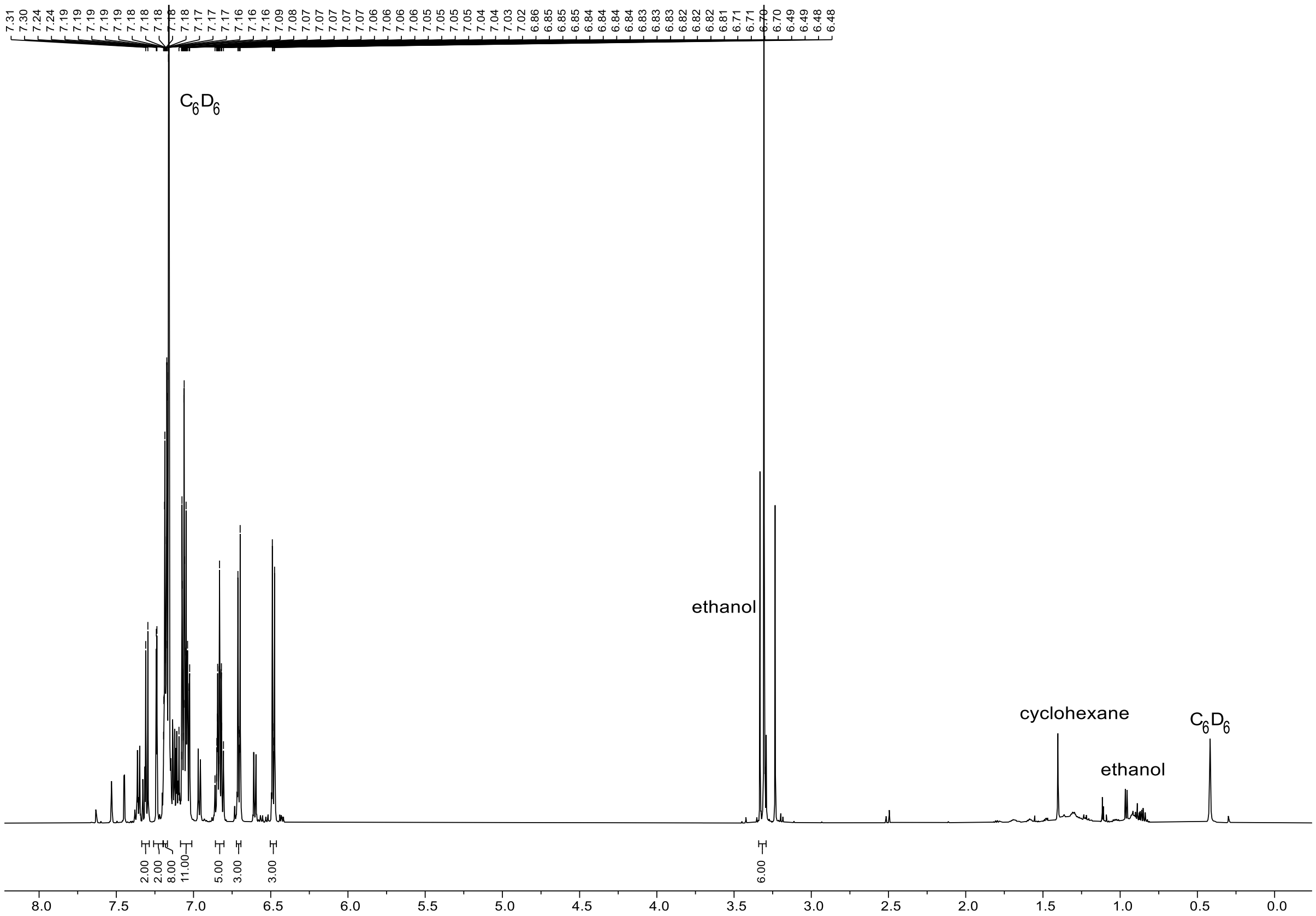

Figure S7. ^{1}H NMR spectrum (benzene-d$_6$, 600 MHz, 298 K) of compound mixture **5a/5a'/5a''**.

Table S3. Expected characteristic signals in the ^{1}H NMR spectrum of compound **5a/5a'/5a''**.

ratio: 10 : 5 : 1

5a (ratio: 10)

proton	signal	coupling	integral	?
6x ^{1}H	s	-	6.00	✓
2x ^{8}H	d	^{3}J	2.00	✓
2x ^{9}H	dd	^{3}J, ^{4}J	2.00	✓
2x ^{11}H	d	^{4}J	2.00	✓
4x ^{15}H	tt	^{3}J, ^{4}J	4.00	X

5a' (ratio: 5)

proton	signal	coupling	integral	?
3x ^{1}H	s	-	1.50	✓
3x ^{1}H	s	-	1.50	✓
1x ^{7}H	ddd	^{3}J,^{4}J,^{4}J	0.50	X
1x ^{8}H	dd	^{3}J, ^{3}J	0.50	X
1x ^{9}H	ddd	^{3}J,^{4}J,^{4}J	0.50	X
1x ^{11}H	dd	^{4}J, ^{4}J	0.50	✓
1x ^{15}H	tt	^{3}J, ^{4}J	0.50	X
1x ^{21}H	d	^{3}J	0.50	✓
1x ^{22}H	dd	^{3}J, ^{4}J	0.50	X
1x ^{24}H	d	^{4}J	0.50	
2x ^{34}H	tt	^{3}J, ^{4}J	1.00	X

5a'' (ratio: 1)

proton	signal	coupling	integral	?
6x ^{1}H	s	-	0.60	✓
2x ^{7}H	ddd	^{3}J, ^{4}J, ^{4}J	0.20	X
2x ^{8}H	dd	^{3}J, ^{3}J	0.20	X
2x ^{9}H	ddd	^{3}J, ^{4}J, ^{4}J	0.20	X
2x ^{11}H	dd	^{4}J, ^{4}J	0.20	X
2x ^{15}H	tt	^{3}J, ^{4}J	0.20	X

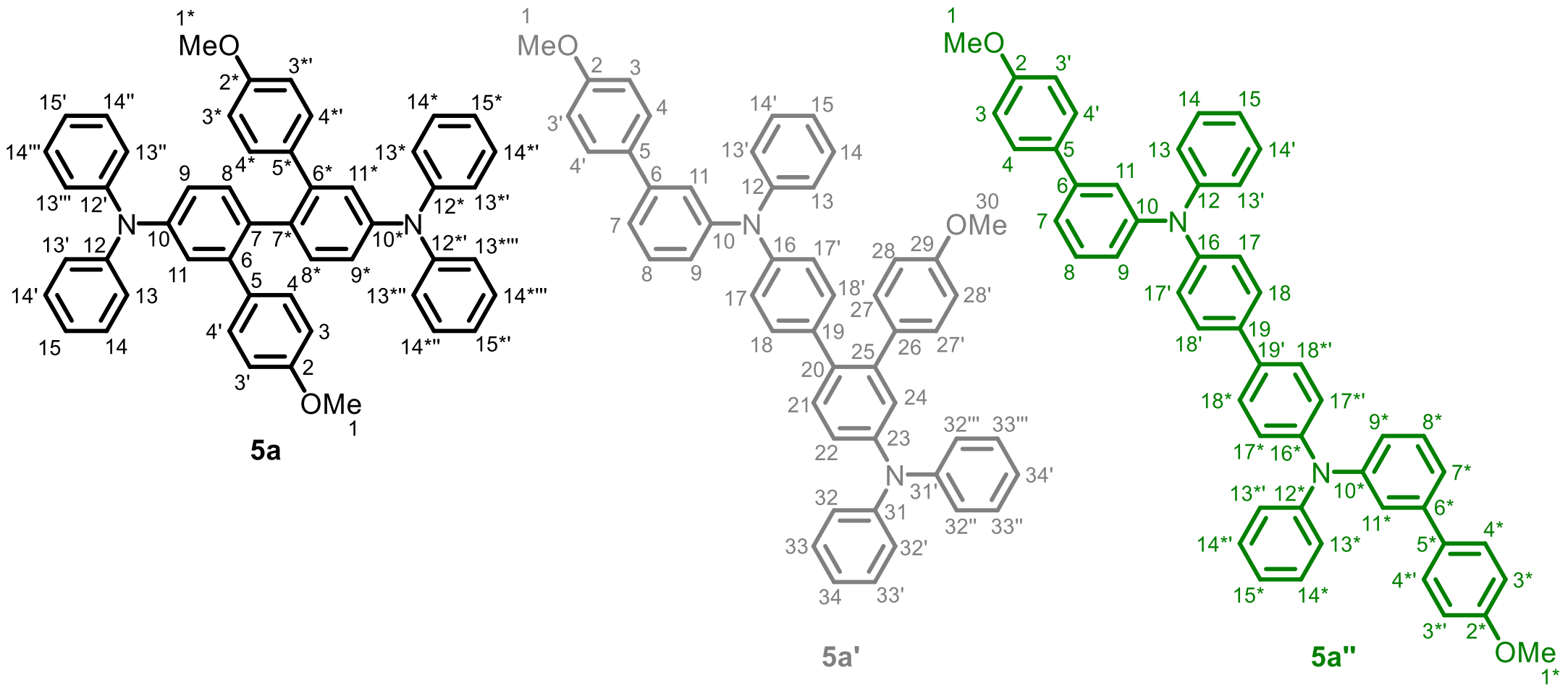

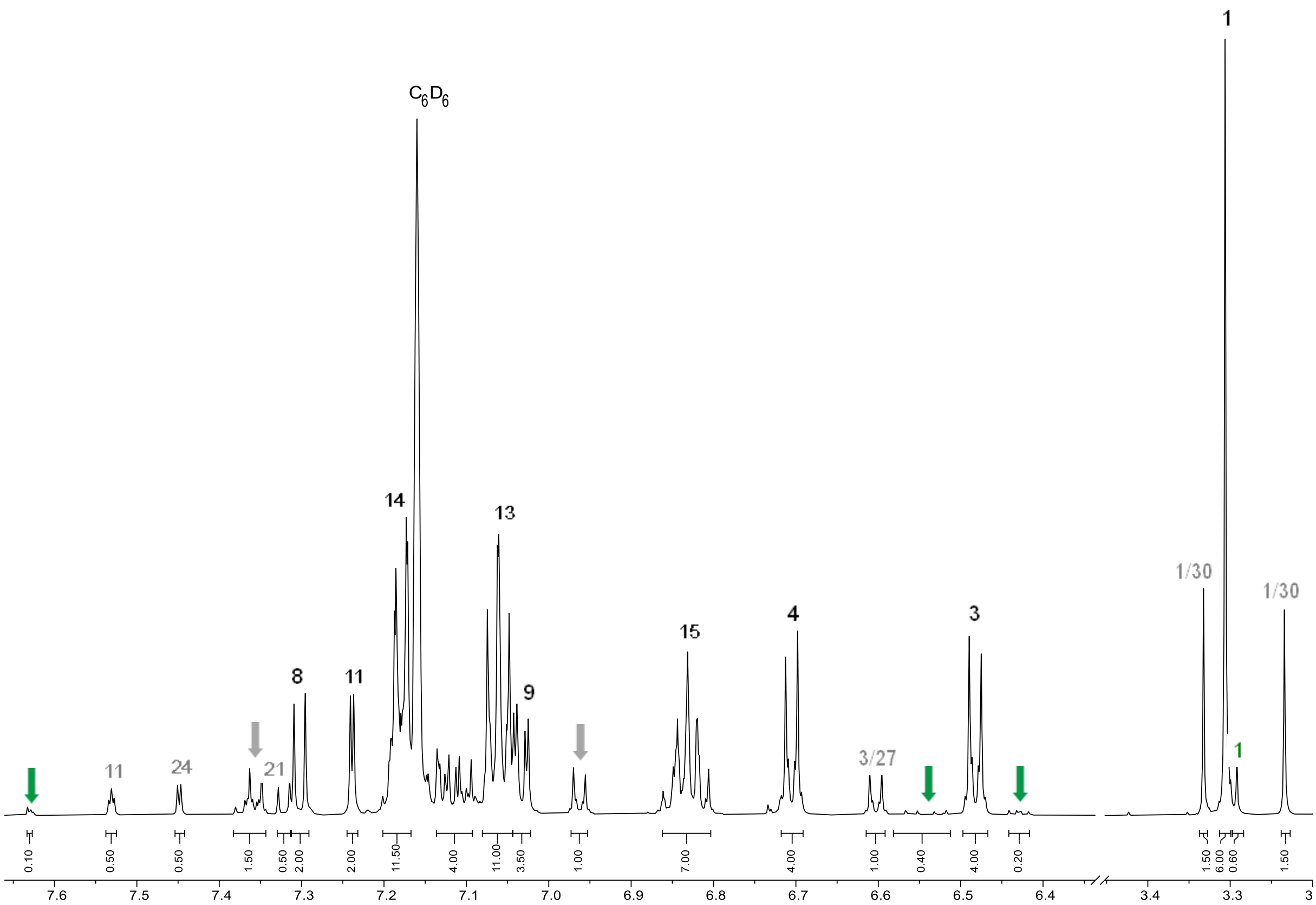

Figure S8. ^{1}H NMR spectrum of product mixture **5a** (black), **5a'** (gray), and **5a''** (green) (benzene-d$_6$, 600 MHz, 298 K).

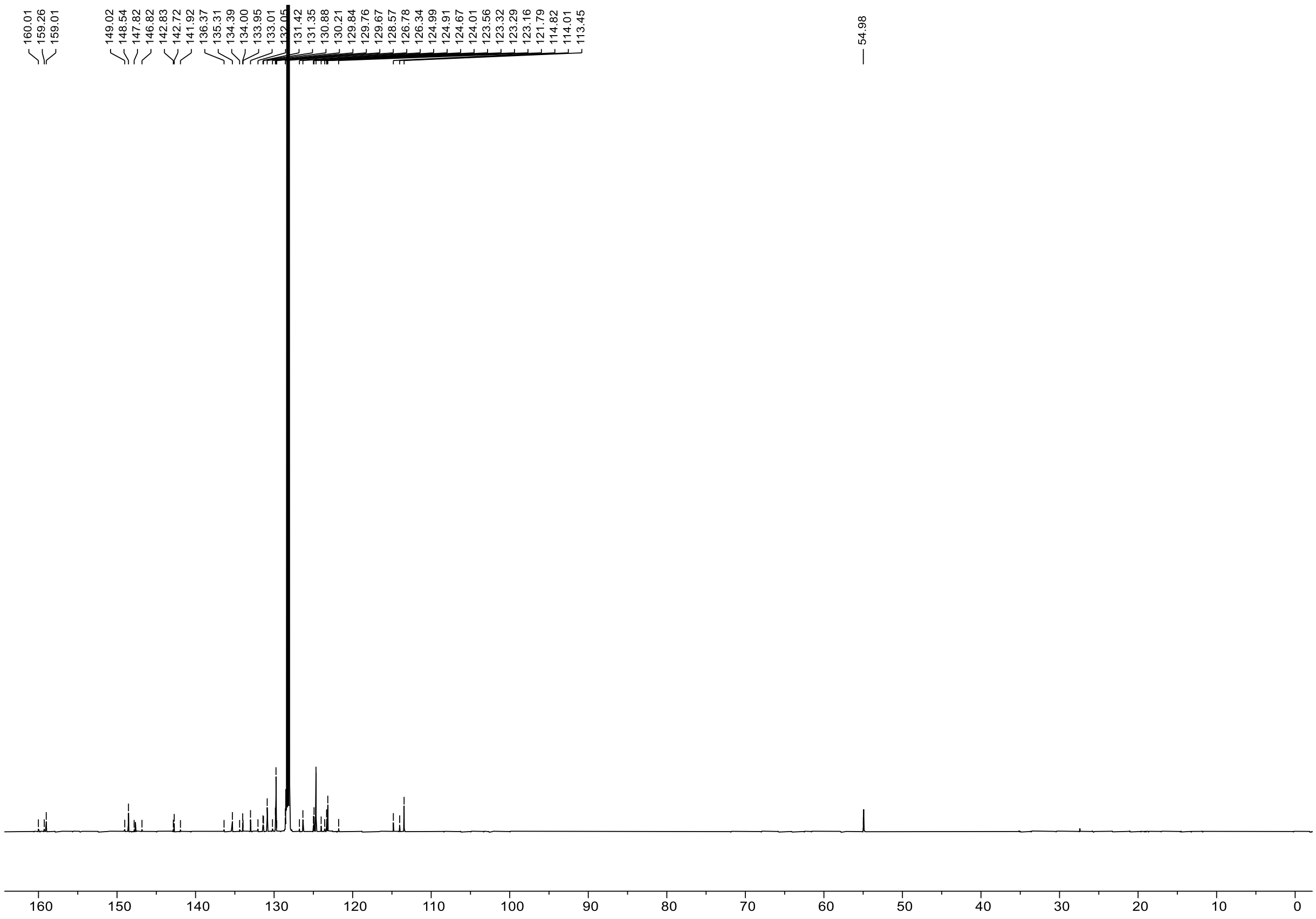

Figure S9. ^{13}C NMR spectrum (benzene-d_6, 150 MHz, 298 K) of compound mixture **5a/5a'/5a"**.

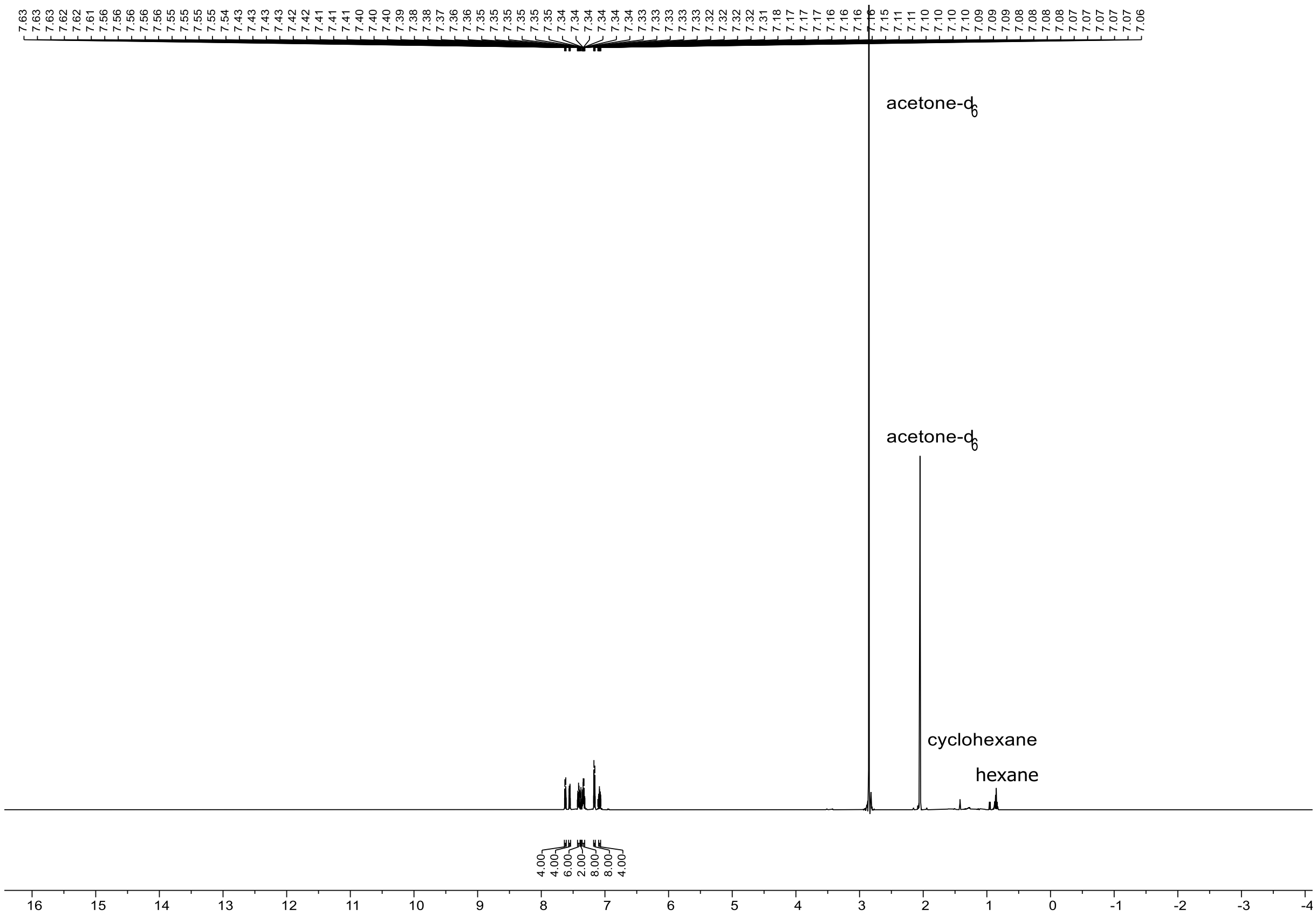

Figure S10. ^{1}H NMR spectrum (acetone-d_6, 600 MHz, 298 K) of compound mixture **5b/5b'/5b''**.

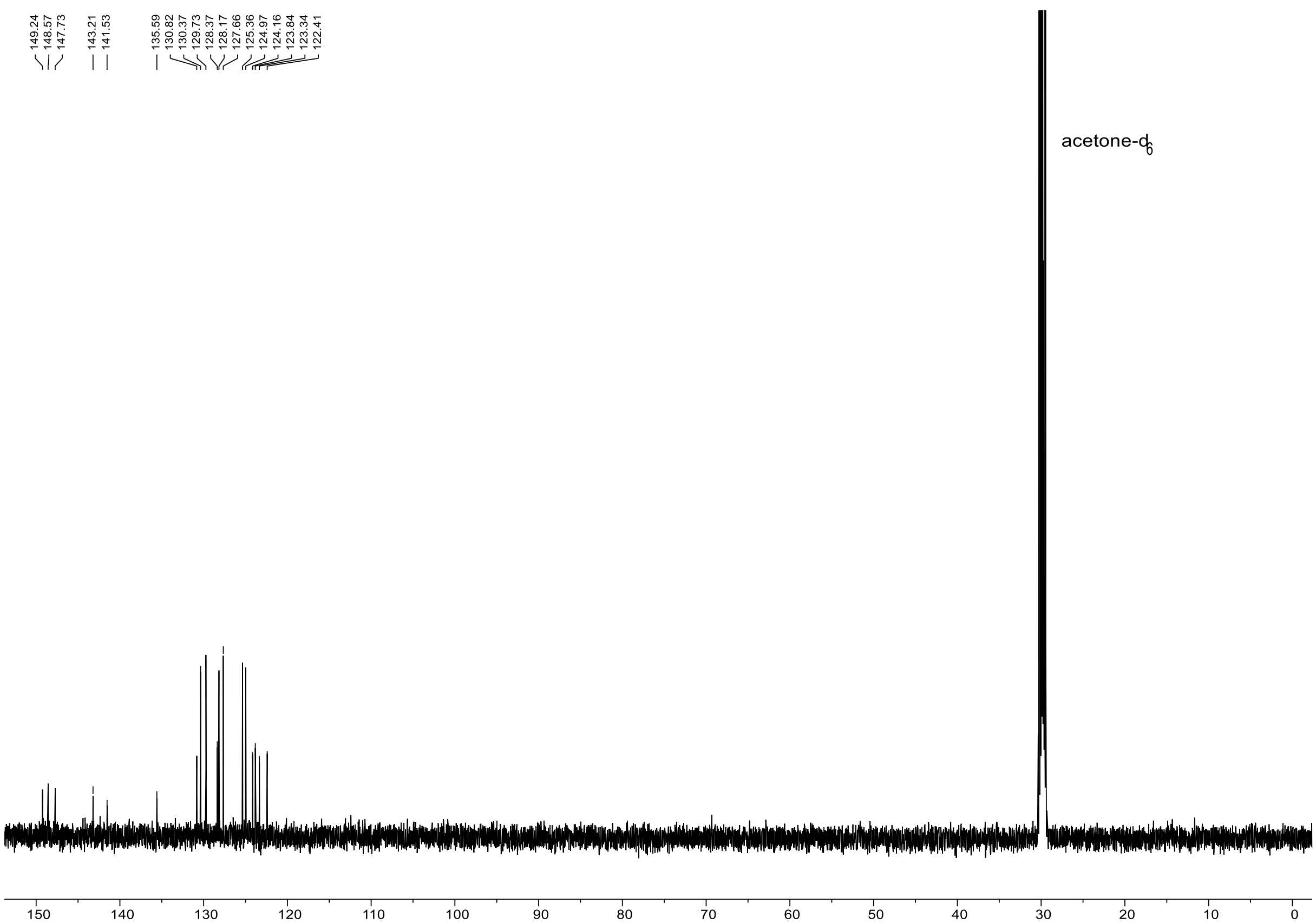

Figure S11. ^{13}C NMR spectrum (acetone-d_6, 150 MHz, 298 K) of compound mixture **5b/5b'/5b''**.

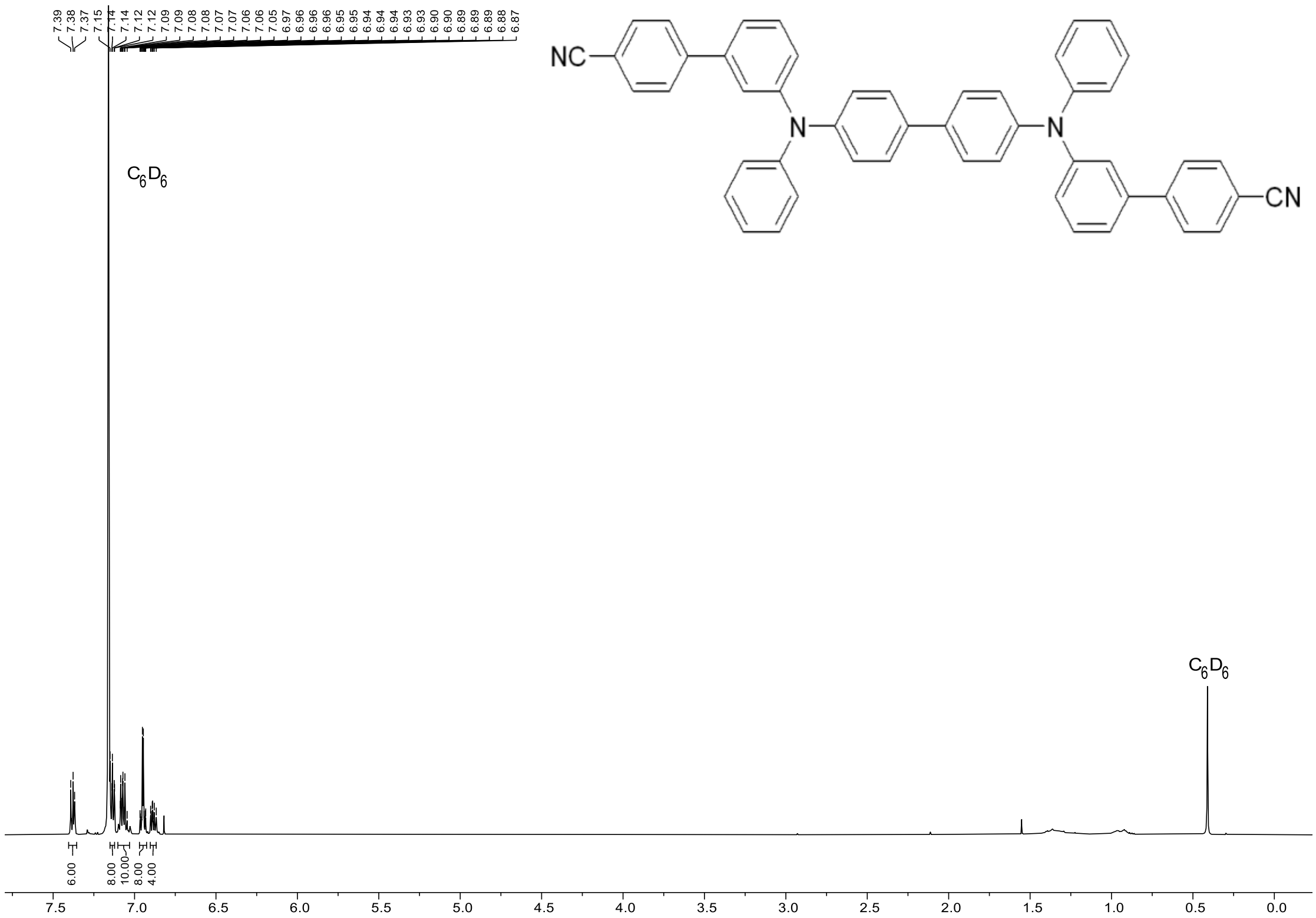

Figure S12. ^{1}H NMR spectrum (benzene-d_6, 600 MHz, 298 K) of compound **5c**.

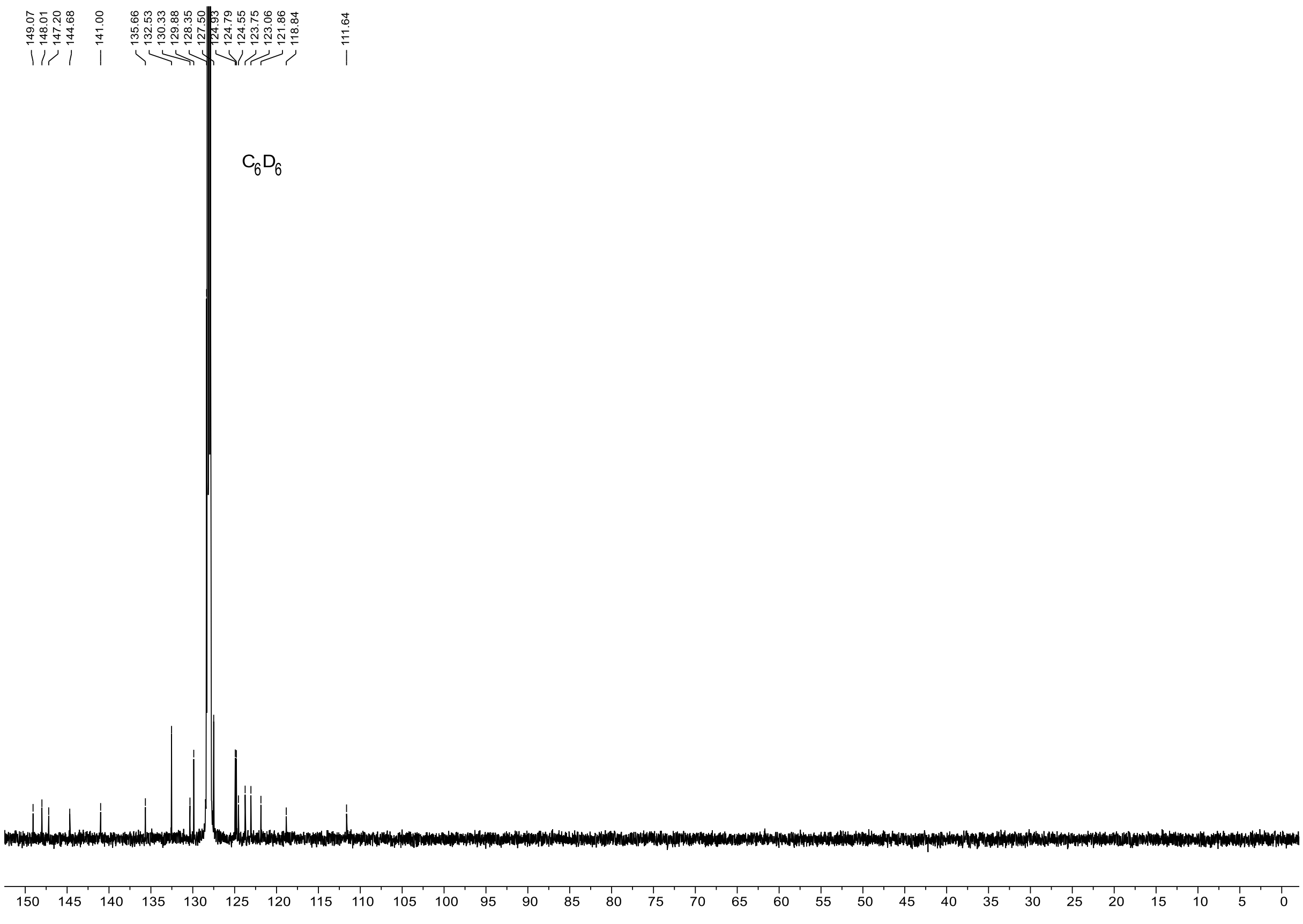

Figure S13. ^{13}C NMR spectrum (benzene-d_6, 150 MHz, 298 K) of compound **5c**.

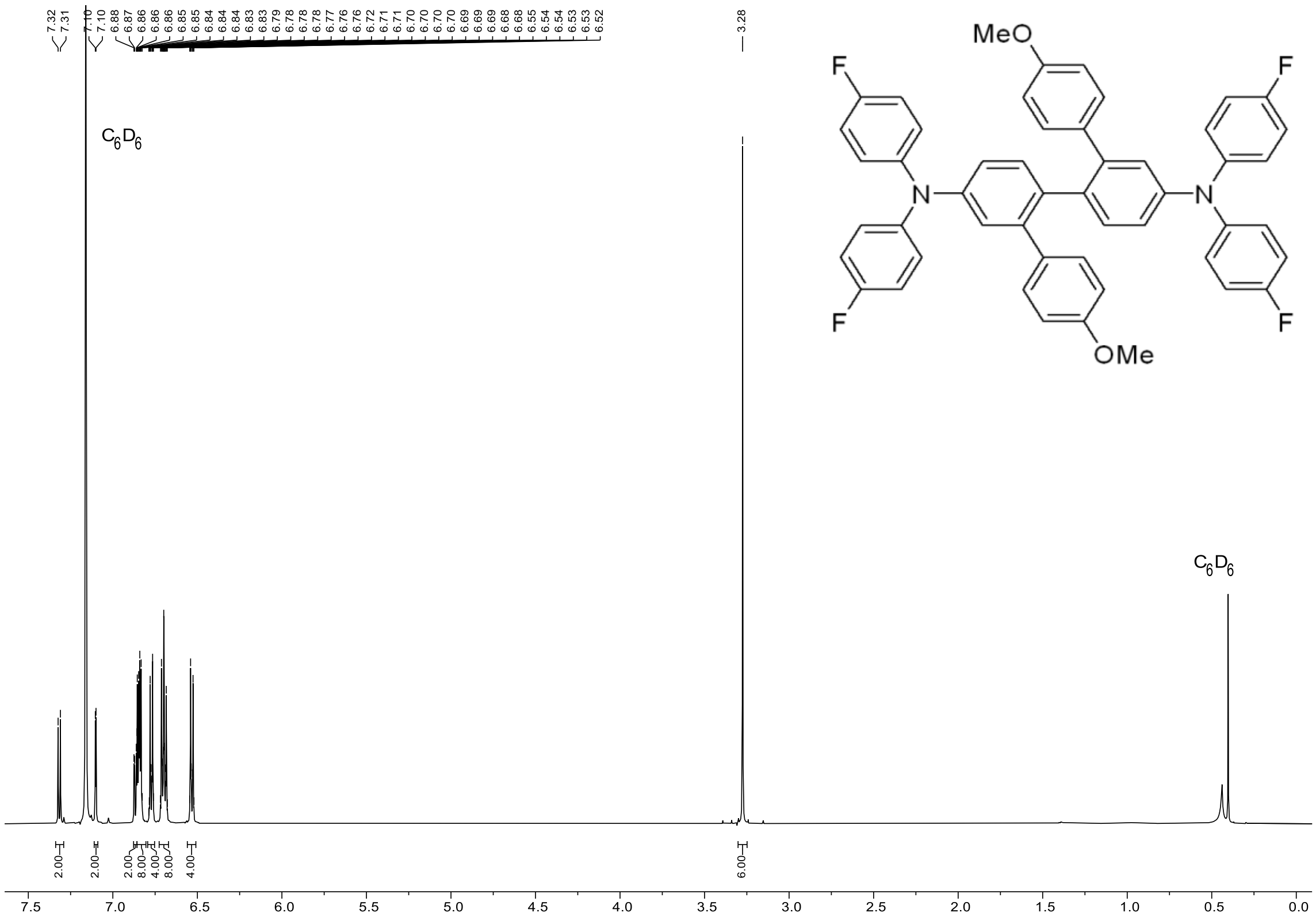

Figure S14. ^{1}H NMR spectrum (acetone-d$_6$, 600 MHz, 298 K) of compound **5d**.

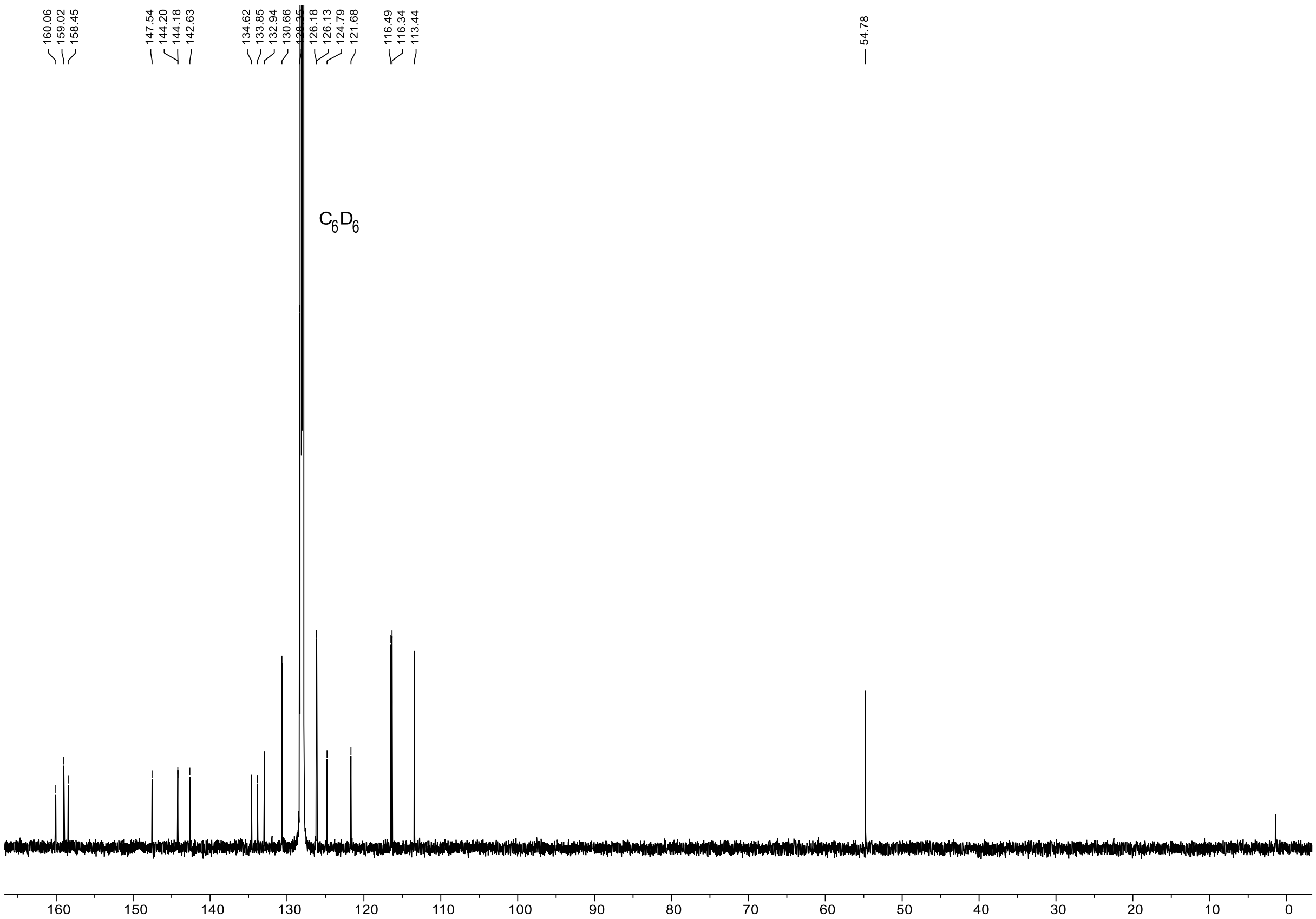

Figure S15. ^{13}C NMR spectrum (acetone-d$_6$, 150 MHz, 298 K) of compound **5d**.

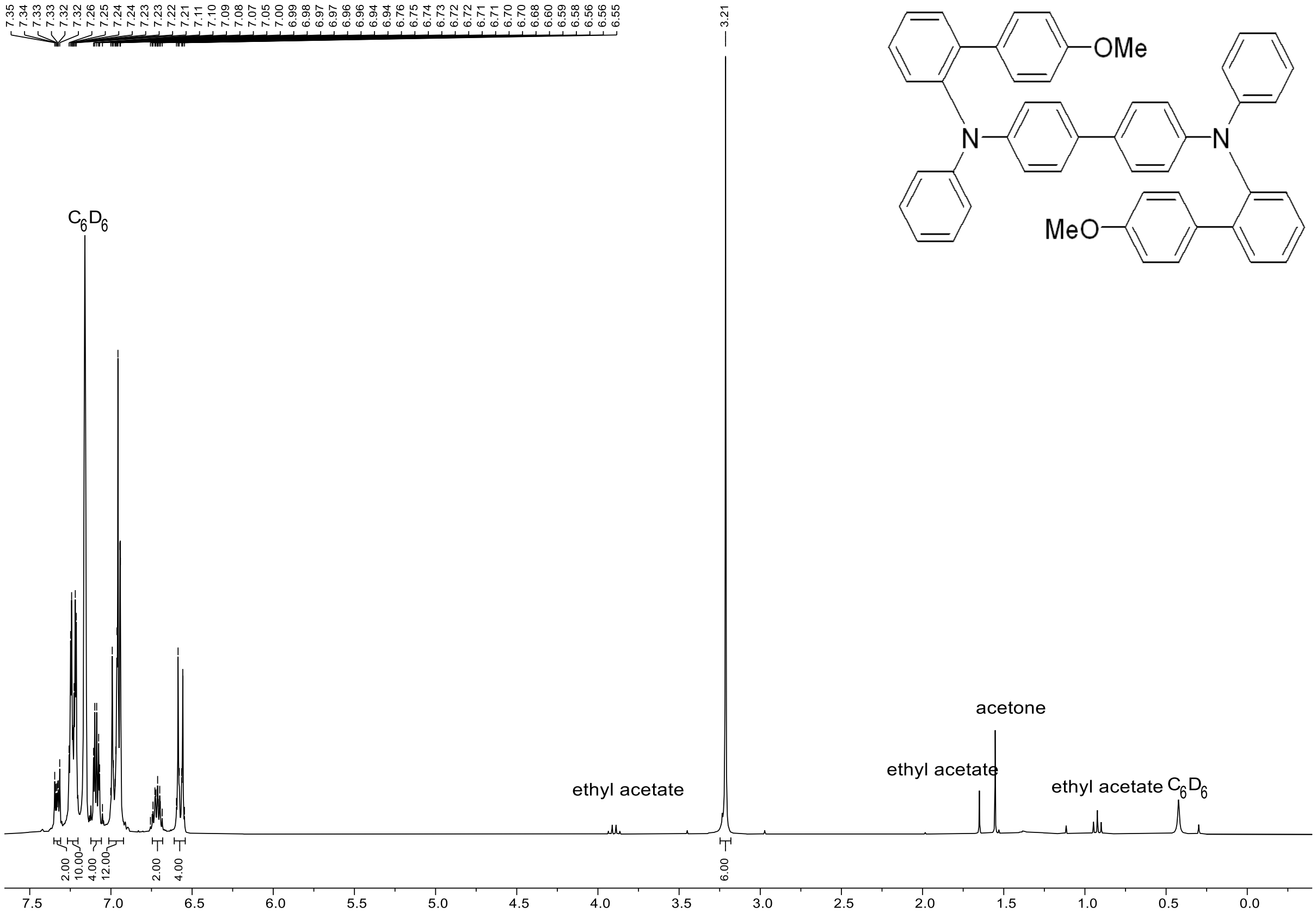

Figure S16. ^{1}H NMR spectrum (benzene-d_6, 600 MHz, 298 K) of compound **6a**.

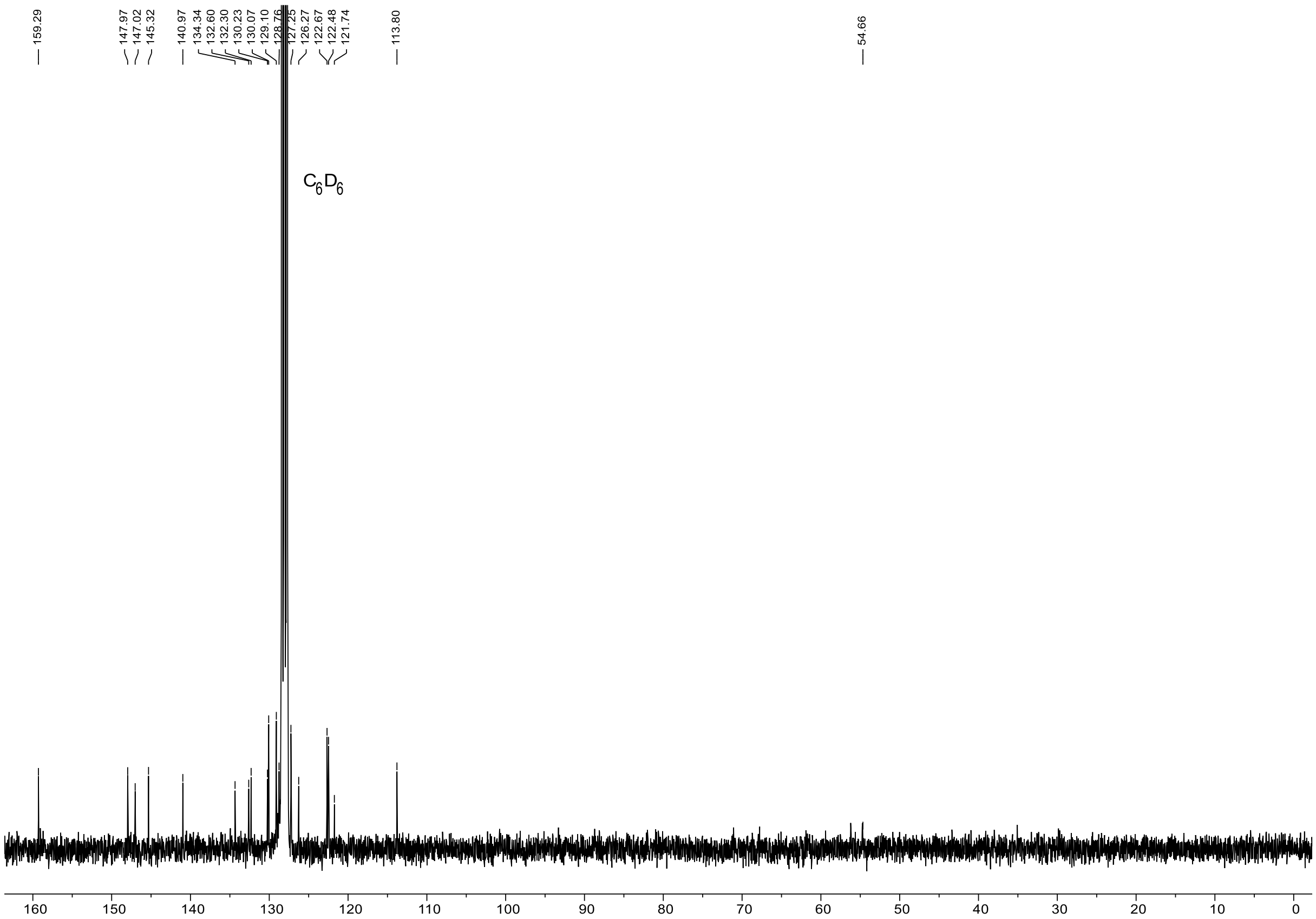

Figure S17. ^{13}C NMR spectrum (benzene-d_6, 150 MHz, 298 K) of compound **6a**.

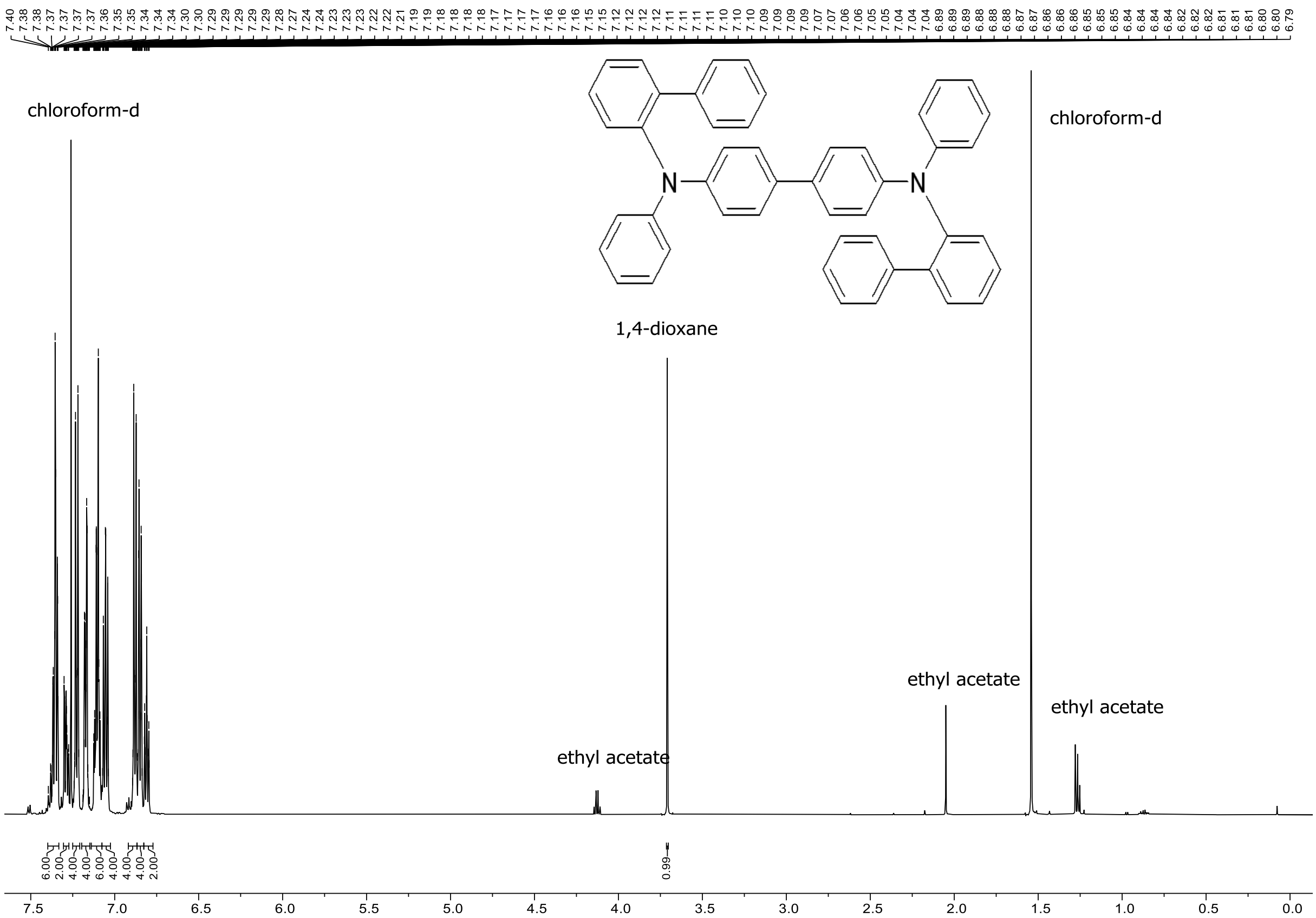

Figure S18. ^{1}H NMR spectrum (chloroform-d, 600 MHz, 298 K) of compound **6b**.

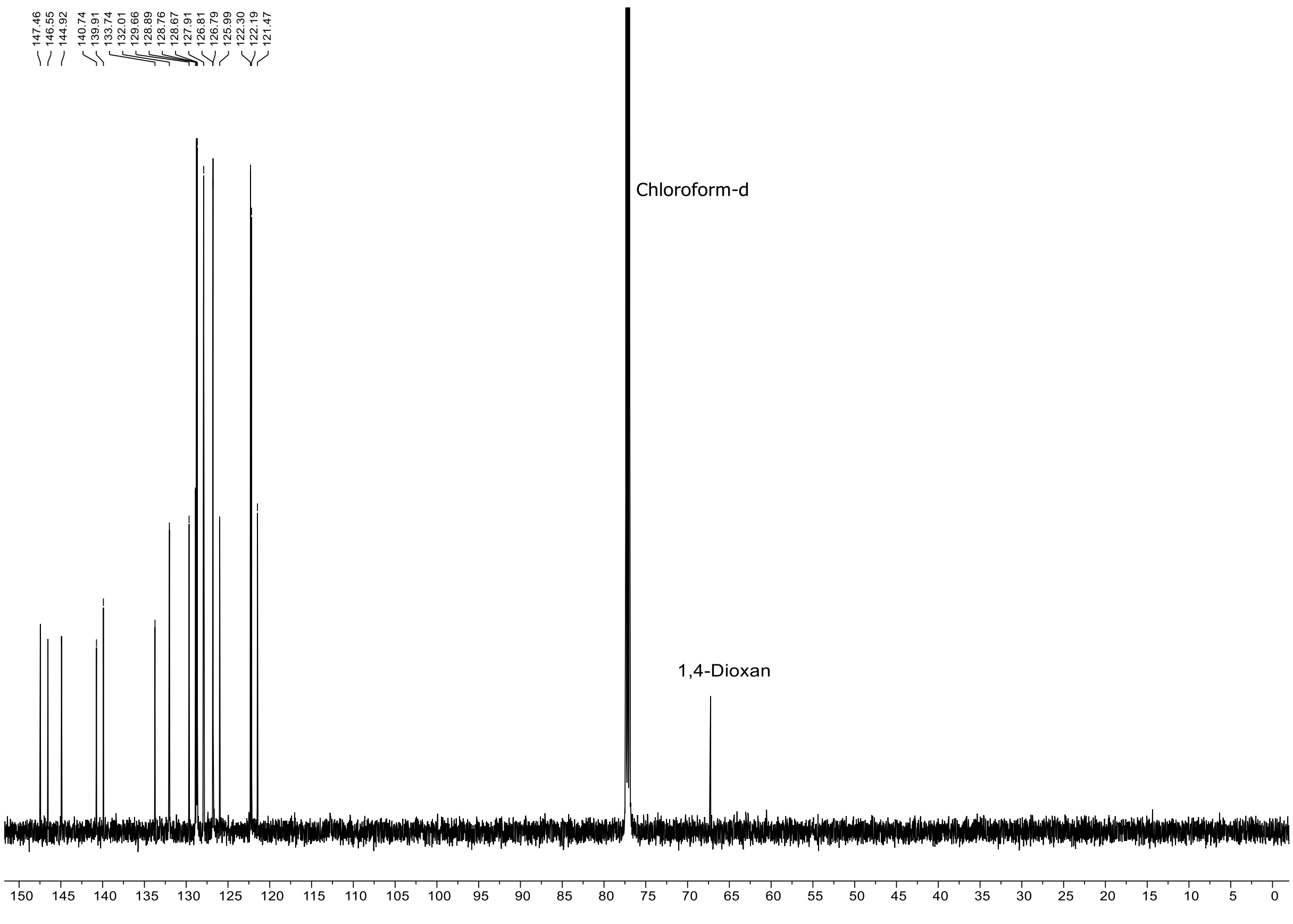

Figure S19. ^{13}C NMR spectrum (chloroform-d_1, 150 MHz, 298 K) of compound **6b**.

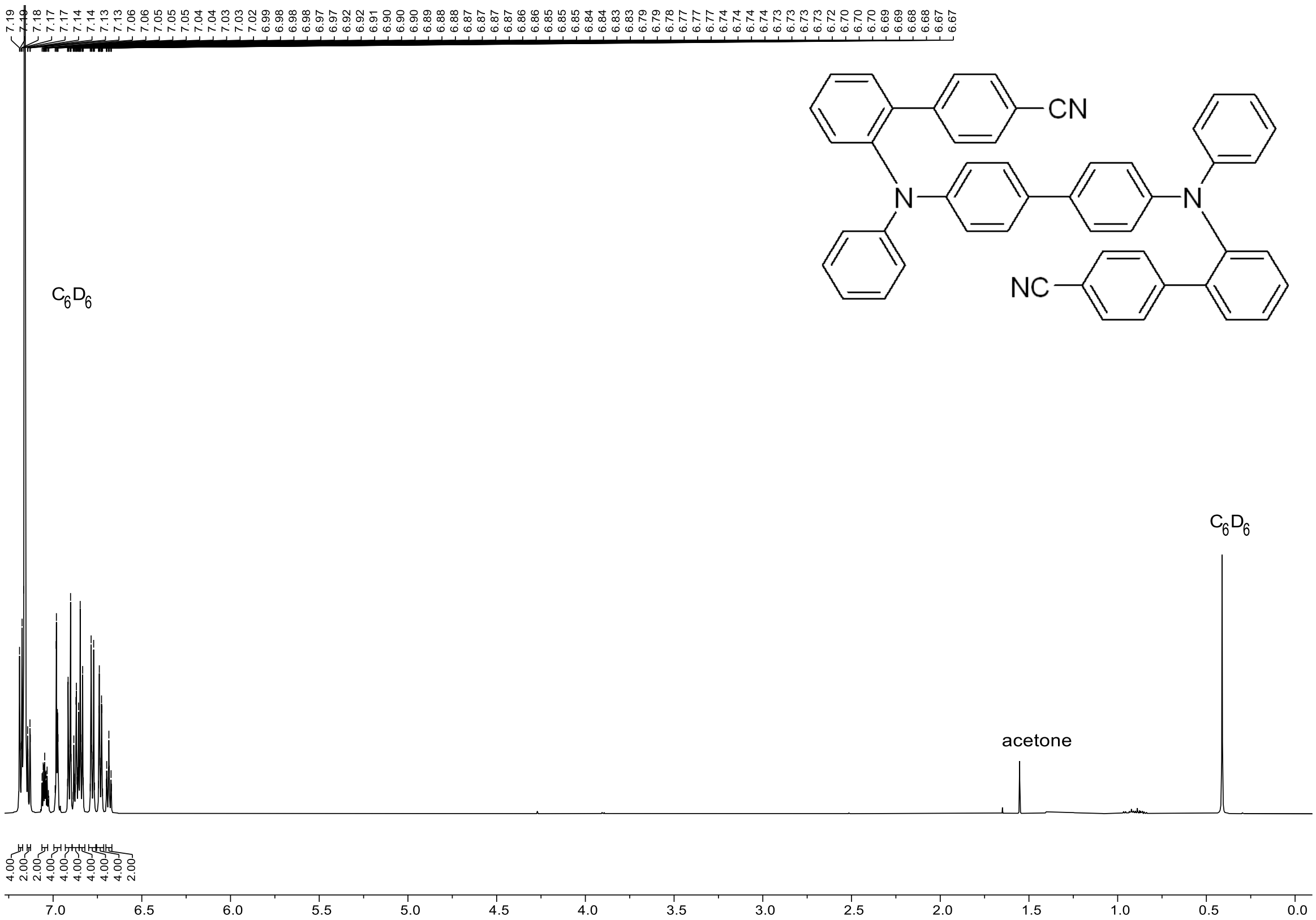

Figure S20. ^{1}H NMR spectrum (benzene-d_6, 600 MHz, 298 K) of compound **6c**.

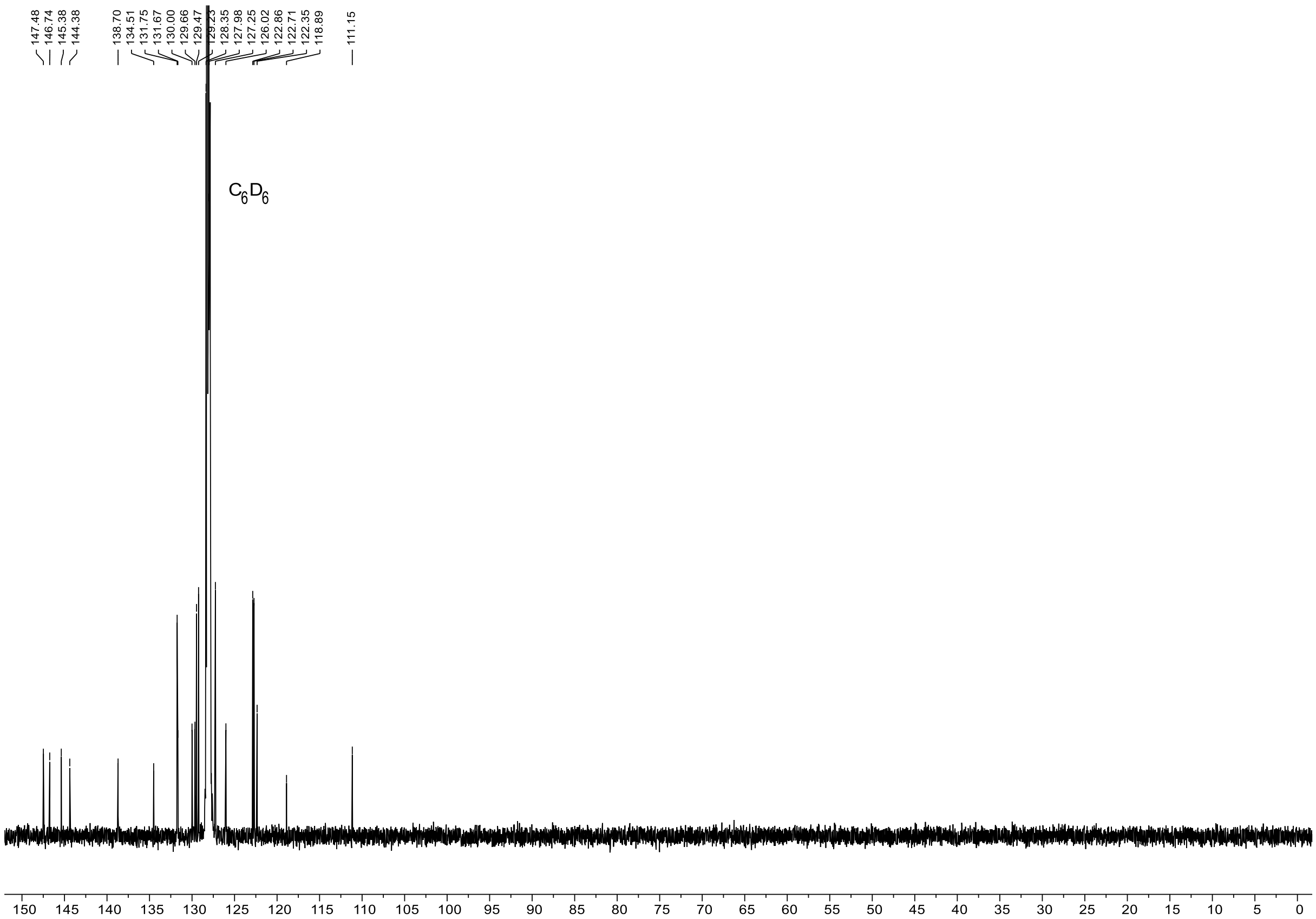

Figure S21. ^{13}C NMR spectrum (benzene-d_6, 150 MHz, 298 K) of compound **6c**.

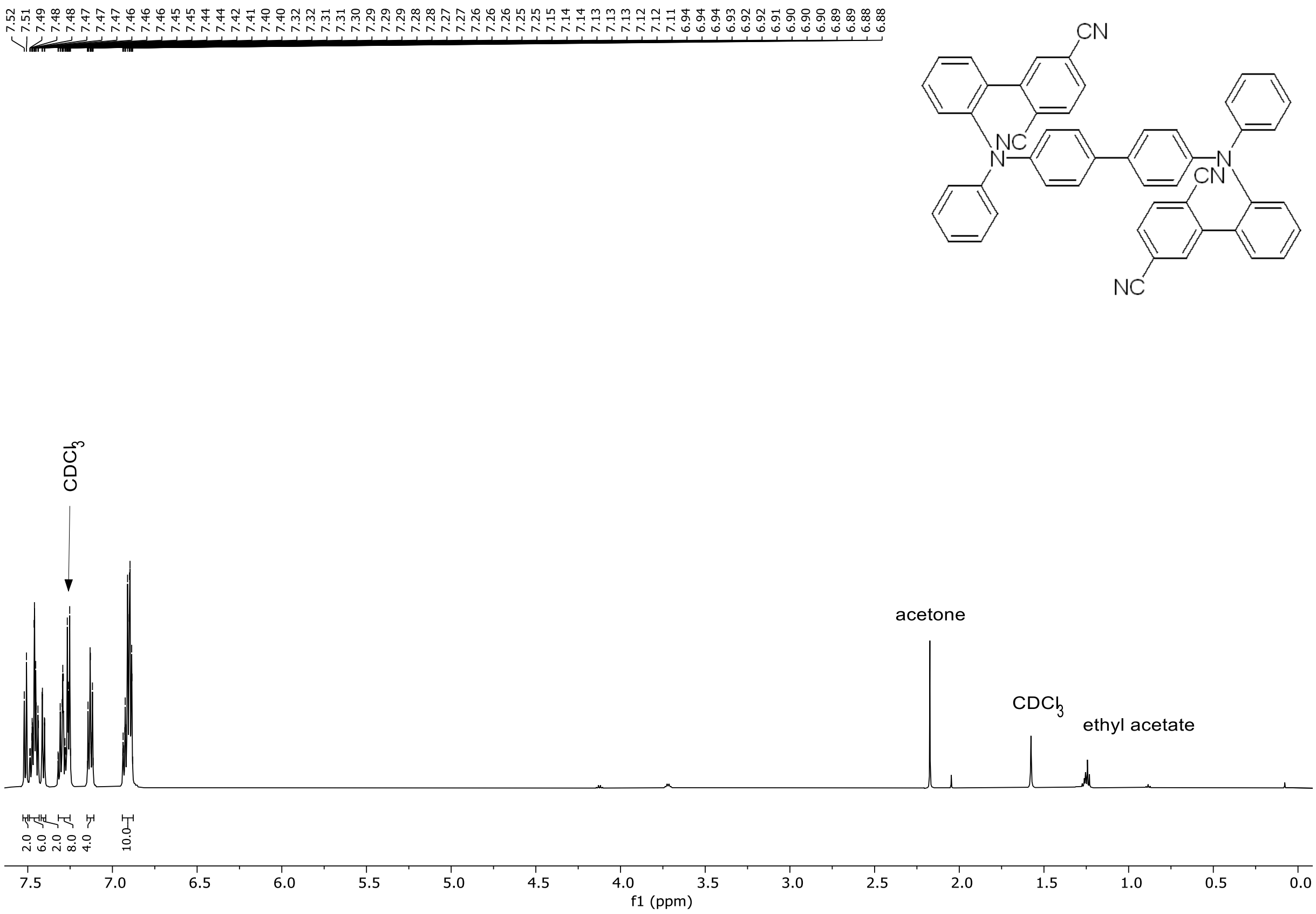

Figure S22. ^{1}H NMR spectrum (chloroform-d$_1$, 600 MHz, 298 K) of compound **6d**.

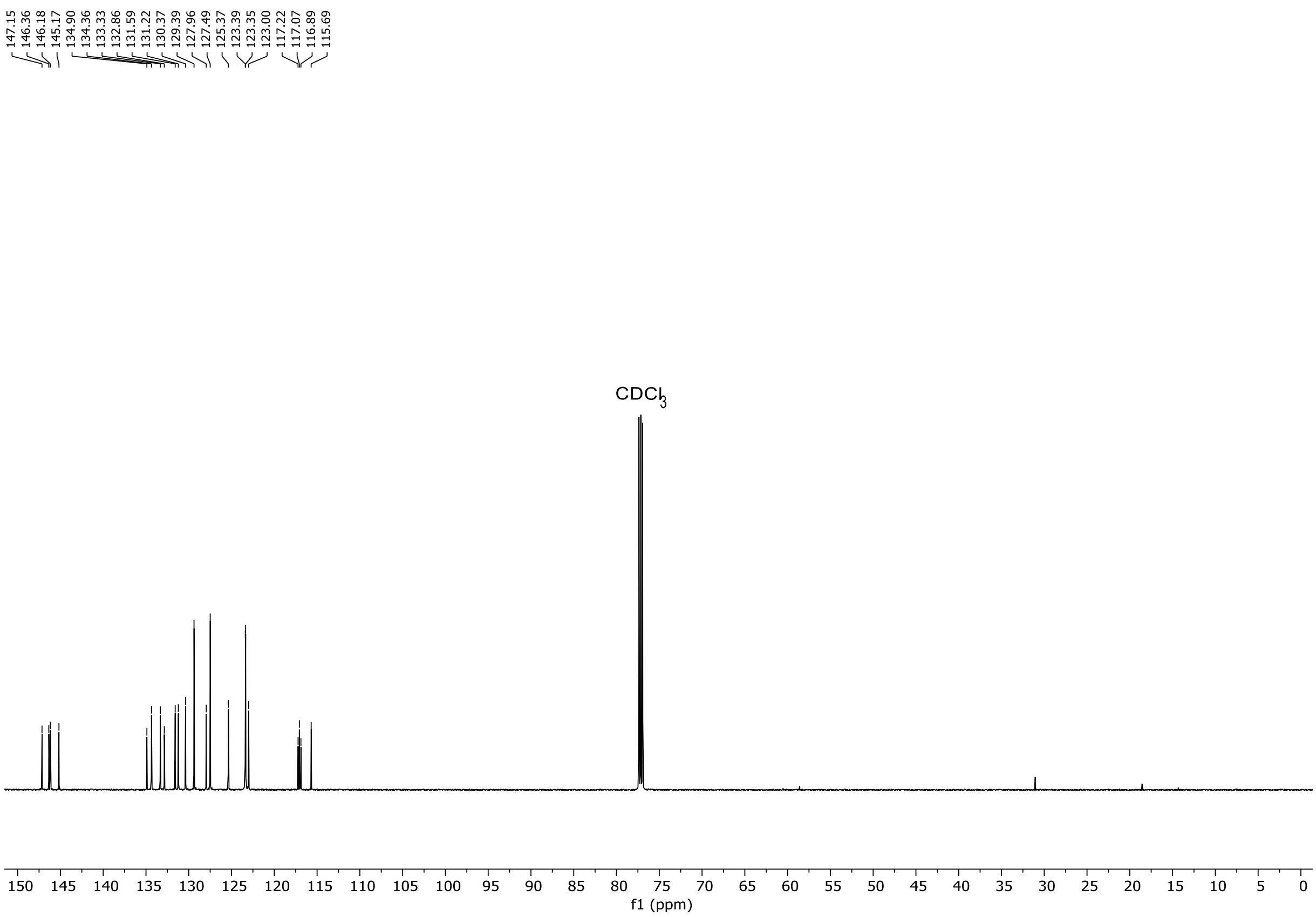

Figure S23. ^{13}C NMR spectrum (chloroform-d$_1$, 151 MHz, 298 K) of compound **6d**.

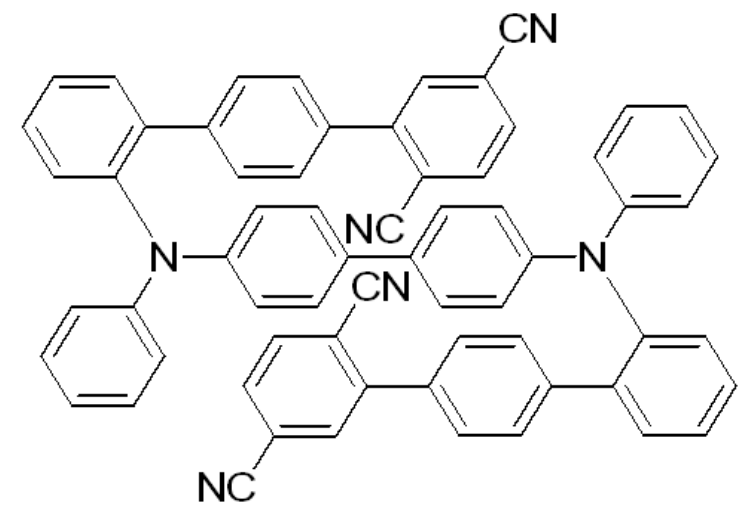

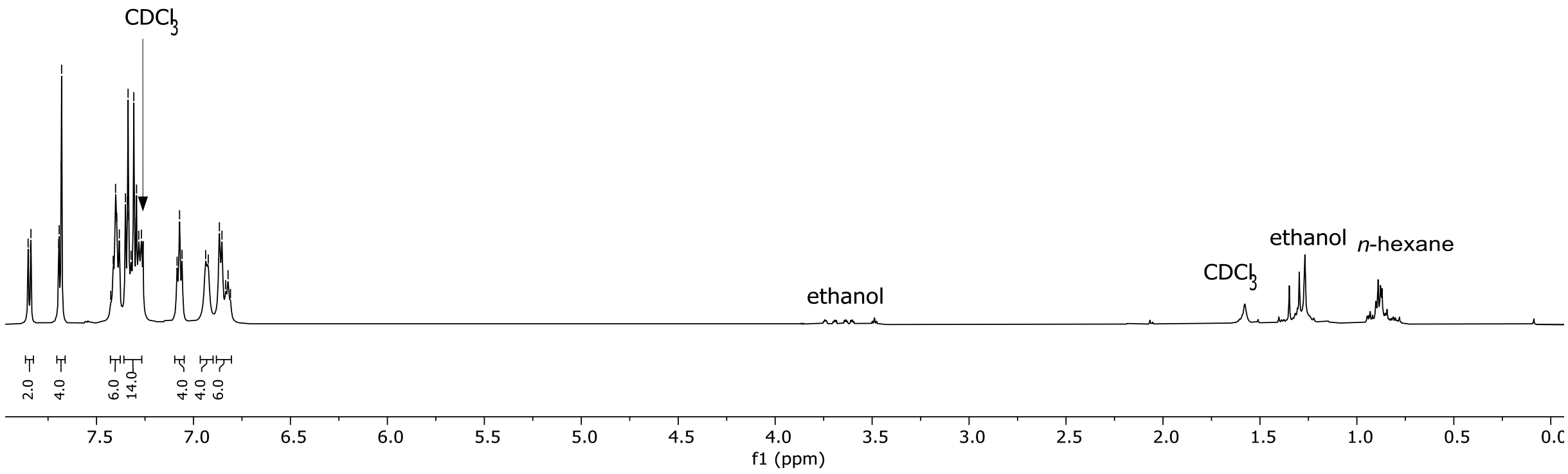

Figure S24. ^{1}H NMR spectrum (chloroform-d$_1$, 600 MHz, 298 K) of compound **6e**.

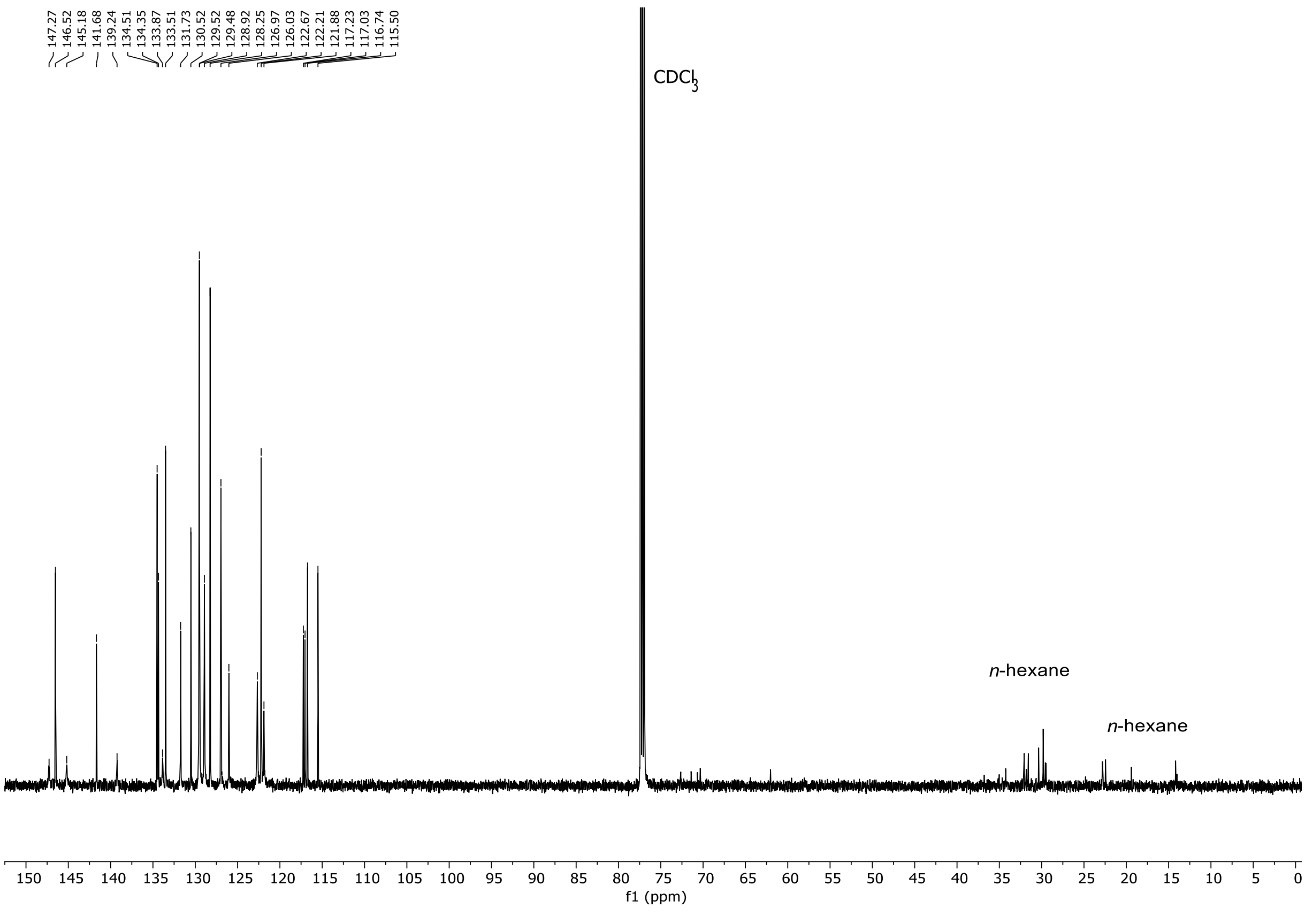

Figure S25. ^{13}C NMR spectrum (chloroform-d$_1$, 151 MHz, 299 K) of compound **6e**.

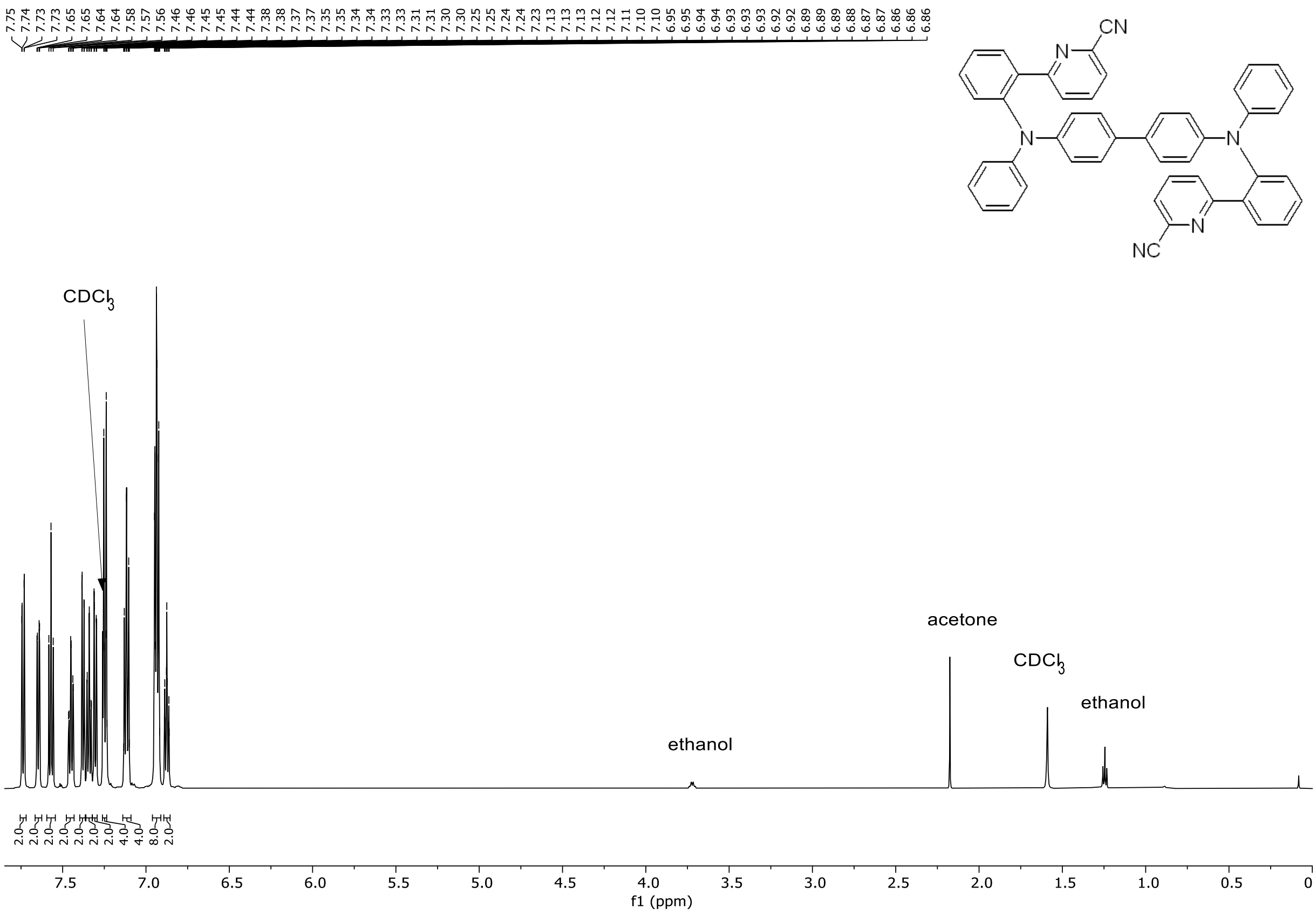

Figure S26. ^{1}H NMR spectrum (chloroform-d_1, 600 MHz, 298 K) of compound **7a**.

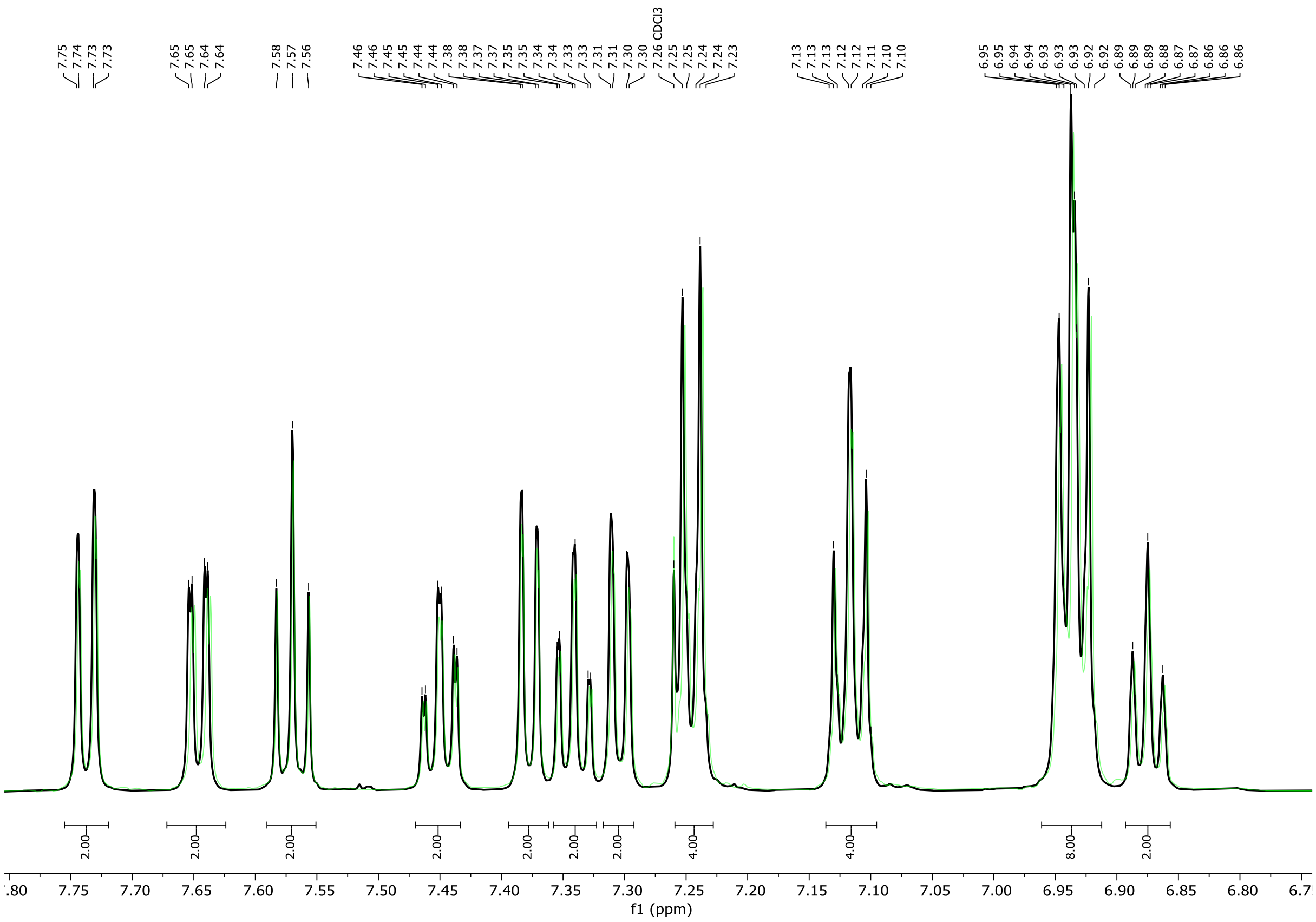

Figure S27. ^{1}H NMR spectrum in the range of 7.80–6.75 ppm (chloroform-d_1, 600 MHz, 298 K) of compound **7a** synthesized by dimerization (black) and by BLEBS sequence (green).

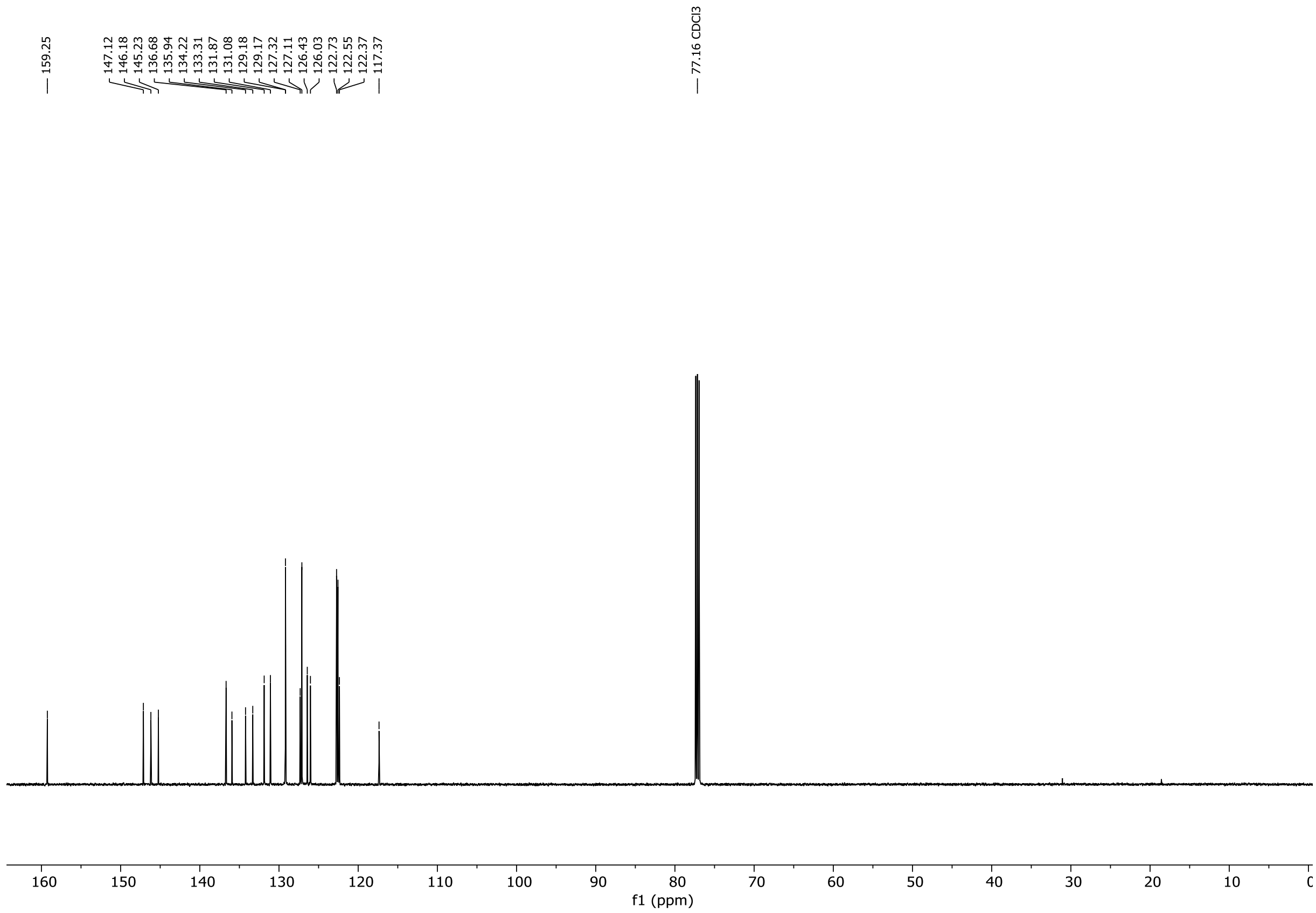

Figure S28. ^{13}C NMR spectrum (chloroform-d_1, 151 MHz, 298 K) of compound **7a**.

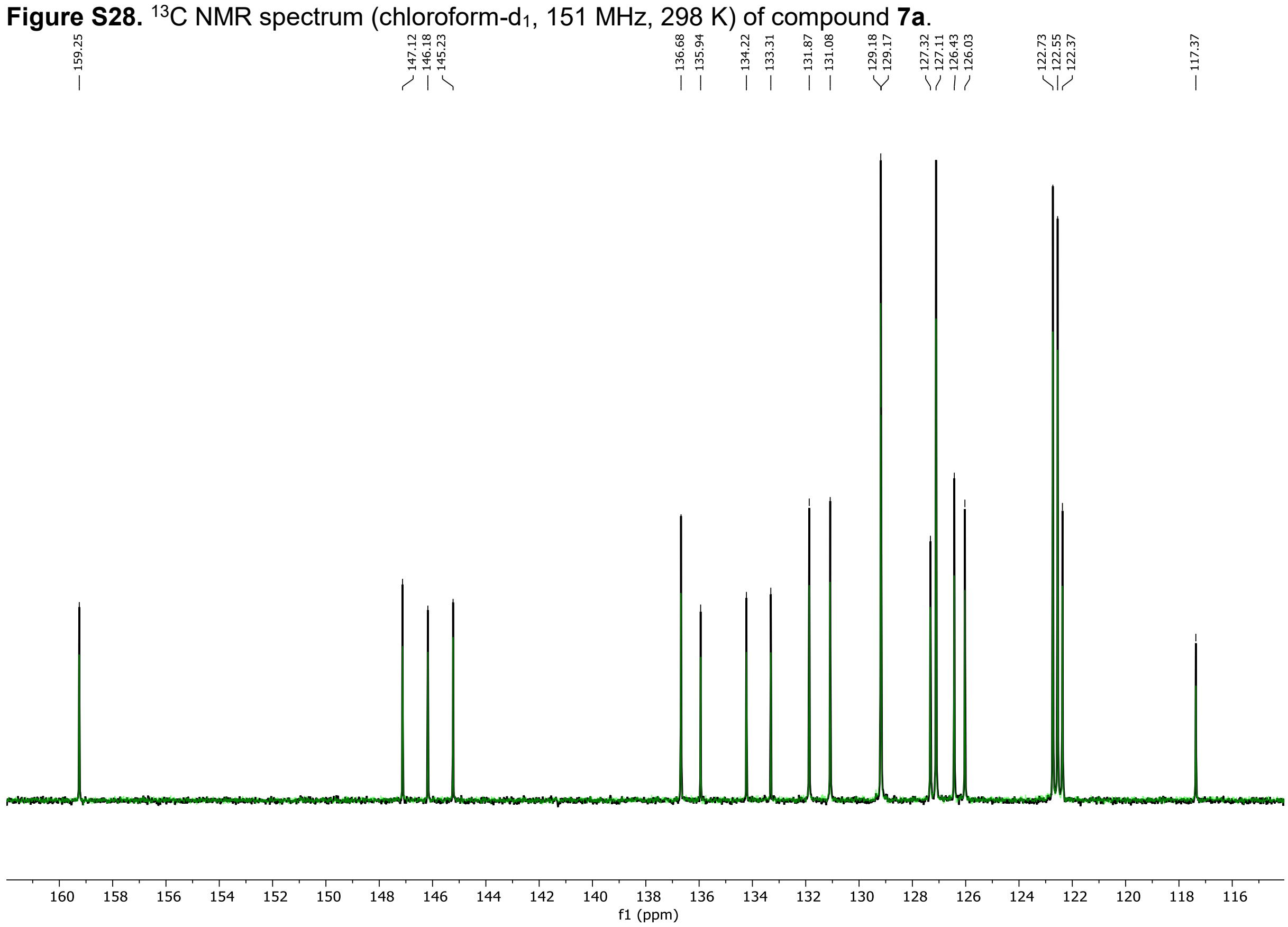

Figure S29. ^{13}C NMR spectrum in the range of 162–114 ppm (chloroform-d_1, 151 MHz, 298 K) of compound **7a** synthesized by dimerization (black) and by BLEBS sequence (green).

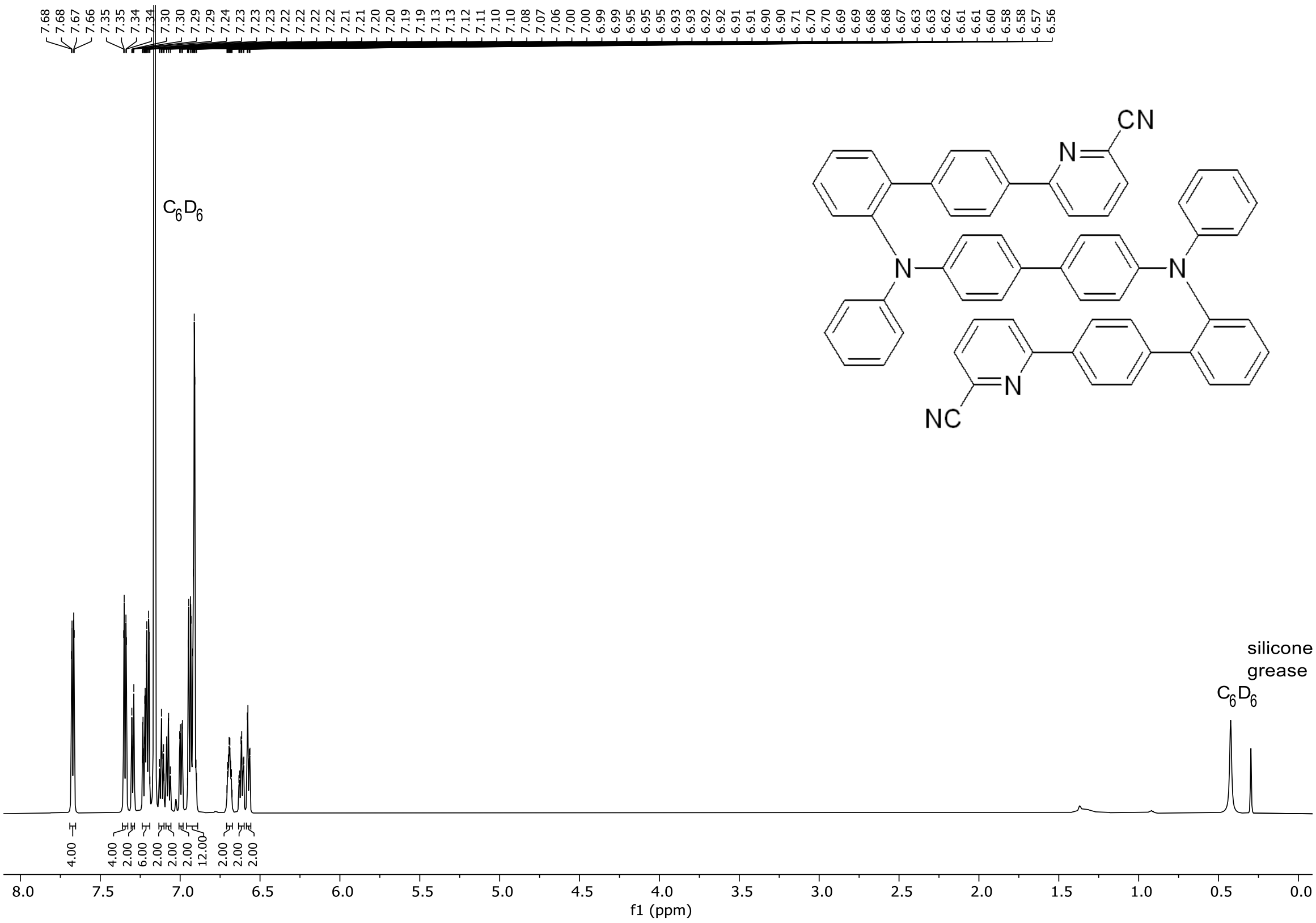

Figure S30. ^1H NMR spectrum (benzene-d_6, 600 MHz, 298 K) of compound **7b**.

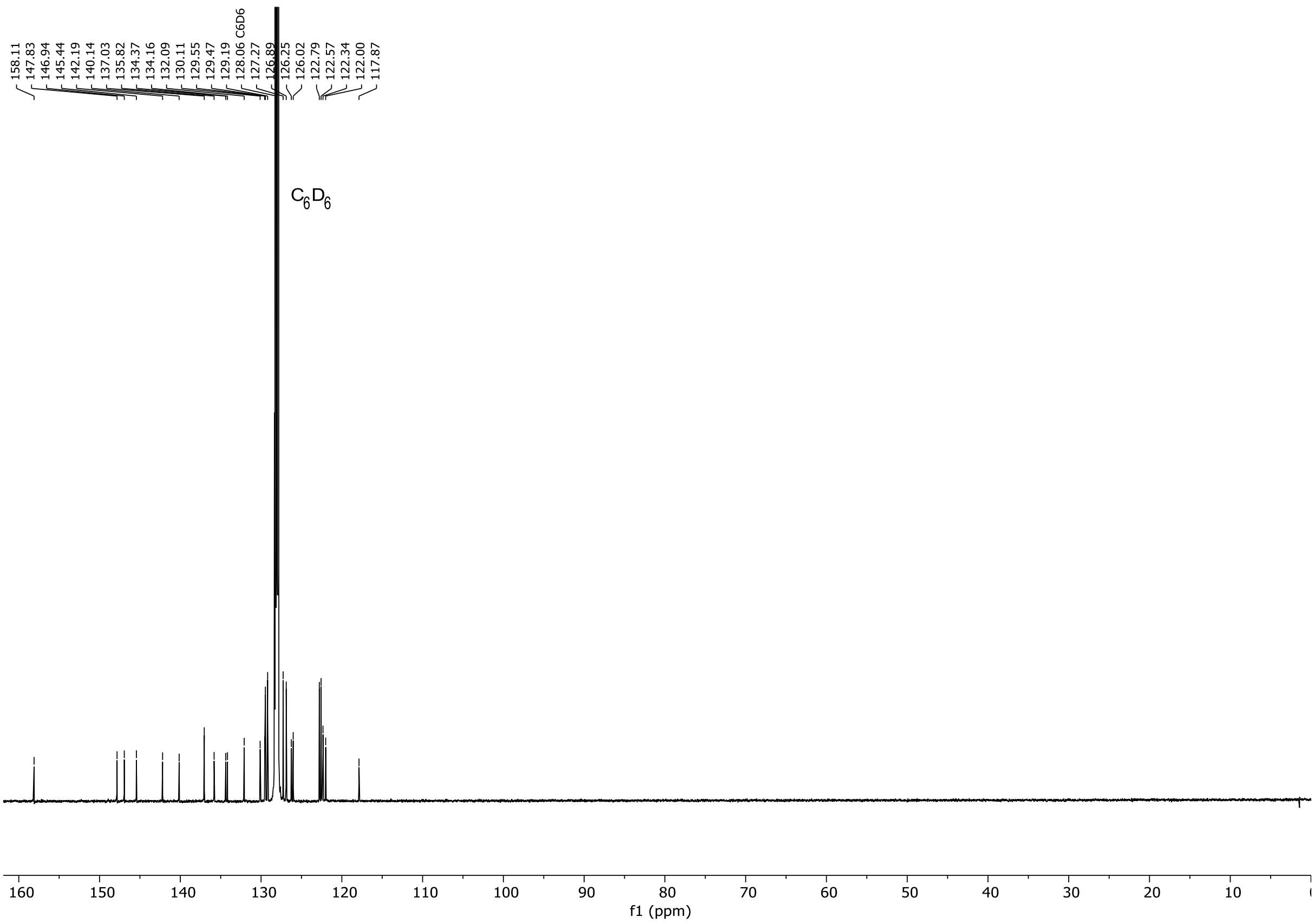

Figure S31. ^{13}C NMR spectrum (benzene-d_6, 151 MHz, 298 K) of compound **7b**.

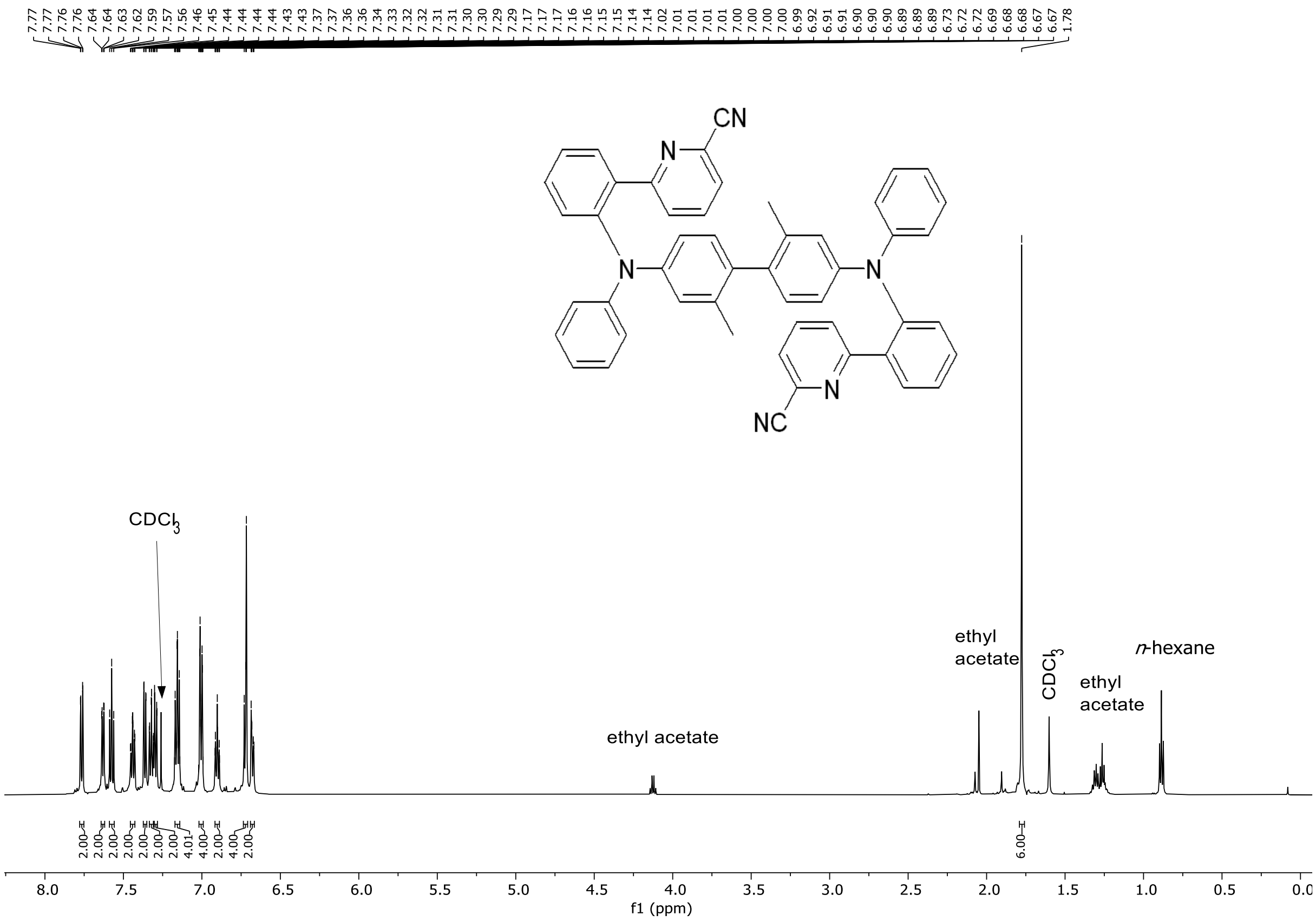

Figure S32. ^{1}H NMR spectrum (chloroform-d$_1$, 600 MHz, 298 K) of compound **7c**.

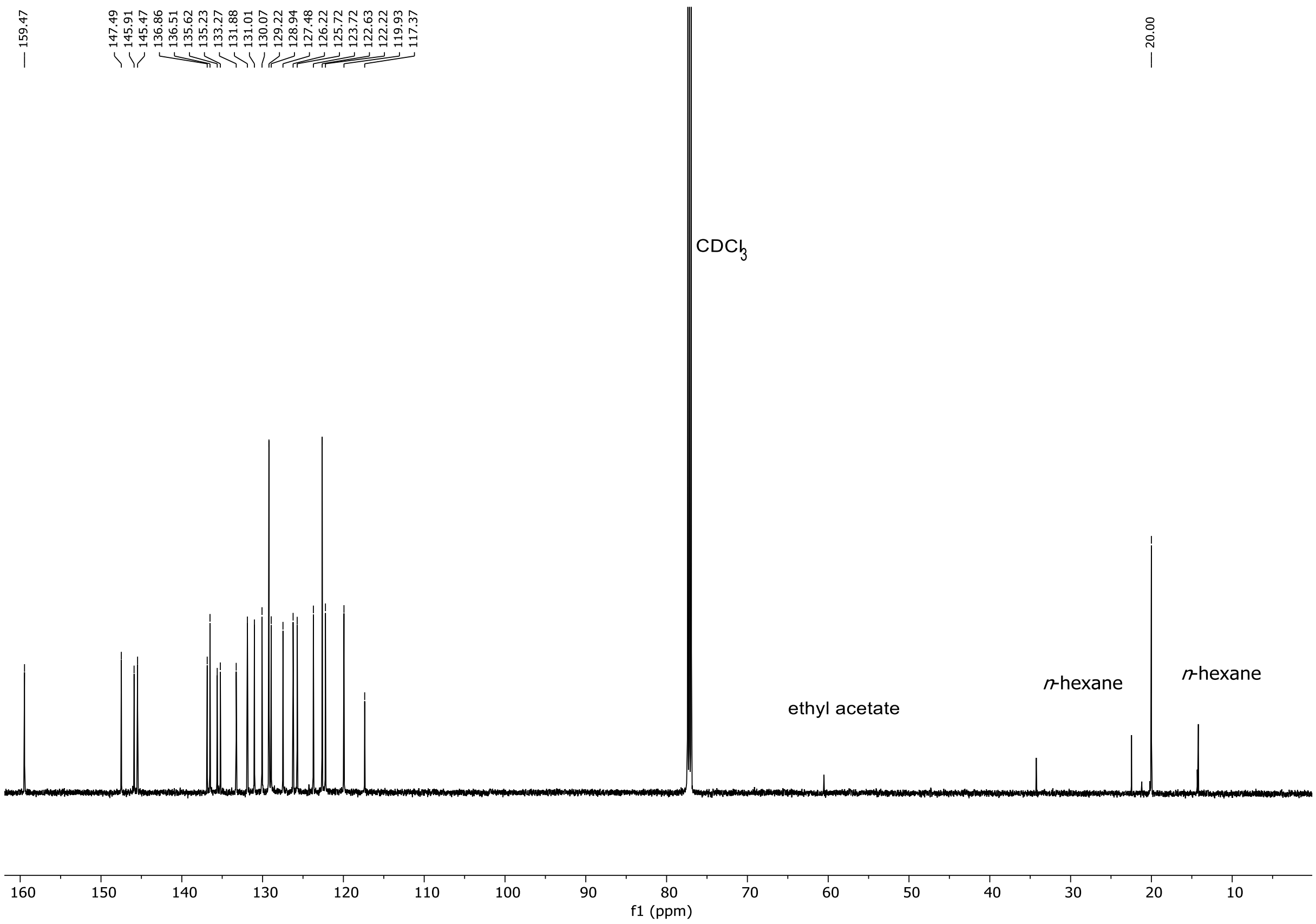

Figure S33. ^{13}C NMR spectrum (chloroform-d$_1$, 151 MHz, 298 K) of compound **7c**.

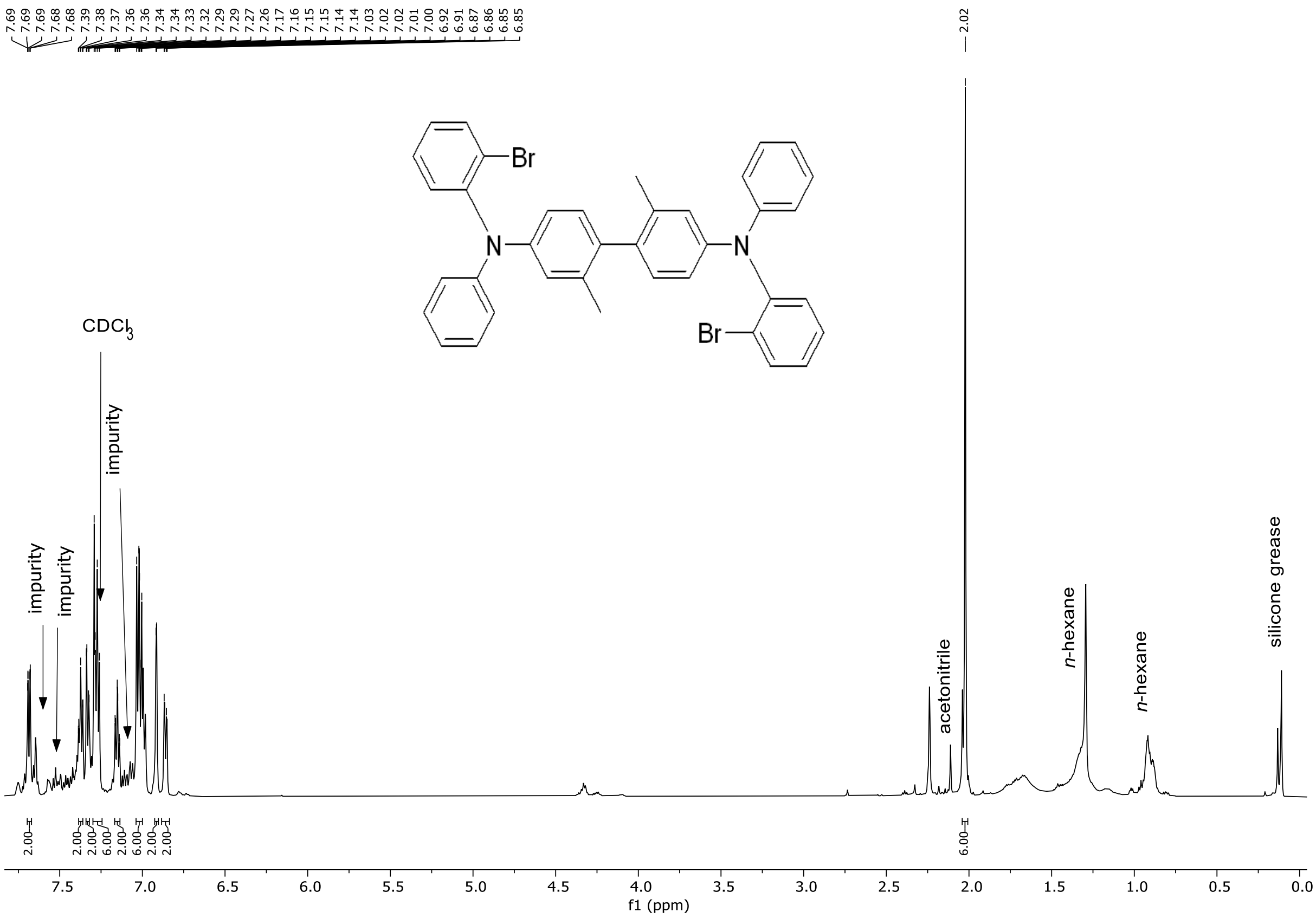

Figure S34. ^{1}H NMR spectrum (chloroform-d$_1$, 600 MHz, 298 K) of compound **10**.

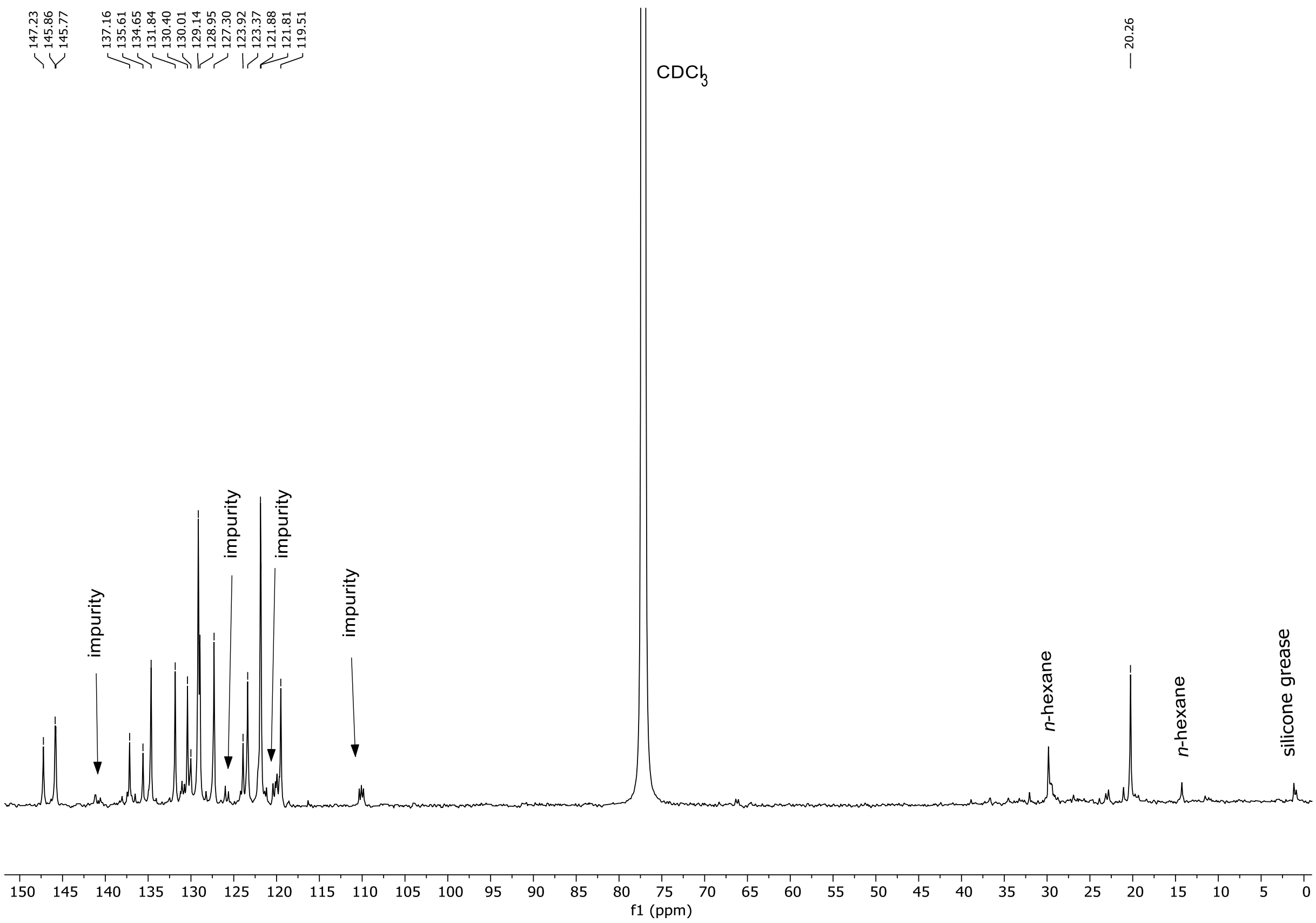

Figure S35. ^{13}C NMR spectrum (chloroform-d$_1$, 151 MHz, 298 K) of compound **10**.

4.2 High-Performance Liquid Chromatography (HPLC) spectra of *meta*/*ortho*/*para*-triarylamine dimers (*sym*-*m*/*o*/*p*-bTAA)$_2$ 4, 5c, 6, and 7

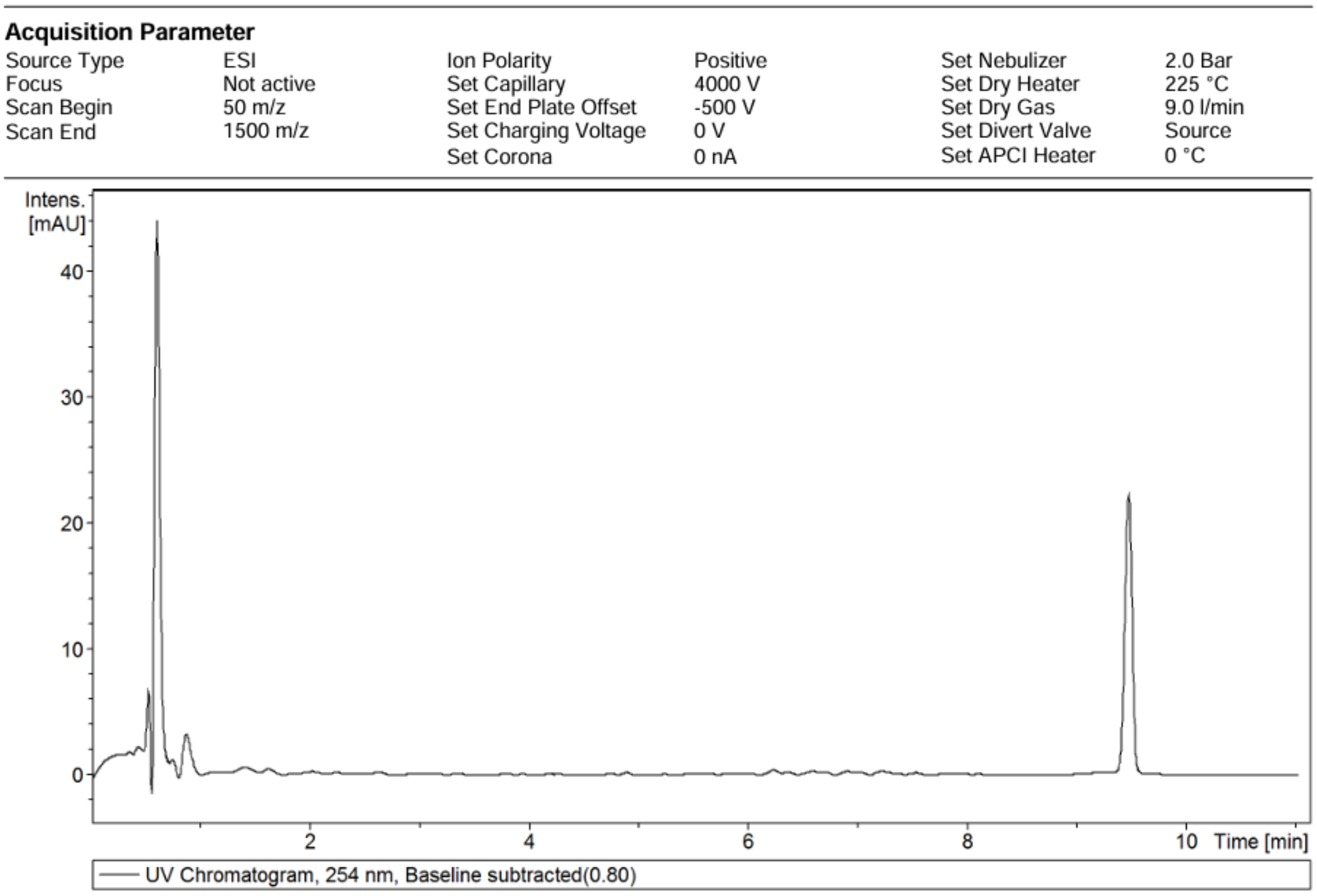

Figure S36. HPLC spectrogram of compound **4a**.

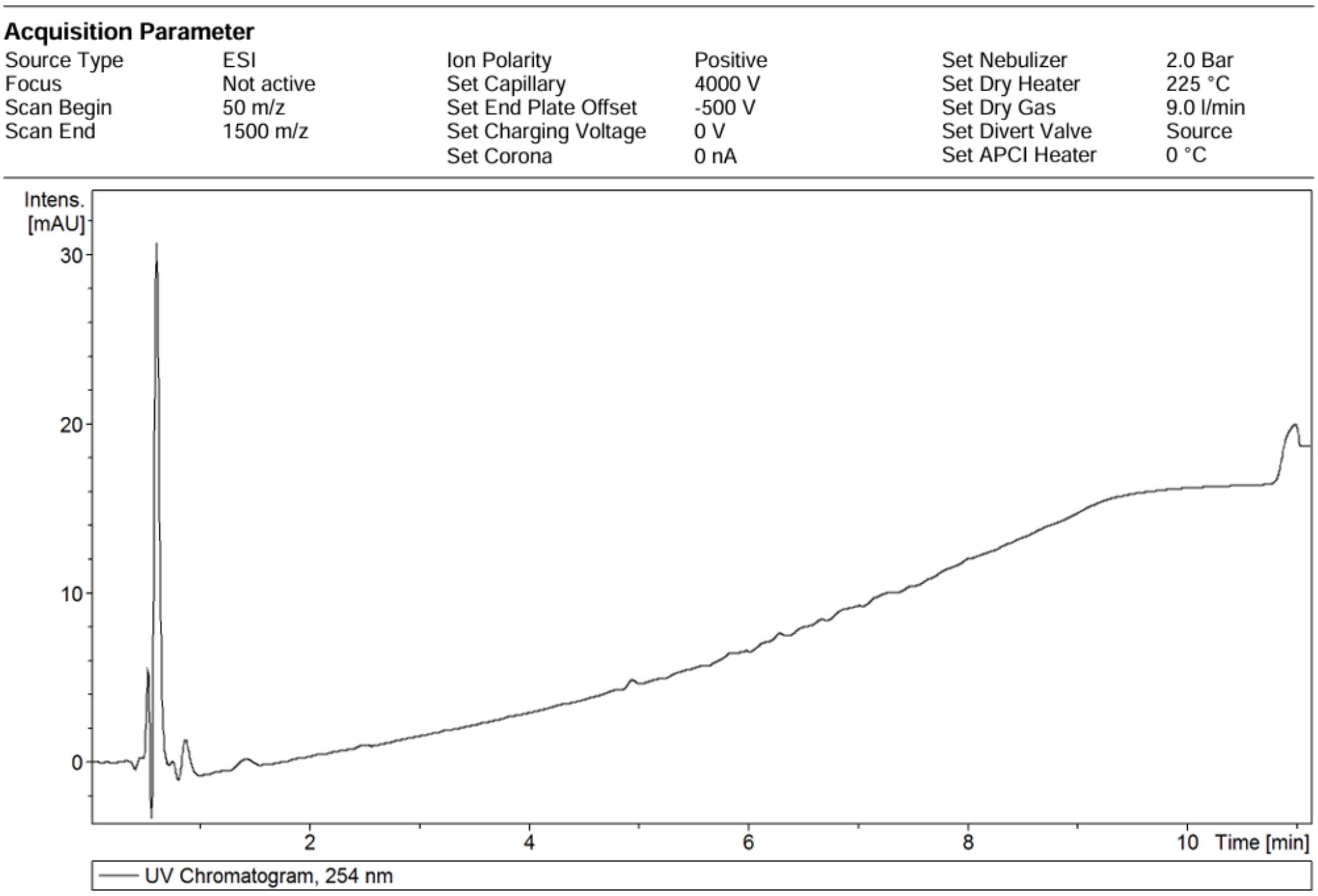

Figure S37. HPLC spectrogram of compound **4b**.

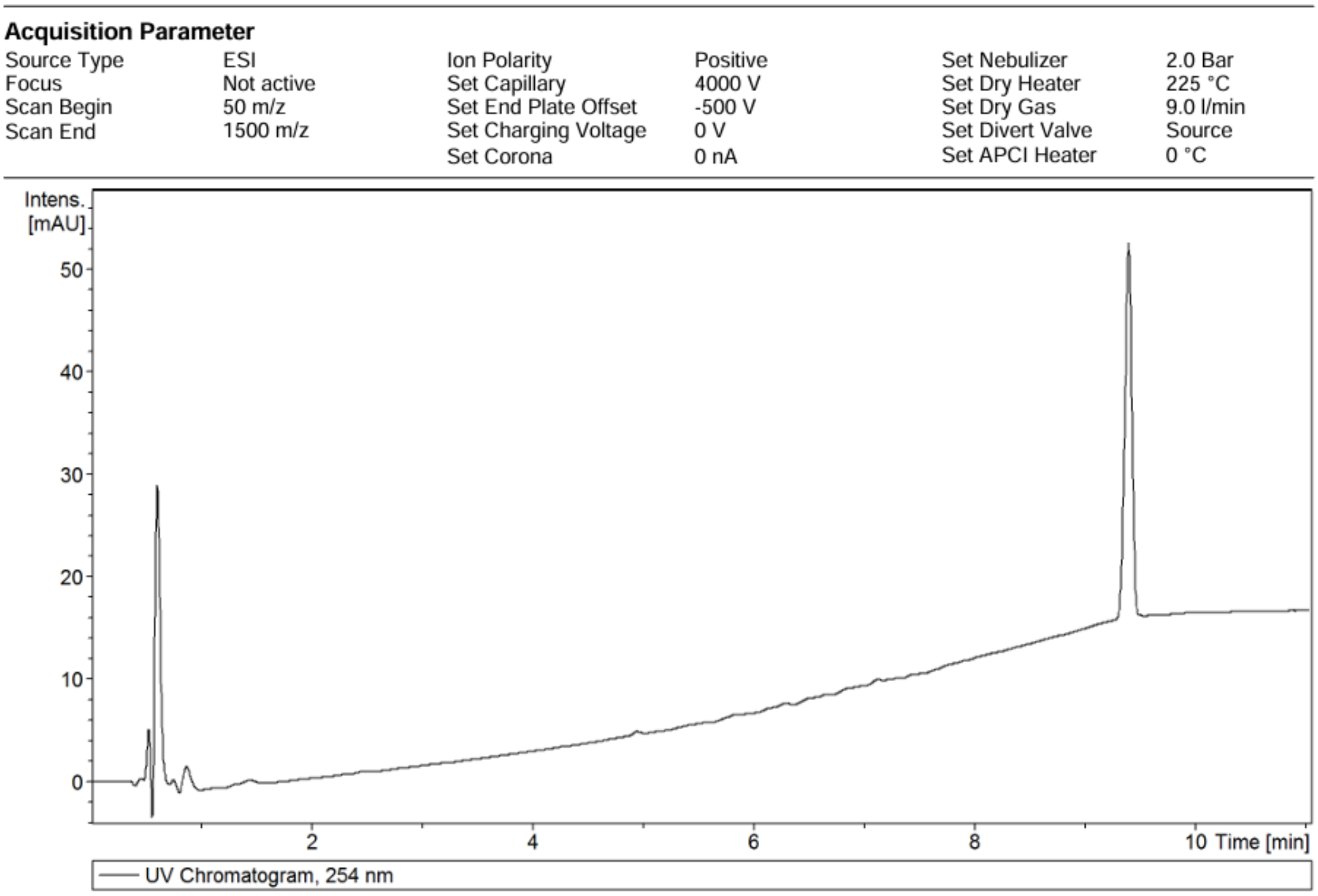

Figure S38. HPLC spectrogram of compound **4c**.

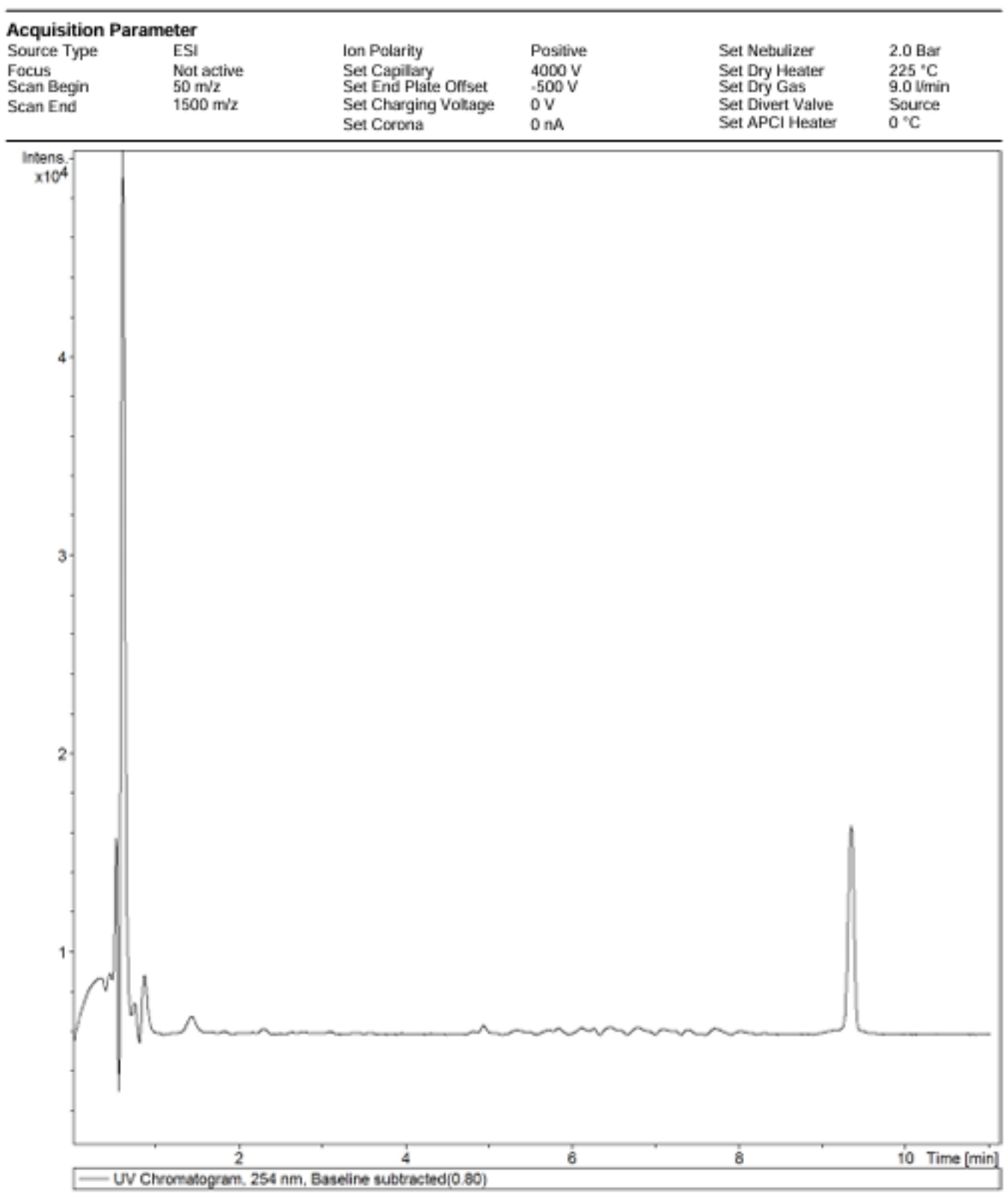

Figure S39. HPLC spectrogram of compound **5c**.

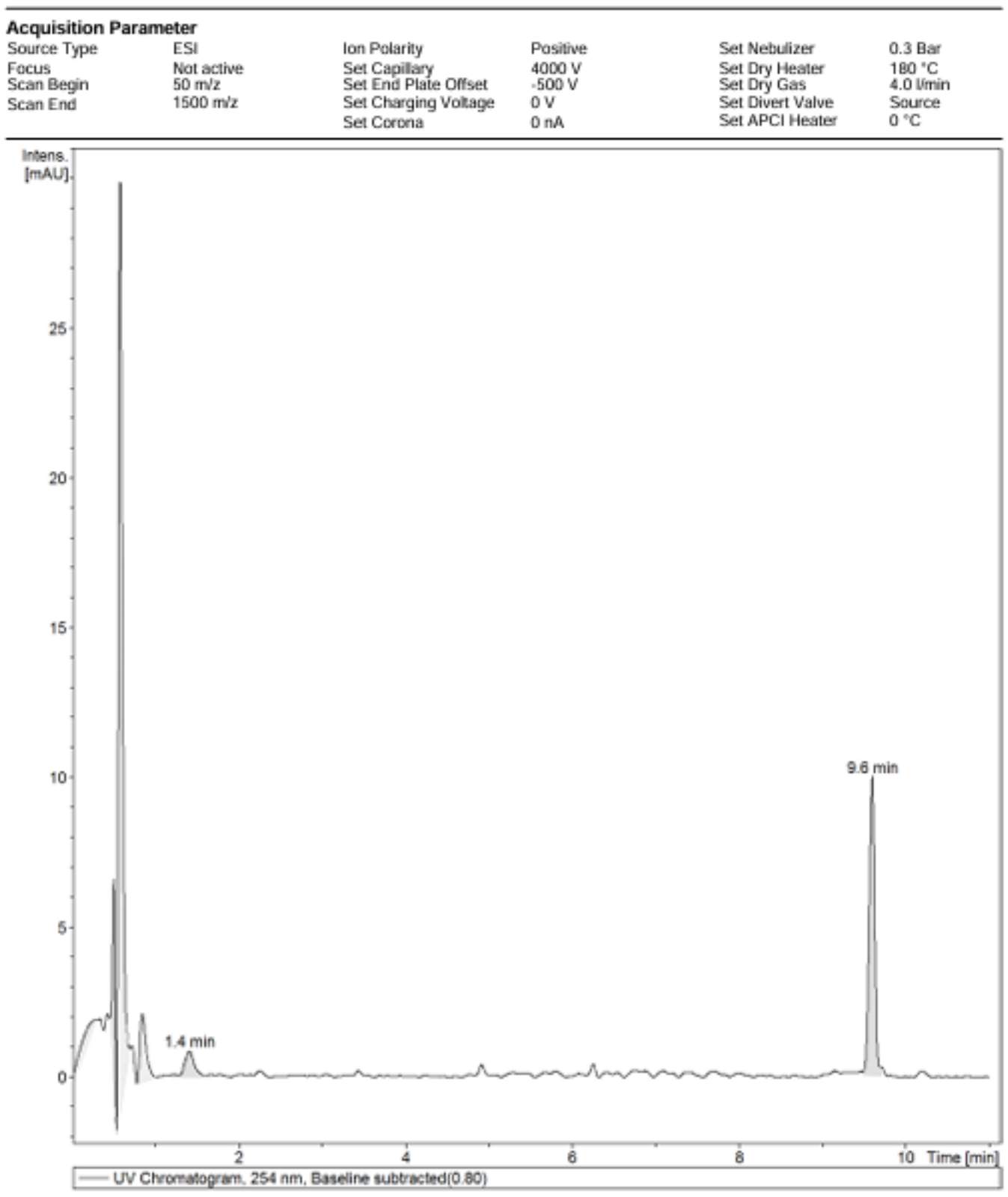

Figure S40. HPLC spectrogram of compound **6a**.

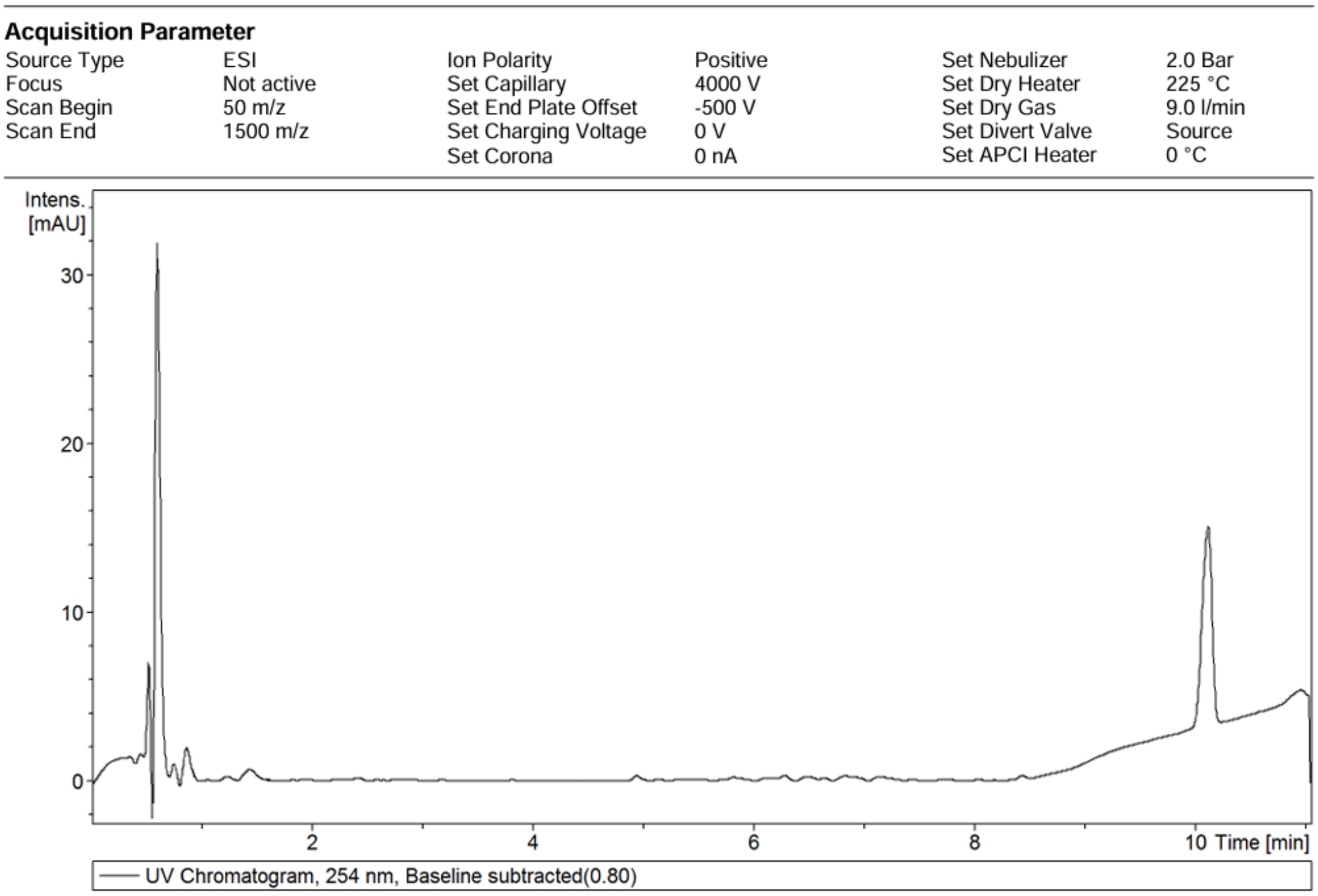

Figure S41. HPLC spectrogram of compound **6b**.

Acquisition Parameter

Source Type	ESI	Ion Polarity	Positive	Set Nebulizer	2.0 Bar
Focus	Not active	Set Capillary	4000 V	Set Dry Heater	225 °C
Scan Begin	50 m/z	Set End Plate Offset	-500 V	Set Dry Gas	9.0 l/min
Scan End	1500 m/z	Set Charging Voltage	0 V	Set Divert Valve	Source
		Set Corona	0 nA	Set APCI Heater	0 °C

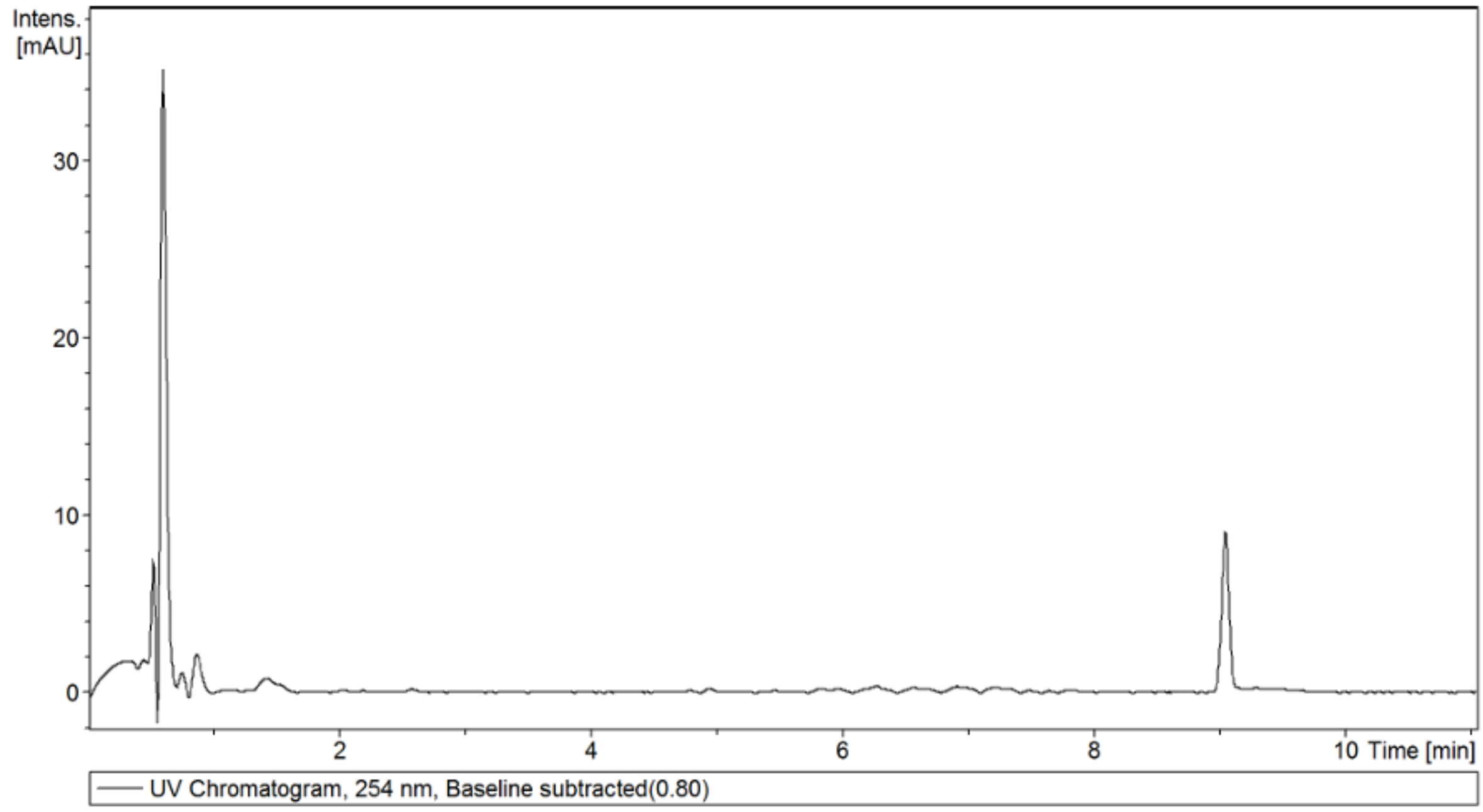

Figure S42. HPLC spectrogram of compound **6c**.

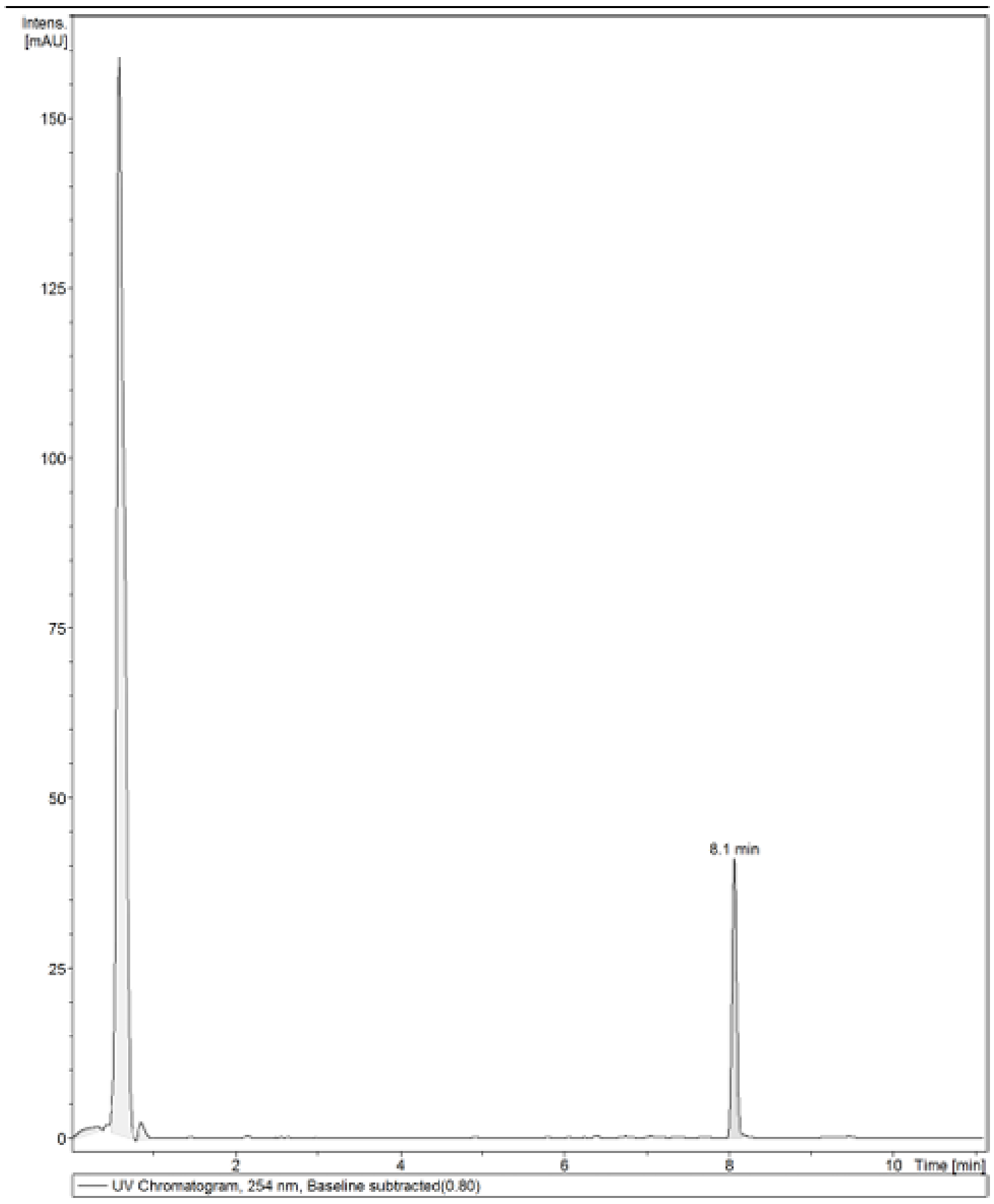

Figure S43. HPLC spectrogram of compound **6d**.

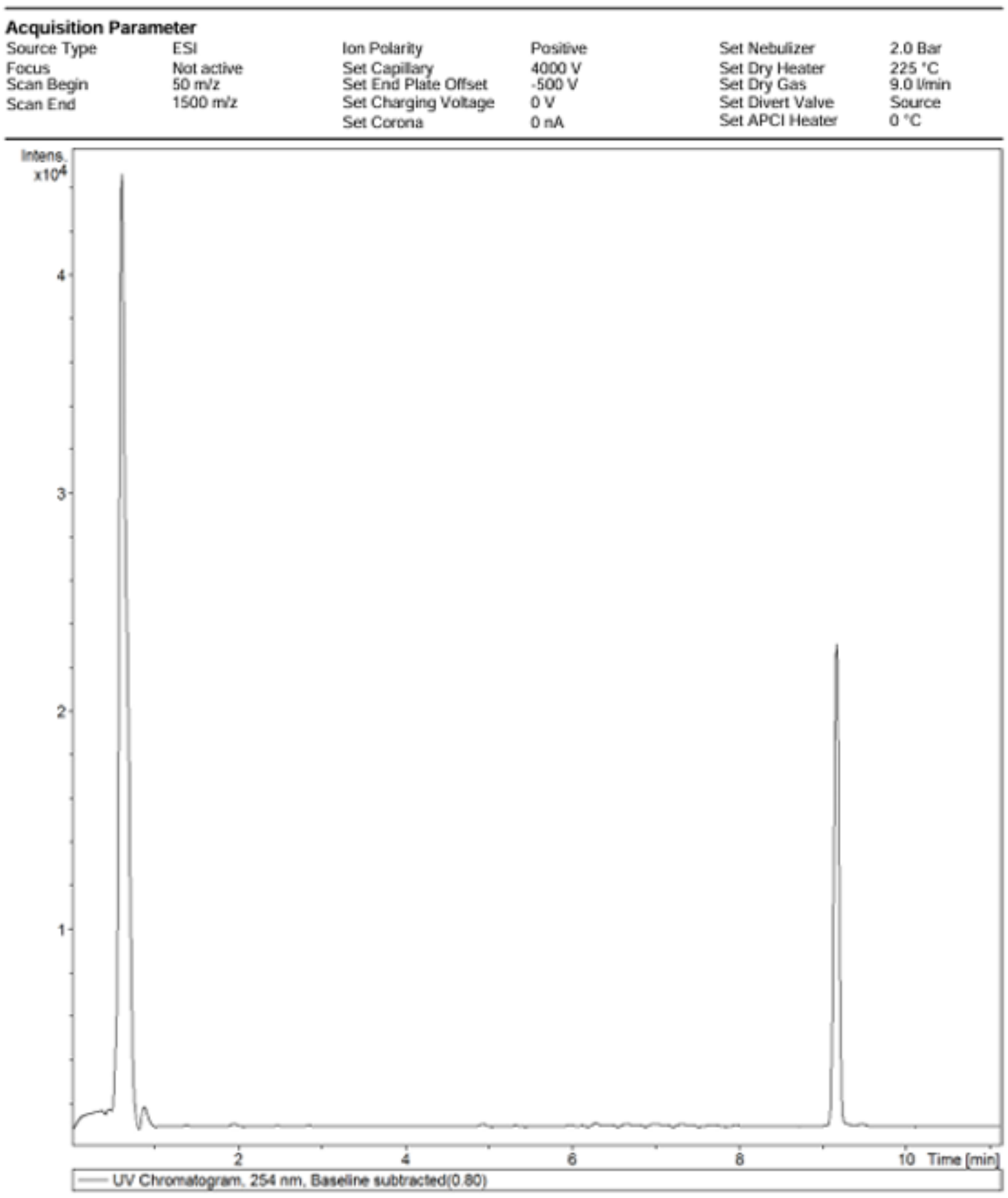

Figure S44. HPLC spectrogram of compound **6e**.

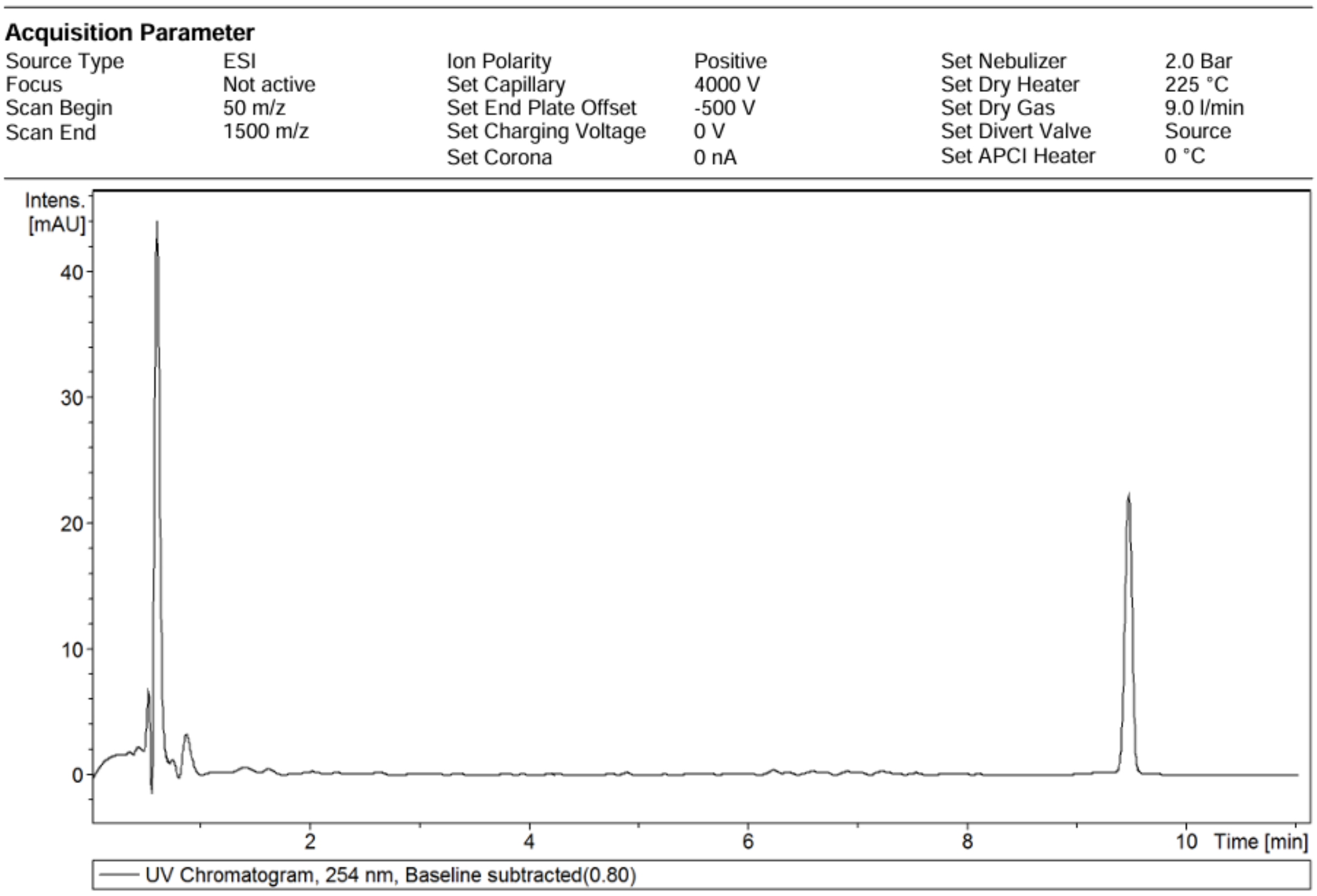

Figure S45. HPLC spectrogram of compound **7b**.

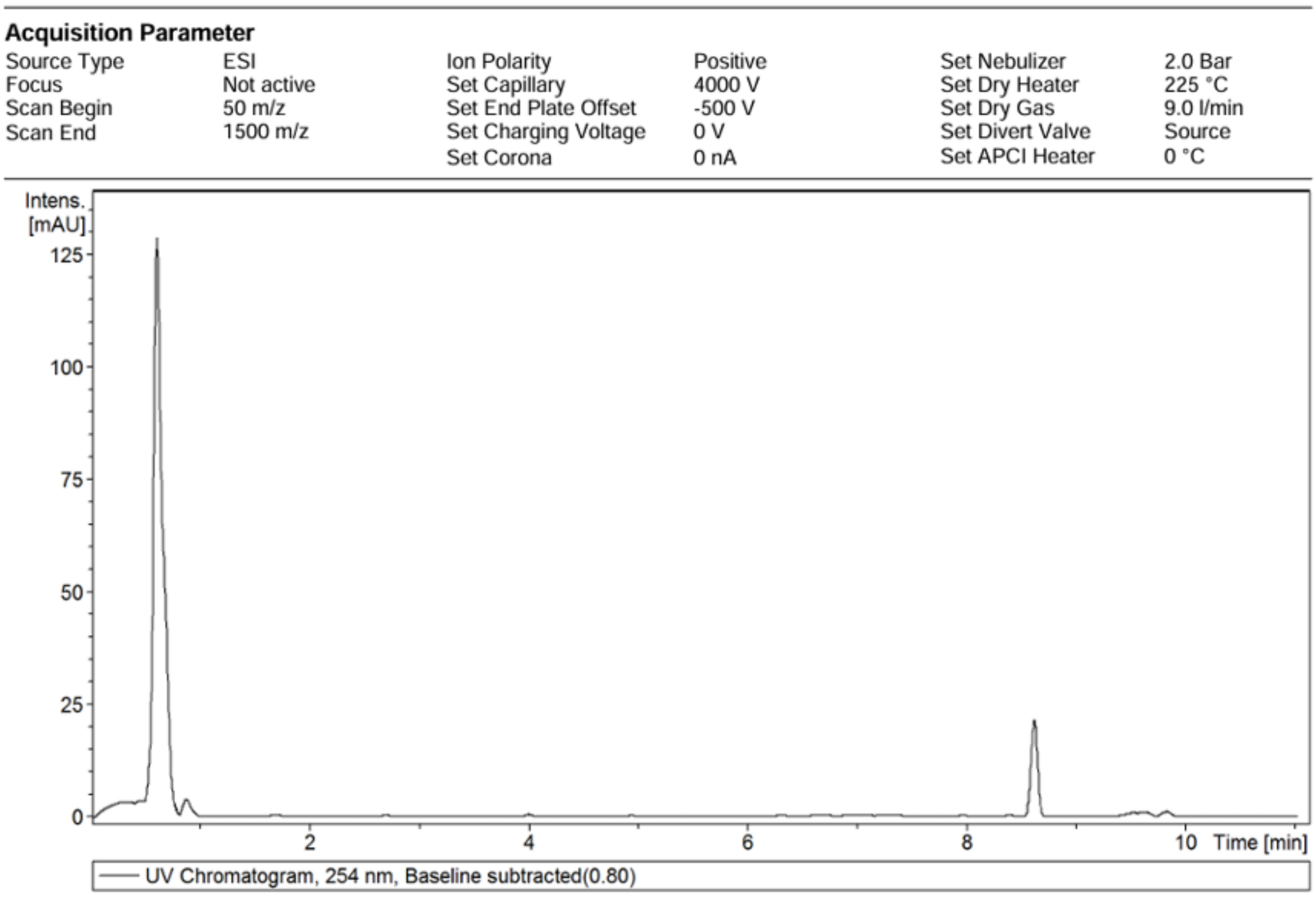

Figure S46. HPLC spectrogram of compound **7c**.

5 Cyclic voltammetry

5.1 Dimerization process exemplified by a *sym-o*-bTAA[2]

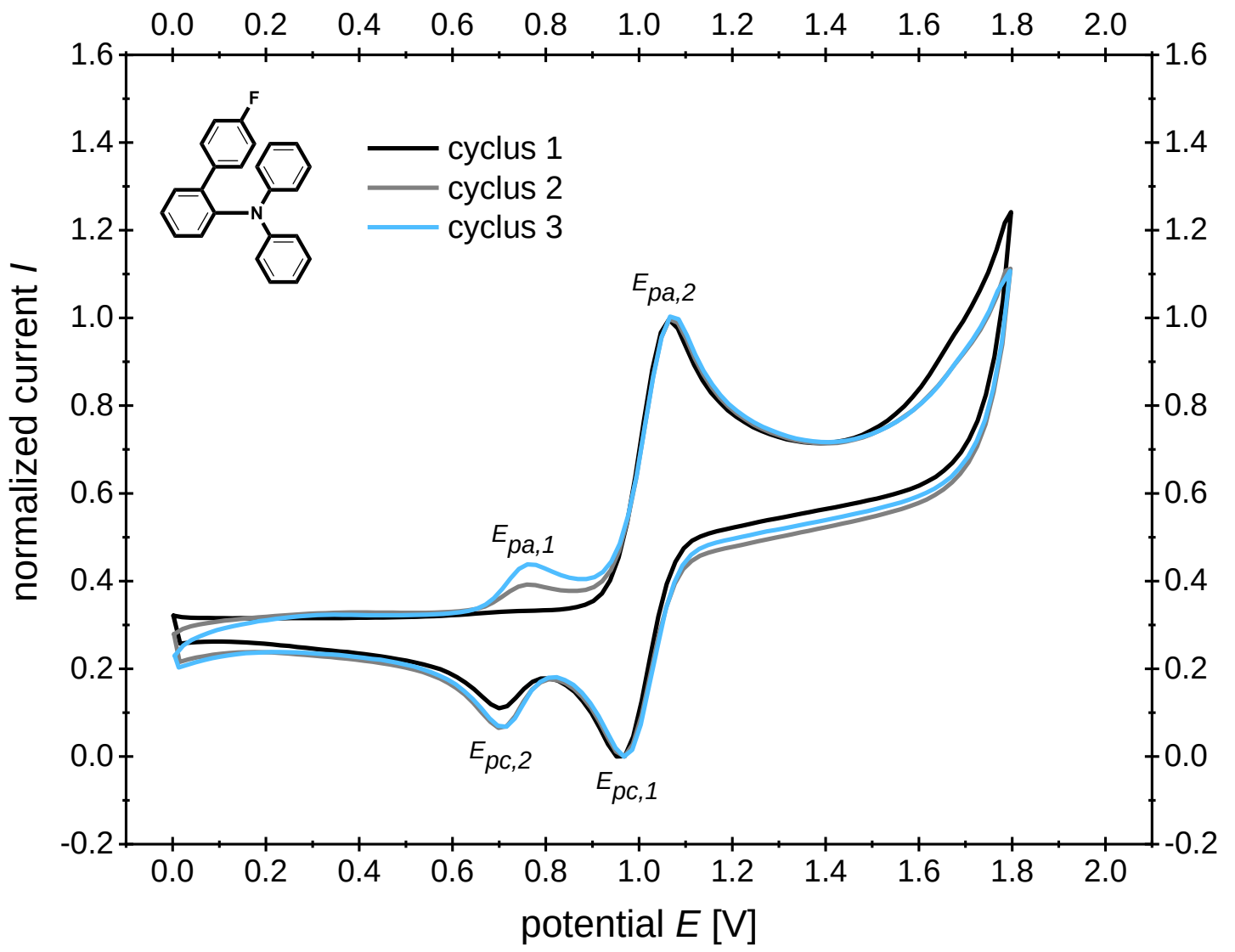

Figure S47. Cyclic voltammograms of *sym-o*-bTAA example recorded in dichloromethane (T = 293 K, ν = 100 mV/s, electrolyte: [^{n}Bu$_4$N][PF$_6$], Pt working electrode, Pt counter electrode, Ag/AgCl reference electrode, redox standard: decamethylferrocene $E_0^{0/+1}$ = -540 mV (vs. ferrocene $E_0^{0/+1}$ = 0.00 mV)[8]).

5.2 Cyclic voltammograms of dimers and monomers with dimerization processes in comparison

5.2.1 Comparison of *sym-m*-bTAA 2 and *sym-m*-(bTAA)$_2$ 5

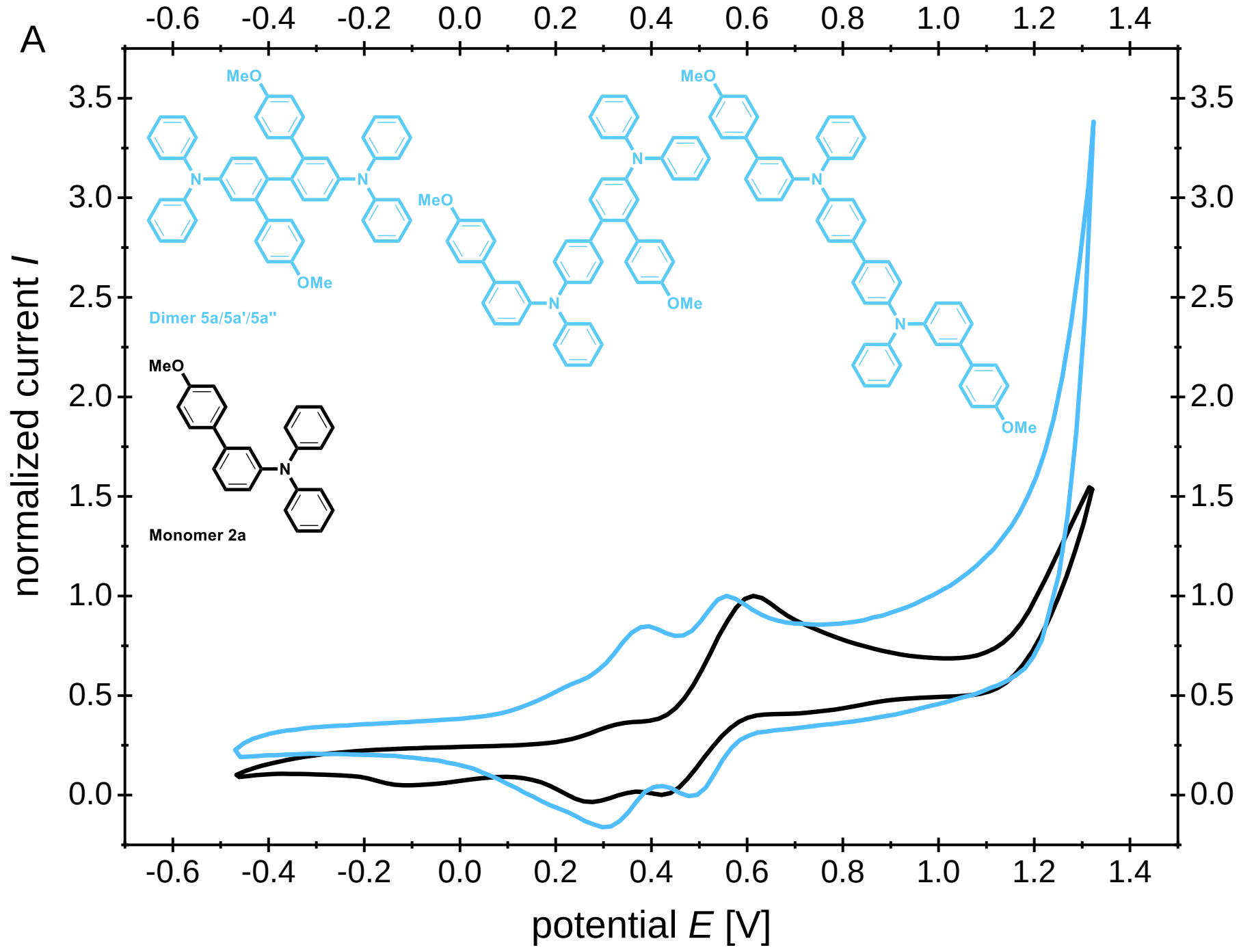

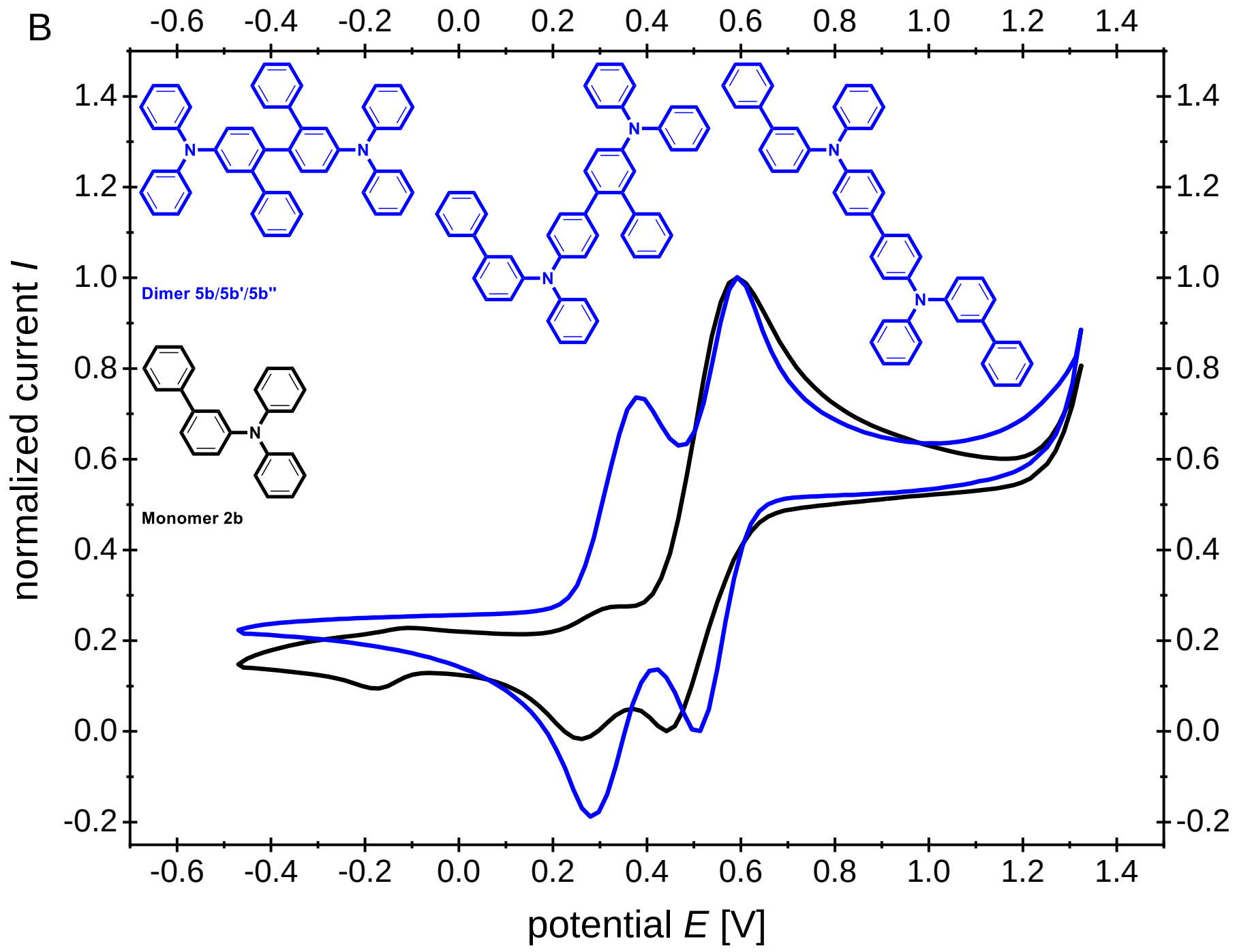

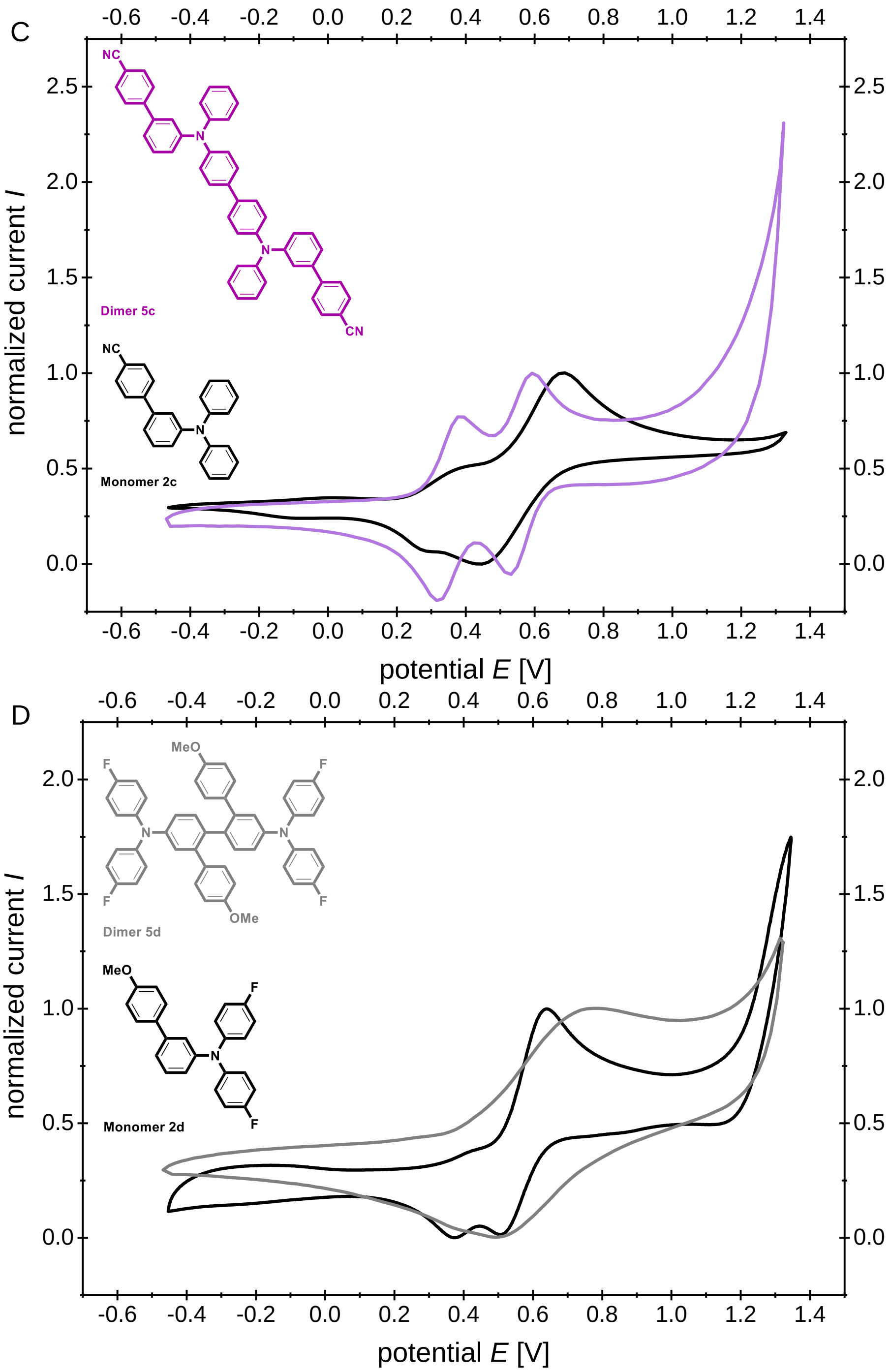

Figure S48. Cyclic voltammograms of *sym*-*m*-(bTAA)$_2$ **5** compared with the cyclic voltammograms of their monomers *sym*-*m*-bTAA **2** (A: mixture **5a**/**5a'**/**5a''** and **2a**, B: mixture **5b**/**5b'**/**5b''** and **2b**, C: **5c** und **2c,** D: **5d** and **2d**) recorded in dichloromethane (T = 293 K, ν = 100 mV/s, electrolyte: [^{n}Bu$_4$N][PF$_6$], Pt working electrode, Pt counter electrode, Ag/AgCl reference electrode, redox standard: decamethylferrocene $E_0^{0/+1}$ = -540 mV (vs. ferrocene $E_0^{0/+1}$ = 0.00 mV)[8]).

5.2.2 Comparison of *sym-o*-bTAA 3 and *sym-o*-(bTAA)$_2$ 6

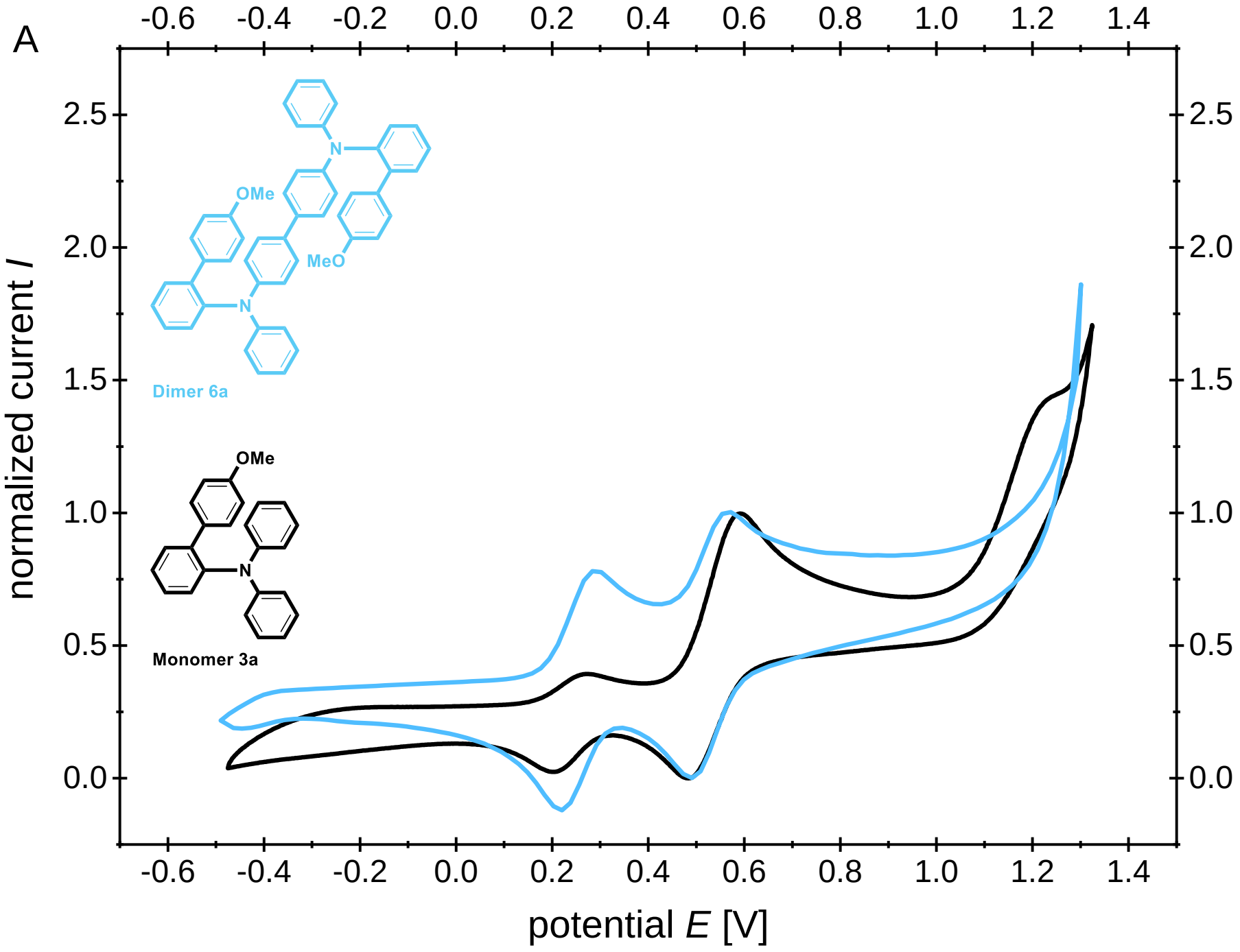

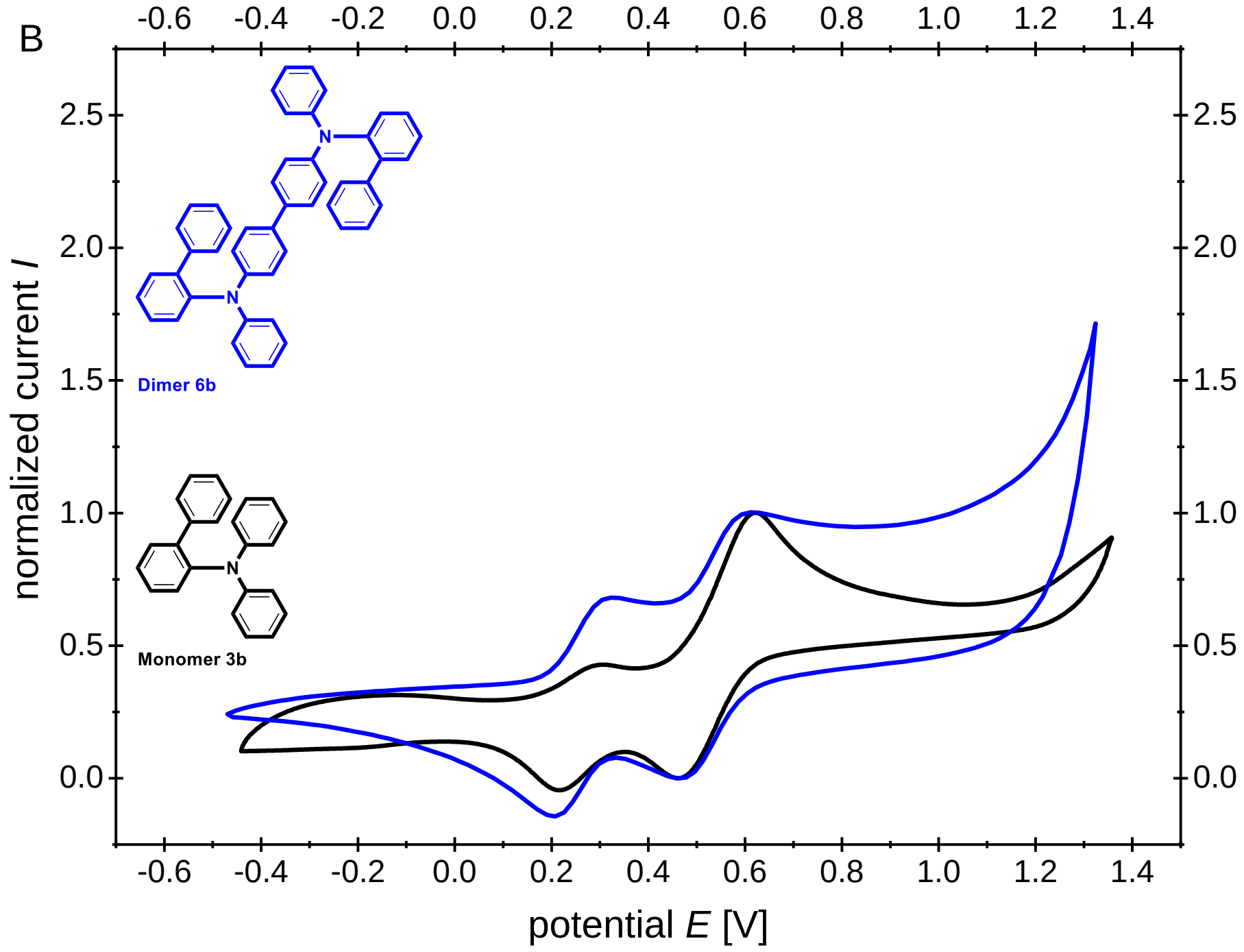

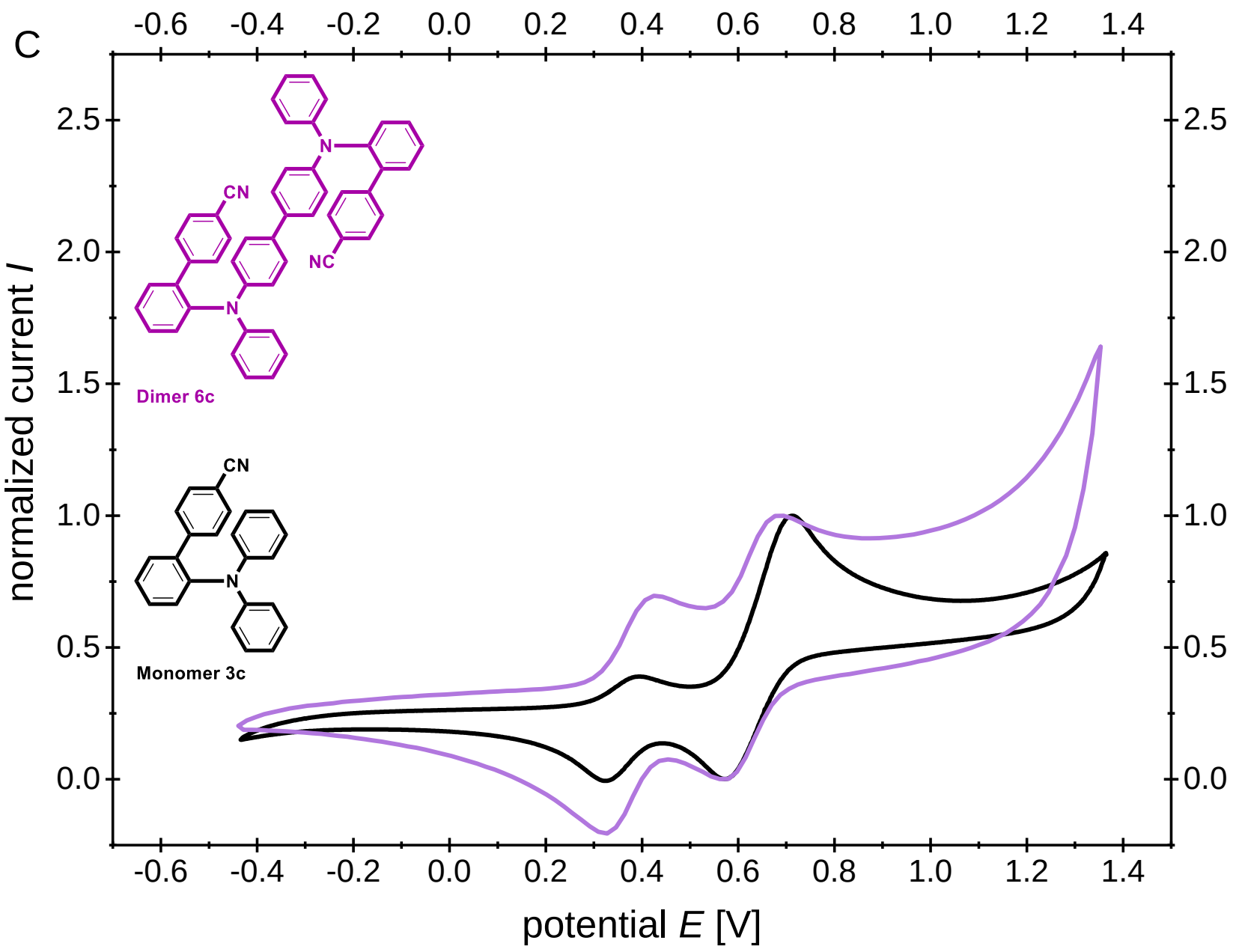
C
Dimer 6c
Monomer 3c
normalized current I
potential E [V]

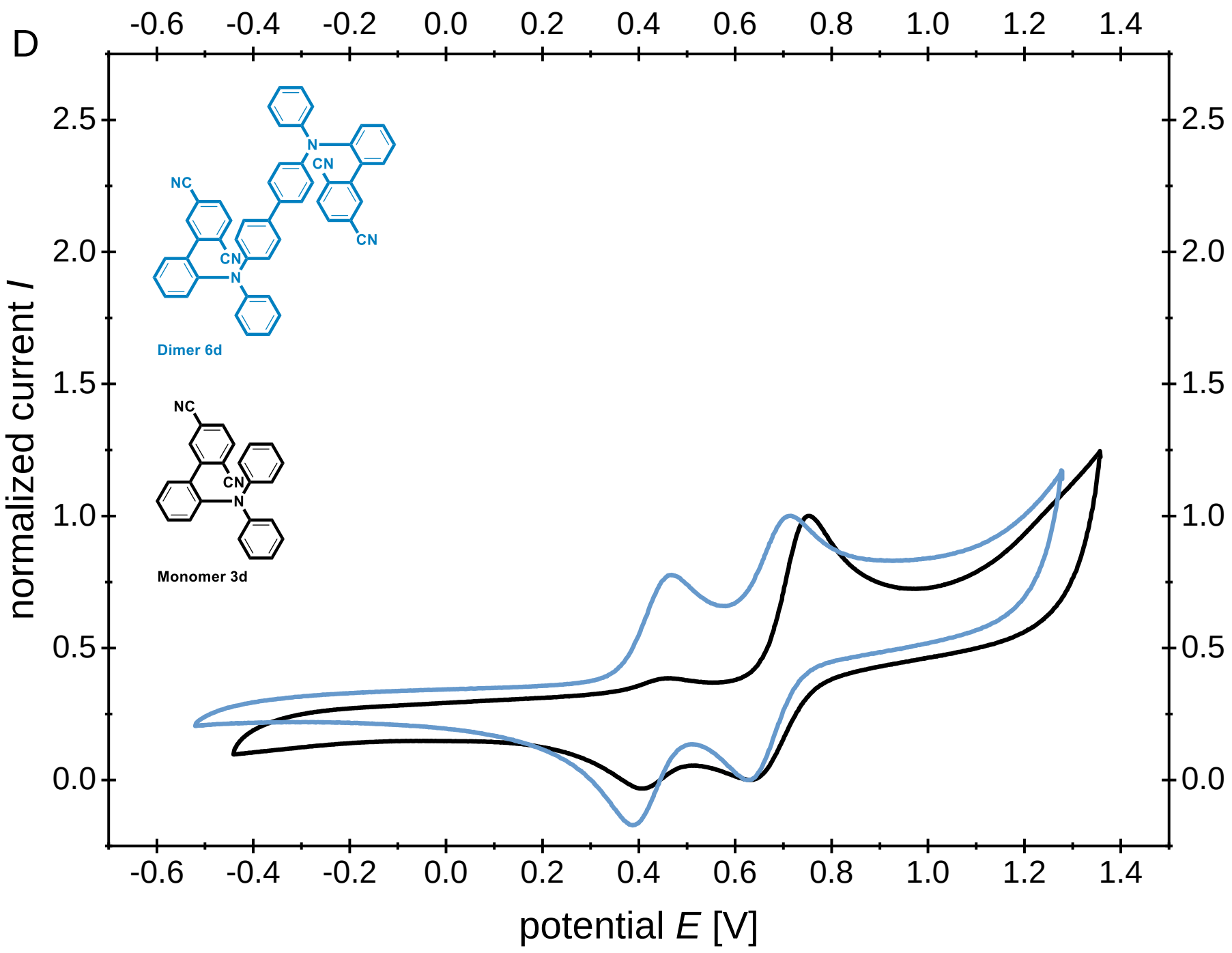
D
Dimer 6d
Monomer 3d
normalized current I
potential E [V]

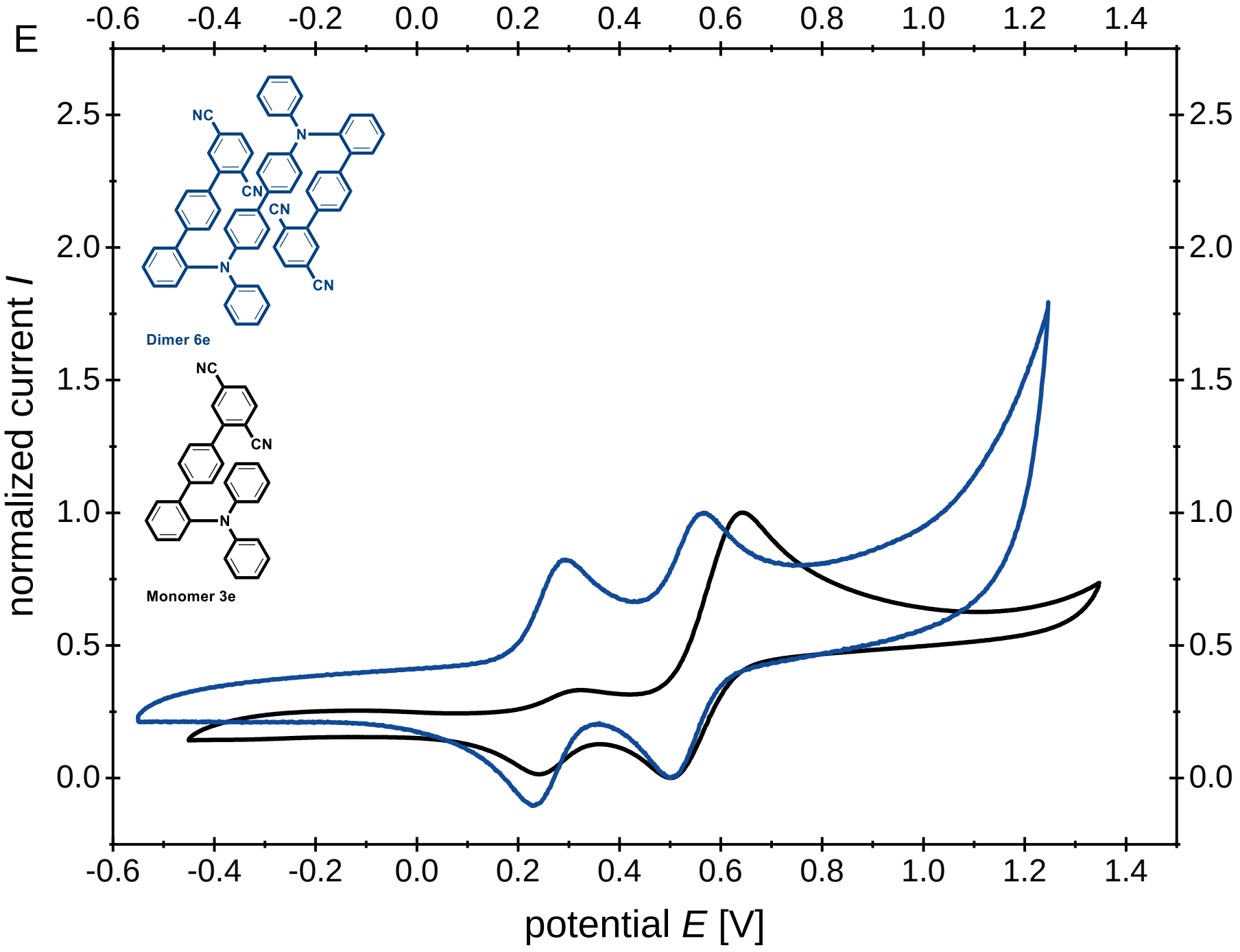

Figure S49. Cyclic voltammograms of *sym-o*-(bTAA)$_2$ **6** compared with the cyclic voltammograms of their monomers *sym-o*-bTAA **3** (A: **6a** and **3a**, B: **6b** and **3b**, C: **6c** and **3c**, D: **6d** and **3d**, E: **6e** and **3e**) recorded in dichloromethane (T = 293 K, ν = 100 mV/s, electrolyte: [^{n}Bu$_4$N][PF$_6$], Pt working electrode, Pt counter electrode, Ag/AgCl reference electrode, redox standard: decamethylferrocene $E_0^{0/+1}$ = -540 mV (vs. ferrocene $E_0^{0/+1}$ = 0.00 mV)[8]).

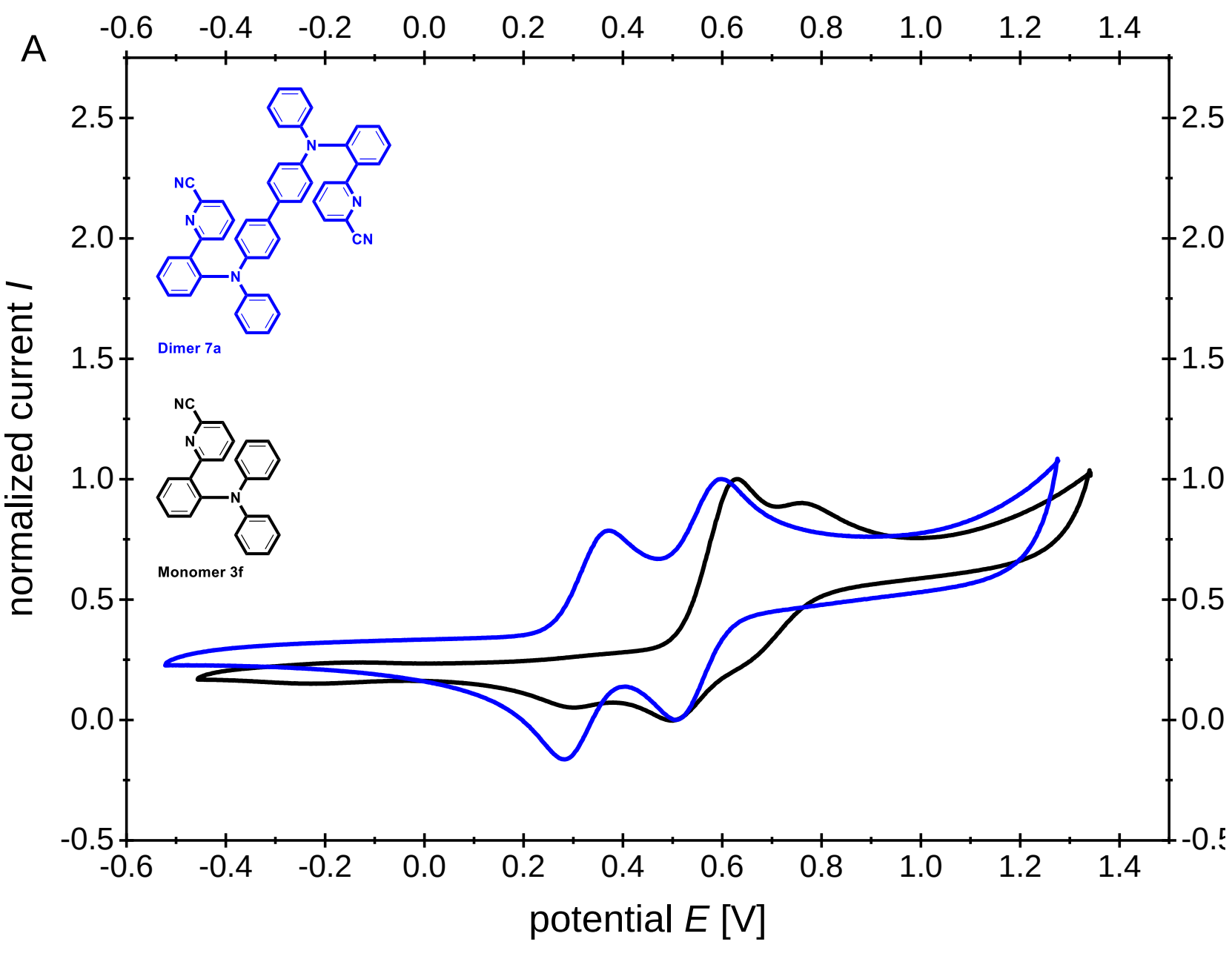

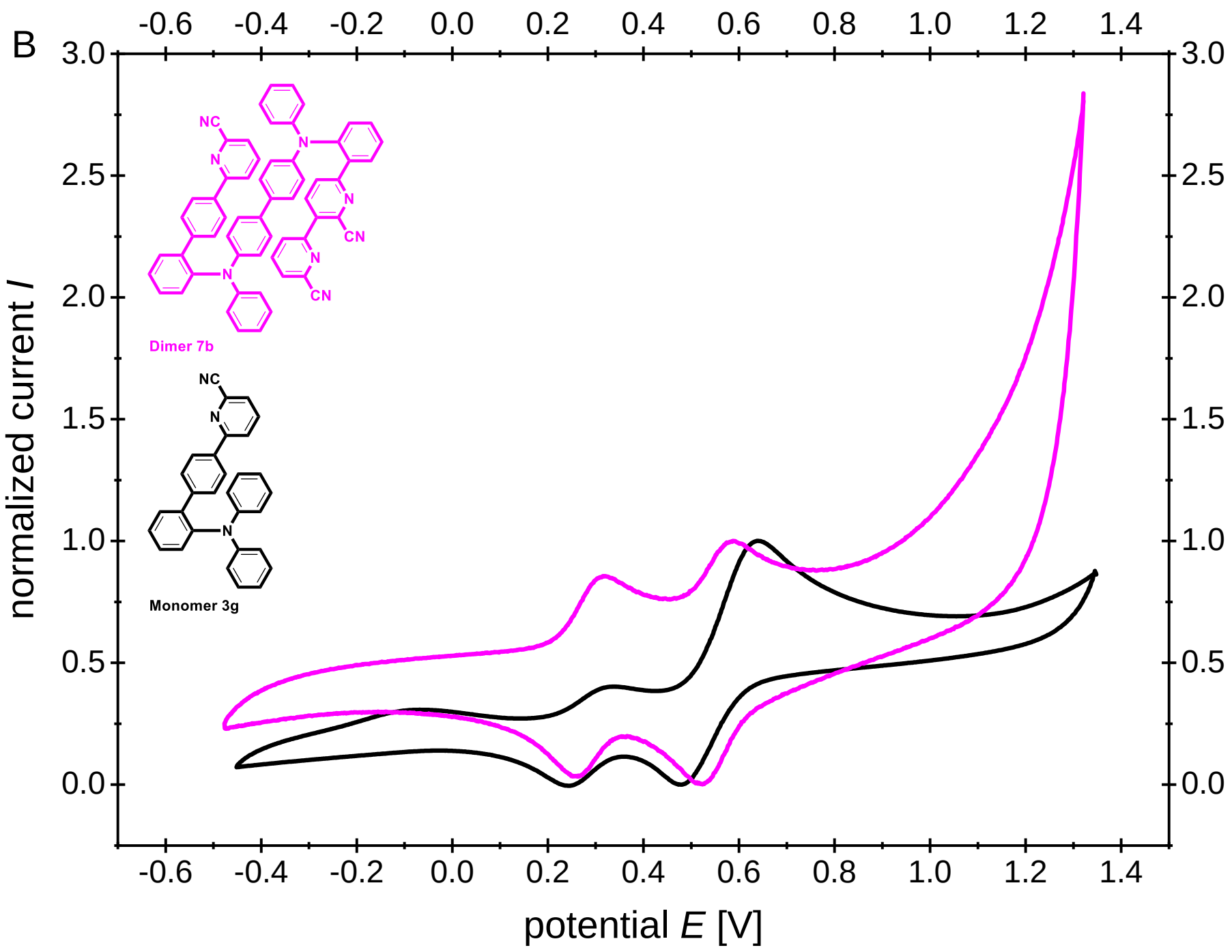

Figure S50. Cyclic voltammograms of *sym-o*-(bTAA)$_2$ **7** compared with the cyclic voltammograms of their monomers *sym-o*-bTAA **3** (A: **7a** and **3f**, B: **7b** and **3g**) recorded in dichloromethane (T = 293 K, ν = 100 mV/s, electrolyte: [^{n}Bu$_4$N][PF$_6$], Pt working electrode, Pt counter electrode, Ag/AgCl reference electrode, redox standard: decamethylferrocene $E_0^{0/+1}$ = -540 mV (vs. ferrocene $E_0^{0/+1}$ = 0.00 mV)[8]).

5.3 Cyclic voltammogram of *para-/meta-/ortho*-triarylamine dimers (*sym-p-/m-/o*-bTAA)$_2$ 4, 5, 6 and 7

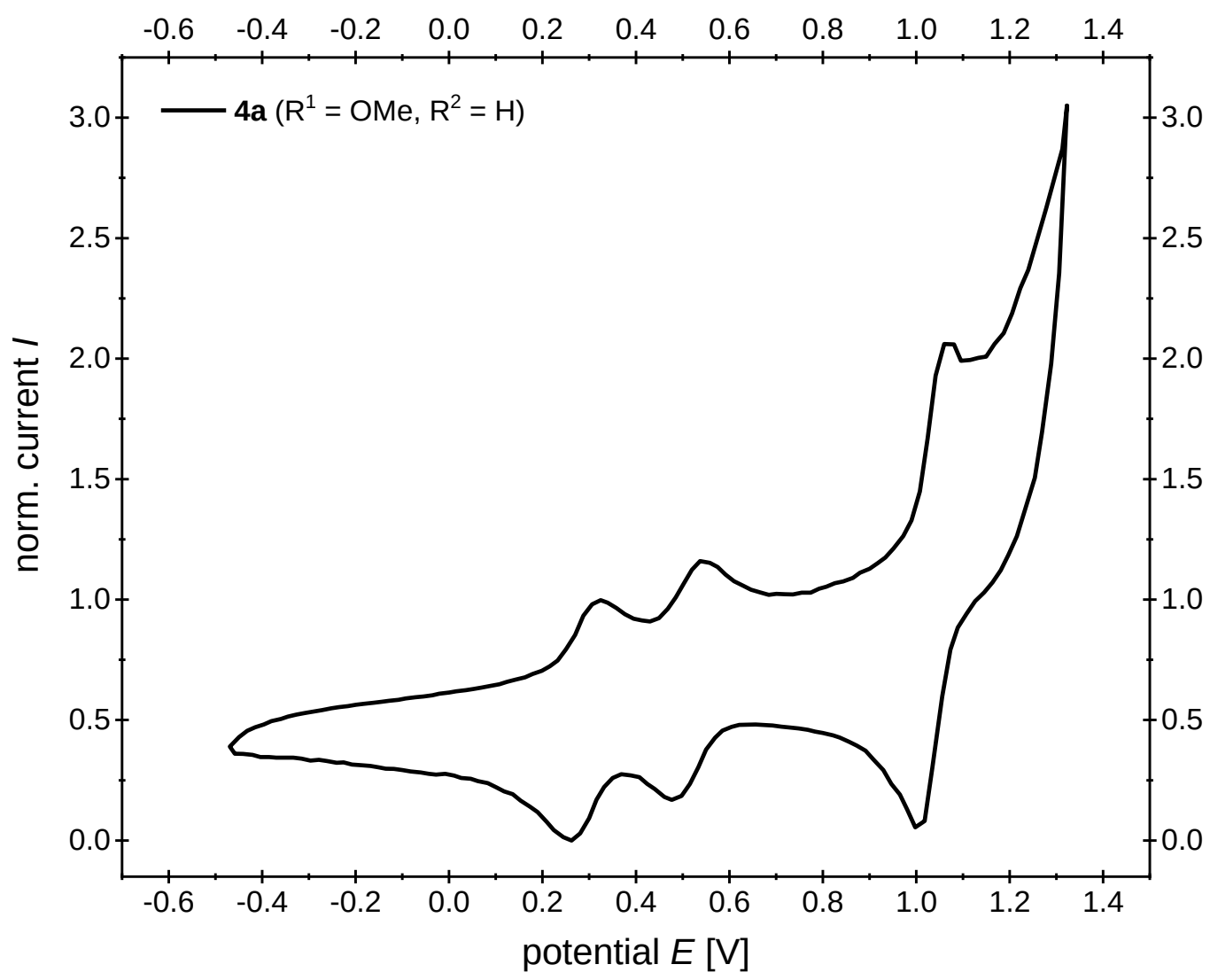

Figure S51. Cyclic voltammogram of compound **4a** recorded in dichloromethane (T = 293 K, ν = 100 mV/s, electrolyte: [^{n}Bu$_4$N][PF$_6$], Pt working electrode, Pt counter electrode, Ag/AgCl reference electrode, redox standard: decamethylferrocene $E_0^{0/+1}$ = -540 mV (vs. ferrocene $E_0^{0/+1}$ = 0.00 mV)[8]).

Compound	$E_0^{0/+1}$ [V]	$E_0^{+1/+2}$ [V]	$E_0^{+2/+3}$ [V]
**4a**	0.29	0.51	1.03

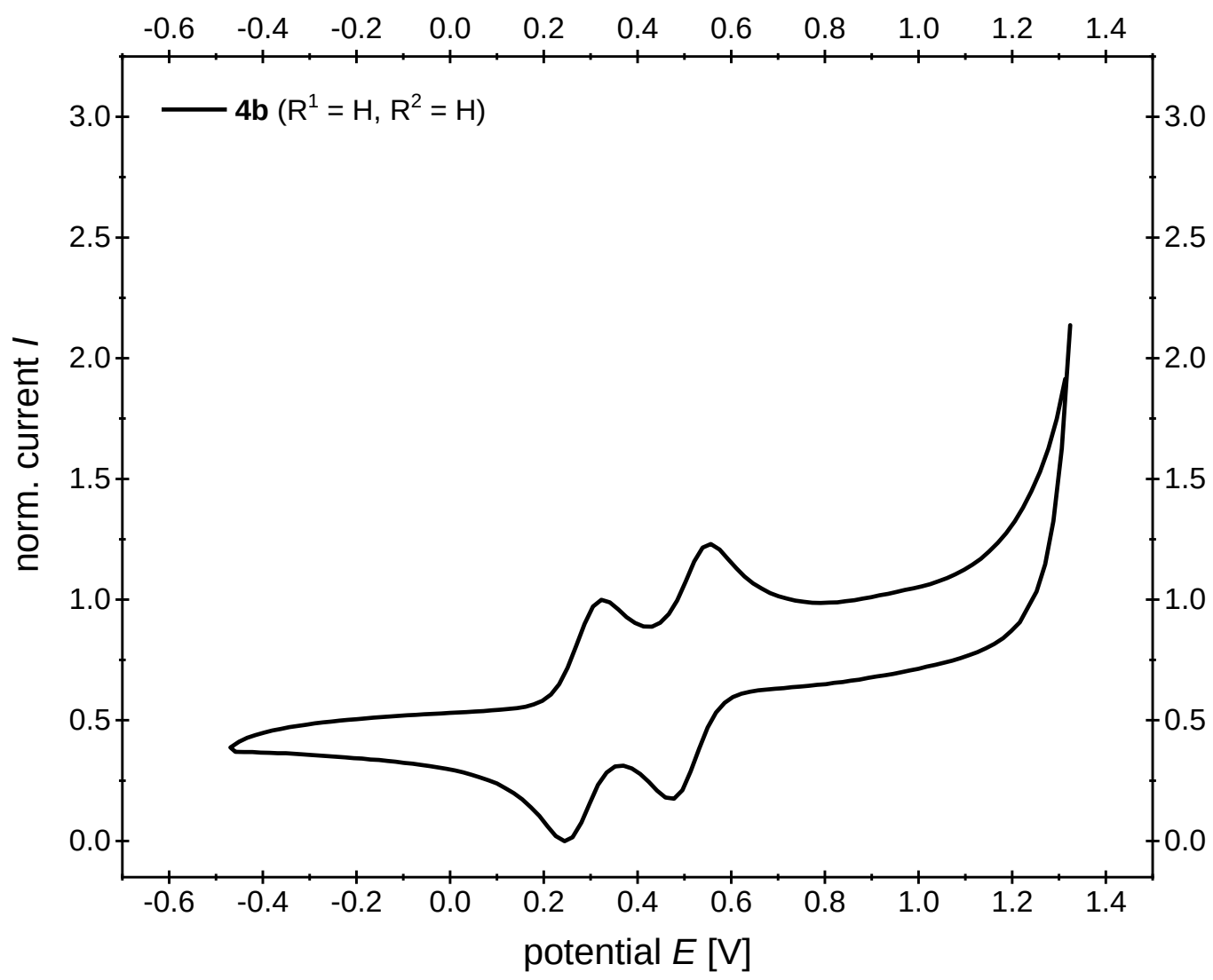

Figure S52. Cyclic voltammogram of compound **4b** recorded in dichloromethane (T = 293 K, ν = 100 mV/s, electrolyte: [^{n}Bu$_4$N][PF$_6$], Pt working electrode, Pt counter electrode, Ag/AgCl reference electrode, redox standard: decamethylferrocene $E_0^{0/+1}$ = -540 mV (vs. ferrocene $E_0^{0/+1}$ = 0.00 mV)[8]).

Compound	$E_0^{0/+1}$ [V]	$E_0^{+1/+2}$ [V]
**4b**	0.28	0.51

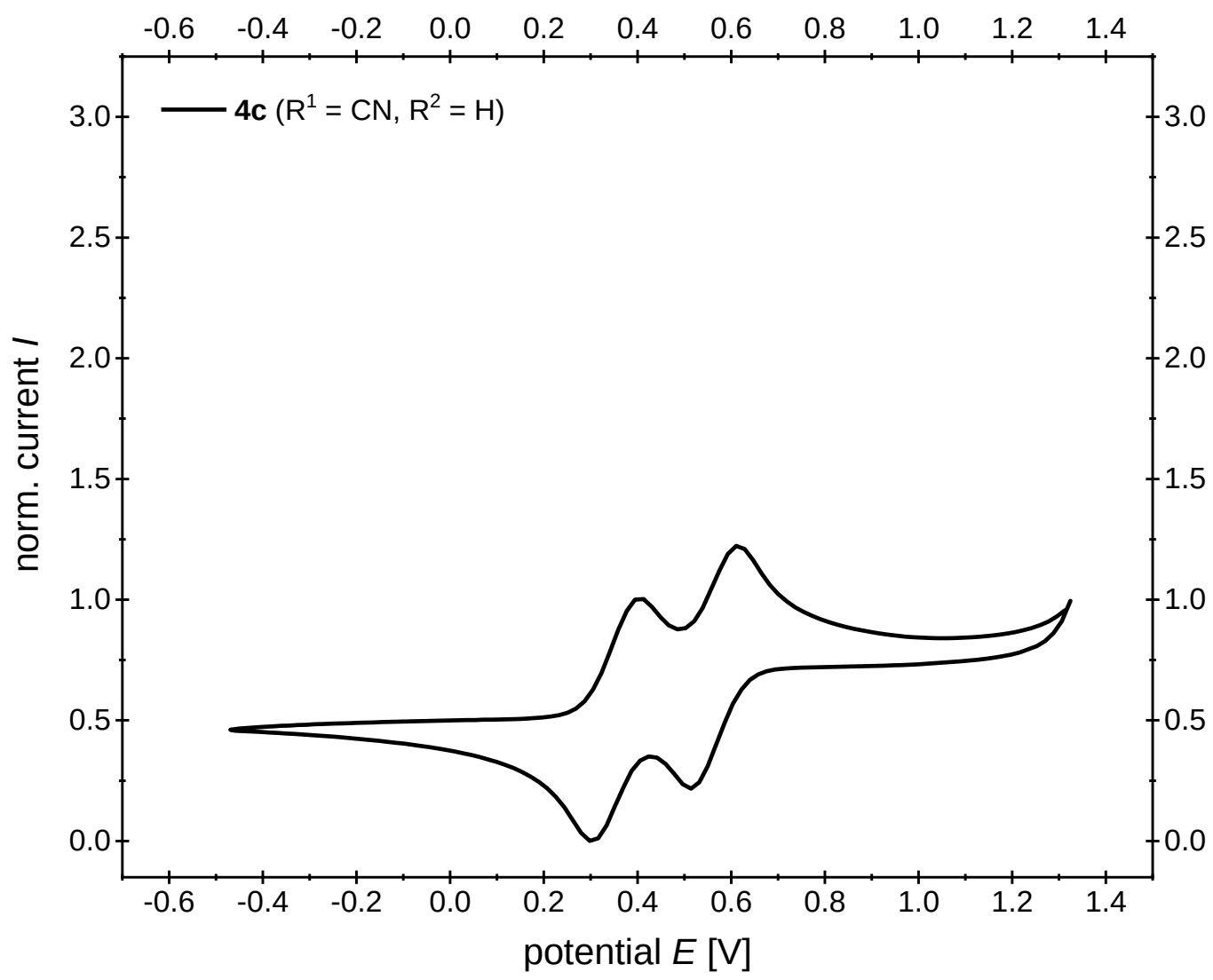

Figure S53. Cyclic voltammogram of compound **4c** recorded in dichloromethane (T = 293 K, ν = 100 mV/s, electrolyte: [^{n}Bu$_4$N][PF$_6$], Pt working electrode, Pt counter electrode, Ag/AgCl reference electrode, redox standard: decamethylferrocene $E_0^{0/+1}$ = -540 mV (vs. ferrocene $E_0^{0/+1}$ = 0.00 mV)[8]).

Compound	$E_0^{0/+1}$ [V]	$E_0^{+1/+2}$ [V]
**4c**	0.36	0.56

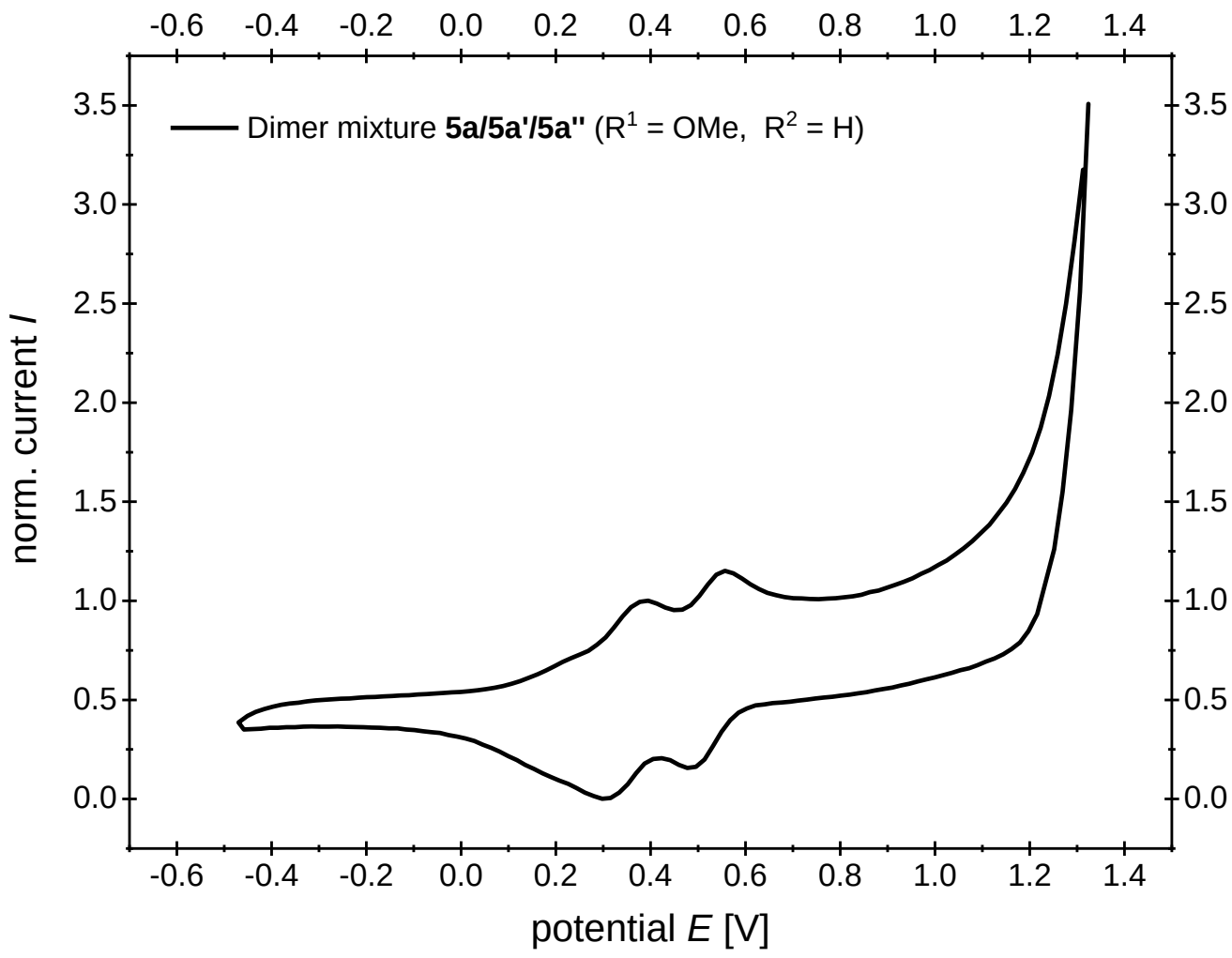

Figure S54. Cyclic voltammogram of compound mixture **5a/5a'/5a''** recorded in dichloromethane (T = 293 K, ν = 100 mV/s, electrolyte: [^{n}Bu$_4$N][PF$_6$], Pt working electrode, Pt counter electrode, Ag/AgCl reference electrode, redox standard: decamethylferrocene $E_0^{0/+1}$ = -540 mV (vs. ferrocene $E_0^{0/+1}$ = 0.00 mV)[8]).

Compound mixture	$E_0^{0/+1}$ [V]	$E_0^{+1/+2}$ [V]	K_{SEM}
**5a/5a'/5a''**	0.35	0.52	$6.47 \cdot 10^2$

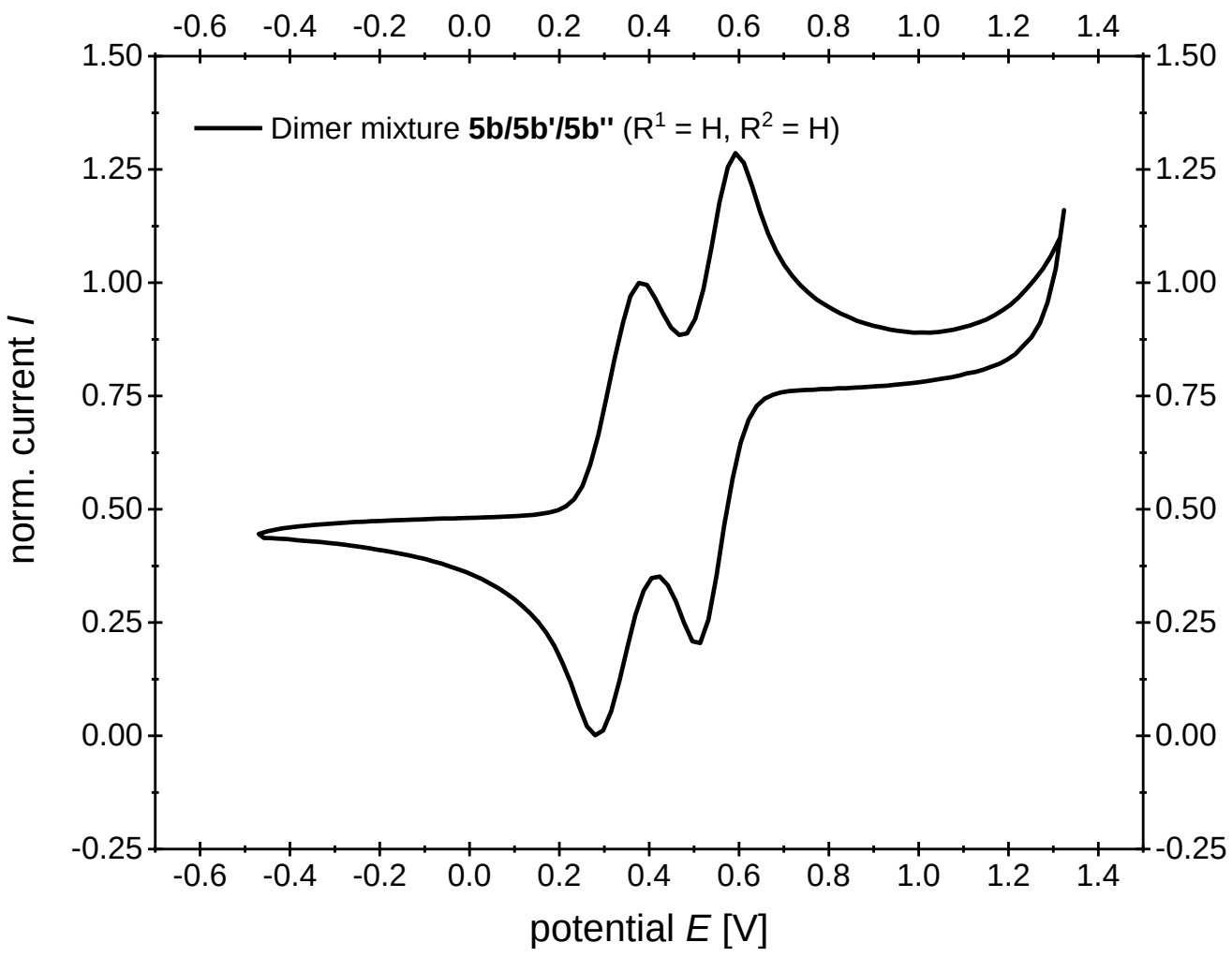

Figure S55. Cyclic voltammogram of compound mixture **5b/5b'/5b''** recorded in dichloromethane (T = 293 K, ν = 100 mV/s, electrolyte: [^{n}Bu$_4$N][PF$_6$], Pt working electrode, Pt counter electrode, Ag/AgCl reference electrode, redox standard: decamethylferrocene $E_0^{0/+1}$ = -540 mV (vs. ferrocene $E_0^{0/+1}$ = 0.00 mV)[8]).

Compound mixture	$E_0^{0/+1}$ [V]	$E_0^{+1/+2}$ [V]	K_{SEM}
**5b/5b'/5b''**	0.36	0.55	$2.02 \cdot 10^3$

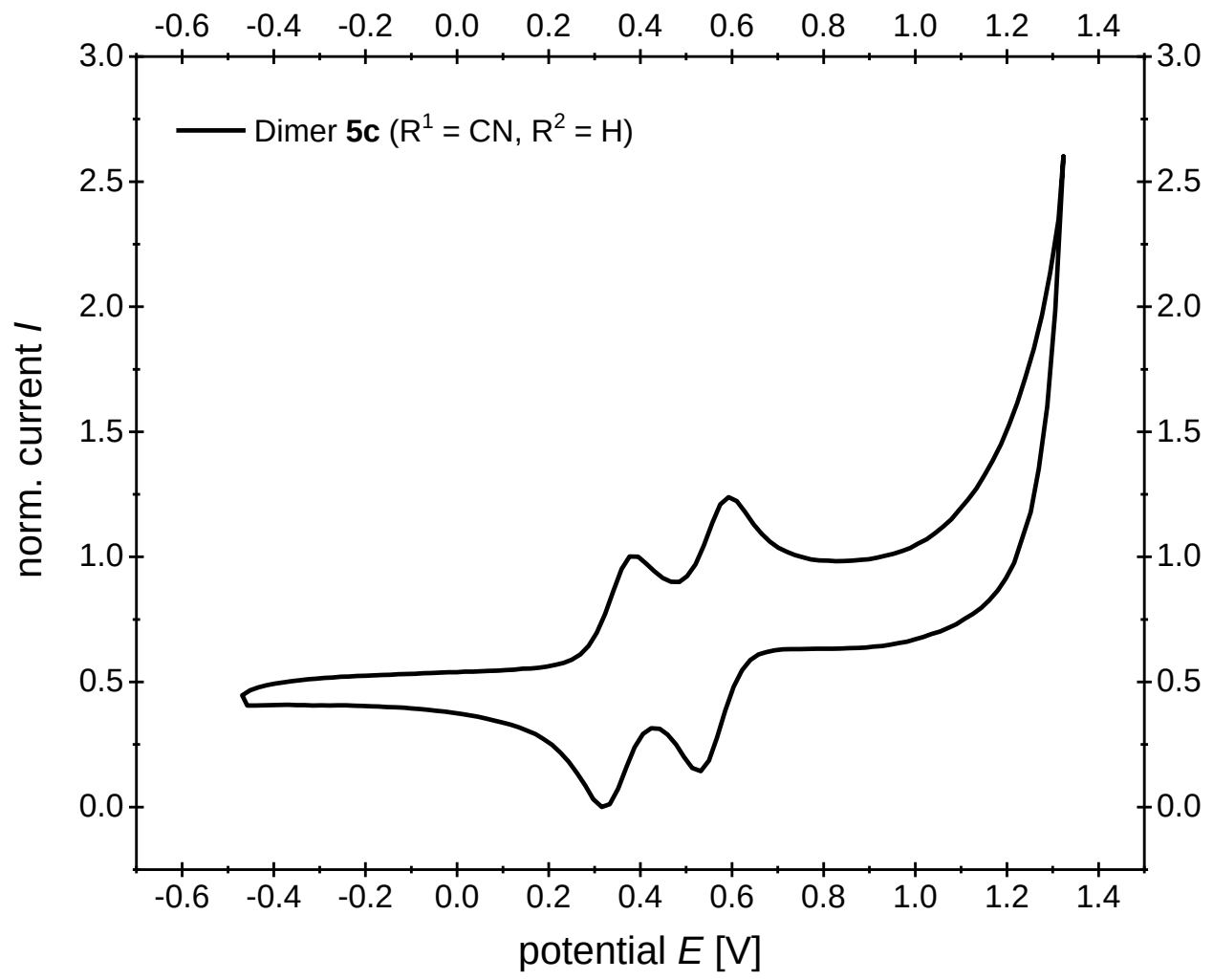

Figure S56. Cyclic voltammogram of compound **5c** recorded in dichloromethane (T = 293 K, ν = 100 mV/s, electrolyte: [^{n}Bu$_4$N][PF$_6$], Pt working electrode, Pt counter electrode, Ag/AgCl reference electrode, redox standard: decamethylferrocene $E_0^{0/+1}$ = -540 mV (vs. ferrocene $E_0^{0/+1}$ = 0.00 mV)[8]).

Compound	$E_0^{0/+1}$ [V]	$E_0^{+1/+2}$ [V]
**5c**	0.36	0.56

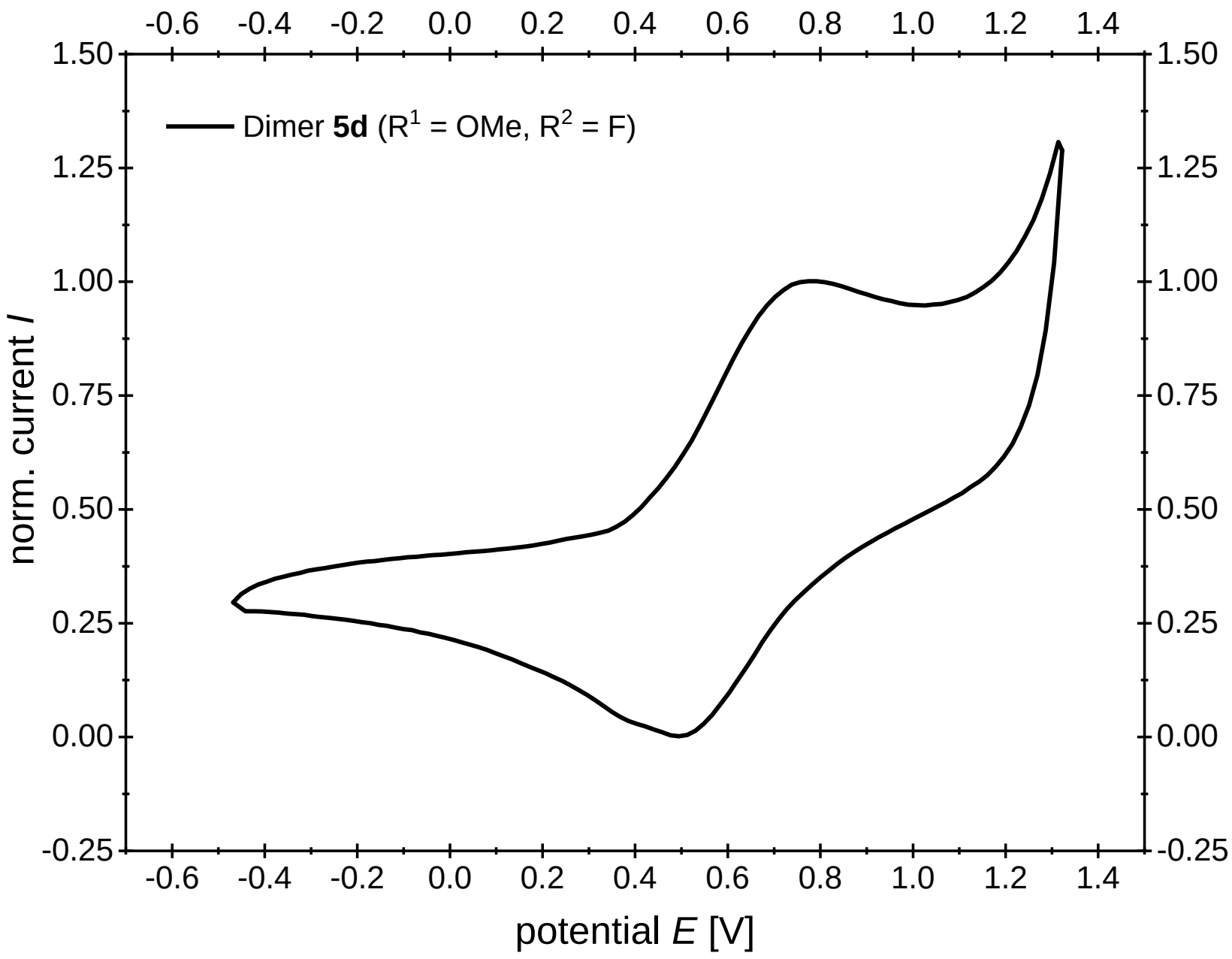

Figure S57. Cyclic voltammogram of compound **5d** recorded in dichloromethane (T = 293 K, ν = 100 mV/s, electrolyte: [^{n}Bu$_4$N][PF$_6$], Pt working electrode, Pt counter electrode, Ag/AgCl reference electrode, redox standard: decamethylferrocene $E_0^{0/+1}$ = -540 mV (vs. ferrocene $E_0^{0/+1}$ = 0.00 mV)[8]).

Compoun d	$E_0^{0/+1}$ [V]
**5d**	0.62

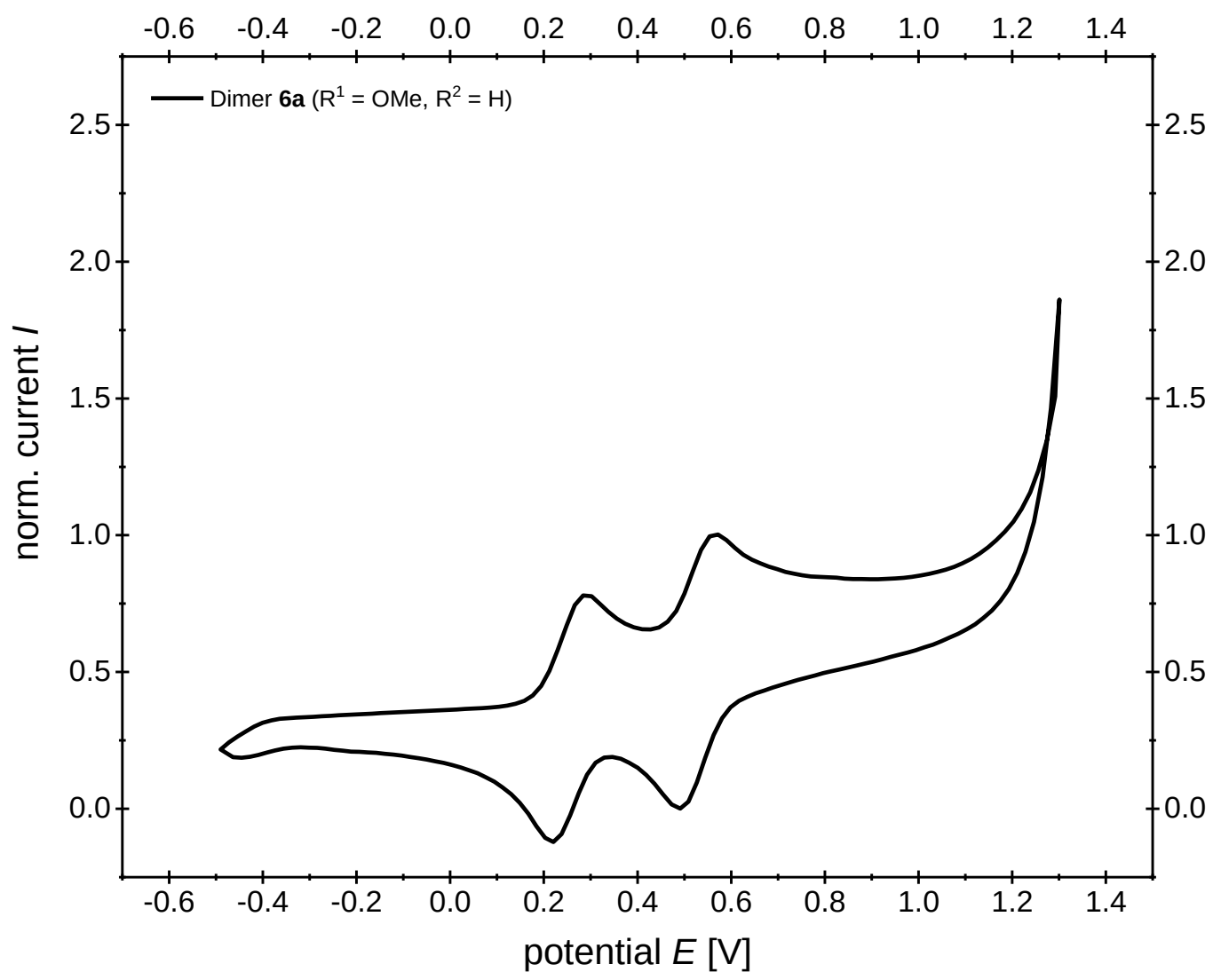

Figure S58. Cyclic voltammogram of compound **6a** recorded in dichloromethane (T = 293 K, ν = 100 mV/s, electrolyte: [$^{n}Bu_4N$][PF_6], Pt working electrode, Pt counter electrode, Ag/AgCl reference electrode, redox standard: decamethylferrocene $E_0^{0/+1}$ = -540 mV (vs. ferrocene $E_0^{0/+1}$ = 0.00 mV)[8]).

Compound	$E_0^{0/+1}$ [V]	$E_0^{+1/+2}$ [V]
**6a**	0.24	0.55

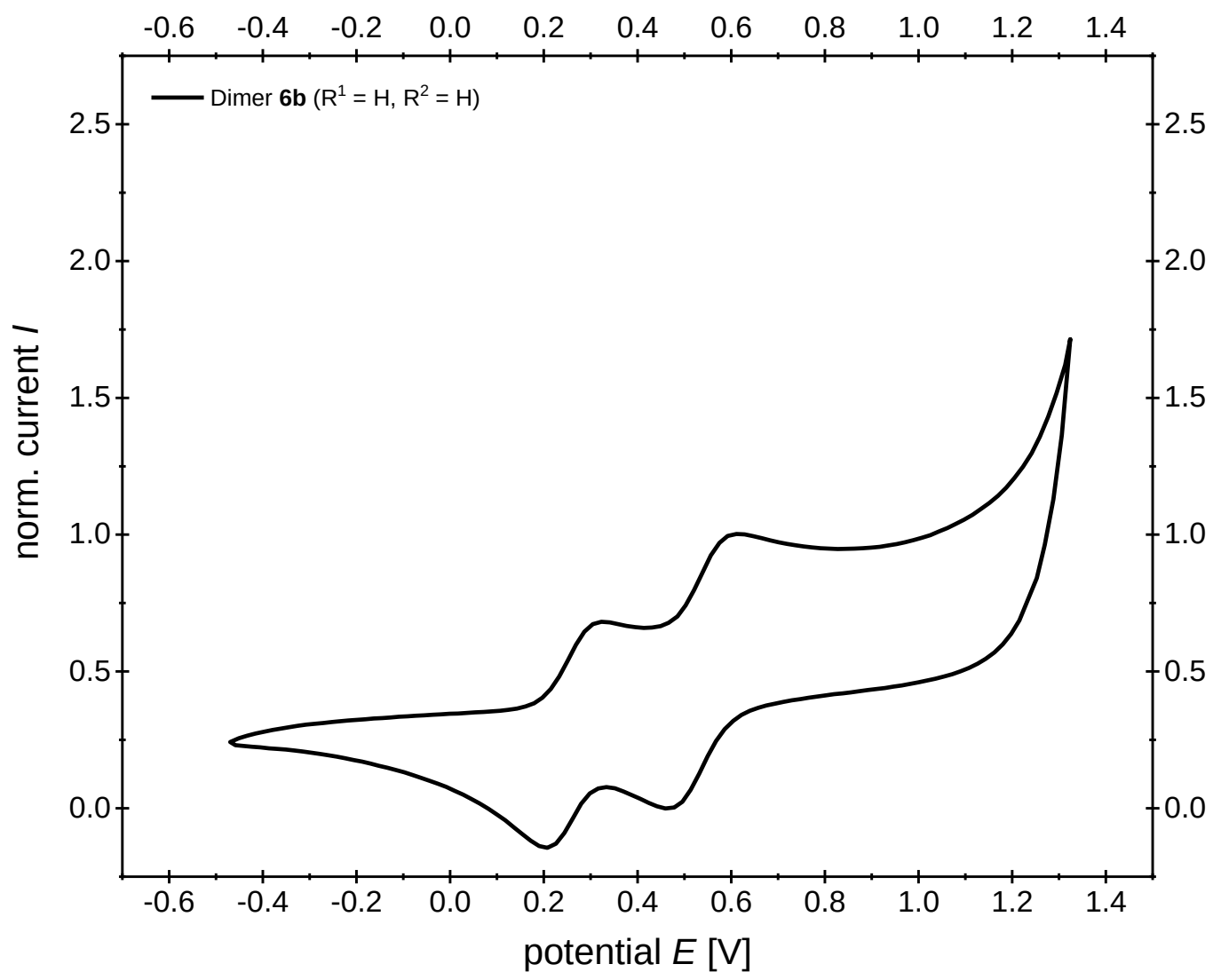

Figure S59. Cyclic voltammogram of compound **6b** recorded in dichloromethane (T = 293 K, ν = 100 mV/s, electrolyte: [$^{n}Bu_4N$][PF_6], Pt working electrode, Pt counter electrode, Ag/AgCl reference electrode, redox standard: decamethylferrocene $E_0^{0/+1}$ = -540 mV (vs. ferrocene $E_0^{0/+1}$ = 0.00 mV)[8]).

Compound	$E_0^{0/+1}$ [V]	$E_0^{+1/+2}$ [V]
**6b**	0.29	0.54

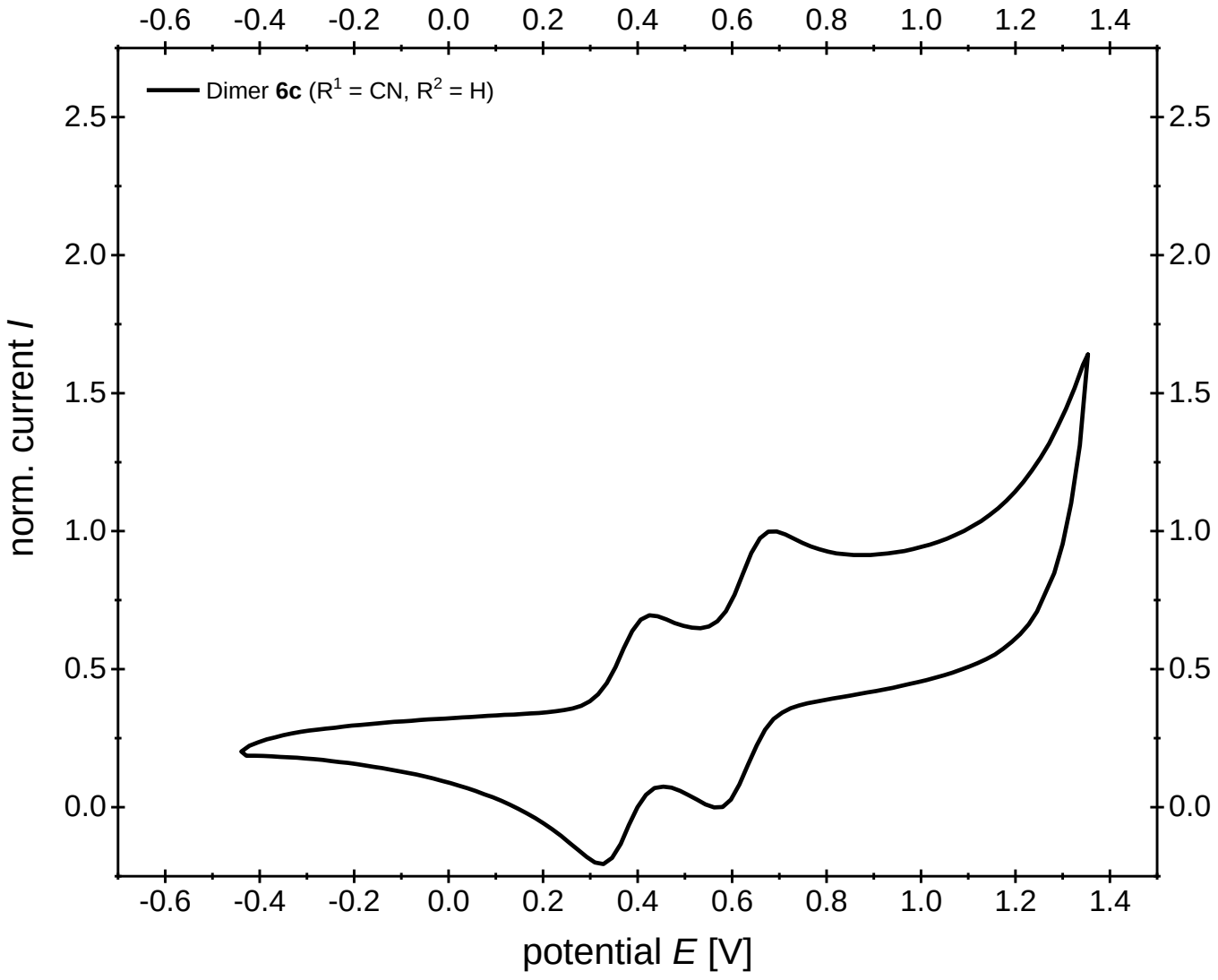

Figure S60. Cyclic voltammogram of compound **6c** recorded in dichloromethane (T = 293 K, ν = 100 mV/s, electrolyte: [^{n}Bu$_4$N][PF$_6$], Pt working electrode, Pt counter electrode, Ag/AgCl reference electrode, redox standard: decamethylferrocene $E_0^{0/+1}$ = -540 mV (vs. ferrocene $E_0^{0/+1}$ = 0.00 mV)[8]).

Compound	$E_0^{0/+1}$ [V]	$E_0^{+1/+2}$ [V]
**6c**	0.34	0.60

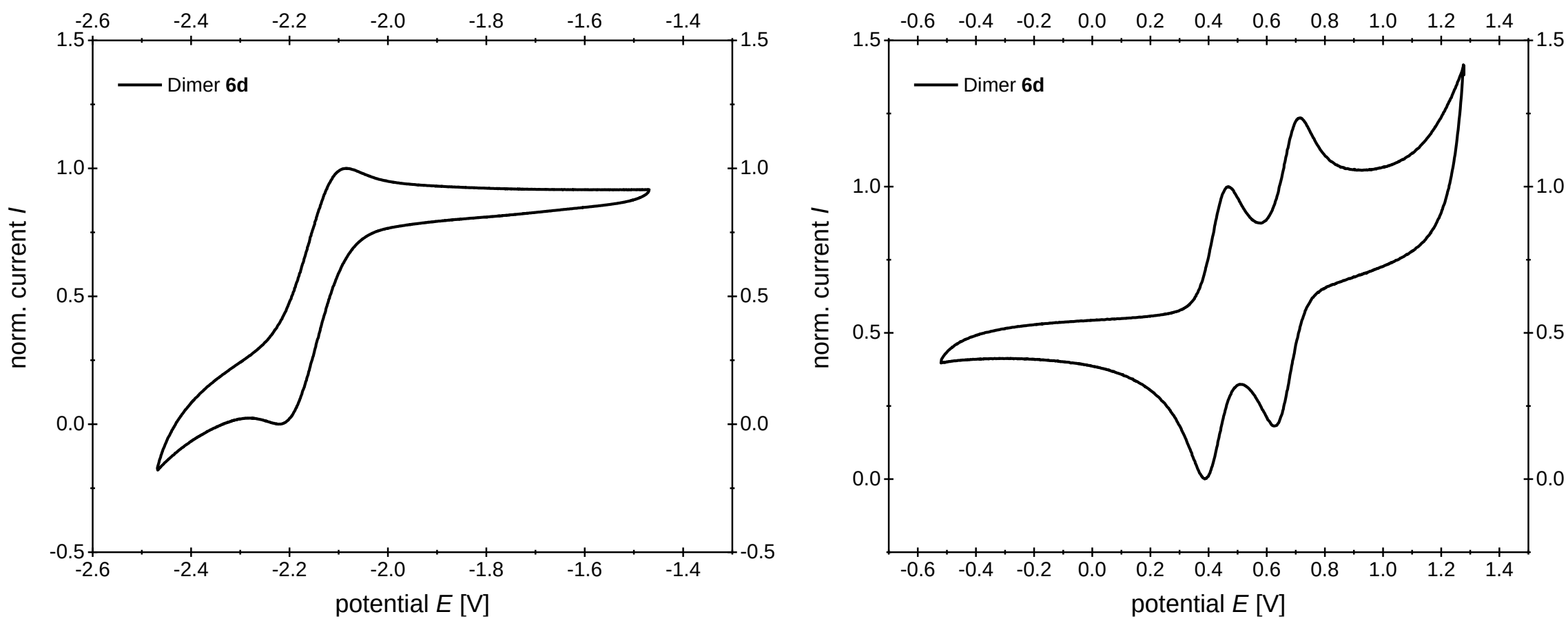

Figure S61. Cyclic voltammogram of compound **6d** recorded in dichloromethane (T = 293 K, ν = 100 mV/s, electrolyte: [^{n}Bu$_4$N][PF$_6$], Pt working electrode, Pt counter electrode, Ag/AgCl reference electrode, redox standard: decamethylferrocene $E_0^{0/+1}$ = -540 mV (vs. ferrocene $E_0^{0/+1}$ = 0.00 mV)[8]).

Compound	$E_0^{-1/0}$ [V]	$E_0^{0/+1}$ [V]	$E_0^{+1/+2}$ [V]
**6d**	-2.15	0.43	0.67

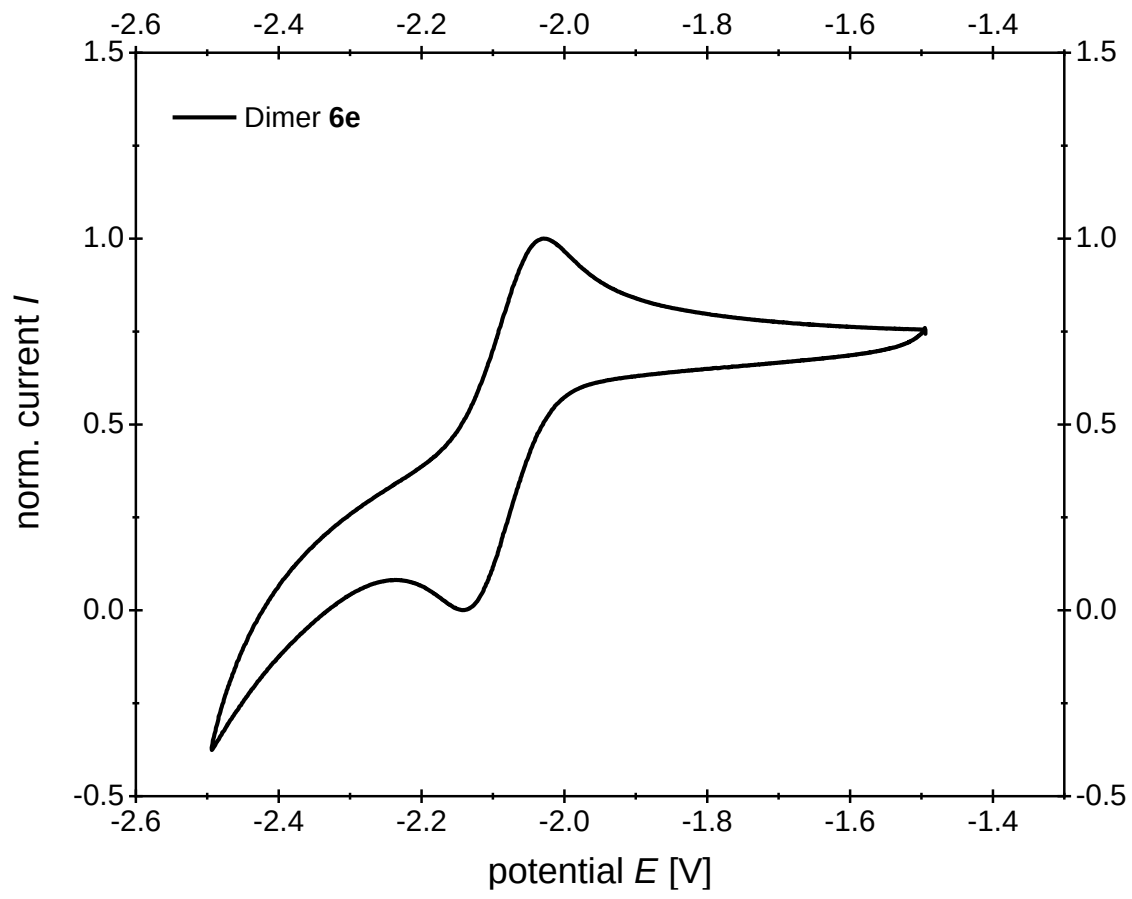

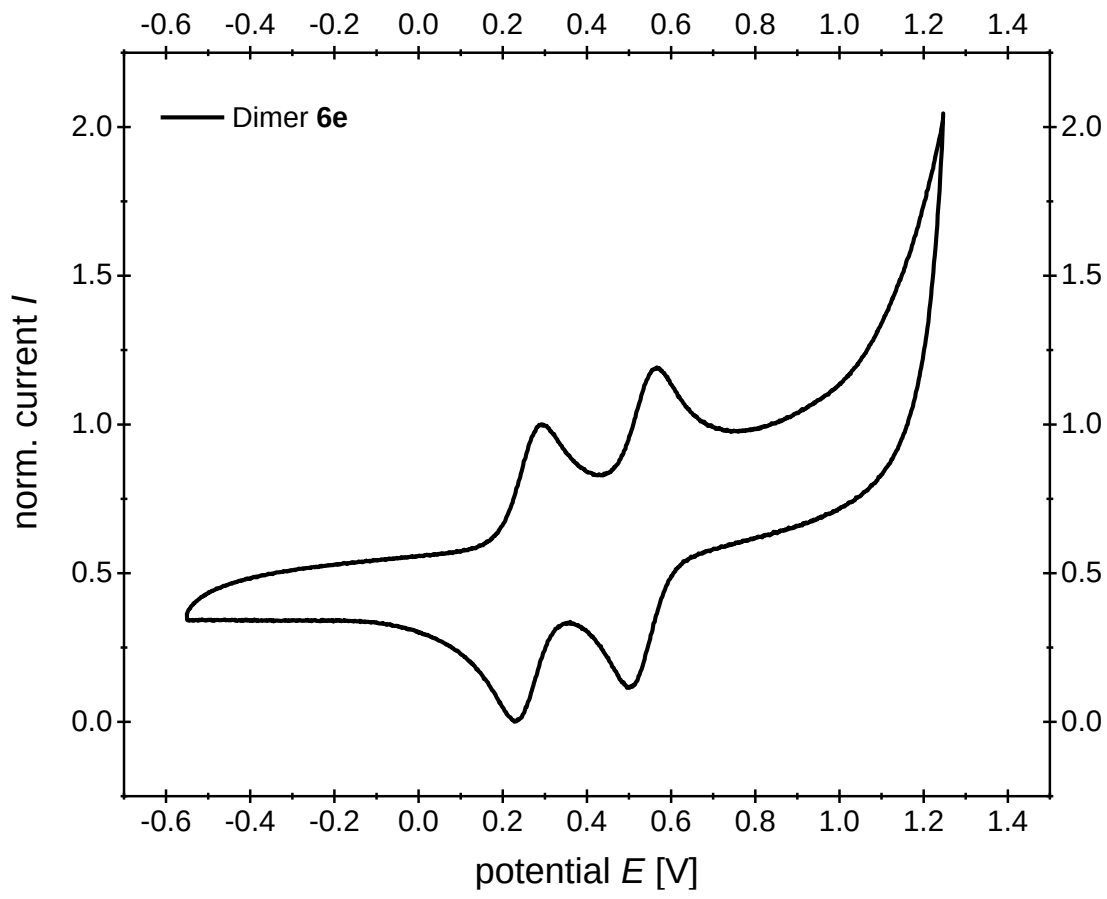

Figure S62. Cyclic voltammogram of compound **6e** recorded in dichloromethane (T = 293 K, ν = 100 mV/s, electrolyte: [$^{n}Bu_4N$][PF_6], Pt working electrode, Pt counter electrode, Ag/AgCl reference electrode, redox standard: decamethylferrocene $E_0^{0/+1}$ = -540 mV (vs. ferrocene $E_0^{0/+1}$ = 0.00 mV)[8]).

Compound	$E_0^{-1/0}$ [V]	$E_0^{0/+1}$ [V]	$E_0^{+1/+2}$ [V]
**6e**	-2.08	0.26	0.53

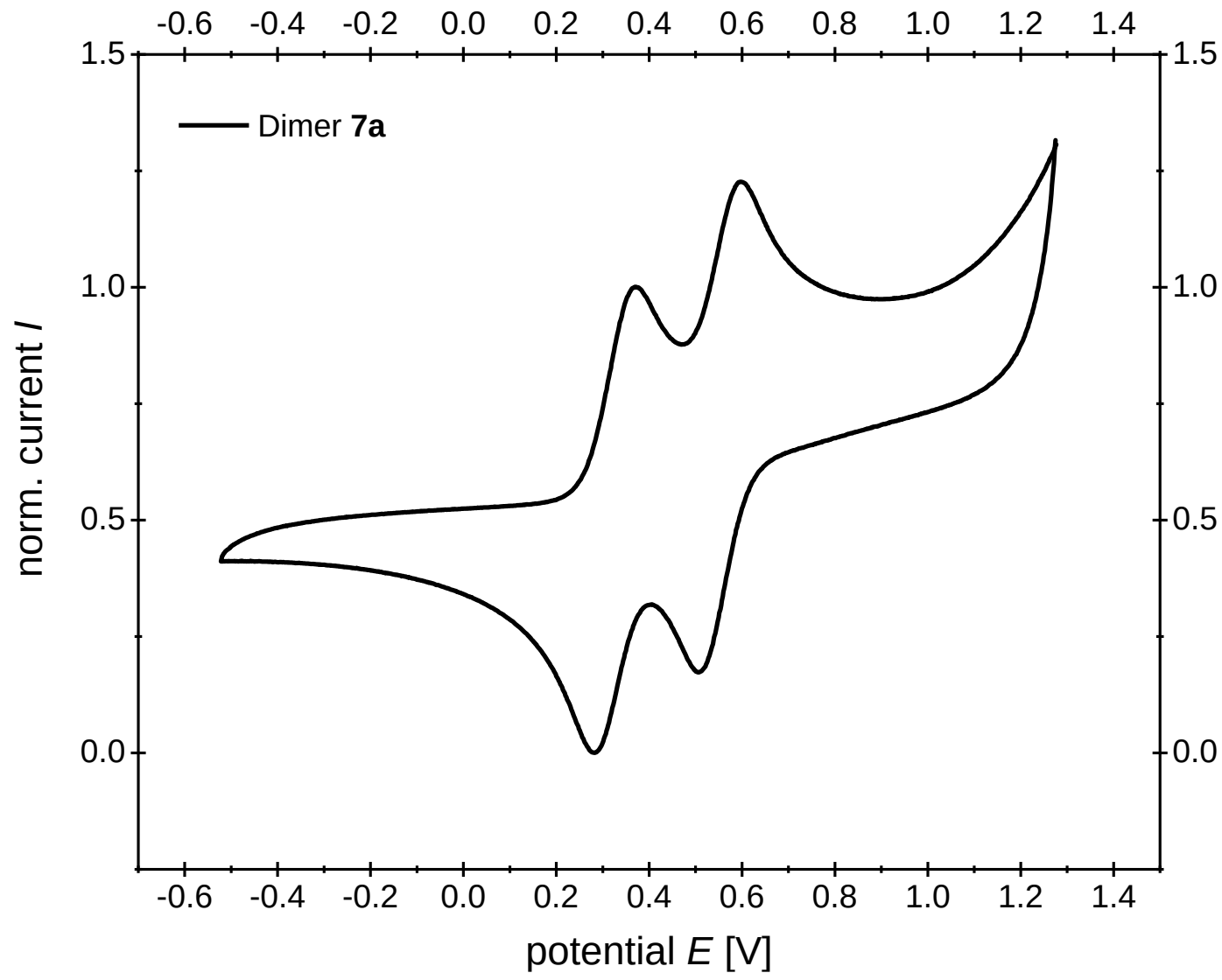

Figure S63. Cyclic voltammogram of compound **7a** recorded in dichloromethane (T = 293 K, ν = 100 mV/s, electrolyte: [$^{n}Bu_4N$][PF_6], Pt working electrode, Pt counter electrode, Ag/AgCl reference electrode, redox standard: decamethylferrocene $E_0^{0/+1}$ = -540 mV (vs. ferrocene $E_0^{0/+1}$ = 0.00 mV)[8]).

Compound	$E_0^{0/+1}$ [V]	$E_0^{+1/+2}$ [V]
**7a**	0.33	0.55

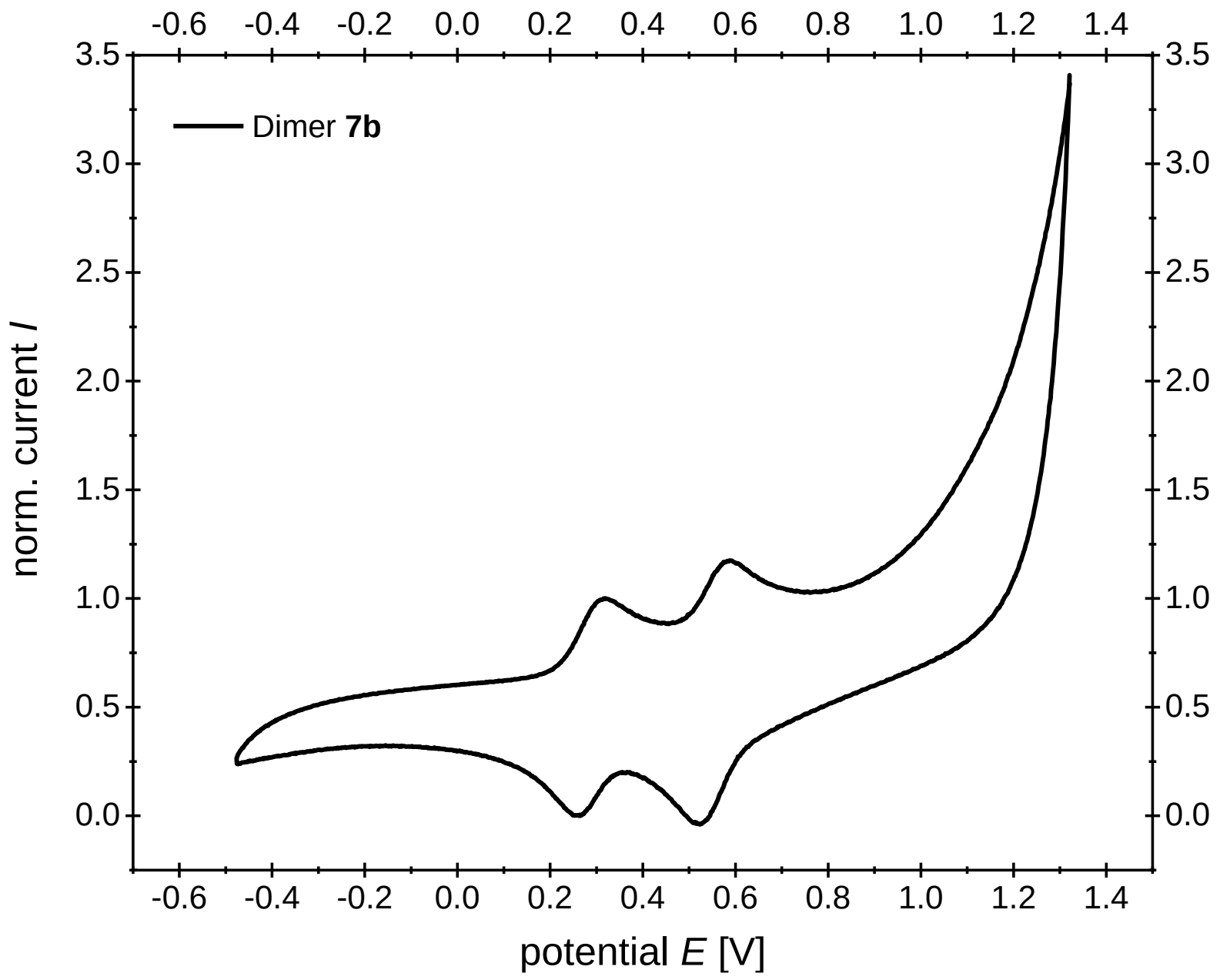

Figure S64. Cyclic voltammogram of compound **7b** recorded in dichloromethane (T = 293 K, ν = 100 mV/s, electrolyte: [^{n}Bu$_4$N][PF$_6$], Pt working electrode, Pt counter electrode, Ag/AgCl reference electrode, redox standard: decamethylferrocene $E_0^{0/+1}$ = -540 mV (vs. ferrocene $E_0^{0/+1}$ = 0.00 mV)[8]).

Compound	$E_0^{0/+1}$ [V]	$E_0^{+1/+2}$ [V]
**7b**	0.29	0.55

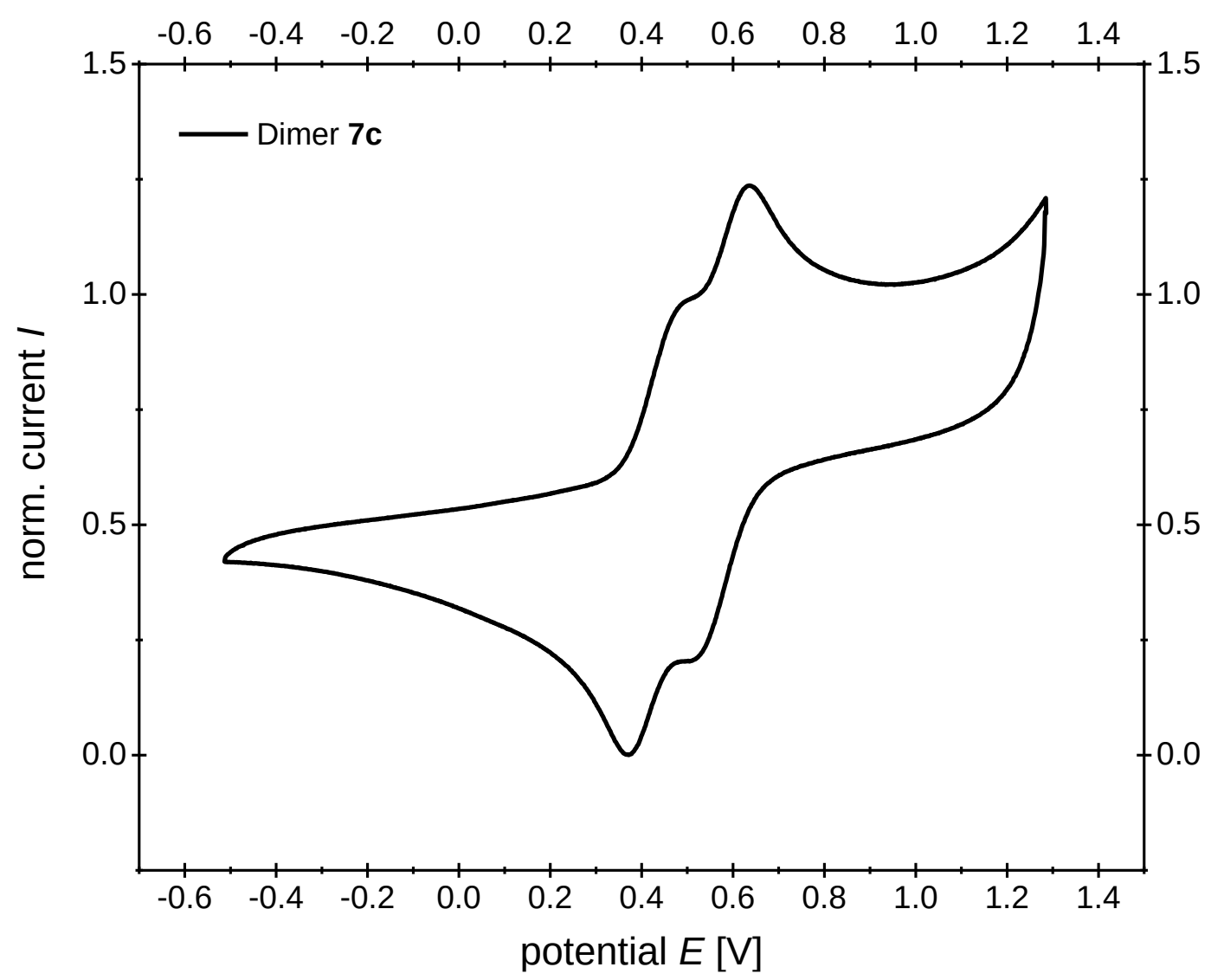

Figure S65. Cyclic voltammogram of compound **7c** recorded in dichloromethane (T = 293 K, ν = 100 mV/s, electrolyte: [^{n}Bu$_4$N][PF$_6$], Pt working electrode, Pt counter electrode, Ag/AgCl reference electrode, redox standard: decamethylferrocene $E_0^{0/+1}$ = -540 mV (vs. ferrocene $E_0^{0/+1}$ = 0.00 mV)[8]).

Compound	$E_0^{0/+1}$ [V]	$E_0^{+1/+2}$ [V]
**7c**	0.44	0.59

5.4 Electrochemical reversibility

The electrochemical reversibility of the dimers **4–7** is evaluated by comparison with the established redox standards decamethylferrocene (**DMFc**) and ferrocene (**Fc**).[9,10] Cyclic voltammograms of the standards are recorded at different scan rates of 100, 250, 500 and 1000 mV/s (Figure S66). To account for chemical reversibility, the difference of peak potentials ΔE_p at different scan rates ν is plotted against the square root of the scan rate $\nu^{1/2}$ (Figure S67). Linear regression analysis is employed to extrapolate ΔE_p to a scan rate of 0 mV/s corresponding to the y-intercept.[9] For an ideal Nernstian reversible one-electron process, a peak separation of 59 mV (T = 298 K) is expected.[10] However, under the experimental conditions employed herein, the standard redox systems **Fc** and **DMFc** exhibit at our setup extrapolated ΔE_p values of 52 up to 98 mV despite being known by literature to be fully Nernstian reversible.[9,10] Representative plots of two measurements are shown in Figure S67 and display high linearity (r^2 = 0.99–1.00). It is well established, that due to uncompensated solution resistance (ohmic loss) the electrochemical reversibility can appear to be quasi-reversibility.[11] Therefore, strict comparison of the extrapolated ΔE_p values with the ideal value of 59 mV is not appropriate under these experimental conditions. Instead, the reversibility of dimers **4–7** is assessed relative to the reversible redox standards under identical measurements conditions established by literature procedures.[9,10] The samples of compound **4–7** are measured at multiple scan rates in presence of the redox standard allowing direct comparison (exemplified for compound **5c**, Figure S69A). Furthermore, no decrease in peak current is observed upon repeated cycling, indicating the absence of significant electrochemical degradation on the experimental timescale. To account for electrochemical reversibility, the observed ΔE_p values are plotted against the square root of the scan rate $\nu^{1/2}$ and extrapolated to a scan rate of 0 mV/s by linear regression for all compounds (exemplified for compounds **4c**, **5c**, **6c**, **6d**, and **7a**, Figures S68–S72, Table S4–S5). Since peak separations of the redox standards of up to 98 mV are observed and measured in comparison of the samples, dimers with values of < 98 mV fall within the same range and are considered to be electrochemically reversible under the experimental conditions (Table S6).

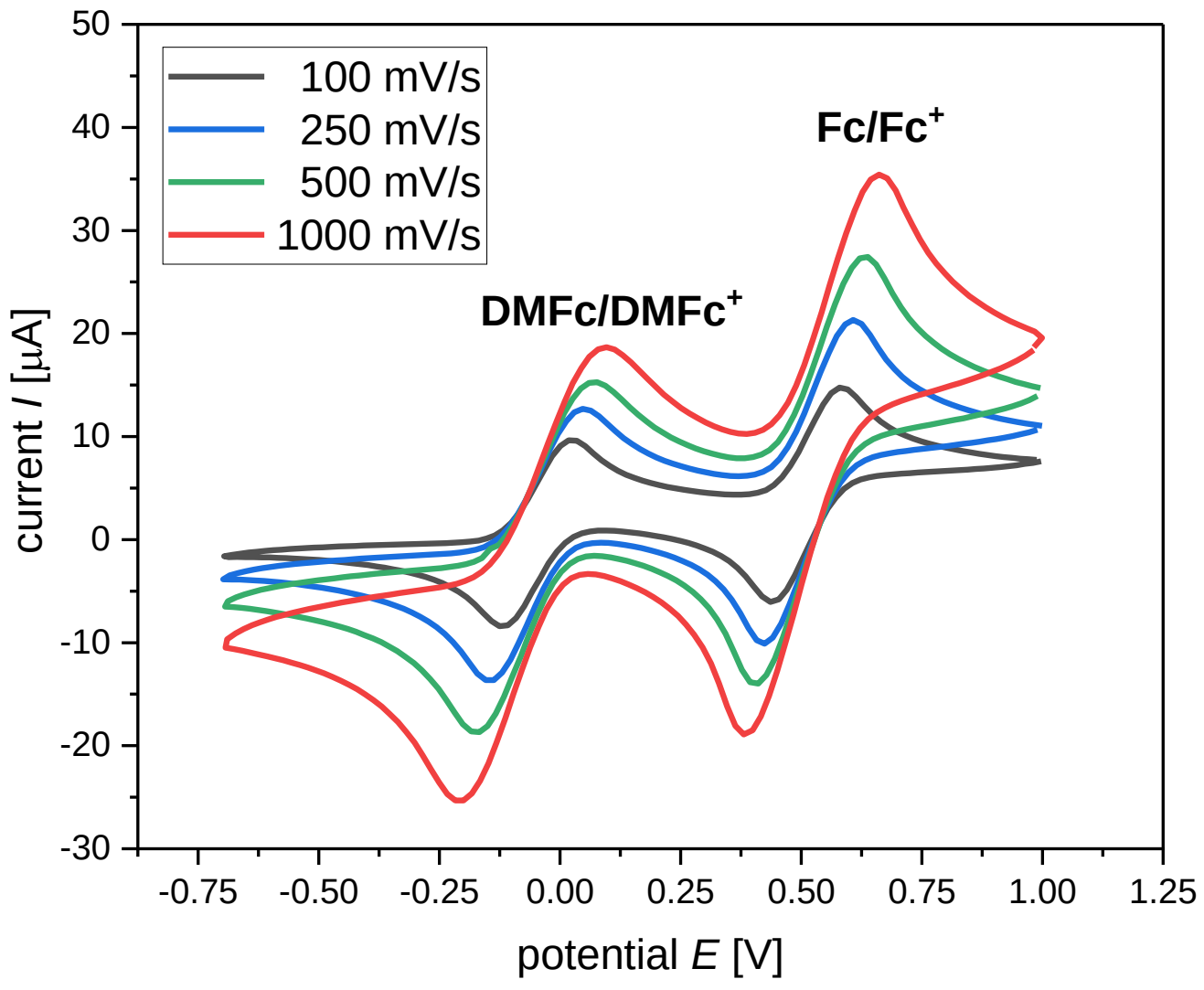

Figure S66. Cyclic voltammogram of the utilized redox standards (**DMFc** and **Fc**) recorded in dichloromethane at different scan rates (T = 293 K, ν = 100–1000 mV/s, electrolyte: [^{n}Bu$_4$N][PF$_6$], Pt working electrode, Pt counter electrode, Ag/AgCl reference electrode.

A

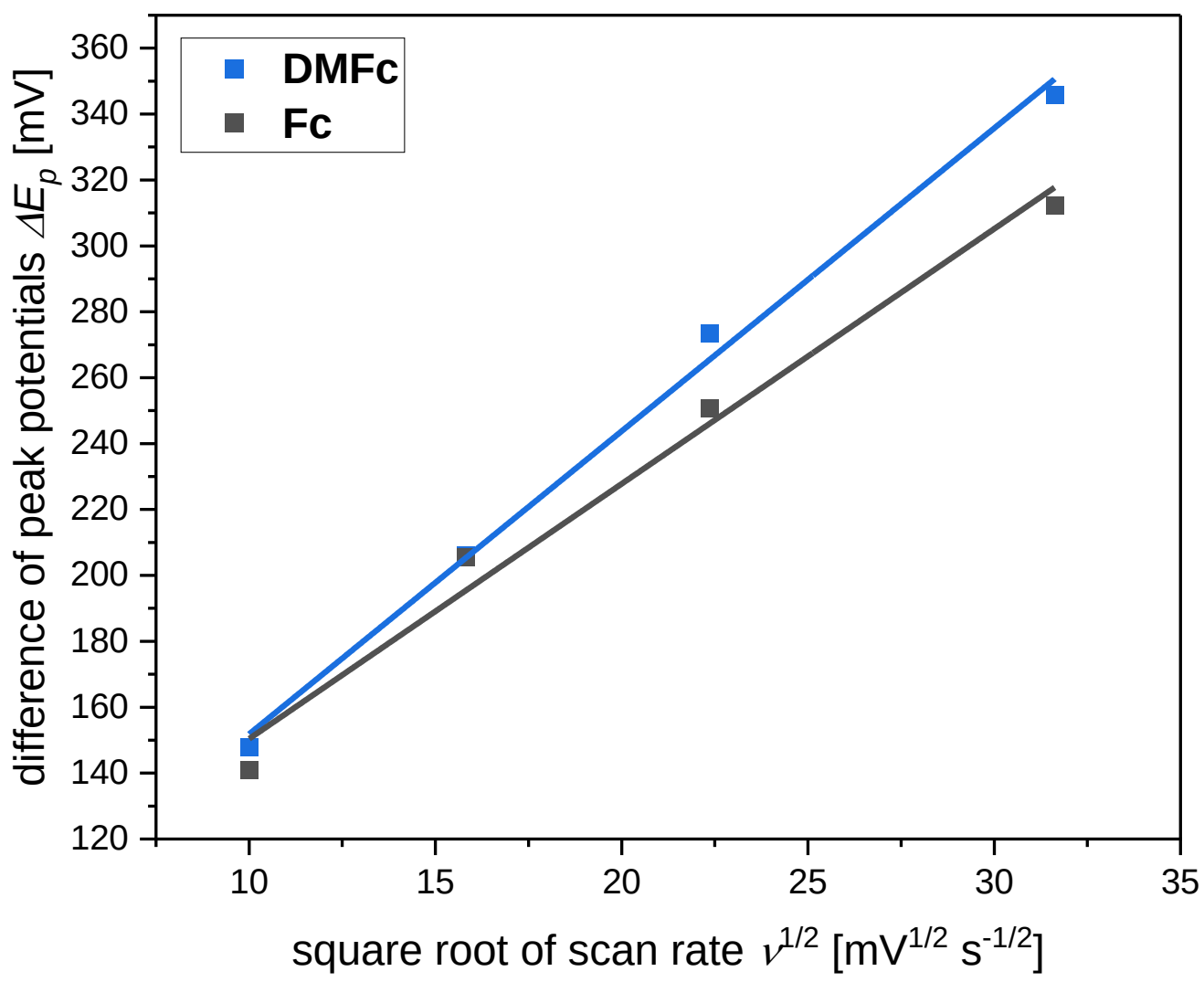

B

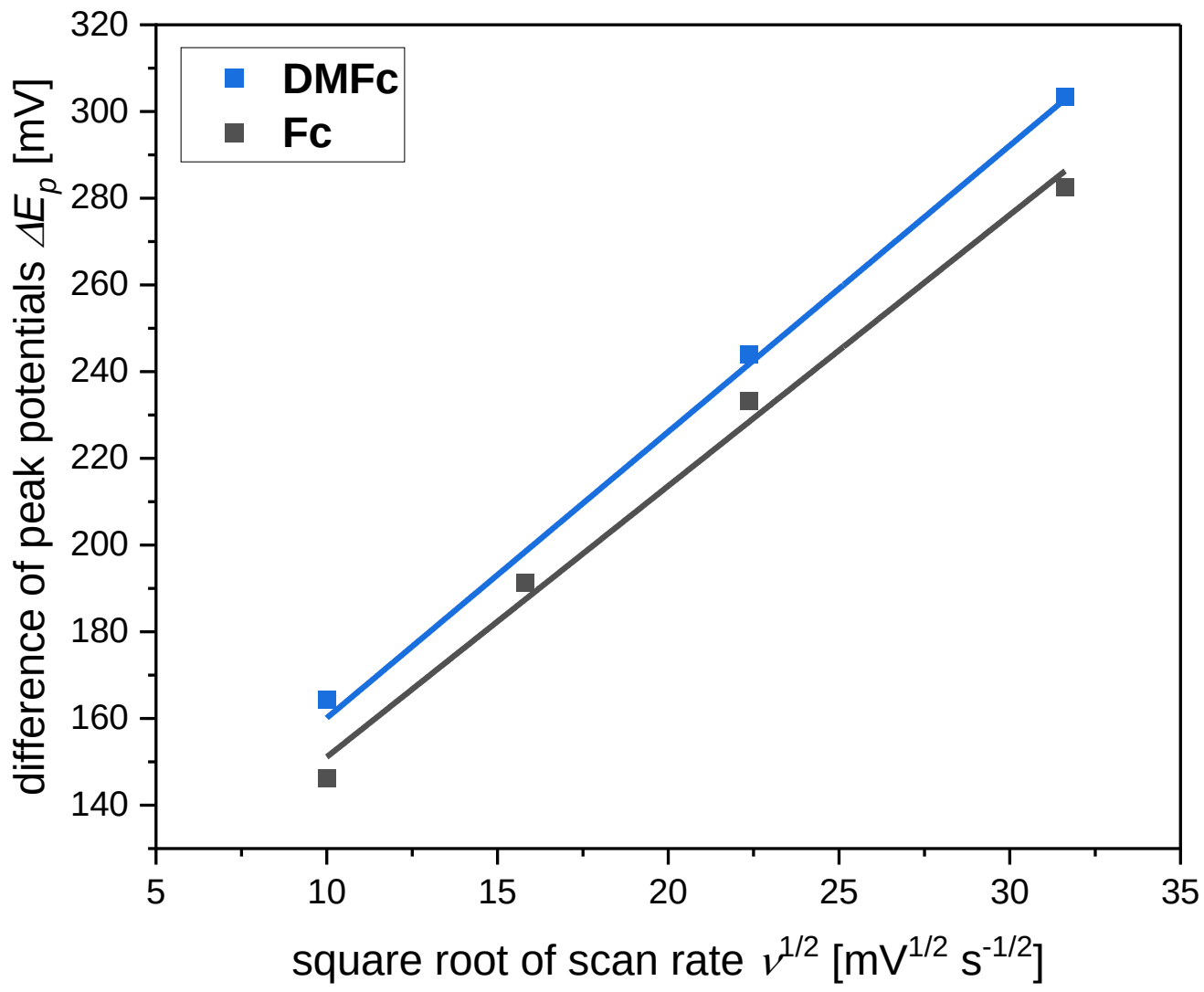

Figure S67. Exemplified difference of peak potentials ΔE_p vs. square root of scan rate $\nu^{1/2}$ (blue line, Decamethylferrocene **DMFc**: (A: ΔE_p = 9.19 mV$^{-1/2}$ s$^{1/2}$ $\nu^{1/2}$ + **59.91 mV** (r^2 = 1.00), B: ΔE_p = 6.60 mV$^{-1/2}$ s$^{1/2}$ $\nu^{1/2}$ + **94.08 mV** (r^2 = 1.00)); black line, Ferrocene **Fc**: (A: ΔE_p = 7.74 mV$^{-1/2}$ s$^{1/2}$ $\nu^{1/2}$ + **73.04 mV** (r^2 = 0.99), B: ΔE_p = 6.25 mV$^{-1/2}$ s$^{1/2}$ $\nu^{1/2}$ + **88.57 mV** (r^2 = 1.00)).

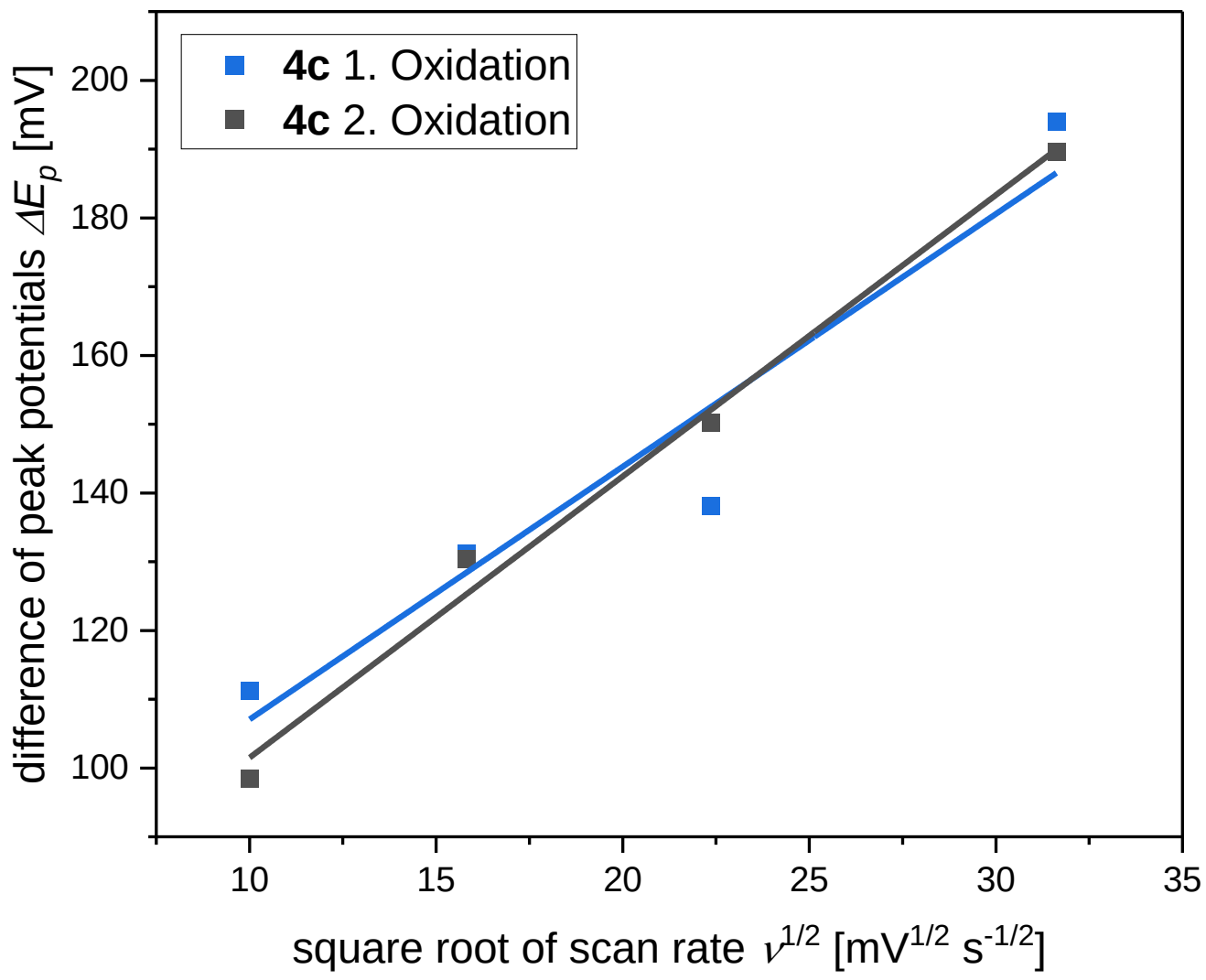

Figure S68. Difference of peak potentials ΔE_p vs. square root of scan rate $\nu^{1/2}$ (blue line, compound **4c** 1. oxidation: ΔE_p = 3.68 mV$^{-1/2}$ s$^{1/2}$ $\nu^{1/2}$ + **70.31 mV** (r^2 = 0.96); black line, compound **4c** 2. oxidation: ΔE_p = 4.09 mV$^{-1/2}$ s$^{1/2}$ $\nu^{1/2}$ + **60.60 mV** (r^2 = 1.00)).

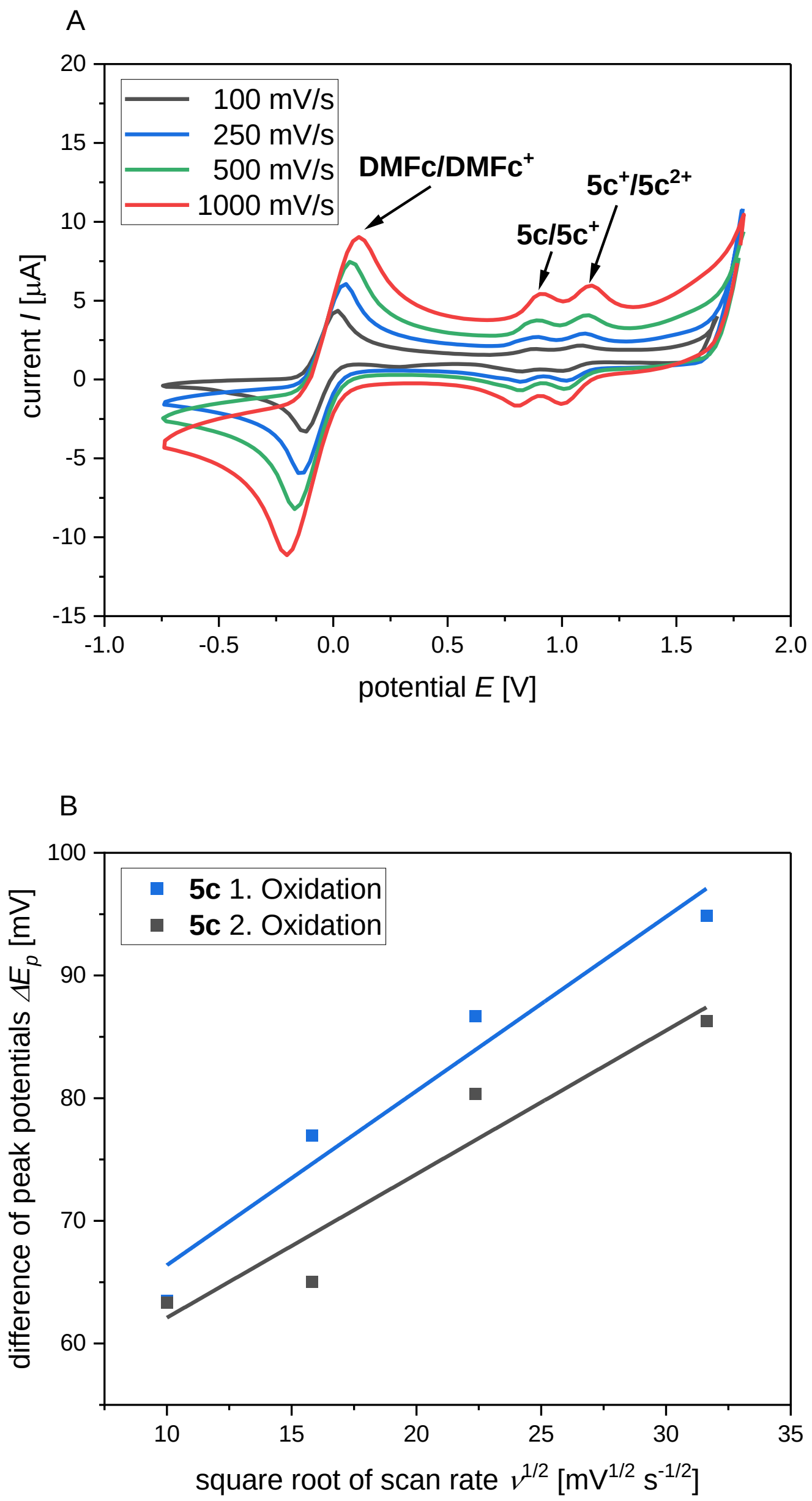

Figure S69. A: Cyclic voltammogram of compound **5c** and redox standard **DMFc** recorded in dichloromethane at different scan rates (T = 293 K, ν = 100–1000 mV/s, electrolyte: [^{n}Bu$_4$N][PF$_6$], Pt working electrode, Pt counter electrode, Ag/AgCl reference electrode. B: Difference of peak potentials ΔE_p vs. square root of scan rate $\nu^{1/2}$ (blue line, compound **5c** 1. oxidation: ΔE_p = 1.42 mV$^{-1/2}$ s$^{1/2}$ $\nu^{1/2}$ + **52.19 mV** (r^2 = 0.96); black line, compound **5c** 2. oxidation: ΔE_p = 1.17 mV$^{-1/2}$ s$^{1/2}$ $\nu^{1/2}$ + **50.39 mV** (r^2 = 1.00)).

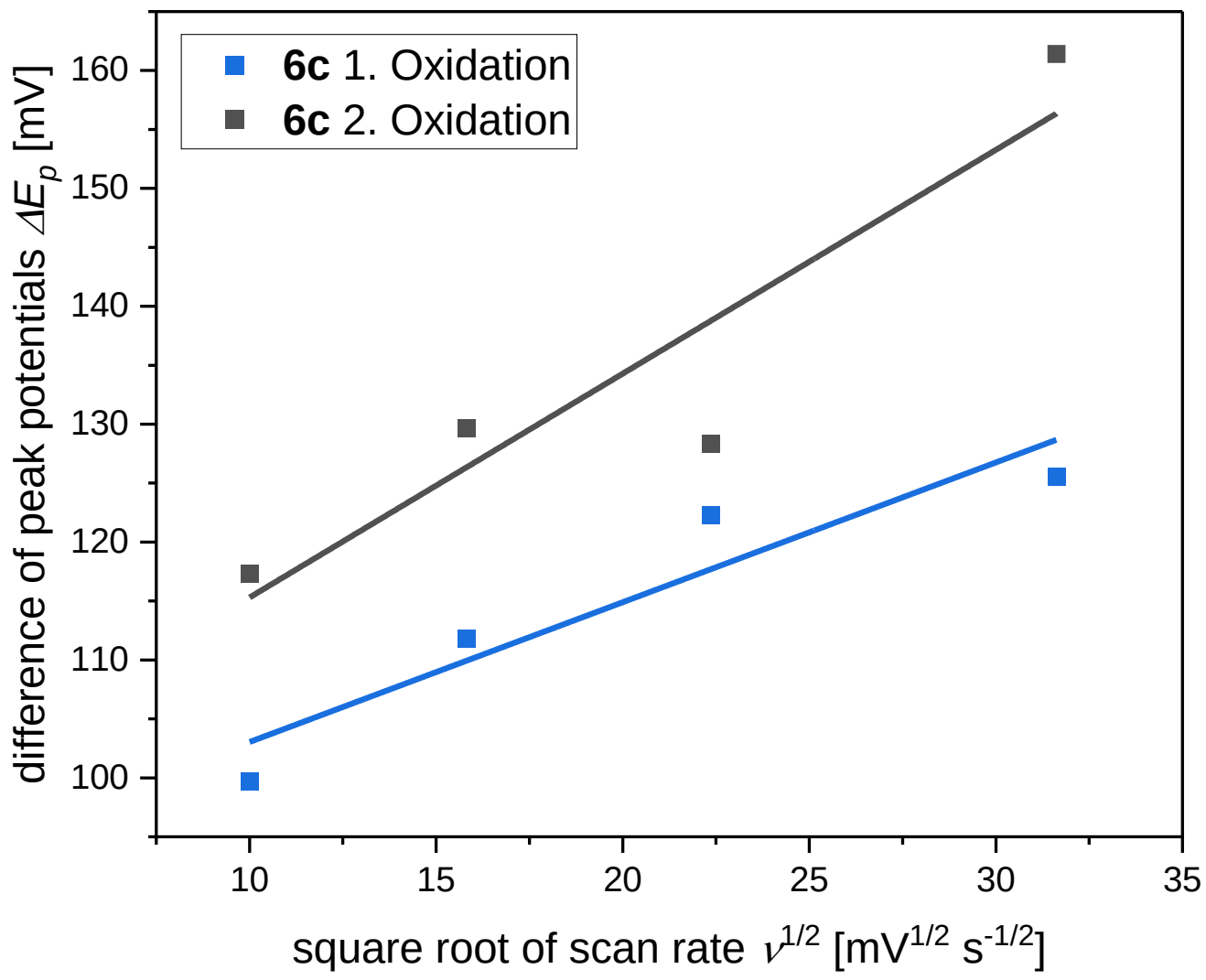

Figure S70. Difference of peak potentials ΔE_p vs. square root of scan rate $\nu^{1/2}$ (blue line, compound **6c** 1. oxidation: ΔE_p = 1.19 mV$^{-1/2}$ s$^{1/2}$ $\nu^{1/2}$ + **91.18 mV** (r^2 = 0.94); black line, compound **6c** 2. oxidation: ΔE_p = 1.90 mV$^{-1/2}$ s$^{1/2}$ $\nu^{1/2}$ + **96.31 mV** (r^2 = 0.93)).

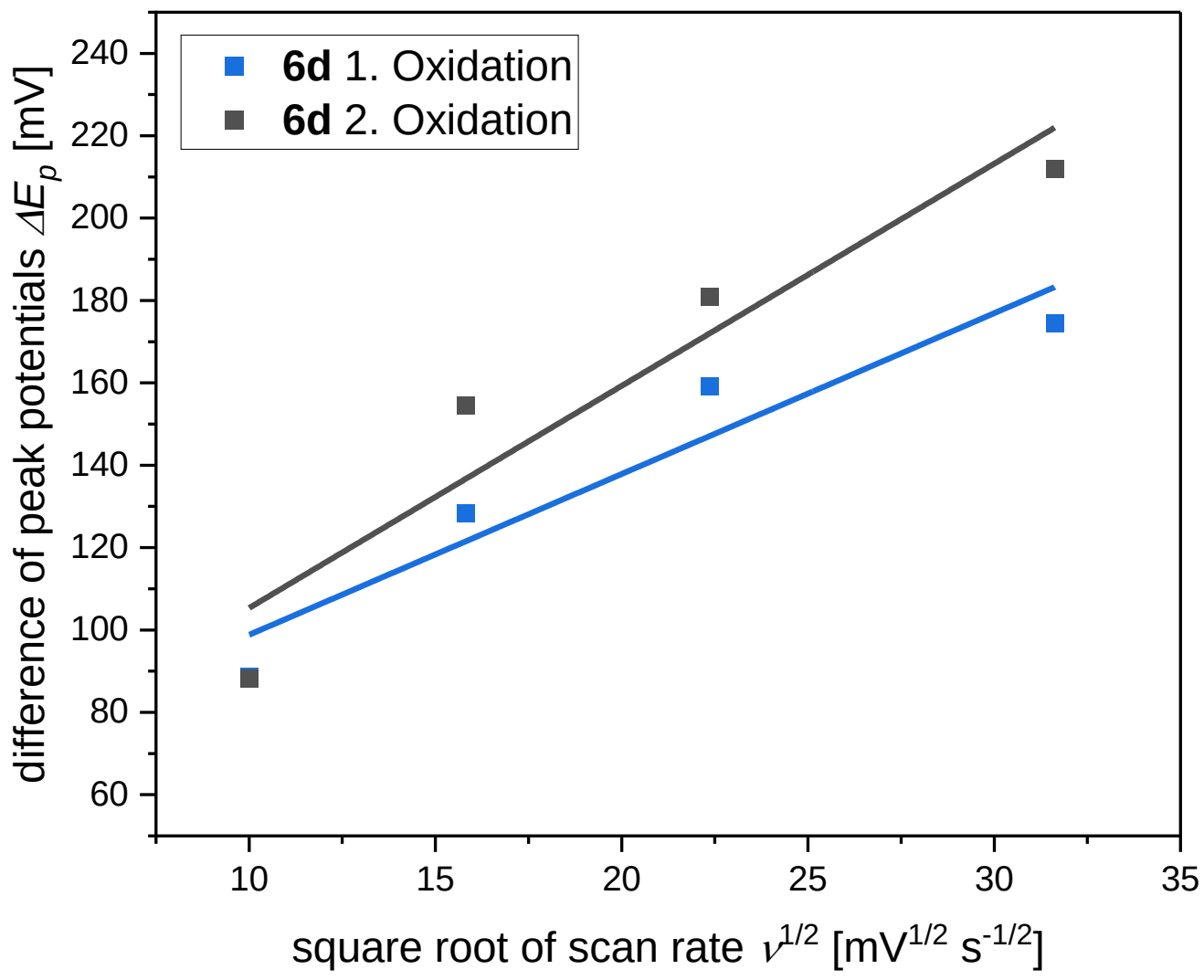

Figure S71. Difference of peak potentials ΔE_p vs. square root of scan rate $\nu^{1/2}$ (blue line, compound **6d** 1. oxidation: ΔE_p = 3.90 mV$^{-1/2}$ s$^{1/2}$ $\nu^{1/2}$ + **59.77 mV** (r^2 = 0.96); black line, compound **6d** 2. oxidation: ΔE_p = 5.39 mV$^{-1/2}$ s$^{1/2}$ $\nu^{1/2}$ + **51.39 mV** (r^2 = 0.95)).

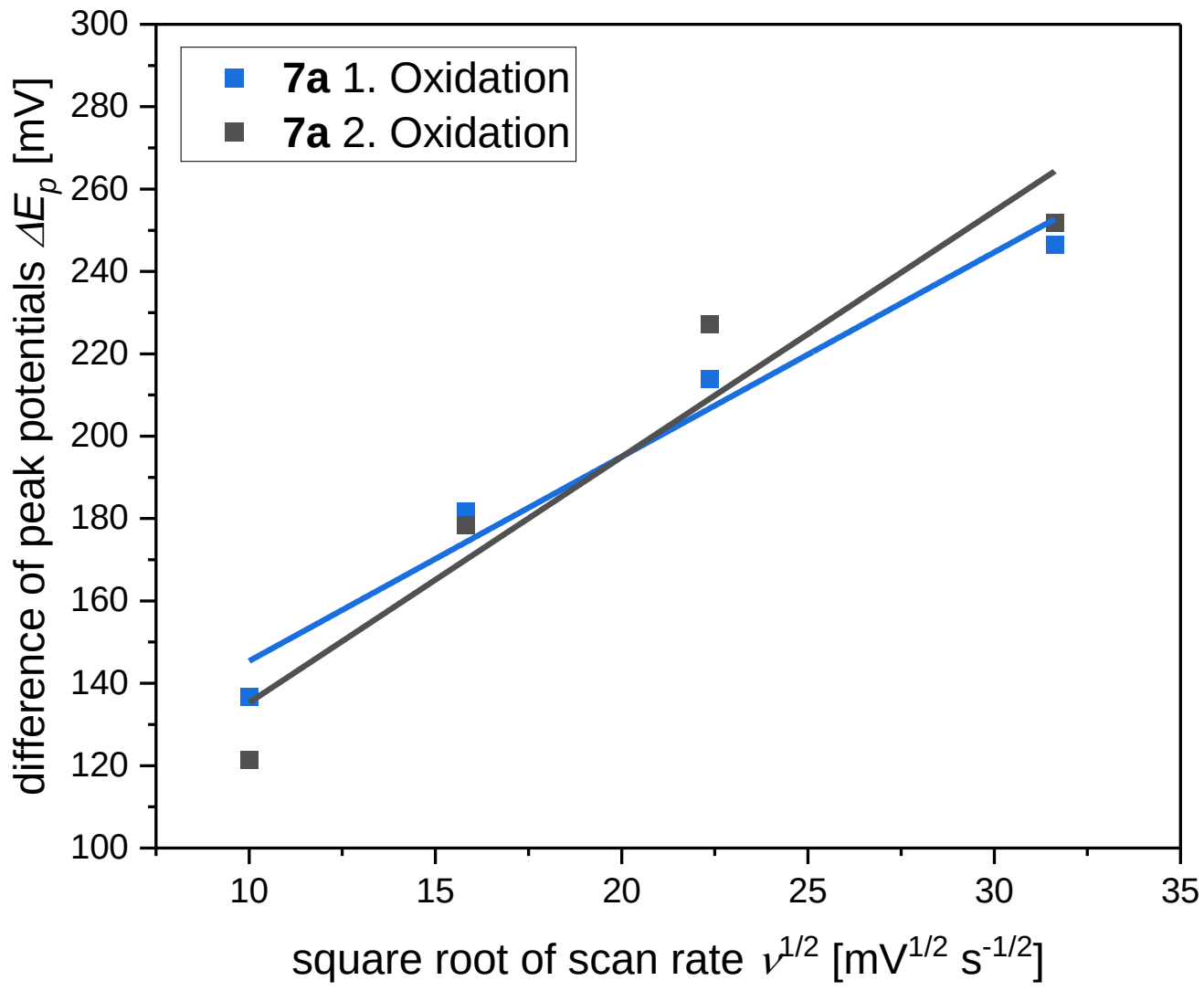

Figure S72. Difference of peak potentials ΔE_p vs. square root of scan rate $\nu^{1/2}$ (blue line, compound **7a** 1. oxidation: ΔE_p = 4.96 mV$^{-1/2}$ s$^{1/2}$ $\nu^{1/2}$ + **95.78 mV** (r^2 = 0.98); black line, compound **7a** 2. oxidation: ΔE_p = 5.97 mV$^{-1/2}$ s$^{1/2}$ $\nu^{1/2}$ + **75.63 mV** (r^2 = 0.95)).

Table S4. Utilized values of the difference of peak potentials ΔE_p vs. square root of scan rate $\nu^{1/2}$ plots of dimers **4–6c**.

scan rate [mV]	square root of scan rate $\nu^{1/2}$ [mV$^{-1/2}$ s$^{1/2}$]	compound **4c**		compound **5c**		compound **6c**	
		ΔE_p 1. oxidation [mV]	ΔE_p 2. oxidation [mV]	ΔE_p 1. oxidation [mV]	ΔE_p 2. oxidation [mV]	ΔE_p 1. oxidation [mV]	ΔE_p 2. oxidation [mV]
100	10.00	111.23	98.50	63.49	63.33	99.68	117.33
250	15.81	131.17	130.46	76.98	65.02	111.84	129.67
500	22.36	138.09	150.22	86.70	80.33	122.27	128.33
1000	31.62	194.04	189.62	94.87	86.31	125.54	161.42

Table S5. Utilized values of the difference of peak potentials ΔE_p vs. square root of scan rate $\nu^{1/2}$ plots of dimers **6d** and **7a**.

scan rate [mV]	square root of scan rate $\nu^{1/2}$	compound **6d**		compound **7a**	
		ΔE_p 1. oxidation	ΔE_p 2. oxidation	ΔE_p 1. oxidation	ΔE_p 2. oxidation
100	10.00	88.59	88.33	136.79	121.43
250	15.81	128.37	154.67	181.77	178.33
500	22.36	159.26	181.00	213.98	227.20
1000	31.62	174.45	212.00	246.54	251.72

Table S6. Extrapolated values of the peak potentials difference ΔE_p to a scan rate of 0 mV/s.

compound	ΔE_p 1. oxidation	ΔE_p 2. oxidation	ΔE_p 1. reduction
**4a**	52.58	53.12	nd[a]
**4b**	59.20	55.10	nd[a]
**4c**	70.31	60.60	nd[a]
**5c**	52.19	50.39	nd[a]
**5d**	97.10	nd	nd[a]
**6a**	53.72	56.31	nd[a]
**6b**	56.45	93.85	nd[a]
**6c**	91.18	96.31	nd[a]
**6d**	59.77	51.39	77.72
**6e**	56.28	67.44	53.01
**7a**	95.78	75.63	nd[a]
**7b**	72.72	53.16	nd[a]
**7c**	53.72	58.80	nd[a]

[a] Values cannot be determined.

5.5 Electrochemical process of compound 5d

For compound **5d** instead of the expected distinct two oxidation processes, a broad electrochemical signal with an additional shoulder in the cathodic wave is observed and therefore, additional analyses are performed to further investigate the origin of this behavior (Figure S57). First, the peak width at half maximum (FWHM) is determined from the cathodic wave and found to be approximately 390 mV, which is substantially larger than the theoretical value of 90.4 mV expected for a reversible one-electron Nernstian process[12] (Figure S73). This observation is indicative for the possibility of two oxidation processes overlapping.

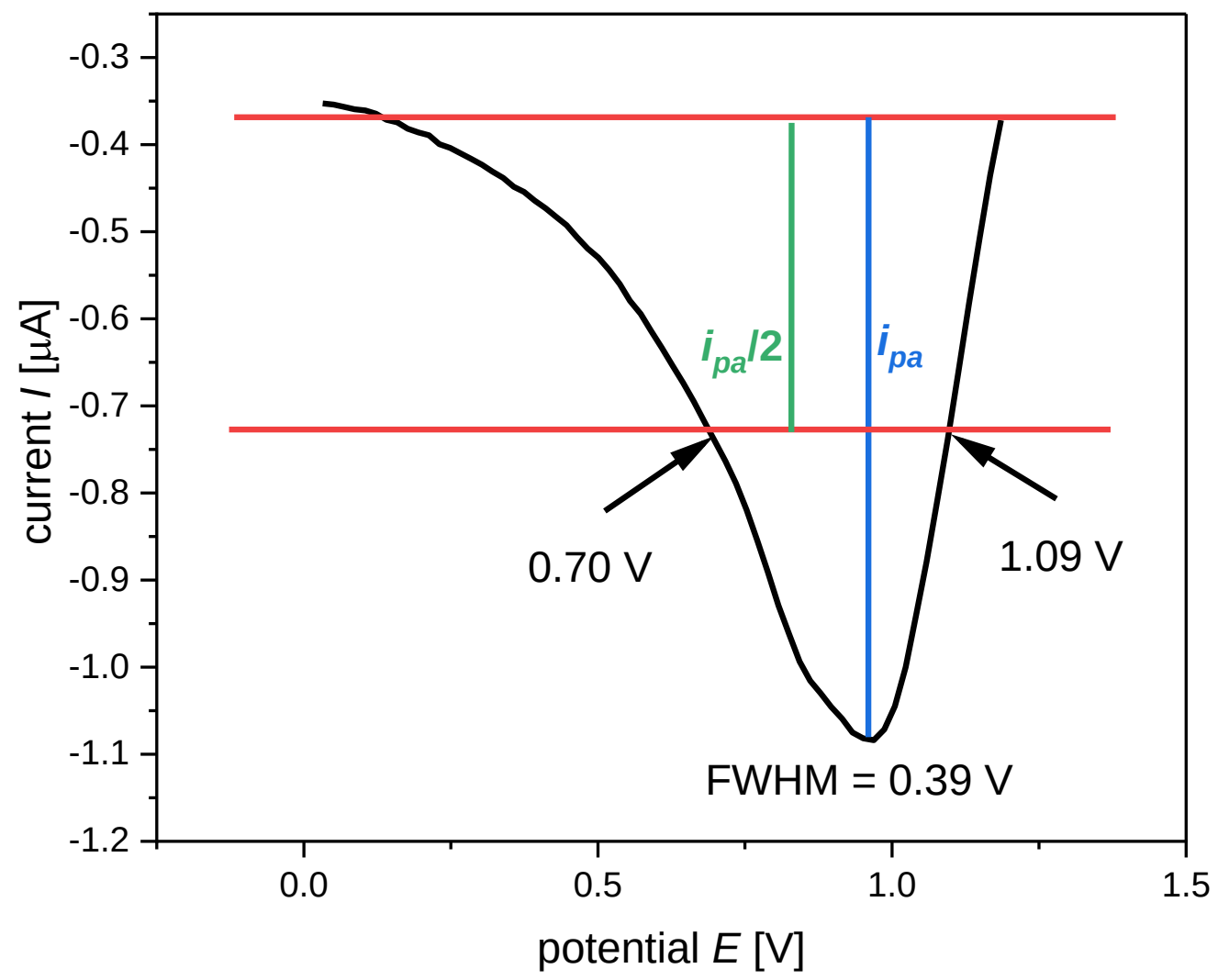

Figure S73. Peak width at half maximum analysis of the cathodic wave of compound **5d** recorded in dichloromethane (T = 293 K, ν = 100 mV/s, electrolyte: [^{n}Bu$_4$N][PF$_6$], Pt working electrode, Pt counter electrode, Ag/AgCl reference electrode. Spectra not corrected by the standard DMFc.

To assess whether the broad signal originates from two electrochemical processes that are sufficiently separated to be experimentally resolved, cyclic voltammograms were recorded at different scan rates (Figure S74). No discernible peak splitting or development of additional features was observed over the investigated scan rate range, indicating that the underlying processes remain strongly overlapped under these experimental conditions.

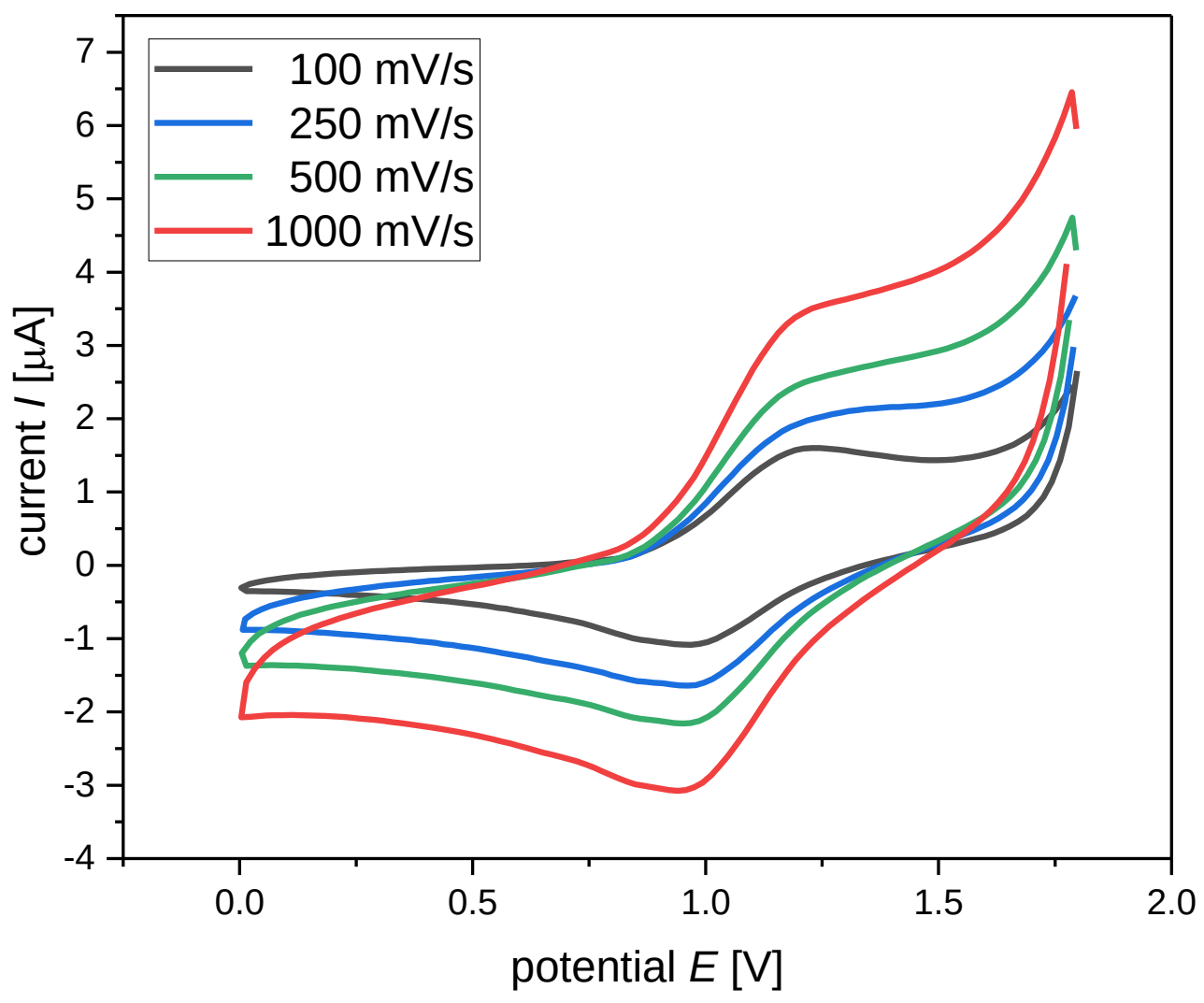

Figure S74. Cyclic voltammogram of compound **5d** recorded in dichloromethane at different scan rates (T = 293 K, ν = 100–1000 mV/s, electrolyte: [^{n}Bu$_4$N][PF$_6$], Pt working electrode, Pt counter electrode, Ag/AgCl reference electrode. Spectra not corrected by the standard DMFc.

Furthermore, a deconvolution analysis was performed by calculating the semi-derivative of the current with respect to time using the el-Chem Viewer software package[13]. Semi-derivative voltammetry is known to enhance the resolution of closely spaced redox events. However, this treatment did not reveal any distinct peak separation, providing further evidence that the individual processes cannot be resolved directly from the experimental data (Figure S75).

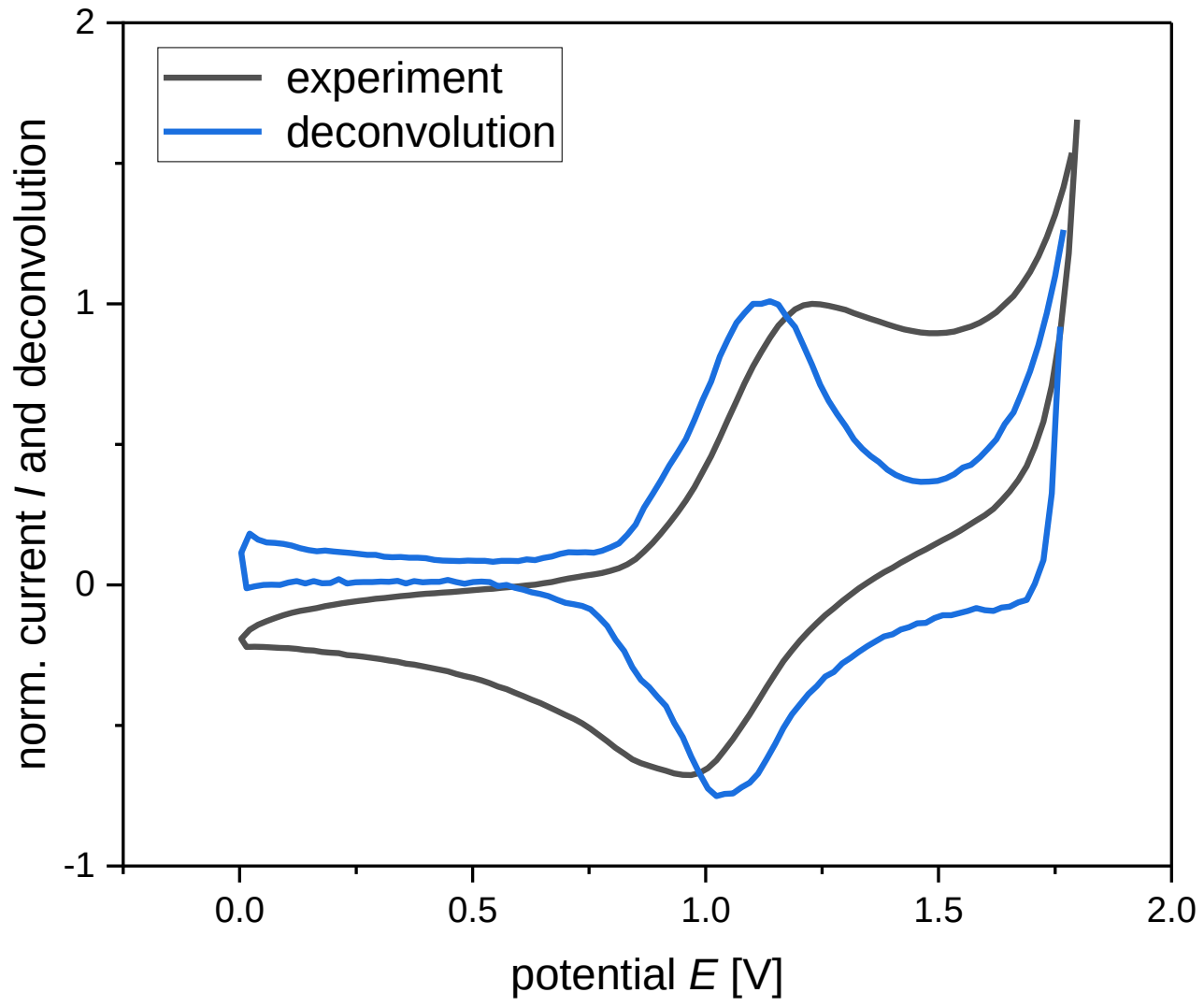

Figure S75. Cyclic voltammogram of compound **5d** recorded in dichloromethane in black (T = 293 K, ν = 100 mV/s, electrolyte: [^{n}Bu$_4$N][PF$_6$], Pt working electrode, Pt counter electrode, Ag/AgCl reference electrode) and deconvolution spectra in blue. Spectra not corrected by the standard DMFc.

In addition, quantitative peak-shape analyses are performed using both Gaussian and Lorentzian fitting functions (Figure S76A and B). The broad signal could be satisfactorily reproduced using a two-component model consisting of one narrow and one broad contribution (corrected r^2 values of 0.98–0.99). While both models provided satisfactory fits to the experimental data , the Lorentzian functions reproduced the experimental peak shape more accurately. The Lorentzian model consisting of a narrow contribution centered at 0.98 V and a broader contribution centered at 0.82 V. The separation between the fitted peak maxima (0.16 V) is significantly smaller than the width of the broad component (0.43 V), resulting in extensive overlap and preventing experimental resolution of the individual contributions (Table S7). While this result supports the possibility that the observed wave may arise from two overlapping electrochemical processes, the absence of experimentally resolved peak splitting suggests that the corresponding formal potentials are very close and therefore remain indistinguishable under the applied conditions. Based on these complementary investigations, the assignment of the broad wave of compound **5d** to two strongly overlapping redox processes remains a plausible interpretation since a single Lorentzian function do not adequately reproduce the experimental peak shape (adjusted r^2 = 0.95).

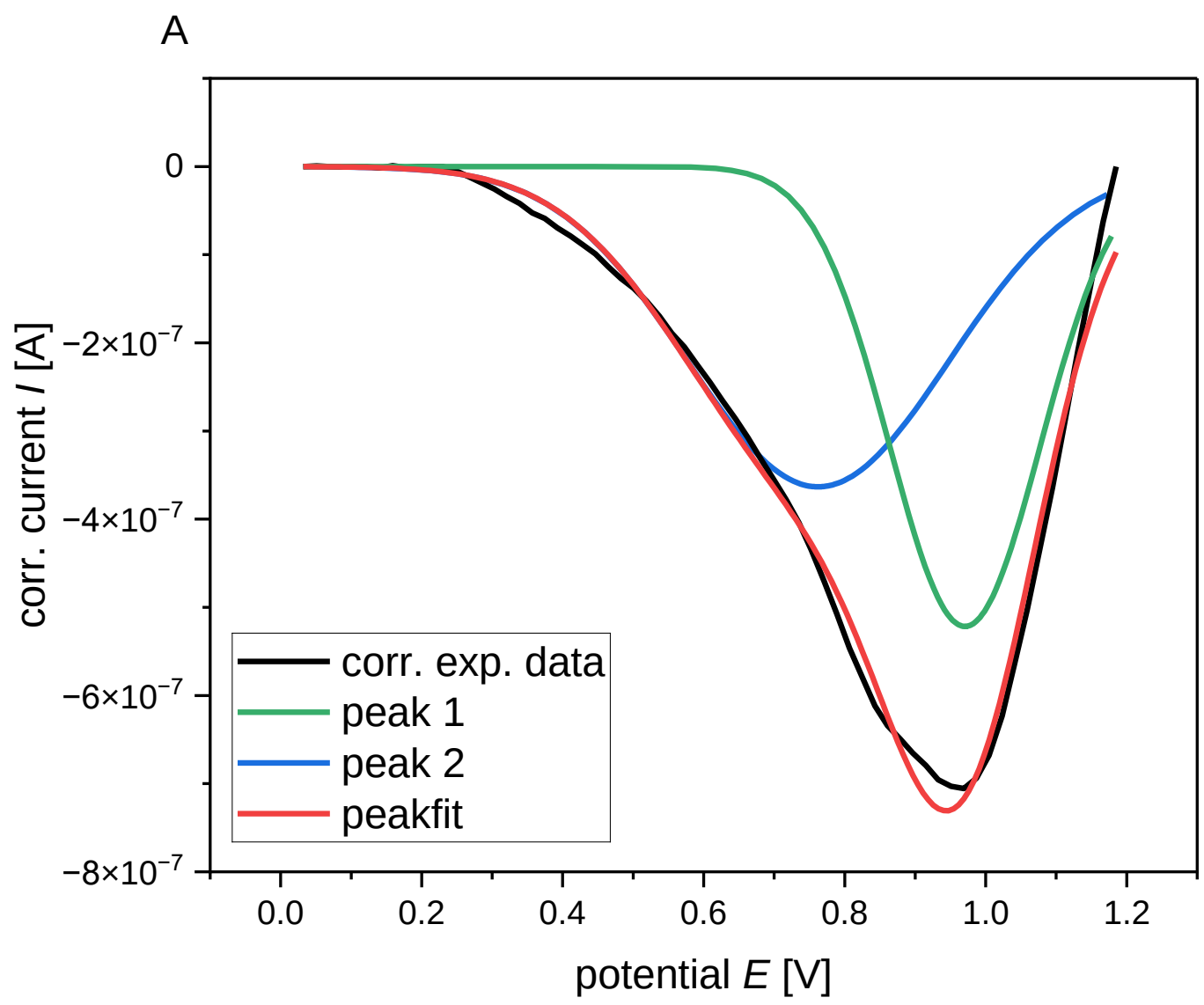

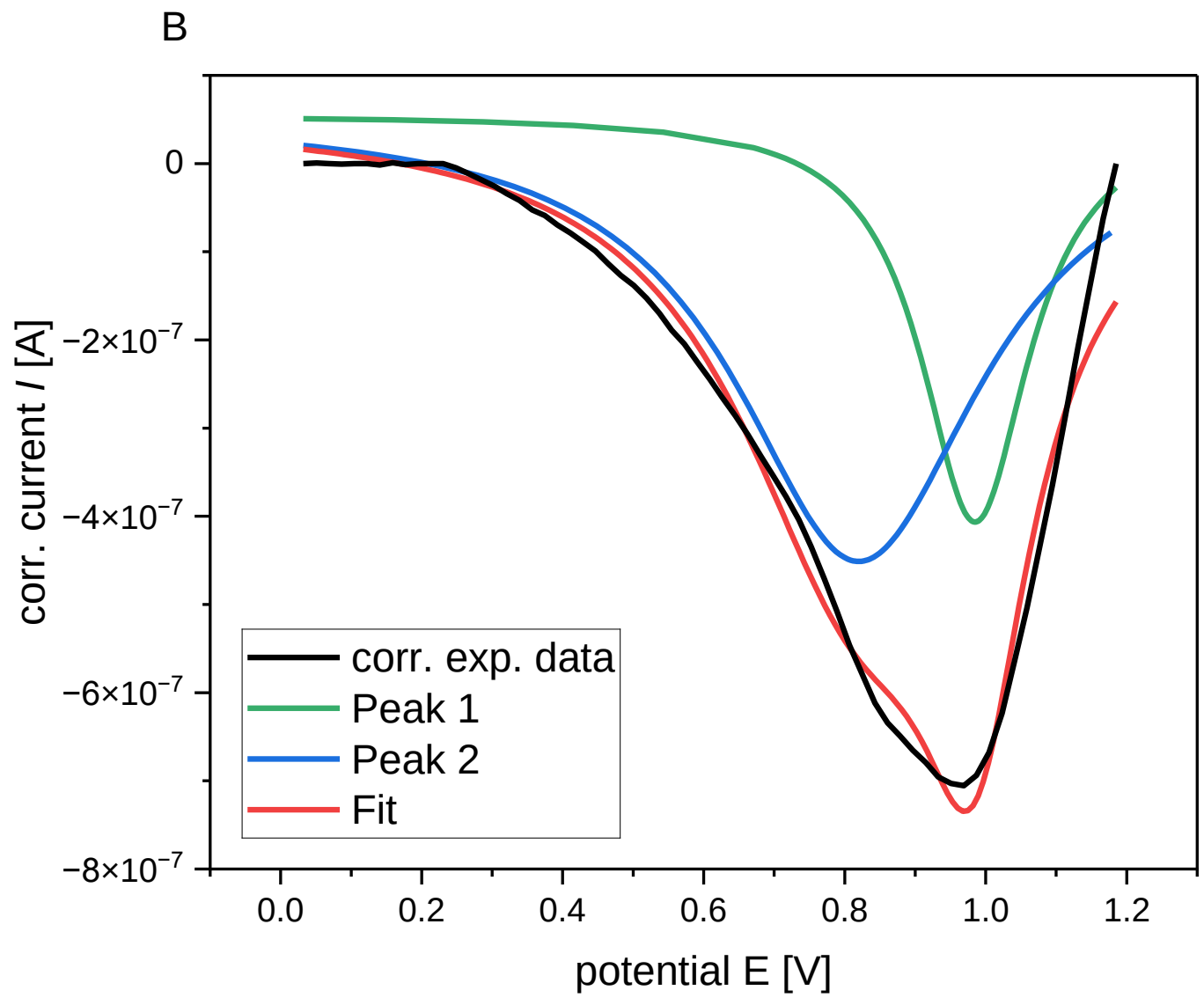

Figure S76. Peak-shape analysis of the cathodic wave of the cyclic voltammogram of compound **5d** recorded in dichloromethane in black (T = 293 K, ν = 100 mV/s, electrolyte: [$^n Bu_4N$][PF_6], Pt working electrode, Pt counter electrode, Ag/AgCl reference electrode), corrected to a baseline of 0 A. A: Gaussian fitting functions utilizing two Peaks (Peak 1 in green and Peak 2 in blue) resulting in an overall fit in red (corrected r^2 = 0.99). B: Lorentzian fitting functions utilizing two Peaks (Peak 1 in green and Peak 2 in blue) resulting in an overall fit in red (corrected r^2 = 0.98).

Table S7. Parameter of peak-shape analysis of the cathodic wave of compound **5d** by Gaussian and Lorentzian fitting functions.

	Gaussian fitting		Lorentzian fitting	
	Peak 1	Peak 2	Peak 1	Peak 2
peak maximum [V]	0.97	0.76	0.98	0.82
relative peak area [%]	45	55	28	72
FWHM [V]	0.25	0.44	0.19	0.43
peak seperation [V]	0.21		0.16	

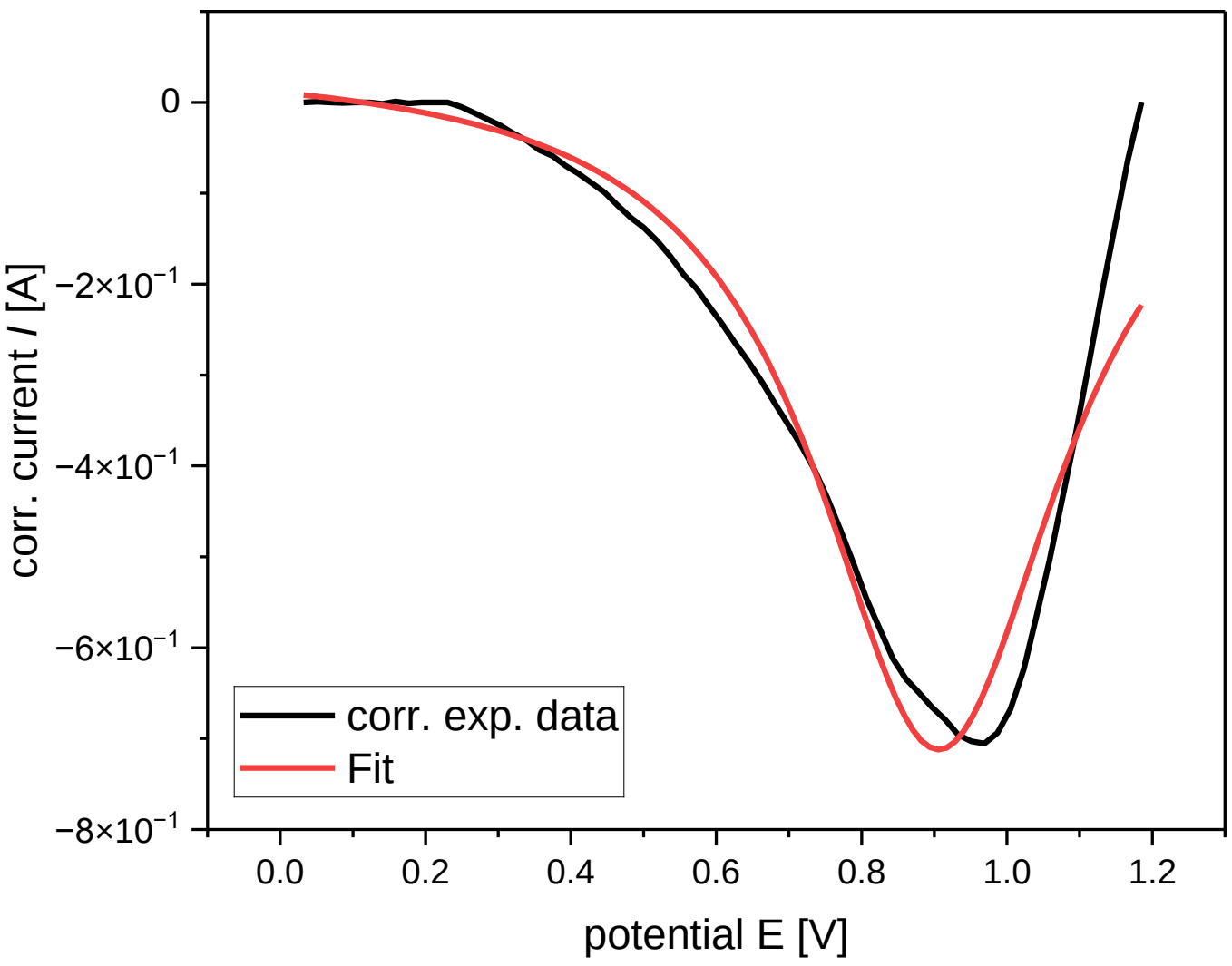

Figure S77. Peak-shape analysis of the cathodic wave of the cyclic voltammogram of compound **5d** recorded in dichloromethane in black (T = 293 K, ν = 100 mV/s, electrolyte: [$^{n}Bu_4N$][PF_6], Pt working electrode, Pt counter electrode, Ag/AgCl reference electrode), corrected to a baseline of 0 A. Lorentzian fitting functions utilizing one peak (peak 1 in green and peak 2 in blue) resulting in an overall fit in red (corrected r^2 = 0.95).

6 Photophysical data

6.1 Absorption and emission spectra of *para-/meta-/ortho*-triarylamine dimers (*sym-p-/m-/o*-bTAA)$_2$ 4, 5, 6 and 7

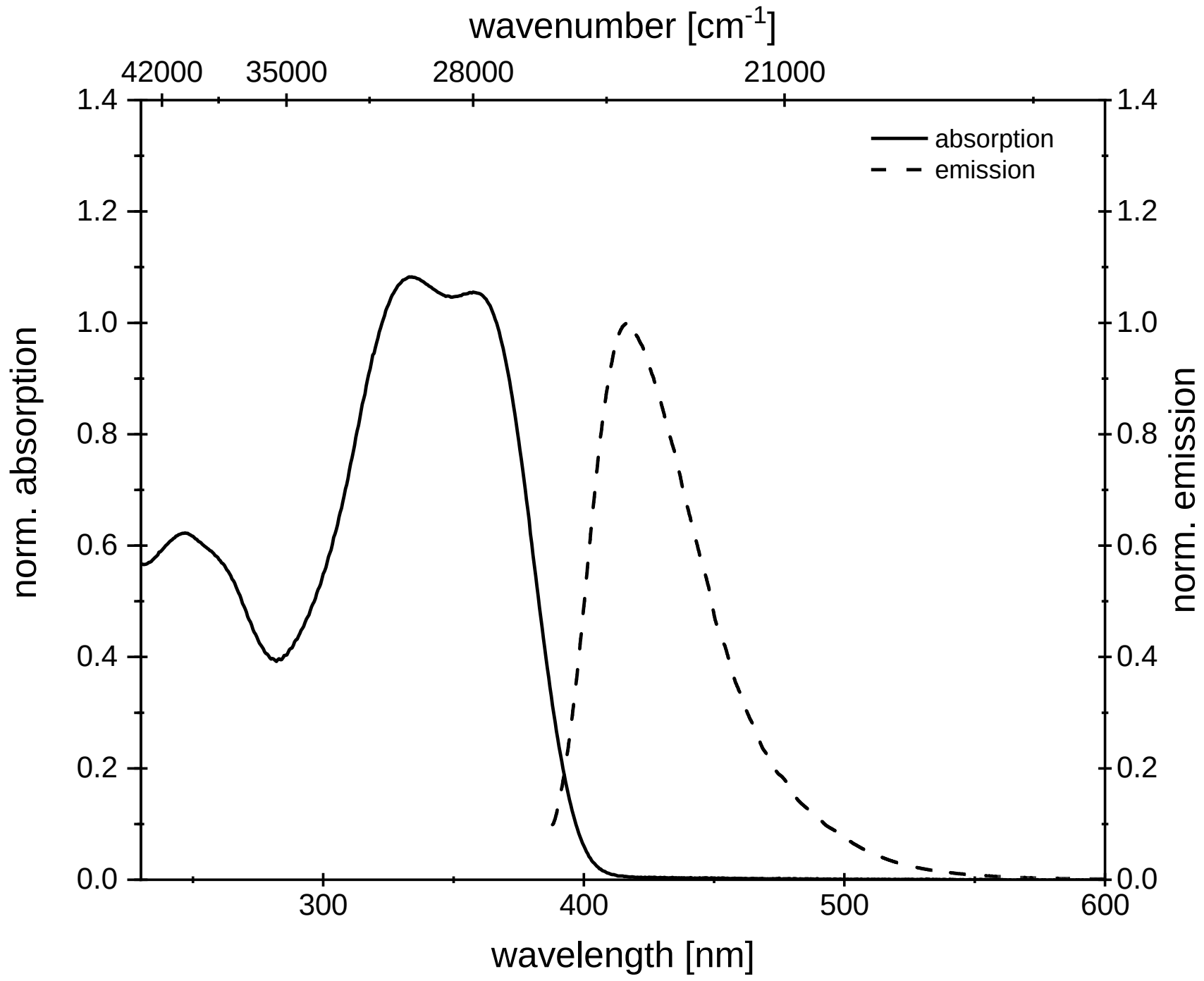

Figure S78. Absorption (solid line) and emission (dashed line) spectra of compound **4a** (recorded in dichloromethane, T = 293 K, c_{abs}(**4a**) = 10^{-5} M, c_{em}(**4a**) = 10^{-7} M, $\lambda_{ex} = \lambda_{max,abs}$).

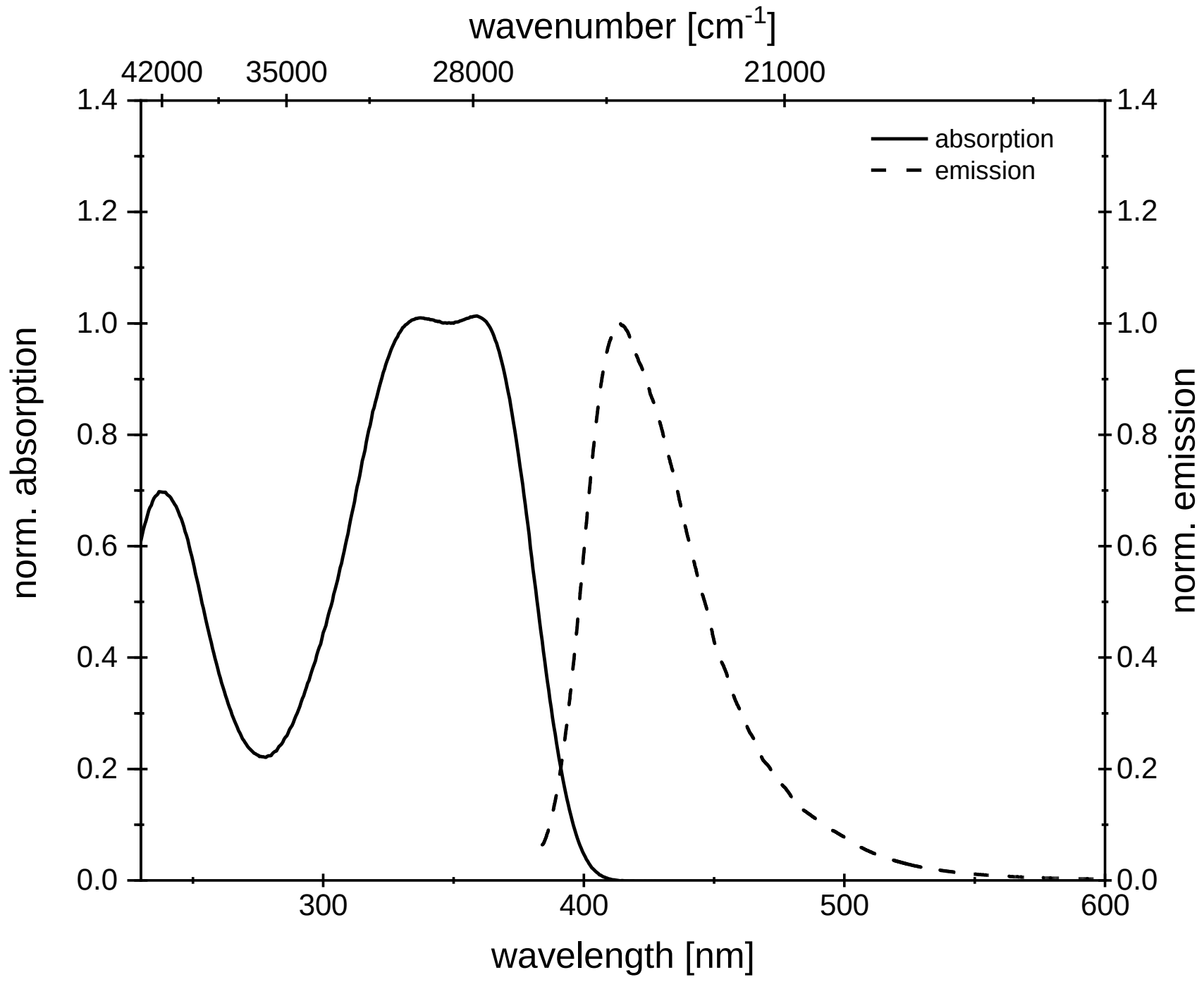

Figure S79. Absorption (solid line) and emission (dashed line) spectra of compound **4b** (recorded in dichloromethane, T = 293 K, c_{abs}(**4b**) = 10^{-5} M, c_{em}(**4b**) = 10^{-7} M, $\lambda_{ex} = \lambda_{max,abs}$).

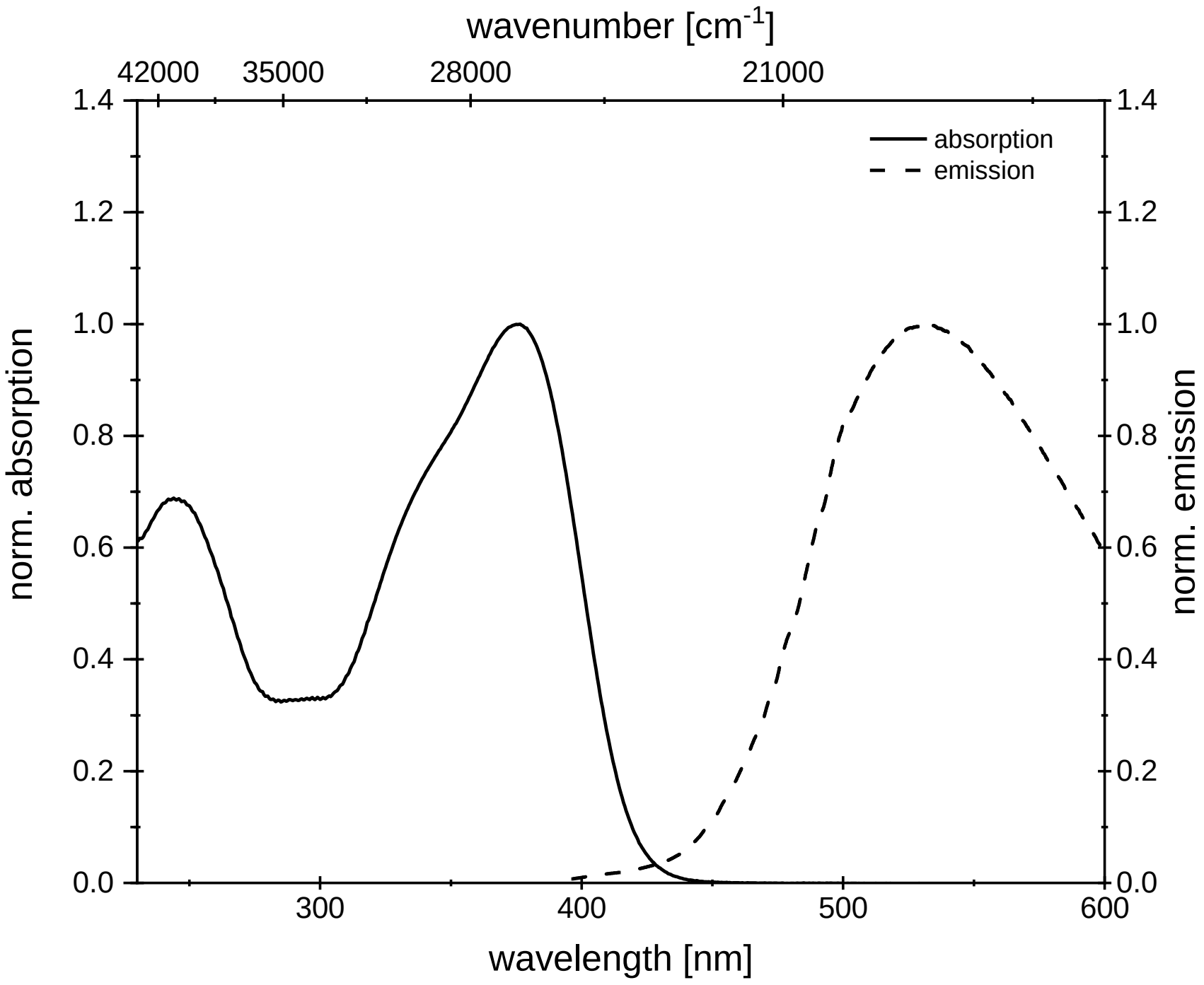

Figure S80. Absorption (solid line) and emission (dashed line) spectra of compound **4c** (recorded in dichloromethane, T = 293 K, c_{abs}(**4c**) = 10^{-5} M, c_{em}(**4c**) = 10^{-7} M, $\lambda_{ex} = \lambda_{max,abs}$).

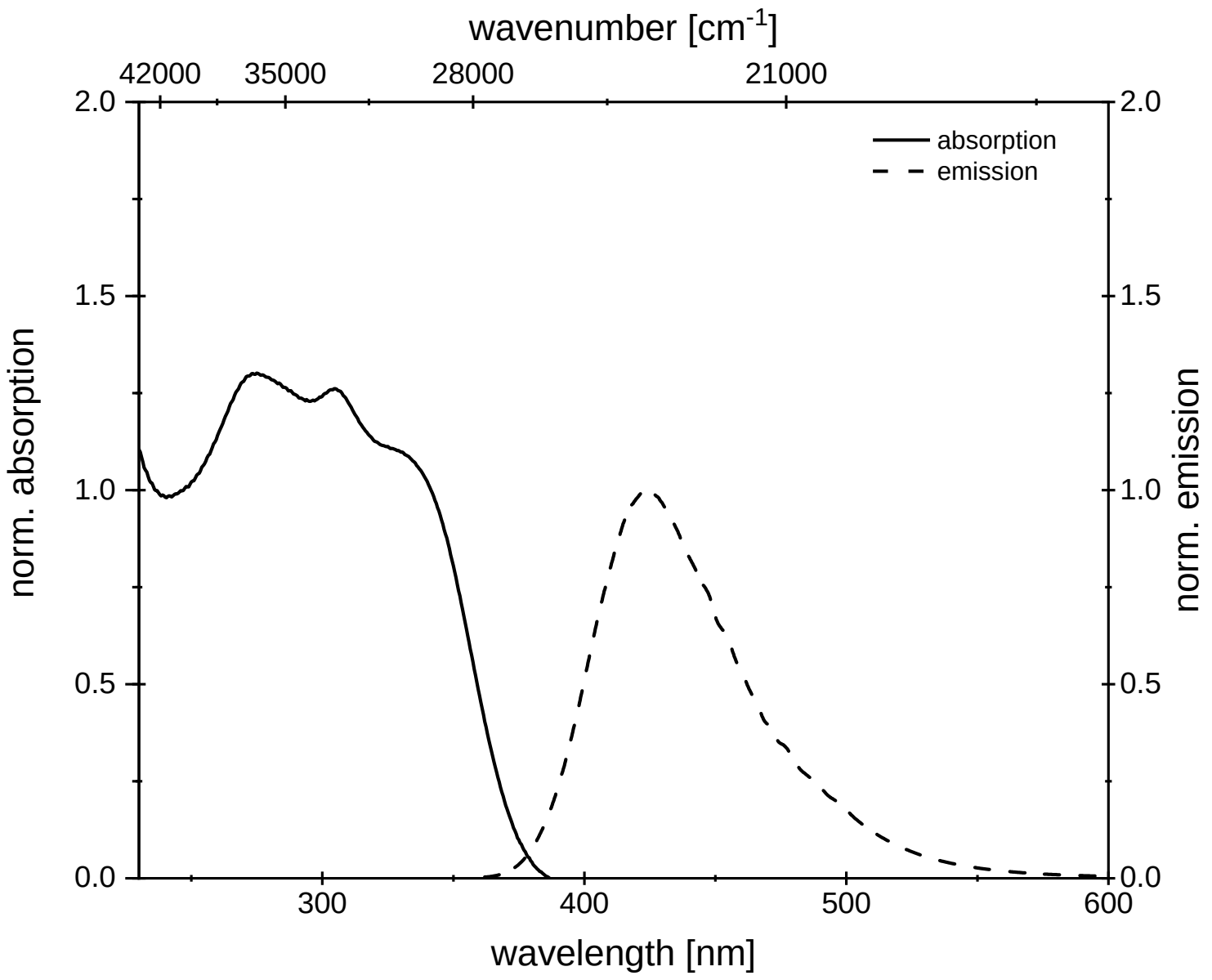

Figure S81. Absorption (solid line) and emission (dashed line) spectra of compound mixture **5a/5a'/5a''** (recorded in dichloromethane, T = 293 K, c_{abs}(**5a/5a'/5a''**) = 10^{-5} M, c_{em}(**5a/5a'/5a''**) = 10^{-7} M, $\lambda_{ex} = \lambda_{max,abs}$).

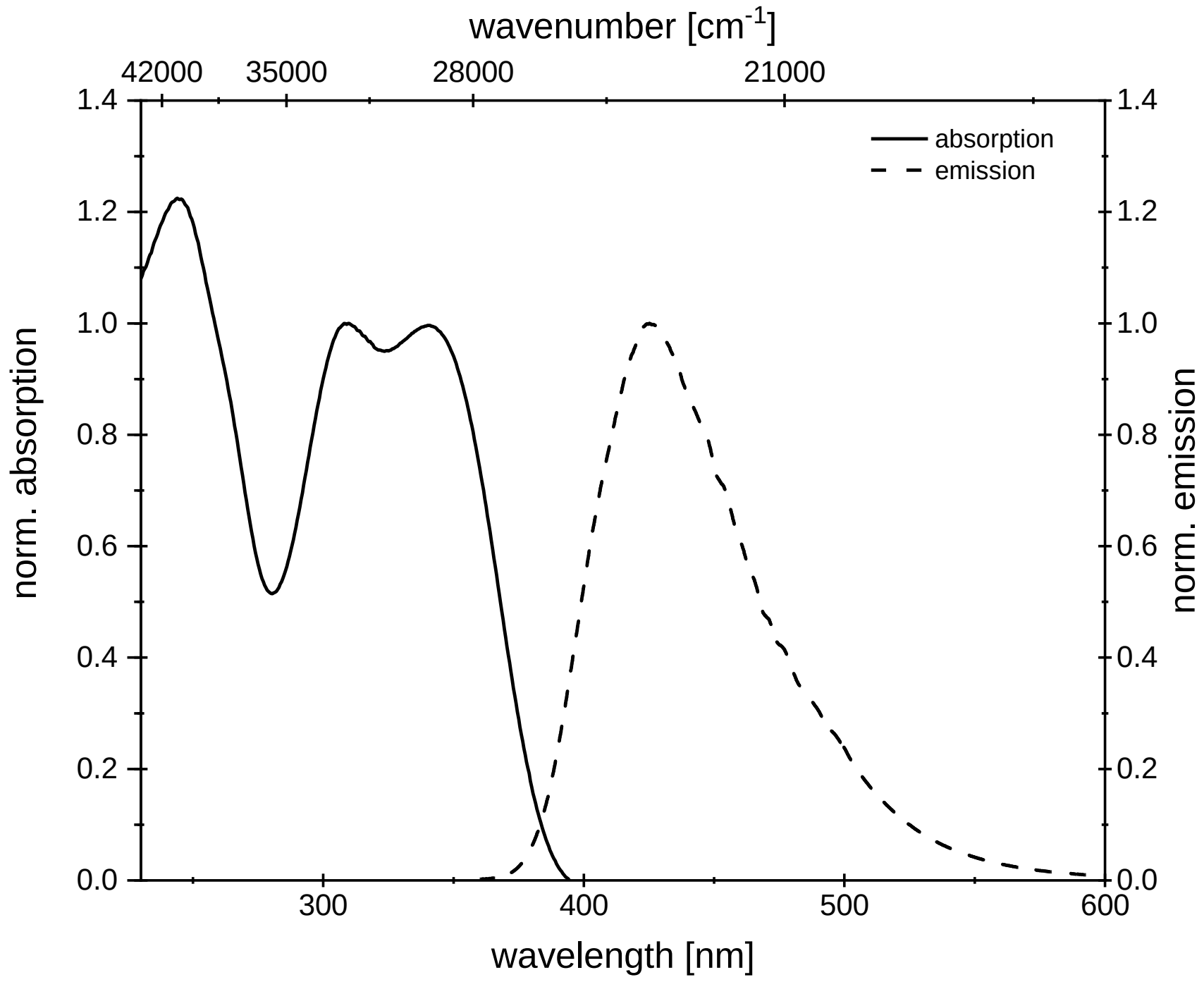

Figure S82. Absorption (solid line) and emission (dashed line) spectra of compound mixture **5b/5b'/5b''** (recorded in dichloromethane, T = 293 K, c_{abs}(**5b/5b'/5b''**) = 10^{-5} M, c_{em}(**5b/5b'/5b''**) = 10^{-7} M, $\lambda_{ex} = \lambda_{max,abs}$).

Table S8. Photophysical data of the regioisomeric mixtures **5a/5a'/5a''** and **5b/5b'/5b''** recorded in dichloromethane, T = 293 K, c_{abs} = 10^{-5} M, c_{em} = 10^{-7} M, $\lambda_{ex} = \lambda_{max,abs}$.

compound mixtures	$\lambda_{max,abs}$ [nm][a] (ε [M^{-1} cm^{-1}])	$\lambda_{max,em(solution)}$ [nm][b] (Φ_F)[c]	$\Delta\tilde{\nu}_S$ [cm^{-1}][d]	$\lambda_{max,em(solid)}$ [nm][e] (Φ_F)[f]
**5a/5a'/5a''**	274 (36500), 306 (sh, 35800), 342 (sh, 27700)	424 (0.48)	5600	440 (0.07)
**5b/5b'/5b''**	244 (44200), 316 (36000), 335 (36000)	425 (0.37)	6300	430 (0.24)

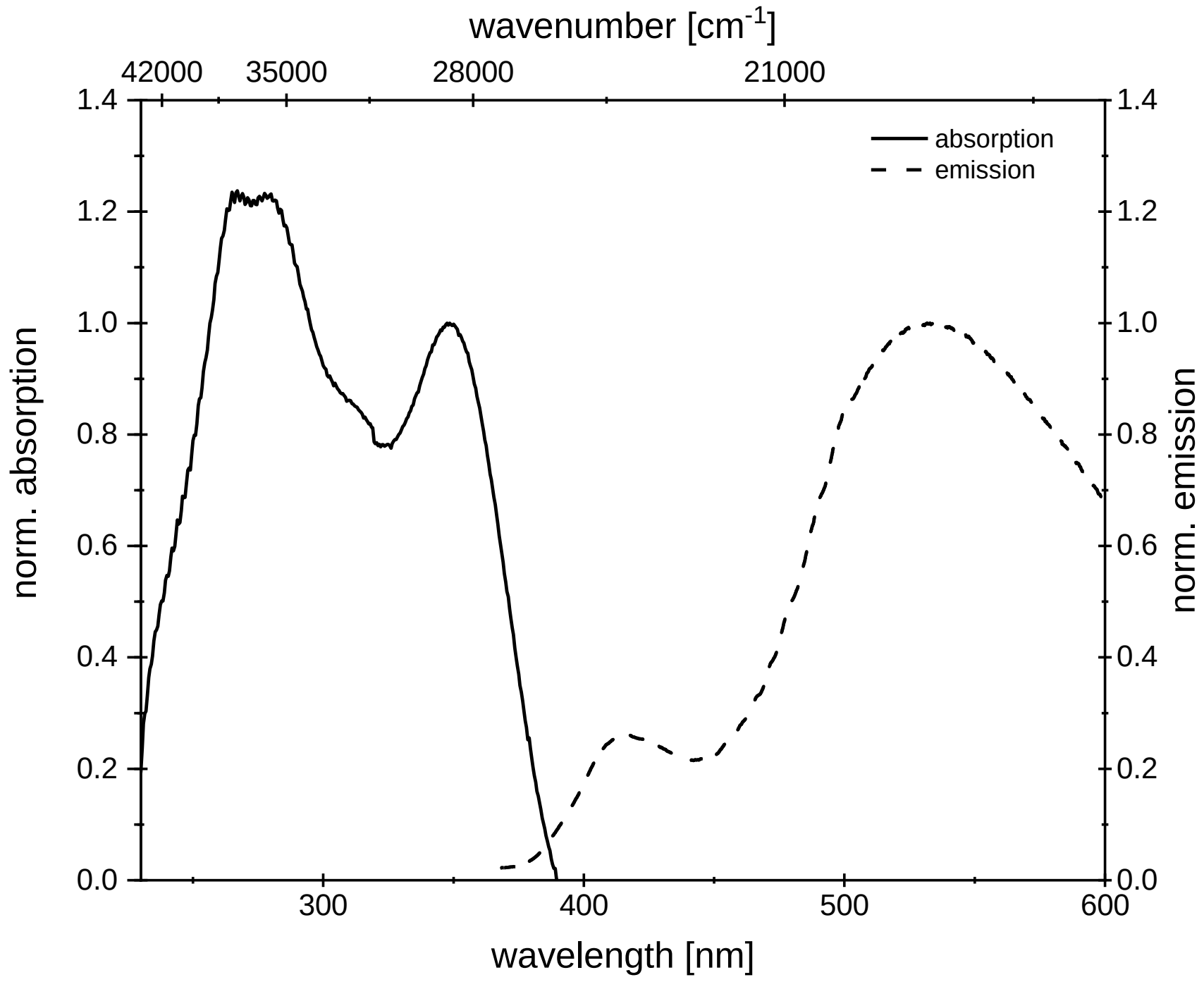

Figure S83. Absorption (solid line) and emission (dashed line) spectra of compound **5c** (recorded in dichloromethane, T = 293 K, c_{abs}(**5c**) = 10^{-5} M, c_{em}(**5c**) = 10^{-7} M, $\lambda_{ex} = \lambda_{max,abs}$).

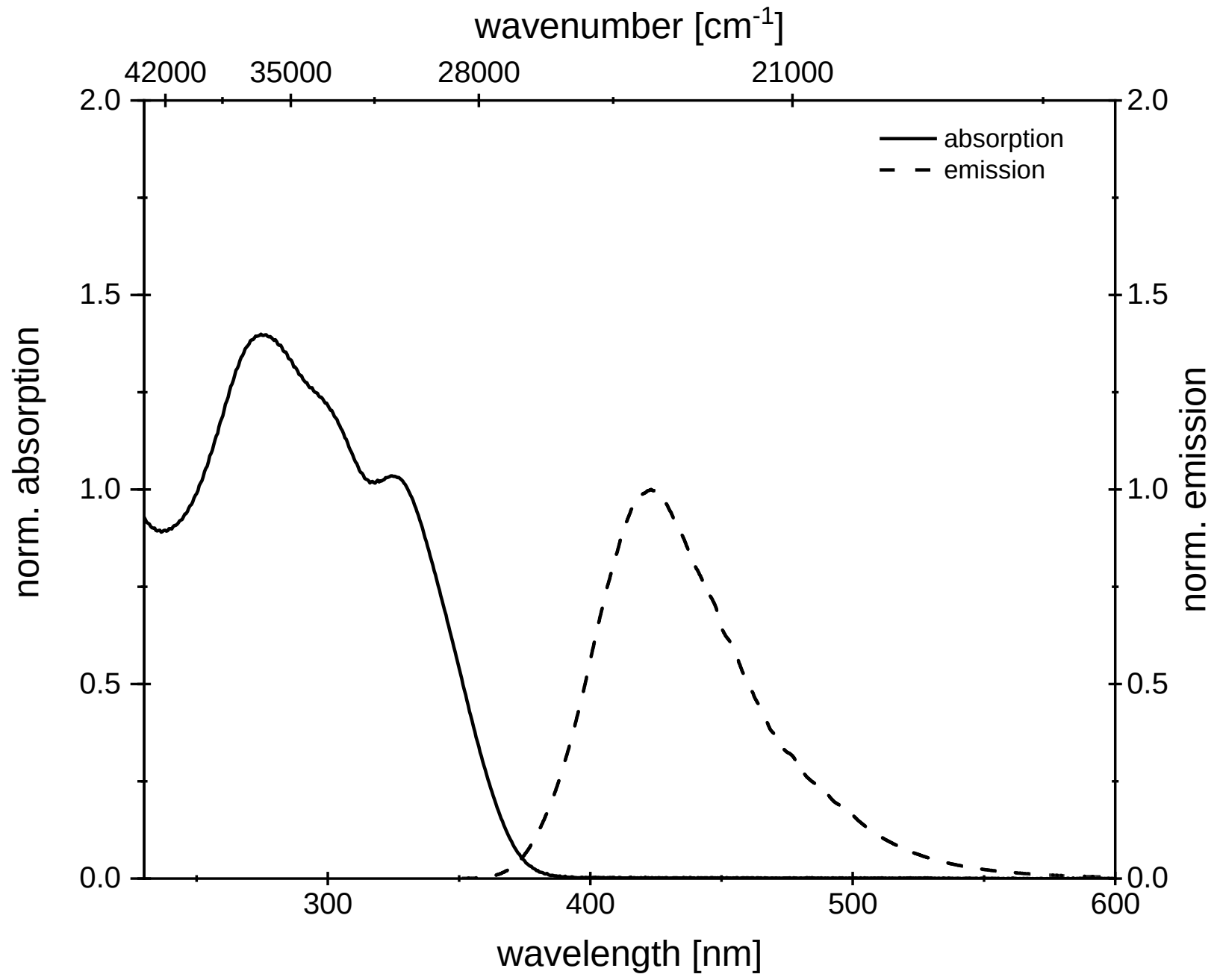

Figure S84. Absorption (solid line) and emission (dashed line) spectra of compound **5d** (recorded in dichloromethane, T = 293 K, c_{abs}(**5d**) = 10^{-5} M, c_{em}(**5d**) = 10^{-7} M, $\lambda_{ex} = \lambda_{max,abs}$).

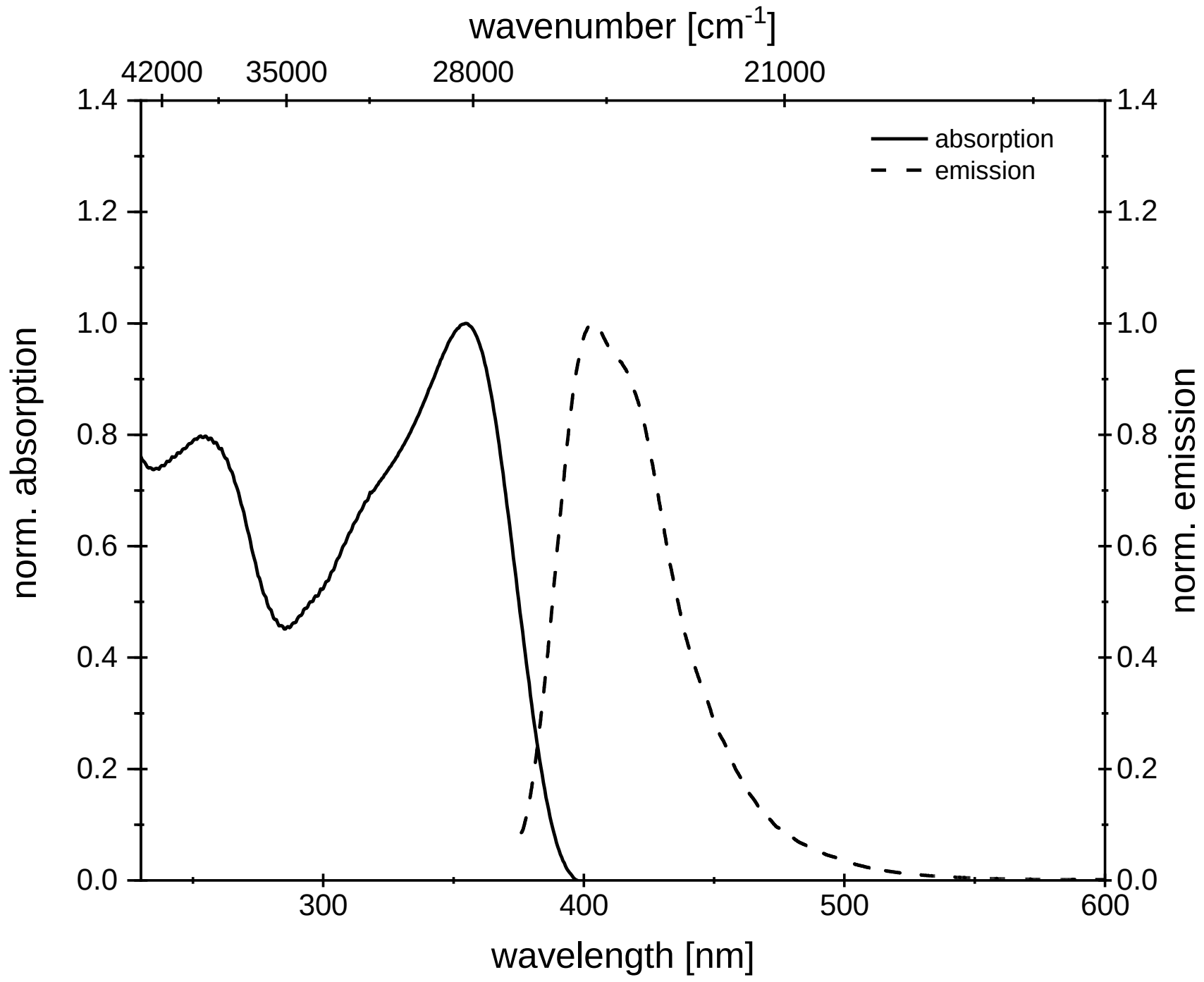

Figure S85. Absorption (solid line) and emission (dashed line) spectra of compound **6a** (recorded in dichloromethane, T = 293 K, c_{abs}(**6a**) = 10^{-5} M, c_{em}(**6a**) = 10^{-7} M, $\lambda_{ex} = \lambda_{max,abs}$).

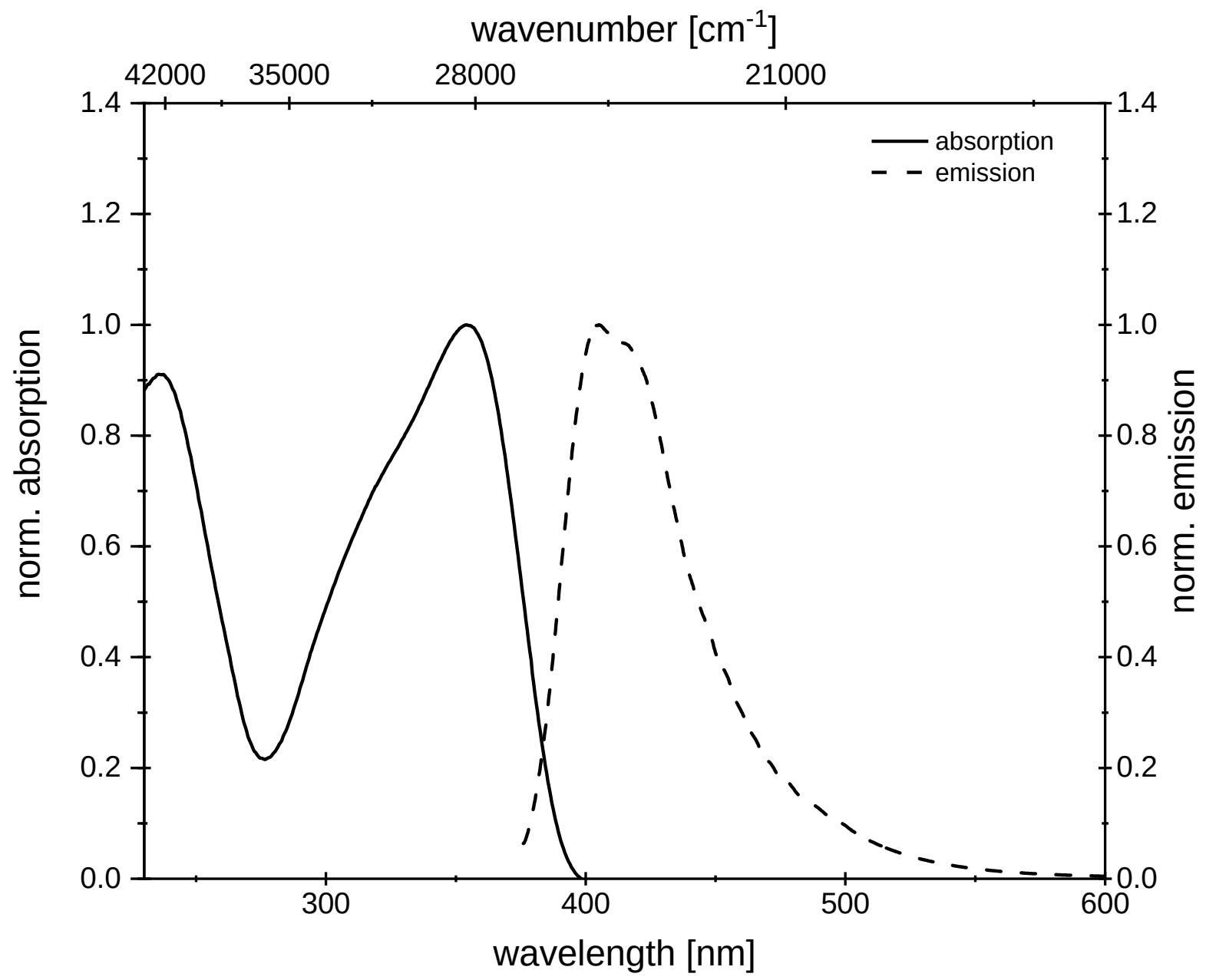

Figure S86. Absorption (solid line) and emission (dashed line) spectra of compound **6b** (recorded in dichloromethane, T = 293 K, c_{abs}(**6b**) = 10^{-5} M, c_{em}(**6b**) = 10^{-7} M, $\lambda_{ex} = \lambda_{max,abs}$).

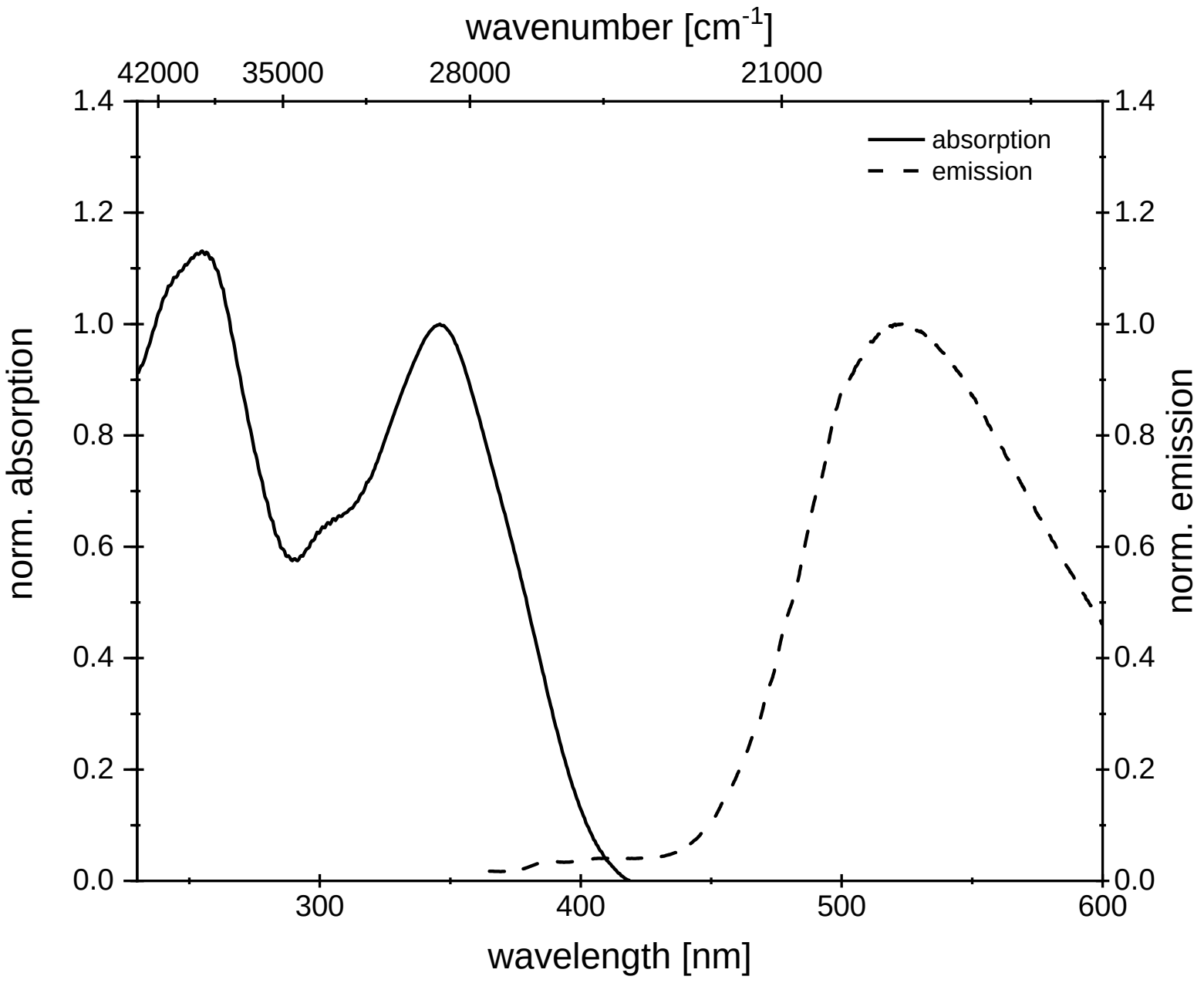

Figure S87. Absorption (solid line) and emission (dashed line) spectra of compound **6c** (recorded in dichloromethane, T = 293 K, c_{abs}(**6c**) = 10^{-5} M, c_{em}(**6c**) = 10^{-7} M, $\lambda_{ex} = \lambda_{max,abs}$).

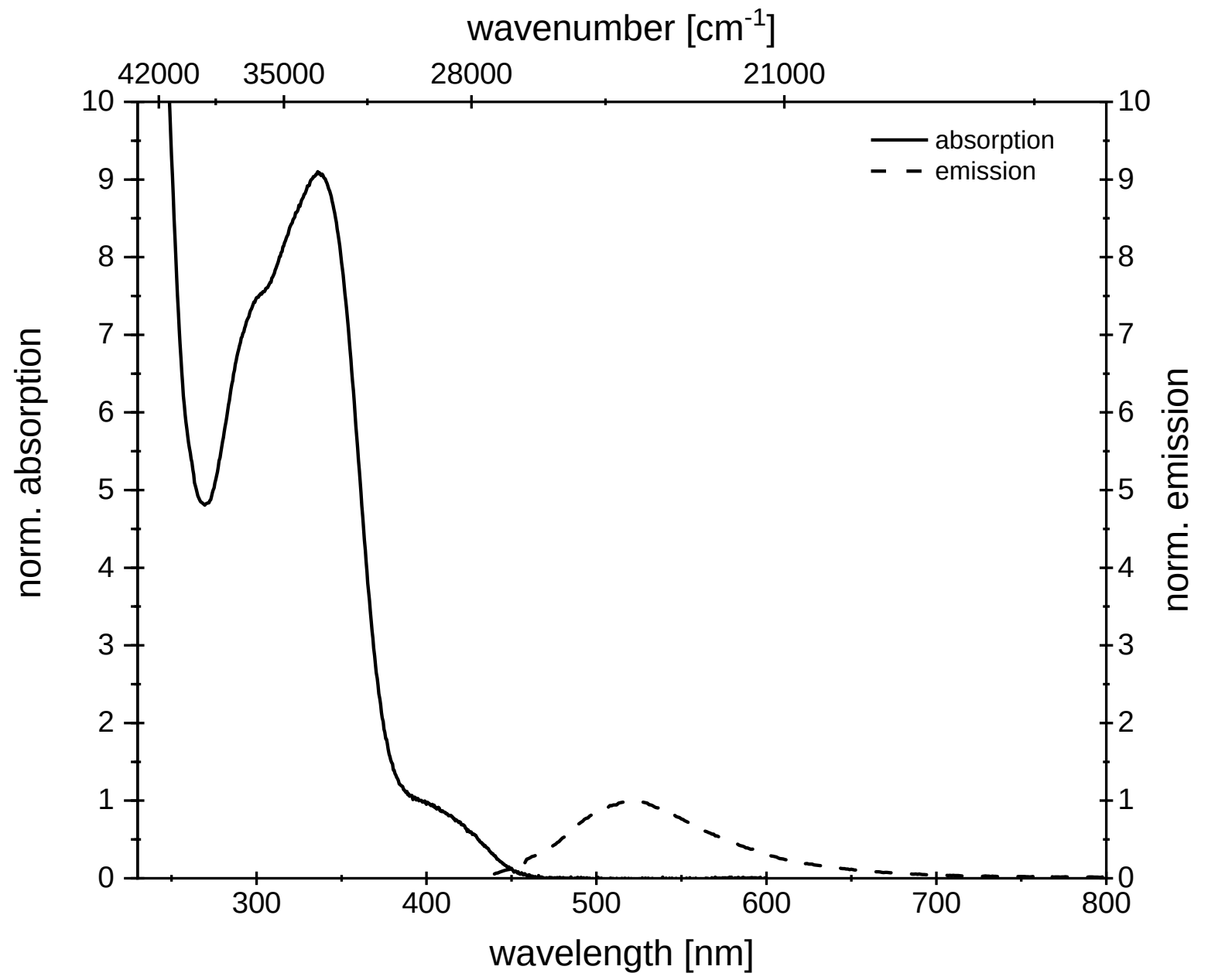

Figure S88. Absorption (solid line) and emission (dashed line) spectra of compound **6d** (recorded in cyclohexane, T = 293 K, c_{abs}(**6d**) = 10^{-5} M, c_{em}(**6d**) = 10^{-7} M, $\lambda_{ex} = \lambda_{max,abs}$).

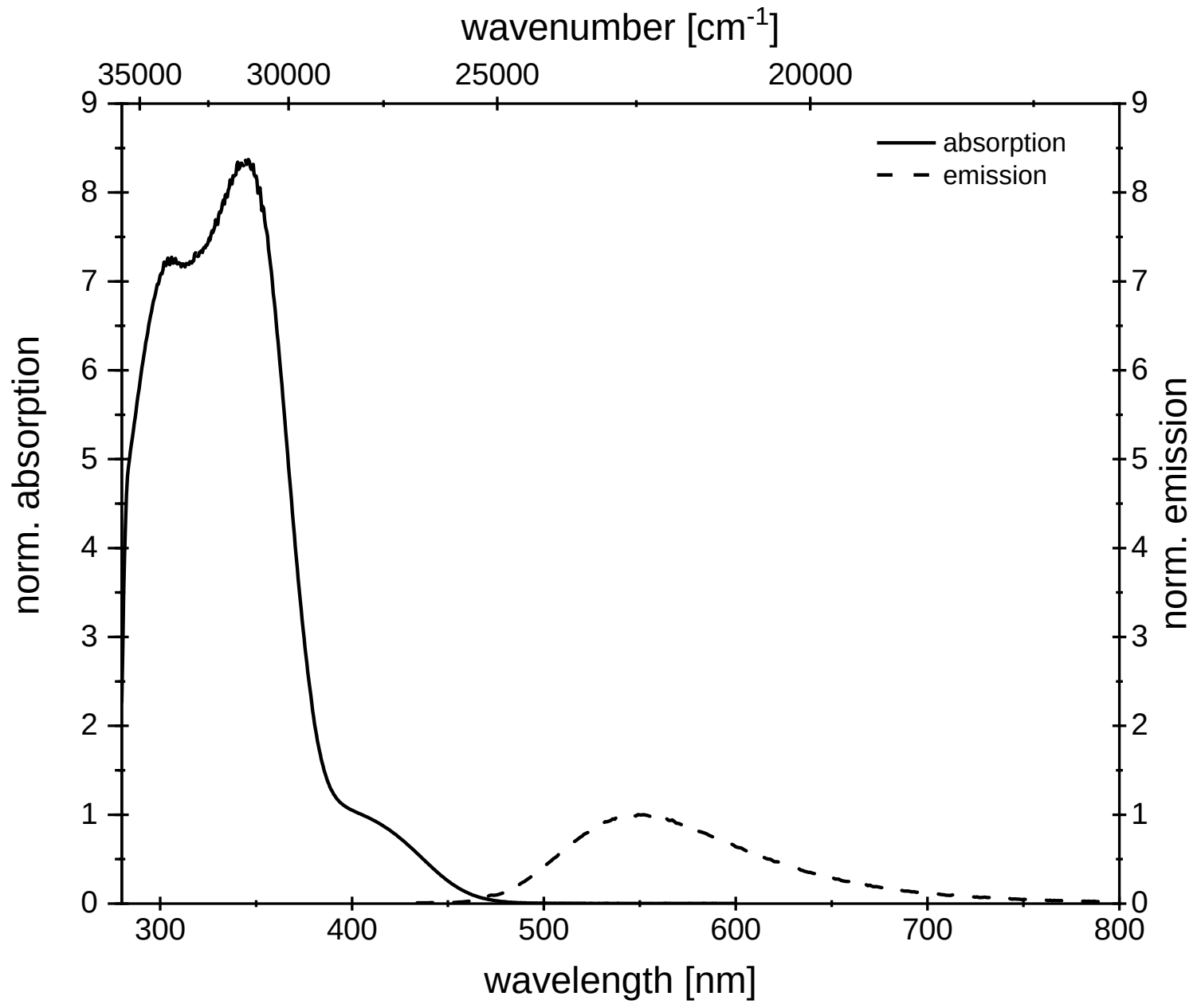

Figure S89. Absorption (solid line) and emission (dashed line) spectra of compound **6d** (recorded in toluene, T = 293 K, c_{abs}(**6d**) = 10^{-5} M, c_{em}(**6d**) = 10^{-7} M, $\lambda_{ex} = \lambda_{max,abs}$).

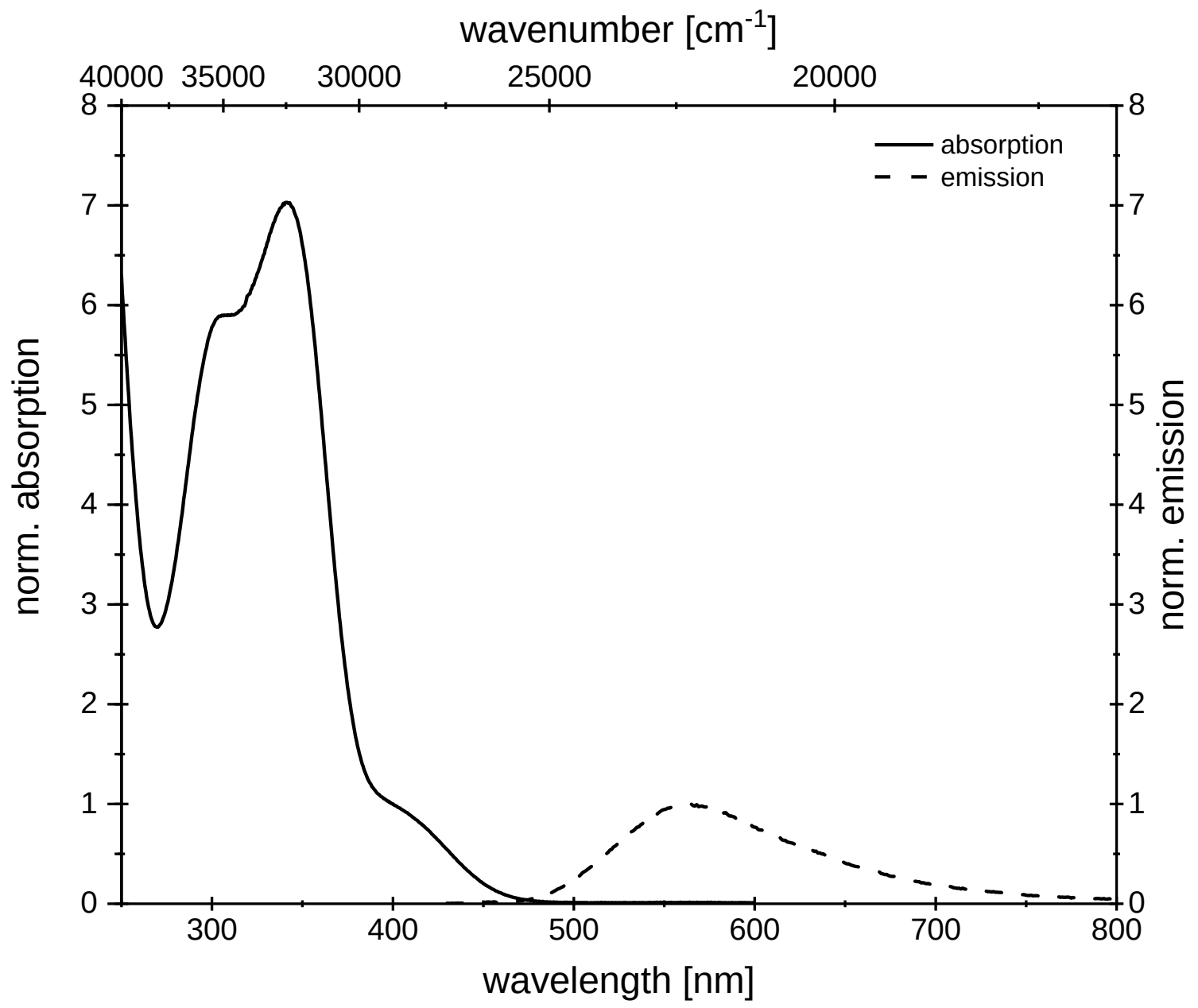

Figure S90. Absorption (solid line) and emission (dashed line) of compound **6d** (recorded in 1,4-dioxane, T = 293 K, c_{abs}(**6d**) = 10^{-5} M, c_{em}(**6d**) = 10^{-7} M, $\lambda_{ex} = \lambda_{max,abs}$).

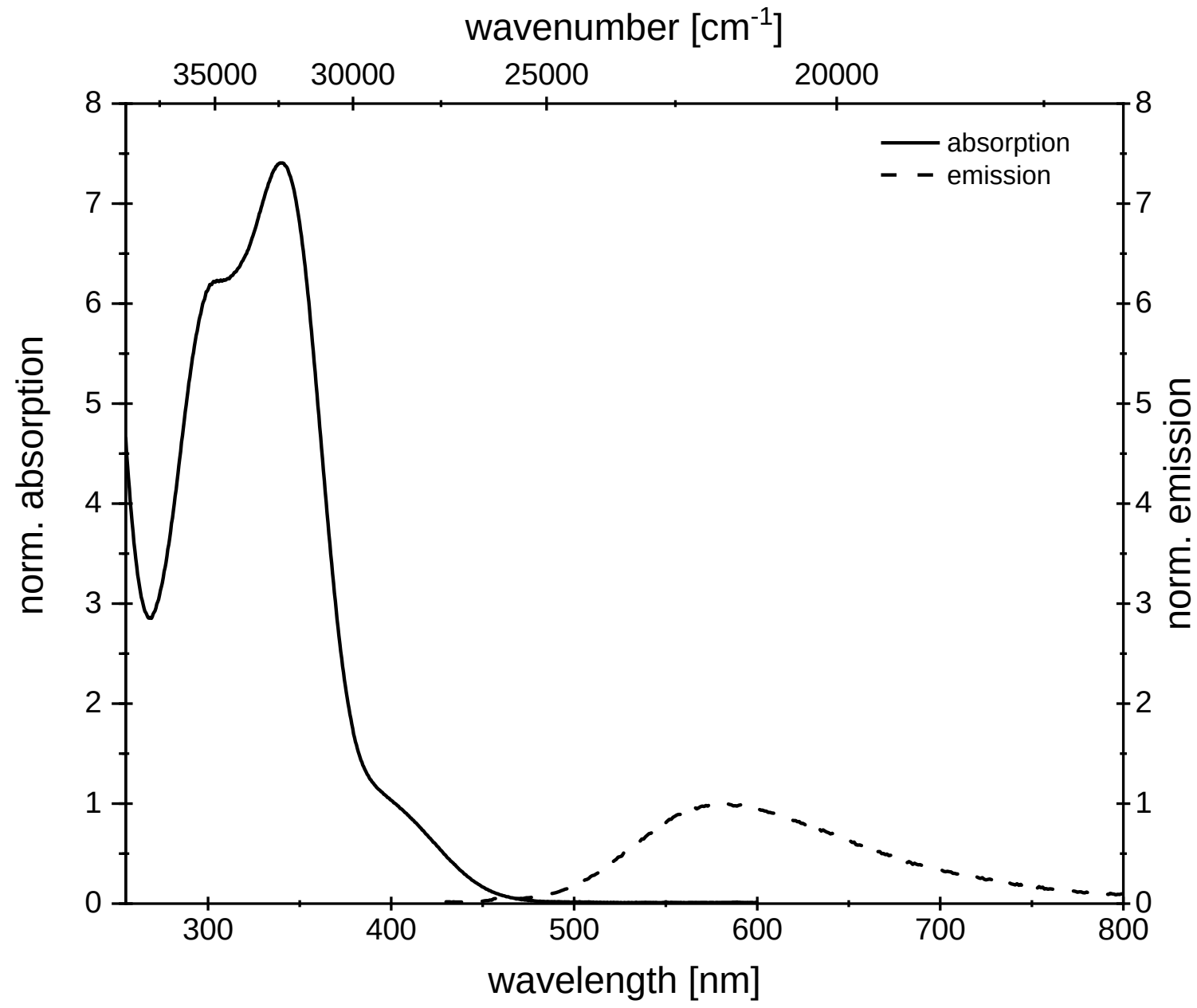

Figure S91. Absorption (solid line) and emission (dashed line) spectra of compound **6d** (recorded in ethyl acetate, T = 293 K, c_{abs}(**6d**) = 10^{-5} M, c_{em}(**6d**) = 10^{-7} M, $\lambda_{ex} = \lambda_{max,abs}$).

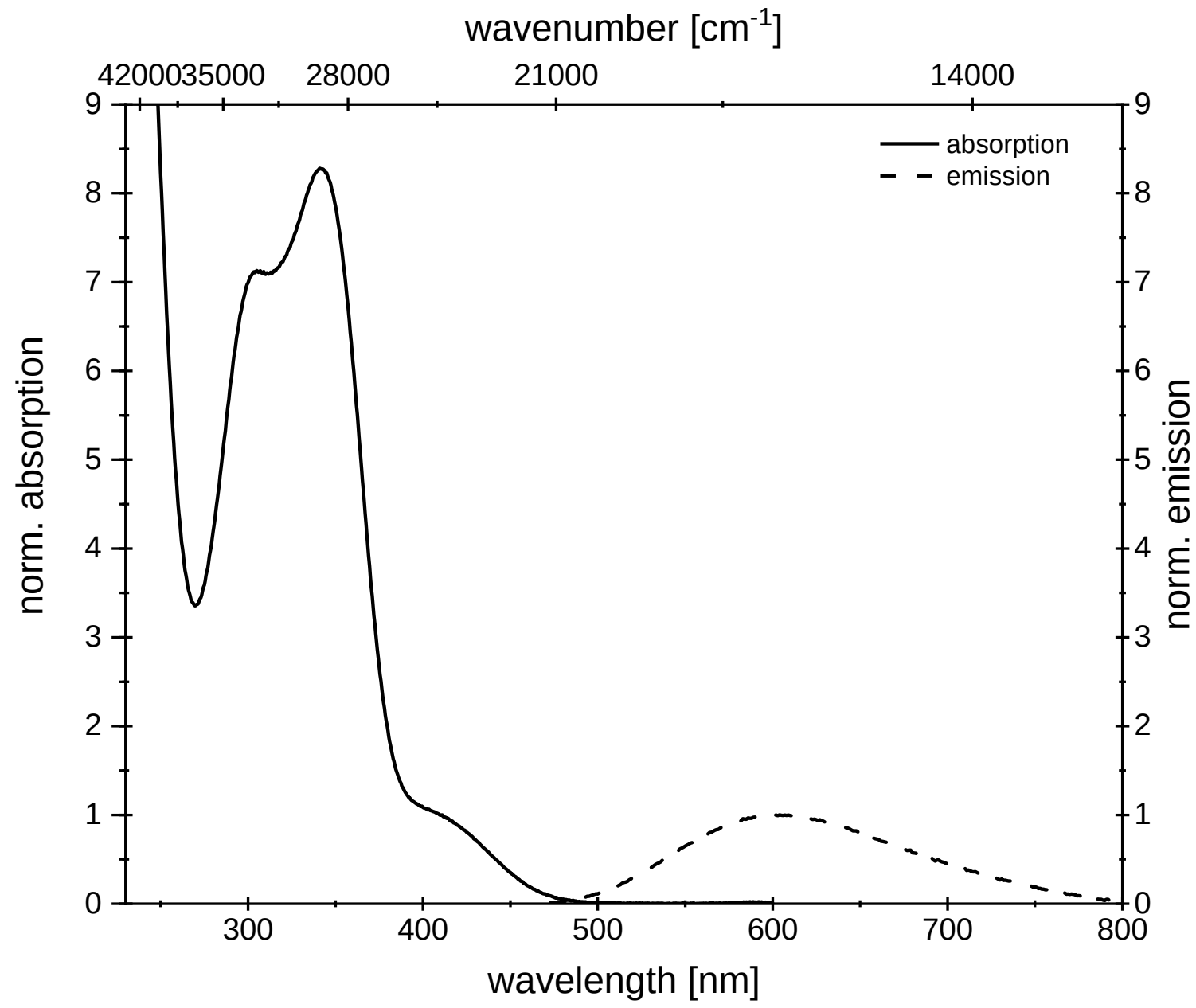

Figure S92. Absorption (solid line) and emission (dashed line) spectra of compound **6d** (recorded in dichloromethane, T = 293 K, c_{abs}(**6d**) = 10^{-5} M, c_{em}(**6d**) = 10^{-7} M, $\lambda_{ex} = \lambda_{max,abs}$).

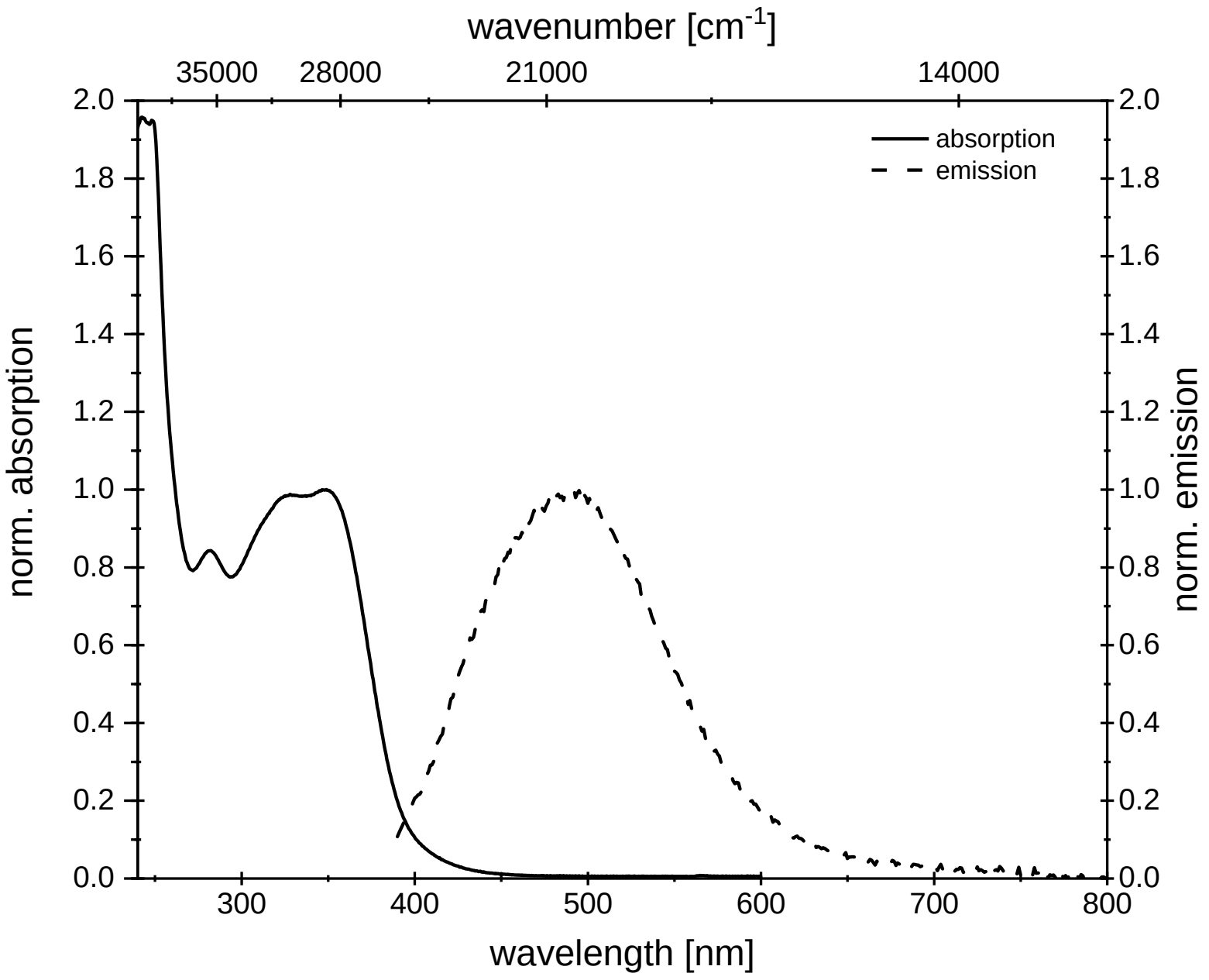

Figure S93. Absorption (solid line) and emission (dashed line) spectra of compound **6e** (recorded in dichloromethane, T = 293 K, c_{abs}(**6e**) = 10^{-5} M, c_{em}(**6e**) = 10^{-7} M, $\lambda_{ex} = \lambda_{max,abs}$).

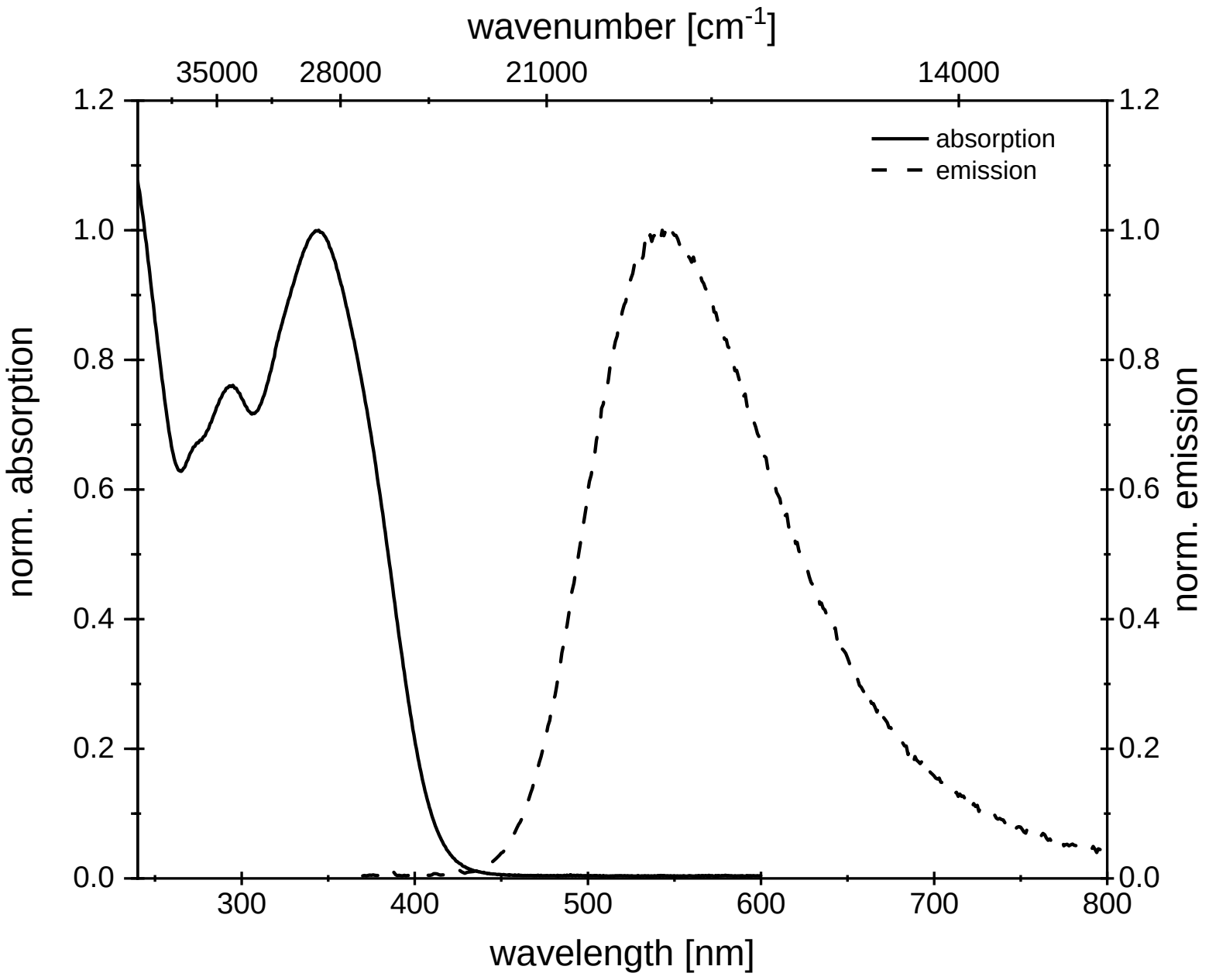

Figure S94. Absorption (solid line) and emission (dashed line) of compound **7a** (recorded in dichloromethane, T = 293 K, c_{abs}(**7a**) = 10^{-5} M, c_{em}(**7a**) = 10^{-7} M, $\lambda_{ex} = \lambda_{max,abs}$).

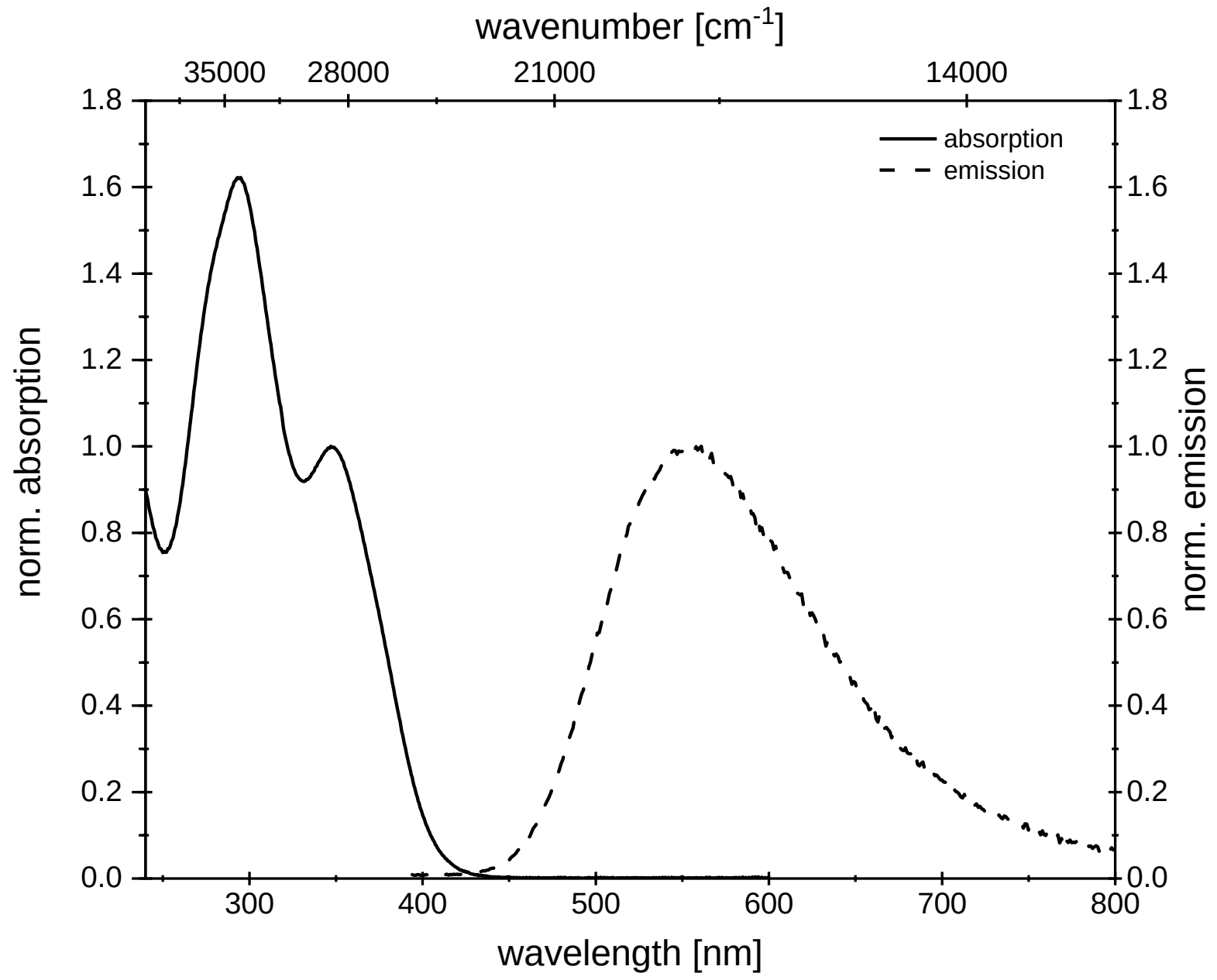

Figure S95. Absorption (solid line) and emission (dashed line) spectra of compound **7b** (recorded in dichloromethane, T = 293 K, c_{abs}(**7b**) = 10^{-5} M, c_{em}(**7b**) = 10^{-7} M, $\lambda_{ex} = \lambda_{max,abs}$).

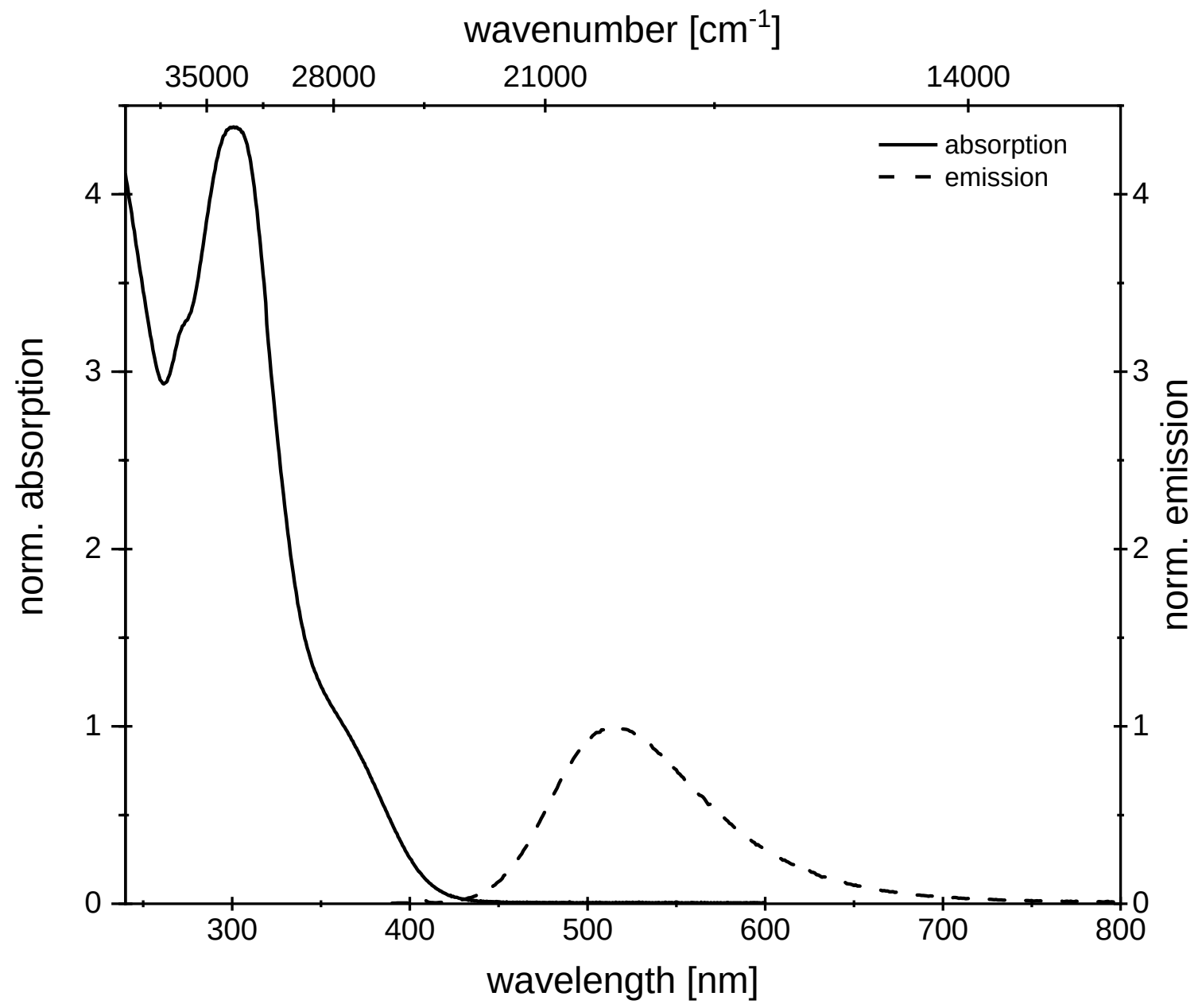

Figure S96. Absorption (solid line) and emission (dashed line) spectra of compound **7c** (recorded in dichloromethane, T = 293 K, c_{abs}(**7c**) = 10^{-5} M, c_{em}(**7c**) = 10^{-7} M, $\lambda_{ex} = \lambda_{max,abs}$).

6.2 Solid state emission spectra of *para-/meta-/ortho*-triarylamine dimers (*sym-p-/m-/o*-bTAA)$_2$ 4, 5, 6 and 7

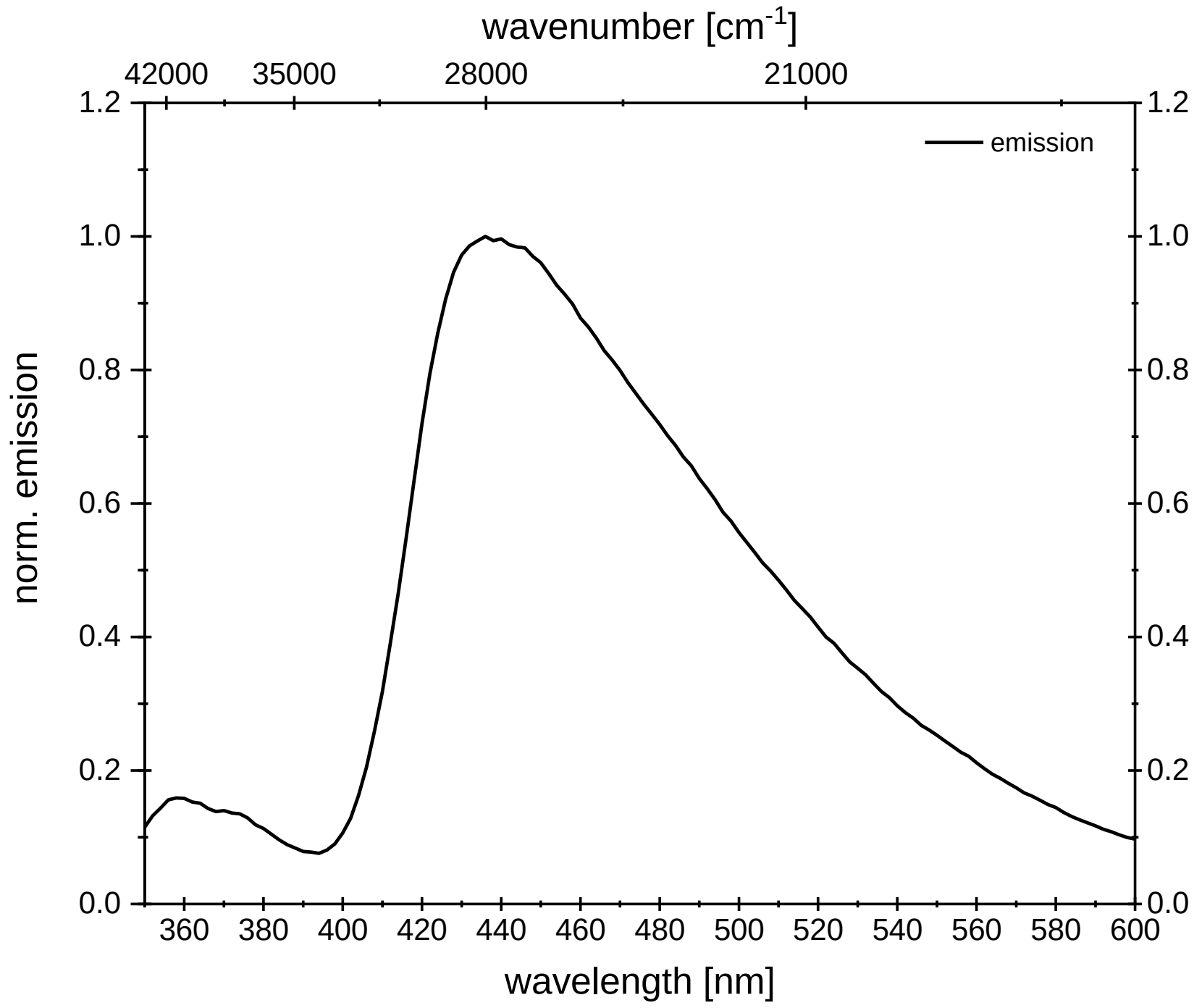

Figure S97. Solid state emission spectra of compound **4a** (recorded at, *T* = 293 K).

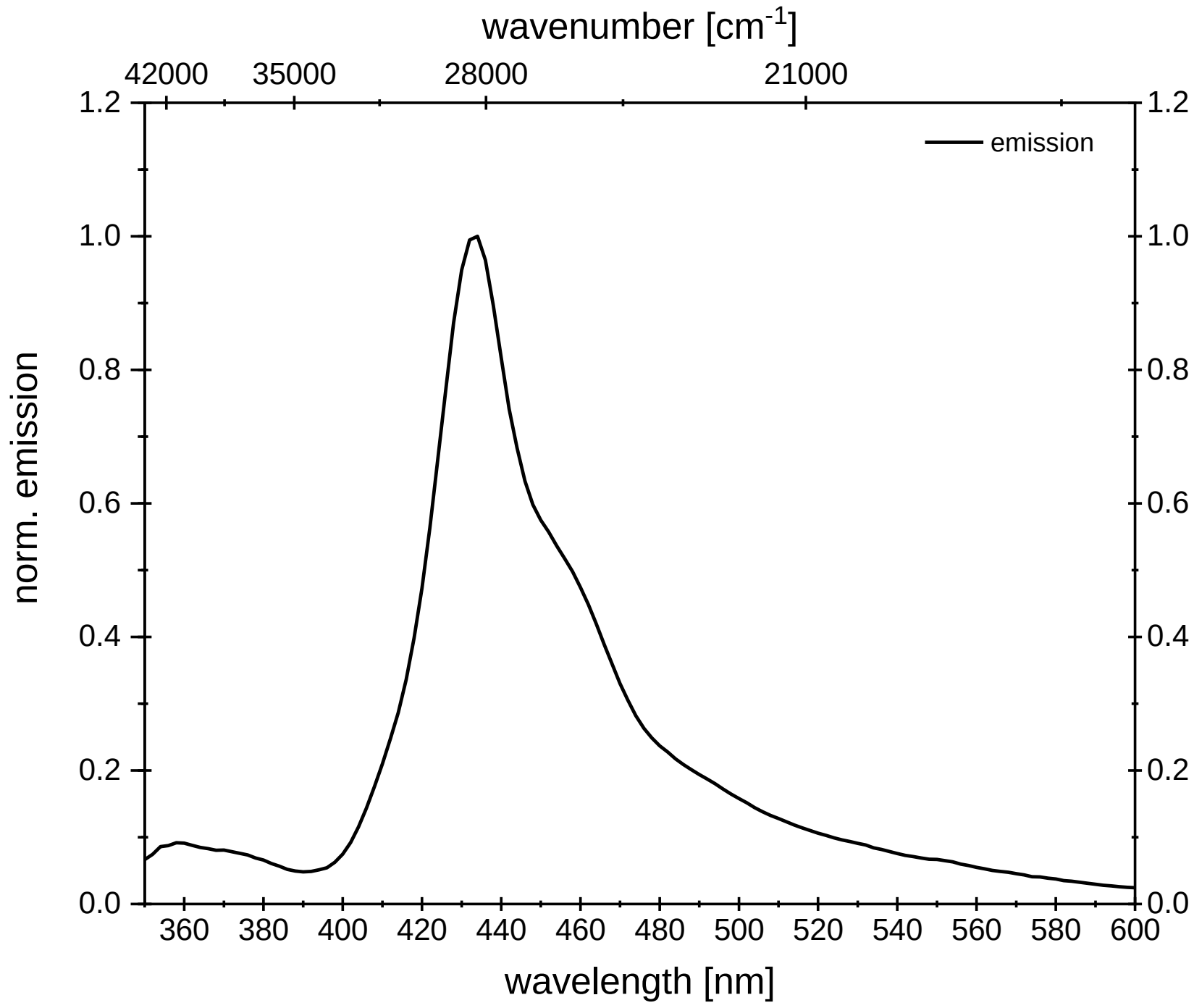

Figure S98. Solid state emission spectra of compound **4b** (recorded at *T* = 293 K).

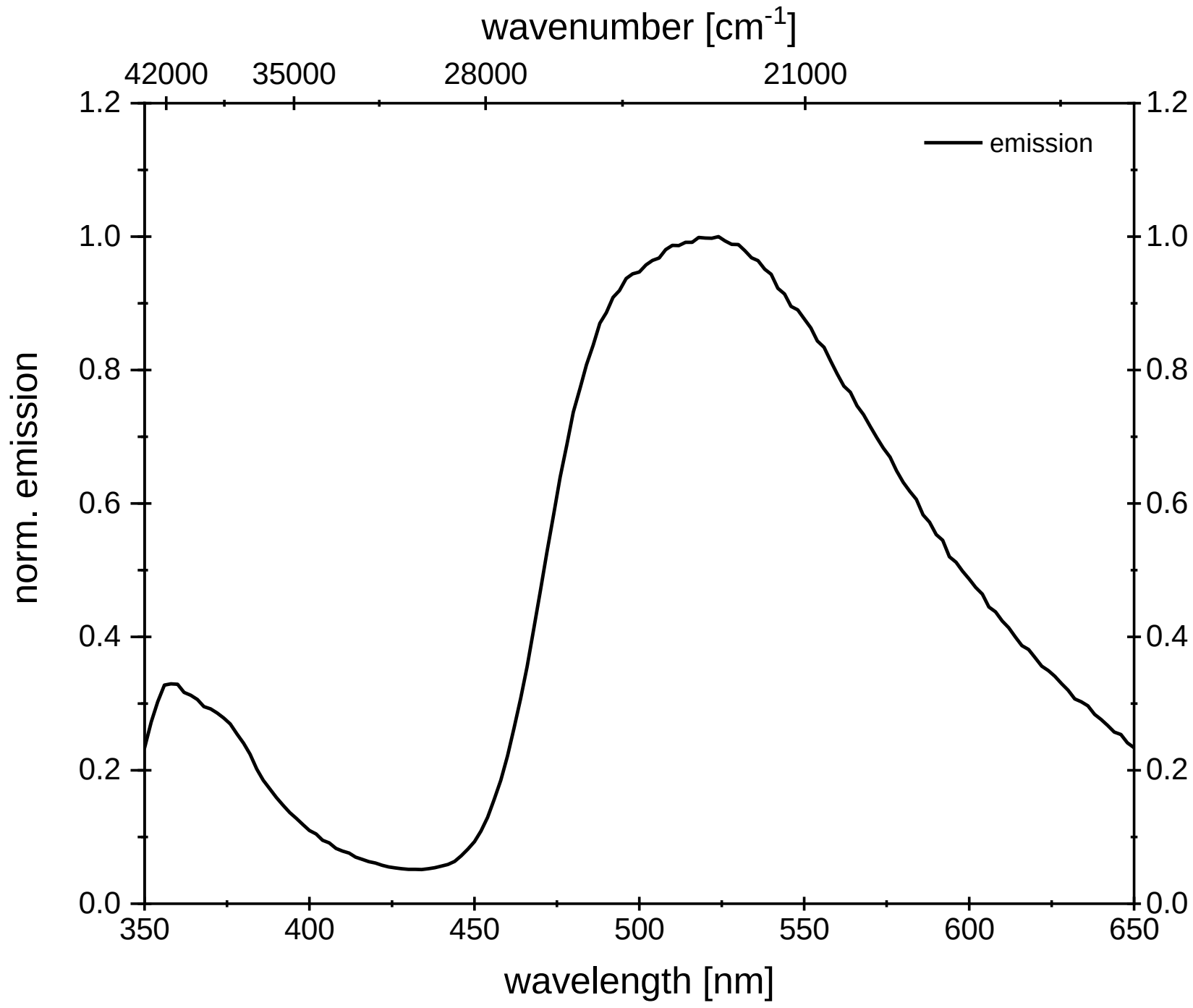

Figure S99. Solid state emission spectra of compound **4c** (recorded at *T* = 293 K).

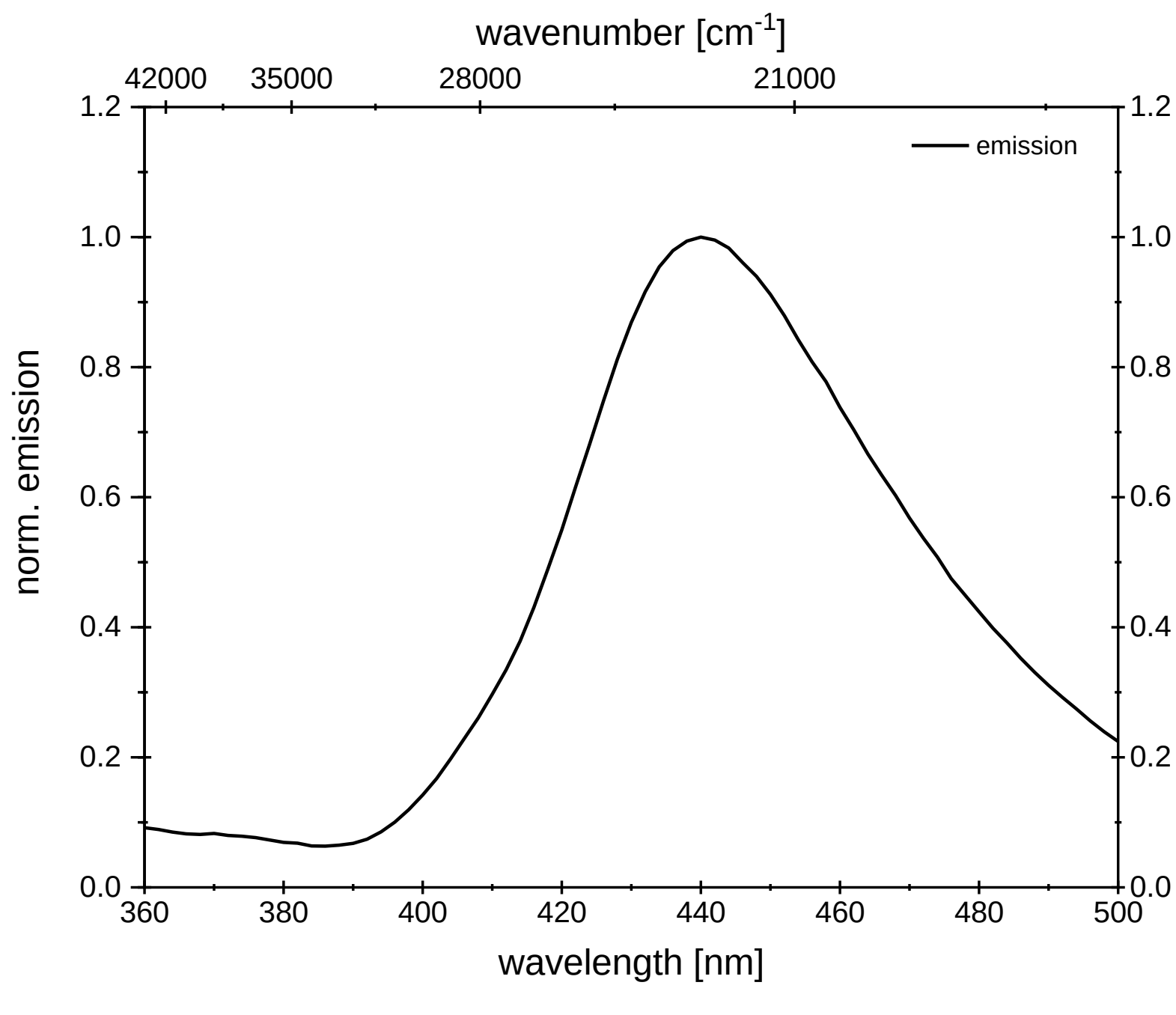

Figure S100. Solid state emission spectra of compound mixture **5a/5a'/5a''** (recorded at *T* = 293 K).

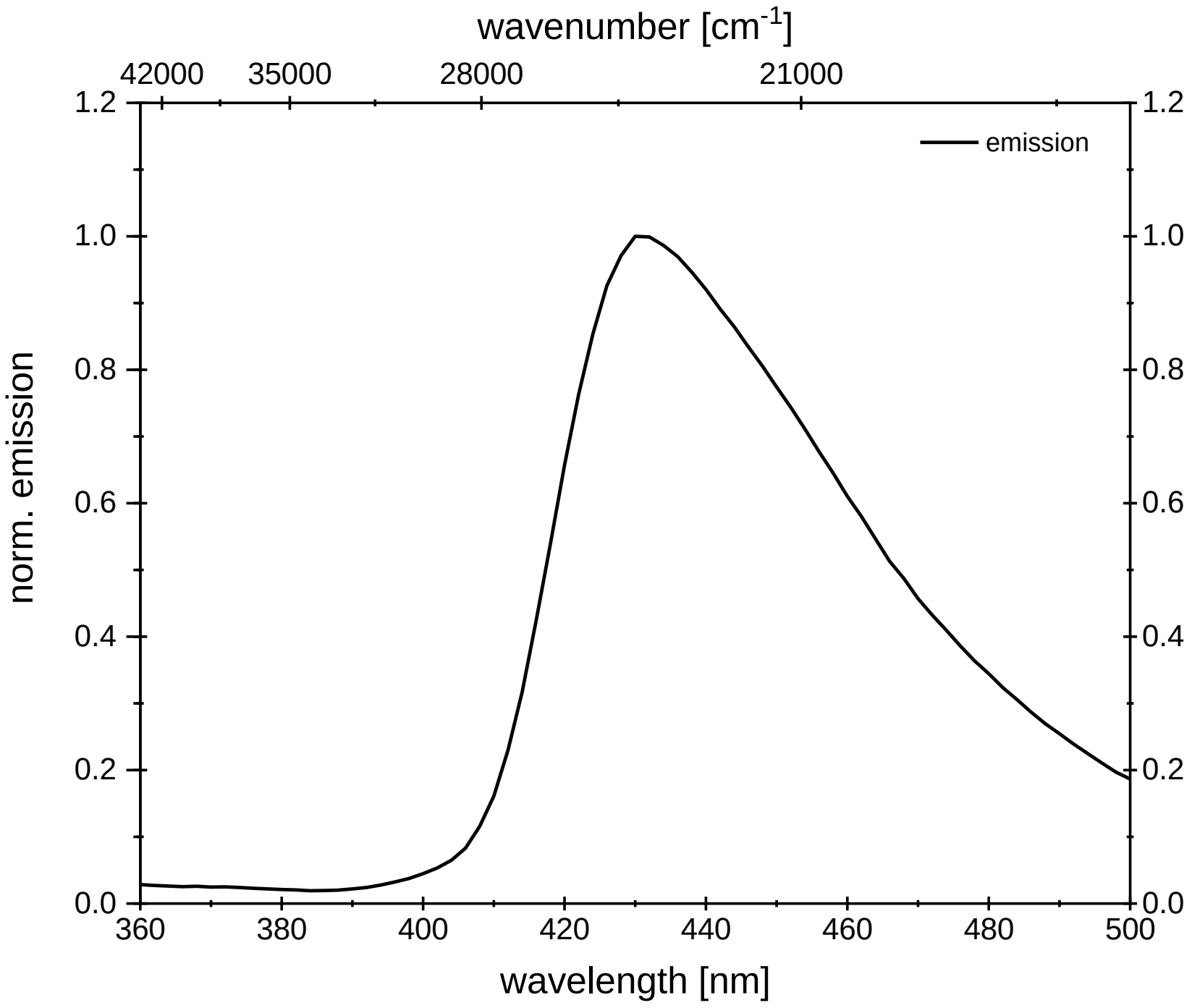

Figure S101. Solid state emission spectra of compound mixture **5b/5b'/5b''** (recorded at T = 293 K).

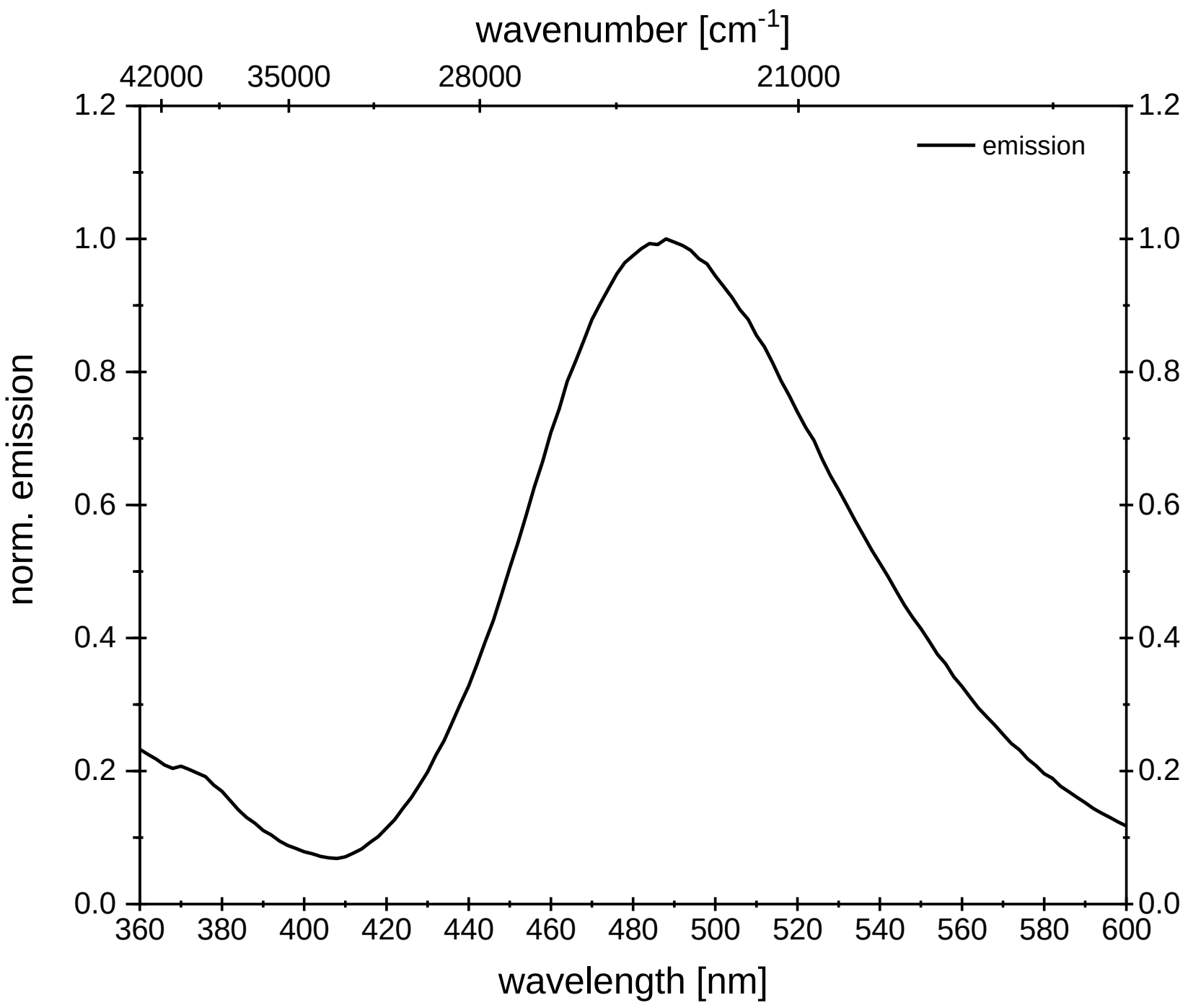

Figure S102. Solid state emission spectra of compound **5c** (recorded at T = 293 K).

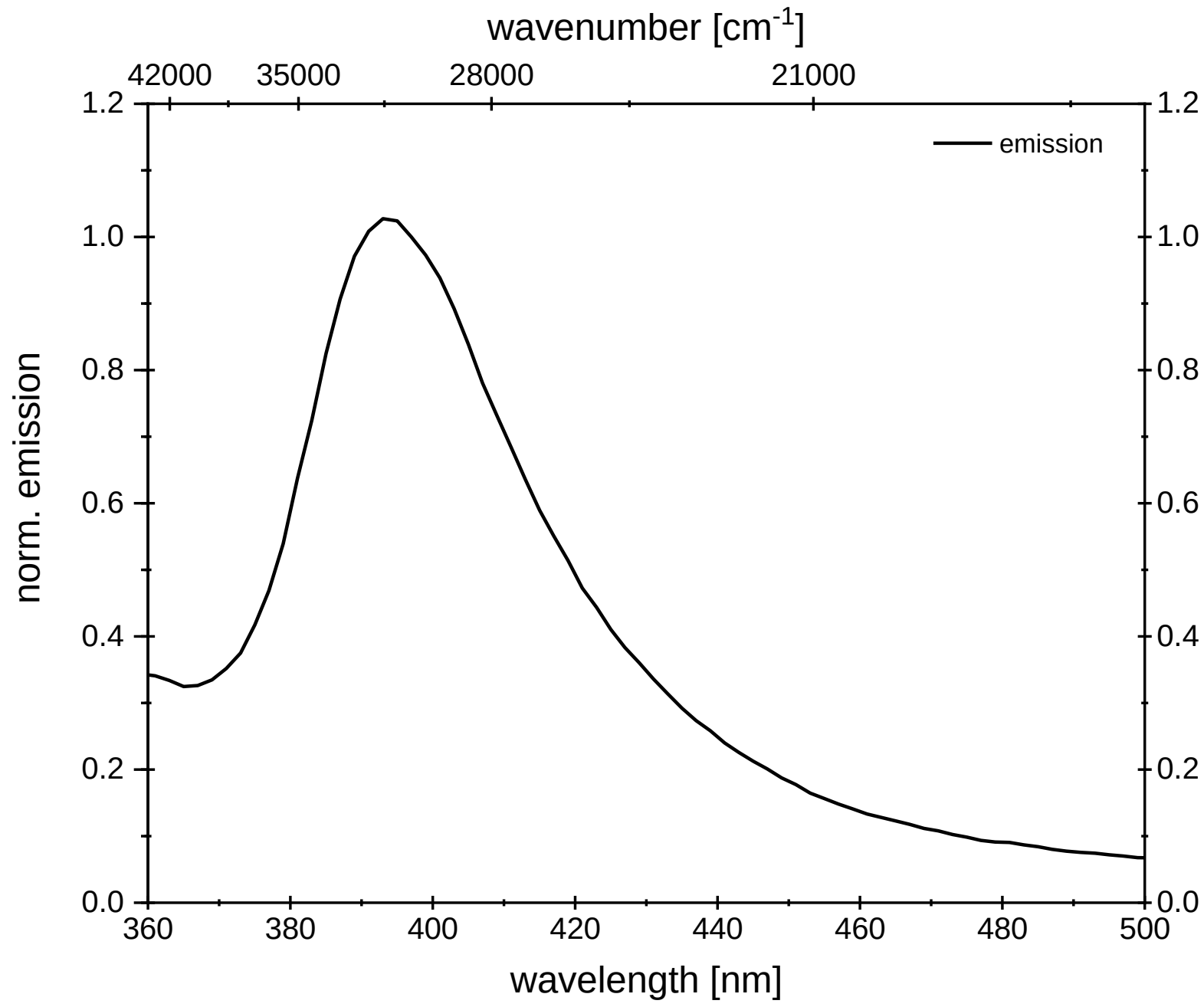

Figure S103. Solid state emission spectra of compound **5d** (recorded at T = 293 K).

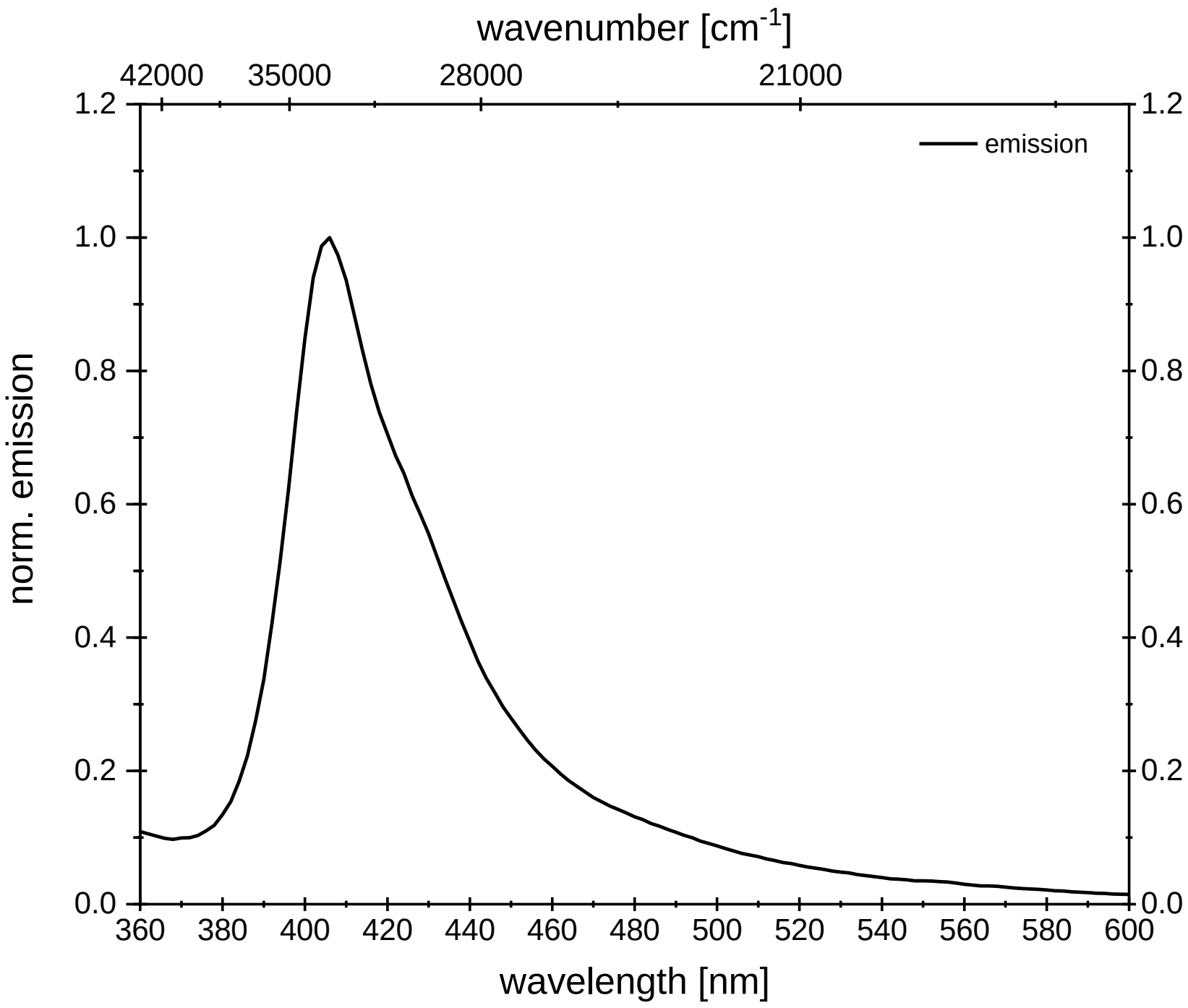

Figure S104. Solid state emission spectra of compound **6a** (recorded at T = 293 K).

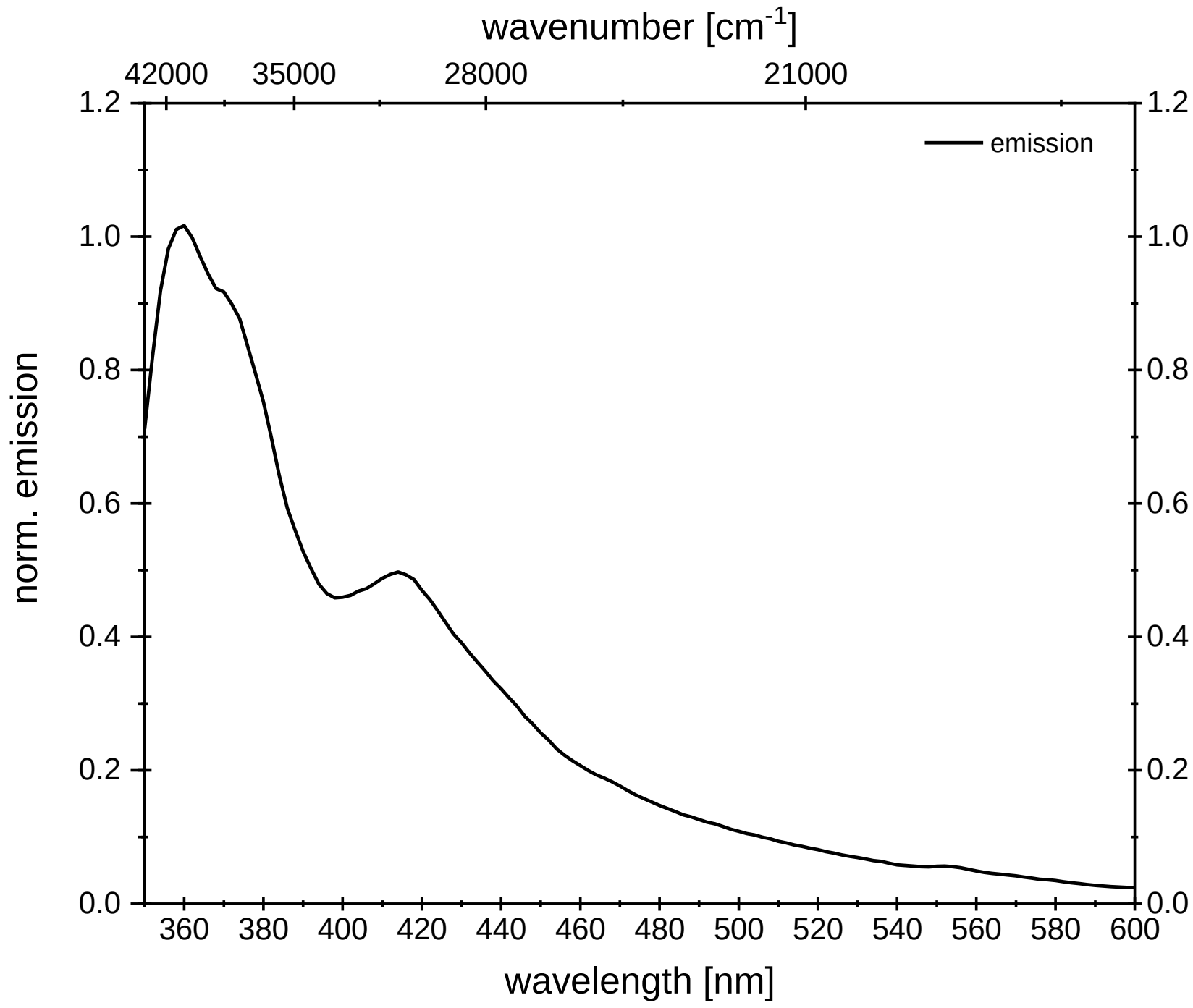

Figure S105. Solid state emission spectra of compound **6b** (recorded at *T* = 293 K).

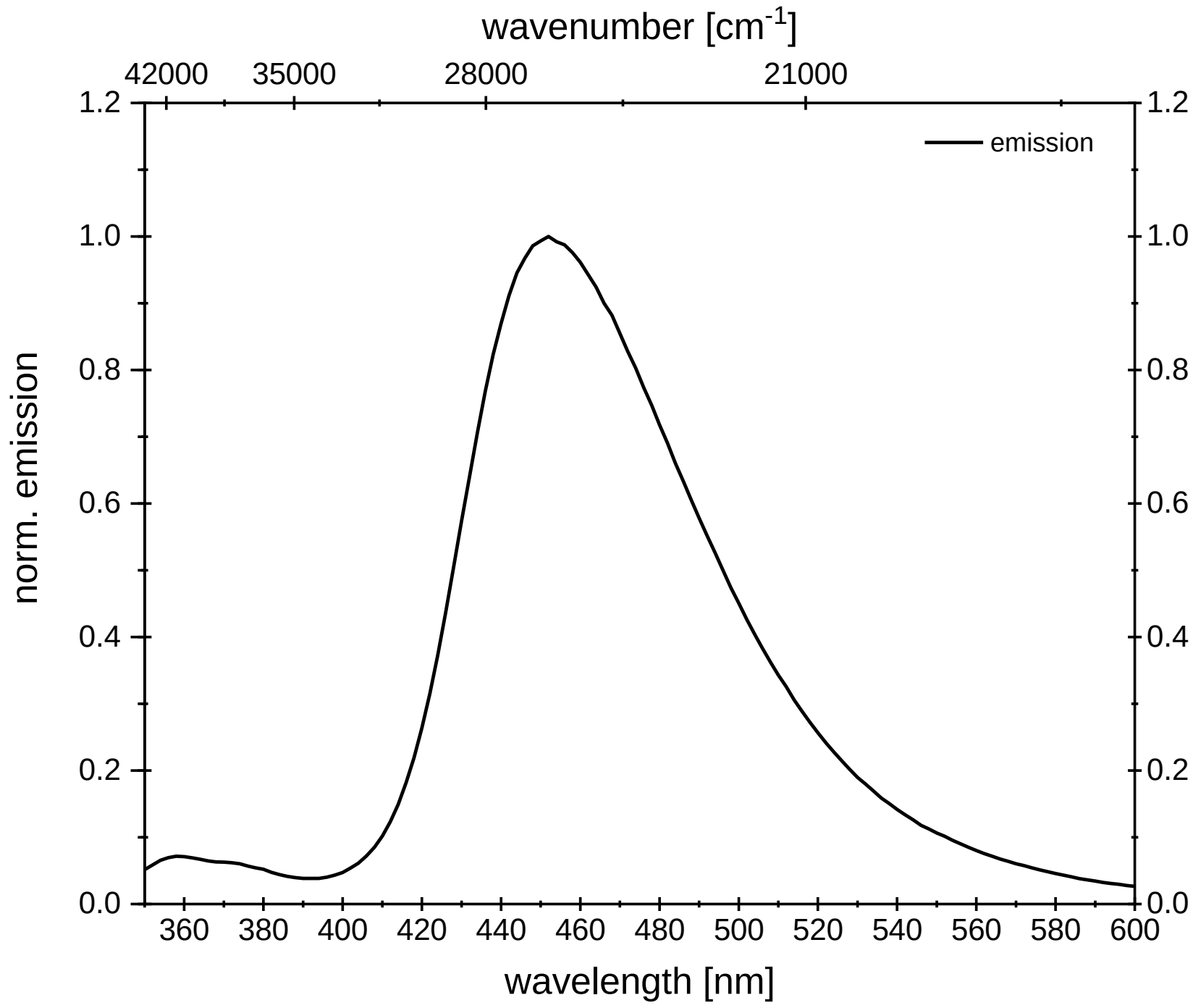

Figure S106. Solid state emission spectra of compound **6c** (recorded at *T* = 293 K).

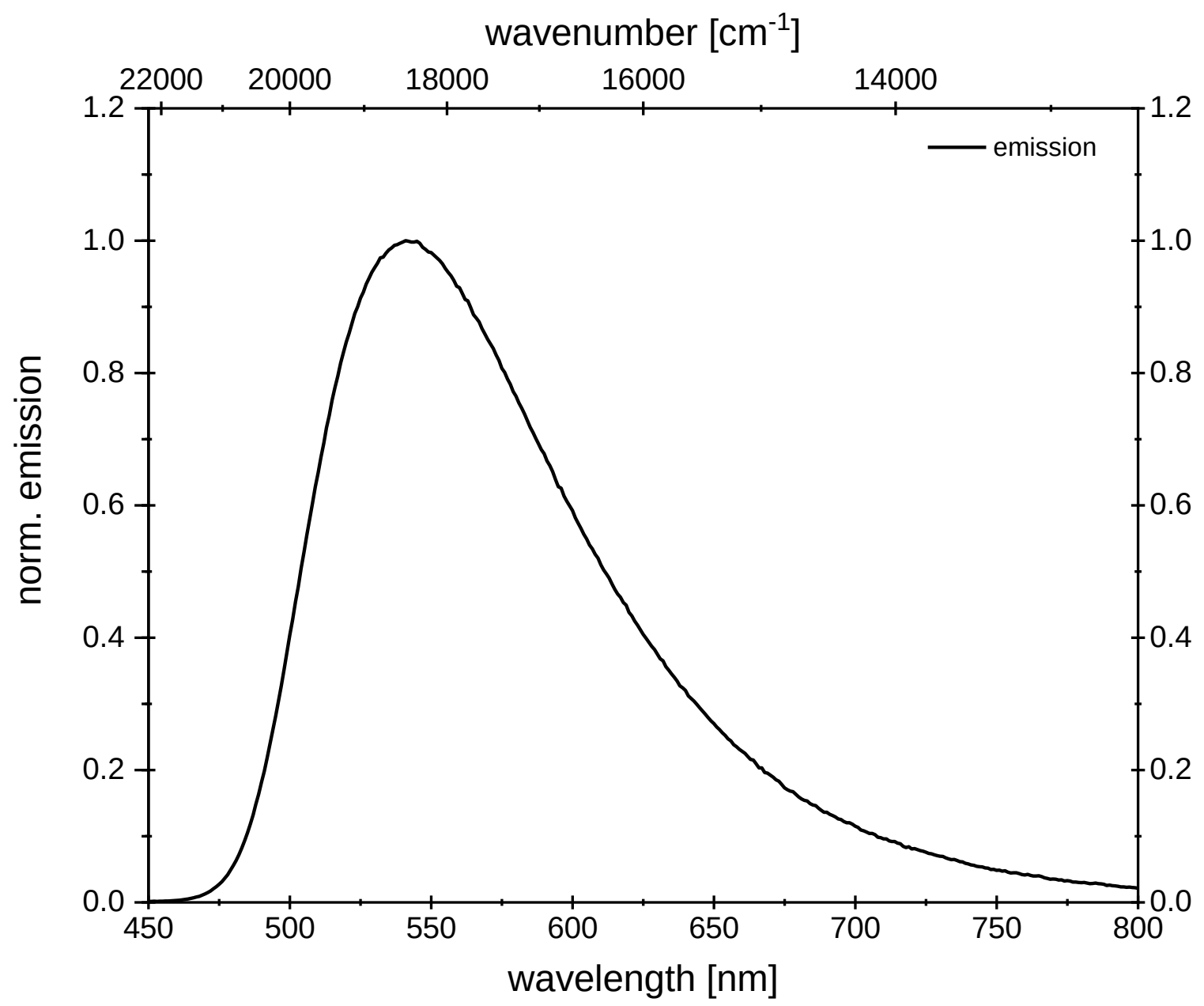

Figure S107. Solid state emission spectra of compound **6d** (recorded at *T* = 293 K).

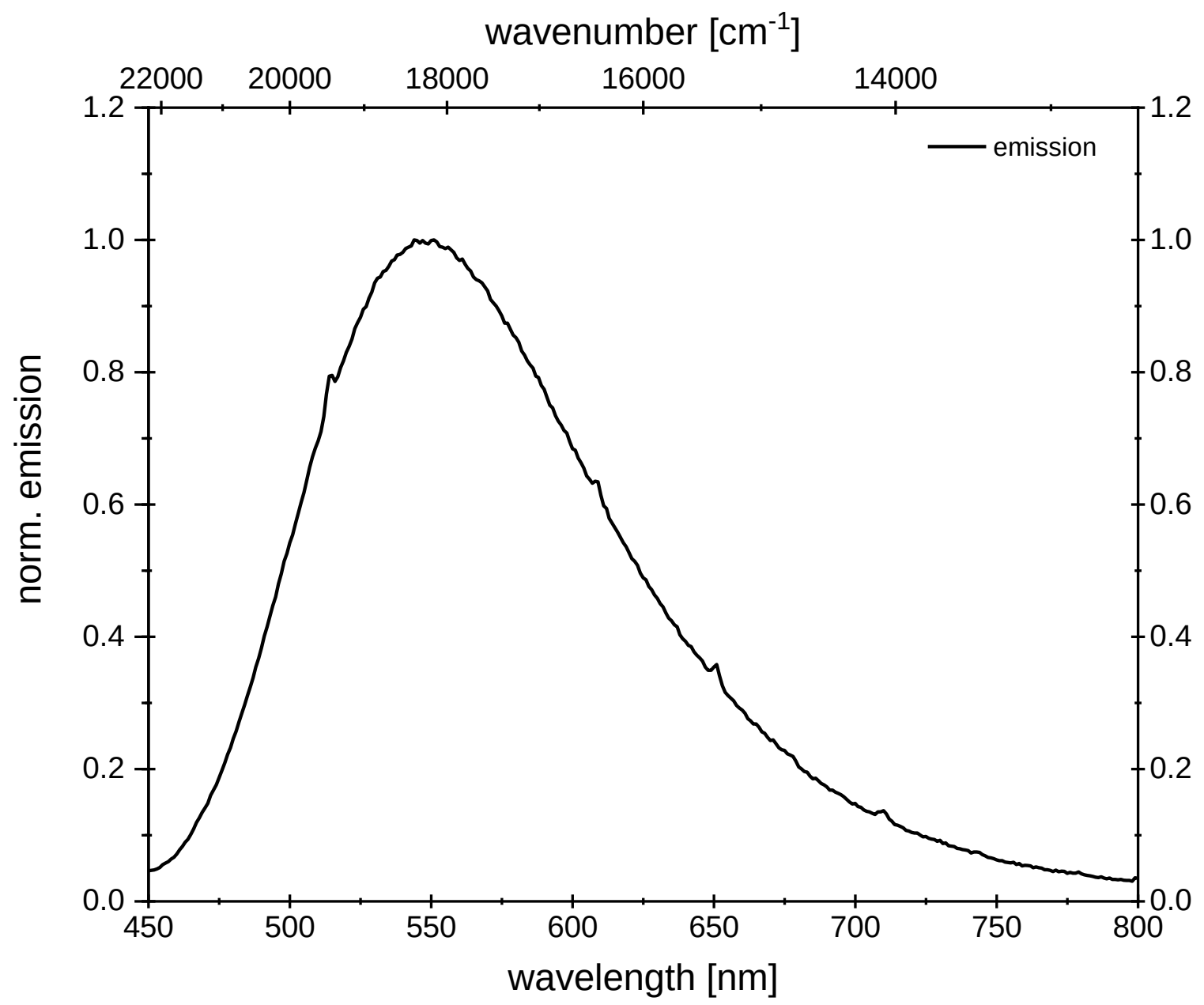

Figure S108. Solid state emission spectra of compound **6e** (recorded at *T* = 293 K).

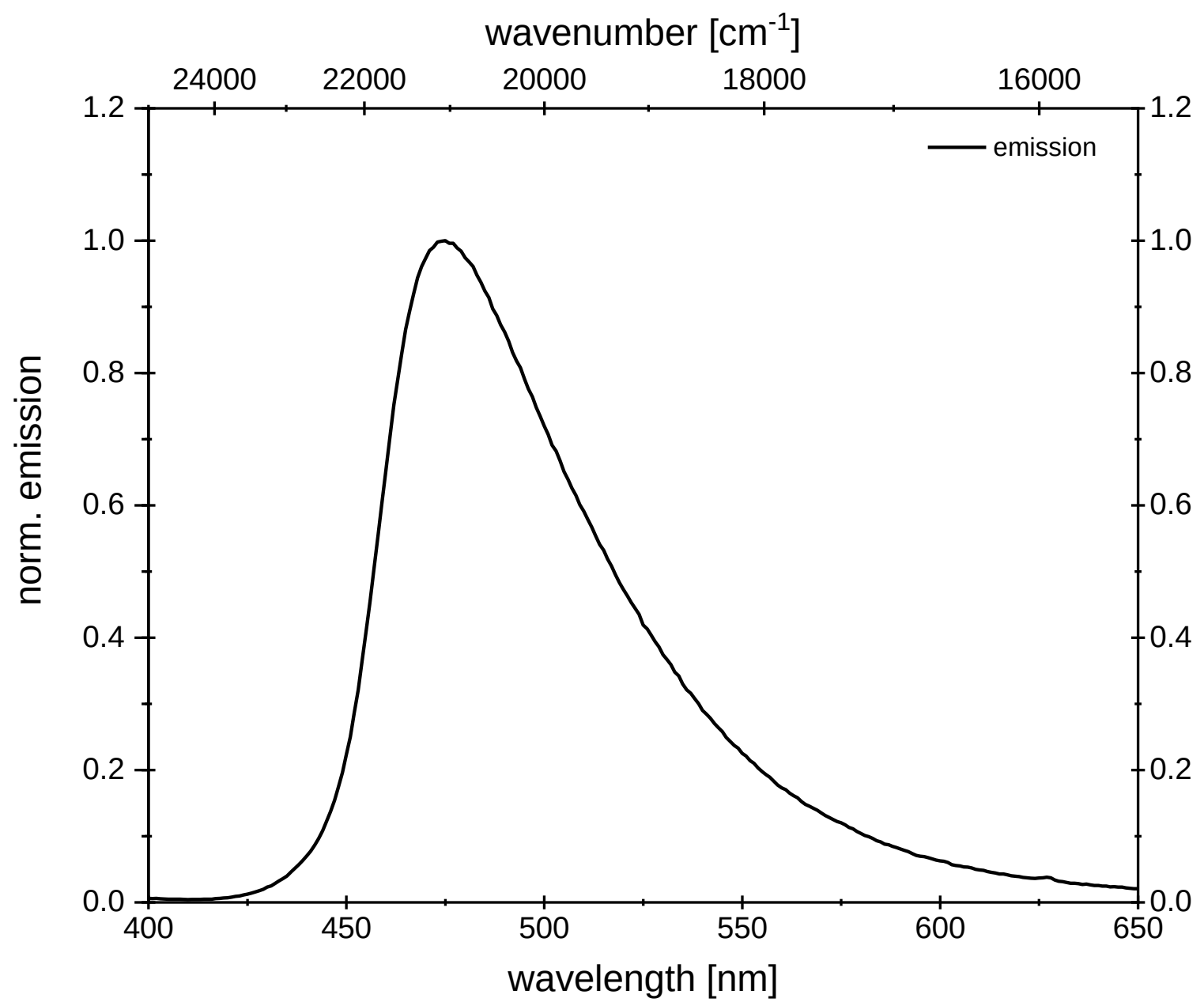

Figure S109. Solid state emission spectra of compound **7a** (recorded at *T* = 293 K).

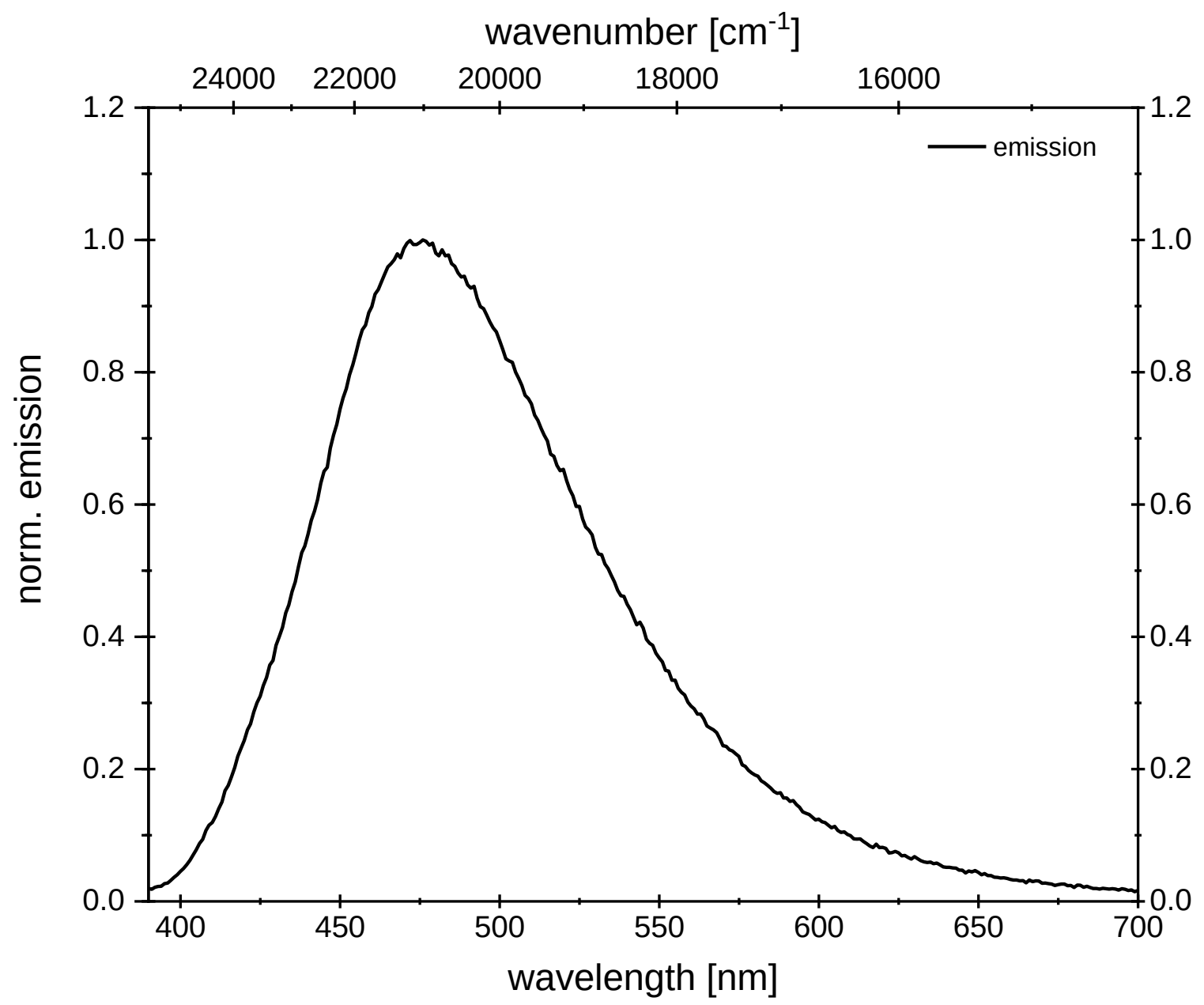

Figure S110. Solid state emission spectra of compound **7b** (recorded at *T* = 293 K).

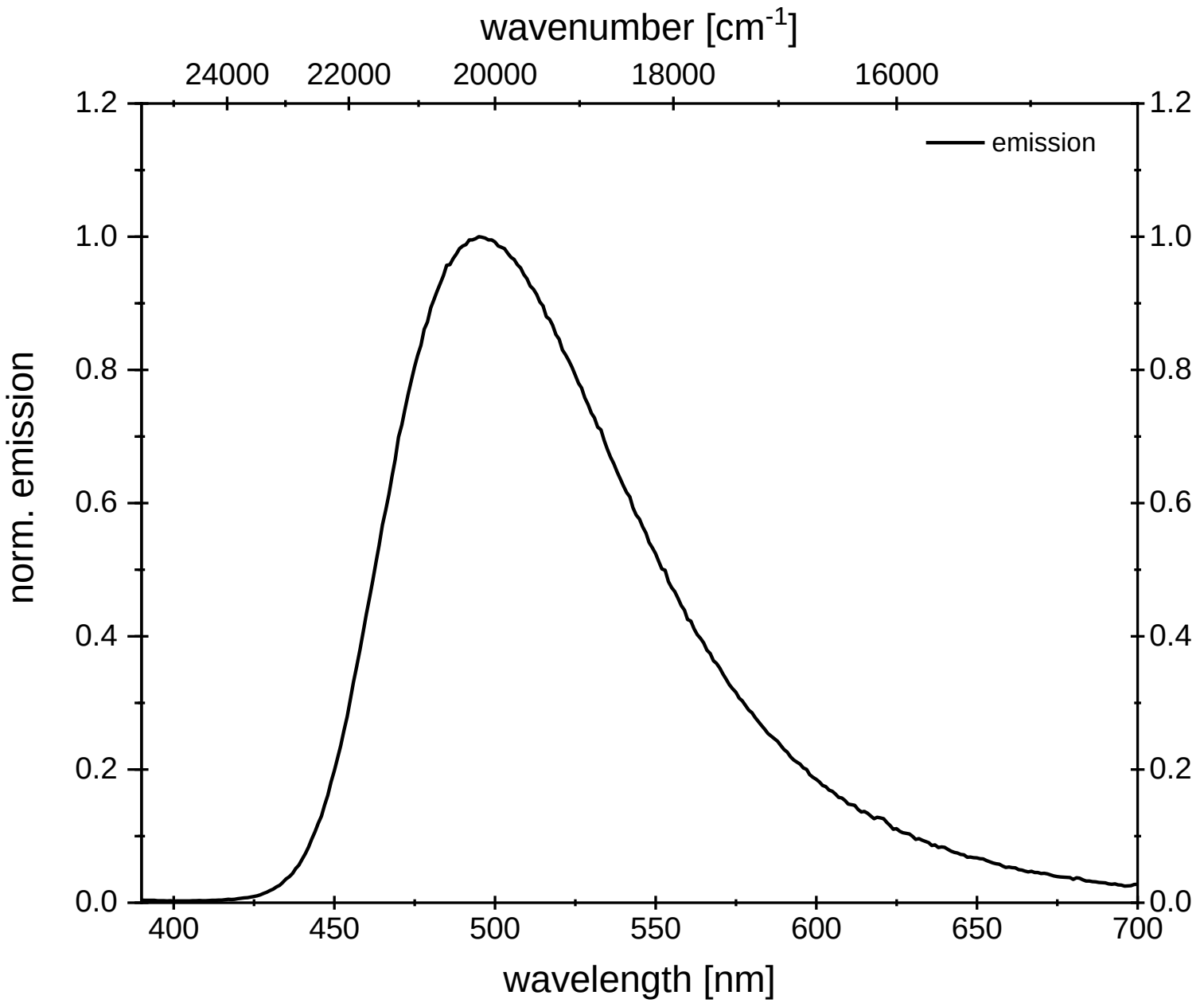

Figure S111. Solid state emission spectra of compound **7c** (recorded at *T* = 293 K).

6.2.1 Solid state emission spectra of 6d and 6e in comparison to their spectra in solution

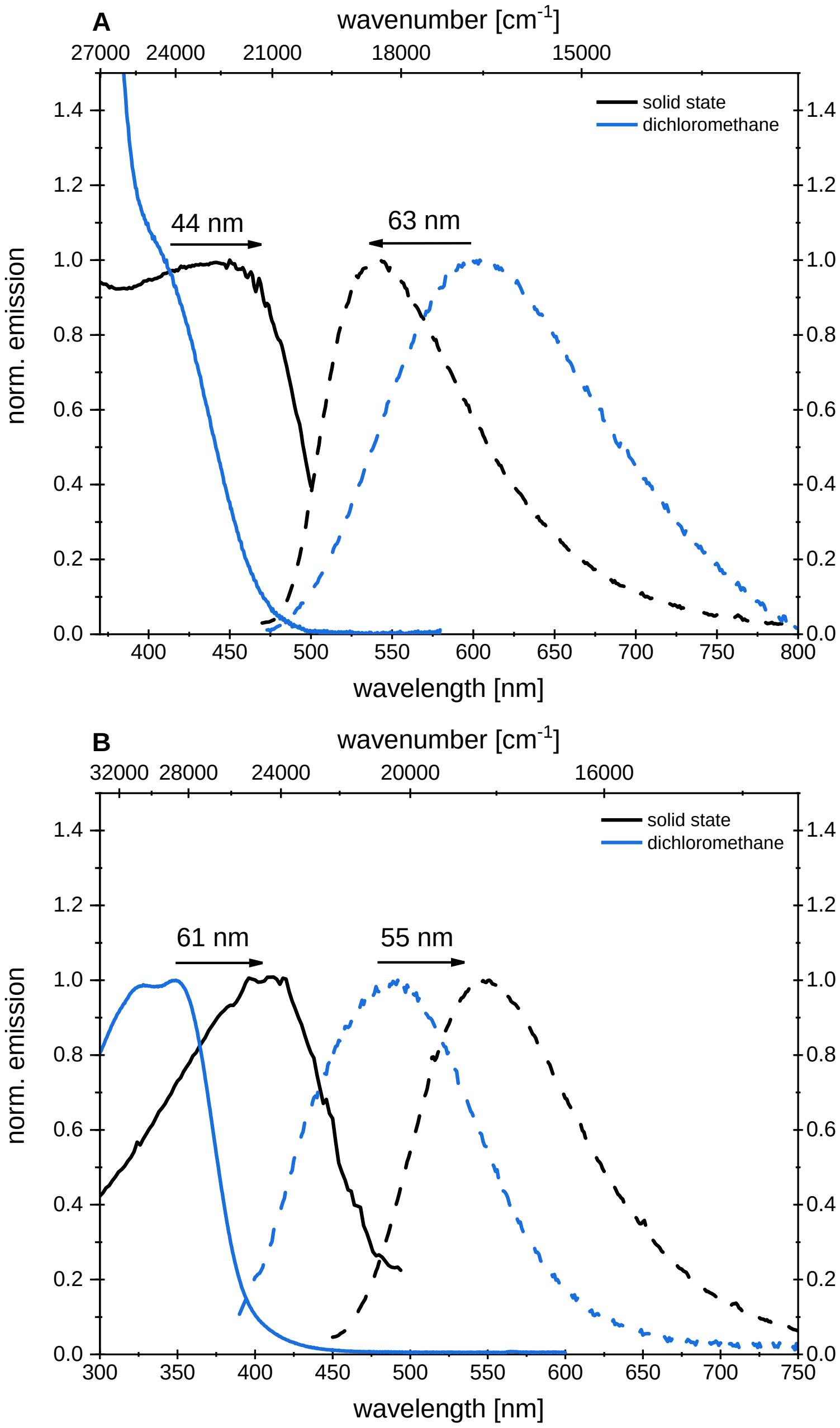

Figure S112. Comparison of solid state excitation (black, solid line) and emission (black, dashed line) spectra with absorption (blue, solid line) and emission spectra (blue, dashed line) recorded in dichloromethane of compound A: **6d** and B: **6e** (recorded at T = 293 K, c_{abs}(**6d**–**e**) = 10^{-5} M, c_{em}(**6d**–**e**) = 10^{-7} M, $\lambda_{ex} = \lambda_{max,abs}$).

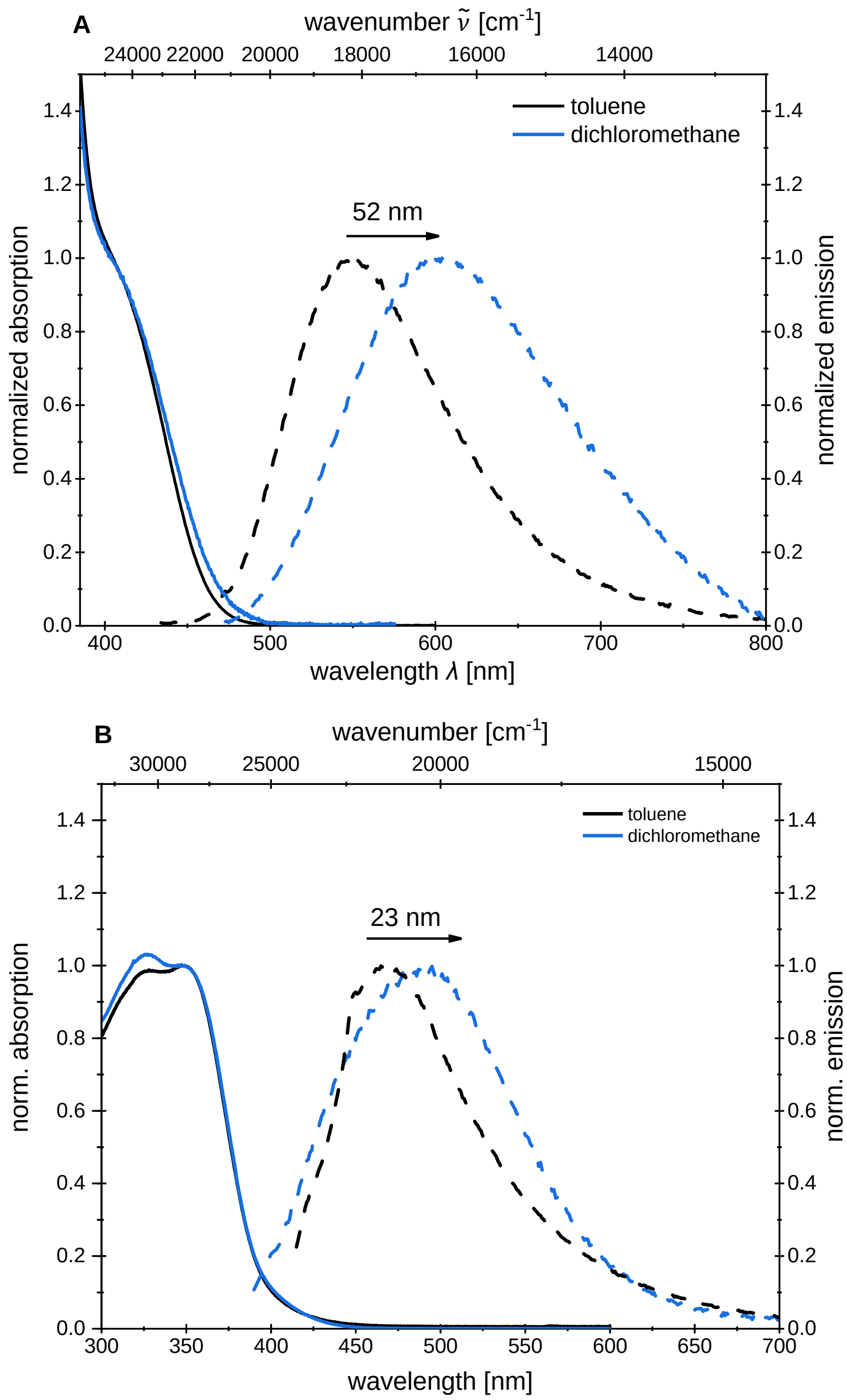

Figure S113. Comparison of absorption (solid line) and emission (dashed line) spectra of compound A: **6d** and B: **6e** recorded in toluene (black line) and dichloromethane (blue line) (recorded at T = 293 K, c_{abs}(**6d**–**e**) = 10^{-5} M, c_{em}(**6d**–**e**) = 10^{-7} M, $\lambda_{ex} = \lambda_{max,abs}$).

6.3 PMMA matrix emission spectra of *ortho*-triarylamine dimers (*sym-o*-bTAA)$_2$ 6d, 6e and 7

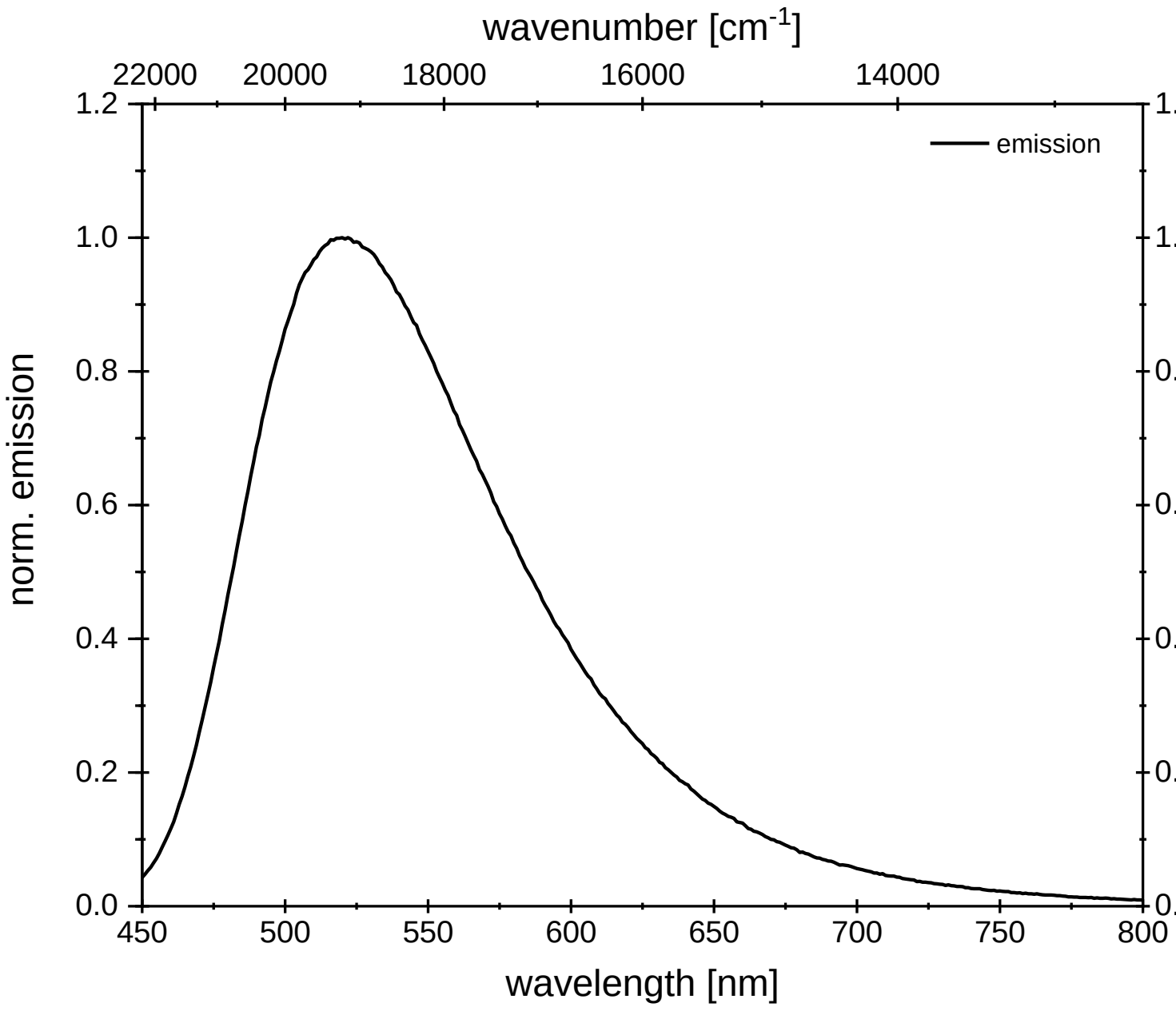

Figure S114. PMMA matrix emission spectra of compound **6d** (recorded at *T*= 293 K).

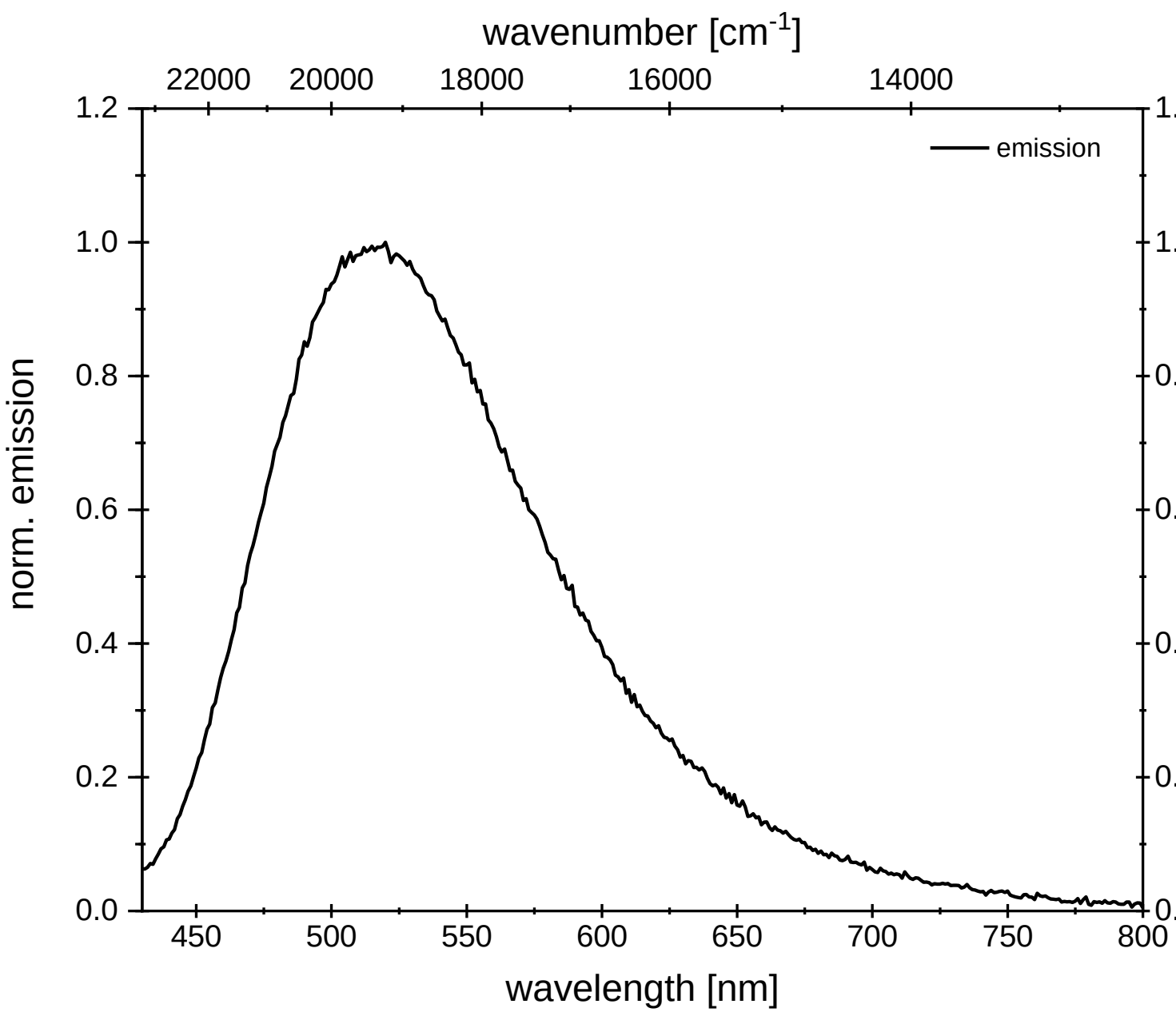

Figure S115. PMMA matrix emission spectra of compound **6e** (recorded at *T* = 293 K).

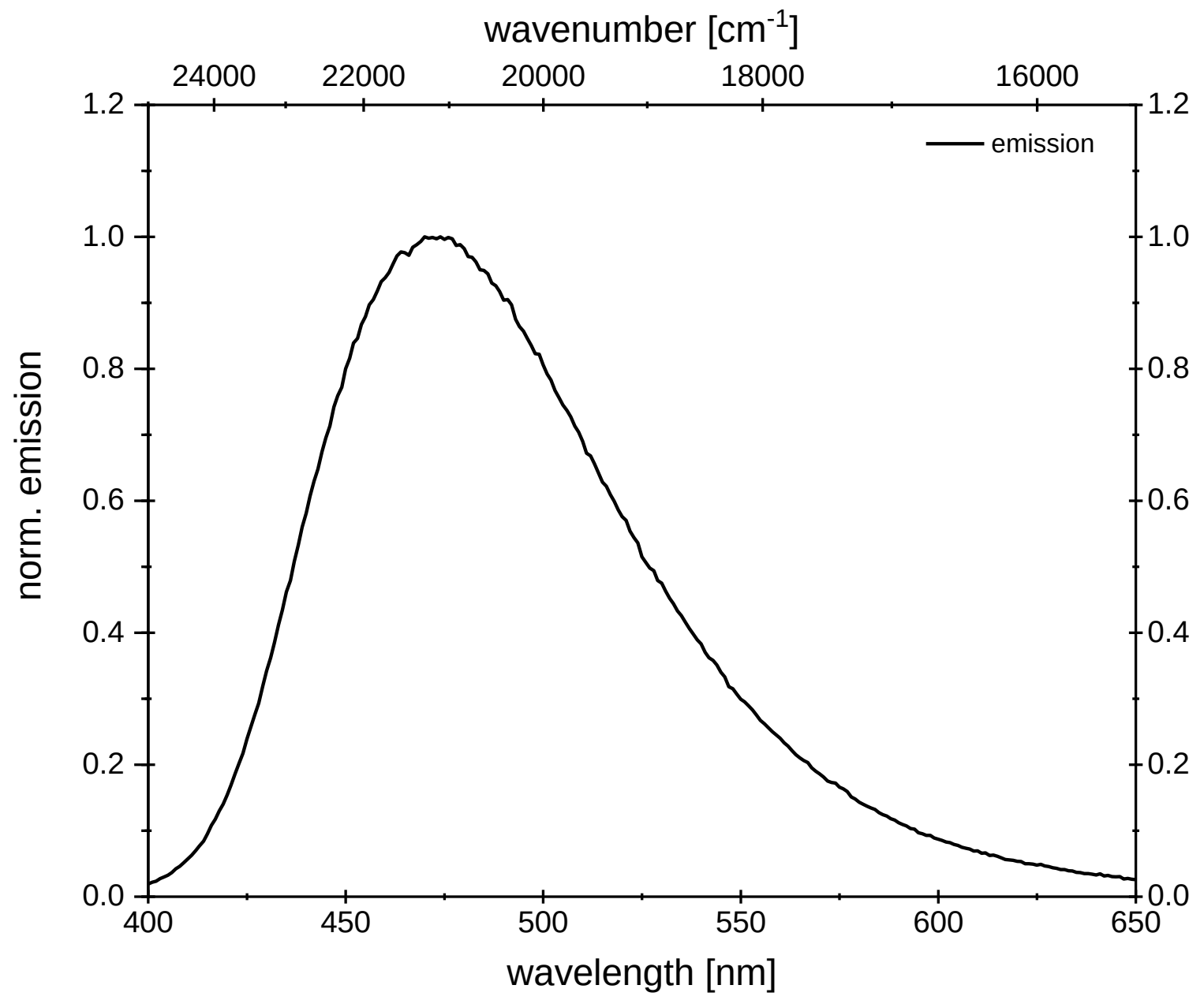

Figure S116. PMMA matrix emission spectra of compound **7a** (recorded at *T*= 293 K).

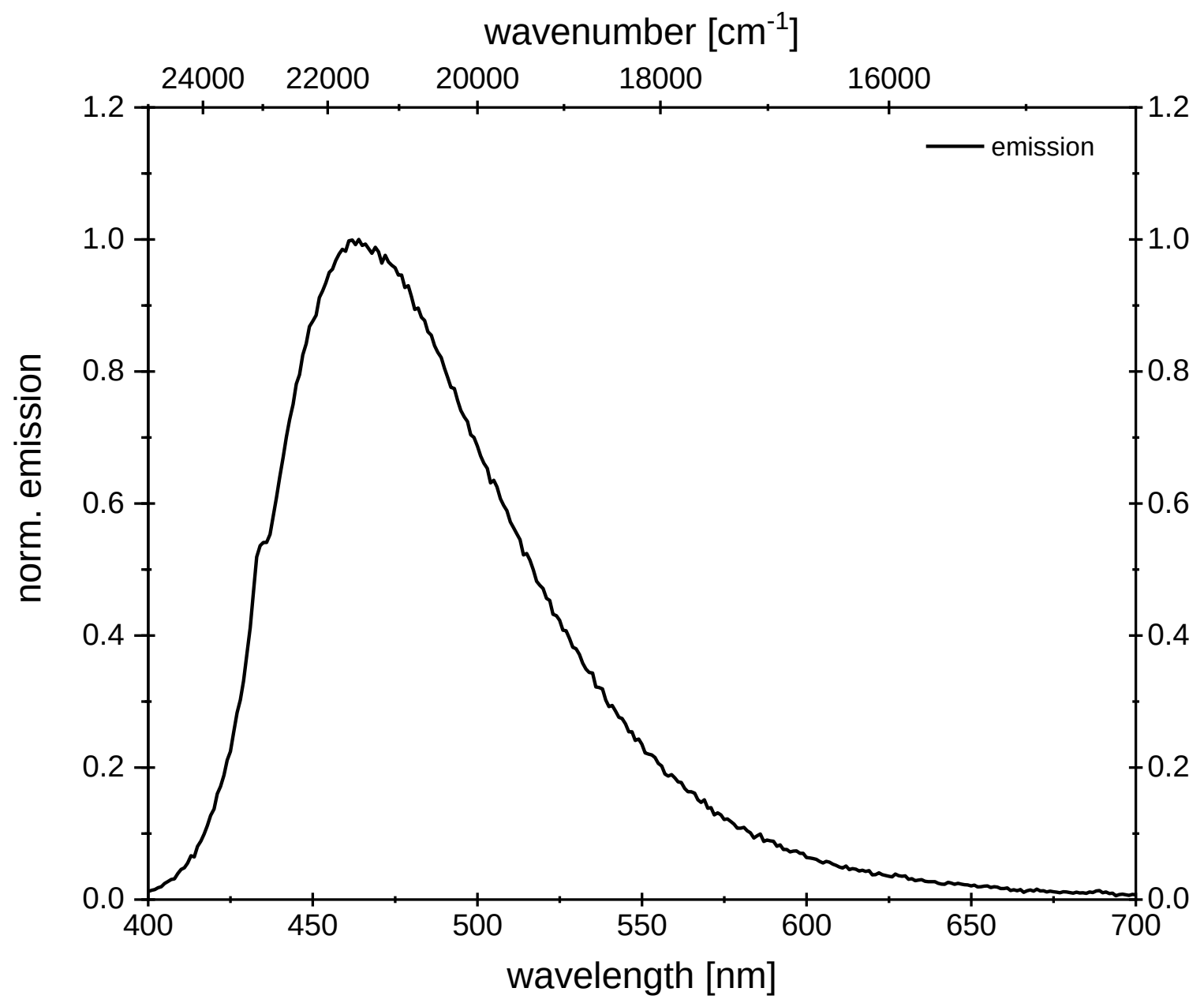

Figure S117. PMMA matrix emission spectra of compound **7b** (recorded at *T* = 293 K).

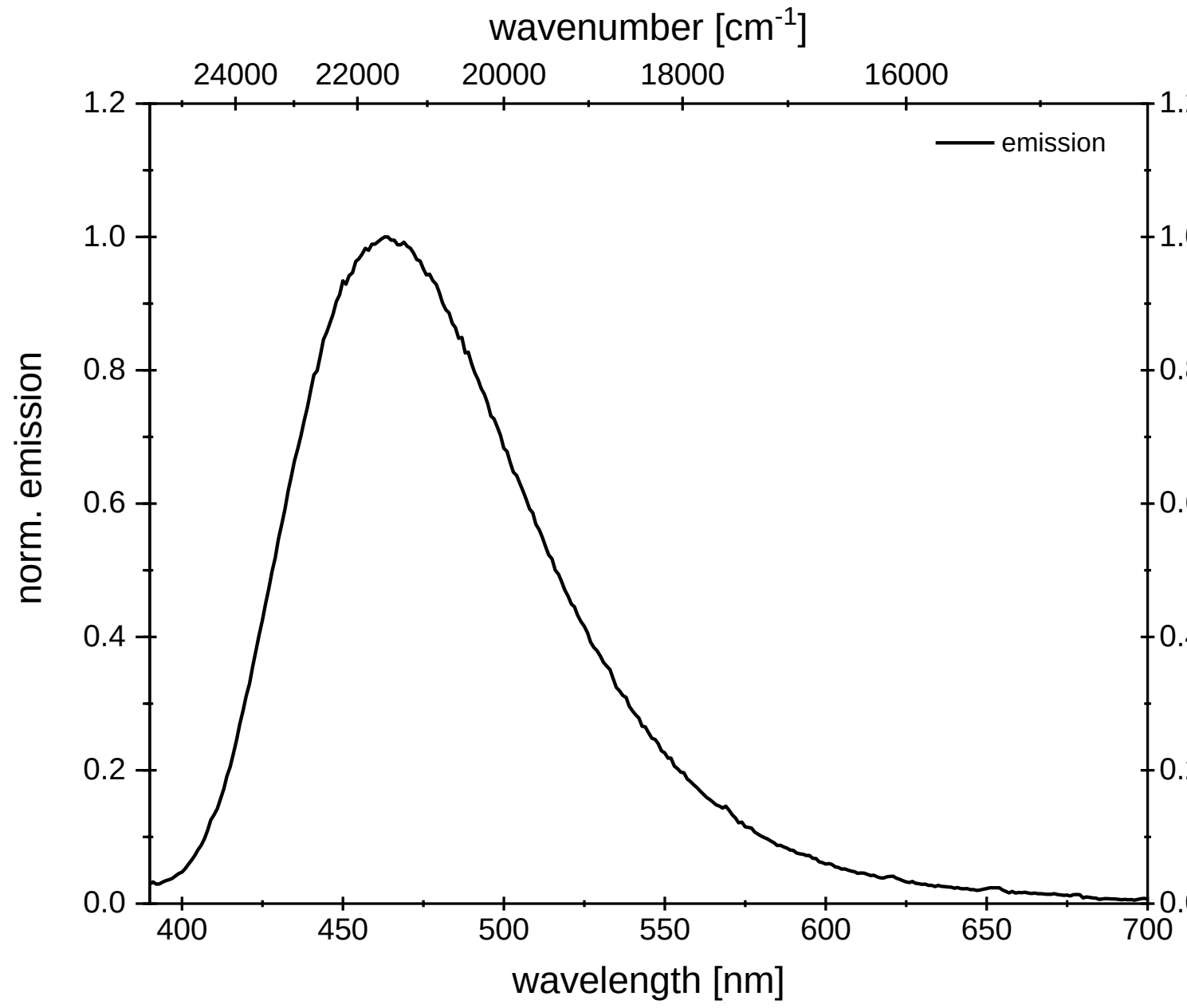

Figure S118. PMMA matrix emission spectra of compound **7c** (recorded at T = 293 K).

7 Data of quantum chemical calculations

7.1 Overview of quantum chemical calculations of dimers 4, 5 and 6

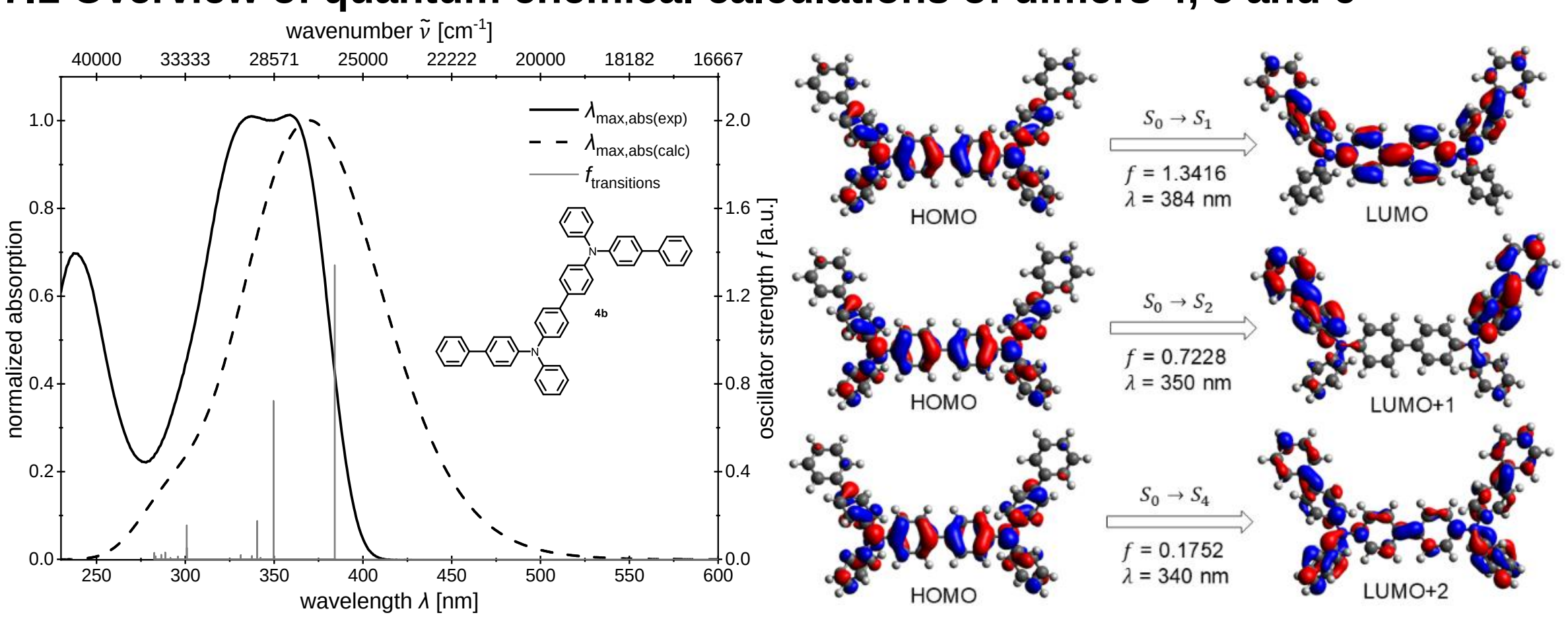

Figure S119. Comparison of the calculated (Gaussian 16, PBE1PBE/6 31+G**, PCM CH_2Cl_2) and experimentally determined (recorded in CH_2Cl_2, T = 293 K, c = 10^{-5} M) UV/Vis spectrum of *sym-o*-(bTAA)$_2$ **4b** with the calculated transitions as bars (left). Calculated molecular orbitals (Gaussian 16, PBE1PBE/6 31+G**, PCM CH_2Cl_2, isosurface value at 0.025 a.u.) of **4b** for the HOMO→LUMO transition and the three dominant energy transitions (right).

Table S9. TD DFT calculations for the UV/Vis absorption and emission maxima of *sym-p*-(bTAA)$_2$ **4** (Gaussian 16, PBE1PBE/6-31+G**, PCM CH_2Cl_2).

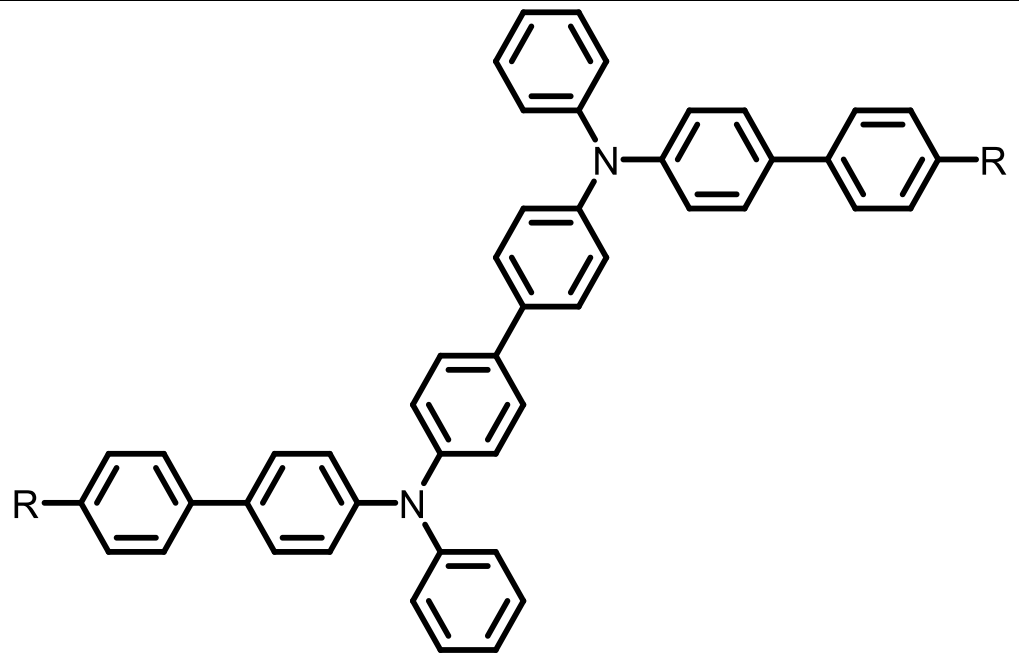

**Com-pound**	**R[1]**	$\lambda_{max,abs(exp)}$ **[nm][a] (**ε**[M^{-1} cm^{-1}])**	$\lambda_{max,\text{ abs}(calcd)}$ **[nm] (oscillator strength), most dominant contribution**	$\lambda_{max,em(exp}$ **[nm][b]**	$\lambda_{max,em(calcd)}$ **[nm] (oscillator strength), most dominant contribution**
**4a**	OMe	247 (42600)	298 (0.1174) HOMO-1 → LUMO+2 (41%)	417	450 (1.5601) HOMO → LUMO (94%)
		333 (75000)	339 (1.449) HOMO → LUMO+4 (75%)		
			346 (0.7440) HOMO → LUMO+1 (61%)		
		367 (sh, 68800)	383 (1.3952) HOMO → LUMO (94%)		
**4b**	H	239 (56100)	301 (0.1558) HOMO-1 → LUMO+2 (51%)	414	447 (1.5707) HOMO → LUMO (92%)
		333 (81000)	340 (0.1752) HOMO → LUMO+2 (55%)		
			350 (0.7228) HOMO → LUMO+1 (70%)		
		363 (sh, 60800)	384 (1.3416) HOMO → LUMO (93%)		
**4c**	CN	245 (37700)	319 (0.1094) HOMO-1 → LUMO+2 (81%)	531	521 (0.5967) HOMO → LUMO (84%)
		331 (sh, 38300)	350 (0.1772) HOMO-1 → LUMO+1 (75%)		
			355 (0.4702) HOMO → LUMO+2 (81%)		
			357 (0.1461) HOMO-1 → LUMO (82%)		
		376 (60800)	403 (0.7113) HOMO → LUMO+1 (85%)		
			420 (0.9938) HOMO → LUMO (85%)		

[a] Recorded in CH_2Cl_2, T = 293 K, c = 10^{-5} M. [b] Recorded in CH_2Cl_2, T = 293 K, c = 10^{-7} M.

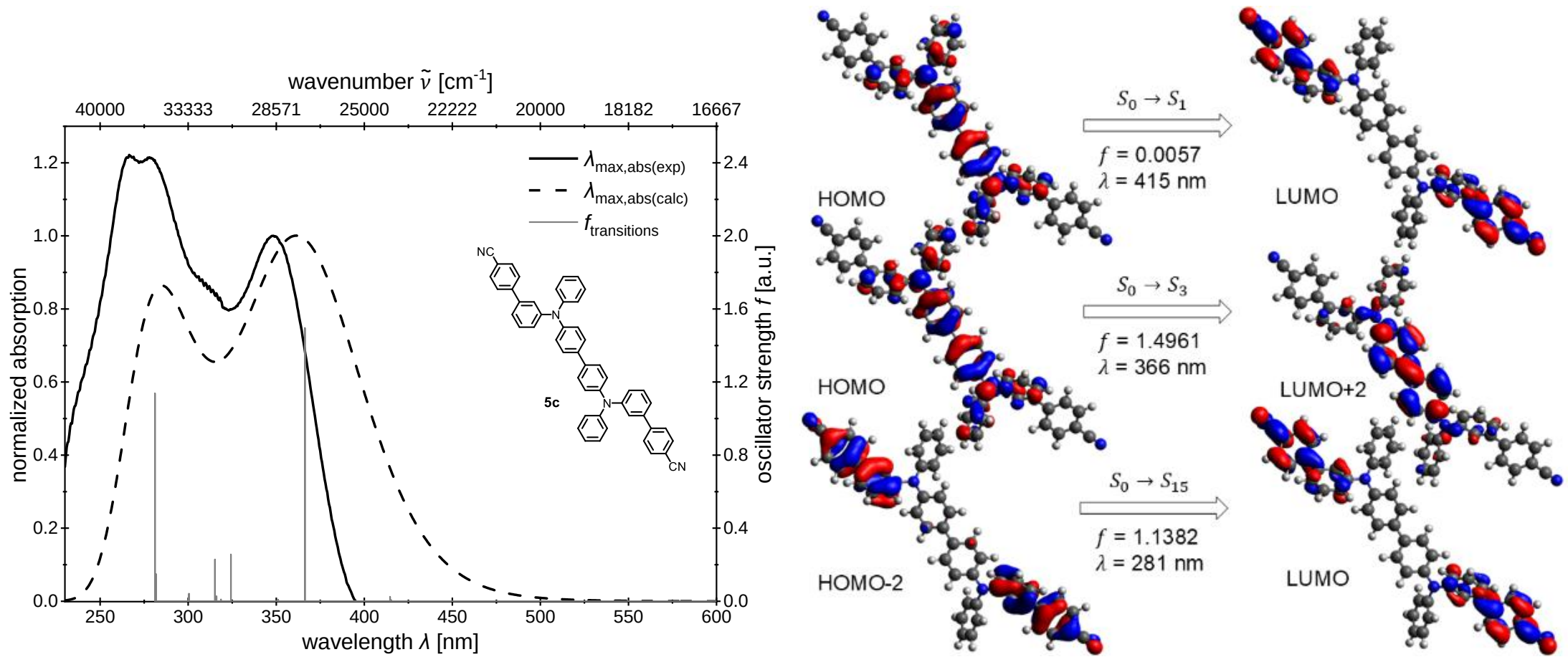

Figure S120. Comparison of the calculated (Gaussian 16, PBE1PBE/6 31+G**, PCM CH_2Cl_2) and experimentally determined (recorded in CH_2Cl_2, T = 293 K, c = 10^{-5} m) UV/Vis spectrum of *sym-m*-(bTAA)$_2$ **5c** with the calculated transitions as bars (left). Calculated molecular orbitals (Gaussian 16, PBE1PBE/6 31+G**, PCM CH_2Cl_2, isosurface value at 0.025 a.u.) of **5c** for the HOMO→LUMO transition and the three dominant energy transitions (right).

Table S10. TD DFT calculations for the UV/Vis absorption and emission maxima of *sym*-*m*-(bTAA)$_2$ **5b**, **5b'**, **5b''** and **5c** (Gaussian 16, PBE1PBE/6-31+G**, PCM CH_2Cl_2).

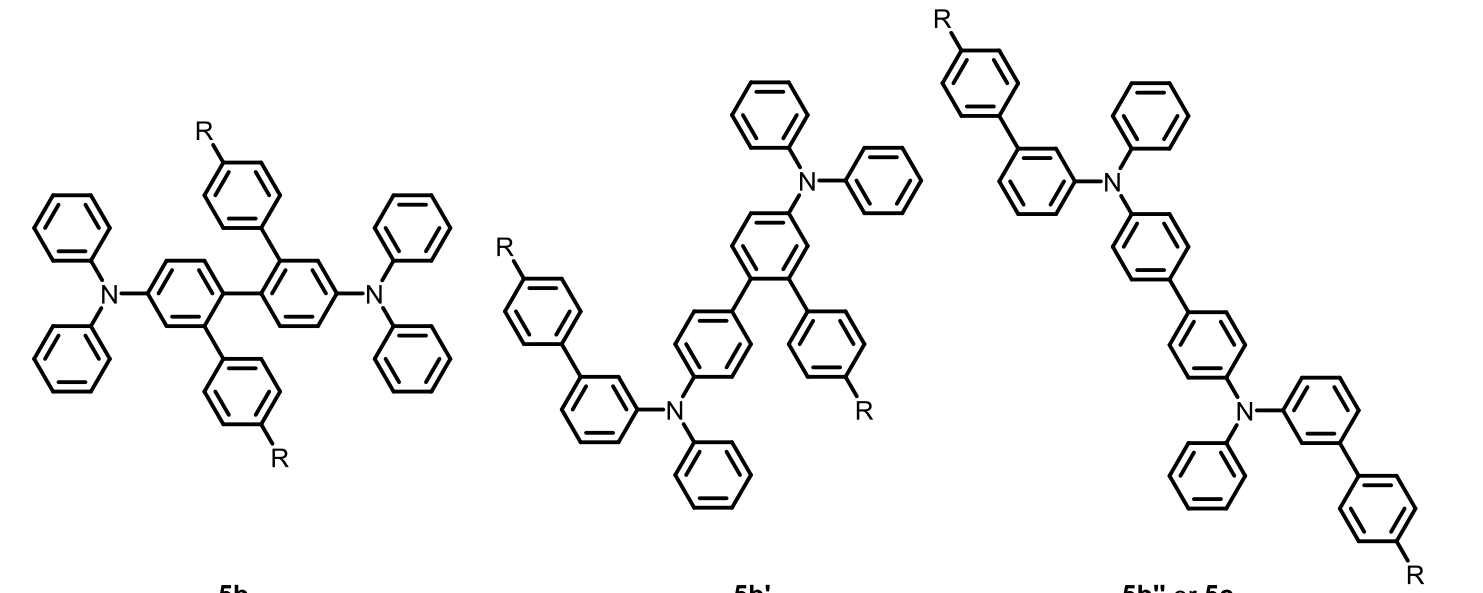

5b **5b'** **5b''** or **5c**

Compound	R	$\lambda_{max,abs(exp)}$ [nm][a] (ε[M^{-1} cm^{-1}])	$\lambda_{max,\ abs(calcd)}$ [nm] (oscillator strength), most dominant contribution	$\lambda_{max,em(exp)}$ [nm][b]	$\lambda_{max,em(calcd)}$ [nm] (oscillator strength), most dominant contribution
**5b**	H	244 (44200)[c]	298 (0.0663) HOMO → LUMO+5 (45%)	425[d]	430 (0.8420) HOMO → LUMO (95%)
		316 (36000)[c]	314 (0.3871) HOMO → LUMO+3 (69%)		
		335 (36000)[c]	342 (0.3612) HOMO → LUMO+1 (85%)		
			366 (0.5579) HOMO → LUMO (95%)		
**5b'**	H	244 (44200)[c]	283 (0.0398) HOMO → LUMO+9 (57%)	425[d]	432 (0.9765) HOMO → LUMO (91%)
		316 (36000)[c]	314 (0.2485) HOMO → LUMO+4 (70%)		
			320 (0.1906) HOMO → LUMO+3 (67%)		
		335 (36000)[c]	341 (0.1602) HOMO → LUMO+2 (77%)		
			352 (0.1602) HOMO → LUMO+1 (72%)		
			363 (0.7715) HOMO → LUMO (88%)		
**5b''**	H	244 (44200)[c]	303 (0.0875) HOMO → LUMO+6 (66%)	425[d]	440 (1.0833) HOMO → LUMO (88%)
		316 (36000)[c]	321 (0.4039) HOMO → LUMO+4 (75%)		
		335 (36000)[c]	353 (0.1462) HOMO → LUMO+2 (75%)		
			371 (1.2086) HOMO → LUMO (90%)		
**5c**	CN	266 (63000)	281 (1.1382) HOMO-3 → LUMO+1 (34%) HOMO-2 → LUMO (34%)	532	557 (0.0103) HOMO → LUMO (85%)
		280 (sh, 63000)	282 (0.1475) HOMO-1 → LUMO+3 (55%)		
		302 (sh, 16700)	315 (0.2286) HOMO → LUMO+8 (67%)		
			324 (0.2564) HOMO → LUMO+4 (49%)		
		348 (51500)	366 (1.4961) HOMO → LUMO+2 (95%)		

415 (0.0245)
HOMO → LUMO+1 (86%)
415 (0.0057)
HOMO → LUMO (86%)

[a] Recorded in CH_2Cl_2, T = 293 K, c = 10^{-5} M. [b] Recorded in CH_2Cl_2, T = 293 K, c = 10^{-7} M. [c] Experimental absorption of mixture **5b**/**5b'**/**5b''**. [d] Experimental emission of mixture **5b**/**5b'**/**5b''**.

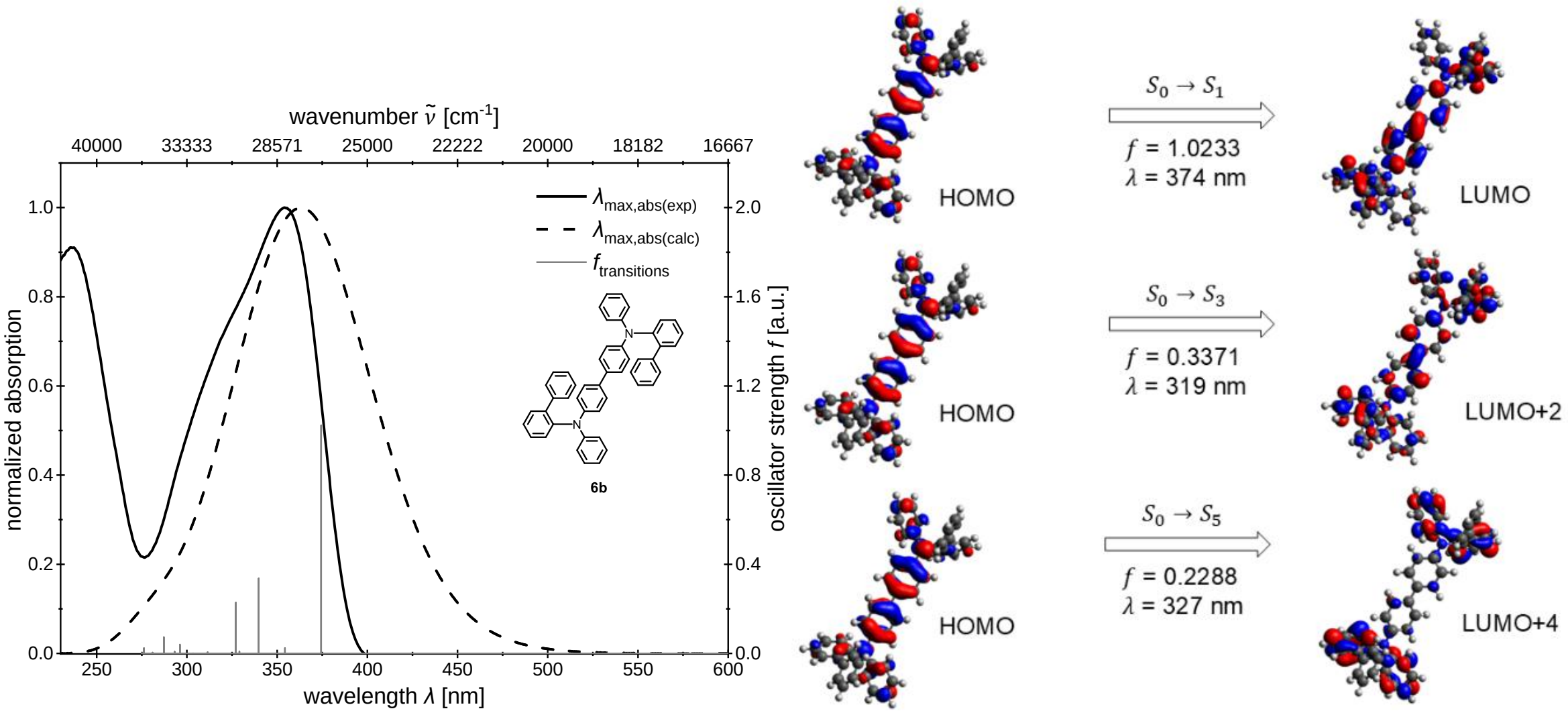

Figure S121. Comparison of the calculated (Gaussian 16, PBE1PBE/6 31+G**, PCM CH_2Cl_2) and experimentally determined (recorded in CH_2Cl_2, T = 293 K, c = 10^{-5} m) UV/Vis spectrum of *sym*-*o*-(bTAA)$_2$ **6b** with the calculated transitions as bars (left). Calculated molecular orbitals (Gaussian 16, PBE1PBE/6 31+G**, PCM CH_2Cl_2, isosurface value at 0.025 a.u.) of **6b** for the HOMO→LUMO transition and the three dominant energy transitions (right).

Table S11. TD DFT calculations for the UV/Vis absorption and emission maxima of *sym-o*-(bTAA)$_2$ **6** (Gaussian 16, PBE1PBE/6-31+G**, PCM CH_2Cl_2).

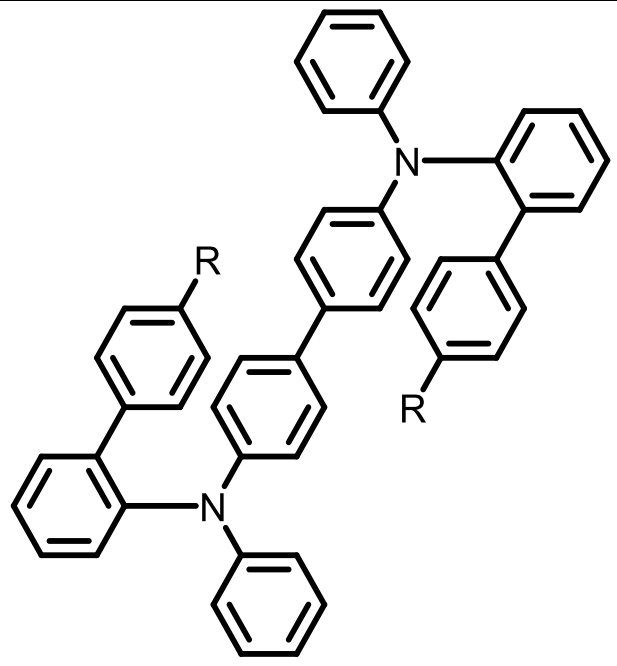

**Com-pound**	**R**	$\lambda_{max,abs(exp)}$ **[nm]**[a] **(ε[M^{-1} cm^{-1}])**	$\lambda_{max,\,\mathrm{abs}(calcd)}$ **[nm] (oscillator strength), most dominant contribution**	$\lambda_{max,em(exp)}$ **[nm]**[b]	$\lambda_{max,em(calcd)}$ **[nm] (oscillator strength), most dominant contribution**
**6a**	OMe	253 (42000)	326 (0.2480) HOMO-1 → LUMO+4 (81%)	403	446 (1.5373) HOMO → LUMO (94%)
		318 (38600)	337 (0.1460) HOMO → LUMO+2 (83%)		
		355 (55400)	370 (1.1404) HOMO → LUMO (94%)		
**6b**	H	238 (37900)	287 (0.0737) HOMO → LUMO+8 (61%)	405	411 (1.5067) HOMO → LUMO (92%)
		319 (29700)	327 (0.2288) HOMO → LUMO+4 (83%)		
			340 (0.3371) HOMO → LUMO+2 (84%)		
		355 (41800)	374 (1.0233) HOMO → LUMO (92%)		
**6c**	CN	255 (66300)	299 (0.1515) HOMO → LUMO+8 (78%)	523	527 (0.1554) HOMO → LUMO (93%)
		303 (37400)	328 (0.1275) HOMO → LUMO+4 (82%)		
			342 (0.0805) HOMO-1 → LUMO+1 (74%)		
			352 (1.0132) HOMO → LUMO+2 (84%)		
		346 (58300)	421 (0.0272) HOMO → LUMO+1 (85%)		
			429 (0.2841) HOMO → LUMO (87%)		

[a] Recorded in CH_2Cl_2, T = 293 K, c = 10^{-5} M. [b] Recorded in CH_2Cl_2, T = 293 K, c = 10^{-7} M.

Table S12. TD DFT calculations for the UV/Vis absorption and emission maxima of *sym-o*-(bTAA)$_2$ **6d** (Gaussian16, *functional*/6-31+G**, PCM CH_2Cl_2).

compound	functional	$\lambda_{max,abs(exp)}$ [nm][a] (ε[M^{-1} cm^{-1}])	$\lambda_{max,\ \mathrm{abs}(calcd)}$ [nm] (oscillator strength), most dominant contribution
**6d**	PBE1PBE	304 (sh, 26500)	346 (0.2555) HOMO-1 → LUMO+4 (79%)
		343 (35000)	362 (0.7401) HOMO → LUMO+2 (89%)
		406 (sh, 4300)	514 (0.0128) HOMO → LUMO (78%)
**6d**	ωB97XD	304 (sh, 26500)	286 (0.2688) HOMO → LUMO+4 (24%)
			288 (0.1875) HOMO → LUMO+3 (31%)
		343 (35000)	308 (0.9143) HOMO → LUMO+2 (71%)
		406 (sh, 4300)	361 (0.0030) HOMO → LUMO+1 (35%)
			363 (0.0036) HOMO → LUMO (39%)
**6d**	CAM-B3LYP	304 (sh, 26500)	289 (0.3327) HOMO → LUMO+4 (25%)
		343 (35000)	309 (0.9825) HOMO → LUMO+2 (65%)
		406 (sh, 4300)	367 (0.0214) HOMO → LUMO (68%)

[a] Recorded in CH_2Cl_2, T = 293 K, c = 10^{-5} M. [b] Recorded in CH_2Cl_2, T = 293 K, c = 10^{-7} M.

Table S13. TD DFT calculations for the UV/Vis absorption and emission maxima of *sym-o*-(bTAA)$_2$ **7** (Gaussian 16, *functional*/6-31+G**, PCM CH_2Cl_2).

**compound**	**functional**	$\lambda_{max,abs(exp)}$ **[nm]**[a] **(ε[M^{-1} cm^{-1}])**	$\lambda_{max,\,abs(calcd)}$ **[nm] (oscillator strength), most dominant contribution**	$\lambda_{max,em(exp)}$ **[nm]**[b]	$\lambda_{max,em(calcd)}$ **[nm] (oscillator strength), most dominant contribution**
**7a**	PBE1PBE	273 (sh, 38000)	351 (0.9070) HOMO-1 → LUMO+4 (88%)	543	1023 (0.0186) HOMO → LUMO (96%)
		294 (43500)	398 (0.2340) HOMO → LUMO+2 (80%)		
		344 (57800)	433 (0.0119) HOMO → LUMO (75%)		
			433 (0.0109) HOMO → LUMO+1 (80%)		
**7a**	CAM-B3LYP	273 (sh, 38000)	277 (0.1426) HOMO → LUMO+4 (34%)	543	nd[c]
		294 (43500)	307 (0.4644) HOMO → LUMO+4 (54%)		
		344 (57800)	327 (0.4344) HOMO → LUMO (51%)		
			332 (0.4123) HOMO → LUMO+2 (44%)		
**7b**	PBE1PBE	277 (sh, 48700)	318 (0.1485) HOMO → LUMO+8 (51%)	558	nd[c]
		294 (55800)	358 (0.4710) HOMO-1 → LUMO (52%)		
			361 (0.4678) HOMO → LUMO+4 (46%)		
		348 (35200)	422 (0.1528) HOMO-1 → LUMO+3 (62%)		
			427 (0.0339) HOMO → LUMO+2 (58%)		
**7c**	PBE1PBE	272 (sh, 43700)	298 (0.1536) HOMO-1 → LUMO+8 (30%)	516	nd[c]
		301 (58800)	306 (0.7833) HOMO → LUMO+7 (46%)		
			323 (0.2279) HOMO → LUMO+5 (75%)		
		363 (sh, 13400)	396 (0.0794) HOMO-1 → LUMO+2 (95%)		
			429 (0.0092) HOMO-1 → LUMO (96%)		
			431 (0.0068) HOMO → LUMO+1 (96%)		

[a] Recorded in CH_2Cl_2, T = 293 K, c = 10^{-5} M. [b] Recorded in CH_2Cl_2, T = 293 K, c = 10^{-7} M. [c] Not determined.

Table S14. TD DFT calculations for the UV/Vis absorption and emission maxima of *sym*-*o*-(bTAA)$_2$ **4a**–**c, 5b, 5c, 6a**–**c** (Gaussian 16, PBE1PBE/6-31+G**, PCM CH_2Cl_2).

excitation (S_0 to S_1*)			emission (S_1 to S_0*)		
**compound**	**Δr [Å]**	**S_r**	**compound**	**Δr [Å]**	**S_r**
**4a**	0.35	0.67	**4a**	0.15	0.70
**4b**	1.16	0.64	**4b**	0.31	0.70
**4c**	3.72	0.53	**4c**	8.17	0.36
**5b**	0.71	0.58	**5b**	0.70	0.70
**5c**	0.91	0.31	**5c**	8.09	0.22
**6a**	0.21	0.68	**6a**	0.03	0.73
**6b**	0.47	0.64	**6b**	0.03	0.73
**6c**	2.60	0.35	**6c**	5.58	0.29

7.2 Computed xyz-coordinates of cation 1a$^+$ (uPBE1PBE/6-31+G**)

Inputfile:

%NProcShared=16

%Chk=gauss.chk

%mem=16GB

#p uPBE1PBE 6-31+G** pop=full GFInput opt freq output=WFN

1a$^+$

1 2

C 0 -4.502222 5.001292 -1.351313

C 0 -4.004452 3.795521 -1.028893

C 0 -4.771261 2.723634 -0.731536

C 0 -6.095945 2.981029 -0.789786

C 0 -6.597625 4.183681 -1.112172

C 0 -5.824128 5.242216 -1.406819

C 0 -4.260526 1.504860 -0.404935

O 0 -6.414885 6.440987 -1.725376

C 0 -5.017123 0.441030 -0.066622

C 0 -4.510485 -0.762059 0.252242

C 0 -3.191096 -1.049983 0.280197

C 0 -2.440783 0.021534 -0.056525

C 0 -2.941484 1.225079 -0.383070

N 0 -2.704954 -2.211460 0.591875

C 0 -1.512415 -2.301040 1.095673

C 0 -3.411814 -3.282616 0.399703

C 0 -0.728602 -3.398433 0.961764

C 0 0.507720 -3.505150 1.474744

C 0 1.038464 -2.493717 2.171650

C 0 0.304770 -1.386801 2.335208

C 0 -0.927172 -1.307072 1.806995

C 0 -3.263571 -4.413283 1.131946

C	0	-3.988933	-5.528049	0.946983
C	0	-4.923602	-5.573685	-0.009407
C	0	-5.108801	-4.483718	-0.763059
C	0	-4.371354	-3.381560	-0.552119
C	0	-5.589452	7.545834	-2.013048
H	0	-3.780047	5.804127	-1.572450
H	0	-2.905072	3.758541	-1.021665
H	0	-6.863686	2.221351	-0.582620
H	0	-7.693830	4.316165	-1.139988
H	0	-6.114033	0.496976	-0.001382
H	0	-5.261370	-1.503185	0.578941
H	0	-1.344560	-0.062328	-0.161902
H	0	-2.178542	1.967109	-0.662221
H	0	-1.034444	-4.260919	0.344702
H	0	1.103054	-4.419086	1.307033
H	0	2.051600	-2.570243	2.598995
H	0	0.712474	-0.548653	2.926073
H	0	-1.467636	-0.380677	2.067280
H	0	-2.576196	-4.463007	1.994049
H	0	-3.834386	-6.406934	1.596232
H	0	-5.524079	-6.483608	-0.171850
H	0	-5.857788	-4.505770	-1.573239
H	0	-4.557053	-2.571773	-1.278639
H	0	-6.249114	8.413539	-2.239026
H	0	-4.966687	7.804267	-1.127678
H	0	-4.966671	7.340043	-2.912070

$1a^{+}$.wfn

SCF Done: E(UPBE1PBE) = -1093.77312502 A.U. after 24 cycles

Zero-point correction= 0.395565 (Hartree/Particle)

Thermal correction to Energy= 0.417509

Thermal correction to Enthalpy= 0.418453

Thermal correction to Gibbs Free Energy= 0.342054

Sum of electronic and zero-point Energies= -1093.432183

Sum of electronic and thermal Energies= -1093.410239

Sum of electronic and thermal Enthalpies= -1093.409295

Sum of electronic and thermal Free Energies= -1093.485694

7.3 Computed xyz-coordinates of cation 1b+ (uPBE1PBE/6-31+G**)

Inputfile:

%NProcShared=16

%Chk=gauss.chk

%mem=16GB

#p uPBE1PBE 6-31+G** pop=full GFInput opt freq output=WFN

1b+

1 2

C 0 -4.566304 5.707268 -1.527673

C 0 -4.062855 4.504062 -1.206660

C 0 -4.829923 3.427963 -0.915411

C 0 -6.160944 3.664742 -0.974757

C 0 -6.687195 4.858659 -1.293869

C 0 -5.890168 5.896194 -1.574526

C 0 -4.307888 2.212668 -0.590876

C 0 -5.053247 1.140987 -0.251627

C 0 -4.535448 -0.058024 0.064802

C 0 -3.213645 -0.334708 0.089378

C 0 -2.474028 0.743946 -0.247982

C 0 -2.986443 1.943247 -0.572001

N 0 -2.716449 -1.492174 0.398478

C 0 -1.521570 -1.571969 0.898280

C 0 -3.414314 -2.569437 0.207645

C 0 -0.728641 -2.662259 0.759959

C 0 0.510343 -2.758866 1.268516

C 0 1.034513 -1.743893 1.965232

C 0 0.291619 -0.643773 2.133130

C 0 -0.942761 -0.573984 1.609205

C 0 -3.253634 -3.699119 0.938798

C 0 -3.969546 -4.820237 0.755430

C 0   -4.906749   -4.873674   -0.198055

C 0   -5.104070   -3.784970   -0.950475

C 0   -4.375867   -2.676361   -0.741233

H 0   -3.891916   6.551233   -1.753839

H 0   -2.963606   4.478700   -1.196196

H 0   -6.917909   2.893268   -0.772429

H 0   -7.782082   4.993608   -1.329111

H 0   -6.312702   6.879849   -1.837204

H 0   -6.150354   1.185517   -0.182418

H 0   -5.278997   -0.806061   0.392650

H 0   -1.377358   0.669910   -0.356297

H 0   -2.229551   2.690726   -0.852710

H 0   -1.029020   -3.526471   0.142607

H 0   1.113182   -3.667208   1.097292

H 0   2.049746   -1.812101   2.388997

H 0   0.693963   0.196902   2.724056

H 0   -1.490659   0.347119   1.872810

H 0   -2.563108   -3.743189   1.798706

H 0   -3.805128   -5.698070   1.403642

H 0   -5.499566   -5.788830   -0.359226

H 0   -5.855328   -3.813360   -1.758313

H 0   -4.570943   -1.867830   -1.466723

$1b^{+}$.wfn

SCF Done: E(UPBE1PBE) = -979.415073931   A.U. after   25 cycles

Zero-point correction=   0.362543 (Hartree/Particle)

Thermal correction to Energy=   0.381858

Thermal correction to Enthalpy=   0.382802

Thermal correction to Gibbs Free Energy=   0.312484

Sum of electronic and zero-point Energies=   -979.054154

Sum of electronic and thermal Energies=   -979.034839

Sum of electronic and thermal Enthalpies=   -979.033895

Sum of electronic and thermal Free Energies= -979.104213

7.4 Computed xyz-coordinates of cation 1c^{+} (uPBE1PBE/6-31+G**)

Inputfile:

%NProcShared=16

%Chk=gauss.chk

%mem=16GB

#p uPBE1PBE 6-31+G** pop=full GFInput opt freq output=WFN

1c^{+}

1 2

C	0	-4.566952	5.522370	-1.482103
C	0	-4.063526	4.319027	-1.160851
C	0	-4.829312	3.243436	-0.866169
C	0	-6.159709	3.482230	-0.922512
C	0	-6.686139	4.676267	-1.241645
C	0	-5.891537	5.716210	-1.526653
C	0	-4.307287	2.028191	-0.541652
C	0	-5.052658	0.956757	-0.201687
C	0	-4.534807	-0.242268	0.114667
C	0	-3.213025	-0.519106	0.138627
C	0	-2.473458	0.559365	-0.199374
C	0	-2.985857	1.758645	-0.523537
N	0	-2.715834	-1.676580	0.447744
C	0	-1.520993	-1.756310	0.947654
C	0	-3.413723	-2.753822	0.256790
C	0	-0.727892	-2.846581	0.810144
C	0	0.510936	-2.942876	1.319158
C	0	1.034809	-1.927500	2.015494
C	0	0.291791	-0.827353	2.182576
C	0	-0.942432	-0.757919	1.658230
C	0	-3.253053	-3.883957	0.987255

C 0 -3.968929 -5.005011 0.803343

C 0 -4.906266 -5.057867 -0.150039

C 0 -5.103724 -3.968716 -0.901768

C 0 -4.375472 -2.860244 -0.691972

C 0 -6.395792 6.890368 -1.840177

N 0 -6.839384 7.924168 -2.115496

H 0 -3.881578 6.358030 -1.709237

H 0 -2.964211 4.292160 -1.153395

H 0 -6.916242 2.711126 -0.716774

H 0 -7.783281 4.799201 -1.270467

H 0 -6.149751 1.001397 -0.132070

H 0 -5.278250 -0.990194 0.443068

H 0 -1.376833 0.485068 -0.308201

H 0 -2.228972 2.505991 -0.804677

H 0 -1.027923 -3.711172 0.193193

H 0 1.113815 -3.851354 1.148671

H 0 2.049886 -1.995493 2.439673

H 0 0.693787 0.013751 2.773147

H 0 -1.490372 0.163315 1.921207

H 0 -2.562462 -3.928629 1.847048

H 0 -3.804300 -5.883271 1.450989

H 0 -5.499013 -5.972990 -0.311699

H 0 -5.855131 -3.996491 -1.709534

H 0 -4.570756 -2.051367 -1.416990

$1c^{+}$.wfn

SCF Done: E(UPBE1PBE) = -1071.54824149 A.U. after 26 cycles

Zero-point correction= 0.360902 (Hartree/Particle)

Thermal correction to Energy= 0.382071

Thermal correction to Enthalpy= 0.383015

Thermal correction to Gibbs Free Energy= 0.308127

Sum of electronic and zero-point Energies= -1071.187619

Sum of electronic and thermal Energies= -1071.166449

Sum of electronic and thermal Enthalpies= -1071.165505

Sum of electronic and thermal Free Energies= -1071.240394

7.5 Computed xyz-coordinates of cation 2a⁺ (uPBE1PBE/6-31+G**)

Inputfile:

%NProcShared=16

%Chk=gauss.chk

%mem=16GB

#p uPBE1PBE 6-31+G** pop=full GFInput opt freq output=WFN

2a⁺

1 2

C 0 -0.351187 0.935162 -0.982329

C 0 0.129918 -0.307062 -0.852509

C 0 -0.652381 -1.295362 -0.403516

C 0 -1.943336 -1.095198 -0.061608

C 0 -2.350962 0.189978 -0.211709

C 0 -1.626778 1.239358 -0.667648

N 0 -2.712068 -2.047220 0.373178

C 0 -3.994605 -1.983185 0.182792

C 0 -2.197446 -3.064761 0.992844

C 0 -4.899829 -2.581383 0.994668

C 0 -6.228619 -2.519482 0.811402

C 0 -6.736238 -1.840311 -0.223707

C 0 -5.885095 -1.231459 -1.057419

C 0 -4.561180 -1.308028 -0.847230

C 0 -2.766056 -4.294770 1.006891

C 0 -2.243309 -5.356128 1.641949

C 0 -1.094995 -5.239068 2.318695

C 0 -0.492949 -4.044282 2.338017

C 0 -1.036965 -3.001623 1.689988

C 0 -2.128890 2.500870 -0.789364

C 0 -1.367187 3.573910 -1.095799

C 0 -1.858628 4.817132 -1.217929

C 0	-3.159418	5.107783	-1.048600
C 0	-3.933471	4.048899	-0.752623
C 0	-3.435927	2.806741	-0.629273
O 0	-3.585616	6.405595	-1.195349
C 0	-4.959291	6.685530	-1.055765
H 0	0.370665	1.659498	-1.385761
H 0	1.170554	-0.526362	-1.150041
H 0	-0.186234	-2.295353	-0.408914
H 0	-3.353974	0.409170	0.175027
H 0	-4.597986	-3.102441	1.919575
H 0	-6.912347	-3.009270	1.525861
H 0	-7.825171	-1.782831	-0.384452
H 0	-6.279311	-0.682776	-1.929954
H 0	-3.953790	-0.829084	-1.634780
H 0	-3.672885	-4.524127	0.421134
H 0	-2.745623	-6.337494	1.592363
H 0	-0.657066	-6.102740	2.845213
H 0	0.443230	-3.918151	2.908511
H 0	-0.495306	-2.050829	1.834508
H 0	-0.280015	3.513075	-1.250368
H 0	-1.168789	5.643789	-1.464510
H 0	-5.018746	4.176270	-0.607357
H 0	-4.203610	2.049705	-0.409067
H 0	-5.102027	7.777390	-1.218812
H 0	-5.545852	6.139270	-1.827831
H 0	-5.301414	6.441024	-0.025431

$2a^{+}$.wfn

SCF Done: E(RPBE1PBE) = -1093.67293001 A.U. after   24 cycles

Zero-point correction = 0.394948 (Hartree/Particle)

Thermal correction to Energy = 0.416903

Thermal correction to Enthalpy = 0.417848

Thermal correction to Gibbs Free Energy = 0.341506

Sum of electronic and zero-point Energies = -1093.424562

Sum of electronic and thermal Energies = -1093.402607

Sum of electronic and thermal Enthalpies = -1093.401663

Sum of electronic and thermal Free Energies = -1093.478005

7.6 Computed xyz-coordinates of cation $2b^+$ (uPBE1PBE/6-31+G**)

Inputfile:

%NProcShared=16

%Chk=gauss.chk

%mem=16GB

#p uPBE1PBE 6-31+G** pop=full GFInput opt freq output=WFN

$2b^+$

1 2

```
C 0   -0.325574    1.557769   -1.033435
C 0    0.136373    0.304204   -0.952218
C 0   -0.655586   -0.685555   -0.524073
C 0   -1.937510   -0.475369   -0.155481
C 0   -2.324706    0.820984   -0.255888
C 0   -1.590663    1.873522   -0.688621
N 0   -2.715990   -1.428609    0.258992
C 0   -4.000021   -1.335978    0.091662
C 0   -2.210275   -2.475937    0.834807
C 0   -4.902909   -1.946202    0.897155
C 0   -6.233109   -1.855023    0.737889
C 0   -6.744793   -1.131642   -0.264750
C 0   -5.896112   -0.509386   -1.091036
C 0   -4.570629   -0.616167   -0.905352
C 0   -2.800818   -3.695524    0.815347
C 0   -2.287766   -4.787578    1.404624
C 0   -1.127098   -4.714603    2.066318
C 0   -0.503220   -3.532009    2.117382
C 0   -1.038185   -2.457799    1.514914
C 0   -2.073590    3.146914   -0.761142
C 0   -1.288792    4.214694   -1.037035
C 0   -1.748229    5.474485   -1.114540
```

C	0	-3.046076	5.734574	-0.919463
C	0	-3.861547	4.708710	-0.649798
C	0	-3.378386	3.457558	-0.576217
H	0	0.402871	2.281468	-1.425890
H	0	1.168256	0.077676	-1.273800
H	0	-0.206669	-1.692324	-0.570434
H	0	-3.317167	1.042980	0.155623
H	0	-4.596031	-2.504079	1.798654
H	0	-6.914126	-2.357284	1.446232
H	0	-7.834942	-1.049595	-0.405342
H	0	-6.294165	0.075833	-1.937699
H	0	-3.967307	-0.120988	-1.685978
H	0	-3.720576	-3.888389	0.236582
H	0	-2.808538	-5.757584	1.328897
H	0	-0.696843	-5.603876	2.555211
H	0	0.443882	-3.442572	2.676665
H	0	-0.477076	-1.522364	1.683591
H	0	-0.204083	4.136994	-1.200359
H	0	-1.058134	6.306987	-1.335822
H	0	-3.433242	6.764681	-0.982818
H	0	-4.937731	4.900619	-0.496995
H	0	-4.156266	2.705782	-0.376359

$2b^{+}$.wfn

SCF Done: E(RPBE1PBE) = -979.408230836 A.U. after 24 cycles

Zero-point correction= 0.362223 (Hartree/Particle)

Thermal correction to Energy= 0.381562

Thermal correction to Enthalpy= 0.382506

Thermal correction to Gibbs Free Energy= 0.312175

Sum of electronic and zero-point Energies= -979.050023

Sum of electronic and thermal Energies= -979.030684

Sum of electronic and thermal Enthalpies= -979.029739

Sum of electronic and thermal Free Energies= -979.100070

7.7 Computed xyz-coordinates of cation 2c⁺ (uPBE1PBE/6-31+G**)

Inputfile:

%NProcShared=16

%Chk=gauss.chk

%mem=16GB

#p uPBE1PBE 6-31+G** pop=full GFInput opt freq output=WFN

$2c^+$

1 2

C 0 -0.330913 1.367914 -1.019353

C 0 0.134720 0.117121 -0.918328

C 0 -0.655976 -0.869052 -0.479589

C 0 -1.940362 -0.657946 -0.120242

C 0 -2.331715 0.635345 -0.241408

C 0 -1.598677 1.684027 -0.684967

N 0 -2.717624 -1.607468 0.304717

C 0 -4.001114 -1.522696 0.129130

C 0 -2.210737 -2.643613 0.899440

C 0 -4.906160 -2.124373 0.938628

C 0 -6.235790 -2.041210 0.770394

C 0 -6.744636 -1.335173 -0.245970

C 0 -5.893741 -0.721629 -1.076504

C 0 -4.568923 -0.820100 -0.881683

C 0 -2.796024 -3.865850 0.896184

C 0 -2.281482 -4.946192 1.505435

C 0 -1.124690 -4.857674 2.172008

C 0 -0.506028 -3.671784 2.207569

C 0 -1.042322 -2.609608 1.585269

C 0 -2.085337 2.954632 -0.776669

C 0 -1.303028 4.022167 -1.058523

C 0 -1.766061 5.279573 -1.154163

C 0	-3.066915	5.541525	-0.972965
C 0	-3.878884	4.512541	-0.698079
C 0	-3.391996	3.263888	-0.605988
C 0	-3.532404	6.768661	-1.065863
N 0	-3.942012	7.848794	-1.148576
H 0	0.396666	2.088740	-1.418706
H 0	1.168880	-0.110498	-1.231771
H 0	-0.203568	-1.874926	-0.508979
H 0	-3.326907	0.859955	0.162060
H 0	-4.602228	-2.667352	1.850139
H 0	-6.918862	-2.535720	1.482226
H 0	-7.834254	-1.259963	-0.394178
H 0	-6.289272	-0.150625	-1.934008
H 0	-3.963105	-0.334007	-1.666042
H 0	-3.711881	-4.071722	0.315761
H 0	-2.797812	-5.919480	1.442501
H 0	-0.693278	-5.737160	2.677290
H 0	0.437727	-3.569379	2.770291
H 0	-0.486082	-1.669234	1.742005
H 0	-0.216639	3.945345	-1.211338
H 0	-1.066274	6.103679	-1.379623
H 0	-4.959458	4.689502	-0.553807
H 0	-4.168298	2.511026	-0.403725

$2c^+$.wfn

SCF Done: E(RPBE1PBE) = -1071.54099481   A.U. after   25 cycles

Zero-point correction=   0.360688 (Hartree/Particle)

Thermal correction to Energy=   0.381881

Thermal correction to Enthalpy=   0.382825

Thermal correction to Gibbs Free Energy=   0.307900

Sum of electronic and zero-point Energies=   -1071.184521

Sum of electronic and thermal Energies=   -1071.163328

Sum of electronic and thermal Enthalpies= -1071.162384

Sum of electronic and thermal Free Energies= -1071.237309

7.8 Computed xyz-coordinates of cation 3a⁺ (uPBE1PBE/6-31+G**)

Inputfile:

%NProcShared=16

%Chk=gauss.chk

%mem=16GB

#p uPBE1PBE 6-31+G** pop=full GFInput opt freq output=WFN

3a⁺

1 2

C 0 1.171193 1.967778 -1.077878

C 0 1.676729 0.781113 -0.735449

C 0 0.841610 -0.190986 -0.346645

C 0 -0.504565 -0.047861 -0.256128

C 0 -1.000151 1.192575 -0.548833

C 0 -0.150778 2.153495 -0.979007

N 0 -1.205285 -1.104183 0.029518

C 0 -2.354739 -1.273416 -0.548955

C 0 -0.732229 -1.986701 0.855006

C 0 -3.361386 -2.017886 -0.031311

C 0 -4.554451 -2.180335 -0.625529

C 0 -4.808537 -1.594408 -1.801333

C 0 -3.845819 -0.849921 -2.357201

C 0 -2.663383 -0.701832 -1.738687

C 0 -1.073341 -3.297300 0.846912

C 0 -0.580278 -4.204193 1.705748

C 0 0.301267 -3.837149 2.643173

C 0 0.674634 -2.553124 2.691481

C 0 0.168496 -1.668547 1.817388

C 0 -2.280192 1.590676 -0.312241

C 0 -2.910138 2.545122 -1.028507

C 0 -4.175595 2.922729 -0.782656

C 0	-4.904601	2.389938	0.215623
C 0	-4.265764	1.467446	0.956628
C 0	-3.003520	1.087096	0.708711
O 0	-6.199812	2.721721	0.532039
C 0	-6.849054	3.713201	-0.229595
H 0	1.838430	2.782819	-1.405153
H 0	2.761408	0.598344	-0.819240
H 0	1.343730	-1.164585	-0.203664
H 0	-0.472509	3.186213	-1.191511
H 0	-3.293453	-2.473928	0.970834
H 0	-5.343777	-2.775328	-0.135031
H 0	-5.785722	-1.717343	-2.296079
H 0	-4.026670	-0.369338	-3.333886
H 0	-1.925753	-0.116650	-2.313681
H 0	-1.728899	-3.725658	0.070122
H 0	-0.880671	-5.263369	1.629624
H 0	0.712635	-4.573403	3.352862
H 0	1.388642	-2.224068	3.465830
H 0	0.496905	-0.627577	1.984918
H 0	-2.431619	3.022157	-1.900118
H 0	-4.608632	3.688491	-1.446220
H 0	-4.798964	1.016984	1.811986
H 0	-2.567248	0.369127	1.422932
H 0	-7.875084	3.841150	0.182742
H 0	-6.313745	4.684492	-0.137284
H 0	-6.939553	3.388608	-1.290251

$3a^{+}$.wfn

SCF Done: E(RPBE1PBE) = -1093.81310053 A.U. after 26 cycles
Zero-point correction= 0.394943 (Hartree/Particle)
Thermal correction to Energy= 0.416871
Thermal correction to Enthalpy= 0.417815

Thermal correction to Gibbs Free Energy= 0.342267
Sum of electronic and zero-point Energies= -1093.420196
Sum of electronic and thermal Energies= -1093.398268
Sum of electronic and thermal Enthalpies= -1093.397323
Sum of electronic and thermal Free Energies= -1093.472872

7.9 Computed xyz-coordinates of cation 3b$^+$ (uPBE1PBE/6-31+G**)

Inputfile:

%NProcShared=16

%Chk=gauss.chk

%mem=16GB

#p uPBE1PBE 6-31+G** pop=full GFInput opt freq output=WFN

3b$^+$

1 2

C 0 -0.400434 2.248211 -1.300545

C 0 0.066406 1.015368 -1.093242

C 0 -0.774845 0.069660 -0.655769

C 0 -2.087327 0.282611 -0.385468

C 0 -2.535500 1.564936 -0.543495

C 0 -1.685270 2.501539 -1.023289

N 0 -2.812183 -0.749026 -0.071259

C 0 -4.033245 -0.817689 -0.505539

C 0 -2.296147 -1.707080 0.635865

C 0 -5.011492 -1.537013 0.095383

C 0 -6.277062 -1.597192 -0.349054

C 0 -6.638920 -0.926075 -1.448630

C 0 -5.709147 -0.201068 -2.081142

C 0 -4.451924 -0.156215 -1.612080

C 0 -2.717161 -2.993721 0.597906

C 0 -2.178641 -3.979396 1.333842

C 0 -1.165638 -3.722030 2.169418

C 0 -0.709353 -2.466211 2.241603

C 0 -1.264137 -1.501101 1.490984

C 0 -3.748057 2.020992 -0.123967

C 0 -4.392747 3.052665 -0.712736

C 0 -5.587413 3.494749 -0.288062

C 0	-6.181261	2.927384	0.769886
C 0	-5.555187	1.924187	1.398258
C 0	-4.362654	1.493543	0.957600
H 0	0.272031	3.043573	-1.663600
H 0	1.118269	0.777083	-1.325173
H 0	-0.322173	-0.937812	-0.632884
H 0	-1.962792	3.562699	-1.133721
H 0	-4.847151	-2.058040	1.053737
H 0	-7.034152	-2.178290	0.204835
H 0	-7.675789	-0.965029	-1.820315
H 0	-5.980426	0.348774	-2.998562
H 0	-3.756920	0.422763	-2.243660
H 0	-3.490098	-3.334998	-0.111111
H 0	-2.551921	-5.013422	1.237398
H 0	-0.715801	-4.523598	2.777745
H 0	0.115046	-2.227153	2.935063
H 0	-0.852674	-0.492614	1.673151
H 0	-3.997821	3.548539	-1.614798
H 0	-6.092311	4.321257	-0.816636
H 0	-7.160771	3.287860	1.124644
H 0	-6.015161	1.466513	2.290505
H 0	-3.882664	0.709866	1.566917

$3b^{+}$.wfn

SCF Done: E(RPBE1PBE) = -979.397858762 A.U. after 25 cycles

Zero-point correction= 0.362108 (Hartree/Particle)

Thermal correction to Energy= 0.381467

Thermal correction to Enthalpy= 0.382411

Thermal correction to Gibbs Free Energy= 0.312627

Sum of electronic and zero-point Energies= -979.045385

Sum of electronic and thermal Energies= -979.026026

Sum of electronic and thermal Enthalpies= -979.025082

Sum of electronic and thermal Free Energies= -979.094866

7.10 Computed xyz-coordinates of cation 3c$^+$ (uPBE1PBE/6-31+G**)

Inputfile:

```
%NProcShared=16

%Chk=gauss.chk

%mem=16GB

#p uPBE1PBE 6-31+G** pop=full GFInput opt freq output=WFN


3c+


 1 2
C 0    0.784432    2.209838   -1.251562
C 0    1.267702    0.989367   -1.011108
C 0    0.429934    0.032108   -0.592455
C 0   -0.895574    0.221796   -0.373307
C 0   -1.362859    1.492744   -0.564279
C 0   -0.514564    2.440480   -1.025101
N 0   -1.611802   -0.819730   -0.072209
C 0   -2.814275   -0.918579   -0.550382
C 0   -1.103527   -1.758329    0.665968
C 0   -3.800102   -1.649308    0.023842
C 0   -5.047040   -1.739853   -0.465824
C 0   -5.380918   -1.090113   -1.586874
C 0   -4.442406   -0.355226   -2.194562
C 0   -3.204440   -0.279635   -1.680353
C 0   -1.497574   -3.053509    0.629752
C 0   -0.967057   -4.018899    1.397693
C 0    0.009330   -3.730873    2.266191
C 0    0.437794   -2.465317    2.338510
C 0   -0.107911   -1.521023    1.555434
C 0   -2.599063    1.928188   -0.195253
C 0   -3.244110    2.937911   -0.819673
C 0   -4.463855    3.357573   -0.446082
```

C 0 -5.088557 2.790798 0.596014

C 0 -4.460387 1.809918 1.259355

C 0 -3.242444 1.401729 0.869443

C 0 -6.283368 3.197061 0.968379

N 0 -7.335776 3.553676 1.294830

H 0 1.453963 3.014111 -1.600084

H 0 2.331938 0.769703 -1.201152

H 0 0.901201 -0.965699 -0.539826

H 0 -0.809391 3.494159 -1.159765

H 0 -3.661305 -2.154543 0.994629

H 0 -5.812959 -2.328226 0.067899

H 0 -6.402498 -1.154001 -1.995649

H 0 -4.690212 0.177569 -3.128526

H 0 -2.497879 0.305163 -2.293479

H 0 -2.237162 -3.419055 -0.102391

H 0 -1.316123 -5.061367 1.301290

H 0 0.452195 -4.515638 2.900999

H 0 1.231285 -2.201136 3.058412

H 0 0.276686 -0.502254 1.739248

H 0 -2.825982 3.431162 -1.712749

H 0 -4.956689 4.166754 -1.012762

H 0 -4.933173 1.343824 2.141234

H 0 -2.767993 0.636679 1.506282

$3c^{+}$.wfn

SCF Done: E(RPBE1PBE) = -1071.53930162 A.U. after 24 cycles

Zero-point correction= 0.360572 (Hartree/Particle)

Thermal correction to Energy= 0.381788

Thermal correction to Enthalpy= 0.382732

Thermal correction to Gibbs Free Energy= 0.308353

Sum of electronic and zero-point Energies= -1071.179426

Sum of electronic and thermal Energies= -1071.158210

Sum of electronic and thermal Enthalpies= -1071.157266

Sum of electronic and thermal Free Energies= -1071.231646

7.11 Quantum chemical calculation data of *N*[4],*N*[4]'-Bis(4'-methoxy-[1,1'-biphenyl]-4-yl)-*N*[4],*N*[4]'-diphenyl-[1,1'-biphenyl]-4,4'-diamine (4a)

7.11.1 Computed xyz-coordinates of compound 4a (PBE1PBE/6-31+G** PCM CH_2Cl_2)

Inputfile:

%NProcShared=10

%Chk=gauss.chk

%mem=10GB

#p PBE1PBE 6-31+G** pop=full GFInput opt freq SCRF=(Solvent=Dichloromethane)

Optimized S_0-geometry:

0 1

C	0	7.507043	0.711274	-1.017180
C	0	6.747358	-0.404354	-1.346319
C	0	5.733169	-0.852658	-0.490305
C	0	5.502477	-0.148298	0.698506
C	0	6.259556	0.973085	1.012975
C	0	7.280889	1.430574	0.166284
N	0	4.960951	-1.988698	-0.817409
C	0	3.570167	-1.987439	-0.581152
C	0	5.581405	-3.123264	-1.388187
C	0	2.924862	-3.129855	-0.088875
C	0	1.554456	-3.125021	0.136585
C	0	0.773980	-1.983679	-0.105298
C	0	1.433575	-0.844347	-0.592177
C	0	2.801306	-0.843174	-0.832806
C	0	4.963388	-3.820318	-2.435223
C	0	5.575364	-4.940767	-2.989750
C	0	6.816660	-5.375165	-2.524096
C	0	7.436969	-4.677421	-1.487431
C	0	6.824560	-3.565681	-0.916087
C	0	8.085367	2.622658	0.506743

C	0	9.450913	2.699231	0.181010
C	0	10.208494	3.816509	0.495955
C	0	9.621582	4.904002	1.154889
C	0	8.266464	4.851155	1.491171
C	0	7.518742	3.720174	1.164250
C	0	-0.681687	-1.980928	0.141621
C	0	-1.344064	-0.826840	0.588420
C	0	-2.712140	-0.820060	0.827029
C	0	-3.478579	-1.973643	0.613391
C	0	-2.830385	-3.131159	0.161904
C	0	-1.459642	-3.131486	-0.061548
N	0	-4.869905	-1.969231	0.846471
C	0	-5.489907	-3.086057	1.451828
C	0	-5.643867	-0.847107	0.478316
C	0	-6.728961	-3.548363	0.988148
C	0	-7.340821	-4.642490	1.593119
C	0	-6.723906	-5.302793	2.656001
C	0	-5.486566	-4.848786	3.113429
C	0	-4.875237	-3.745469	2.524877
C	0	-5.409070	-0.180757	-0.731381
C	0	-6.167788	0.927618	-1.085865
C	0	-7.195645	1.408258	-0.260217
C	0	-7.424829	0.727716	0.945392
C	0	-6.663615	-0.374201	1.314415
C	0	-8.003811	2.584305	-0.644844
C	0	-7.433482	3.666010	-1.338627
C	0	-8.187021	4.771584	-1.700966
C	0	-9.548157	4.834363	-1.377460
C	0	-10.138218	3.771920	-0.688039
C	0	-9.365484	2.666379	-0.333392
O	0	10.440491	5.952828	1.421971
C	0	9.890571	7.073089	2.092820
O	0	-10.203951	5.955613	-1.770821

C	0	-11.580144	6.068369	-1.453314
H	0	8.267884	1.050020	-1.715337
H	0	6.932607	-0.928642	-2.279130
H	0	4.731893	-0.490060	1.383171
H	0	6.075244	1.481178	1.955802
H	0	3.504831	-4.022074	0.127469
H	0	1.089687	-4.018089	0.545599
H	0	0.861712	0.049798	-0.825206
H	0	3.279474	0.047453	-1.229468
H	0	4.003180	-3.480002	-2.811323
H	0	5.082469	-5.468714	-3.801555
H	0	7.294382	-6.245825	-2.963375
H	0	8.400595	-5.006977	-1.108596
H	0	7.307332	-3.034202	-0.101389
H	0	9.935539	1.858502	-0.307995
H	0	11.265008	3.861469	0.248618
H	0	7.779939	5.680115	1.992711
H	0	6.460396	3.710919	1.410936
H	0	-0.774383	0.076126	0.790975
H	0	-3.192535	0.082822	1.192023
H	0	-3.408342	-4.031391	-0.024557
H	0	-0.992638	-4.037187	-0.439036
H	0	-7.208852	-3.046217	0.153385
H	0	-8.301176	-4.988067	1.220313
H	0	-7.201091	-6.159931	3.121663
H	0	-4.996312	-5.347621	3.944997
H	0	-3.918044	-3.389548	2.894173
H	0	-4.633474	-0.541505	-1.400441
H	0	-5.979030	1.405834	-2.043319
H	0	-8.189647	1.087498	1.628520
H	0	-6.851437	-0.867933	2.263255
H	0	-6.373766	3.652768	-1.578808
H	0	-7.734282	5.606527	-2.227561

H 0 -11.191787   3.784711   -0.432495

H 0   -9.850009   1.839829   0.179512

H 0   10.708301   7.784566   2.208578

H 0   9.088391   7.534739   1.505235

H 0   9.507519   6.796131   3.081931

H 0 -11.900974   7.032522   -1.847999

H 0 -12.164615   5.270437   -1.925993

H 0 -11.741370   6.047840   -0.369161

SCF Done: E(RPBE1PBE) = -2186.94945199   A.U. after   2 cycles

Zero-point correction=   0.768030 (Hartree/Particle)

Thermal correction to Energy=   0.812914

Thermal correction to Enthalpy=   0.813858

Thermal correction to Gibbs Free Energy=   0.681090

Sum of electronic and zero-point Energies=   -2186.181422

Sum of electronic and thermal Energies=   -2186.136538

Sum of electronic and thermal Enthalpies=   -2186.135594

Sum of electronic and thermal Free Energies=   -2186.268362

7.11.2 Computed excitations of compound 4a (PBE1PBE/6-31+G** PCM CH_2Cl_2)

Inputfile:

%NProcShared=10

%Chk=gauss.chk

%mem=10GB

#p PBE1PBE 6-31+G** geom=check guess=read

SCRF=(Solvent=Dichloromethane) TD=(Nstates=15,root=1)

Excitation analysis by Multiwfn:

Integral of hole: 0.966859

Integral of electron: 0.966025

Integral of transition density: -0.000836

Transition dipole moment in X/Y/Z: -4.168118 -0.009840   0.079840 a.u.

Transition magnetic dipole moment in X/Y/Z: 0.188676 -0.008594 3.104053 a.u.

Sm index (integral of Sm function): 0.40701 a.u.

Sr index (integral of Sr function): 0.67298 a.u.

Centroid of hole in X/Y/Z: 0.055356 -1.408174 0.004325 Angstrom

Centroid of electron in X/Y/Z: 0.035007 -1.062989 0.004618 Angstrom

D_x: 0.020 D_y: 0.345 D_z: 0.000 **D index: 0.346 Angstrom**

Variation of dipole moment with respect to ground state:

X: 0.037163 Y: -0.630416 Z: -0.000535 Norm: 0.631511 a.u.

RMSD of hole in X/Y/Z: 5.478 2.346 1.299 Norm: 6.099 Angstrom

RMSD of electron in X/Y/Z: 4.703 2.208 1.128 Norm: 5.316 Angstrom

Difference between RMSD of hole and electron (delta sigma):

X: -0.775 Y: -0.138 Z: -0.171 Overall: -0.783 Angstrom

H_x: 5.090 H_y: 2.277 H_z: 1.214 H_CT: 2.293 H index: 5.708 Angstrom

t index: -1.947 Angstrom

Hole delocalization index (HDI): 5.51

Electron delocalization index (EDI): 4.70

Ghost-hunter index: -37.668 eV, 1st term: 3.976 eV, 2nd term: 41.643 eV

Excitation energy of this state: 3.232 eV

Excitation energies and oscillator strengths:

Excited State 1: Singlet-A 3.2316 eV 383.66 nm f=1.3952 <S**2>=0.000

184 -> 187 -0.11068

185 -> 186 0.68649

This state for optimization and/or second-order correction.

Total Energy, E(TD-HF/TD-DFT) = -2186.83069112

Copying the excited state density for this state as the 1-particle RhoCI density.

Excited State 2: Singlet-A 3.5880 eV 345.55 nm f=0.7440 <S**2>=0.000

184 -> 186 -0.34216

184 -> 188 -0.19007

185 -> 187 0.55000

185 -> 189 -0.13957

Excited State 3: Singlet-A 3.5969 eV 344.69 nm f=0.0628 <S**2>=0.000

184 -> 190 -0.27255

185 -> 187 0.12093
185 -> 189 0.61425

Excited State 4: Singlet-A 3.6417 eV 340.46 nm f=0.0420 <S**2>=0.000
184 -> 189 -0.32286
185 -> 190 0.59646

Excited State 5: Singlet-A 3.6564 eV 339.09 nm f=0.1449 <S**2>=0.000
184 -> 187 -0.28781
185 -> 188 0.61252

Excited State 6: Singlet-A 3.7318 eV 332.23 nm f=0.0622 <S**2>=0.000
184 -> 186 0.57468
184 -> 188 -0.22656
185 -> 187 0.28613
185 -> 193 0.14122

Excited State 7: Singlet-A 4.1503 eV 298.73 nm f=0.0165 <S**2>=0.000
184 -> 188 0.10754
184 -> 190 0.39368
185 -> 189 0.14408
185 -> 191 -0.28445
185 -> 192 -0.11192
185 -> 194 -0.39089

Excited State 8: Singlet-A 4.1579 eV 298.19 nm f=0.1174 <S**2>=0.000
184 -> 186 0.14913
184 -> 188 0.45365
184 -> 190 -0.12648
185 -> 187 0.26538
185 -> 193 -0.35794
185 -> 194 0.11995

Excited State 9: Singlet-A 4.1760 eV 296.90 nm f=0.0636 <S**2>=0.000
184 -> 187 0.61946
185 -> 186 0.11965
185 -> 188 0.28887

Excited State 10:   Singlet-A   4.2073 eV 294.69 nm f=0.0213 <S**2>=0.000
184 -> 189   0.60073
185 -> 190   0.31755

Excited State 11:   Singlet-A   4.2411 eV 292.34 nm f=0.0097 <S**2>=0.000
184 -> 188   0.14178
184 -> 191   -0.24956
184 -> 192   -0.11071
184 -> 195   -0.10439
185 -> 191   -0.13208
185 -> 192   0.46179
185 -> 193   0.21218
185 -> 195   0.13719
185 -> 196   -0.15771
185 -> 197   0.15669

Excited State 12:   Singlet-A   4.2611 eV 290.97 nm f=0.0111 <S**2>=0.000
184 -> 190   0.25935
184 -> 191   0.10111
184 -> 192   -0.22307
184 -> 196   0.10419
185 -> 189   0.13327
185 -> 191   0.44752
185 -> 192   0.12295
185 -> 194   -0.13548
185 -> 195   0.17311
185 -> 196   0.17077

Excited State 13:   Singlet-A   4.3034 eV 288.11 nm f=0.0636 <S**2>=0.000
184 -> 188   0.37157
185 -> 191   0.16086
185 -> 192   -0.27398
185 -> 193   0.44616

Excited State 14:   Singlet-A   4.3371 eV 285.87 nm f=0.0196 <S**2>=0.000
184 -> 190   0.38277

184 -> 196 -0.10246

184 -> 197 -0.17953

185 -> 189 0.18985

185 -> 194 0.43978

185 -> 195 -0.18118

185 -> 196 -0.12574

Excited State 15: Singlet-A 4.3587 eV 284.45 nm f=0.0394 <S**2>=0.000

184 -> 194 -0.25767

184 -> 195 0.15330

185 -> 195 -0.29545

185 -> 196 0.25452

185 -> 197 0.45274

7.11.3 Computed emission of compound 4a (PBE1PBE/6-31+G PCM CH_2Cl_2)**

Inputfile:

%NProcShared=10

%Chk=gauss.chk

%mem=10GB

#p PBE1PBE 6-31+G** geom=check guess=read opt freq

SCRF=(Solvent=Dichloromethane) TD=(Nstates=6,root=1)

--link1--

%NProcShared=10

%Chk=gauss.chk

%mem=10GB

#p PBE1PBE 6-31+G** geom=check guess=read

SCRF=(Solvent=Dichloromethane) TD=(Nstates=6,root=1)

Excitation analysis by Multiwfn:

Integral of hole: 0.971759

Integral of electron: 0.971672

Integral of transition density: 0.000108

Transition dipole moment in X/Y/Z: -4.785629 0.028322 0.860634 a.u.

Sm index (integral of Sm function): 0.41630 a.u.

Sr index (integral of Sr function): 0.70026 a.u.

Centroid of hole in X/Y/Z: 0.054849 -1.236810 0.019762 Angstrom

Centroid of electron in X/Y/Z: 0.045238 -1.387077 0.025144 Angstrom

D_x: 0.010 D_y: 0.150 D_z: 0.005 **D index: 0.151 Angstrom**

Variation of dipole moment with respect to ground state:

X: 0.017650 Y: 0.275931 Z: -0.009882 Norm: 0.276672 a.u.

RMSD of hole in X/Y/Z: 5.255 1.995 1.736 Norm: 5.883 Angstrom

RMSD of electron in X/Y/Z: 3.913 1.694 1.357 Norm: 4.475 Angstrom

Difference between RMSD of hole and electron (delta sigma):

X: -1.341 Y: -0.302 Z: -0.379 Overall: -1.408 Angstrom

H_x: 4.584 H_y: 1.845 H_z: 1.547 H_CT: 1.864 H index: 5.179 Angstrom

t index: -1.713 Angstrom

Hole delocalization index (HDI): 5.31

Electron delocalization index (EDI): 5.46

Ghost-hunter index: -92.155 eV, 1st term: 3.416 eV, 2nd term: 95.571 eV

Excitation energy of this state: 2.757 eV

Excitation energies and oscillator strengths:

Excited State 1: Singlet-A 2.7575 eV 449.62 nm f=1.5601 <S**2>=0.000

185 -> 186 0.69702

This state for optimization and/or second-order correction.

Total Energy, E(TD-HF/TD-DFT) = -2186.83951762

Copying the excited state density for this state as the 1-particle RhoCI density.

Excited State 2: Singlet-A 3.3361 eV 371.64 nm f=0.1632 <S**2>=0.000

184 -> 186 0.66723

185 -> 187 0.19952

Excited State 3: Singlet-A 3.3919 eV 365.53 nm f=0.0132 <S**2>=0.000

184 -> 190 -0.17442

185 -> 189 0.66996

Excited State 4: Singlet-A 3.4456 eV 359.83 nm f=0.4450 <S**2>=0.000

184 -> 186 -0.19708

184 -> 188 0.21376

185 -> 187 0.63086

Excited State 5: Singlet-A 3.4693 eV 357.38 nm f=0.3158 <S**2>=0.000

184 -> 187 0.21985

185 -> 188 0.65864

Excited State 6: Singlet-A 3.5542 eV 348.84 nm f=0.0068 <S**2>=0.000

184 -> 189 -0.29962

185 -> 190 0.62261

7.12 Quantum chemical calculation data of *N*^{4},*N*$^{4'}$-Di([1,1'-biphenyl]-4-yl)-*N*^{4},*N*$^{4'}$-diphenyl-[1,1'-biphenyl]-4,4'-diamine (4b)

7.12.1 Computed xyz-coordinates of compound 4b (PBE1PBE/6-31+G** PCM CH_2Cl_2)

Input file:

%NProcShared=10

%Chk=gauss.chk

%mem=10GB

#p PBE1PBE 6-31+G**

pop=full GFInput opt freq SCRF=(Solvent=Dichloromethane)

Optimized S_0-geometry:

0 1

C 0 -7.539989 1.302246 0.641982

C 0 -6.782261 0.210391 1.045378

C 0 -5.709128 -0.239232 0.264034

C 0 -5.417065 0.442413 -0.925263

C 0 -6.171601 1.542378 -1.311990

C 0 -7.252451 1.997407 -0.542124

N 0 -4.940702 -1.351631 0.664537

C 0 -3.541800 -1.350589 0.475448

C 0 -5.569015 -2.472131 1.256112

C 0 -2.878868 -2.501893 0.030729

C 0 -1.501453 -2.498914 -0.147146

C 0 -0.731760 -1.351026 0.098177

C 0 -1.409167 -0.203007 0.538170

C 0 -2.784549 -0.199720 0.730424

C 0 -4.985814 -3.111855 2.357881

C 0 -5.601474 -4.220432 2.931962

C 0 -6.812101 -4.698715 2.430117

C 0 -7.397701 -4.058004 1.337908

C 0 -6.780601 -2.958780 0.747615

C 0 -8.057846 3.164388 -0.962594

C	0	-9.441883	3.206405	-0.733313
C	0	-10.200175	4.306144	-1.127075
C	0	-9.592467	5.388180	-1.763524
C	0	-8.218249	5.359509	-1.999907
C	0	-7.459825	4.261286	-1.602173
C	0	0.731741	-1.351025	-0.098179
C	0	1.409146	-0.203005	-0.538171
C	0	2.784529	-0.199717	-0.730424
C	0	3.541782	-1.350584	-0.475449
C	0	2.878851	-2.501890	-0.030731
C	0	1.501435	-2.498912	0.147143
N	0	4.940684	-1.351625	-0.664537
C	0	5.569001	-2.472121	-1.256116
C	0	5.709115	-0.239231	-0.264031
C	0	6.780589	-2.958764	-0.747619
C	0	7.397697	-4.057982	-1.337915
C	0	6.812103	-4.698692	-2.430129
C	0	5.601474	-4.220413	-2.931975
C	0	4.985807	-3.111842	-2.357891
C	0	5.417059	0.442414	0.925267
C	0	6.171605	1.542372	1.311995
C	0	7.252459	1.997393	0.542129
C	0	7.539990	1.302230	-0.641977
C	0	6.782252	0.210382	-1.045374
C	0	8.057864	3.164367	0.962600
C	0	7.459856	4.261263	1.602194
C	0	8.218291	5.359478	1.999928
C	0	9.592506	5.388145	1.763530
C	0	10.200201	4.306111	1.127065
C	0	9.441899	3.206379	0.733303
H	0	-8.349173	1.642726	1.282458
H	0	-7.015509	-0.294225	1.978105
H	0	-4.600147	0.099867	-1.553220

H	0	-5.936885	2.033197	-2.252693
H	0	-3.450183	-3.399582	-0.186137
H	0	-1.021555	-3.399271	-0.521042
H	0	-0.847196	0.696952	0.772685
H	0	-3.277873	0.697692	1.091760
H	0	-4.049562	-2.736771	2.760464
H	0	-5.136178	-4.704389	3.786312
H	0	-7.293062	-5.559950	2.884194
H	0	-8.336560	-4.423193	0.930711
H	0	-7.234918	-2.472219	-0.110380
H	0	-9.933547	2.359269	-0.262569
H	0	-11.271393	4.312631	-0.944960
H	0	-10.184011	6.245358	-2.072229
H	0	-7.731545	6.200028	-2.487114
H	0	-6.386013	4.264893	-1.769167
H	0	0.847175	0.696953	-0.772685
H	0	3.277851	0.697696	-1.091759
H	0	3.450166	-3.399578	0.186134
H	0	1.021538	-3.399271	0.521039
H	0	7.234901	-2.472203	0.110378
H	0	8.336557	-4.423167	-0.930718
H	0	7.293070	-5.559922	-2.884208
H	0	5.136184	-4.704369	-3.786329
H	0	4.049555	-2.736761	-2.760476
H	0	4.600140	0.099874	1.553226
H	0	5.936894	2.033190	2.252700
H	0	8.349177	1.642703	-1.282454
H	0	7.015497	-0.294237	-1.978101
H	0	6.386047	4.264876	1.769200
H	0	7.731596	6.199997	2.487148
H	0	10.184058	6.245318	2.072235
H	0	11.271417	4.312593	0.944938
H	0	9.933553	2.359244	0.262547

SCF Done: E(RPBE1PBE) = -1958.13779290 A.U. after 2 cycles

Zero-point correction= 0.702661 (Hartree/Particle)

Thermal correction to Energy= 0.742303

Thermal correction to Enthalpy= 0.743247

Thermal correction to Gibbs Free Energy= 0.622785

Sum of electronic and zero-point Energies= -1957.435132

Sum of electronic and thermal Energies= -1957.395490

Sum of electronic and thermal Enthalpies= -1957.394546

Sum of electronic and thermal Free Energies= -1957.515008

7.12.2 Computed excitations of compound 4b (PBE1PBE/6-31+G** PCM CH_2Cl_2)

Input file:

%NProcShared=10

%Chk=gauss.chk

%mem=10GB

#p PBE1PBE 6-31+G** geom=check guess=read

SCRF=(Solvent=Dichloromethane) TD=(Nstates=15,root=1)

Excitation analysis by Multiwfn:

Integral of hole: 0.959751

Integral of electron: 0.959821

Integral of transition density: 0.000014

Transition dipole moment in X/Y/Z: 4.033951 -0.000152 0.258511 a.u.

Sm index (integral of Sm function): 0.38538 a.u.

Sr index (integral of Sr function): 0.64407 a.u.

Centroid of hole in X/Y/Z: -0.000175 -1.016313 0.000002 Angstrom

Centroid of electron in X/Y/Z: -0.000202 0.142497 0.000005 Angstrom

D_x: 0.000 D_y: 1.159 D_z: 0.000 **D index: 1.159 Angstrom**

Variation of dipole moment with respect to ground state:

X: 0.000049 Y: -2.101772 Z: -0.000007 Norm: 2.101772 a.u.

RMSD of hole in X/Y/Z: 5.291 2.058 1.243 Norm: 5.812 Angstrom

RMSD of electron in X/Y/Z: 5.386 2.420 1.171 Norm: 6.019 Angstrom

Difference between RMSD of hole and electron (delta sigma):

X: 0.095 Y: 0.362 Z: -0.072 Overall: 0.208 Angstrom

H_x: 5.338 H_y: 2.239 H_z: 1.207 H_CT: 2.239 H index: 5.915 Angstrom

t index: -1.080 Angstrom

Hole delocalization index (HDI): 5.66

Electron delocalization index (EDI): 4.21

Ghost-hunter index: -8.455 eV, 1st term: 3.971 eV, 2nd term: 12.426 eV

Excitation energy of this state: 3.228 eV

Excitation energies and oscillator strengths:

Excited State 1: Singlet-A 3.2282 eV 384.06 nm f=1.3416 <S**2>=0.000
168 -> 171 0.12943
169 -> 170 0.68056
This state for optimization and/or second-order correction.
Total Energy, E(TD-HF/TD-DFT) = -1958.01915743
Copying the excited state density for this state as the 1-particle RhoCI density.

Excited State 2: Singlet-A 3.5463 eV 349.61 nm f=0.7228 <S**2>=0.000
168 -> 170 0.32978
168 -> 172 0.16320
169 -> 171 0.59230

Excited State 3: Singlet-A 3.6219 eV 342.32 nm f=0.0078 <S**2>=0.000
168 -> 171 0.11821
168 -> 174 0.24824
169 -> 172 0.35561
169 -> 173 0.53146

Excited State 4: Singlet-A 3.6420 eV 340.43 nm f=0.1752 <S**2>=0.000
168 -> 171 0.24383
168 -> 174 -0.13218
169 -> 172 0.52328
169 -> 173 -0.35318

Excited State 5: Singlet-A 3.6735 eV 337.51 nm f=0.0159 <S**2>=0.000
168 -> 173 0.33443
169 -> 174 0.60893

Excited State 6: Singlet-A 3.7440 eV 331.16 nm f=0.0200 <S**2>=0.000
168 -> 170 0.58614
168 -> 172 -0.25180

169 -> 171 -0.25152
169 -> 175 -0.13098

Excited State 7: Singlet-A 4.1213 eV 300.83 nm f=0.0534 <S**2>=0.000
168 -> 171 0.62565
169 -> 170 -0.15906
169 -> 172 -0.26757

Excited State 8: Singlet-A 4.1221 eV 300.78 nm f=0.1558 <S**2>=0.000
168 -> 170 0.19023
168 -> 172 0.50543
169 -> 171 -0.27461
169 -> 175 0.31168
169 -> 177 0.12309

Excited State 9: Singlet-A 4.1910 eV 295.84 nm f=0.0136 <S**2>=0.000
168 -> 174 0.41053
168 -> 182 0.10545
169 -> 173 -0.15766
169 -> 176 0.25757
169 -> 178 0.43636

Excited State 10: Singlet-A 4.2519 eV 291.60 nm f=0.0073 <S**2>=0.000
168 -> 173 0.60769
169 -> 174 -0.33142

Excited State 11: Singlet-A 4.2946 eV 288.70 nm f=0.0318 <S**2>=0.000
168 -> 172 -0.31041
168 -> 176 0.15090
168 -> 179 0.14677
169 -> 175 0.30816
169 -> 177 0.39295
169 -> 180 -0.20211
169 -> 182 0.18828

Excited State 12: Singlet-A 4.3285 eV 286.43 nm f=0.0214 <S**2>=0.000
168 -> 174 -0.24451

168 -> 177 0.22161
168 -> 180 -0.19792
169 -> 173 0.12322
169 -> 176 0.41093
169 -> 179 0.37065

Excited State 13: Singlet-A 4.3410 eV 285.61 nm f=0.0027 <S**2>=0.000
168 -> 172 -0.21018
168 -> 176 -0.19877
168 -> 179 -0.11278
169 -> 175 0.49174
169 -> 177 -0.26359
169 -> 180 0.23742

Excited State 14: Singlet-A 4.3760 eV 283.33 nm f=0.0148 <S**2>=0.000
168 -> 174 0.40854
168 -> 180 -0.14801
168 -> 182 -0.18016
169 -> 173 -0.20545
169 -> 178 -0.40617
169 -> 179 0.22770

Excited State 15: Singlet-A 4.3888 eV 282.50 nm f=0.0300 <S**2>=0.000
168 -> 178 0.26801
168 -> 179 -0.16190
169 -> 180 0.35520
169 -> 182 0.47662

7.12.3 Computed emission of compound 4b (PBE1PBE/6-31+G** PCM CH_2Cl_2)

Input file:

%NProcShared=10
%Chk=gauss.chk
%mem=10GB
#p PBE1PBE 6-31+G** geom=check guess=read opt freq
SCRF=(Solvent=Dichloromethane) TD=(Nstates=6,root=1)
--link1--
%NProcShared=10

%Chk=gauss.chk

%mem=10GB

#p PBE1PBE 6-31+G** geom=check guess=read

SCRF=(Solvent=Dichloromethane) TD=(Nstates=6,root=1)

Excitation analysis by Multiwfn:

Integral of hole: 0.970404

Integral of electron: 0.970443

Integral of transition density: -0.000027

Transition dipole moment in X/Y/Z: 4.881729 -0.000059 -0.417298 a.u.

Sm index (integral of Sm function): 0.43497 a.u.

Sr index (integral of Sr function): 0.70480 a.u.

Centroid of hole in X/Y/Z: -0.000070 -0.924427 -0.000018 Angstrom

Centroid of electron in X/Y/Z: 0.000002 -0.615036 0.000012 Angstrom

D_x: 0.000 D_y: 0.309 D_z: 0.000 **D index: 0.309 Angstrom**

Variation of dipole moment with respect to ground state:

X: -0.000134 Y: -0.567372 Z: -0.000056 Norm: 0.567372 a.u.

RMSD of hole in X/Y/Z: 5.010 1.785 1.520 Norm: 5.531 Angstrom

RMSD of electron in X/Y/Z: 4.360 1.888 1.271 Norm: 4.918 Angstrom

Difference between RMSD of hole and electron (delta sigma):

X: -0.650 Y: 0.102 Z: -0.249 Overall: -0.613 Angstrom

H_x: 4.685 H_y: 1.836 H_z: 1.395 H_CT: 1.836 H index: 5.225 Angstrom

t index: -1.527 Angstrom

Hole delocalization index (HDI): 5.48

Electron delocalization index (EDI): 5.13

Ghost-hunter index: -43.106 eV, 1st term: 3.436 eV, 2nd term: 46.542 eV

Excitation energy of this state: 2.773 eV

Excitation energies and oscillator strengths:

Excited State 1: Singlet-A 2.7734 eV 447.05 nm f=1.5707 <S**2>=0.000

169 -> 170 0.69659

This state for optimization and/or second-order correction.

Total Energy, E(TD-HF/TD-DFT) = -1958.02751877

Copying the excited state density for this state as the 1-particle RhoCI density.

Excited State 2: Singlet-A 3.3354 eV 371.73 nm f=0.4927 <S**2>=0.000

168 -> 170 0.30381

168 -> 172 0.14533
169 -> 171 0.61342

Excited State 3: Singlet-A 3.4064 eV 363.97 nm f=0.1384 <S**2>=0.000
168 -> 171 0.11592
168 -> 174 0.12593
169 -> 172 0.40648
169 -> 173 0.53570

Excited State 4: Singlet-A 3.4190 eV 362.63 nm f=0.1133 <S**2>=0.000
168 -> 171 0.14657
168 -> 174 -0.11850
169 -> 172 0.53105
169 -> 173 -0.41027

Excited State 5: Singlet-A 3.4413 eV 360.28 nm f=0.0124 <S**2>=0.000
168 -> 170 0.62659
168 -> 172 -0.12293
169 -> 171 -0.28104

Excited State 6: Singlet-A 3.5755 eV 346.76 nm f=0.0118 <S**2>=0.000
168 -> 173 0.28202
169 -> 174 0.63411

7.13 Quantum chemical calculation data of 4',4'''-([1,1'-Biphenyl]-4,4'-diylbis(phenylazanediyl))bis(([1,1'-biphenyl]-4-carbonitrile)) (4c)

7.13.1 Computed xyz-coordinates of compound 4c (PBE1PBE/6-31+G** PCM CH_2Cl_2)

Inputfile:

%NProcShared=10

%Chk=gauss.chk

%mem=10GB

#p PBE1PBE 6-31+G** pop=full GFInput opt freq SCRF=(Solvent=Dichloromethane)

Optimized S_0-geometry:

0 1

C 0 -7.590352 0.773644 0.897797

C 0 -6.801464 -0.307091 1.264313

C 0 -5.707444 -0.693090 0.474821

C 0 -5.429084 0.045710 -0.685379

C 0 -6.213775 1.135555 -1.033705

C 0 -7.315137 1.524280 -0.255509

N 0 -4.913352 -1.794135 0.836807

C 0 -3.521425 -1.786861 0.594151

C 0 -5.504003 -2.926354 1.451293

C 0 -2.876744 -2.934838 0.116728

C 0 -1.505989 -2.932033 -0.106991

C 0 -0.728096 -1.786767 0.122592

C 0 -1.387608 -0.641804 0.596030

C 0 -2.755543 -0.639238 0.835906

C 0 -4.894359 -3.525413 2.560304

C 0 -5.468855 -4.645805 3.154249

C 0 -6.662880 -5.173766 2.662757

C 0 -7.274066 -4.573308 1.561724

C 0 -6.697356 -3.462691 0.952082

C 0 -8.152262 2.677620 -0.635213

C 0 -9.533550 2.683814 -0.376564

C	0	-10.326386	3.765796	-0.728025
C	0	-9.748186	4.877011	-1.358590
C	0	-8.371850	4.888267	-1.627379
C	0	-7.590833	3.801161	-1.265417
C	0	0.728098	-1.786767	-0.122583
C	0	1.387610	-0.641804	-0.596020
C	0	2.755545	-0.639238	-0.835898
C	0	3.521426	-1.786861	-0.594146
C	0	2.876746	-2.934839	-0.116722
C	0	1.505991	-2.932034	0.106999
N	0	4.913352	-1.794136	-0.836804
C	0	5.504003	-2.926353	-1.451295
C	0	5.707445	-0.693091	-0.474820
C	0	6.697356	-3.462694	-0.952086
C	0	7.274064	-4.573309	-1.561733
C	0	6.662877	-5.173762	-2.662767
C	0	5.468852	-4.645799	-3.154256
C	0	4.894356	-3.525409	-2.560307
C	0	5.429087	0.045708	0.685381
C	0	6.213777	1.135554	1.033706
C	0	7.315138	1.524279	0.255508
C	0	7.590351	0.773645	-0.897800
C	0	6.801463	-0.307091	-1.264315
C	0	8.152263	2.677619	0.635211
C	0	7.590832	3.801164	1.265408
C	0	8.371849	4.888270	1.627371
C	0	9.748186	4.877010	1.358589
C	0	10.326388	3.765793	0.728030
C	0	9.533552	2.683810	0.376569
C	0	-10.558749	5.994576	-1.726265
N	0	-11.218445	6.904674	-2.025834
C	0	10.558750	5.994574	1.726266
N	0	11.218439	6.904677	2.025834

H	0	-8.412297	1.062163	1.547120
H	0	-7.025411	-0.852758	2.175534
H	0	-4.599852	-0.245992	-1.322174
H	0	-5.987219	1.667183	-1.953880
H	0	-3.456789	-3.829798	-0.087773
H	0	-1.039161	-3.829453	-0.503695
H	0	-0.815748	0.254634	0.819931
H	0	-3.234592	0.254208	1.225191
H	0	-3.970404	-3.110249	2.951981
H	0	-4.985010	-5.100011	4.014481
H	0	-7.111765	-6.044165	3.131947
H	0	-8.199739	-4.979270	1.163301
H	0	-7.168800	-3.006614	0.086498
H	0	-9.999546	1.818567	0.084728
H	0	-11.392827	3.750788	-0.527515
H	0	-7.920728	5.751127	-2.106629
H	0	-6.522198	3.835623	-1.453328
H	0	0.815750	0.254635	-0.819919
H	0	3.234594	0.254209	-1.225182
H	0	3.456791	-3.829799	0.087777
H	0	1.039163	-3.829453	0.503703
H	0	7.168800	-3.006619	-0.086502
H	0	8.199737	-4.979273	-1.163312
H	0	7.111761	-6.044161	-3.131961
H	0	4.985005	-5.100002	-4.014489
H	0	3.970402	-3.110243	-2.951982
H	0	4.599856	-0.245995	1.322177
H	0	5.987224	1.667182	1.953882
H	0	8.412295	1.062164	-1.547123
H	0	7.025408	-0.852757	-2.175536
H	0	6.522196	3.835626	1.453313
H	0	7.920726	5.751132	2.106616
H	0	11.392830	3.750782	0.527525

H 0 9.999548 1.818561 -0.084718

SCF Done: E(RPBE1PBE) = -2142.42934589 A.U. after 3 cycles
Zero-point correction= 0.699898 (Hartree/Particle)
Thermal correction to Energy= 0.743224
Thermal correction to Enthalpy= 0.744168
Thermal correction to Gibbs Free Energy= 0.614416
Sum of electronic and zero-point Energies= -2141.729448
Sum of electronic and thermal Energies= -2141.686122
Sum of electronic and thermal Enthalpies= -2141.685178
Sum of electronic and thermal Free Energies= -2141.814930

7.13.2 Computed excitations of compound 4c (PBE1PBE/6-31+G PCM CH_2Cl_2)**

Inputfile:

%NProcShared=10

%Chk=gauss.chk

%mem=10GB

#p PBE1PBE 6-31+G** geom=check guess=read

SCRF=(Solvent=Dichloromethane) TD=(Nstates=15,root=1)

Excitation analysis with Multiwfn:

Integral of hole: 0.983384
Integral of electron: 0.983539
Integral of transition density: -0.000007
Transition dipole moment in X/Y/Z: -3.678351 -0.000017 -0.701379 a.u.
Sm index (integral of Sm function): 0.27010 a.u.
Sr index (integral of Sr function): 0.52890 a.u.
Centroid of hole in X/Y/Z: -0.000130 -1.406523 0.000013 Angstrom
Centroid of electron in X/Y/Z: 0.000606 2.313632 0.000045 Angstrom
D_x: 0.001 D_y: 3.720 D_z: 0.000 **D index: 3.720 Angstrom**
Variation of dipole moment with respect to ground state:
X: -0.001368 Y: -6.913806 Z: -0.000060 Norm: 6.913807 a.u.
RMSD of hole in X/Y/Z: 5.325 2.113 1.312 Norm: 5.878 Angstrom
RMSD of electron in X/Y/Z: 8.002 2.930 1.351 Norm: 8.628 Angstrom
Difference between RMSD of hole and electron (delta sigma):

X: 2.676 Y: 0.817 Z: 0.039   Overall: 2.750 Angstrom

H_x: 6.663 H_y: 2.522 H_z: 1.332 H_CT: 2.522 H index: 7.253 Angstrom

t index: 1.198 Angstrom

Hole delocalization index (HDI):   5.79

Electron delocalization index (EDI):   4.11

Ghost-hunter index: -0.308 eV, 1st term: 3.563 eV, 2nd term: 3.871 eV

Excitation energy of this state: 2.952 eV

Excitation energies and oscillator strengths:

Excited State   1:   Singlet-A   2.9519 eV 420.01 nm f=0.9938 <S**2>=0.000

180 -> 183   0.21581

181 -> 182   0.65165

181 -> 184   -0.14342

This state for optimization and/or second-order correction.

Total Energy, E(TD-HF/TD-DFT) = -2142.32086493

Copying the excited state density for this state as the 1-particle RhoCI density.

Excited State   2:   Singlet-A   3.0734 eV 403.42 nm f=0.7113 <S**2>=0.000

180 -> 182   0.26151

181 -> 183   0.65020

Excited State   3:   Singlet-A   3.4695 eV 357.35 nm f=0.1461 <S**2>=0.000

180 -> 182   0.64196

180 -> 184   -0.11203

181 -> 183   -0.26295

Excited State   4:   Singlet-A   3.4901 eV 355.25 nm f=0.4702 <S**2>=0.000

180 -> 183   0.25565

181 -> 184   0.63645

Excited State   5:   Singlet-A   3.5430 eV 349.94 nm f=0.1772 <S**2>=0.000

180 -> 183   0.61323

181 -> 182   -0.26018

181 -> 184   -0.22047

Excited State   6:   Singlet-A   3.6662 eV 338.19 nm f=0.0389 <S**2>=0.000

180 -> 186   0.27613

181 -> 185 0.62086
181 -> 187 0.12123

Excited State 7: Singlet-A 3.6994 eV 335.15 nm f=0.0130 <S**2>=0.000
180 -> 185 0.32702
181 -> 186 0.58470
181 -> 188 -0.16744

Excited State 8: Singlet-A 3.8927 eV 318.50 nm f=0.1094 <S**2>=0.000
180 -> 184 0.63687
181 -> 189 0.22260

Excited State 9: Singlet-A 4.1110 eV 301.59 nm f=0.0002 <S**2>=0.000
180 -> 186 -0.15486
180 -> 188 -0.22523
181 -> 187 0.60896
181 -> 191 -0.14256

Excited State 10: Singlet-A 4.1218 eV 300.80 nm f=0.0004 <S**2>=0.000
180 -> 187 -0.27890
181 -> 186 0.18182
181 -> 188 0.60024

Excited State 11: Singlet-A 4.1562 eV 298.31 nm f=0.0539 <S**2>=0.000
180 -> 184 -0.23504
180 -> 190 0.25154
181 -> 189 0.59462

Excited State 12: Singlet-A 4.1788 eV 296.69 nm f=0.0166 <S**2>=0.000
180 -> 189 0.29057
181 -> 190 0.58815
181 -> 191 -0.14891

Excited State 13: Singlet-A 4.2519 eV 291.59 nm f=0.0234 <S**2>=0.000
180 -> 186 0.40218
180 -> 188 -0.18911
181 -> 185 -0.22069

181 -> 190 0.17200
181 -> 191 0.41185

Excited State 14: Singlet-A 4.2891 eV 289.07 nm f=0.0059 <S**2>=0.000
180 -> 185 0.59478
181 -> 186 -0.31719
181 -> 188 0.10273

Excited State 15: Singlet-A 4.4040 eV 281.52 nm f=0.0301 <S**2>=0.000
180 -> 186 -0.31771
180 -> 192 -0.21191
180 -> 194 0.17971
181 -> 185 0.12952
181 -> 191 0.32649
181 -> 193 0.39295

7.13.3 Computed emission of compound 4c (PBE1PBE/6-31+G** PCM CH_2Cl_2)

Inputfile:

```
%NProcShared=10
%Chk=gauss.chk
%mem=10GB
#p PBE1PBE 6-31+G** geom=check guess=read opt freq
SCRF=(Solvent=Dichloromethane) TD=(Nstates=6,root=1)
--link1--
%NProcShared=10
%Chk=gauss.chk
%mem=10GB
#p PBE1PBE 6-31+G** geom=check guess=read
SCRF=(Solvent=Dichloromethane) TD=(Nstates=6,root=1)
```

Excitation analysis by Multiwfn:

Integral of hole: 0.979179
Integral of electron: 0.978961
Integral of transition density: -0.000026
Transition dipole moment in X/Y/Z: -2.111610 -2.036618 -0.381244 a.u.
Sm index (integral of Sm function): 0.11813 a.u.
Sr index (integral of Sr function): 0.35979 a.u.

Centroid of hole in X/Y/Z: 1.500546 -1.772859 -0.153861 Angstrom

Centroid of electron in X/Y/Z: 7.990497 3.137047 0.514828 Angstrom

D_x: 6.490 D_y: 4.910 D_z: 0.669 **D index: 8.165 Angstrom**

Variation of dipole moment with respect to ground state:

X: -12.007540 Y: -9.084181 Z: -1.237192 Norm: 15.107415 a.u.

RMSD of hole in X/Y/Z: 4.481 1.914 1.009 Norm: 4.976 Angstrom

RMSD of electron in X/Y/Z: 2.224 2.771 1.126 Norm: 3.727 Angstrom

Difference between RMSD of hole and electron (delta sigma):

X: -2.257 Y: 0.857 Z: 0.117 Overall: -1.249 Angstrom

H_x: 3.352 H_y: 2.342 H_z: 1.067 H_CT: 3.015 H index: 4.351 Angstrom

t index: 5.150 Angstrom

Hole delocalization index (HDI): 6.25

Electron delocalization index (EDI): 6.48

Ghost-hunter index: 1.130 eV, 1st term: 2.893 eV, 2nd term: 1.763 eV

Excitation energy of this state: 2.411 eV

Excitation energies and oscillator strengths:

Excitation energies and oscillator strengths:

Excited State 1: Singlet-A 2.3806 eV 520.82 nm f=0.5967 <S**2>=0.000

180 ->182 0.14224

181 ->182 -0.68483

This state for optimization and/or second-order correction.

Total Energy, E(TD-HF/TD-DFT) = -2141.76921148

Copying the excited state density for this state as the 1-particle RhoCI density.

Excited State 2: Singlet-A 2.7837 eV 445.40 nm f=0.7355 <S**2>=0.000

180 ->183 -0.14665

181 ->183 -0.67623

181 ->184 0.10007

Excited State 3: Singlet-A 3.1487 eV 393.77 nm f=0.2983 <S**2>=0.000

180 ->182 0.67850

181 ->182 0.15393

Excited State 4: Singlet-A 3.2869 eV 377.21 nm f=1.0332 <S**2>=0.000

181 ->184 -0.68188

Excited State 5: Singlet-A 3.5115 eV 353.08 nm f=0.0594 <S**2>=0.000

180 ->183 0.17705

180 ->185 0.10571

180 ->186 0.11199

181 ->185 -0.57558

181 ->186 0.24680

181 ->187 0.11757

181 ->188 -0.11460

Excited State 6: Singlet-A 3.5406 eV 350.18 nm f=0.1524 <S**2>=0.000

180 ->183 -0.64543

181 ->183 0.15041

181 ->185 -0.18227

7.14 Quantum chemical calculation data of *N*4″,*N*4″,*N*5′,*N*5′-tetraphenyl-[1,1':2',1'':2'',1'''-quaterphenyl]-4'',5'-diamine (5b)

7.14.1 Computed xyz-coordinates of compound 5b (PBE1PBE/6-31+G** PCM CH_2Cl_2)

Inputfile:

%NProcShared=10

%Chk=gauss.chk

%mem=10GB

#p PBE1PBE 6-31+G** pop=full GFInput opt freq SCRF=(Solvent=Dichloromethane)

Optimized S_0-geometry:

0 1

C	0	1.333932	-0.607903	3.534084
C	0	0.823726	-1.259489	4.655459
C	0	-0.098761	-2.293566	4.505384
C	0	-0.506869	-2.668116	3.225067
C	0	-0.000850	-2.011648	2.106359
C	0	0.928760	-0.971374	2.242229
C	0	6.740193	1.610613	-1.386165
C	0	7.358110	2.767025	-1.853262
C	0	6.845782	4.024628	-1.533473
C	0	5.708631	4.111844	-0.730096
C	0	5.094339	2.960937	-0.244735
C	0	5.413895	-1.876539	0.037205
C	0	6.186834	-2.940920	0.492161
C	0	7.327379	-2.711631	1.262214
C	0	7.682013	-1.400404	1.580004
C	0	6.904819	-0.331156	1.143705
C	0	5.763498	-0.559264	0.363398
C	0	5.602335	1.696554	-0.572501
N	0	4.976890	0.524869	-0.089289
C	0	1.415184	0.841550	-1.070254
C	0	2.797292	0.942876	-1.117205

C 0	3.568540	0.436268	-0.064111
C 0	2.912437	-0.166686	1.012050
C 0	1.518404	-0.289764	1.058229
C 0	0.743668	0.228262	-0.003112
C 0	-1.333835	-0.602025	-3.535030
C 0	-0.823544	-1.251742	-4.657450
C 0	0.098996	-2.286011	-4.509033
C 0	0.507079	-2.662632	-3.229315
C 0	0.000972	-2.008033	-2.109553
C 0	-0.928708	-0.967606	-2.243756
C 0	-5.413928	-1.876519	-0.040003
C 0	-6.186828	-2.940226	-0.496597
C 0	-7.327337	-2.709796	-1.266363
C 0	-7.681974	-1.398098	-1.582201
C 0	-6.904818	-0.329496	-1.144253
C 0	-6.740358	1.608462	1.388536
C 0	-7.358280	2.764162	1.857385
C 0	-6.845874	4.022247	1.539625
C 0	-5.708631	4.110682	0.736512
C 0	-5.094331	2.960516	0.249405
C 0	-5.763536	-0.558760	-0.364230
C 0	-5.602415	1.695638	0.575120
N 0	-4.976960	0.524700	0.090129
C 0	-0.743737	0.228278	0.003561
C 0	-1.415279	0.839844	1.071676
C 0	-2.797390	0.941081	1.118758
C 0	-3.568606	0.436162	0.064830
C 0	-2.912473	-0.165020	-1.012304
C 0	-1.518435	-0.287989	-1.058654
H 0	2.044547	0.204843	3.661152
H 0	1.146030	-0.954969	5.647433
H 0	-0.496904	-2.803738	5.377921
H 0	-1.220666	-3.477060	3.095448

H	0	-0.328448	-2.314817	1.116515
H	0	7.137364	0.634760	-1.649187
H	0	8.239134	2.681548	-2.483498
H	0	7.326494	4.924644	-1.905108
H	0	5.301114	5.083846	-0.465771
H	0	4.216689	3.036782	0.390298
H	0	4.533804	-2.061159	-0.571659
H	0	5.900946	-3.956004	0.230283
H	0	7.931795	-3.544000	1.610181
H	0	8.563558	-1.205410	2.184613
H	0	7.179720	0.686538	1.404601
H	0	0.830183	1.251488	-1.888901
H	0	3.280278	1.417983	-1.965475
H	0	3.502215	-0.577437	1.826278
H	0	-2.044488	0.210890	-3.660794
H	0	-1.145820	-0.945610	-5.648937
H	0	0.497207	-2.794727	-5.382389
H	0	1.220926	-3.471739	-3.100994
H	0	0.328553	-2.312799	-1.120191
H	0	-4.533864	-2.062040	0.568626
H	0	-5.900938	-3.955700	-0.236235
H	0	-7.931723	-3.541650	-1.615612
H	0	-8.563488	-1.202209	-2.186564
H	0	-7.179718	0.688586	-1.403632
H	0	-7.137592	0.632213	1.649989
H	0	-8.239372	2.677726	2.487396
H	0	-7.326593	4.921696	1.912621
H	0	-5.301046	5.083082	0.473763
H	0	-4.216608	3.037328	-0.385410
H	0	-0.830301	1.248451	1.891002
H	0	-3.280407	1.414806	1.967784
H	0	-3.502229	-0.574442	-1.827217

SCF Done: E(RPBE1PBE) = -1958.12444871 A.U. after 16 cycles

Zero-point correction= 0.702069 (Hartree/Particle)

Thermal correction to Energy= 0.741976

Thermal correction to Enthalpy= 0.742920

Thermal correction to Gibbs Free Energy= 0.622650

Sum of electronic and zero-point Energies= -1957.423410

Sum of electronic and thermal Energies= -1957.383503

Sum of electronic and thermal Enthalpies= -1957.382558

Sum of electronic and thermal Free Energies= -1957.502829

7.14.2 Computed excitations of compound 5b (PBE1PBE/6-31+G PCM CH_2Cl_2)**

Inputfile:

%NProcShared=10

%Chk=gauss.chk

%mem=10GB

#p PBE1PBE 6-31+G** geom=check guess=read SCRF=(Solvent=Dichloromethane) TD=(Nstates=15,root=1)

Excitation analysis by Multiwfn:

Integral of hole: 0.947614

Integral of electron: 0.947515

Integral of transition density: 0.000002

Transition dipole moment in X/Y/Z: 2.415619 -0.000677 0.881051 a.u.

Sm index (integral of Sm function): 0.31457 a.u.

Sr index (integral of Sr function): **0.57538 a.u.**

Centroid of hole in X/Y/Z: 0.000104 0.474650 0.000372 Angstrom

Centroid of electron in X/Y/Z: -0.000147 -0.234005 -0.000270 Angstrom

D_x: 0.000 D_y: 0.709 D_z: 0.001 D index: **0.709 Angstrom**

Variation of dipole moment with respect to ground state:

X: 0.000450 Y: 1.268944 Z: 0.001149 Norm: 1.268944 a.u.

RMSD of hole in X/Y/Z: 5.005 1.715 1.107 Norm: 5.405 Angstrom

RMSD of electron in X/Y/Z: 3.001 1.591 2.251 Norm: 4.074 Angstrom

Difference between RMSD of hole and electron (delta sigma):

X: -2.005 Y: -0.124 Z: 1.144 Overall: -1.331 Angstrom

H_x: 4.003 H_y: 1.653 H_z: 1.679 H_CT: 1.653 H index: 4.740 Angstrom

t index: -0.944 Angstrom

Hole delocalization index (HDI):   5.91

Electron delocalization index (EDI):   4.26

Ghost-hunter index: -16.202 eV, 1st term: 4.118 eV, 2nd term: 20.320 eV

Excitation energy of this state: 3.389 eV

Excitation energies and oscillator strengths:

Excited State   1:   Singlet-A   3.3886 eV 365.89 nm f=0.5579 <S**2>=0.000
    169 -> 170   0.68831
This state for optimization and/or second-order correction.
Total Energy, E(TD-HF/TD-DFT) = -1958.00095153
Copying the excited state density for this state as the 1-particle RhoCI density.

Excited State   2:   Singlet-A   3.6262 eV 341.91 nm f=0.3612 <S**2>=0.000
    168 -> 172   0.20675
    168 -> 175   0.12372
    169 -> 171   0.65276

Excited State   3:   Singlet-A   3.6281 eV 341.73 nm f=0.0045 <S**2>=0.000
    168 -> 170   -0.40417
    168 -> 171   0.20691
    169 -> 172   0.51692

Excited State   4:   Singlet-A   3.7897 eV 327.16 nm f=0.0022 <S**2>=0.000
    168 -> 170   0.55014
    168 -> 171   0.25977
    169 -> 172   0.31419
    169 -> 178   -0.10853

Excited State   5:   Singlet-A   3.9468 eV 314.14 nm f=0.3871 <S**2>=0.000
    168 -> 174   0.35052
    169 -> 173   0.58571

Excited State   6:   Singlet-A   3.9475 eV 314.08 nm f=0.0780 <S**2>=0.000
    168 -> 173   0.36587

169 -> 174 0.59258

Excited State 7: Singlet-A 4.0242 eV 308.10 nm f=0.0201 <S**2>=0.000
168 -> 170 -0.11911
168 -> 171 0.47276
169 -> 172 -0.32499
169 -> 173 -0.11557
169 -> 175 0.34433

Excited State 8: Singlet-A 4.0266 eV 307.92 nm f=0.0648 <S**2>=0.000
168 -> 172 0.47377
168 -> 178 0.11353
169 -> 170 0.10435
169 -> 171 -0.16763
169 -> 176 0.44211

Excited State 9: Singlet-A 4.1674 eV 297.51 nm f=0.0663 <S**2>=0.000
168 -> 171 -0.27548
168 -> 176 0.31769
169 -> 175 0.47287
169 -> 178 0.26077

Excited State 10: Singlet-A 4.2346 eV 292.79 nm f=0.0009 <S**2>=0.000
168 -> 172 0.44453
168 -> 175 -0.27100
168 -> 178 -0.11441
169 -> 176 -0.41825

Excited State 11: Singlet-A 4.3378 eV 285.83 nm f=0.0239 <S**2>=0.000
168 -> 171 0.24961
168 -> 176 0.15850
169 -> 175 -0.27507
169 -> 177 -0.23740
169 -> 178 0.48384

Excited State 12: Singlet-A 4.3921 eV 282.29 nm f=0.0002 <S**2>=0.000
169 -> 177 0.63281

169 -> 178 0.24632

Excited State 13: Singlet-A 4.4329 eV 279.69 nm f=0.0083 <S**2>=0.000
169 -> 179 0.67381
169 -> 180 0.14493

Excited State 14: Singlet-A 4.4491 eV 278.67 nm f=0.0178 <S**2>=0.000
168 -> 175 -0.19647
168 -> 178 0.14609
168 -> 181 0.31045
169 -> 179 -0.17848
169 -> 180 0.51366

Excited State 15: Singlet-A 4.4768 eV 276.95 nm f=0.0060 <S**2>=0.000
168 -> 180 0.34998
169 -> 177 0.13812
169 -> 181 0.56441

7.14.3 Computed emission of compound 5b (PBE1PBE/6-31+G** PCM CH_2Cl_2)

Inputfile:

```
%NProcShared=20
%Chk=gauss.chk
%mem=20GB
#p PBE1PBE 6-31+G** opt freq
SCRF=(Solvent=Dichloromethane) TD=(Nstates=3,root=1)
--link1--
%NProcShared=20
%Chk=gauss.chk
%mem=20GB
#p PBE1PBE 6-31+G** geom=check guess=read
SCRF=(Solvent=Dichloromethane) TD=(Nstates=3,root=1)
```

Excitation analysis by Multiwfn:

Integral of hole: 0.984330

Integral of electron: 0.984356

Integral of transition density: -0.000000

Transition dipole moment in X/Y/Z: -3.732843 -0.650541 -0.000045 a.u.

Sm index (integral of Sm function):   0.40163 a.u.

Sr index (integral of Sr function):   0.70078 a.u.

Centroid of hole in X/Y/Z: 0.000086   0.000023   -0.418301 Angstrom

Centroid of electron in X/Y/Z: -0.000023   0.000046   0.276697 Angstrom

D_x: 0.000 D_y: 0.000 D_z: 0.695   **D index: 0.695 Angstrom**

Variation of dipole moment with respect to ground state:

X: 0.000203 Y: -0.000042 Z: -1.292792   Norm: 1.292792 a.u.

RMSD of hole in X/Y/Z: 4.639   1.310   1.552   Norm: 5.064 Angstrom

RMSD of electron in X/Y/Z: 2.861   1.957   1.372   Norm: 3.728 Angstrom

Difference between RMSD of hole and electron (delta sigma):

X: -1.778 Y: 0.647 Z: -0.180   Overall: -1.336 Angstrom

H_x: 3.750 H_y: 1.634 H_z: 1.462 H_CT: 1.462 H index: 4.396 Angstrom

t index: -0.767 Angstrom

Hole delocalization index (HDI):   5.88

Electron delocalization index (EDI):   5.29

Ghost-hunter index: -17.169 eV, 1st term: 3.550 eV, 2nd term: 20.719 eV

Excitation energy of this state: 2.882 eV

Excitation energies and oscillator strengths:

Excited State   1:   Singlet-A   2.8818 eV 430.23 nm f=0.8420 <S**2>=0.000

169 ->170   0.70155

This state for optimization and/or second-order correction.

Total Energy, E(TD-HF/TD-DFT) = -1957.45485985

Copying the excited state density for this state as the 1-particle RhoCI density.

Excited State   2:   Singlet-A   3.4429 eV 360.12 nm f=0.0221 <S**2>=0.000

168 ->170   0.67612

169 ->171   0.17294

Excited State   3:   Singlet-A   3.5133 eV 352.90 nm f=0.0037 <S**2>=0.000

168 ->170   -0.18021

169 ->171 0.66170

7.15 Quantum chemical calculation data of *N*[4]-([1,1'-biphenyl]-3-yl)-*N*[4],*N*[4'],*N*[4']-triphenyl-[1,1':2',1''-terphenyl]-4,4'-diamine (5b')

7.15.1 Computed xyz-coordinates of compound 5b' (PBE1PBE/6-31+G** PCM CH_2Cl_2)

Inputfile:

%NProcShared=10

%Chk=gauss.chk

%mem=10GB

#p PBE1PBE 6-31+G** pop=full GFInput opt freq SCRF=(Solvent=Dichloromethane)

Optimized S_0-geometry:

0 1

C 0 2.650527 0.637024 -0.431455

C 0 4.035021 0.486585 -0.562308

C 0 4.764131 -0.397566 0.238739

C 0 4.075155 -1.154787 1.193113

C 0 2.697781 -1.032184 1.308970

C 0 1.952760 -0.151162 0.511414

N 0 6.162903 -0.513958 0.093577

C 0 6.779206 -1.782969 0.182050

C 0 6.950516 0.636854 -0.140562

C 0 7.992115 -1.936942 0.867020

C 0 8.601150 -3.186168 0.943986

C 0 8.006372 -4.303309 0.357013

C 0 6.794901 -4.153338 -0.318284

C 0 6.187647 -2.904564 -0.414735

C 0 7.997098 0.602947 -1.071640

C 0 8.776480 1.734830 -1.293042

C 0 8.516896 2.920671 -0.605605

C 0 7.469955 2.958479 0.315692

C 0 6.696762 1.826211 0.555642

C 0 1.985856 1.657239 -1.285474

C 0 1.179379 2.655596 -0.722050

C 0	0.598864	3.636602	-1.521433
C 0	0.809387	3.635451	-2.900836
C 0	1.611495	2.649156	-3.472482
C 0	2.197378	1.671070	-2.670300
C 0	0.478297	-0.124513	0.671666
C 0	-0.385991	-0.251685	-0.425158
C 0	-1.765087	-0.264521	-0.261900
C 0	-2.332365	-0.163324	1.015724
C 0	-1.478985	-0.047068	2.119726
C 0	-0.099883	-0.023717	1.944473
N 0	-3.734194	-0.177552	1.186394
C 0	-4.341037	0.697775	2.116066
C 0	-4.531106	-1.060598	0.425112
C 0	-5.753157	-0.628527	-0.102648
C 0	-6.563673	-1.486898	-0.853351
C 0	-6.122274	-2.796686	-1.091559
C 0	-4.900332	-3.225400	-0.579184
C 0	-4.106796	-2.373495	0.182320
C 0	-5.384665	0.249595	2.937118
C 0	-5.985299	1.116990	3.845071
C 0	-5.547074	2.436317	3.962680
C 0	-4.502422	2.881496	3.152313
C 0	-3.907244	2.025299	2.230087
C 0	-7.857103	-1.009142	-1.397042
C 0	-8.295497	-1.410927	-2.667329
C 0	-9.512186	-0.964599	-3.177493
C 0	-10.315716	-0.105766	-2.428037
C 0	-9.891232	0.302172	-1.163798
C 0	-8.674907	-0.145370	-0.653850
H 0	4.561839	1.101336	-1.286322
H 0	4.616078	-1.846163	1.831871
H 0	2.174658	-1.652186	2.032325
H 0	8.454613	-1.073889	1.336596

H	0	9.541323	-3.287249	1.479289
H	0	8.480863	-5.277735	0.424627
H	0	6.322253	-5.012007	-0.787328
H	0	5.251031	-2.792980	-0.952852
H	0	8.196631	-0.313561	-1.618927
H	0	9.583753	1.691125	-2.019050
H	0	9.122310	3.803985	-0.785768
H	0	7.258926	3.872514	0.864015
H	0	5.890726	1.859227	1.282669
H	0	1.013395	2.665269	0.351579
H	0	-0.017563	4.406528	-1.065374
H	0	0.352797	4.398955	-3.524343
H	0	1.780729	2.637372	-4.545723
H	0	2.814575	0.899474	-3.123735
H	0	0.027037	-0.336341	-1.426258
H	0	-2.410852	-0.358611	-1.129867
H	0	-1.899345	0.031557	3.117915
H	0	0.540861	0.078381	2.816744
H	0	-6.061558	0.400019	0.059340
H	0	-6.745044	-3.485506	-1.654577
H	0	-4.570633	-4.245696	-0.754719
H	0	-3.162125	-2.719426	0.590298
H	0	-5.722204	-0.779716	2.859598
H	0	-6.792588	0.752622	4.474578
H	0	-6.013515	3.108554	4.676662
H	0	-4.154305	3.908118	3.226722
H	0	-3.102837	2.381411	1.593349
H	0	-7.667624	-2.061107	-3.270582
H	0	-9.828802	-1.282040	-4.167228
H	0	-11.264411	0.242943	-2.826045
H	0	-10.512582	0.964901	-0.567751
H	0	-8.365934	0.161680	0.341843

SCF Done: E(RPBE1PBE) = -1958.12549964   A.U. after   16 cycles

Zero-point correction=   0.702288 (Hartree/Particle)

Thermal correction to Energy=   0.742088

Thermal correction to Enthalpy=   0.743032

Thermal correction to Gibbs Free Energy=   0.622514

Sum of electronic and zero-point Energies=   -1957.428385

Sum of electronic and thermal Energies=   -1957.388586

Sum of electronic and thermal Enthalpies=   -1957.387642

Sum of electronic and thermal Free Energies=   -1957.508160

7.15.2 Computed excitations of compound 5b' (PBE1PBE/6-31+G PCM CH_2Cl_2)**

Inputfile:

%NProcShared=10

%Chk=gauss.chk

%mem=10GB

#p PBE1PBE 6-31+G** geom=check guess=read SCRF=(Solvent=Dichloromethan

e) TD=(Nstates=15,root=1)

Excitation energies and oscillator strengths:

Excited State   1:   Singlet-A   3.4150 eV 363.06 nm f=0.7715 <S**2>=0.000

168 -> 171   -0.10054

169 -> 170   0.66141

169 -> 171   0.16673

This state for optimization and/or second-order correction.

Total Energy, E(TD-HF/TD-DFT) = -1958.00517635

Copying the excited state density for this state as the 1-particle RhoCI density.

Excited State   2:   Singlet-A   3.5252 eV 351.71 nm f=0.1602 <S**2>=0.000

168 -> 170   -0.21425

168 -> 171   0.19969

169 -> 170   -0.11326

169 -> 171   0.59896

169 -> 173   0.11215

Excited State   3:   Singlet-A   3.6328 eV 341.29 nm f=0.1602 <S**2>=0.000

168 -> 170   -0.13230

168 -> 172 -0.23384
169 -> 172 0.62180

Excited State 4: Singlet-A 3.8348 eV 323.32 nm f=0.0131 <S**2>=0.000
168 -> 170 0.56412
168 -> 171 0.31082
168 -> 172 -0.15295
169 -> 172 0.10375
169 -> 178 -0.10121

Excited State 5: Singlet-A 3.8709 eV 320.30 nm f=0.1906 <S**2>=0.000
168 -> 172 -0.13959
168 -> 173 0.27694
169 -> 171 -0.13659
169 -> 173 0.58013

Excited State 6: Singlet-A 3.9435 eV 314.40 nm f=0.2485 <S**2>=0.000
168 -> 174 -0.34995
169 -> 174 0.59290

Excited State 7: Singlet-A 4.0116 eV 309.07 nm f=0.0403 <S**2>=0.000
168 -> 171 0.14237
168 -> 172 0.29568
168 -> 173 0.10361
168 -> 175 0.13085
168 -> 176 0.10599
169 -> 172 0.16398
169 -> 175 0.52843

Excited State 8: Singlet-A 4.1011 eV 302.32 nm f=0.0149 <S**2>=0.000
168 -> 170 -0.30152
168 -> 171 0.49166
168 -> 172 -0.14808
168 -> 175 -0.10578
169 -> 170 0.15389
169 -> 171 -0.25781
169 -> 178 -0.11650

Excited State 9: Singlet-A 4.1188 eV 301.02 nm f=0.0540 <S**2>=0.000

168 -> 172 -0.29979

168 -> 173 -0.10456

168 -> 175 0.20124

169 -> 172 -0.14613

169 -> 173 -0.12712

169 -> 175 0.22989

169 -> 176 0.46710

Excited State 10: Singlet-A 4.2085 eV 294.60 nm f=0.0226 <S**2>=0.000

168 -> 171 0.18648

168 -> 172 0.37634

168 -> 175 0.13230

168 -> 176 -0.22070

169 -> 172 0.11561

169 -> 173 0.12532

169 -> 175 -0.20162

169 -> 176 0.31375

169 -> 177 0.14549

169 -> 178 0.16696

Excited State 11: Singlet-A 4.3237 eV 286.76 nm f=0.0205 <S**2>=0.000

168 -> 171 0.14188

168 -> 172 -0.15362

168 -> 181 -0.12425

169 -> 176 -0.23141

169 -> 177 0.32321

169 -> 178 0.42181

169 -> 179 0.15464

169 -> 181 -0.16689

Excited State 12: Singlet-A 4.3878 eV 282.56 nm f=0.0398 <S**2>=0.000

168 -> 172 0.12486

168 -> 173 -0.12217

168 -> 176 0.13582

168 -> 180 -0.13558

169 -> 175   -0.11772

169 -> 177   -0.18535

169 -> 178   -0.10330

169 -> 179   0.53508

169 -> 180   0.11531

169 -> 181   -0.12285

Excited State 13:   Singlet-A   4.4641 eV 277.73 nm f=0.0152 <S**2>=0.000

168 -> 179   -0.13304

168 -> 180   -0.29953

168 -> 181   0.10424

169 -> 180   0.55514

Excited State 14:   Singlet-A   4.4768 eV 276.95 nm f=0.0354 <S**2>=0.000

168 -> 173   0.34873

168 -> 175   -0.15693

168 -> 177   0.19160

168 -> 181   -0.11984

169 -> 173   -0.15028

169 -> 176   0.14938

169 -> 177   0.26536

169 -> 178   -0.21130

169 -> 181   -0.29694

Excited State 15:   Singlet-A   4.4914 eV 276.05 nm f=0.0024 <S**2>=0.000

168 -> 173   -0.27569

168 -> 177   0.16144

168 -> 178   -0.17364

169 -> 176   -0.10486

169 -> 177   0.39399

169 -> 178   -0.37150

169 -> 181   0.13624

7.15.3 Computed emission of compound 5b' (PBE1PBE/6-31+G** PCM CH_2Cl_2)

Inputfile:

%NProcShared=20

%Chk=gauss.chk

```
%mem=20GB
#p PBE1PBE 6-31+G** opt freq
SCRF=(Solvent=Dichloromethane) TD=(Nstates=3,root=1)
--link1--
%NProcShared=20
%Chk=gauss.chk
%mem=20GB
#p PBE1PBE 6-31+G** geom=check guess=read
SCRF=(Solvent=Dichloromethane) TD=(Nstates=3,root=1)
```

Excitation energies and oscillator strengths:

```
 Excited State   1:      Singlet-A      2.8180 eV  439.97 nm  f=1.0833  <S**2>=0.000
     169 ->170        -0.70108
 This state for optimization and/or second-order correction.
 Total Energy, E(TD-HF/TD-DFT) =  -1957.46261422
 Copying the excited state density for this state as the 1-particle RhoCI density.

 Excited State   2:      Singlet-A      3.4380 eV  360.63 nm  f=0.0317  <S**2>=0.000
     168 ->170        -0.67618
     169 ->171         0.14023

 Excited State   3:      Singlet-A      3.5184 eV  352.39 nm  f=0.0593  <S**2>=0.000
     168 ->170         0.16558
     168 ->171         0.15577
     169 ->171         0.61104
     169 ->172         0.21965
```

7.16 Quantum chemical calculation data of *N*[4],*N*[4']-Di([1,1'-biphenyl]-3-yl)-*N*[4],*N*[4']-diphenyl-[1,1'-biphenyl]-4,4'-diamine (5b'')

7.16.1 Computed xyz-coordinates of compound 5b'' (PBE1PBE/6-31+G** PCM CH_2Cl_2)

Inputfile:

%NProcShared=10

%Chk=gauss.chk

%mem=10GB

#p PBE1PBE 6-31+G** pop=full GFInput opt freq SCRF=(Solvent=Dichloromethane)

Optimized S_0-geometry:

0 1

C 0 -6.551109 -8.217089 4.007760

C 0 -5.855203 -9.143962 4.686619

C 0 -6.408639 -9.779335 5.725923

C 0 -7.665685 -9.466189 6.060423

C 0 -8.341941 -8.536765 5.365469

C 0 -11.678336 8.073603 -5.757381

C 0 -11.596909 9.353769 -6.155778

C 0 -11.418836 10.331218 -5.259660

C 0 -11.327638 9.994177 -3.968065

C 0 -11.410642 8.706736 -3.594107

C 0 -11.585695 7.684323 -4.464280

C 0 -7.822386 -7.864258 4.311918

C 0 -7.970696 -6.128920 2.670352

C 0 -8.524383 -6.924791 3.615858

C 0 -9.838001 -6.700947 3.824011

C 0 -10.513227 -5.771342 3.137530

C 0 -9.896423 -5.030055 2.210106

C 0 -6.733307 -6.270688 0.222151

C 0 -5.668442 -6.782593 -0.415694

C 0 -4.728360 -5.972252 -0.915530

C 0 -4.885460 -4.652523 -0.760615

C 0	-5.962547	-4.169160	-0.120813
C 0	-8.584629	-5.176065	1.925011
C 0	-6.942097	-4.943821	0.405184
N 0	-7.976864	-4.464164	1.025018
C 0	-8.690745	-1.440710	-0.753837
C 0	-8.237862	-2.670134	-0.455177
C 0	-8.409055	-3.274753	0.740460
C 0	-9.086833	-2.488481	1.604825
C 0	-9.534499	-1.254973	1.315506
C 0	-9.364621	-0.661830	0.116522
C 0	-10.760879	5.231986	1.019507
C 0	-10.956063	6.123657	2.004348
C 0	-11.865508	7.095145	1.864683
C 0	-12.562981	7.147366	0.724038
C 0	-12.343906	6.243892	-0.244744
C 0	-10.989772	2.573744	0.373893
C 0	-10.537746	1.343936	0.672236
C 0	-9.824022	0.583484	-0.182529
C 0	-9.617739	1.193523	-1.367248
C 0	-10.080374	2.420080	-1.662472
C 0	-11.437378	5.240036	-0.154633
C 0	-10.794078	3.189763	-0.812163
N 0	-11.236555	4.374364	-1.100839
C 0	-12.009638	5.391580	-4.930988
C 0	-12.085838	4.114359	-4.538214
C 0	-11.829531	3.777344	-3.269034
C 0	-11.484547	4.690216	-2.335743
C 0	-11.419104	5.957530	-2.815342
C 0	-11.672754	6.379587	-4.076925
H 0	-5.991886	-7.791769	3.161240
H 0	-4.823076	-9.396032	4.387386
H 0	-5.845974	-10.542086	6.288627
H 0	-8.139860	-9.976852	6.916403

H	0	-9.356776	-8.359525	5.750217
H	0	-11.807674	7.366817	-6.589903
H	0	-11.672067	9.610256	-7.226705
H	0	-11.352831	11.384993	-5.576282
H	0	-11.192165	10.786739	-3.211846
H	0	-11.348621	8.568972	-2.504431
H	0	-6.881386	-6.182113	2.551345
H	0	-10.452424	-7.268840	4.537019
H	0	-11.595505	-5.638200	3.312181
H	0	-10.556948	-4.350505	1.645203
H	0	-7.477407	-7.025925	0.529473
H	0	-5.571223	-7.873660	-0.549379
H	0	-3.848535	-6.380554	-1.439078
H	0	-4.110778	-3.966013	-1.143216
H	0	-5.949665	-3.073975	0.014753
H	0	-8.491253	-1.127234	-1.789560
H	0	-7.773952	-3.205759	-1.302376
H	0	-9.243216	-2.798908	2.653292
H	0	-10.034918	-0.749632	2.155153
H	0	-9.935895	4.524638	1.212005
H	0	-10.351953	6.075438	2.926620
H	0	-12.034576	7.832513	2.666301
H	0	-13.333286	7.926846	0.594281
H	0	-13.023560	6.339991	-1.109254
H	0	-11.622635	3.031084	1.155006
H	0	-10.825829	0.989453	1.673322
H	0	-9.026186	0.730331	-2.171201
H	0	-9.762805	2.805193	-2.647803
H	0	-12.270008	5.554280	-5.986522
H	0	-12.396531	3.336394	-5.257797
H	0	-11.998499	2.713964	-3.028432
H	0	-11.019364	6.700064	-2.113586

SCF Done: E(RPBE1PBE) = -1958.13704833   A.U. after   16 cycles

Zero-point correction=   0.702606 (Hartree/Particle)

Thermal correction to Energy=   0.742277

Thermal correction to Enthalpy=   0.743221

Thermal correction to Gibbs Free Energy=   0.622494

Sum of electronic and zero-point Energies=   -1957.434508

Sum of electronic and thermal Energies=   -1957.394836

Sum of electronic and thermal Enthalpies=   -1957.393892

Sum of electronic and thermal Free Energies=   -1957.514619

7.16.2 Computed excitations of compound 5b'' (PBE1PBE/6-31+G PCM CH_2Cl_2)**

Inputfile:

%NProcShared=10

%Chk=gauss.chk

%mem=10GB

#p PBE1PBE 6-31+G** geom=check guess=read SCRF=(Solvent=Dichloromethane) TD=(Nstates=15,root=1)

Excitation energies and oscillator strengths:

Excited State   1:   Singlet-A   3.3443 eV 370.74 nm f=1.2086 <S**2>=0.000

169 -> 170   0.66939

169 -> 172   -0.16727

This state for optimization and/or second-order correction.

Total Energy, E(TD-HF/TD-DFT) = -1958.01421372

Copying the excited state density for this state as the 1-particle RhoCI density.

Excited State   2:   Singlet-A   3.4833 eV 355.94 nm f=0.0409 <S**2>=0.000

168 -> 170   -0.16788

168 -> 172   -0.22206

169 -> 171   0.62211

169 -> 173   0.12251

Excited State   3:   Singlet-A   3.5127 eV 352.96 nm f=0.1462 <S**2>=0.000

168 -> 171   -0.27966

169 -> 170   0.12125

169 -> 172   0.61191

Excited State 4: Singlet-A 3.8221 eV 324.39 nm f=0.0068 <S**2>=0.000
168 -> 170 0.44090
168 -> 172 -0.16026
168 -> 174 0.18535
169 -> 173 0.47155

Excited State 5: Singlet-A 3.8638 eV 320.88 nm f=0.4039 <S**2>=0.000
168 -> 173 0.29645
169 -> 172 0.11348
169 -> 174 0.61058

Excited State 6: Singlet-A 3.8646 eV 320.82 nm f=0.0200 <S**2>=0.000
168 -> 170 0.42738
168 -> 172 -0.19785
168 -> 174 -0.21448
169 -> 171 0.12214
169 -> 173 -0.40569
169 -> 175 -0.18972

Excited State 7: Singlet-A 4.0365 eV 307.15 nm f=0.0046 <S**2>=0.000
168 -> 170 0.12516
168 -> 172 -0.13118
168 -> 176 0.21629
169 -> 173 -0.11188
169 -> 175 0.59487
169 -> 179 -0.13395

Excited State 8: Singlet-A 4.0950 eV 302.77 nm f=0.0875 <S**2>=0.000
168 -> 171 -0.14582
168 -> 175 0.28729
169 -> 172 -0.16437
169 -> 176 0.57471

Excited State 9: Singlet-A 4.1218 eV 300.80 nm f=0.0204 <S**2>=0.000
168 -> 171 0.60638
169 -> 170 0.12417

169 -> 172 0.21676
169 -> 174 -0.10629
169 -> 176 0.20607

Excited State 10: Singlet-A 4.1242 eV 300.62 nm f=0.0200 <S**2>=0.000
168 -> 170 0.24376
168 -> 172 0.55993
168 -> 176 0.10159
169 -> 171 0.28059
169 -> 179 0.13716

Excited State 11: Singlet-A 4.3362 eV 285.93 nm f=0.0018 <S**2>=0.000
168 -> 172 -0.17397
168 -> 174 -0.11442
168 -> 176 0.12987
168 -> 181 0.19571
169 -> 177 0.13347
169 -> 179 0.45747
169 -> 180 -0.37379

Excited State 12: Singlet-A 4.4222 eV 280.37 nm f=0.0883 <S**2>=0.000
168 -> 179 0.18296
168 -> 180 -0.23173
169 -> 178 0.14645
169 -> 181 0.58868

Excited State 13: Singlet-A 4.4470 eV 278.81 nm f=0.0000 <S**2>=0.000
168 -> 174 0.12937
168 -> 176 -0.15652
168 -> 178 0.11983
169 -> 177 0.40433
169 -> 179 0.28289
169 -> 180 0.39688

Excited State 14: Singlet-A 4.4626 eV 277.83 nm f=0.0070 <S**2>=0.000
168 -> 173 0.10816
168 -> 177 0.23041

169 -> 178 0.62391

169 -> 181 -0.14359

Excited State 15: Singlet-A 4.4691 eV 277.43 nm f=0.0004 <S**2>=0.000

168 -> 178 0.21430

169 -> 177 0.49790

169 -> 179 -0.34778

169 -> 180 -0.23289

7.16.3 Computed emission of compound 5b'' (PBE1PBE/6-31+G** PCM CH_2Cl_2)

Inputfile:

%NProcShared=10

%Chk=gauss.chk

%mem=10GB

#p PBE1PBE 6-31+G** geom=check guess=read opt freq
SCRF=(Solvent=Dichloromethane) TD=(Nstates=6,root=1)

Excitation energies and oscillator strengths:

Excited State 1: Singlet-A 2.8493 eV 435.13 nm f=1.5063 <S**2>=0.000

169 -> 170 0.69887

This state for optimization and/or second-order correction.

Total Energy, E(TD-HF/TD-DFT) = -1958.02346771

Copying the excited state density for this state as the 1-particle RhoCI density.

Excited State 2: Singlet-A 3.2864 eV 377.27 nm f=0.0276 <S**2>=0.000

168 -> 172 -0.17262

169 -> 171 0.65421

169 -> 173 -0.13192

Excited State 3: Singlet-A 3.3679 eV 368.14 nm f=0.0377 <S**2>=0.000

168 -> 171 -0.22166

169 -> 172 0.65346

Excited State 4: Singlet-A 3.5221 eV 352.01 nm f=0.0000 <S**2>=0.000

168 -> 170 0.68825

Excited State 5: Singlet-A 3.6747 eV 337.40 nm f=0.0819 <S**2>=0.000

168 -> 174 0.19108

169 -> 171 0.13614

169 -> 173 0.65167

Excited State 6: Singlet-A 3.6939 eV 335.65 nm f=0.2760 <S**2>=0.000

168 -> 173 0.20468

169 -> 172 -0.10168

169 -> 174 0.65450

7.17 Quantum chemical calculation data of 3',3'''-([1,1'-Biphenyl]-4,4'-diylbis(phenylazanediyl))bis(([1,1'-biphenyl]-4-carbonitrile)) (5c)

7.17.1 Computed xyz-coordinates of compound 5c (PBE1PBE/6-31+G** PCM CH_2Cl_2)

Inputfile:

%NProcShared=10

%Chk=gauss.chk

%mem=10GB

#p PBE1PBE 6-31+G**

pop=full GFInput opt freq SCRF=(Solvent=Dichloromethane)

Optimized S_0-geometry:

0 1

C 0 9.927585 -0.113603 -0.527959

C 0 11.138872 0.010154 -1.192839

C 0 11.729462 1.274151 -1.330322

C 0 11.094972 2.405207 -0.797744

C 0 9.883151 2.265885 -0.137046

C 0 -9.883151 -2.265886 -0.137047

C 0 -11.094971 -2.405207 -0.797744

C 0 -11.729462 -1.274151 -1.330322

C 0 -11.138871 -0.010154 -1.192839

C 0 -9.927584 0.113603 -0.527959

C 0 -9.278132 -1.008113 0.010466

C 0 9.278133 1.008113 0.010466

C 0 7.030296 -0.045219 0.264149

C 0 7.986439 0.868653 0.719323

C 0 7.697223 1.656037 1.842951

C 0 6.474255 1.513459 2.493100

C 0 5.531940 0.592674 2.047570

C 0 6.264567 -3.113857 0.652391

C 0 6.687592 -4.348970 0.169434

C 0 6.128694 -4.885586 -0.990804

C	0	5.144010	-4.167029	-1.669089
C	0	4.727328	-2.923488	-1.202089
C	0	5.803697	-0.195906	0.921189
C	0	5.282719	-2.386976	-0.033487
N	0	4.855478	-1.127067	0.449656
C	0	1.171810	-1.446859	0.804784
C	0	2.524190	-1.762849	0.812992
C	0	3.480944	-0.805575	0.451268
C	0	3.042146	0.474290	0.087554
C	0	1.688412	0.784748	0.095701
C	0	0.719302	-0.166453	0.450485
C	0	-4.727331	2.923488	-1.202089
C	0	-5.144013	4.167029	-1.669089
C	0	-6.128697	4.885585	-0.990803
C	0	-6.687593	4.348969	0.169437
C	0	-6.264567	3.113856	0.652393
C	0	-2.524190	1.762850	0.812991
C	0	-1.171810	1.446859	0.804783
C	0	-0.719302	0.166453	0.450485
C	0	-1.688412	-0.784748	0.095702
C	0	-3.042145	-0.474290	0.087555
C	0	-5.282720	2.386975	-0.033486
C	0	-3.480943	0.805575	0.451268
N	0	-4.855478	1.127067	0.449656
C	0	-7.697223	-1.656038	1.842951
C	0	-6.474254	-1.513460	2.493099
C	0	-5.531939	-0.592675	2.047570
C	0	-5.803696	0.195905	0.921188
C	0	-7.030295	0.045219	0.264148
C	0	-7.986438	-0.868654	0.719323
C	0	-12.977667	-1.409801	-2.013710
N	0	-13.993235	-1.520368	-2.569539
C	0	12.977668	1.409800	-2.013710

N	0	13.993235	1.520377	-2.569537
H	0	9.490154	-1.099912	-0.406037
H	0	11.634071	-0.866426	-1.597702
H	0	11.547711	3.384770	-0.911734
H	0	9.387574	3.150713	0.250415
H	0	-9.387574	-3.150713	0.250415
H	0	-11.547710	-3.384770	-0.911734
H	0	-11.634070	0.866426	-1.597702
H	0	-9.490153	1.099912	-0.406037
H	0	7.224893	-0.633233	-0.627643
H	0	8.435333	2.354975	2.224568
H	0	6.259631	2.111562	3.374138
H	0	4.586741	0.479190	2.569110
H	0	6.693052	-2.707217	1.563769
H	0	7.449285	-4.900867	0.713381
H	0	6.456053	-5.852382	-1.361402
H	0	4.703988	-4.569111	-2.577507
H	0	3.969150	-2.362581	-1.740540
H	0	0.456679	-2.202857	1.117539
H	0	2.845636	-2.756303	1.111230
H	0	3.766692	1.225099	-0.213451
H	0	1.378316	1.777702	-0.218579
H	0	-3.969153	2.362581	-1.740542
H	0	-4.703993	4.569111	-2.577507
H	0	-6.456056	5.852381	-1.361400
H	0	-7.449286	4.900866	0.713385
H	0	-6.693050	2.707216	1.563772
H	0	-2.845636	2.756304	1.111228
H	0	-0.456678	2.202858	1.117537
H	0	-1.378316	-1.777703	-0.218577
H	0	-3.766691	-1.225100	-0.213448
H	0	-8.435332	-2.354976	2.224568
H	0	-6.259631	-2.111563	3.374137

H 0 -4.586740 -0.479191 2.569110

H 0 -7.224893 0.633233 -0.627643

SCF Done: E(RPBE1PBE) = -2142.42716280 A.U. after 16 cycles

Zero-point correction= 0.699902 (Hartree/Particle)

Thermal correction to Energy= 0.743231

Thermal correction to Enthalpy= 0.744175

Thermal correction to Gibbs Free Energy= 0.614677

Sum of electronic and zero-point Energies= -2141.727580

Sum of electronic and thermal Energies= -2141.684251

Sum of electronic and thermal Enthalpies= -2141.683306

Sum of electronic and thermal Free Energies= -2141.812804

7.17.2 Computed excitations of compound 5c (PBE1PBE/6-31+G** PCM CH_2Cl_2)

Inputfile:

%NProcShared=10

%Chk=gauss.chk

%mem=10GB

#p PBE1PBE 6-31+G** geom=check guess=read

SCRF=(Solvent=Dichloromethane) TD=(Nstates=15,root=1)

Excitation analysis by Multiwfn:

Integral of hole: 0.977486

Integral of electron: 0.977367

Integral of transition density: -0.000004

Transition dipole moment in X/Y/Z: 0.142406 -0.333250 0.000014 a.u.

Sm index (integral of Sm function): 0.15617 a.u.

Sr index (integral of Sr function): **0.30624 a.u.**

Centroid of hole in X/Y/Z: 0.000113 -0.000066 0.451111 Angstrom

Centroid of electron in X/Y/Z: -0.000550 -0.000076 -0.456035 Angstrom

D_x: 0.001 D_y: 0.000 D_z: 0.907 D index: **0.907 Angstrom**

Variation of dipole moment with respect to ground state:

X: 0.001224 Y: 0.000019 Z: 1.675560 Norm: 1.675560 a.u.

RMSD of hole in X/Y/Z: 4.960 1.996 1.031 Norm: 5.445 Angstrom

RMSD of electron in X/Y/Z: 10.359 1.545 1.541 Norm: 10.586 Angstrom

Difference between RMSD of hole and electron (delta sigma):

X: 5.399 Y: -0.452 Z: 0.510 Overall: 5.141 Angstrom

H_x: 7.659 H_y: 1.770 H_z: 1.286 H_CT: 1.286 H index: 8.016 Angstrom

t index: -0.379 Angstrom

Hole delocalization index (HDI): 6.02

Electron delocalization index (EDI): 4.79

Ghost-hunter index: -12.324 eV, 1st term: 3.549 eV, 2nd term: 15.874 eV

Excitation energy of this state: 2.986 eV

Excitation energies and oscillator strengths:

Excited State 1: Singlet-A 2.9860 eV 415.22 nm f=0.0057 <S**2>=0.000

180 -> 183 0.24364

181 -> 182 0.65526

This state for optimization and/or second-order correction.

Total Energy, E(TD-HF/TD-DFT) = -2142.31774945

Copying the excited state density for this state as the 1-particle RhoCI density.

Excited State 2: Singlet-A 2.9904 eV 414.61 nm f=0.0245 <S**2>=0.000

180 -> 182 0.24709

181 -> 183 0.65474

Excited State 3: Singlet-A 3.3849 eV 366.29 nm f=1.4961 <S**2>=0.000

180 -> 191 0.10316

181 -> 184 0.68767

Excited State 4: Singlet-A 3.5311 eV 351.12 nm f=0.0089 <S**2>=0.000

180 -> 182 0.64961

181 -> 183 -0.25802

Excited State 5: Singlet-A 3.5360 eV 350.63 nm f=0.0006 <S**2>=0.000

180 -> 183 0.64647

181 -> 182 -0.25754

Excited State 6: Singlet-A 3.7624 eV 329.53 nm f=0.0000 <S**2>=0.000

180 -> 184 -0.16335

180 -> 186 -0.18480

180 -> 188 -0.16639

181 -> 185 0.57182

181 -> 187 -0.25353

Excited State 7: Singlet-A 3.8235 eV 324.27 nm f=0.2564 <S**2>=0.000
180 -> 185 -0.29219
181 -> 186 0.49522
181 -> 188 0.36683

Excited State 8: Singlet-A 3.8912 eV 318.63 nm f=0.0101 <S**2>=0.000
180 -> 184 0.64085
181 -> 185 0.15290
181 -> 189 -0.14894
181 -> 191 0.12418

Excited State 9: Singlet-A 3.9225 eV 316.09 nm f=0.0269 <S**2>=0.000
180 -> 184 0.17390
180 -> 186 0.12313
180 -> 190 -0.25158
181 -> 187 -0.28769
181 -> 189 0.52874

Excited State 10: Singlet-A 3.9332 eV 315.22 nm f=0.2286 <S**2>=0.000
180 -> 187 0.14111
180 -> 189 -0.26241
181 -> 186 -0.13138
181 -> 188 0.19764
181 -> 190 0.57783

Excited State 11: Singlet-A 4.1257 eV 300.52 nm f=0.0013 <S**2>=0.000
180 -> 186 -0.12868
180 -> 188 0.17829
180 -> 190 -0.11449
181 -> 185 0.24308
181 -> 187 0.53266
181 -> 189 0.30136

Excited State 12: Singlet-A 4.1266 eV 300.45 nm f=0.0413 <S**2>=0.000
180 -> 187 0.20536
180 -> 189 0.12473

181 -> 186 -0.38649
181 -> 188 0.47582
181 -> 190 -0.24061

Excited State 13: Singlet-A 4.3179 eV 287.14 nm f=0.0001 <S**2>=0.000
180 -> 186 0.17422
180 -> 188 0.22509
180 -> 190 0.14546
180 -> 193 -0.12756
181 -> 191 0.36793
181 -> 192 0.42612

Excited State 14: Singlet-A 4.3991 eV 281.84 nm f=0.1475 <S**2>=0.000
178 -> 183 0.16516
179 -> 182 -0.15767
180 -> 185 0.50520
180 -> 187 -0.23453
181 -> 186 0.19346
181 -> 188 0.19241
181 -> 193 0.12508
181 -> 195 0.12310

Excited State 15: Singlet-A 4.4100 eV 281.15 nm f=1.1382 <S**2>=0.000
178 -> 183 0.41234
179 -> 182 -0.40951
180 -> 185 -0.22591
180 -> 191 -0.10895
180 -> 192 -0.10803
181 -> 188 -0.11022
181 -> 193 0.10569
181 -> 195 0.16939

7.17.3 Computed emission of compound 5c (PBE1PBE/6-31+G** PCM CH_2Cl_2)

Inputfile:

%NProcShared=10

%Chk=gauss.chk

%mem=10GB

#p PBE1PBE 6-31+G** opt freq

SCRF=(Solvent=Dichloromethane) TD=(Nstates=6,root=1)

--link1--

%NProcShared=10

%Chk=gauss.chk

%mem=10GB

#p PBE1PBE 6-31+G** geom=check guess=read

SCRF=(Solvent=Dichloromethane) TD=(Nstates=6,root=1)

Excitation analysis by Multiwfn:

Integral of hole: 0.991537

Integral of electron: 0.991526

Integral of transition density: -0.000020

Transition dipole moment in X/Y/Z: 0.242552 -0.078469 -0.569752 a.u.

Sm index (integral of Sm function): 0.07796 a.u.

Sr index (integral of Sr function): 0.21671 a.u.

Centroid of hole in X/Y/Z: 1.496276 -0.651810 0.723717 Angstrom

Centroid of electron in X/Y/Z: 9.213128 1.296222 -0.724628 Angstrom

D_x: 7.717 D_y: 1.948 D_z: 1.448 **D index: 8.090 Angstrom**

Variation of dipole moment with respect to ground state:

X: -14.459241 Y: -3.650072 Z: 2.713797 Norm: 15.157750 a.u.

RMSD of hole in X/Y/Z: 4.016 1.802 0.892 Norm: 4.492 Angstrom

RMSD of electron in X/Y/Z: 2.286 1.243 2.024 Norm: 3.297 Angstrom

Difference between RMSD of hole and electron (delta sigma):

X: -1.730 Y: -0.559 Z: 1.132 Overall: -1.195 Angstrom

H_x: 3.151 H_y: 1.523 H_z: 1.458 H_CT: 3.039 H index: 3.894 Angstrom

t index: 5.050 Angstrom

Hole delocalization index (HDI): 6.58

Electron delocalization index (EDI): 6.70

Ghost-hunter index: 0.966 eV, 1st term: 2.746 eV, 2nd term: 1.780 eV

Excitation energy of this state: 2.229 eV

Excitation energies and oscillator strengths:

Excited State 1: Singlet-A 2.2260 eV 556.98 nm f=0.0103 <S**2>=0.000

180 -> 182 -0.13149

181 -> 182 0.69178

This state for optimization and/or second-order correction.

Total Energy, E(TD-HF/TD-DFT) = -2142.33160589

Copying the excited state density for this state as the 1-particle RhoCI density.

Excited State 2: Singlet-A 2.7161 eV 456.49 nm f=0.0132 <S**2>=0.000

180 -> 183 -0.13666

181 -> 183 -0.68791

Excited State 3: Singlet-A 3.0965 eV 400.40 nm f=1.5192 <S**2>=0.000

180 -> 182 -0.31232

181 -> 184 0.61755

Excited State 4: Singlet-A 3.0985 eV 400.15 nm f=0.3432 <S**2>=0.000

180 -> 182 -0.61156

181 -> 182 -0.12358

181 -> 184 -0.31330

Excited State 5: Singlet-A 3.4891 eV 355.35 nm f=0.1248 <S**2>=0.000

180 -> 185 0.14838

181 -> 185 -0.64159

181 -> 190 0.17149

Excited State 6: Singlet-A 3.5384 eV 350.40 nm f=0.0327 <S**2>=0.000

180 -> 183 -0.55680

181 -> 183 0.14639

181 -> 186 0.14880

181 -> 187 -0.33433

7.18 Quantum chemical calculation data of *N*[4],*N*[4]'-Bis(4'-methoxy-[1,1'-biphenyl]-2-yl)-*N*[4],*N*[4]'-diphenyl-[1,1'-biphenyl]-4,4'-diamine (6a)

7.18.1 Computed xyz-coordinates of compound 6a (PBE1PBE/6-31+G** PCM CH_2Cl_2)

Inputfile:

%NProcShared=10

%Chk=gauss.chk

%mem=10GB

#p PBE1PBE 6-31+G**

pop=full GFInput opt freq SCRF=(Solvent=Dichloromethane)

Optimized S_0-geometry:

0 1

C 0 7.507043 0.711274 -1.017180

C 0 6.747358 -0.404354 -1.346319

C 0 5.733169 -0.852658 -0.490305

C 0 5.502477 -0.148298 0.698506

C 0 6.259556 0.973085 1.012975

C 0 7.280889 1.430574 0.166284

N 0 4.960951 -1.988698 -0.817409

C 0 3.570167 -1.987439 -0.581152

C 0 5.581405 -3.123264 -1.388187

C 0 2.924862 -3.129855 -0.088875

C 0 1.554456 -3.125021 0.136585

C 0 0.773980 -1.983679 -0.105298

C 0 1.433575 -0.844347 -0.592177

C 0 2.801306 -0.843174 -0.832806

C 0 4.963388 -3.820318 -2.435223

C 0 5.575364 -4.940767 -2.989750

C 0 6.816660 -5.375165 -2.524096

C 0 7.436969 -4.677421 -1.487431

C 0 6.824560 -3.565681 -0.916087

C 0 8.085367 2.622658 0.506743

C	0	9.450913	2.699231	0.181010
C	0	10.208494	3.816509	0.495955
C	0	9.621582	4.904002	1.154889
C	0	8.266464	4.851155	1.491171
C	0	7.518742	3.720174	1.164250
C	0	-0.681687	-1.980928	0.141621
C	0	-1.344064	-0.826840	0.588420
C	0	-2.712140	-0.820060	0.827029
C	0	-3.478579	-1.973643	0.613391
C	0	-2.830385	-3.131159	0.161904
C	0	-1.459642	-3.131486	-0.061548
N	0	-4.869905	-1.969231	0.846471
C	0	-5.489907	-3.086057	1.451828
C	0	-5.643867	-0.847107	0.478316
C	0	-6.728961	-3.548363	0.988148
C	0	-7.340821	-4.642490	1.593119
C	0	-6.723906	-5.302793	2.656001
C	0	-5.486566	-4.848786	3.113429
C	0	-4.875237	-3.745469	2.524877
C	0	-5.409070	-0.180757	-0.731381
C	0	-6.167788	0.927618	-1.085865
C	0	-7.195645	1.408258	-0.260217
C	0	-7.424829	0.727716	0.945392
C	0	-6.663615	-0.374201	1.314415
C	0	-8.003811	2.584305	-0.644844
C	0	-7.433482	3.666010	-1.338627
C	0	-8.187021	4.771584	-1.700966
C	0	-9.548157	4.834363	-1.377460
C	0	-10.138218	3.771920	-0.688039
C	0	-9.365484	2.666379	-0.333392
O	0	10.440491	5.952828	1.421971
C	0	9.890571	7.073089	2.092820
O	0	-10.203951	5.955613	-1.770821

C	0	-11.580144	6.068369	-1.453314
H	0	8.267884	1.050020	-1.715337
H	0	6.932607	-0.928642	-2.279130
H	0	4.731893	-0.490060	1.383171
H	0	6.075244	1.481178	1.955802
H	0	3.504831	-4.022074	0.127469
H	0	1.089687	-4.018089	0.545599
H	0	0.861712	0.049798	-0.825206
H	0	3.279474	0.047453	-1.229468
H	0	4.003180	-3.480002	-2.811323
H	0	5.082469	-5.468714	-3.801555
H	0	7.294382	-6.245825	-2.963375
H	0	8.400595	-5.006977	-1.108596
H	0	7.307332	-3.034202	-0.101389
H	0	9.935539	1.858502	-0.307995
H	0	11.265008	3.861469	0.248618
H	0	7.779939	5.680115	1.992711
H	0	6.460396	3.710919	1.410936
H	0	-0.774383	0.076126	0.790975
H	0	-3.192535	0.082822	1.192023
H	0	-3.408342	-4.031391	-0.024557
H	0	-0.992638	-4.037187	-0.439036
H	0	-7.208852	-3.046217	0.153385
H	0	-8.301176	-4.988067	1.220313
H	0	-7.201091	-6.159931	3.121663
H	0	-4.996312	-5.347621	3.944997
H	0	-3.918044	-3.389548	2.894173
H	0	-4.633474	-0.541505	-1.400441
H	0	-5.979030	1.405834	-2.043319
H	0	-8.189647	1.087498	1.628520
H	0	-6.851437	-0.867933	2.263255
H	0	-6.373766	3.652768	-1.578808
H	0	-7.734282	5.606527	-2.227561

H 0 -11.191787   3.784711   -0.432495

H 0   -9.850009   1.839829   0.179512

H 0   10.708301   7.784566   2.208578

H 0   9.088391   7.534739   1.505235

H 0   9.507519   6.796131   3.081931

H 0 -11.900974   7.032522   -1.847999

H 0 -12.164615   5.270437   -1.925993

H 0 -11.741370   6.047840   -0.369161

SCF Done: E(RPBE1PBE) = -2186.93892925   A.U. after   16 cycles
Zero-point correction=   0.768174 (Hartree/Particle)
Thermal correction to Energy=   0.812893
Thermal correction to Enthalpy=   0.813838
Thermal correction to Gibbs Free Energy=   0.684014
Sum of electronic and zero-point Energies=   -2186.172433
Sum of electronic and thermal Energies=   -2186.127714
Sum of electronic and thermal Enthalpies=   -2186.126769
Sum of electronic and thermal Free Energies=   -2186.256592

7.18.2 Computed excitations of compound 6a (PBE1PBE/6-31+G PCM CH_2Cl_2)**

Inputfile:
%NProcShared=10
%Chk=gauss.chk
%mem=10GB
#p PBE1PBE 6-31+G** geom=check guess=read SCRF=(Solvent=Dichloromethane)
TD=(Nstates=15,root=1)

Excitation analysis by Multiwfn:
Integral of hole: 0.971272
Integral of electron: 0.971192
Integral of transition density: -0.000009
Transition dipole moment in X/Y/Z: 3.916071 -0.000162 -0.060745 a.u.
Sm index (integral of Sm function):   0.41580 a.u.
Sr index (integral of Sr function):   **0.68421 a.u.**
Centroid of hole in X/Y/Z: 0.000146   -1.057140   0.000003 Angstrom

Centroid of electron in X/Y/Z: -0.000242 -0.847904 -0.000076 Angstrom

D_x: 0.000 D_y: 0.209 D_z: 0.000 D index: **0.209 Angstrom**

Variation of dipole moment with respect to ground state:

X: 0.000712 Y: -0.384025 Z: 0.000145 Norm: 0.384025 a.u.

RMSD of hole in X/Y/Z: 4.826 1.001 1.610 Norm: 5.185 Angstrom

RMSD of electron in X/Y/Z: 4.910 1.552 1.724 Norm: 5.430 Angstrom

Difference between RMSD of hole and electron (delta sigma):

X: 0.084 Y: 0.550 Z: 0.114 Overall: 0.245 Angstrom

H_x: 4.868 H_y: 1.276 H_z: 1.667 H_CT: 1.277 H index: 5.307 Angstrom

t index: -1.067 Angstrom

Hole delocalization index (HDI): 6.06

Electron delocalization index (EDI): 4.14

Ghost-hunter index: -64.699 eV, 1st term: 4.120 eV, 2nd term: 68.820 eV

Excitation energy of this state: 3.352 eV

Excitation energies and oscillator strengths:

Excited State 1: Singlet-A 3.3515 eV 369.94 nm f=1.2404 <S**2>=0.000

184 -> 187 -0.12404

185 -> 186 0.68574

This state for optimization and/or second-order correction.

Total Energy, E(TD-HF/TD-DFT) = -2186.81744162

Copying the excited state density for this state as the 1-particle RhoCI density.

Excited State 2: Singlet-A 3.5861 eV 345.74 nm f=0.0394 <S**2>=0.000

184 -> 186 -0.18391

184 -> 188 -0.19523

185 -> 187 0.64266

Excited State 3: Singlet-A 3.6838 eV 336.57 nm f=0.1460 <S**2>=0.000

184 -> 187 -0.23531

185 -> 188 0.64219

Excited State 4: Singlet-A 3.7799 eV 328.01 nm f=0.0019 <S**2>=0.000

184 -> 186 0.14645

184 -> 190 -0.25727

185 -> 189 0.62888

```
Excited State   5:      Singlet-A      3.8027 eV  326.04 nm  f=0.2480  <S**2>=0.000
    184 -> 189       -0.25454
    185 -> 190        0.63893

Excited State   6:      Singlet-A      3.9894 eV  310.78 nm  f=0.0085  <S**2>=0.000
    184 -> 186        0.58543
    184 -> 188       -0.11954
    184 -> 190        0.10933
    185 -> 187        0.10717
    185 -> 189       -0.11892
    185 -> 191       -0.27833
    185 -> 195       -0.11495

Excited State   7:      Singlet-A      4.1045 eV  302.07 nm  f=0.0013  <S**2>=0.000
    184 -> 186        0.21189
    184 -> 192        0.15546
    184 -> 194       -0.10605
    185 -> 187        0.11103
    185 -> 191        0.49949
    185 -> 193        0.34078
    185 -> 195       -0.11906

Excited State   8:      Singlet-A      4.1769 eV  296.83 nm  f=0.0083  <S**2>=0.000
    184 -> 191        0.22232
    184 -> 195       -0.13556
    185 -> 192        0.60684
    185 -> 194       -0.20086

Excited State   9:      Singlet-A      4.1855 eV  296.22 nm  f=0.0010  <S**2>=0.000
    184 -> 186       -0.13046
    184 -> 188       -0.22331
    184 -> 192       -0.14320
    185 -> 187       -0.10604
    185 -> 191       -0.30994
    185 -> 193        0.51888
    185 -> 196        0.10150
```

Excited State 10: Singlet-A 4.2963 eV 288.59 nm f=0.0762 <S**2>=0.000
184 -> 187 -0.31618
184 -> 193 -0.16191
185 -> 188 -0.10667
185 -> 192 0.15682
185 -> 194 0.51606
185 -> 197 0.18345

Excited State 11: Singlet-A 4.3090 eV 287.73 nm f=0.0102 <S**2>=0.000
184 -> 187 0.53982
184 -> 193 -0.19101
185 -> 186 0.10659
185 -> 188 0.22515
185 -> 192 0.13555
185 -> 194 0.27623

Excited State 12: Singlet-A 4.3297 eV 286.36 nm f=0.0065 <S**2>=0.000
184 -> 186 0.16639
184 -> 188 0.27770
184 -> 192 -0.14574
185 -> 187 0.14266
185 -> 193 0.15694
185 -> 195 0.53667

Excited State 13: Singlet-A 4.4178 eV 280.65 nm f=0.0034 <S**2>=0.000
184 -> 188 0.47483
184 -> 194 -0.16334
185 -> 187 0.14012
185 -> 191 -0.13221
185 -> 193 0.17687
185 -> 195 -0.25313
185 -> 196 -0.27046

Excited State 14: Singlet-A 4.4674 eV 277.53 nm f=0.0236 <S**2>=0.000
184 -> 196 -0.24577
185 -> 194 -0.16212
185 -> 197 0.59545

Excited State 15:   Singlet-A   4.4933 eV 275.93 nm f=0.0007 <S**2>=0.000

184 -> 188   0.22213

184 -> 197   -0.20838

185 -> 195   -0.22573

185 -> 196   0.55649

7.18.3 Computed emission of compound 6a (PBE1PBE/6-31+G PCM CH_2Cl_2)**

Inputfile:

%NProcShared=10

%Chk=gauss.chk

%mem=10GB

#p PBE1PBE 6-31+G** opt freq

SCRF=(Solvent=Dichloromethane) TD=(Nstates=6,root=1)

--link1--

%NProcShared=10

%Chk=gauss.chk

%mem=10GB

#p PBE1PBE 6-31+G** geom=check guess=read

SCRF=(Solvent=Dichloromethane) TD=(Nstates=6,root=1)

Excitation analysis by Multiwfn:

Integral of hole: 0.974263

Integral of electron: 0.974130

Integral of transition density: -0.000008

Transition dipole moment in X/Y/Z: -4.889365 -0.000019   0.533212 a.u.

Sm index (integral of Sm function):   0.45739 a.u.

Sr index (integral of Sr function):   0.72850 a.u.

Centroid of hole in X/Y/Z: -0.000041   -0.895477   -0.000012 Angstrom

Centroid of electron in X/Y/Z: -0.000048   -0.920410   -0.000000 Angstrom

D_x: 0.000 D_y: 0.025 D_z: 0.000   **D index: 0.025 Angstrom**

Variation of dipole moment with respect to ground state:

X: 0.000014 Y: 0.045902 Z: -0.000021   Norm: 0.045902 a.u.

RMSD of hole in X/Y/Z: 4.632   1.182   1.561   Norm: 5.029 Angstrom

RMSD of electron in X/Y/Z: 3.980   1.283   1.470   Norm: 4.433 Angstrom

Difference between RMSD of hole and electron (delta sigma):

X: -0.652 Y: 0.101 Z: -0.091   Overall: -0.596 Angstrom

H_x: 4.306 H_y: 1.232 H_z: 1.515 H_CT: 1.232 H index: 4.731 Angstrom

t index: -1.207 Angstrom

Hole delocalization index (HDI):   5.77

Electron delocalization index (EDI):   5.28

Ghost-hunter index: -574.011 eV, 1st term: 3.509 eV, 2nd term: 577.519 eV

Excitation energy of this state: 2.846 eV

Excitation energies and oscillator strengths:

Excited State   1:   Singlet-A   2.7786 eV 446.21 nm f=1.6591 <S**2>=0.000

185 -> 186   0.69868

This state for optimization and/or second-order correction.

Total Energy, E(TD-HF/TD-DFT) = -2186.82958983

Copying the excited state density for this state as the 1-particle RhoCI density.

Excited State   2:   Singlet-A   3.3381 eV 371.43 nm f=0.0227 <S**2>=0.000

184 -> 186   0.14866

184 -> 188   -0.15730

185 -> 187   0.65904

Excited State   3:   Singlet-A   3.4747 eV 356.82 nm f=0.1227 <S**2>=0.000

184 -> 187   0.21898

185 -> 188   -0.66284

Excited State   4:   Singlet-A   3.5444 eV 349.80 nm f=0.0031 <S**2>=0.000

184 -> 186   -0.52228

184 -> 190   -0.11436

185 -> 189   0.43374

Excited State   5:   Singlet-A   3.6101 eV 343.44 nm f=0.0585 <S**2>=0.000

184 -> 186 -0.42619

184 -> 188 -0.10492

184 -> 190 0.14084

185 -> 187 0.12961

185 -> 189 -0.50634

Excited State 6: Singlet-A 3.6416 eV 340.46 nm f=0.1613 <S**2>=0.000

184 -> 189 0.22166

185 -> 190 -0.66152

7.19 Quantum chemical calculation data of *N*[4],*N*[4']-Di([1,1'-biphenyl]-2-yl)-*N*[4],*N*[4']-diphenyl-[1,1'-biphenyl]-4,4'-diamine (6b)

7.19.1 Computed xyz-coordinates of compound 6b (PBE1PBE/6-31+G** PCM CH_2Cl_2)

Inputfile:

%NProcShared=10

%Chk=gauss.chk

%mem=10GB

#p PBE1PBE 6-31+G**

pop=full GFInput opt freq SCRF=(Solvent=Dichloromethane)

Optimized S_0-geometry:

0 1

C 0 -7.539989 1.302246 0.641982

C 0 -6.782261 0.210391 1.045378

C 0 -5.709128 -0.239232 0.264034

C 0 -5.417065 0.442413 -0.925263

C 0 -6.171601 1.542378 -1.311990

C 0 -7.252451 1.997407 -0.542124

N 0 -4.940702 -1.351631 0.664537

C 0 -3.541800 -1.350589 0.475448

C 0 -5.569015 -2.472131 1.256112

C 0 -2.878868 -2.501893 0.030729

C 0 -1.501453 -2.498914 -0.147146

C 0 -0.731760 -1.351026 0.098177

C 0 -1.409167 -0.203007 0.538170

C 0 -2.784549 -0.199720 0.730424

C 0 -4.985814 -3.111855 2.357881

C 0 -5.601474 -4.220432 2.931962

C 0 -6.812101 -4.698715 2.430117

C 0 -7.397701 -4.058004 1.337908

C 0 -6.780601 -2.958780 0.747615

C 0 -8.057846 3.164388 -0.962594

C 0 -9.441883 3.206405 -0.733313

C 0 -10.200175 4.306144 -1.127075

C 0 -9.592467 5.388180 -1.763524

C 0 -8.218249 5.359509 -1.999907

C	0	-7.459825	4.261286	-1.602173
C	0	0.731741	-1.351025	-0.098179
C	0	1.409146	-0.203005	-0.538171
C	0	2.784529	-0.199717	-0.730424
C	0	3.541782	-1.350584	-0.475449
C	0	2.878851	-2.501890	-0.030731
C	0	1.501435	-2.498912	0.147143
N	0	4.940684	-1.351625	-0.664537
C	0	5.569001	-2.472121	-1.256116
C	0	5.709115	-0.239231	-0.264031
C	0	6.780589	-2.958764	-0.747619
C	0	7.397697	-4.057982	-1.337915
C	0	6.812103	-4.698692	-2.430129
C	0	5.601474	-4.220413	-2.931975
C	0	4.985807	-3.111842	-2.357891
C	0	5.417059	0.442414	0.925267
C	0	6.171605	1.542372	1.311995
C	0	7.252459	1.997393	0.542129
C	0	7.539990	1.302230	-0.641977
C	0	6.782252	0.210382	-1.045374
C	0	8.057864	3.164367	0.962600
C	0	7.459856	4.261263	1.602194
C	0	8.218291	5.359478	1.999928
C	0	9.592506	5.388145	1.763530
C	0	10.200201	4.306111	1.127065
C	0	9.441899	3.206379	0.733303
H	0	-8.349173	1.642726	1.282458
H	0	-7.015509	-0.294225	1.978105
H	0	-4.600147	0.099867	-1.553220
H	0	-5.936885	2.033197	-2.252693
H	0	-3.450183	-3.399582	-0.186137
H	0	-1.021555	-3.399271	-0.521042
H	0	-0.847196	0.696952	0.772685
H	0	-3.277873	0.697692	1.091760
H	0	-4.049562	-2.736771	2.760464
H	0	-5.136178	-4.704389	3.786312
H	0	-7.293062	-5.559950	2.884194

H	0	-8.336560	-4.423193	0.930711
H	0	-7.234918	-2.472219	-0.110380
H	0	-9.933547	2.359269	-0.262569
H	0	-11.271393	4.312631	-0.944960
H	0	-10.184011	6.245358	-2.072229
H	0	-7.731545	6.200028	-2.487114
H	0	-6.386013	4.264893	-1.769167
H	0	0.847175	0.696953	-0.772685
H	0	3.277851	0.697696	-1.091759
H	0	3.450166	-3.399578	0.186134
H	0	1.021538	-3.399271	0.521039
H	0	7.234901	-2.472203	0.110378
H	0	8.336557	-4.423167	-0.930718
H	0	7.293070	-5.559922	-2.884208
H	0	5.136184	-4.704369	-3.786329
H	0	4.049555	-2.736761	-2.760476
H	0	4.600140	0.099874	1.553226
H	0	5.936894	2.033190	2.252700
H	0	8.349177	1.642703	-1.282454
H	0	7.015497	-0.294237	-1.978101
H	0	6.386047	4.264876	1.769200
H	0	7.731596	6.199997	2.487148
H	0	10.184058	6.245318	2.072235
H	0	11.271417	4.312593	0.944938
H	0	9.933553	2.359244	0.262547

SCF Done: E(RPBE1PBE) = -1958.12832794 A.U. after 16 cycles

Zero-point correction= 0.702712 (Hartree/Particle)

Thermal correction to Energy= 0.742250

Thermal correction to Enthalpy= 0.743194

Thermal correction to Gibbs Free Energy= 0.625119

Sum of electronic and zero-point Energies= -1957.425686

Sum of electronic and thermal Energies= -1957.386148

Sum of electronic and thermal Enthalpies= -1957.385204

Sum of electronic and thermal Free Energies= -1957.503279

7.19.2 Computed excitations of compound 6b (PBE1PBE/6-31+G** PCM CH_2Cl_2)

Inputfile:

%NProcShared=10

%Chk=gauss.chk

%mem=10GB

#p PBE1PBE 6-31+G** geom=check guess=read SCRF=(Solvent=Dichloromethane)

TD=(Nstates=15,root=1)

Excitation analysis by Multiwfn:

Integral of hole: 0.975467

Integral of electron: 0.975402

Integral of transition density: 0.000007

Transition dipole moment in X/Y/Z: -3.533566 -0.082615 0.000315 a.u.

Sm index (integral of Sm function): 0.37348 a.u.

Sr index (integral of Sr function): **0.63742 a.u.**

Centroid of hole in X/Y/Z: 0.000440 0.000010 0.630854 Angstrom

Centroid of electron in X/Y/Z: 0.002442 0.000366 0.157532 Angstrom

D_x: 0.002 D_y: 0.000 D_z: 0.473 D index: **0.473 Angstrom**

Variation of dipole moment with respect to ground state:

X: -0.003689 Y: -0.000656 Z: 0.872477 Norm: 0.872485 a.u.

RMSD of hole in X/Y/Z: 4.801 1.642 0.931 Norm: 5.159 Angstrom

RMSD of electron in X/Y/Z: 5.438 1.736 1.798 Norm: 5.985 Angstrom

Difference between RMSD of hole and electron (delta sigma):

X: 0.637 Y: 0.094 Z: 0.866 Overall: 0.826 Angstrom

H_x: 5.120 H_y: 1.689 H_z: 1.365 H_CT: 1.365 H index: 5.572 Angstrom

t index: -0.891 Angstrom

Hole delocalization index (HDI): 6.13

Electron delocalization index (EDI): 3.72

Ghost-hunter index: -26.333 eV, 1st term: 4.090 eV, 2nd term: 30.422 eV

Excitation energy of this state: 3.312 eV

Excitation energies and oscillator strengths:

Excited State 1: Singlet-A 3.3120 eV 374.35 nm f=1.0233 <S**2>=0.000

168 -> 171 -0.16175

169 -> 170 0.67940

This state for optimization and/or second-order correction.

Total Energy, E(TD-HF/TD-DFT) = -1958.00668362
Copying the excited state density for this state as the 1-particle RhoCI density.

Excited State 2: Singlet-A 3.4996 eV 354.28 nm f=0.0253 <S**2>=0.000
168 -> 170 -0.22858
168 -> 172 -0.15962
169 -> 171 0.64177

Excited State 3: Singlet-A 3.6491 eV 339.76 nm f=0.3372 <S**2>=0.000
168 -> 171 -0.22330
169 -> 172 0.64989

Excited State 4: Singlet-A 3.7676 eV 329.08 nm f=0.0095 <S**2>=0.000
168 -> 170 0.12794
168 -> 174 -0.25254
169 -> 173 0.63273

Excited State 5: Singlet-A 3.7900 eV 327.14 nm f=0.2288 <S**2>=0.000
168 -> 173 -0.25821
169 -> 174 0.64223

Excited State 6: Singlet-A 3.9804 eV 311.49 nm f=0.0062 <S**2>=0.000
168 -> 170 0.59151
168 -> 172 -0.16222
168 -> 174 0.10254
169 -> 171 0.15253
169 -> 173 -0.12220
169 -> 175 -0.23427

Excited State 7: Singlet-A 4.0863 eV 303.41 nm f=0.0012 <S**2>=0.000
168 -> 170 0.17789
168 -> 176 0.14710
169 -> 171 0.11654
169 -> 175 0.54651
169 -> 177 -0.30640

Excited State 8: Singlet-A 4.1875 eV 296.08 nm f=0.0408 <S**2>=0.000

168 -> 175 0.22712
168 -> 179 -0.12247
169 -> 176 0.57050
169 -> 178 -0.25780

Excited State 9: Singlet-A 4.1971 eV 295.40 nm f=0.0007 <S**2>=0.000
168 -> 170 0.15442
168 -> 172 0.32864
168 -> 176 0.10655
169 -> 171 0.14360
169 -> 175 0.24493
169 -> 177 0.48320
169 -> 179 0.10550
169 -> 180 -0.10159

Excited State 10: Singlet-A 4.2289 eV 293.19 nm f=0.0086 <S**2>=0.000
168 -> 171 0.62692
169 -> 170 0.15993
169 -> 172 0.21823

Excited State 11: Singlet-A 4.3167 eV 287.22 nm f=0.0737 <S**2>=0.000
168 -> 177 0.21927
169 -> 176 0.26824
169 -> 178 0.55415
169 -> 181 0.19150

Excited State 12: Singlet-A 4.3173 eV 287.18 nm f=0.0034 <S**2>=0.000
168 -> 170 0.12075
168 -> 172 0.30729
168 -> 176 -0.17078
169 -> 171 0.11972
169 -> 177 -0.26912
169 -> 179 0.46762
169 -> 180 0.11305

Excited State 13: Singlet-A 4.4130 eV 280.95 nm f=0.0044 <S**2>=0.000
168 -> 172 0.43775

168 -> 174 0.10951

168 -> 178 -0.11585

169 -> 171 0.10062

169 -> 175 -0.14958

169 -> 177 -0.20538

169 -> 179 -0.36606

169 -> 180 -0.19558

Excited State 14: Singlet-A 4.4892 eV 276.18 nm f=0.0244 <S**2>=0.000

168 -> 173 -0.22241

168 -> 180 -0.22023

169 -> 176 -0.11786

169 -> 178 -0.16882

169 -> 181 0.56451

Excited State 15: Singlet-A 4.5096 eV 274.94 nm f=0.0026 <S**2>=0.000

168 -> 172 0.15964

168 -> 178 0.12127

168 -> 181 -0.19031

169 -> 179 -0.23373

169 -> 180 0.57917

7.19.3 Computed emission of compound 6b (PBE1PBE/6-31+G** PCM CH_2Cl_2)

Inputfile:

%NProcShared=20

%Chk=gauss.chk

%mem=20GB

#p PBE1PBE 6-31+G** opt freq

SCRF=(Solvent=Dichloromethane) TD=(Nstates=6,root=1)

--link1--

%NProcShared=20

%Chk=gauss.chk

%mem=20GB

#p PBE1PBE 6-31+G** geom=check guess=read

SCRF=(Solvent=Dichloromethane) TD=(Nstates=6,root=1)

Excitation analysis by Multiwfn:

Integral of hole: 0.968322

Integral of electron: 0.968317

Integral of transition density: -0.000011

Transition dipole moment in X/Y/Z: -4.752705 -0.298962 -0.001686 a.u.

Sm index (integral of Sm function): 0.45593 a.u.

Sr index (integral of Sr function): 0.72883 a.u.

Centroid of hole in X/Y/Z: -0.000044 0.003163 -0.527370 Angstrom

Centroid of electron in X/Y/Z: 0.000376 0.002812 -0.499992 Angstrom

D_x: 0.000 D_y: 0.000 D_z: 0.027 **D index: 0.027 Angstrom**

Variation of dipole moment with respect to ground state:

X: -0.000768 Y: 0.000642 Z: -0.050099 Norm: 0.050109 a.u.

RMSD of hole in X/Y/Z: 4.636 1.539 1.093 Norm: 5.005 Angstrom

RMSD of electron in X/Y/Z: 4.237 1.355 1.308 Norm: 4.636 Angstrom

Difference between RMSD of hole and electron (delta sigma):

X: -0.399 Y: -0.184 Z: 0.215 Overall: -0.369 Angstrom

H_x: 4.436 H_y: 1.447 H_z: 1.201 H_CT: 1.202 H index: 4.821 Angstrom

t index: -1.175 Angstrom

Hole delocalization index (HDI): 5.95

Electron delocalization index (EDI): 5.22

Ghost-hunter index: -522.170 eV, 1st term: 3.671 eV, 2nd term: 525.842 eV

Excitation energy of this state: 3.014 eV

Excitation energies and oscillator strengths:

Excited State 1: Singlet-A 3.0139 eV 411.37 nm f=1.5067 <S**2>=0.000

169 ->170 0.69580

This state for optimization and/or second-order correction.

Total Energy, E(TD-HF/TD-DFT) = -1957.45756074

Copying the excited state density for this state as the 1-particle RhoCI density.

Excited State 2: Singlet-A 3.4403 eV 360.39 nm f=0.0065 <S**2>=0.000

168 ->170 0.15774

168 ->172 -0.17450

169 ->171 0.66142

Excited State 3: Singlet-A 3.5656 eV 347.73 nm f=0.2230 <S**2>=0.000

168 ->171 -0.20394

169 ->172 0.66838

Excited State 4: Singlet-A 3.7619 eV 329.57 nm f=0.0082 <S**2>=0.000

168 ->170 -0.45446

168 ->174 0.12962

169 ->173 0.49649

Excited State 5: Singlet-A 3.8087 eV 325.53 nm f=0.0325 <S**2>=0.000

168 ->170 0.50755

168 ->174 0.10577

169 ->171 -0.11743

169 ->173 0.44330

Excited State 6: Singlet-A 3.8683 eV 320.51 nm f=0.0980 <S**2>=0.000

168 ->173 0.21908

169 ->174 0.66134

7.20 Quantum chemical calculation data of 2',2'''-([1,1'-Biphenyl]-4,4'-diylbis(phenylazanediyl))bis(([1,1'-biphenyl]-4-carbonitrile)) (6c)

7.20.1 Computed xyz-coordinates of compound 6c (PBE1PBE/6-31+G** PCM CH_2Cl_2)

Inputfile:

%NProcShared=10

%Chk=gauss.chk

%mem=10GB

#p PBE1PBE 6-31+G**

pop=full GFInput opt freq SCRF=(Solvent=Dichloromethane)

Optimized S_0-geometry:

0 1

C 0 -7.590352 0.773644 0.897797

C 0 -6.801464 -0.307091 1.264313

C 0 -5.707444 -0.693090 0.474821

C 0 -5.429084 0.045710 -0.685379

C 0 -6.213775 1.135555 -1.033705

C 0 -7.315137 1.524280 -0.255509

N 0 -4.913352 -1.794135 0.836807

C 0 -3.521425 -1.786861 0.594151

C 0 -5.504003 -2.926354 1.451293

C 0 -2.876744 -2.934838 0.116728

C 0 -1.505989 -2.932033 -0.106991

C 0 -0.728096 -1.786767 0.122592

C 0 -1.387608 -0.641804 0.596030

C 0 -2.755543 -0.639238 0.835906

C 0 -4.894359 -3.525413 2.560304

C 0 -5.468855 -4.645805 3.154249

C 0 -6.662880 -5.173766 2.662757

C 0 -7.274066 -4.573308 1.561724

C 0 -6.697356 -3.462691 0.952082

C 0 -8.152262 2.677620 -0.635213

C	0	-9.533550	2.683814	-0.376564
C	0	-10.326386	3.765796	-0.728025
C	0	-9.748186	4.877011	-1.358590
C	0	-8.371850	4.888267	-1.627379
C	0	-7.590833	3.801161	-1.265417
C	0	0.728098	-1.786767	-0.122583
C	0	1.387610	-0.641804	-0.596020
C	0	2.755545	-0.639238	-0.835898
C	0	3.521426	-1.786861	-0.594146
C	0	2.876746	-2.934839	-0.116722
C	0	1.505991	-2.932034	0.106999
N	0	4.913352	-1.794136	-0.836804
C	0	5.504003	-2.926353	-1.451295
C	0	5.707445	-0.693091	-0.474820
C	0	6.697356	-3.462694	-0.952086
C	0	7.274064	-4.573309	-1.561733
C	0	6.662877	-5.173762	-2.662767
C	0	5.468852	-4.645799	-3.154256
C	0	4.894356	-3.525409	-2.560307
C	0	5.429087	0.045708	0.685381
C	0	6.213777	1.135554	1.033706
C	0	7.315138	1.524279	0.255508
C	0	7.590351	0.773645	-0.897800
C	0	6.801463	-0.307091	-1.264315
C	0	8.152263	2.677619	0.635211
C	0	7.590832	3.801164	1.265408
C	0	8.371849	4.888270	1.627371
C	0	9.748186	4.877010	1.358589
C	0	10.326388	3.765793	0.728030
C	0	9.533552	2.683810	0.376569
C	0	-10.558749	5.994576	-1.726265
N	0	-11.218445	6.904674	-2.025834
C	0	10.558750	5.994574	1.726266

N	0	11.218439	6.904677	2.025834
H	0	-8.412297	1.062163	1.547120
H	0	-7.025411	-0.852758	2.175534
H	0	-4.599852	-0.245992	-1.322174
H	0	-5.987219	1.667183	-1.953880
H	0	-3.456789	-3.829798	-0.087773
H	0	-1.039161	-3.829453	-0.503695
H	0	-0.815748	0.254634	0.819931
H	0	-3.234592	0.254208	1.225191
H	0	-3.970404	-3.110249	2.951981
H	0	-4.985010	-5.100011	4.014481
H	0	-7.111765	-6.044165	3.131947
H	0	-8.199739	-4.979270	1.163301
H	0	-7.168800	-3.006614	0.086498
H	0	-9.999546	1.818567	0.084728
H	0	-11.392827	3.750788	-0.527515
H	0	-7.920728	5.751127	-2.106629
H	0	-6.522198	3.835623	-1.453328
H	0	0.815750	0.254635	-0.819919
H	0	3.234594	0.254209	-1.225182
H	0	3.456791	-3.829799	0.087777
H	0	1.039163	-3.829453	0.503703
H	0	7.168800	-3.006619	-0.086502
H	0	8.199737	-4.979273	-1.163312
H	0	7.111761	-6.044161	-3.131961
H	0	4.985005	-5.100002	-4.014489
H	0	3.970402	-3.110243	-2.951982
H	0	4.599856	-0.245995	1.322177
H	0	5.987224	1.667182	1.953882
H	0	8.412295	1.062164	-1.547123
H	0	7.025408	-0.852757	-2.175536
H	0	6.522196	3.835626	1.453313
H	0	7.920726	5.751132	2.106616

H 0   11.392830   3.750782   0.527525

H 0   9.999548   1.818561   -0.084718

SCF Done: E(RPBE1PBE) = -2142.41916128   A.U. after   16 cycles

Zero-point correction=   0.699919 (Hartree/Particle)

Thermal correction to Energy=   0.743164

Thermal correction to Enthalpy=   0.744108

Thermal correction to Gibbs Free Energy=   0.616392

Sum of electronic and zero-point Energies=   -2141.719486

Sum of electronic and thermal Energies=   -2141.676241

Sum of electronic and thermal Enthalpies=   -2141.675297

Sum of electronic and thermal Free Energies=   -2141.803013

7.20.2 Computed excitations of compound 6c (PBE1PBE/6-31+G PCM CH_2Cl_2)**

Inputfile:

%NProcShared=10

%Chk=gauss.chk

%mem=10GB

#p PBE1PBE 6-31+G** geom=check guess=read

SCRF=(Solvent=Dichloromethane) TD=(Nstates=15,root=1)

Excitation analysis by Multiwfn:

Integral of hole: 0.983684

Integral of electron: 0.983644

Integral of transition density: -0.000012

Transition dipole moment in X/Y/Z: 1.776553 -0.000015   0.200272 a.u.

Sm index (integral of Sm function):   0.13184 a.u.

Sr index (integral of Sr function):   **0.35076 a.u.**

Centroid of hole in X/Y/Z: -0.000158   -0.982633   -0.000043 Angstrom

Centroid of electron in X/Y/Z: 0.001121   1.626330   0.000211 Angstrom

D_x: 0.001 D_y: 2.609 D_z: 0.000   D index: **2.609 Angstrom**

Variation of dipole moment with respect to ground state:

X: -0.002378 Y: -4.849685 Z: -0.000472   Norm: 4.849686 a.u.

RMSD of hole in X/Y/Z: 4.855   0.955   1.654   Norm: 5.217 Angstrom

RMSD of electron in X/Y/Z: 7.236   2.529   1.442   Norm: 7.800 Angstrom

Difference between RMSD of hole and electron (delta sigma):

X: 2.382 Y: 1.574 Z: -0.212   Overall: 2.583 Angstrom

H_x: 6.045 H_y: 1.742 H_z: 1.548 H_CT: 1.742 H index: 6.508 Angstrom

t index: 0.867 Angstrom

Hole delocalization index (HDI):   6.14

Electron delocalization index (EDI):   4.69

Ghost-hunter index: -1.924 eV, 1st term: 3.595 eV, 2nd term: 5.519 eV

Excitation energy of this state: 2.890 eV

Excitation energies and oscillator strengths:

Excited State   1:   Singlet-A   2.8896 eV 429.07 nm f=0.2831 <S**2>=0.000

180 -> 183   -0.23675

181 -> 182   0.66015

This state for optimization and/or second-order correction.

Total Energy, E(TD-HF/TD-DFT) = -2142.31321303

Copying the excited state density for this state as the 1-particle RhoCI density.

Excited State   2:   Singlet-A   2.9463 eV 420.82 nm f=0.0272 <S**2>=0.000

180 -> 182   -0.25905

181 -> 183   0.65334

Excited State   3:   Singlet-A   3.5247 eV 351.76 nm f=1.0132 <S**2>=0.000

180 -> 183   0.24288

181 -> 184   0.64522

Excited State   4:   Singlet-A   3.5740 eV 346.91 nm f=0.0057 <S**2>=0.000

180 -> 182   0.64713

181 -> 183   0.26128

Excited State   5:   Singlet-A   3.6272 eV 341.81 nm f=0.0805 <S**2>=0.000

180 -> 183   0.60986

181 -> 182   0.24668

181 -> 184   -0.23515

Excited State   6:   Singlet-A   3.7478 eV 330.82 nm f=0.0130 <S**2>=0.000

180 -> 186   -0.24448

181 -> 185   0.59552

181 -> 187 0.23822

Excited State 7: Singlet-A 3.7754 eV 328.40 nm f=0.1275 <S**2>=0.000
180 -> 185 -0.21095
180 -> 187 -0.16070
181 -> 186 0.63899

Excited State 8: Singlet-A 3.8958 eV 318.25 nm f=0.0059 <S**2>=0.000
180 -> 184 -0.11386
180 -> 188 0.22515
181 -> 185 -0.22598
181 -> 187 0.58596
181 -> 189 0.14719

Excited State 9: Singlet-A 3.9352 eV 315.06 nm f=0.0640 <S**2>=0.000
180 -> 185 -0.14232
180 -> 187 0.20796
181 -> 188 0.63269
181 -> 190 -0.11885

Excited State 10: Singlet-A 4.0627 eV 305.18 nm f=0.0010 <S**2>=0.000
180 -> 184 0.28674
180 -> 190 -0.14121
181 -> 189 0.58106
181 -> 191 0.15403

Excited State 11: Singlet-A 4.1363 eV 299.75 nm f=0.0009 <S**2>=0.000
180 -> 184 0.54966
180 -> 188 0.12534
180 -> 190 0.11433
181 -> 185 -0.13069
181 -> 189 -0.25421
181 -> 191 0.20961
181 -> 193 -0.11432

Excited State 12: Singlet-A 4.1470 eV 298.98 nm f=0.1515 <S**2>=0.000
180 -> 189 -0.24884

181 -> 188 0.13498
181 -> 190 0.62504

Excited State 13: Singlet-A 4.3426 eV 285.51 nm f=0.0002 <S**2>=0.000
180 -> 184 -0.22364
180 -> 190 0.14350
180 -> 192 -0.15675
181 -> 191 0.57215
181 -> 193 0.19971

Excited State 14: Singlet-A 4.3986 eV 281.87 nm f=0.0316 <S**2>=0.000
180 -> 191 -0.23178
180 -> 194 -0.10773
181 -> 192 0.60306
181 -> 195 0.14584

Excited State 15: Singlet-A 4.4163 eV 280.74 nm f=0.0002 <S**2>=0.000
180 -> 184 0.13603
180 -> 186 -0.26795
180 -> 188 -0.11232
180 -> 190 0.12517
180 -> 192 0.13182
180 -> 195 -0.11665
181 -> 185 -0.13517
181 -> 191 -0.16196
181 -> 193 0.42943
181 -> 194 -0.30290

7.20.3 Computed emission of compound 6c (PBE1PBE/6-31+G PCM CH_2Cl_2)**

Inputfile:

%NProcShared=10

%Chk=gauss.chk

%mem=10GB

#p PBE1PBE 6-31+G** opt freq

SCRF=(Solvent=Dichloromethane) TD=(Nstates=6,root=1)

--link1--

%NProcShared=10

%Chk=gauss.chk

%mem=10GB

#p PBE1PBE 6-31+G** geom=check guess=read

SCRF=(Solvent=Dichloromethane) TD=(Nstates=6,root=1)

Excitation analysis by Multiwfn:

Integral of hole: 0.991541

Integral of electron: 0.991558

Integral of transition density: -0.000002

Transition dipole moment in X/Y/Z: 1.378385 -0.762088 0.199034 a.u.

Sm index (integral of Sm function): 0.08725 a.u.

Sr index (integral of Sr function): 0.29165 a.u.

Centroid of hole in X/Y/Z: -2.020709 -1.074998 0.186068 Angstrom

Centroid of electron in X/Y/Z: -6.804704 1.646632 -0.698636 Angstrom

D_x: 4.784 D_y: 2.722 D_z: 0.885 **D index: 5.575 Angstrom**

Variation of dipole moment with respect to ground state:

X: 8.964048 Y: -5.099676 Z: 1.657719 Norm: 10.445520 a.u.

RMSD of hole in X/Y/Z: 4.045 0.956 1.508 Norm: 4.422 Angstrom

RMSD of electron in X/Y/Z: 1.512 2.686 1.356 Norm: 3.368 Angstrom

Difference between RMSD of hole and electron (delta sigma):

X: -2.533 Y: 1.730 Z: -0.153 Overall: -1.054 Angstrom

H_x: 2.778 H_y: 1.821 H_z: 1.432 H_CT: 2.555 H index: 3.895 Angstrom

t index: 3.020 Angstrom

Hole delocalization index (HDI): 6.74

Electron delocalization index (EDI): 6.76

Ghost-hunter index: 0.351 eV, 1st term: 2.934 eV, 2nd term: 2.583 eV

Excitation energy of this state: 2.296 eV

Excitation energies and oscillator strengths:

Excited State 1: Singlet-A 2.3508 eV 527.41 nm f=0.1554 <S**2>=0.000

180 ->182 -0.16999

181 ->182 0.68295

This state for optimization and/or second-order correction.

Total Energy, E(TD-HF/TD-DFT) = -2141.76167729

Copying the excited state density for this state as the 1-particle RhoCI density.

Excited State 2: Singlet-A 2.7788 eV 446.19 nm f=0.1994 <S**2>=0.000

180 ->183 0.17238

181 ->183 0.67928

Excited State 3: Singlet-A 3.2278 eV 384.11 nm f=0.0232 <S**2>=0.000

180 ->182 0.67631

181 ->182 0.17417

Excited State 4: Singlet-A 3.3237 eV 373.03 nm f=1.4168 <S**2>=0.000

181 ->184 -0.69171

Excited State 5: Singlet-A 3.5727 eV 347.03 nm f=0.0224 <S**2>=0.000

180 ->185 0.11213

180 ->186 -0.13318

181 ->185 -0.63620

181 ->186 -0.14873

181 ->188 0.14143

Excited State 6: Singlet-A 3.6116 eV 343.29 nm f=0.0698 <S**2>=0.000

180 ->183 0.34680

180 ->185 0.13989

181 ->185 -0.15212

181 ->186 0.52760

181 ->187 0.14176

7.21 Quantum chemical calculation data of 2',2'''-([1,1'-Biphenyl]-4,4'-diylbis(phenylazanediyl))bis(([1,1'-biphenyl]-2,5-dicarbonitrile)) (6d)

7.21.1 Computed xyz-coordinates of compound 6d (PBE1PBE/6-31+G** PCM CH_2Cl_2)

Input file:

%NProcShared=16

%Chk=gauss.chk

%mem=10GB

#p PBE1PBE 6-31+G** pop=full GFInput opt SCRF=(Solvent=Dichloromethane)

Optimized S_0-geometry:

0 1

C	0	-3.528811	-0.415649	0.491576
C	0	-5.737791	0.435718	1.055153
C	0	-2.784874	-0.910770	-0.585960
C	0	-1.410988	-0.721629	-0.636894
C	0	-0.730769	-0.021710	0.373363
C	0	-1.487735	0.468317	1.446377
C	0	-2.862787	0.269092	1.512634
C	0	-5.669698	1.735747	0.510639
C	0	-6.419615	2.762245	1.094756
C	0	-7.263545	2.515728	2.173402
C	0	-7.350229	1.224137	2.690370
C	0	-6.586041	0.199635	2.144112
N	0	-4.934116	-0.607244	0.532141
C	0	-5.452346	-1.924632	0.466555
C	0	-4.821881	2.057245	-0.667884
C	0	-4.686054	-3.023908	0.875859
C	0	-5.207137	-4.313268	0.792926
C	0	-6.500404	-4.528366	0.320205
C	0	-7.267867	-3.432708	-0.077848
C	0	-6.750854	-2.143062	-0.014840
C	0	-5.160306	1.626631	-1.965798

C	0	-4.363472	1.949517	-3.073220
C	0	-3.223441	2.718387	-2.907049
C	0	-2.890873	3.174350	-1.624915
C	0	-3.683083	2.846521	-0.518879
C	0	0.730176	0.190881	0.306507
C	0	1.519869	0.181132	1.468221
C	0	2.893019	0.371521	1.410442
C	0	3.527009	0.592875	0.181711
C	0	2.751831	0.617616	-0.981743
C	0	1.377931	0.411617	-0.916665
N	0	4.931130	0.783060	0.133298
C	0	5.511670	1.818056	0.909308
C	0	5.646439	0.252399	-0.968269
C	0	4.772693	2.953481	1.266402
C	0	5.355290	3.955778	2.038941
C	0	6.682513	3.853548	2.451994
C	0	7.422359	2.727970	2.086590
C	0	6.844849	1.714032	1.329493
C	0	5.561999	-1.119674	-1.287321
C	0	6.223555	-1.599676	-2.422817
C	0	6.997508	-0.759341	-3.217254
C	0	7.101798	0.589771	-2.882717
C	0	6.423598	1.090496	-1.777648
C	0	4.788037	-2.081819	-0.458771
C	0	3.610778	-2.641596	-0.951423
C	0	2.886413	-3.567364	-0.192122
C	0	3.327024	-3.954102	1.080085
C	0	4.506150	-3.421337	1.574333
C	0	5.234926	-2.498719	0.810454
C	0	-6.366184	0.888698	-2.191593
N	0	-7.351612	0.316432	-2.418683
C	0	-1.725413	3.985409	-1.439341
N	0	-0.780710	4.645308	-1.290825

C	0	6.482808	-2.026046	1.329052
N	0	7.504116	-1.689295	1.769131
C	0	1.679338	-4.121865	-0.726835
N	0	0.701471	-4.572878	-1.163146
H	0	-3.290153	-1.452873	-1.380085
H	0	-0.854863	-1.135475	-1.473458
H	0	-0.999827	1.028846	2.239104
H	0	-3.425836	0.659364	2.355726
H	0	-6.355599	3.761536	0.672340
H	0	-7.849586	3.323221	2.601075
H	0	-7.999476	1.014769	3.535642
H	0	-6.632230	-0.799396	2.566495
H	0	-3.682199	-2.870398	1.258910
H	0	-4.596291	-5.152808	1.113408
H	0	-6.904831	-5.534399	0.260812
H	0	-8.275473	-3.581321	-0.456429
H	0	-7.348501	-1.300654	-0.347283
H	0	-4.650751	1.604535	-4.060651
H	0	-2.602245	2.973215	-3.758529
H	0	-3.403283	3.197453	0.468651
H	0	1.057379	-0.016384	2.431433
H	0	3.487205	0.338098	2.319114
H	0	3.226006	0.807223	-1.940535
H	0	0.796700	0.458649	-1.833621
H	0	3.742870	3.053053	0.938171
H	0	4.765968	4.828720	2.306089
H	0	7.134905	4.638890	3.050174
H	0	8.456385	2.627998	2.405076
H	0	7.422424	0.833261	1.068393
H	0	6.147491	-2.656693	-2.663693
H	0	7.516217	-1.156085	-4.084390
H	0	7.696616	1.261673	-3.494723
H	0	6.481875	2.147153	-1.535444

H 0 3.248206 -2.344038 -1.929511

H 0 2.757947 -4.668277 1.664772

H 0 4.876712 -3.724384 2.547632

SCF Done: E(RPBE1PBE) = -2326.69346548 A.U. after 3 cycles

Zero-point correction= 0.696441 (Hartree/Particle)

Thermal correction to Energy= 0.743489

Thermal correction to Enthalpy= 0.744434

Thermal correction to Gibbs Free Energy= 0.608679

Sum of electronic and zero-point Energies= -2325.997025

Sum of electronic and thermal Energies= -2325.949976

Sum of electronic and thermal Enthalpies= -2325.949032

Sum of electronic and thermal Free Energies= -2326.084786

7.21.1.1 Computed excitation of compound 6d (PBE1PBE/6-31+G PCM CH_2Cl_2)**

Inputfile:

%NProcShared=4

%Chk=gauss.chk

%mem=10GB

#p PBE1PBE 6-31+G** geom=check guess=read SCRF=(Solvent=Dichloromethan

e, correctedLR) TD=(Nstates=15,root=1)

Excitation energies and oscillator strengths:

Excited State 1: Singlet-A 2.4118 eV 514.08 nm f=0.0128 <S**2>=0.000

192 -> 195 0.25254

193 -> 194 0.62332

193 -> 195 0.21104

This state for optimization and/or second-order correction.

Total Energy, E(TD-HF/TD-DFT) = -2326.60483405

Copying the excited state density for this state as the 1-particle RhoCI density.

Excited State 2: Singlet-A 2.4184 eV 512.67 nm f=0.0007 <S**2>=0.000

192 -> 194 0.25790

193 -> 194 -0.20884

193 -> 195 0.62195

Excited State 3: Singlet-A 2.9222 eV 424.29 nm f=0.0008 <S**2>=0.000

192 -> 194 0.64013

192 -> 195 -0.13351

193 -> 194 0.15128

193 -> 195 -0.21179

Excited State 4: Singlet-A 2.9287 eV 423.34 nm f=0.0055 <S**2>=0.000

192 -> 194 0.13426

192 -> 195 0.64249

193 -> 194 -0.20688

193 -> 195 -0.14669

Excited State 5: Singlet-A 3.4251 eV 361.99 nm f=0.7401 <S**2>=0.000

193 -> 196 0.66778

193 -> 198 0.16944

Excited State 6: Singlet-A 3.5439 eV 349.85 nm f=0.0141 <S**2>=0.000

192 -> 196 0.22198

192 -> 198 -0.17959

193 -> 197 0.63963

Excited State 7: Singlet-A 3.5797 eV 346.36 nm f=0.2555 <S**2>=0.000

192 -> 197 -0.26461

193 -> 196 -0.13271

193 -> 198 0.62991

Excited State 8: Singlet-A 3.8326 eV 323.49 nm f=0.0041 <S**2>=0.000

192 -> 196 0.36857

192 -> 198 0.21572

192 -> 200 0.21820

193 -> 199 0.48835

Excited State 9: Singlet-A 3.8593 eV 321.26 nm f=0.0008 <S**2>=0.000
192 -> 196 0.45798
192 -> 198 0.21922
192 -> 200 -0.19144
193 -> 199 -0.39813
193 -> 201 0.10923

Excited State 10: Singlet-A 3.8867 eV 318.99 nm f=0.0405 <S**2>=0.000
192 -> 199 0.34396
193 -> 200 0.60017

Excited State 11: Singlet-A 3.9748 eV 311.93 nm f=0.0386 <S**2>=0.000
191 -> 194 0.68653

Excited State 12: Singlet-A 3.9860 eV 311.05 nm f=0.0032 <S**2>=0.000
191 -> 195 0.68191

Excited State 13: Singlet-A 4.0902 eV 303.12 nm f=0.0199 <S**2>=0.000
192 -> 196 -0.27610
192 -> 198 0.43919
192 -> 202 -0.14550
193 -> 197 0.23835
193 -> 201 0.36253

Excited State 14: Singlet-A 4.1024 eV 302.22 nm f=0.0800 <S**2>=0.000
192 -> 197 0.62498
193 -> 196 -0.15360
193 -> 198 0.23932
193 -> 202 0.11841

Excited State 15: Singlet-A 4.1749 eV 296.97 nm f=0.0838 <S**2>=0.000

187 -> 194 0.25950

187 -> 195 0.10906

188 -> 195 0.31835

189 -> 194 -0.12580

190 -> 195 -0.19137

192 -> 197 -0.12260

192 -> 201 -0.21454

193 -> 202 0.40823

7.21.2 Computed xyz-coordinates of compound 6d (ωB97XD/6-31+G PCM CH_2Cl_2)**

Input file:

%NProcShared=16

%Chk=gauss.chk

%mem=10GB

#p wB97XD 6-31+G** pop=full GFInput opt SCRF=(Solvent=Dichloromethane)

Optimized S_0-geometry:

0 1

C 0 -3.541251 -0.376758 0.569508

C 0 -5.694572 0.691059 0.960197

C 0 -2.818069 -0.880122 -0.514710

C 0 -1.442783 -0.717032 -0.571478

C 0 -0.750010 -0.039600 0.442015

C 0 -1.481614 0.432687 1.536943

C 0 -2.859797 0.256589 1.608948

C 0 -5.449595 1.931612 0.338205

C 0 -6.139658 3.065945 0.771992

C 0 -7.093837 2.982509 1.781068

C 0 -7.350496 1.749939 2.378634

C 0 -6.647929 0.618628 1.980770

N 0 -4.960715 -0.459829 0.575022

C 0 -5.590386 -1.724210 0.490622

C 0 -4.450741 2.059868 -0.759402

C 0	-4.925211	-2.883347	0.908196
C 0	-5.545866	-4.125522	0.800328
C 0	-6.840008	-4.231783	0.295552
C 0	-7.506438	-3.075334	-0.110758
C 0	-6.888914	-1.832604	-0.024384
C 0	-4.689578	1.540965	-2.042347
C 0	-3.721829	1.613519	-3.051241
C 0	-2.497842	2.208803	-2.792328
C 0	-2.260068	2.750429	-1.524510
C 0	-3.229564	2.685287	-0.521927
C 0	0.713101	0.180192	0.342752
C 0	1.536624	0.105627	1.475056
C 0	2.906866	0.297207	1.378048
C 0	3.492045	0.575150	0.140120
C 0	2.681240	0.682922	-0.989616
C 0	1.310061	0.478470	-0.886886
N 0	4.905437	0.694116	0.035662
C 0	5.575748	1.701164	0.771216
C 0	5.549248	0.041224	-1.046250
C 0	4.904732	2.863715	1.168564
C 0	5.569720	3.837691	1.909931
C 0	6.911580	3.679120	2.249214
C 0	7.582754	2.525077	1.843333
C 0	6.923256	1.538780	1.118512
C 0	5.335672	-1.333399	-1.270187
C 0	5.926891	-1.950407	-2.374757
C 0	6.756024	-1.236959	-3.234818
C 0	6.984701	0.117061	-2.997137
C 0	6.376626	0.752591	-1.920718
C 0	4.477232	-2.134351	-0.353818
C 0	3.209688	-2.544192	-0.759045
C 0	2.374776	-3.236500	0.120312
C 0	2.795154	-3.548306	1.417688

C 0	4.066172	-3.173663	1.821894
C 0	4.899768	-2.473941	0.941748
C 0	-5.955325	0.929591	-2.343740
N 0	-6.975698	0.453263	-2.619718
C 0	-0.995986	3.372510	-1.236442
N 0	0.021156	3.876818	-1.001535
C 0	6.219031	-2.111695	1.383551
N 0	7.285653	-1.852438	1.756827
C 0	1.060074	-3.621700	-0.316675
N 0	0.002862	-3.937367	-0.672738
H 0	-3.343309	-1.385745	-1.318827
H 0	-0.898429	-1.124319	-1.417478
H 0	-0.976343	0.970529	2.333554
H 0	-3.415440	0.650671	2.454332
H 0	-5.936600	4.018494	0.291454
H 0	-7.632080	3.870271	2.095065
H 0	-8.087184	1.669817	3.171500
H 0	-6.830413	-0.335376	2.464279
H 0	-3.921473	-2.814656	1.313814
H 0	-5.012192	-5.013416	1.125258
H 0	-7.322450	-5.200563	0.217643
H 0	-8.512473	-3.139163	-0.513948
H 0	-7.408801	-0.943462	-0.363519
H 0	-3.931881	1.200748	-4.031028
H 0	-1.736909	2.259047	-3.562126
H 0	-3.020625	3.090376	0.461506
H 0	1.106139	-0.147360	2.439360
H 0	3.538127	0.202000	2.255986
H 0	3.127733	0.913680	-1.951888
H 0	0.693271	0.579749	-1.774946
H 0	3.863901	3.006847	0.898197
H 0	5.032693	4.731936	2.211070
H 0	7.428008	4.442537	2.821828

H 0	8.626782	2.381415	2.104284
H 0	7.448348	0.636317	0.825447
H 0	5.749080	-3.008646	-2.541998
H 0	7.220447	-1.734476	-4.079350
H 0	7.624279	0.688066	-3.662473
H 0	6.534958	1.812635	-1.752360
H 0	2.859741	-2.289672	-1.752953
H 0	2.137065	-4.078931	2.095418
H 0	4.416666	-3.418318	2.817790

SCF Done: E(RwB97XD) = -2328.64061650 A.U. after 2 cycles

Zero-point correction= 0.700129 (Hartree/Particle)

Thermal correction to Energy= 0.746412

Thermal correction to Enthalpy= 0.747356

Thermal correction to Gibbs Free Energy= 0.616306

Sum of electronic and zero-point Energies= -2327.940487

Sum of electronic and thermal Energies= -2327.894205

Sum of electronic and thermal Enthalpies= -2327.893261

Sum of electronic and thermal Free Energies= -2328.024311

7.21.2.1 Computed excitation of compound 6d (ωB97XD /6-31+G PCM CH_2Cl_2)**

Input file:

%NProcShared=4

%Chk=gauss.chk

%mem=10GB

#p wB97XD 6-31+G** geom=check guess=read SCRF=(Solvent=Dichloromethane, correctedLR) TD=(Nstates=15,root=1)

Excitation energies and oscillator strengths:

Excited State 1: Singlet-A 3.4113 eV 363.45 nm f=0.0036 <S**2>=0.000

192 -> 194 0.25909

192 -> 195 0.27742

193 -> 194 0.43884

193 -> 195 0.36777

This state for optimization and/or second-order correction.

Total Energy, E(TD-HF/TD-DFT) = -2328.51525415

Copying the excited state density for this state as the 1-particle RhoCI density.

Excited State   2:   Singlet-A   3.4339 eV 361.06 nm f=0.0030 <S**2>=0.000

192 -> 194   0.30318

192 -> 195   -0.28609

193 -> 194   -0.34925

193 -> 195   0.41963

Excited State   3:   Singlet-A   4.0304 eV 307.62 nm f=0.9143 <S**2>=0.000

191 -> 196   0.10234

192 -> 201   -0.11270

192 -> 206   -0.20656

193 -> 196   0.59592

193 -> 198   0.20380

Excited State   4:   Singlet-A   4.3075 eV 287.83 nm f=0.1875 <S**2>=0.000

192 -> 196   -0.16303

192 -> 197   0.24711

192 -> 198   0.19118

192 -> 201   -0.13902

192 -> 202   -0.12840

193 -> 197   0.39252

193 -> 198   0.23635

193 -> 201   -0.14869

193 -> 202   -0.12121

Excited State   5:   Singlet-A   4.3306 eV 286.30 nm f=0.2688 <S**2>=0.000

192 -> 196   0.13410

192 -> 197   0.23091

192 -> 198   -0.25907

192 -> 199   0.12646

192 -> 201 -0.13717

192 -> 202 0.14408

193 -> 196 -0.11203

193 -> 197 -0.23785

193 -> 198 0.34296

193 -> 201 0.10526

193 -> 202 -0.15619

Excited State 6: Singlet-A 4.3589 eV 284.44 nm f=0.0226 <S**2>=0.000

192 -> 196 0.25014

192 -> 198 0.11711

192 -> 200 -0.33986

193 -> 199 0.45195

193 -> 200 -0.12818

193 -> 206 -0.10535

Excited State 7: Singlet-A 4.4102 eV 281.13 nm f=0.0774 <S**2>=0.000

192 -> 197 0.11572

192 -> 199 -0.39539

193 -> 198 0.10390

193 -> 199 0.10037

193 -> 200 0.47138

Excited State 8: Singlet-A 4.4586 eV 278.08 nm f=0.0031 <S**2>=0.000

192 -> 196 0.42042

192 -> 198 0.20659

192 -> 200 0.21245

193 -> 199 -0.19868

193 -> 201 -0.23627

193 -> 203 0.11086

193 -> 206 -0.23579

Excited State 9: Singlet-A 4.5259 eV 273.94 nm f=0.0906 <S**2>=0.000

182 -> 195 0.10581

186 -> 194 0.10186

186 -> 195 0.16424

191 -> 194 0.32937

191 -> 195 0.17924

192 -> 194 -0.19687

192 -> 195 -0.31227

193 -> 194 0.20022

193 -> 195 0.10427

Excited State 10: Singlet-A 4.5395 eV 273.12 nm f=0.0418 <S**2>=0.000

182 -> 194 0.10302

186 -> 194 0.17375

186 -> 195 -0.10869

191 -> 194 -0.17498

191 -> 195 0.32307

192 -> 194 -0.30048

192 -> 195 0.18702

193 -> 194 -0.12647

193 -> 195 0.21809

Excited State 11: Singlet-A 4.8016 eV 258.21 nm f=0.0446 <S**2>=0.000

178 -> 194 -0.10611

178 -> 195 -0.10295

179 -> 194 -0.17003

179 -> 195 -0.15451

185 -> 194 -0.17357

185 -> 195 -0.15939

186 -> 194 0.20173

186 -> 195 0.19655

190 -> 194 -0.11344

190 -> 195 -0.10593

192 -> 194 0.19778

192 -> 195 0.19488

193 -> 194 -0.13445

193 -> 195 -0.12173

193 -> 204 0.11038

Excited State 12: Singlet-A 4.8097 eV 257.78 nm f=0.0025 <S**2>=0.000

191 -> 199 -0.14515

192 -> 194 -0.18643

192 -> 200 -0.13081

192 -> 204 0.24127

192 -> 205 -0.11194

193 -> 195 0.11652

193 -> 203 0.40160

193 -> 204 0.11614

Excited State 13: Singlet-A 4.8185 eV 257.31 nm f=0.0370 <S**2>=0.000

178 -> 195 -0.10471

179 -> 194 -0.15244

179 -> 195 0.16222

180 -> 194 0.11461

180 -> 195 -0.11043

185 -> 194 -0.14556

185 -> 195 0.16173

186 -> 194 -0.17324

186 -> 195 0.18591

192 -> 194 -0.17618

192 -> 195 0.17940

192 -> 203 0.11885

193 -> 194 -0.12583

193 -> 195 0.14186

193 -> 203 -0.14923

193 -> 204 0.11620

Excited State 14:   Singlet-A   4.8545 eV 255.40 nm f=0.0488 <S**2>=0.000

191 -> 200   0.10428

192 -> 195   -0.20405

192 -> 203   0.30458

193 -> 194   0.12802

193 -> 204   0.35798

193 -> 205   -0.17214

Excited State 15:   Singlet-A   4.8849 eV 253.81 nm f=0.1703 <S**2>=0.000

192 -> 197   0.10905

192 -> 201   0.27526

192 -> 202   0.14196

193 -> 197   0.17432

193 -> 198   0.16687

193 -> 201   0.23517

193 -> 202   0.33599

193 -> 206   -0.13903

7.21.3 Computed xyz-coordinates of compound 6d (CAM-B3LYP/6-31+G PCM CH_2Cl_2)**

Input file:

%NProcShared=16

%Chk=gauss.chk

%mem=10GB

#p CAM-B3LYP 6-31+G** pop=full GFInput opt SCRF=(Solvent=Dichloromethane)

Optimized S_0-geometry:

0 1

C 0   -3.527532   -0.598213   -0.205853

C 0   -5.655532   -0.331881   0.964744

C 0   -2.899451   -0.295437   -1.416628

C 0   -1.527844   -0.099548   -1.467106

C 0   -0.736933   -0.184719   -0.312895

C 0   -1.378079   -0.484005   0.892978

C	0	-2.751079	-0.695905	0.948610
C	0	-5.625452	1.028975	1.323002
C	0	-6.294231	1.446686	2.476306
C	0	-7.018562	0.551622	3.254455
C	0	-7.065589	-0.789101	2.884215
C	0	-6.381478	-1.225566	1.757474
N	0	-4.937093	-0.797934	-0.171100
C	0	-5.499358	-1.832256	-0.971300
C	0	-4.897349	2.050034	0.516209
C	0	-4.748755	-2.953697	-1.336497
C	0	-5.313955	-3.949396	-2.128503
C	0	-6.635383	-3.852145	-2.553539
C	0	-7.387241	-2.738807	-2.181678
C	0	-6.825948	-1.732814	-1.404981
C	0	-5.392138	2.517967	-0.712474
C	0	-4.710969	3.491857	-1.450498
C	0	-3.528830	4.025854	-0.967951
C	0	-3.039324	3.585323	0.264545
C	0	-3.716711	2.608670	0.997045
C	0	0.729453	0.032706	-0.371348
C	0	1.407009	0.693402	0.662740
C	0	2.779319	0.886978	0.616519
C	0	3.521272	0.439198	-0.479244
C	0	2.857544	-0.207829	-1.521481
C	0	1.483902	-0.414511	-1.460233
N	0	4.930413	0.645261	-0.512976
C	0	5.429323	1.976296	-0.444843
C	0	5.743015	-0.371828	-1.086909
C	0	4.655926	3.062577	-0.864915
C	0	5.159020	4.357823	-0.779518
C	0	6.441575	4.589572	-0.292223
C	0	7.216622	3.506177	0.118422
C	0	6.716143	2.211854	0.051970

C 0	5.735798	-1.676545	-0.559299
C 0	6.498005	-2.668902	-1.180577
C 0	7.291380	-2.383201	-2.285280
C 0	7.314871	-1.086345	-2.789691
C 0	6.539336	-0.095726	-2.202026
C 0	4.937075	-2.044497	0.645052
C 0	3.792450	-2.825573	0.514515
C 0	3.052916	-3.200214	1.637969
C 0	3.442708	-2.801087	2.919264
C 0	4.588014	-2.038098	3.065707
C 0	5.331603	-1.668669	1.939856
C 0	-6.646847	2.035663	-1.220870
N 0	-7.665684	1.688626	-1.645374
C 0	-1.822412	4.141718	0.789404
N 0	-0.844894	4.591919	1.214373
C 0	6.547108	-0.929008	2.142400
N 0	7.532884	-0.357341	2.342652
C 0	1.875417	-4.007480	1.470786
N 0	0.929230	-4.660316	1.339044
H 0	-3.496275	-0.205224	-2.318205
H 0	-1.068289	0.156858	-2.416237
H 0	-0.794765	-0.585525	1.802484
H 0	-3.220204	-0.945652	1.894384
H 0	-6.261454	2.497096	2.748485
H 0	-7.542945	0.899740	4.137681
H 0	-7.621838	-1.503367	3.482511
H 0	-6.399166	-2.274715	1.484692
H 0	-3.722546	-3.049950	-1.001230
H 0	-4.714862	-4.812182	-2.402548
H 0	-7.074530	-4.631872	-3.166956
H 0	-8.417663	-2.643030	-2.509424
H 0	-7.412883	-0.861917	-1.137948
H 0	-5.118363	3.833365	-2.394766

H 0 -2.995744 4.781208 -1.532634

H 0 -3.315982 2.271952 1.945746

H 0 0.851862 1.070716 1.515390

H 0 3.284615 1.397092 1.429888

H 0 3.417838 -0.563175 -2.379817

H 0 0.996471 -0.944032 -2.272495

H 0 3.659600 2.897255 -1.258121

H 0 4.542119 5.188336 -1.108231

H 0 6.832333 5.599807 -0.230563

H 0 8.216730 3.668295 0.508150

H 0 7.319654 1.378849 0.392685

H 0 6.482305 -3.674095 -0.770867

H 0 7.887188 -3.165135 -2.743381

H 0 7.924911 -0.845982 -3.654352

H 0 6.539993 0.908230 -2.611377

H 0 3.468470 -3.135472 -0.471885

H 0 2.861797 -3.092890 3.785911

H 0 4.918678 -1.733778 4.051744

SCF Done: E(RCAM-B3LYP) = -2328.12336689 A.U. after 3 cycles

Zero-point correction= 0.700247 (Hartree/Particle)

Thermal correction to Energy= 0.746860

Thermal correction to Enthalpy= 0.747805

Thermal correction to Gibbs Free Energy= 0.612364

Sum of electronic and zero-point Energies= -2327.423120

Sum of electronic and thermal Energies= -2327.376507

Sum of electronic and thermal Enthalpies= -2327.375562

Sum of electronic and thermal Free Energies= -2327.511003

7.21.3.1 Computed excitation of compound 6d (CAM-B3LYP/6-31+G PCM CH_2Cl_2)**

Inputfile:

%NProcShared=4

%Chk=gauss.chk

%mem=10GB

#p CAM-B3LYP 6-31+G** geom=check guess=read SCRF=(Solvent=Dichlorometh
ane, correctedLR) TD=(Nstates=15,root=1)

Excitation energies and oscillator strengths:

Excited State 1: Singlet-A 3.3741 eV 367.46 nm f=0.0214 <S**2>=0.000

192 -> 195 -0.36754

193 -> 194 0.58502

This state for optimization and/or second-order correction.

Total Energy, E(TD-HF/TD-DFT) = -2327.99937144

Copying the excited state density for this state as the 1-particle RhoCI density.

Excited State 2: Singlet-A 3.3833 eV 366.46 nm f=0.0002 <S**2>=0.000

192 -> 194 -0.37474

193 -> 195 0.58146

Excited State 3: Singlet-A 4.0077 eV 309.37 nm f=0.9825 <S**2>=0.000

192 -> 207 0.17675

193 -> 196 0.57186

193 -> 198 0.28761

Excited State 4: Singlet-A 4.2865 eV 289.24 nm f=0.0548 <S**2>=0.000

191 -> 195 0.11237

192 -> 194 -0.22981

192 -> 196 -0.20571

192 -> 198 0.25194

192 -> 202 0.16454

193 -> 195 -0.22658

193 -> 197 0.40254

193 -> 198 0.11849

193 -> 201 -0.16462

Excited State 5: Singlet-A 4.2925 eV 288.84 nm f=0.3327 <S**2>=0.000

192 -> 195 0.18199

192 -> 197 0.32761
192 -> 201 -0.17337
193 -> 194 0.19221
193 -> 196 -0.23390
193 -> 197 -0.15245
193 -> 198 0.35641
193 -> 202 0.18906

Excited State 6: Singlet-A 4.3307 eV 286.29 nm f=0.0088 <S**2>=0.000
192 -> 194 -0.23170
192 -> 198 -0.16912
192 -> 200 -0.29746
193 -> 195 -0.17085
193 -> 197 -0.12475
193 -> 199 0.44062
193 -> 201 0.12497

Excited State 7: Singlet-A 4.3626 eV 284.20 nm f=0.0945 <S**2>=0.000
191 -> 194 0.13171
192 -> 195 -0.34610
192 -> 199 -0.29212
193 -> 194 -0.24079
193 -> 198 0.14531
193 -> 200 0.36612

Excited State 8: Singlet-A 4.3681 eV 283.84 nm f=0.0065 <S**2>=0.000
191 -> 195 -0.13747
192 -> 194 0.40131
192 -> 198 0.18014
192 -> 200 -0.21208
193 -> 195 0.20644
193 -> 197 0.17927
193 -> 199 0.29618

193 -> 201 -0.11499

Excited State 9: Singlet-A 4.3886 eV 282.51 nm f=0.0487 <S**2>=0.000

191 -> 194 -0.12664

192 -> 195 0.37167

192 -> 197 -0.12905

192 -> 199 -0.26310

193 -> 194 0.18852

193 -> 198 -0.15797

193 -> 200 0.36206

Excited State 10: Singlet-A 4.4781 eV 276.87 nm f=0.0005 <S**2>=0.000

192 -> 194 -0.17476

192 -> 196 0.49558

192 -> 198 0.19480

193 -> 195 -0.11583

193 -> 201 -0.16552

193 -> 207 0.24878

Excited State 11: Singlet-A 4.7574 eV 260.61 nm f=0.0487 <S**2>=0.000

178 -> 194 0.11783

178 -> 195 -0.10012

179 -> 194 -0.13311

179 -> 195 0.19724

182 -> 194 -0.15219

182 -> 195 0.11840

185 -> 195 0.12772

186 -> 194 -0.20173

186 -> 195 0.16626

187 -> 195 0.13699

191 -> 194 0.11159

191 -> 195 -0.16973

192 -> 194 -0.13898

193 -> 204 0.18774

Excited State 12: Singlet-A 4.7650 eV 260.20 nm f=0.0853 <S**2>=0.000
178 -> 195 -0.13182
179 -> 194 -0.22307
179 -> 195 -0.12503
182 -> 194 0.12053
182 -> 195 0.17217
185 -> 194 -0.16363
186 -> 194 0.13097
186 -> 195 0.20613
187 -> 194 -0.16077
191 -> 194 0.17857
191 -> 195 0.10128
192 -> 194 0.10099
192 -> 195 0.13571
193 -> 204 -0.12991

Excited State 13: Singlet-A 4.7852 eV 259.10 nm f=0.0027 <S**2>=0.000
179 -> 195 -0.10862
191 -> 199 -0.11581
192 -> 200 0.12444
192 -> 202 0.18090
192 -> 205 0.16950
192 -> 206 0.11097
193 -> 197 -0.15124
193 -> 201 -0.19816
193 -> 202 -0.11421
193 -> 204 0.31747
193 -> 205 -0.10820
193 -> 207 -0.17760

Excited State 14: Singlet-A 4.8013 eV 258.23 nm f=0.0690 <S**2>=0.000

192 -> 201 -0.23758

192 -> 204 0.22182

192 -> 207 -0.10322

193 -> 198 -0.17660

193 -> 202 0.29412

193 -> 203 -0.13046

193 -> 204 0.10041

193 -> 205 0.22516

193 -> 206 0.19834

193 -> 208 0.12668

Excited State 15: Singlet-A 4.8586 eV 255.19 nm f=0.0288 <S**2>=0.000

187 -> 195 -0.12380

188 -> 194 0.10943

192 -> 198 0.10056

192 -> 202 -0.28688

193 -> 197 0.23868

193 -> 201 0.35280

193 -> 204 0.19659

193 -> 210 -0.11698

7.22 Quantum chemical calculation data of 6,6'-(([1,1'-biphenyl]-4,4'-diylbis(phenylazanediyl))bis(2,1-phenylene))dipicolinonitrile (7a)

7.22.1 Computed xyz-coordinates of compound 7a (PBE1PBE/6-31+G** PCM CH_2Cl_2)

Input file:

%NProcShared=16

%Chk=gauss.chk

%mem=8GB

#p PBE1PBE 6-31+G** pop=full GFInput opt SCRF=(Solvent=Dichloromethane)

Optimized S_0-geometry:

0 1

N 0	5.408449	0.836519	0.223288
C 0	6.200113	1.498109	-0.738922
C 0	6.045240	0.203264	1.319442
C 0	5.806831	2.733340	-1.273275
C 0	6.605071	3.375528	-2.215361
C 0	7.812675	2.811535	-2.627795
C 0	8.210083	1.587919	-2.088605
C 0	7.410272	0.929466	-1.159243
C 0	5.757734	-1.133387	1.659853
C 0	6.342185	-1.680820	2.807850
C 0	7.235755	-0.948342	3.583532
C 0	7.547251	0.360941	3.218798
C 0	6.945741	0.932818	2.103196
C 0	4.002732	0.896050	0.182844
C 0	3.256298	0.993824	1.364854
C 0	1.867499	1.030263	1.323903
C 0	1.166726	0.982726	0.110177
C 0	1.926959	0.888773	-1.067196
C 0	3.313409	0.838266	-1.037221
C 0	-0.308472	1.026346	0.070359
C 0	-0.992187	1.657157	-0.980826
C 0	-2.379486	1.705583	-1.022379
C 0	-3.142802	1.105770	-0.011135
C 0	-2.472880	0.468463	1.041842
C 0	-1.084999	0.438192	1.080623
N 0	-4.550308	1.152769	-0.028493
C 0	-5.243404	1.375926	1.187529
C 0	-5.273834	1.101560	-1.237021
C 0	-6.427028	1.880310	-1.407392
C 0	-7.151782	1.813836	-2.594927
C 0	-6.735389	0.985477	-3.635894
C 0	-5.585043	0.212204	-3.469197
C 0	-4.863831	0.258829	-2.280974

C	0	-6.284145	0.522029	1.602114
C	0	-7.000327	0.839990	2.761850
C	0	-6.668786	1.949495	3.533780
C	0	-5.610869	2.768181	3.140159
C	0	-4.914276	2.487997	1.969732
C	0	4.880641	-1.990653	0.823574
C	0	-6.622859	-0.721733	0.866440
C	0	3.816484	-2.703608	1.396821
C	0	3.037798	-3.526945	0.594965
C	0	3.333757	-3.621615	-0.760922
C	0	4.412486	-2.872736	-1.233197
N	0	5.175786	-2.076643	-0.476882
C	0	-7.946317	-0.995063	0.488338
C	0	-8.236194	-2.185916	-0.163714
C	0	-7.202175	-3.078073	-0.427979
C	0	-5.921241	-2.712949	-0.011307
N	0	-5.622349	-1.572877	0.620349
C	0	4.765717	-2.938504	-2.630607
N	0	5.035959	-3.003778	-3.757939
C	0	-4.813706	-3.605358	-0.253881
N	0	-3.934533	-4.336288	-0.456237
H	0	4.876895	3.188781	-0.946763
H	0	6.284748	4.332488	-2.618466
H	0	8.435042	3.318655	-3.359038
H	0	9.144114	1.130810	-2.403812
H	0	7.715359	-0.031628	-0.756972
H	0	6.120774	-2.712297	3.069571
H	0	7.692335	-1.400334	4.458754
H	0	8.242523	0.945618	3.814222
H	0	7.157056	1.963264	1.832424
H	0	3.768460	1.053612	2.320751
H	0	1.320879	1.134186	2.257421
H	0	1.423732	0.811556	-2.027340

H 0 3.870464 0.736739 -1.963550

H 0 -0.429536 2.155795 -1.765528

H 0 -2.876065 2.222949 -1.837587

H 0 -3.048902 -0.019750 1.821917

H 0 -0.597175 -0.089652 1.895713

H 0 -6.752234 2.539565 -0.607935

H 0 -8.041939 2.426785 -2.708229

H 0 -7.298370 0.941099 -4.563404

H 0 -5.251686 -0.447144 -4.266100

H 0 -3.981142 -0.360279 -2.153966

H 0 -7.803309 0.180898 3.081599

H 0 -7.225531 2.166156 4.440353

H 0 -5.340560 3.637755 3.732234

H 0 -4.111558 3.140102 1.637753

H 0 3.601424 -2.595006 2.454921

H 0 2.205637 -4.082637 1.015381

H 0 2.755054 -4.246241 -1.432154

H 0 -8.727572 -0.270815 0.694900

H 0 -9.251750 -2.415722 -0.470089

H 0 -7.372744 -4.020013 -0.937228

7.22.1.1 Computed excitation of compound 7a (PBE1PBE/6-31+G** PCM CH_2Cl_2)

Inputfile:

%NProcShared=4

%Chk=gauss.chk

%mem=8GB

#p PBE1PBE 6-31+G** geom=check guess=read SCRF=(Solvent=Dichloromethane, correctedLR) TD=(Nstates=15,root=1)

Excitation energies and oscillator strengths:

Excited State 1: Singlet-A 2.8634 eV 433.00 nm f=0.0109 <S**2>=0.000

180 -> 183 0.20581

181 -> 182 0.20987

181 -> 183 0.63053

This state for optimization and/or second-order correction.

Total Energy, E(TD-HF/TD-DFT) = -2174.35646325

Copying the excited state density for this state as the 1-particle RhoCI density.

Excited State 2: Singlet-A 2.8638 eV 432.94 nm f=0.0119 <S**2>=0.000

180 -> 182 -0.25649

181 -> 182 0.61200

181 -> 183 -0.21625

Excited State 3: Singlet-A 3.1158 eV 397.93 nm f=0.2340 <S**2>=0.000

180 -> 184 -0.27079

181 -> 184 0.63388

Excited State 4: Singlet-A 3.1405 eV 394.79 nm f=0.0241 <S**2>=0.000

180 -> 185 0.24122

181 -> 185 0.64673

Excited State 5: Singlet-A 3.4658 eV 357.73 nm f=0.0021 <S**2>=0.000

180 -> 182 0.53618

180 -> 183 0.37618

181 -> 182 0.22199

181 -> 183 -0.12401

Excited State 6: Singlet-A 3.4673 eV 357.58 nm f=0.0045 <S**2>=0.000

180 -> 182 -0.36573

180 -> 183 0.55337

181 -> 182 -0.15363

181 -> 183 -0.17360

Excited State 7: Singlet-A 3.5318 eV 351.05 nm f=0.9070 <S**2>=0.000

180 -> 184 0.16849

181 -> 186 0.66210

Excited State 8: Singlet-A 3.7702 eV 328.85 nm f=0.0241 <S**2>=0.000

180 -> 184 0.54677

180 -> 186 0.12043

180 -> 187 -0.11491

181 -> 184 0.24041

181 -> 185 0.11619

181 -> 186 -0.13543

181 -> 187 0.24487

Excited State 9: Singlet-A 3.8046 eV 325.88 nm f=0.0420 <S**2>=0.000

180 -> 185 0.58037

180 -> 187 -0.12726

181 -> 185 -0.22591

181 -> 187 0.26506

Excited State 10: Singlet-A 3.8247 eV 324.17 nm f=0.0842 <S**2>=0.000

180 -> 184 -0.25981

180 -> 185 -0.26957

180 -> 187 -0.23596

181 -> 184 -0.14622

181 -> 187 0.49857

Excited State 11: Singlet-A 3.8651 eV 320.78 nm f=0.0805 <S**2>=0.000

180 -> 186 -0.13536

180 -> 188 0.22555

181 -> 188 0.61910

Excited State 12: Singlet-A 4.0544 eV 305.80 nm f=0.1269 <S**2>=0.000

180 -> 188 -0.13704

180 -> 189 -0.21323

181 -> 189 0.61628

Excited State 13:   Singlet-A   4.0718 eV 304.49 nm f=0.0193 <S**2>=0.000

180 -> 186   0.60760

181 -> 190   -0.18420

181 -> 191   -0.15618

181 -> 193   0.10381

Excited State 14:   Singlet-A   4.1190 eV 301.00 nm f=0.0536 <S**2>=0.000

180 -> 186   0.17256

180 -> 189   -0.11066

180 -> 190   0.23972

181 -> 190   0.59699

Excited State 15:   Singlet-A   4.3381 eV 285.80 nm f=0.0451 <S**2>=0.000

180 -> 186   0.10184

180 -> 189   -0.13588

180 -> 192   -0.17307

181 -> 191   0.55888

181 -> 192   -0.12995

181 -> 193   0.20883

181 -> 194   -0.12275

7.22.1.2 Computed emission of compound 7a (PBE1PBE/6-31+G PCM CH_2Cl_2)**

Inputfile:

%NProcShared=16

%Chk=gauss.chk

%mem=10GB

#p PBE1PBE 6-31+G** geom=check guess=read TD=(Nstates=6,root=1)

SCRF=(Solvent=Dichloromethane,ExternalIteration,NonEquilibrium=Save)

--link1--

%NProcShared=16

%Chk=gauss.chk

%mem=10GB

#p PBE1PBE 6-31+G** geom=check guess=read

SCRF=(Solvent=Dichloromethane,NonEquilibrium=Read)

Excitation energies and oscillator strengths:

Excited State 1: Singlet-A 1.2116 eV 1023.30 nm f=0.0186 <S**2>=0.000

180 -> 182 -0.13881

181 -> 182 0.69264

This state for optimization and/or second-order correction.

Total Energy, E(TD-HF/TD-DFT) = -2174.40014499

Copying the excited state density for this state as the 1-particle RhoCI density.

Excited State 2: Singlet-A 1.4986 eV 827.34 nm f=0.0108 <S**2>=0.000

180 -> 183 -0.11780

181 -> 183 0.69665

Excited State 3: Singlet-A 2.1218 eV 584.34 nm f=0.0110 <S**2>=0.000

180 -> 182 0.68165

181 -> 182 0.13978

Excited State 4: Singlet-A 2.4444 eV 507.22 nm f=0.0007 <S**2>=0.000

180 -> 183 0.68928

181 -> 183 0.11645

Excited State 5: Singlet-A 2.6227 eV 472.73 nm f=0.0272 <S**2>=0.000

181 -> 184 -0.36678

181 -> 185 0.59181

Excited State 6: Singlet-A 2.7536 eV 450.26 nm f=0.1849 <S**2>=0.000

180 -> 184 0.13131

181 -> 184 -0.58051

181 -> 185 -0.34665

181 -> 186 0.11764

7.22.2 Computed xyz-coordinates of compound 7a (CAM-B3LYP/6-31+G PCM CH_2Cl_2)**

Inputfile:

%NProcShared=16

%Chk=gauss.chk

%mem=8GB

#p CAM-B3LYP 6-31+G** pop=full GFInput opt SCRF=(Solvent=Dichloromethane)

Optimized S_0-geometry:

```
 0  1
N 0    5.387395    0.855468    0.240076
C 0    6.186377    1.520301   -0.720055
C 0    6.018780    0.226447    1.348514
C 0    5.798225    2.755075   -1.252352
C 0    6.600251    3.398706   -2.188339
C 0    7.807062    2.834979   -2.596492
C 0    8.199612    1.610936   -2.059667
C 0    7.395294    0.952388   -1.136369
C 0    5.764362   -1.118883    1.664330
C 0    6.343645   -1.666845    2.811892
C 0    7.199385   -0.920749    3.614017
C 0    7.476149    0.401273    3.276583
C 0    6.878881    0.970285    2.158988
C 0    3.976660    0.916750    0.193460
C 0    3.227350    1.046150    1.366681
C 0    1.839156    1.078946    1.319706
C 0    1.146102    0.996565    0.107523
C 0    1.908203    0.872808   -1.062056
C 0    3.293682    0.825218   -1.024806
C 0   -0.334996    1.034734    0.061009
C 0   -1.014124    1.668227   -0.986836
C 0   -2.400826    1.710706   -1.033292
C 0   -3.164253    1.101639   -0.031883
```

C 0	-2.497492	0.461726	1.017484
C 0	-1.110405	0.437510	1.062136
N 0	-4.577192	1.142766	-0.056305
C 0	-5.280330	1.385178	1.156086
C 0	-5.294025	1.089106	-1.274011
C 0	-6.425752	1.888148	-1.468265
C 0	-7.140952	1.817569	-2.660077
C 0	-6.734197	0.963630	-3.681051
C 0	-5.604068	0.169429	-3.490976
C 0	-4.894395	0.221228	-2.297866
C 0	-6.301208	0.522895	1.589954
C 0	-7.019083	0.848762	2.743698
C 0	-6.709207	1.980106	3.489651
C 0	-5.672692	2.811842	3.074875
C 0	-4.974197	2.519778	1.910221
C 0	4.917508	-1.987637	0.801714
C 0	-6.624879	-0.741572	0.874465
C 0	3.829793	-2.688768	1.337492
C 0	3.082158	-3.518017	0.516253
C 0	3.432943	-3.631148	-0.823835
C 0	4.530200	-2.895400	-1.261177
N 0	5.262659	-2.093671	-0.483028
C 0	-7.933419	-1.015964	0.457030
C 0	-8.209586	-2.219499	-0.172565
C 0	-7.175242	-3.125302	-0.375885
C 0	-5.909890	-2.761301	0.074770
N 0	-5.627088	-1.607531	0.685734
C 0	4.943926	-2.981167	-2.645678
N 0	5.261929	-3.062357	-3.754562
C 0	-4.797261	-3.669991	-0.103423
N 0	-3.919732	-4.408462	-0.251502
H 0	4.868526	3.210847	-0.930429
H 0	6.282676	4.355706	-2.590715
H 0	8.432856	3.342687	-3.322917

H 0 9.133723 1.153962 -2.371237
H 0 7.696368 -0.009519 -0.737064
H 0 6.145094 -2.705666 3.057134
H 0 7.652386 -1.371694 4.490431
H 0 8.141754 0.996576 3.893110
H 0 7.065217 2.008887 1.906651
H 0 3.734064 1.130441 2.321930
H 0 1.288445 1.202565 2.246852
H 0 1.408849 0.774017 -2.020741
H 0 3.853662 0.702166 -1.945132
H 0 -0.450551 2.169747 -1.767234
H 0 -2.894953 2.229122 -1.847418
H 0 -3.072936 -0.032561 1.792054
H 0 -0.623596 -0.088677 1.877161
H 0 -6.744343 2.566978 -0.684732
H 0 -8.016113 2.446330 -2.791757
H 0 -7.289299 0.915412 -4.611942
H 0 -5.277923 -0.509129 -4.273134
H 0 -4.027218 -0.413210 -2.152653
H 0 -7.809553 0.183748 3.077762
H 0 -7.267796 2.204731 4.391967
H 0 -5.419304 3.698697 3.646546
H 0 -4.185698 3.179431 1.563518
H 0 3.573869 -2.567704 2.383726
H 0 2.232531 -4.065221 0.909164
H 0 2.880146 -4.262333 -1.508896
H 0 -8.714935 -0.282468 0.618181
H 0 -9.214774 -2.449793 -0.507630
H 0 -7.336305 -4.078342 -0.864904

SCF Done: E(RCAM-B3LYP) = -2175.79497328 A.U. after 2 cycles

Zero-point correction= 0.678890 (Hartree/Particle)

Thermal correction to Energy= 0.721635

Thermal correction to Enthalpy= 0.722580

Thermal correction to Gibbs Free Energy= 0.595533

Sum of electronic and zero-point Energies= -2175.116084

Sum of electronic and thermal Energies= -2175.073338

Sum of electronic and thermal Enthalpies= -2175.072394

Sum of electronic and thermal Free Energies= -2175.199441

7.22.2.1 Computed excitation of compound 7a (CAM-B3LYP/6-31+G** PCM CH_2Cl_2)

Inputfile:

%NProcShared=4

%Chk=gauss.chk

%mem=8GB

#p CAM-B3LYP 6-31+G** geom=check guess=read SCRF=(Solvent=Dichloromethane, correctedLR) TD=(Nstates=15,root=1)

Excitation energies and oscillator strengths:

Excited State 1: Singlet-A 3.7258 eV 332.77 nm f=0.4123 <S**2>=0.000

180 -> 182 -0.10293

180 -> 184 0.32562

181 -> 182 -0.10844

181 -> 183 0.17443

181 -> 184 0.46994

181 -> 185 -0.16455

181 -> 186 0.20297

This state for optimization and/or second-order correction.

Total Energy, E(TD-HF/TD-DFT) = -2175.65805263

Copying the excited state density for this state as the 1-particle RhoCI density.

Excited State 2: Singlet-A 3.7410 eV 331.42 nm f=0.0205 <S**2>=0.000

180 -> 183 0.22582

180 -> 185 -0.27612

181 -> 182 -0.11060

181 -> 183 -0.38708

181 -> 184 0.22900

181 -> 185 0.34863

Excited State 3: Singlet-A 3.7928 eV 326.89 nm f=0.4344 <S**2>=0.000
180 -> 182 0.33773
181 -> 182 0.50316
181 -> 184 0.11545
181 -> 185 0.12067
181 -> 186 0.26699

Excited State 4: Singlet-A 3.8668 eV 320.63 nm f=0.0624 <S**2>=0.000
180 -> 183 -0.23144
180 -> 185 -0.23329
181 -> 183 0.43691
181 -> 185 0.38462

Excited State 5: Singlet-A 4.0438 eV 306.60 nm f=0.4644 <S**2>=0.000
180 -> 182 -0.17160
180 -> 184 -0.24475
180 -> 193 -0.10004
181 -> 182 -0.20149
181 -> 184 -0.10616
181 -> 186 0.52161

Excited State 6: Singlet-A 4.2587 eV 291.13 nm f=0.1207 <S**2>=0.000
180 -> 187 0.39048
181 -> 187 0.51531

Excited State 7: Singlet-A 4.3012 eV 288.26 nm f=0.0671 <S**2>=0.000
180 -> 186 0.17117
180 -> 187 0.13126
180 -> 188 -0.28057
181 -> 188 0.53035

Excited State 8: Singlet-A 4.4688 eV 277.44 nm f=0.1426 <S**2>=0.000
180 -> 184 0.14653

180 -> 186 0.41425

180 -> 188 0.21915

181 -> 189 0.19668

181 -> 190 0.17071

181 -> 191 -0.11086

181 -> 192 -0.10246

181 -> 193 -0.20731

181 -> 195 0.13234

Excited State 9: Singlet-A 4.5753 eV 270.99 nm f=0.0683 <S**2>=0.000

180 -> 186 -0.24453

180 -> 188 0.10425

180 -> 189 0.15737

180 -> 191 -0.10301

181 -> 189 0.26733

181 -> 190 0.28176

181 -> 193 0.27708

181 -> 197 0.10599

Excited State 10: Singlet-A 4.6298 eV 267.79 nm f=0.0363 <S**2>=0.000

180 -> 190 -0.24011

180 -> 191 -0.20548

180 -> 192 0.10737

181 -> 185 0.11971

181 -> 190 0.16501

181 -> 191 0.41605

181 -> 192 -0.12064

181 -> 194 -0.11791

181 -> 195 0.14211

Excited State 11: Singlet-A 4.7874 eV 258.98 nm f=0.0410 <S**2>=0.000

180 -> 183 0.16527

180 -> 189 -0.12774

180 -> 191 0.17354

180 -> 201 -0.19120
181 -> 190 -0.13378
181 -> 195 0.19562
181 -> 197 0.22334
181 -> 198 0.19243
181 -> 199 -0.12665
181 -> 201 0.16142

Excited State 12: Singlet-A 4.8394 eV 256.20 nm f=0.0041 <S**2>=0.000
180 -> 183 0.23961
180 -> 194 0.10804
180 -> 196 -0.10774
180 -> 197 -0.13565
180 -> 198 -0.11248
180 -> 199 0.13772
181 -> 183 0.13965
181 -> 196 -0.17700
181 -> 197 -0.12237
181 -> 199 0.16963
181 -> 201 0.26675

Excited State 13: Singlet-A 4.8500 eV 255.64 nm f=0.0356 <S**2>=0.000
167 -> 183 0.10263
177 -> 183 -0.10754
179 -> 183 -0.14554
180 -> 183 0.44094
180 -> 197 0.10773
181 -> 183 0.26671
181 -> 196 0.11137
181 -> 201 -0.21872

Excited State 14: Singlet-A 4.9513 eV 250.41 nm f=0.0262 <S**2>=0.000
166 -> 182 0.32944
167 -> 182 -0.17997

180 -> 182 0.24397

180 -> 193 0.12568

180 -> 194 0.12602

181 -> 182 -0.22623

181 -> 193 0.11507

181 -> 194 0.19888

Excited State 15: Singlet-A 4.9653 eV 249.70 nm f=0.0059 <S**2>=0.000

166 -> 182 -0.23028

167 -> 182 0.12330

180 -> 182 -0.19796

180 -> 193 0.18348

181 -> 182 0.16593

181 -> 185 0.11374

181 -> 194 0.25435

181 -> 195 -0.10602

181 -> 199 -0.13836

7.23 Quantum chemical calculation data of 6,6'-(([1,1'-biphenyl]-4,4'-diylbis(phenylazanediyl))bis([1,1'-biphenyl]-2',4-diyl))dipicolino-nitrile (7b)

7.23.1 Computed xyz-coordinates of compound 7b (PBE1PBE/6-31+G** PCM CH_2Cl_2)

Input file:

%NProcShared=16

%Chk=gauss.chk

%mem=10GB

#p PBE1PBE 6-31+G** pop=full GFInput opt SCRF=(Solvent=Dichloromethane)

Optimized S_0-geometry:

0 1

N 0 -6.508623 1.012913 0.030585

C 0 -7.358861 1.141226 1.147158

C 0 -7.087009 0.803293 -1.252060

C 0 -7.083764 2.053688 2.176868

C 0	-7.938908	2.159218	3.269846
C 0	-9.089796	1.375651	3.355214
C 0	-9.374075	0.478632	2.325857
C 0	-8.518475	0.355220	1.235361
C 0	-6.666562	-0.261064	-2.076011
C 0	-7.225668	-0.364414	-3.358314
C 0	-8.195413	0.525857	-3.807637
C 0	-8.624014	1.557099	-2.972454
C 0	-8.062748	1.696101	-1.708474
C 0	-5.129225	1.294752	0.113842
C 0	-4.476295	1.979443	-0.918603
C 0	-3.110443	2.229151	-0.849322
C 0	-2.343894	1.818664	0.250423
C 0	-3.011633	1.135886	1.280406
C 0	-4.371963	0.870572	1.214999
C 0	-0.894092	2.087749	0.322201
C 0	-0.258797	2.356625	1.544346
C 0	1.104028	2.613343	1.619484
C 0	1.898615	2.597790	0.463633
C 0	1.273694	2.332519	-0.764514
C 0	-0.091768	2.088164	-0.828750
N 0	3.281298	2.856799	0.509049
C 0	3.866946	3.620534	-0.538999
C 0	4.050752	2.576360	1.659391
C 0	5.042928	3.469209	2.084593
C 0	5.819503	3.177752	3.203869
C 0	5.613389	2.003368	3.925123
C 0	4.623245	1.113681	3.504343
C 0	3.854442	1.388228	2.378684
C 0	4.998862	3.152939	-1.237632
C 0	5.567112	3.989287	-2.209901
C 0	5.021544	5.231773	-2.515464
C 0	3.885415	5.671332	-1.836684

C 0	3.322485	4.871415	-0.849187
C 0	-5.678720	-1.276733	-1.640241
C 0	5.599751	1.819206	-0.998909
C 0	-5.798800	-1.938319	-0.409791
C 0	-4.896198	-2.925180	-0.039201
C 0	-3.840721	-3.289975	-0.886239
C 0	-3.714526	-2.627734	-2.113449
C 0	-4.619496	-1.638622	-2.482400
C 0	6.987115	1.685523	-0.849972
C 0	7.573279	0.437978	-0.677331
C 0	6.789049	-0.721540	-0.640885
C 0	5.402341	-0.591944	-0.797909
C 0	4.818520	0.656319	-0.972270
C 0	-2.890892	-4.347157	-0.472946
C 0	7.427345	-2.042067	-0.442550
N 0	-2.777353	-4.574889	0.841246
C 0	-1.929185	-5.525369	1.244801
C 0	-1.144146	-6.302244	0.393404
C 0	-1.274123	-6.069188	-0.972883
C 0	-2.154183	-5.091710	-1.411738
N 0	8.717632	-2.145077	-0.783581
C 0	9.322459	-3.324239	-0.611858
C 0	8.704617	-4.466472	-0.103552
C 0	7.365794	-4.351077	0.259895
C 0	6.722579	-3.133609	0.096088
C 0	10.712836	-3.377995	-0.994990
N 0	11.831868	-3.437740	-1.298533
C 0	-1.846385	-5.728912	2.671152
N 0	-1.767408	-5.905753	3.815894
H 0	-6.201181	2.682522	2.117871
H 0	-7.706907	2.873421	4.055326
H 0	-9.755666	1.465220	4.208182
H 0	-10.265555	-0.140903	2.372384

H	0	-8.752987	-0.347377	0.440956
H	0	-6.914738	-1.187002	-3.996762
H	0	-8.621164	0.408051	-4.799716
H	0	-9.379927	2.260744	-3.308909
H	0	-8.367331	2.511411	-1.058337
H	0	-5.043848	2.325997	-1.777102
H	0	-2.640681	2.786394	-1.655488
H	0	-2.448912	0.765813	2.133299
H	0	-4.851045	0.313772	2.014388
H	0	-0.847508	2.404838	2.456606
H	0	1.553468	2.842317	2.580448
H	0	1.863891	2.317207	-1.676249
H	0	-0.535211	1.859794	-1.794218
H	0	5.205932	4.391914	1.535872
H	0	6.584321	3.883269	3.517073
H	0	6.216544	1.781255	4.800362
H	0	4.456367	0.187542	4.047794
H	0	3.100669	0.679965	2.048807
H	0	6.434336	3.633630	-2.759720
H	0	5.475497	5.848120	-3.285788
H	0	3.448464	6.639731	-2.062514
H	0	2.453861	5.213328	-0.293523
H	0	-6.615282	-1.686062	0.259926
H	0	-5.007183	-3.433278	0.913282
H	0	-2.889161	-2.856070	-2.781304
H	0	-4.487331	-1.122531	-3.429431
H	0	7.613452	2.573543	-0.851308
H	0	8.648258	0.352786	-0.555415
H	0	4.765828	-1.471808	-0.813904
H	0	3.743433	0.725376	-1.106264
H	0	-0.471781	-7.055652	0.787919
H	0	-0.700880	-6.653367	-1.685687
H	0	-2.286421	-4.919391	-2.473958

H 0   9.252991   -5.395225   0.006186

H 0   6.835608   -5.201569   0.676533

H 0   5.688675   -3.021394   0.402593

SCF Done: E(RPBE1PBE) = -2636.06409929   A.U. after   2 cycles

Zero-point correction=   0.838395 (Hartree/Particle)

Thermal correction to Energy=   0.890904

Thermal correction to Enthalpy=   0.891848

Thermal correction to Gibbs Free Energy=   0.740827

Sum of electronic and zero-point Energies=   -2635.225704

Sum of electronic and thermal Energies=   -2635.173195

Sum of electronic and thermal Enthalpies=   -2635.172251

Sum of electronic and thermal Free Energies=   -2635.323272

7.23.2 Computed excitation of compound 7b (PBE1PBE/6-31+G PCM CH_2Cl_2)**

Inputfile:

%NProcShared=16

%Chk=gauss.chk

%mem=8GB

#p PBE1PBE 6-31+G** geom=check guess=read SCRF=(Solvent=Dichloromethane, correctedLR) TD=(Nstates=15,root=1)

Excitation energies and oscillator strengths:

Excited State   1:   Singlet-A   2.9066 eV 426.57 nm f=0.0339 <S**2>=0.000

220 -> 224   0.13514

221 -> 222   0.33037

221 -> 224   0.53991

221 -> 225   0.24846

This state for optimization and/or second-order correction.

Total Energy, E(TD-HF/TD-DFT) = -2635.95728500

Copying the excited state density for this state as the 1-particle RhoCI density.

Excited State   2:   Singlet-A   2.9396 eV 421.78 nm f=0.1528 <S**2>=0.000

220 -> 224 0.10755
220 -> 225 -0.18130
221 -> 223 0.21123
221 -> 224 -0.27577
221 -> 225 0.55863

Excited State 3: Singlet-A 2.9797 eV 416.10 nm f=0.0023 <S**2>=0.000
221 -> 222 0.62005
221 -> 224 -0.28167
221 -> 225 -0.17444

Excited State 4: Singlet-A 3.0554 eV 405.78 nm f=0.0008 <S**2>=0.000
220 -> 223 -0.11843
221 -> 223 0.66039
221 -> 224 0.10562
221 -> 225 -0.18475

Excited State 5: Singlet-A 3.4354 eV 360.91 nm f=0.4678 <S**2>=0.000
220 -> 222 0.30776
220 -> 224 0.30279
220 -> 225 0.21254
221 -> 225 -0.10052
221 -> 226 -0.47203

Excited State 6: Singlet-A 3.4651 eV 357.81 nm f=0.4710 <S**2>=0.000
220 -> 222 0.50819
220 -> 224 0.16011
221 -> 226 0.43969

Excited State 7: Singlet-A 3.4941 eV 354.83 nm f=0.1418 <S**2>=0.000
220 -> 222 -0.37202
220 -> 224 0.50831
220 -> 225 0.18469

221 -> 224 -0.10477

221 -> 226 0.20449

Excited State 8: Singlet-A 3.5411 eV 350.13 nm f=0.0196 <S**2>=0.000

220 -> 223 0.40462

220 -> 224 -0.18634

220 -> 225 0.47968

221 -> 224 -0.10468

221 -> 225 0.17656

Excited State 9: Singlet-A 3.5806 eV 346.27 nm f=0.0053 <S**2>=0.000

220 -> 223 0.56045

220 -> 224 0.17558

220 -> 225 -0.34753

221 -> 225 -0.11077

Excited State 10: Singlet-A 3.7383 eV 331.66 nm f=0.0365 <S**2>=0.000

220 -> 228 0.21386

221 -> 227 0.55963

221 -> 228 -0.24546

221 -> 229 0.12621

221 -> 230 -0.15451

Excited State 11: Singlet-A 3.7847 eV 327.60 nm f=0.0296 <S**2>=0.000

220 -> 227 0.23362

221 -> 227 0.22993

221 -> 228 0.51357

221 -> 229 0.22495

221 -> 230 0.17387

Excited State 12: Singlet-A 3.8994 eV 317.95 nm f=0.1485 <S**2>=0.000

220 -> 225 -0.12057

220 -> 226 -0.14486

220 -> 229 -0.10939

220 -> 230 -0.21258

221 -> 227 0.17258

221 -> 229 -0.27600

221 -> 230 0.50552

Excited State 13: Singlet-A 3.9086 eV 317.21 nm f=0.0544 <S**2>=0.000

220 -> 226 0.14104

220 -> 229 0.19988

220 -> 230 -0.12144

221 -> 227 -0.13814

221 -> 228 -0.24658

221 -> 229 0.45697

221 -> 230 0.29065

Excited State 14: Singlet-A 4.0091 eV 309.26 nm f=0.0582 <S**2>=0.000

220 -> 226 0.61036

221 -> 229 -0.20920

221 -> 231 -0.16560

221 -> 235 -0.12508

Excited State 15: Singlet-A 4.1027 eV 302.20 nm f=0.1197 <S**2>=0.000

218 -> 223 -0.40195

218 -> 224 -0.14778

218 -> 225 0.21109

219 -> 223 0.41404

219 -> 225 -0.24042

7.24 Quantum chemical calculation data of 6,6'-(((2,2'-dimethyl-[1,1'-biphenyl]-4,4'-diyl)bis(phenylazanediyl))bis(2,1-phenylene))dipicolinonitrile (7c)

7.24.1 Computed xyz-coordinates of compound 7c (PBE1PBE/6-31+G** PCM CH_2Cl_2)

Input file:

```
 %NProcShared=16
 %Chk=gauss.chk
 %mem=10GB
#p PBE1PBE 6-31+G** pop=full GFInput opt SCRF=(Solvent=Dichloromethane)
```

Optimized S_0-geometry:

```
  0  1
N 0    5.251519   -0.489973   -0.985229
C 0    5.979910   -1.696577   -0.934191
C 0    5.895136    0.679980   -1.457439
C 0    5.398684   -2.911456   -1.325002
C 0    6.136571   -4.090561   -1.274546
C 0    7.466437   -4.080800   -0.852867
C 0    8.049241   -2.871366   -0.473960
C 0    7.314386   -1.689905   -0.504314
C 0    5.840486    1.889737   -0.736526
C 0    6.414310    3.038042   -1.295214
C 0    7.077351    2.994529   -2.517773
C 0    7.162042    1.786163   -3.208301
C 0    6.564397    0.644677   -2.685981
C 0    3.865979   -0.457498   -0.722036
C 0    3.013466    0.350977   -1.480401
C 0    1.652422    0.381735   -1.190575
C 0    1.100451   -0.389403   -0.164711
C 0    1.954684   -1.210731    0.599539
C 0    3.320026   -1.223539    0.316574
C 0   -0.366240   -0.339192    0.098514
```

C	0	-0.913143	0.571711	1.023994
C	0	-2.292211	0.581805	1.237000
C	0	-3.151947	-0.275247	0.538366
C	0	-2.604854	-1.171722	-0.384979
C	0	-1.229066	-1.200453	-0.585950
N	0	-4.544280	-0.256964	0.769585
C	0	-5.243081	-1.487687	0.832216
C	0	-5.226398	0.941317	1.058582
C	0	-6.268066	0.957439	1.996967
C	0	-6.954857	2.138782	2.266936
C	0	-6.609245	3.325066	1.621725
C	0	-5.569221	3.312098	0.690307
C	0	-4.888626	2.134182	0.401383
C	0	-6.406655	-1.708500	0.068905
C	0	-7.112019	-2.905668	0.239075
C	0	-6.657403	-3.895785	1.104738
C	0	-5.482864	-3.688356	1.827164
C	0	-4.791434	-2.488898	1.699024
C	0	5.224171	1.984486	0.610327
C	0	-6.893538	-0.727413	-0.932851
C	0	4.289413	2.990358	0.901537
C	0	3.765699	3.083770	2.183623
C	0	4.179128	2.170910	3.148449
C	0	5.110305	1.207547	2.758036
N	0	5.630334	1.104744	1.530524
C	0	-8.232024	-0.305408	-0.930990
C	0	-8.668853	0.580239	-1.906638
C	0	-7.762888	1.030960	-2.861137
C	0	-6.454348	0.553446	-2.775241
N	0	-6.015784	-0.302900	-1.846671
C	0	1.413887	-2.063588	1.714460
C	0	-0.032166	1.524945	1.784274
C	0	5.575475	0.238554	3.720725

N	0	5.937474	-0.532849	4.509464
C	0	-5.475261	0.987285	-3.742277
N	0	-4.701253	1.348446	-4.528823
H	0	4.369161	-2.928349	-1.669254
H	0	5.669295	-5.022281	-1.581967
H	0	8.039995	-5.002323	-0.820166
H	0	9.081752	-2.845792	-0.136216
H	0	7.768487	-0.754806	-0.191261
H	0	6.372879	3.970359	-0.737919
H	0	7.531896	3.893970	-2.921724
H	0	7.674756	1.735396	-4.164539
H	0	6.596764	-0.291960	-3.234961
H	0	3.411447	0.949799	-2.294195
H	0	0.997559	1.012200	-1.787269
H	0	3.979996	-1.843286	0.917439
H	0	-2.709301	1.274068	1.963410
H	0	-3.255649	-1.836854	-0.943822
H	0	-0.813022	-1.900402	-1.306520
H	0	-6.536794	0.041991	2.515914
H	0	-7.758146	2.129838	2.998831
H	0	-7.141892	4.246050	1.839195
H	0	-5.292991	4.225142	0.169624
H	0	-4.093915	2.130596	-0.338166
H	0	-8.009529	-3.075385	-0.350185
H	0	-7.210061	-4.824858	1.205616
H	0	-5.115004	-4.451467	2.507007
H	0	-3.894413	-2.306376	2.283641
H	0	3.974174	3.676539	0.122223
H	0	3.039117	3.851842	2.429485
H	0	3.799327	2.196806	4.163791
H	0	-8.909355	-0.662470	-0.162110
H	0	-9.698605	0.922899	-1.921585
H	0	-8.050160	1.725736	-3.642433

H 0   0.708447   -2.810330   1.333300

H 0   2.219611   -2.589904   2.232525

H 0   0.867506   -1.464422   2.451218

H 0   0.463456   2.228950   1.106421

H 0   -0.611983   2.101917   2.509391

H 0   0.763244   0.997695   2.322058

SCF Done: E(RPBE1PBE) = -2253.00025415   A.U. after   1 cycles

Zero-point correction=   0.730106 (Hartree/Particle)

Thermal correction to Energy=   0.776923

Thermal correction to Enthalpy=   0.777867

Thermal correction to Gibbs Free Energy=   0.641903

Sum of electronic and zero-point Energies=   -2252.270148

Sum of electronic and thermal Energies=   -2252.223331

Sum of electronic and thermal Enthalpies=   -2252.222387

Sum of electronic and thermal Free Energies=   -2252.358351

7.24.2 Computed excitation of compound 7c (PBE1PBE/6-31+G** PCM CH_2Cl_2)

Inputfile:

%NProcShared=4

%Chk=gauss.chk

%mem=8GB

#p PBE1PBE 6-31+G** geom=check guess=read SCRF=(Solvent=Dichloromethane, correctedLR) TD=(Nstates=15,root=1)

Excitation energies and oscillator strengths:

Excited State   1:   Singlet-A   2.8765 eV 431.03 nm f=0.0068 <S**2>=0.000

189 -> 191   0.69441

189 -> 193   -0.12284

This state for optimization and/or second-order correction.

Total Energy, E(TD-HF/TD-DFT) = -2252.89454646

Copying the excited state density for this state as the 1-particle RhoCI density.

Excited State 2: Singlet-A 2.8920 eV 428.71 nm f=0.0092 <S**2>=0.000

188 -> 190 0.69331

188 -> 192 -0.12770

Excited State 3: Singlet-A 3.1155 eV 397.96 nm f=0.0483 <S**2>=0.000

189 -> 191 0.12323

189 -> 193 0.69110

Excited State 4: Singlet-A 3.1314 eV 395.94 nm f=0.0794 <S**2>=0.000

188 -> 190 0.12783

188 -> 192 0.68957

Excited State 5: Singlet-A 3.2770 eV 378.35 nm f=0.0000 <S**2>=0.000

188 -> 191 0.70577

Excited State 6: Singlet-A 3.3196 eV 373.49 nm f=0.0000 <S**2>=0.000

189 -> 190 0.70620

Excited State 7: Singlet-A 3.6211 eV 342.40 nm f=0.0002 <S**2>=0.000

188 -> 193 0.70454

Excited State 8: Singlet-A 3.6486 eV 339.82 nm f=0.0003 <S**2>=0.000

189 -> 192 0.70322

Excited State 9: Singlet-A 3.8386 eV 322.99 nm f=0.2279 <S**2>=0.000

188 -> 194 -0.16232

189 -> 194 -0.25750

189 -> 195 0.61293

Excited State 10: Singlet-A 3.8512 eV 321.94 nm f=0.0696 <S**2>=0.000

188 -> 194 0.63518

188 -> 195 0.21909

189 -> 195 0.16285

Excited State 11: Singlet-A 4.0521 eV 305.97 nm f=0.7833 <S**2>=0.000
188 -> 196 -0.46564
188 -> 198 -0.10367
189 -> 197 0.47734
189 -> 198 -0.16236

Excited State 12: Singlet-A 4.1453 eV 299.10 nm f=0.0088 <S**2>=0.000
188 -> 196 -0.46135
188 -> 198 0.14599
188 -> 199 0.14896
189 -> 197 -0.42583
189 -> 199 0.15645

Excited State 13: Singlet-A 4.1674 eV 297.51 nm f=0.1536 <S**2>=0.000
188 -> 195 -0.18191
188 -> 197 -0.16640
188 -> 198 -0.38992
188 -> 199 -0.24271
189 -> 197 -0.18232
189 -> 198 -0.24900
189 -> 199 0.31276

Excited State 14: Singlet-A 4.1778 eV 296.77 nm f=0.0089 <S**2>=0.000
188 -> 195 0.13778
188 -> 196 0.19317
188 -> 197 0.10325
188 -> 198 0.28867
188 -> 199 0.15142
189 -> 197 0.12160
189 -> 198 -0.37872
189 -> 199 0.34779

Excited State 15: Singlet-A 4.3561 eV 284.62 nm f=0.0025 <S**2>=0.000

188 -> 194 -0.20583

188 -> 195 0.55428

188 -> 197 0.18792

188 -> 198 -0.23187

188 -> 199 -0.15905

188 -> 202 0.10229

8 Solvatochromism

Table S15. Overview of the utilized values for the Lippert-Mataga plot of compound **6d**.

solvent	refractive index[14] n_D^{20}	permit-tivity[14] ε_r	orientation polarizability Δf[a]	$\Delta\tilde{\nu}_s$ [cm^{-1}][b]	onsager radius a[c] [Å]	$\Delta f/a^3$ [10^{56} C^{-2} cm^{-3}]
cyclohexane	1.4262	2.02	-0.00164972	5300	6.52	-0.0053864
toluene	1.4969	2.38	0.01323509	6100	7.12	0.0331829
1,4-dioxane	1.4224	2.21	0.020486267	7100	7.37	0.04631139
ethyl acetate	1.3724	6.02	0.199635043	7500	7.17	0.49012488
dichloro-methane	1.4242	8.93	0.217103254	8100	7.33	0.49886363

[a] $\Delta f = \frac{\varepsilon_r - 1}{2\,\varepsilon_r + 1} - \frac{n^2 - 1}{2\,n^2 + 1}$. [b] $\Delta\tilde{\nu}_s = \frac{1}{\lambda_{max,\ Abs}} - \frac{1}{\lambda_{max,\ Em}}$ recorded in different solvents (see Table 3). [c] TD-DFT calculations of the Onsager radius a (Gaussian 16, PBE1PBE/6-31+G** dipole, a_0 = 7.04 Å[d], volume) with different solvents. [d] TD-DFT calculated Onsager radius a_0 of the gas phase geometry (Gaussian 16, PBE1PBE/6-31+G**).

The change in dipole moment can be calculated using the Lippert-Mataga equation 1.[15]

$$\tilde{\nu}_{abs} - \tilde{\nu}_{abs} = \Delta\tilde{\nu}_s = \frac{2\,\Delta f}{4\,\pi\,\varepsilon_0\,h\,c\,a^3}\,(\mu_{AZ} - \mu_{GZ})^2 + const. \qquad (1)$$

Considering the Onsager radius a of the solvents assuming a spherical dipole into the correlation, the plot of the Stokes shift $\Delta\tilde{\nu}_s$ against the orientation polarizability Δf, the dipole moment change $\Delta\mu$ can be determined using the slope m (equation 2).[15]

$$\Delta\mu = \mu_{AZ} - \mu_{GZ} = \sqrt{m} = \sqrt{3.64 \times 10^{-57} (Cm)^2} = 6.033 \times 10^{-29}\,Cm = 18.1\,D \qquad (2)$$

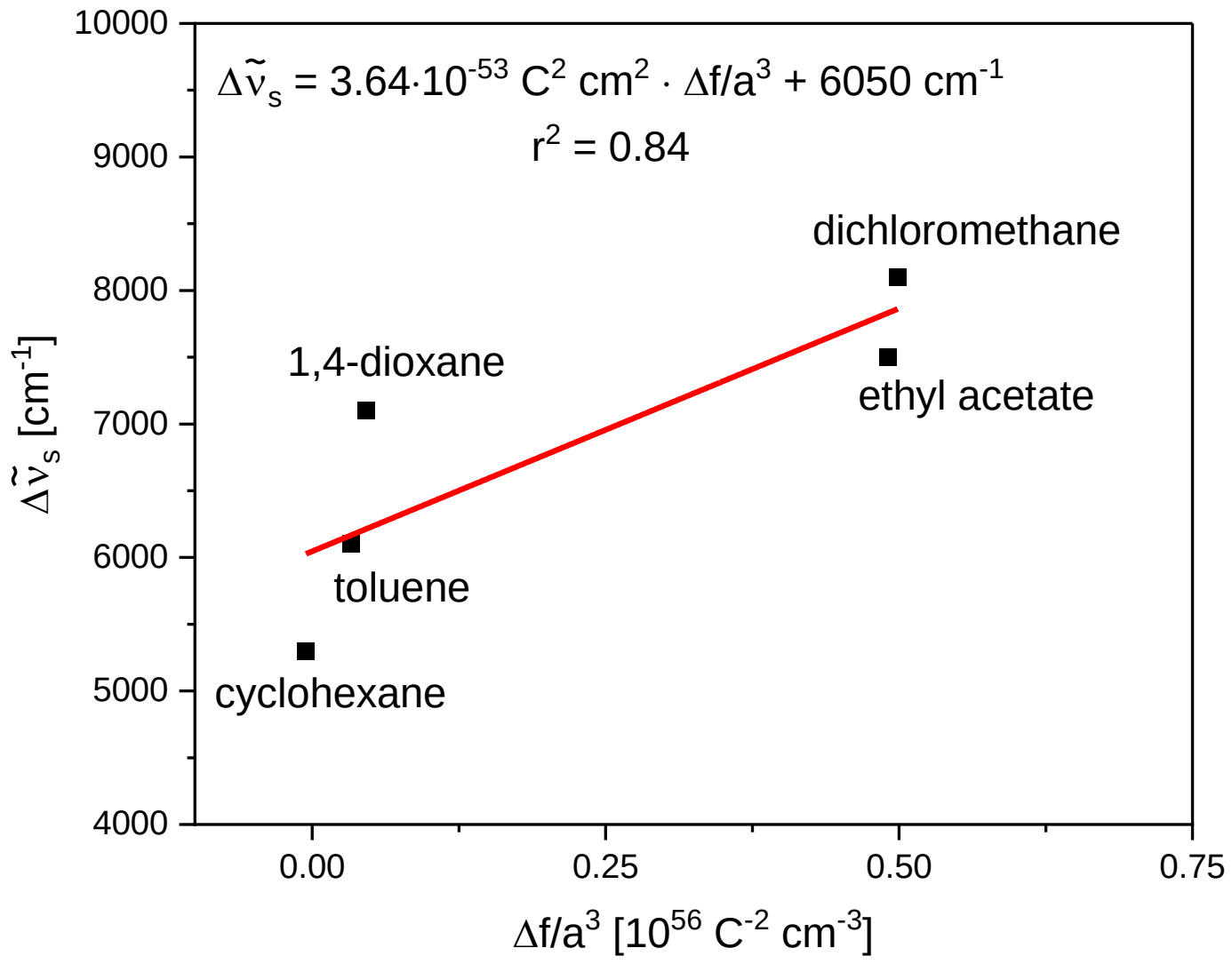

Figure S122. Lippert-Mataga plot for compound **6d** ($\Delta\tilde{\nu}_s$ = 3.64·10^{-53} C^2 cm^2 · $\Delta f/a^3$ + 6050 cm^{-1}, r^2 = 0.84).

The ground state geometries of compound **6d** in different solvents optimized by DFT calculations with the PBE1PBE functional and the 6-31+G** basis set in the program package Gaussian 16[6]. The minima structures were confirmed by analytical frequency analysis (Nimag = 0). The keyword volume requests the computation of the molecule volume in angstroms (≈ Onsager-Radius *a*) and the keyword SCRF requests the computation in the presence of a solvent.

8.1 Quantum chemical calculation data of 2',2'''-([1,1'-Biphenyl]-4,4'-diylbis(phenylazanediyl))bis(([1,1'-biphenyl]-2,5-dicarbonitrile)) (6d)

8.1.1 Computed xyz-coordinates of compound 6d (PBE1PBE/6-31+G**) in the gas phase

Inputfile:

%NProcShared=24

%Chk=gauss.chk

%mem=128GB

#p PBE1PBE 6-31+G** pop=full GFInput opt freq

Optimized S_0-geometry:

C 0 -3.520770 -0.388686 0.543010

C 0 -5.728318 0.500606 1.040788

C 0 -2.765165 -0.969469 -0.482410

C 0 -1.390253 -0.788338 -0.531224

C 0 -0.720750 -0.013674 0.429470

C	0	-1.486595	0.554675	1.455428
C	0	-2.862864	0.365378	1.520121
C	0	-5.643624	1.773206	0.436384
C	0	-6.389968	2.831529	0.963705
C	0	-7.247999	2.643707	2.042473
C	0	-7.355414	1.377819	2.614173
C	0	-6.595463	0.322182	2.125749
N	0	-4.926350	-0.569434	0.579119
C	0	-5.450181	-1.889577	0.590071
C	0	-4.792810	2.027263	-0.756357
C	0	-4.709037	-2.952881	1.119093
C	0	-5.233237	-4.242891	1.112297
C	0	-6.504231	-4.488707	0.598594
C	0	-7.245619	-3.427010	0.080488
C	0	-6.724950	-2.137918	0.065753
C	0	-5.143233	1.536688	-2.029557
C	0	-4.344867	1.800183	-3.151404
C	0	-3.196377	2.563826	-3.026082
C	0	-2.849138	3.077234	-1.769894
C	0	-3.643793	2.808943	-0.649992
C	0	0.740260	0.193622	0.362980
C	0	1.528532	0.187020	1.524582
C	0	2.902216	0.370286	1.467536
C	0	3.538813	0.583642	0.238425
C	0	2.762418	0.614964	-0.924371
C	0	1.388294	0.412110	-0.859237
N	0	4.942358	0.763364	0.194640
C	0	5.525891	1.771687	1.010107
C	0	5.662821	0.276420	-0.920371
C	0	4.822255	2.943090	1.313103
C	0	5.404625	3.919260	2.117402
C	0	6.695456	3.749978	2.613361
C	0	7.398849	2.586516	2.302201

C 0	6.820530	1.597805	1.513468
C 0	5.557111	-1.074371	-1.317046
C 0	6.231315	-1.504761	-2.464174
C 0	7.040056	-0.641096	-3.195404
C 0	7.168500	0.682938	-2.781766
C 0	6.479033	1.137666	-1.664770
C 0	4.752012	-2.065511	-0.555688
C 0	3.572231	-2.572116	-1.097418
C 0	2.807282	-3.516359	-0.404803
C 0	3.220111	-3.981503	0.850210
C 0	4.402269	-3.504982	1.391989
C 0	5.167298	-2.557224	0.697071
C 0	-6.355002	0.797194	-2.215343
N 0	-7.342985	0.218236	-2.410852
C 0	-1.671784	3.880125	-1.628602
N 0	-0.717326	4.533375	-1.519468
C 0	6.407795	-2.130756	1.270533
N 0	7.418522	-1.827646	1.756470
C 0	1.588121	-3.992763	-0.985188
N 0	0.597771	-4.368795	-1.462261
H 0	-3.263305	-1.576471	-1.232846
H 0	-0.822522	-1.275354	-1.319441
H 0	-1.000817	1.169675	2.208578
H 0	-3.436139	0.814698	2.326358
H 0	-6.315263	3.808049	0.492176
H 0	-7.832460	3.474929	2.424376
H 0	-8.020223	1.212621	3.457220
H 0	-6.659890	-0.658471	2.587011
H 0	-3.721525	-2.769929	1.530986
H 0	-4.642589	-5.057758	1.521625
H 0	-6.910977	-5.495530	0.596922
H 0	-8.233563	-3.602642	-0.335872
H 0	-7.298031	-1.323577	-0.365504

H 0 -4.642244 1.410249 -4.119276

H 0 -2.574409 2.773171 -3.889763

H 0 -3.353964 3.199339 0.319702

H 0 1.060559 -0.004543 2.486661

H 0 3.497795 0.341128 2.375451

H 0 3.238476 0.811144 -1.881108

H 0 0.802342 0.465348 -1.773069

H 0 3.821346 3.088265 0.918049

H 0 4.846301 4.823352 2.344013

H 0 7.148447 4.514964 3.236979

H 0 8.402338 2.434917 2.689816

H 0 7.363921 0.683680 1.296669

H 0 6.139992 -2.546267 -2.761681

H 0 7.569452 -1.001566 -4.071827

H 0 7.793968 1.373043 -3.340755

H 0 6.559141 2.175880 -1.357998

H 0 3.233205 -2.210381 -2.062207

H 0 2.620699 -4.710649 1.384528

H 0 4.750107 -3.866845 2.353922

SCF Done: E(RPBE1PBE) = -2326.66804199 A.U. after 1 cycles

Zero-point correction= 0.696697 (Hartree/Particle)

Thermal correction to Energy= 0.743747

Thermal correction to Enthalpy= 0.744691

Thermal correction to Gibbs Free Energy= 0.608226

Sum of electronic and zero-point Energies= -2325.971345

Sum of electronic and thermal Energies= -2325.924295

Sum of electronic and thermal Enthalpies= -2325.923351

Sum of electronic and thermal Free Energies= -2326.059816

8.1.2 Computed molecule volume of compound 6d (PBE1PBE/6-31+G, volume) in the gas phase**

Inputfile:

%NProcShared=24

%Chk=gauss.chk

%mem=128GB

#p PBE1PBE 6-31+G** Geom=AllCheck Guess=TCheck SCRF=Check Test GenChk

scf=tight int=finegrid volume

Outcome:

SCF Done: E(RPBE1PBE) = -2326.66809136 A.U. after 8 cycles

Recommended a0 for SCRF calculation = 7.04 angstrom (13.30 bohr)

8.1.3 Computed molecule volume of compound 6d (PBE1PBE/6-31+G**, volume) in cyclohexane

Inputfile:

%NProcShared=24

%Chk=gauss.chk

%mem=128GB

#p PBE1PBE 6-31+G** Geom=AllCheck Guess=TCheck SCRF=Check Test GenChk

scf=tight SCRF=(Dipole,Solvent=CycloHexane,A0=7.04) volume

Outcome:

SCF Done: E(RPBE1PBE) = -2326.66805747 A.U. after 10 cycles

Recommended a0 for SCRF calculation = 6.52 angstrom (2.32 bohr)

8.1.4 Computed molecule volume of compound 6d (PBE1PBE/6-31+G**, volume) in toluene

Inputfile:

%NProcShared=24

%Chk=gauss.chk

%mem=128GB

#p PBE1PBE 6-31+G** Geom=AllCheck Guess=TCheck SCRF=Check Test GenChk

scf=tight SCRF=(Dipole,Solvent=Toluene,A0=7.04) volume

Outcome:

SCF Done: E(RPBE1PBE) = -2326.66806070   A.U. after   11 cycles

Recommended a0 for SCRF calculation = 7.12 angstrom (13.46 bohr)

8.1.5 Computed molecule volume of compound 6d (PBE1PBE/6-31+G**, volume) in 1,4-dioxane

Inputfile:

%NProcShared=24

%Chk=gauss.chk

%mem=128GB

#p PBE1PBE 6-31+G** Geom=AllCheck Guess=TCheck SCRF=Check Test GenChk

scf=tight SCRF=(Dipole,Solvent=1,4-Dioxane,A0=7.04) volume

Outcome:

SCF Done: E(RPBE1PBE) = -2326.66805930   A.U. after   11 cycles

Recommended a0 for SCRF calculation = 7.37 angstrom (13.92 bohr)

8.1.6 Computed molecule volume of compound 6d (PBE1PBE/6-31+G**, volume) ethyl acetate

Inputfile:

%NProcShared=24

%Chk=gauss.chk

%mem=128GB

#p PBE1PBE 6-31+G** Geom=AllCheck Guess=TCheck SCRF=Check Test GenChk

scf=tight SCRF=(Dipole,Solvent=EthylEthanoate,A0=7.04) volume

Outcome:

SCF Done: E(RPBE1PBE) = -2326.66807481   A.U. after   13 cycles

Recommended a0 for SCRF calculation = 7.17 angstrom (13.56 bohr)

8.1.7 Computed molecule volume of compound 6d (PBE1PBE/6-31+G**, volume) dichloromethane

Inputfile:

%NProcShared=24

%Chk=gauss.chk

%mem=128GB

#p PBE1PBE 6-31+G** Geom=AllCheck Guess=TCheck SCRF=Check Test GenChk

scf=tight SCRF=(Dipole,Solvent=Dichloromethane,A0=7.04) volume

Outcome:

SCF Done: E(RPBE1PBE) = -2326.66807872   A.U. after   12 cycles

Recommended a0 for SCRF calculation = 7.33 angstrom (13.86 bohr)

9 References

1 R. Kohlbecher, T. J. J. Müller, *Chem. Eur. J.* **2024**, *30*, e202304119.

2 R. Kohlbecher, T. Lippert, H. Schröder, D. N. Jordan, C. Janiak, T. J. J. Müller, *Eur. J. Org. Chem.* **2026**, *29*, e70312.

3 M. Flörke, T. J. J. Müller, *manuscript in preparation*.

4 M. Flörke, T. J. J. Müller, *manuscript in preparation.*

5 P. Zanello, *Electrochemical and X-ray Structural Aspects of Transition Metal Complexes Containing Redox-Active Ferrocene Ligands*, Wiley-VCH Verlag GmbH, **2007**.

6 M. J. Frisch, G. W. Trucks, H. B. Schlegel, G. E. Scuseria, M. A. Robb, J. R. Cheeseman, G. Scalmani, V. Barone, G. A. Petersson, H. Nakatsuji, X. Li, M. Caricato, A. V. Marenich, J. Bloino, B. G. Janesko, R. Gomperts, B. Mennucci, H. P. Hratchian, J. V. Ortiz, A. F. Izmaylov, J. L. Sonnenberg, D. Williams-Young, F. Ding, F. Lipparini, F. Egidi, J. Goings, B. Peng, A. Petrone, T. Henderson, D. Ranasinghe, V. G. Zakrzewski, J. Gao, N. Rega, G. Zheng, W. Liang, M. Hada, M. Ehara, K. Toyota, R. Fukuda, J. Hasegawa, M. Ishida, T. Nakajima, Y. Honda, O. Kitao, H. Nakai, T. Vreven, K. Throssell, J. J. A. Montgomery, J. E. Peralta, F. Ogliaro, M. J. Bearpark, J. J. Heyd, E. N. Brothers, K. N. Kudin, V. N. Staroverov, T. A. Keith, R. Kobayashi, J. Normand, K. Raghavachari, A. P. Rendell, J. C. Burant, S. S. Iyengar, J. Tomasi, M. Cossi, J. M. Millam, M. Klene, C. Adamo, R. Cammi, J. W. Ochterski, R. L. Martin, K. Morokuma, O. Farkas, J. B. Foresman, D. J. Fox, *Gaussian 16, Revision A.03*, Gaussian, Inc., Wallingford CT, **2016**.

7 S. Maddala, S. Mallick, P. Venkatakrishnan, *J. Org. Chem.* **2017**, *82*, 8958–8972.

8 V. V. Pavlishchuk, A. W. Addison, *Inorg. Chim. Acta* **2000**, *298*, 97–102.

9 N. Elgrishi, K. J. Rountree, B. D. McCarthy, E. S. Rountree, T. T. Eisenhart, J. L. Dempsey, *J. Chem. Educ.* **2018**, 95, 197–206.

10 R. R. Gagne, C. A. Koval and G. C. Lisensky, *Inorg. Chem.* **1980**, 19, 2854–2855.

11 G. A. Mabbott, *J. Chem. Educ.* **1983**, 60, 697.

12 A. J. Bard, L. R. Faulkner, H. S. White, *Electrochemical methods: fundamentals and applications*, John Wiley & Sons, New York, **2022**.

13 J. Hrbac, V. Halouzka, L. Trnkova, J. Vacek *Sensors* **2014**, 14, 13943-13954.

14 C. Reichardt, T. Welton, *Solvents and Solvent Effects in Organic Chemistry, Vol. 4* (Eds.: C. Reichardt, T. Welton), Wiley-VCH, Weinheim, **2010**, 549–589.

15 a) J. G. Kirkwood, *J. Chem. Phys.* **1934**, 2, 351–361. b) L. Onsager, *J. Am. Chem. Soc.* **1936**, 58, 1486–1493. c) M. W. Wong, M. J. Frisch, K. B. Wiberg, *J. Am. Chem. Soc.* **1991**, 113, 4776–4782. d) M. W. Wong, K. B. Wiberg, M. Frisch, *J. Chem. Phys.* **1991**, 95, 8991–8998. e) M. W. Wong, K. B. Wiberg, M. J. Frisch, *J. Am. Chem. Soc.* **1992**, 114, 523–529. f) M. W. Wong, K. B. Wiberg, M. J. Frisch, *J. Am. Chem. Soc.* **1992**, 114, 1645–1652.
